# Electrochemical Synthesis of 1,2-Substituted *N*‑Amido Benzimidazoles by Reduction of Nitroarenes

**DOI:** 10.1021/acs.orglett.5c05349

**Published:** 2026-02-16

**Authors:** Daniel Doellerer, Aaron Schüll, Thomas Weyhermüller, Sebastian B. Beil, Siegfried R. Waldvogel

**Affiliations:** † 28313 Max-Planck-Institute for Chemical Energy Conversion, Department of Electrosynthesis, Stiftstrasse 34−36, 45470 Mülheim an der Ruhr, Germany; ‡ 28313 Max-Planck-Institute for Chemical Energy Conversion, Department of Inorganic Spectroscopy, Stiftstrasse 34−36, 45470 Mülheim an der Ruhr, Germany; § Karlsruhe Institute of Technology, Institute of Biological and Chemical Systems - Functional Molecular Systems (IBCS FMS), 76131 Karlsruhe, Germany

## Abstract

Despite the vast number of reports on benzimidazoles, 1,2-substituted *N*-amido benzimidazoles remain an underrepresented and scarcely accessible compound class with promising pharmacological relevance. We present a safe, reliable electrochemical protocol that provides easy access to those structures. The reaction exhibits broad functional group tolerance and affords the target compounds in ≤89% yields.

## Graphical abstract

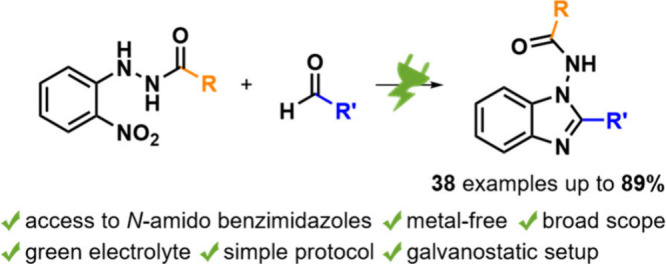

About 59% of small molecule drugs approved by the Food and Drug Administration (FDA) contain at least one nitrogen-based heterocycle, where newly approved derivatives involve as much as 82% *N*-heterocycles.^1,2^ Among these active pharmaceutical ingredients (APIs), benzimidazole moieties are common structural motifs that lead to a variety of therapeutic applications.^2–5^ Examples of drugs containing at least one benzimidazole are the well-known proton pump inhibitors such as omeprazole,^6^ carbendazim, a commonly used fungicide,^7^ second-generation antihistamine astemizole,^8^ and telmisartan, an antihypertensive API.^3,9^ All FDA-approved benzimidazole-based drugs are substituted at position 2, and almost half of them (46%) are substituted in position 1, as well.^1^ The conventional synthesis of benzimidazoles usually involves a condensation of *o*-phenylenediamines with different carbonyl components in a cyclo-condensation reaction.^10,11^ Alternative transformations usually start with *o*-nitroaniline. Metal catalysts and/or stoichiometric amounts of reducing agents are often used cogenerating large amounts of waste, which leads to bad atom economy.^11^ Synthetic approaches toward *N*-amido benzimidazoles have only scratched the surface. Very few syntheses by direct reaction of *N*-amino benzimidazoles and acid anhydrides or acid chlorides have been reported, whereby the scope was limited to alkyl-substituted benzimidazoles (Scheme 1, middle).^12–14^ However, those few examples of *N*-amido benzimidazoles displayed high biological reactivity as protein arginine methyltransferase (PRMT) 5 or carbonic anhydrase VA inhibitors, which are usually upregulated in tumors or leukemia (see Figure 1).^14,15^ In contrast, aryl-substituted *N*-amido benzimidazoles have so far been reported only as minor byproducts during the synthesis of 1,2,4-benzotriazines (Scheme 1, top)^16^ or when using the expensive and radioactive lanthanoid samarium.^17^

**Figure 1:**
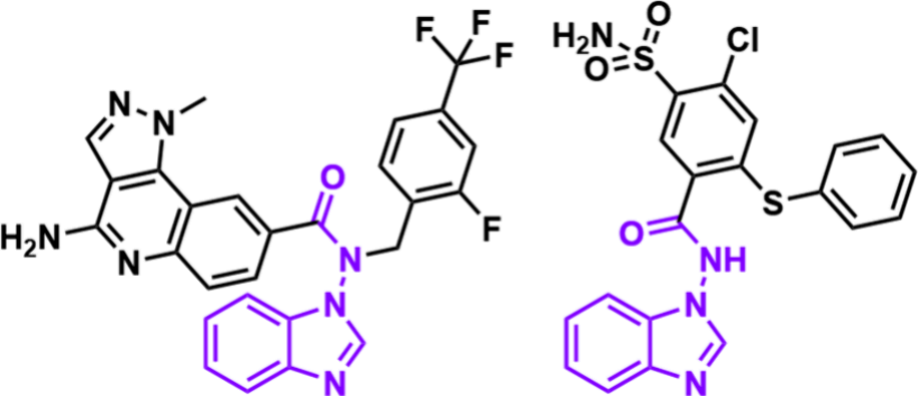
Structures of *N*-amido benzimidazole-based PRMT5 (left) and CA VA (right) inhibitors.

**Scheme 1:**
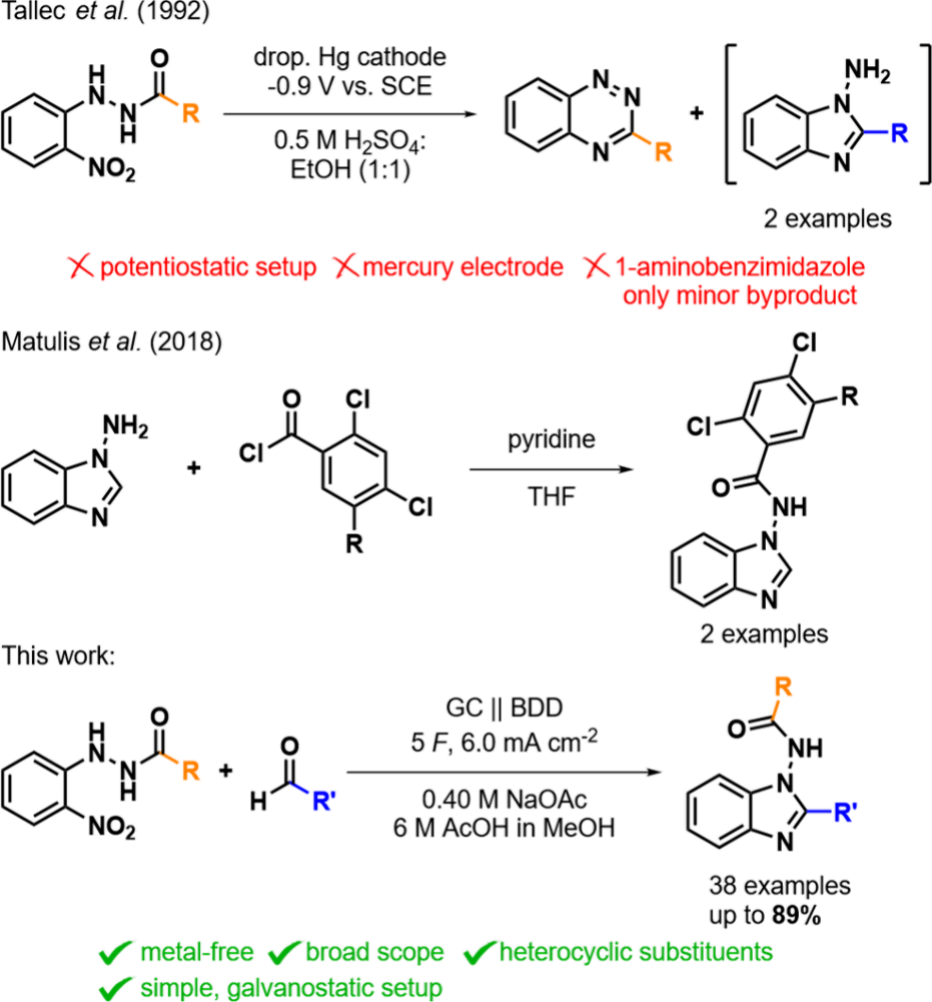
Methods for the Synthesis of *N*-Amino Benzimidazoles (electrochemical, top) and *N*-Amido Benzimidazoles (*N*-Acylation, Middle, and Electrochemical, Bottom)^a^

a Abbreviations: GC, glassy carbon; BDD, boron-doped diamond.

Electro-organic synthesis is a valuable alternative to traditional chemistry, circumventing those disadvantages.^18–22^ The use of electric current as a traceless oxidizing or reducing agent circumvents the formation of reagent waste. Furthermore, electrochemical reactions are often conducted under mild conditions, increasing functional group tolerance and saving energy by means of avoiding heating.^21,23^ Moreover, electro-organic transformations are considered to be inherently safe, since switching off the electrical current ceases the reaction almost immediately and thermal runaway reactions can be circumvented.^24,25^

Our group established the electrochemical reduction of nitro groups as a promising and easy-to-use method for the synthesis of a large variety of *N*-heterocycles;^26,27^ among others, five-membered heterocycles like triazoles^28^ and 2,1-benzisoxazoles^29,30^ have been synthesized. Since *N*-oxy and *N*-hydroxy heterocycles have shown interesting pharmacological properties and are often discussed as metabolites,^31^ our group has reported a number of transformations with conditions, specifically chosen to yield these motifs. Among others, quinoline *N*-oxides,^32^ 1-hydroxy quinazolinones,^33^ 4-hydroxy-benzo­[*e*]-1,2,4-thiadiazine-1,1-dioxides,^34^ 1-hydroxyquin-ol-4-ones,^35^ and cyclic hydroxamic acids^36^ have been synthesized in this way.

Recently, the electro-organic synthesis of benzimidazoles has been realized by some groups in an entirely different way. In 2021, a method by Xu et al. was reported to synthesize benzimidazoles by electrochemical dehydrogenative cyclization of amidines.^37^ Another approach for the electrochemical synthesis of benzimidazoles utilizing the oxidation of an alcohol for the generation of the carbonyl component is needed for the reaction while reducing a nitro group on the cathode.^38,39^ To the best of our knowledge, an electrochemical synthesis of *N*-amido benzimidazoles has not been reported. However, in 1992, Tallec et al. observed traces of 1-amino benzimidazoles as byproducts, while investigating the electrochemical synthesis of 1,2,4-benzotriazines from *o*-nitro phenyl hydrazides (Scheme 1, top).^40^

We present the first electrochemical synthesis of 2-substituted *N*-amido benzimidazoles from *o*-nitrophenyl hydrazides in a simple-to-operate and metal-free galvanostatic setup. The method tolerates a broad variety of functional groups such as double bonds, halo substituents, nitriles, alcohols, and other heterocycles, giving access to the desired benzimidazoles substituted in position 2 (Scheme 1 bottom). To optimize the electrolysis conditions, *o*-nitro phenylhydrazide **1a** was chosen as a test substrate to react with benzaldehyde to form benzimidazole **2a**. **1a** was synthesized in a single step from 2-nitrophenylhydrazine and 4-methylbenzoyl chloride (see the Supporting Information for experimental details).^41^ To avoid the oxidation of the used aldehyde in the reaction, a divided cell setup was chosen for the electrochemical reduction of the nitro group. An applied charge slightly above the theoretical value of 4 *F* proved to be beneficial for the reaction, leading to the optimized conditions of 5 *F* with 6 mA cm^–2^ using a glassy carbon (GC) anode and boron-doped diamond (BDD) cathode,^42,43^ reacting 2 equiv of aldehyde with the respective hydrazide in a sodium acetate (0.4 M) and acetic acid (6 M) in a MeOH electrolyte solution, affording a 70% yield of **2a** (Table 1, entry 1). The optimized conditions are the result of combining two alterations discussed in entries 2–5 in Table 1, where the reaction was limited by the cell voltage reaching the upper limit of the galvanostat used. For this reason, this multifactor interaction was investigated in a design of experiments (DoE) together with the electrochemical parameters and the concentrations of the starting materials (see the Supporting Information for more details).^44,45^ The DoE provided benzimidazole **2a** in 70% yield, which could not be further improved. Using 4 *F* in sodium acetate (0.27 M) and acetic acid (6 M) in a MeOH electrolyte solution utilizing just 1 equiv of aldehyde affords 52% and 60% yields of **2a** (Table 1, entries 2 and 3, respectively). Increasing the current density results in a 53% yield at 10.0 mA cm^–2^, while decreasing the current density decreases the yield further to 44% (Table 1, entries 4 and 5). Different cathode materials were screened (Table 1, entries 6–9).^46^ Lead cathodes afford **2a** in 65% yield but were subjected to severe corrosion,^47^ which could not be suppressed. Using diammonium salt additives previously shown to stabilize lead electrodes had no visible effect (see the Supporting Information for details).^48^ Different electrolyte systems were investigated, as well (see the Supporting Information). Of the electrolyte systems tested, a system of acetic acid and sodium acetate in methanol was the only system affording **2a** in a sufficient yield while sulfuric acid in methanol was the only other electrolyte to afford molecule **2a**, be in a yield of only 5% (Table 1, entries 1 and 2, and the Supporting Information). An investigation of the reaction temperature indicated that decreasing the reaction temperature had no significant impact on the reaction and afforded molecule **2a** in 69% yield. Increasing the temperature decreased the yield of **2a** to only 61% (Table 1, entries 10 and 11). Furthermore, the reaction does not proceed when no electricity is applied (entry 12) or acids/esters are used as condensation partners instead of aldehydes (investigated with 4-fluorobenzoic acid and methyl 4-fluorobenzoate due to the ease of analysis).

**Table 1:**
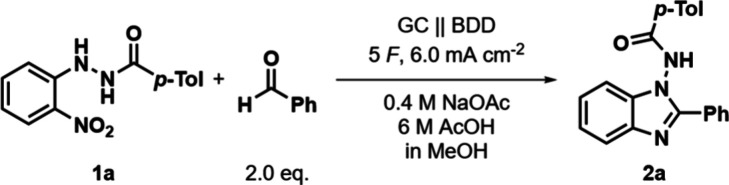
Screening of Electrolysis Parameters for the Synthesis of **2a** [Table-fn t1fn66]

entry	deviation from the optimized conditions	yield (%)
1	none	70
2	4 *F*, 5.0 mA cm^–2^, 0.27 M NaOAc, 4.37 M AcOH, 1 equiv of aldehyde	52
3	4.5 *F*, 5.0 mA cm^–2^	60
4	4 *F*, 2.5 mA cm^–2^	44
5	4 F, 10.0 mA cm^–2^	53
6	GC cathode	49
7	graphite cathode	42
8	Pb cathode^a^	65
9	CuSn7Pb15 cathode	27
10	entry 1 at 15 °C	69
11	entry 1 at 30 °C	61
12	no electricity	–

a With these optimized conditions, the reaction was carried out on a 0.375 mmol scale on other substrates and with different aldehydes to expand the scope to a broad variety of functional groups and structural motifs.

b Subject to severe cathodic corrosion.

Hydrazides **1a–q** were synthesized and subjected to the electrochemical protocol (see the Supporting Information for details). The substitution position at the benzo ring made a negligible difference as synthesizing toluoyl hydrazides **2a–c** gave yields between 64% and 71%. Benzohydrazide **2d** and *para*-substituted benzohydrazides **2e** and **2f** were obtained in yields of 62% (R = H), 41% (R = OMe), and 72% (R = ^*t*^Bu), respectively. Different substitution patterns were tested for halo substituents, and **2g**–**j** were obtained in yields between 52% and 67%. Naphthoyl hydrazide **2k** was obtained in 58% yield. We were able to isolate alkyl-substituted products **2l** and **2m** in yields of 52% and 58%, respectively. Phenylacethydrazide **2n** was obtained in 42% yield. The method even tolerated a double bond in **1o** to yield **2o** in 73% isolated yield (see Figure 2 for the structures). However, functional groups that are not stable under reductive conditions (e.g., oxalates) and pyridines, pyrroles, or sterically overly demanding substrates on the aldehyde pose a limitation for this method (see the Supporting Information).

**Figure 2:**
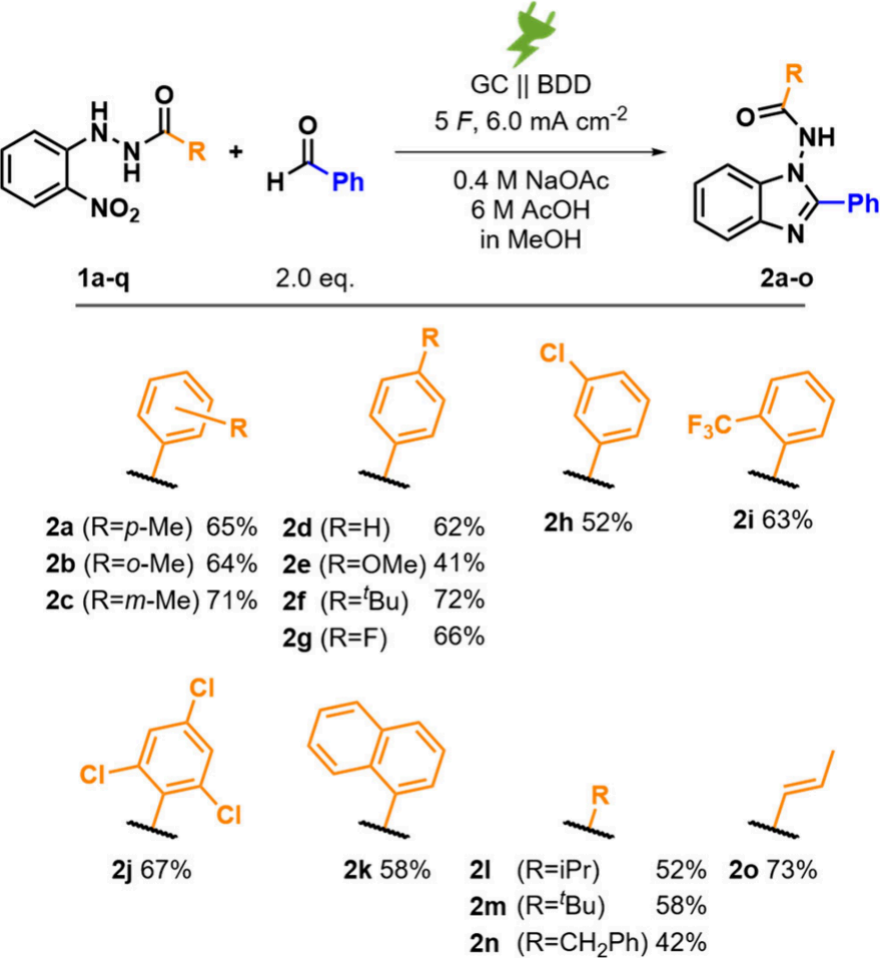
Synthesis of 1-amido-2-phenyl benzimidazoles **2a–o** and isolated yields.

Next, a range of different aldehydes were reacted with **1a** to afford the corresponding 2-substituted benzimidazoles. The transformation displayed broad functional group tolerance without compromising the reactivity. *p*-Methyl-substituted (**3a**) and halo-substituted (**3b–d**) compounds, amenable for further postfunctionalization, and electron-withdrawing esters (**3e**) were obtained in yields ranging from 73% to 78%. Fluorobenzaldehydes substituted at any ring position delivered *N*-amido benzimidazoles **3f–h** in 63–71% yields. The reaction tolerated among other functionalities free phenols at the *meta* position and *m*-methoxy and *meta*-nitrile benzimidazoles (**3i–k**) were isolated in 72%, 89%, and 81% yields, respectively, with the *m*-methoxy derivative having the highest overall yield. Additionally, the *ortho*-substituted methoxy benzaldehyde afforded benzimidazole **3l** in 68% yield. Heteroatomic aldehydes, among others, thiophene-2/3-carboxaldehyde and furfural, respectively, 5-methyl furfural, were well tolerated, resulting in *N*-amido benzimidazoles **3m–p** in 68–77% yields. The reaction also tolerated more complex aldehydes like vanillin or (1*R*)-(−)-myrtenal, resulting in **3q** or **3r**, respectively, although with slightly lower yields of 58% for both compared to the other used aldehydes. Last, the reaction was performed with formaldehyde and alkyl-substituted aldehydes, affording *N*-amido benzimidazoles **3s–w** with 28% and 40–85% yields, whereas phenylacetaldehyde gave the lowest yield with 40% of the alkyl group-bearing aldehydes (see Figure 3 for structures). A 30-fold scale-up experiment (3.05 g (11.25 mmol) compared to 101.7 mg (0.375 mmol)) was performed using **1a** and benzaldehyde (see the Supporting Information) to show the applicability of this method. Desired compound **2a** was isolated in 74% yield, in comparison to 65% in the small scale reaction, which could be attributed to more efficient convection and handling on a larger scale.

**Figure 3:**
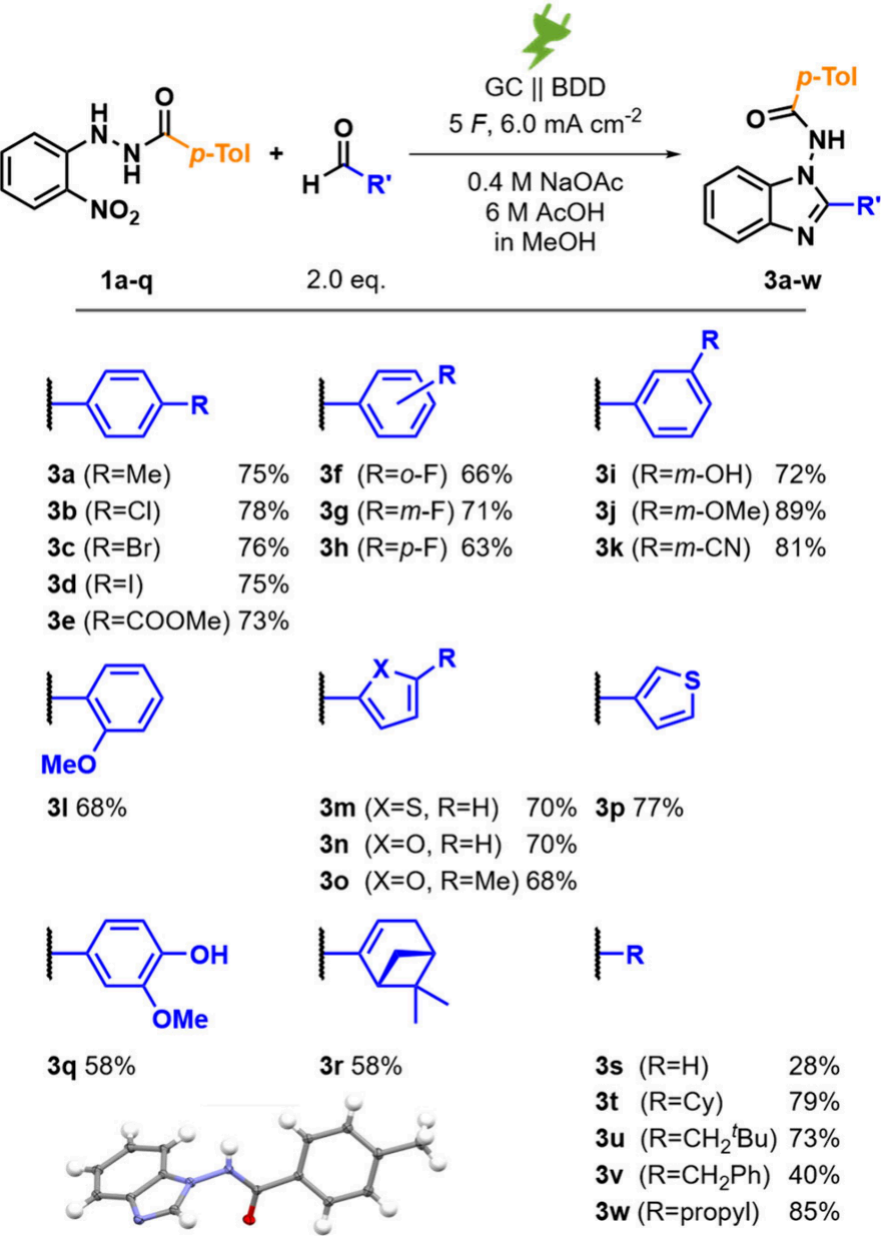
Synthesis of 2-substituted *N*-amido benzimidazoles **3a–w** and isolated yields and molecular structure of benzimidazole **3s** determined by X-ray analysis of a single crystal (ORTEP image ellipsoid at 50% probability; CCDC 2514730; bottom left).

The structure of molecule **3s** was unequivocally confirmed by X-ray single-crystal diffraction and matches the NMR data (see the bottom left of Figure 3 and the Supporting Information).

We established the first electrochemical synthesis to previously difficult-to-access 1,2-aryl-substituted *N*-amido benzimidazoles under mild conditions in a simple and metal-free key step. The broad applicability of this method was demonstrated with almost 40 examples displaying isolated yields of ≤89%. The method tolerates a myriad of functional groups on both rings of the benzimidazole, including halo substituents, nitriles, alcohols, double bonds, alkyl chains, and even heterocycles at position 2. It represents the first general route to this moiety. The easy access and reliable protocol result in these promising scaffolds with potential biological activity that inherit the potential of postfunctionalization at the amine functionality, opening the door for future pharmaceutical investigations. Furthermore, this electrosynthesis was reliably scaled up.

## Supplementary Material



## Data Availability

The data underlying this study are available in the published article and its Supporting Information.

## Notes

Experimental details, spectral data, and scale-up (PDF)
