# Supplementary material for: Electrochemical Synthesis of 1,2-Substituted N‑Amido Benzimidazoles by Reduction of Nitroarenes
Source: Org Lett. 2026 Feb 16;28(8):2591–5. doi: 10.1021/acs.orglett.5c05349 (PMC12954844; doi:10.1021/acs.orglett.5c05349)
Supplement: Supplementary file 1 [file ol5c05349_si_001.pdf]

Supporting Information

Electrochemical Synthesis of 1,2-Substituted *N*-Amido Benzimidazoles by Reduction of Nitroarenes

Daniel Doellerer,[a†] Aaron Schüll,[a†] Thomas Weyhermüller,[b] Sebastian B. Beil,[a] Siegfried R. Waldvogel[a,c]*

[a] Max-Planck-Institute for Chemical Energy Conversion, Department of Electrosynthesis, Stiftstr. 34–36, 45470 Mülheim an der Ruhr (Germany).

[b] Max-Planck-Institute for Chemical Energy Conversion, Department of Inorganic Spectroscopy, Stiftstr. 34–36, 45470 Mülheim an der Ruhr (Germany).

[c] Karlsruhe Institute of Technology, Institute of Biological and Chemical Systems − Functional Molecular Systems (IBCS FMS), 76131 Karlsruhe, (Germany

* E-mail: siegfried.waldvogel@cec.mpg.de

† These authors contributed equally to this work

Table of Contents

General Remarks **S3**

Experimental Procedures **S5**

Screening Studies **S6**

Compounds **S11**

Scale-Up **S35**

References **S36**

Appendix **S37**

Crystallographic Data S37

NMR Spectra S38

General Remarks

Chemicals were purchased from commercial sources, Sigma-Aldrich, Fluorochem, TCI, BLDpharm and used without further purification. Unless stated otherwise, all reactions were carried out under ambient conditions. Analytical thin layer chromatography (TLC) was performed on silica gel 60 F254 (Merck KGaA, Darmstadt, Germany).

All NMR spectra of the synthesized compounds can be found in the appendix of the Supporting Information.

Flash Column Chromatography

Preparative column chromatography was performed on prepacked puriFlash™ silica columns (15 µm, PF-15SIHP-F0025 (PF-15SIHC-F0120 for the scale-up) Interchim, Montlucon Cedex, France) using a puriFlash™-System (puriFlash™ XS 520 Plus, Interchim, Montlucon Cedex, France) with an integrated UV detector. Synthesis grade cyclohexane/ethyl acetate and HPLC grade CH_2Cl_2/MeOH were used as eluents without further purification.

Gas Chromatography

Analysis of crude reaction mixtures and purified products were performed using a GC-2030 (Shimadzu, Kyoto, Japan) equipped with a flame ionization detector (FID) and a quartz capillary column Zebron ZB-5plus (Phenomenex Inc., Torrance, California, USA) with following specification: length of 30 m, inner diameter of 0.25 mm and a stationary phase ((5%-phenyl)dimethylsiloxane) of 0.25 µm thickness. Hydrogen was used as carrier gas with a constant velocity of 40 cm/s. Measurements were performed at an injector temperature of 290 °C (split ratio of 35 °C), a detector temperature of 320 °C, and a FID temperature of 335 °C (starting at 50 °C, hold for 1 min, and heating to 320 °C with a ramp of 30 °C/min for 3 min and 17.5 °C/min 10 min, hold for 7 min – total run time 21 min)

Nuclear Magnetic Resonance (NMR) Spectroscopy

NMR spectra were recorded on a Bruker AVANCE III HD 500 or Bruker Ascend Evo 400 MHz NMR spectrometer, respectively with a Bruker Prodigy probe 80 K or a Bruker iProbe BBFO room temperature probe (Bruker BioSpin GmbH, Rheinstetten, Germany) at 25 °C unless stated otherwise. Chemical shifts are reported in δ-scale as parts per million [ppm] (multiplicity, coupling constant (J), number of protons), referenced to the residual solvent signal (DMSO-d_6: δ = 2.50 for ^{1}H and 39.5 for ^{13}C{^{1}H}) and thereby stated relatively to TMS. The resonance multiplicity is indicated as s = singlet, d = doublet, t = triplet, m = multiplet and combinations thereof. The coupling constant values J are given in hertz [Hz]. Structural assignments were made with additional information from gCOSY, gHSQC, and gHMBC experiments.

High Resolution Mass Spectrometry (HRMS)

High resolution mass spectra were recorded via electrospray-ionization (ESI+, ESI-) using a Q Exactive™ mass spectrometer (Thermo Fischer Scientific™, Waltham, USA) and via electron-ionization (EI) and chemical ionization (CI) using a Q Exactive™ GC Orbitrap™ GC-MS/MS (Thermo Fischer Scientific™, Waltham, USA) equipped with an XTI-5 or a MS90 ZB-1 HT column (Restek GmbH, Bad Homburg v. d. Höhe, Deutschland and Phenomenex Inc., Torrance, United States).

X-ray Crystallography

Single crystal measurements were carried out on a Bruker D8 Venture Kappa Diffractometer equipped with an IµS3 diamond Mo-source (50 kV, 1.4 mA; Mo Kα, λ = 0.71073 Å), Oxford cryostream 800 cooler, Incoatec Helios mirror optics and a Photon III detector.

Electrochemical Setup

Parameter screenings of the electrolytic conditions were performed in 6 mL divided PTFE cells using an IKA Screening System Package (*IKA-Werke GmbH & Co. KG*, Staufen, Germany) and a multichannel power supply HMP4040 (*Rohde & Schwarz*, München, Germany) as power source with electrodes the size of 70 mm × 10 mm × 3 mm). This set-up was previously developed by our lab.[1] The electrode surfaces were cleaned prior use.[2] Glassy carbon (GC, SIGRADUR™ G, *HTW*, Thierhaupten, Germany) was rinsed with acetone and methanol and wiped with a paper towel. Boron-doped diamond (BDD, DIACHEM™, 15 µm diamond layer on silicon support, *CONDIAS GmbH*, Itzehoe, Germany) was conditioned by electrolyzing as anode in 20% aqueous sulfuric acid (10 C/cm², 10 mA/cm²) and subsequently rinsed with water. Isostatic graphite (C_{gr}, Sigrafine™ V2100, *SGL Carbon*, Bonn, Germany), lead and leaded-bronze (CuSn7Pb15) were wet-polished with sandpaper (grade 1000 + 1200, *Bosch*, Stuttgart, Germany), rinsed with acetone and the abrasion was wiped off with a paper towel until the latter was not stained anymore.

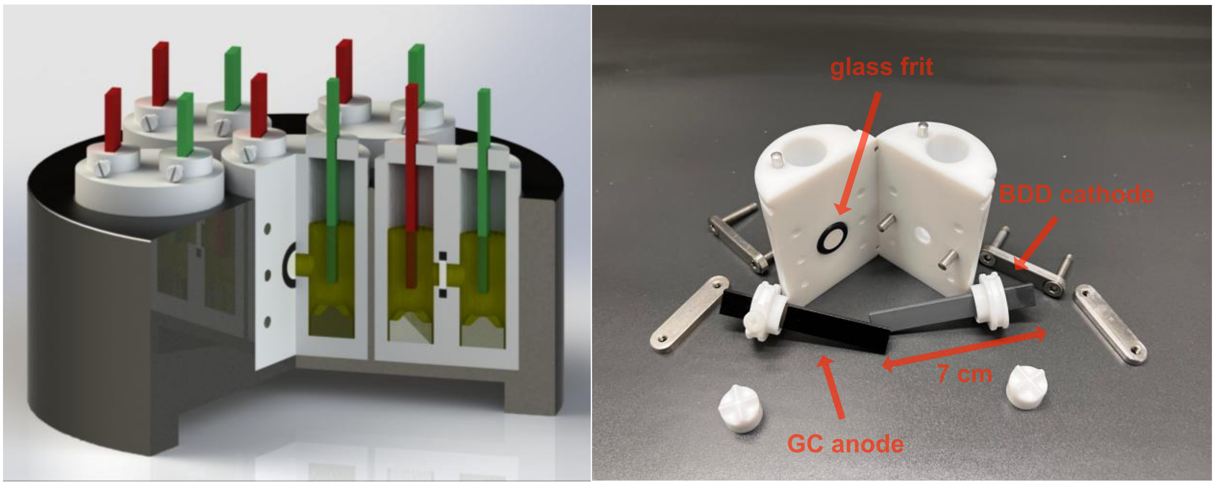

***Figure S1**: Exemplary screening block for divided PTFE cells (left) and opened divided cell with glass frit and mounted electrodes (GC and BDD; right).*

Experimental Procedures

General Procedure for the Synthesis of *N'*-(2-Nitrophenyl) Hydrazides (GPI)

2-nitrophenyl hydrazine hydrochloride (10 mmol, 1.0 eq.) was dispersed in CH_2Cl_2 at 0 °C (ice/water) in a 50 mL round bottom flask. After the slow addition of pyridine (22 mmol, 2.2 eq.) the respective acid chloride (11 mmol, 1.1 eq.) was added dropwise and the reaction mixture was stirred at room temperature overnight. The organic solvent was removed under reduced pressure after the reaction reached completion, the crude washed with aqueous HCl (1 M) and extracted with EtOAc (3x50 mL). The combined organic layers were dried over $MgSO_4$ and the solvent removed under reduced pressure. The crude product was purified by recrystallization (EtOH or toluene).

General Procedure for Screening and Optimization Studies (GPII)

In a 6 mL divided PTFE screening cell equipped with a glass frit, the respective aldehyde was dissolved in 5 mL of electrolyte solution in an AcOH/MeOH mixture at room temperature in the cathode compartment. The anode compartment was filled with an equal volume of electrolyte solution. Under stirring, the hydrazide was added to the cathode compartment and dispersed in the mixture. The mixture was subjected to galvanostatic electrolysis using electrodes with a relevant surface area of 2.25 cm^2 (surface: 70 mm x 10 mm, immersion depth: 22.5 mm). After electrolysis, dodecylbenzene (30 µL) and ethyl acetate (3 mL) were added to the cathode compartment while ethyl acetate (3 mL) was added to the anode compartment to keep the level of liquid in both compartments equal. The reaction mixture was stirred for 2 minutes and 15 drops of the catholyte were filtered over a silica plug. A gas chromatogram of the mixture was measured and the amount of product determined by internal calibration (see below for details).

General Procedure for Synthesis of 1,2-Substituted *N*-Amido Benzimidazoles (GPIII)

In a 6 mL divided PTFE screening cell equipped with a glass frit, the respective aldehyde was dissolved in 5 mL of electrolyte solution in an AcOH/MeOH mixture at room temperature in the cathode compartment. The anode compartment was filled with an equal volume of electrolyte solution. Under stirring (cross stirring bar 400 rpm), the hydrazide was added to the cathode compartment and dispersed in the mixture. The mixture was subjected to galvanostatic electrolysis using electrodes with a relevant surface area of 2.25 cm^2 (surface: 70 mm x 10 mm, immersion depth: 22.5 mm). After electrolysis, the solvent of the catholyte was removed under reduced pressure and the crude product purified via flash chromatography (SiO_2, 6% EtOAc in Cy to 94% EtOAc (2.5 column volumes (CV) to 8%; 3.5 CV to 17.5%; 5.5 CV to 54.5%; 3 CV to 94% and hold 4.5 CV at 94%) or CH_2Cl_2 to 20% MeOH in CH_2Cl_2 (hold 2.5 CV at 0%; 3.5 CV to 2%; 5.5 CV to 5%; hold 1.5 CV at 5%; 3 CV to 10%; 4.5 CV to 15%; 2 CV to 20% and hold 1 CV at 20%)).

Screening Studies: Synthesis of 1*H*-4-Methyl-*N*-(2-phenyl-benzo[*d*]imidazol-1-yl)benzamide (2a)

The optimization of the nitroreduction of **1a** was performed with a 'one variable at a time' (OVAT) as well as a “Design of Experiment” (DoE) approach.[3] Experiments were carried out according to **GPII** and evaluated via GC. An exemplary GC measurement is shown in **Figure S2** to illustrate the quantification. The yield was determined against the standard of *n*-dodecylbenzene (30 µL). Parameters as well as the visualization of the internal calibration are shown in **Figure S3**.

Table S1: *Screening experiments for the optimization of the synthesis of* ***2a***. [a]*Yields were determined by GC measurements using n-dodecylbenzene as internal standard.*

Entry	Anode ||Cathode	*J* [mA cm^{-2}]	*Q* [*F*]	T [°C]	Eq. ald.	Supporting electrolyte	Solvent	Yield [%][a]
**1**	GC || BDD	0.0	0.0	rt	3.0	NaOAc (0.27 M)	MeOH/AcOH (4.37 M)	-
**2**	GC || BDD	5.0	4.0	rt	3.0	NaOAc (0.27 M)	MeOH/AcOH (4.37 M)	52
**3**	GC || GC	5.0	4.0	rt	3.0	NaOAc (0.27 M)	MeOH/AcOH (4.37 M)	49
**4**	GC || C_{gr}	5.0	4.0	rt	3.0	NaOAc (0.27 M)	MeOH/AcOH (4.37 M)	42
**5**	BDD || BDD	5.0	4.0	rt	3.0	NaOAc (0.27 M)	MeOH/AcOH (4.37 M)	52
**6**	BDD || GC	5.0	4.0	rt	3.0	NaOAc (0.27 M)	MeOH/AcOH (4.37 M)	49
**7**	GC || Pb	5.0	4.0	rt	3.0	NaOAc (0.27 M)	MeOH/AcOH (4.37 M)	65
**8**	GC || CuSn7Pb15	5.0	4.0	rt	3.0	NaOAc (0.27 M)	MeOH/AcOH (4.37 M)	27
**9**	GC || BDD	5.0	4.0	rt	3.0	NaOAc (0.27 M)	MeOH/AcOH (6.47 M)	60
**10**	GC || BDD	5.0	4.0	rt	3.0	NaOAc (0.27 M)	MeOH/AcOH (8.60 M)	62
**11**	GC || BDD	5.0	4.0	rt	3.0	NaOAc (0.27 M)	MeOH/AcOH (10.77 M)	62
**12**	GC || BDD	5.0	4.0	rt	3.0	NaOAc (0.27 M)	MeOH/AcOH (12.90 M)	62
**13**	GC || BDD	5.0	4.0	rt	3.0	NaOAc (0.15 M)	MeOH/AcOH (4.37 M)	55
**14**	GC || BDD	5.0	4.0	rt	3.0	NaOAc (0.50 M)	MeOH/AcOH (4.37 M)	47
**15**	GC || BDD	2.5	4.0	rt	3.0	NaOAc (0.27 M)	MeOH/AcOH (4.37 M)	44
**16**	GC || BDD	10.0	4.0	rt	3.0	NaOAc (0.27 M)	MeOH/AcOH (4.37 M)	53
**17**	GC || BDD	5.0	4.0	rt	3.0	-	MeOH/H_2SO_4 (0.5 M)	5
**18**	GC || BDD	5.0	4.0	rt	3.0	HCOONa (0.5 M)	MeOH/HCOOH (5 M)	-
**19**	GC || BDD	5.0	4.0	rt	3.0	HCOONa (0.5 M)	MeOH/HCOOH (10 M)	-

**20**	GC || BDD	5.0	4.0	rt	3.0	HCOONa (0.5 M)	HCOOH	-
**21**	GC || BDD	5.0	4.0	rt	3.0	NaOAc (0.27 M)	AcOH	-
**22**	GC || BDD	5.0	4.0	rt	1.0	NaOAc (0.27 M)	MeOH/AcOH (4.37 M)	55
**23**	GC || BDD	5.0	4.0	rt	2.0	NaOAc (0.27 M)	MeOH/AcOH (4.37 M)	53
**24**	GC || BDD	5.0	4.0	rt	4.0	NaOAc (0.27 M)	MeOH/AcOH (4.37 M)	55
**25**	GC || BDD	5.0	4.0	rt	5.0	NaOAc (0.27 M)	MeOH/AcOH (4.37 M)	54
**26**	GC || BDD	5.0	4.5	rt	3.0	NaOAc (0.27 M)	MeOH/AcOH (4.37 M)	60
**27**	GC || BDD	5.0	5.0	rt	3.0	NaOAc (0.27 M)	MeOH/AcOH (4.37 M)	62
**28**	GC || BDD	6.0	5.0	15	2.0	NaOAc (0.40 M)	MeOH/AcOH (6 M)	69
**29**	**GC || BDD**	**6.0**	**5.0**	**rt**	**2.0**	**NaOAc (0.40 M)**	**MeOH/AcOH (6 M)**	**70**
**30**	GC || BDD	6.0	5.0	30	2.0	NaOAc (0.40 M)	MeOH/AcOH (6 M)	61
**31**	GC || BDD	6.0	5.0	40	2.0	NaOAc (0.40 M)	MeOH/AcOH (6 M)	57
**32**	GC || BDD	6.0	5.0	50	2.0	NaOAc (0.40 M)	MeOH/AcOH (6 M)	54
**33**	GC || BDD	6.0	5.0	rt	2.0	Bu_4OAc (0.20 M)	MeOH/AcOH (6 M)	56

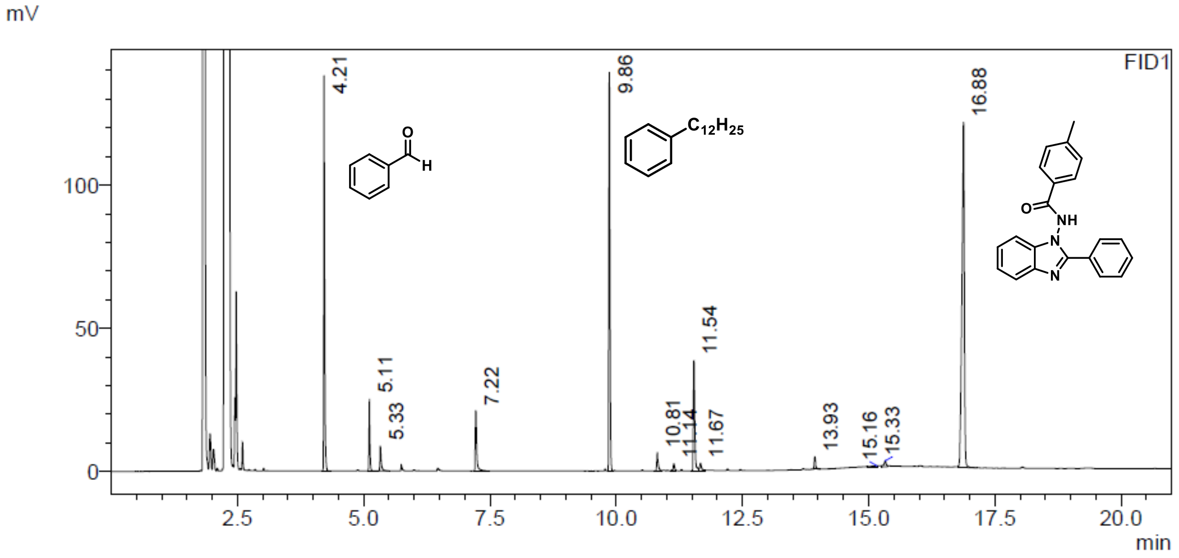

***Figure S2**: Crude GC measurement of a reaction mixture during the reaction optimization with signals for benzaldehyde (4.21 min), n-dodecylbenzene as internal standard (9.86 min) and **2a** (16.88 min).*

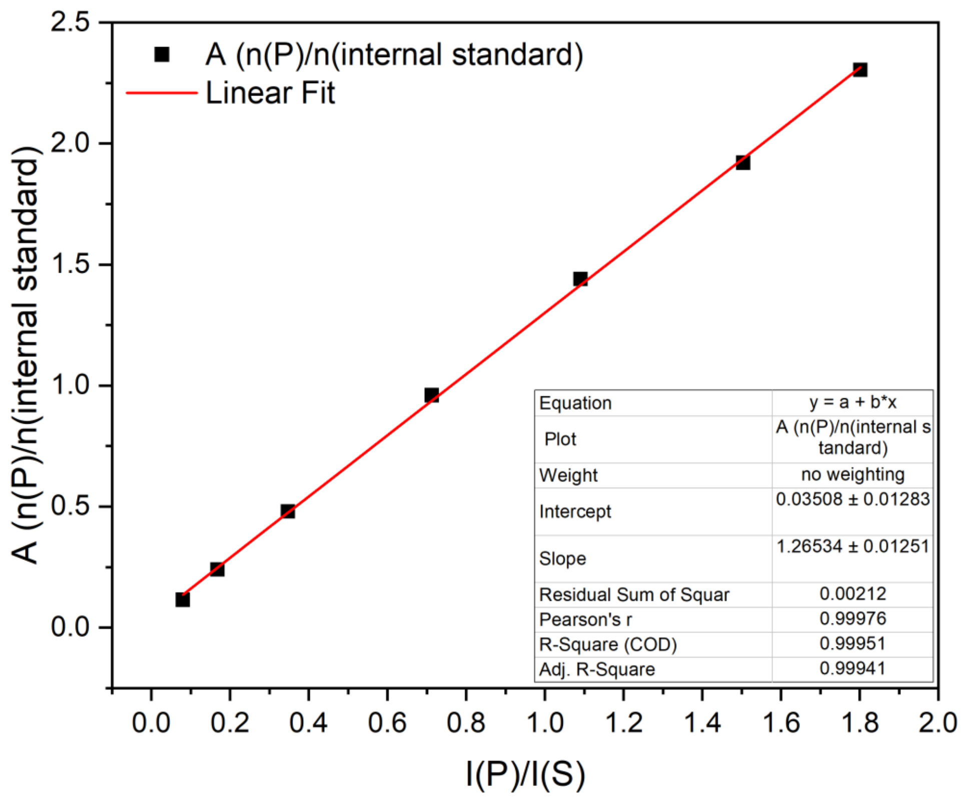

***Figure S3**: Calibration curve used for GC calibration. n-Dodecylbenzene (30 µL) was used as internal standard.*

***Table S2**: Experiments conducted and parameters investigated in the Design of Experiments during the optimization of the synthesis of **2a**.*

Std	Run	Factor 1 A: *j* [mA cm^{-2}]	Factor 2 B: *Q* [*F*]	Factor 3 C: *n*(SM) [mmol]	Factor 4 D: eq. Ald.	Factor 5 E: *c*(NaOAc) [mol L^{-1}]	Factor 6 F: *c*(AcOH) [mol L^{-1}]	Response 1 Yield [%]
32	1	8	5.5	0.5	3.0	0.5	8	61.7
24	2	8	5.5	0.25	3.0	0.3	4	59.8
17	3	4	4.5	0.25	3.0	0.3	8	64.4
33	4	6	5.0	0.375	2.0	0.4	6	66.6
31	5	8	5.5	0.5	3.0	0.5	8	61.3
16	6	8	5.5	0.5	1.0	0.5	4	53.3
15	7	8	5.5	0.5	1.0	0.5	4	57.1
30	8	4	5.5	0.5	3.0	0.3	8	56.7
21	9	4	5.5	0.25	3.0	0.5	4	62.4
12	10	8	4.5	0.5	1.0	0.3	8	59.6
20	11	8	4.5	0.25	3.0	0.5	8	54.6
3	12	8	4.5	0.25	1.0	0.5	4	49.0
11	13	8	4.5	0.5	1.0	0.3	8	58.9
10	14	4	4.5	0.5	1.0	0.5	8	56.8
14	15	4	5.5	0.5	1.0	0.3	4	54.0

19	16	8	4.5	0.25	3.0	0.5	8	61.2
9	17	4	4.5	0.5	1.0	0.5	8	53.6
23	18	8	5.5	0.25	3.0	0.3	4	62.5
25	19	4	4.5	0.5	3.0	0.5	4	57.8
34	20	6	5.0	0.375	2.0	0.4	6	57.2
35	21	6	5.0	0.375	2.0	0.4	6	62.2
1	22	4	4.5	0.25	1.0	0.3	4	52.4
27	23	8	4.5	0.5	3.0	0.3	4	58.0
26	24	4	4.5	0.5	3.0	0.5	4	51.2
2	25	4	4.5	0.25	1.0	0.3	4	55.9
29	26	4	5.5	0.5	3.0	0.3	8	62.9
8	27	8	5.5	0.25	1.0	0.3	8	47.8
4	28	8	4.5	0.25	1.0	0.5	4	51.2
22	29	4	5.5	0.25	3.0	0.5	4	57.7
18	30	4	4.5	0.25	3.0	0.3	8	60.9
28	31	8	4.5	0.5	3.0	0.3	4	58.9
13	32	4	5.5	0.5	1.0	0.3	4	54.9
36	33	6	5.0	0.375	2.0	0.4	6	64.9
7	34	8	5.5	0.25	1.0	0.3	8	47.7
6	35	4	5.5	0.25	1.0	0.5	8	53.0
5	36	4	5.5	0.25	1.0	0.5	8	49.2

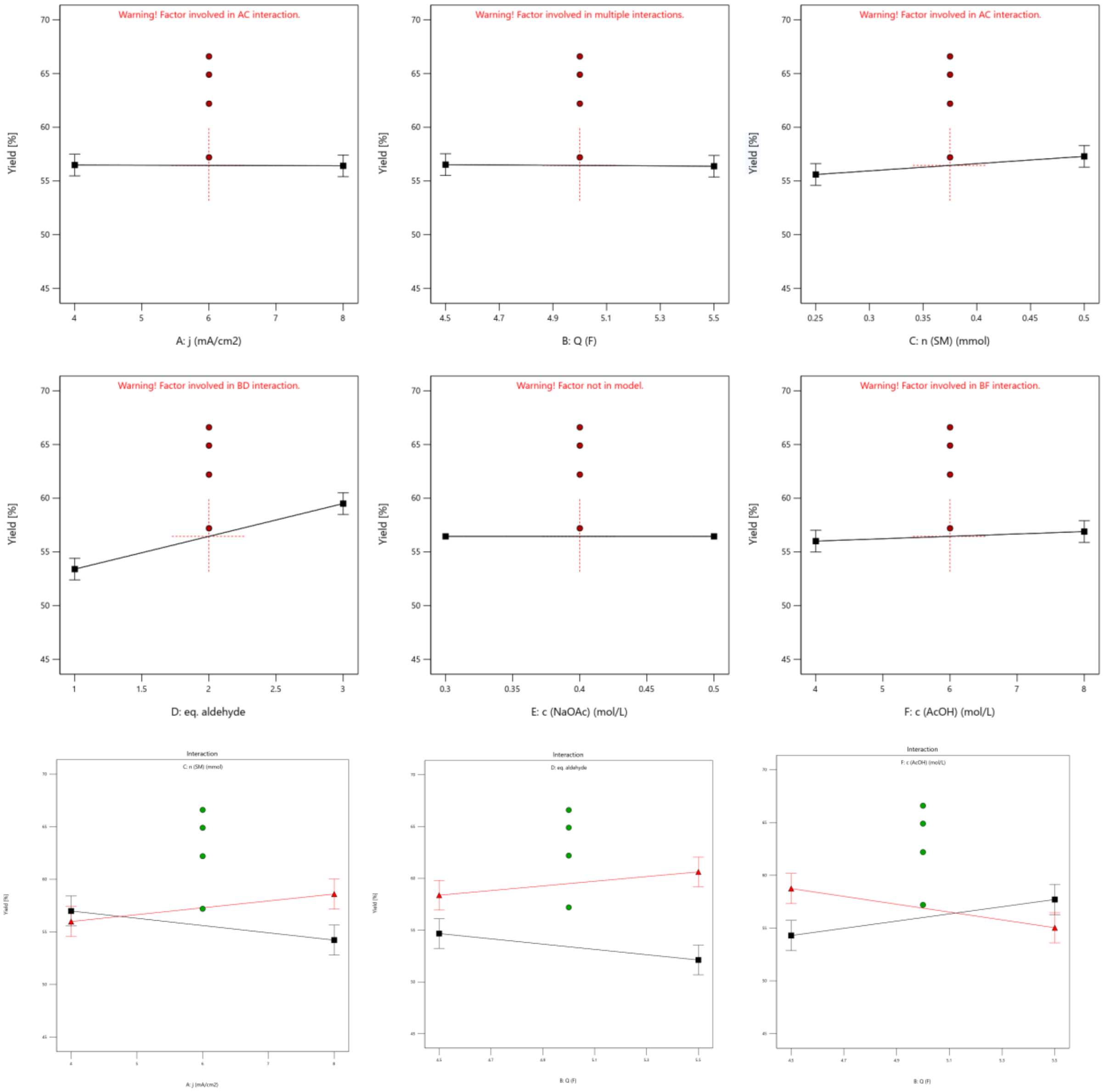

Figure S4*: Main Effect plots (top and middle) and two-factor interactions regarding the yield.*

Compounds

4-Methyl-*N*'-(2-nitrophenyl)benzohydrazide (**1a**)

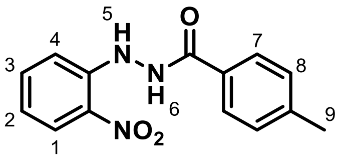

1a

$C_{14}H_{13}N_3O_3$
Mw = 271.28 g/mol

Synthesis of hydrazide **1a** was carried out according to **GPI** using (2-nitrophenyl)hydrazine hydrochloride (3.79 g, 20.00 mmol, 1.0 eq.) and 4-methylbenzoyl chloride (2.91 mL, 3.40 g, 22.00 mmol, 1.1 eq.). After recrystallization from ethanol **1a** was obtained as orange platelets (4.00 g, 14.75 mmol, 74%).

^{1}H NMR (500 MHz, DMSO-*d*$_6$) δ [ppm]: 10.73 (s, 1H, *H*-6), 9.42 (s, 1H, *H*-5), 8.12 (dd, *J* = 8.5, 1.5 Hz, 1H, *H*-1), 7.89 – 7.83 (m, 2H, *H*-7), 7.58 (ddd, *J* = 8.4, 6.9, 1.5 Hz, 1H, *H*-3), 7.37 – 7.31 (m, 2H, *H*-8), 7.16 (dd, *J* = 8.4, 1.1 Hz, 1H, *H*-4), 6.88 (ddd, *J* = 8.5, 6.9, 1.1 Hz, 1H, *H*-2), 2.39 (s, 3H, *H*-9).
^{13}C{^{1}H} NMR (126 MHz, DMSO-*d*$_6$) δ [ppm]: 166.0, 145.6, 142.1, 136.5, 131.8, 129.5, 129.1, 127.6, 125.9, 117.9, 114.9, 21.1.
HRMS (ESI-): *m/z* calculated for $C_{14}H_{12}N_3O_3^-$ [M-H]$^-$ 270.0884; found 270.0887.
m.p. (EtOH): 187.8–188.2 °C.

2-Methyl-*N*'-(2-nitrophenyl)benzohydrazide (1b)

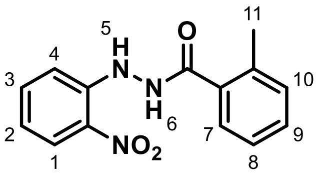

1b

$C_{14}H_{13}N_3O_3$
Mw = 271.28 g/mol

Synthesis of hydrazide **1b** was carried out according to **GPI** using (2-nitrophenyl)hydrazine hydrochloride (1.90 g, 10.00 mmol, 1.0 eq.) and 2-methylbenzoyl chloride (1.43 mL, 1.70 g, 11.00 mmol, 1.1 eq.). After recrystallization from ethanol **1b** was obtained as red crystals (1.00 g, 3.68 mmol, 37%).

^{1}H NMR (400 MHz, DMSO-*d*$_6$) δ [ppm]: 10.52 (s, 1H, *H*-6), 9.44 (s, 1H, *H*-5), 8.13 (dd, *J* = 8.5, 1.5 Hz, 1H, *H*-1), 7.64 (ddd, *J* = 8.6, 6.9, 1.6 Hz, 1H, *H*-3), 7.58 (dd, *J* = 7.8, 1.5 Hz, 1H, *H*-7), 7.42 (td, *J* = 7.4, 1.5 Hz, 1H, *H*-9), 7.35 – 7.28 (m, 2H, *H*-8, *H*-10), 7.23 (dd, *J* = 8.6, 1.3 Hz, 1H, *H*-4), 6.90 (ddd, *J* = 8.5, 6.9, 1.3 Hz, 1H, *H*-2), 2.42 (s, 3H, *H*-11).
^{13}C{^{1}H} NMR (101 MHz, DMSO-*d*$_6$) δ [ppm]: 168.5, 145.4, 136.6, 136.1, 134.3, 131.8, 130.7, 130.2, 127.6, 125.9, 125.6, 117.9, 114.7, 19.4.
HRMS (ESI-): *m/z* calculated for $C_{14}H_{12}N_3O_3^-$ [M-H]$^-$ 270.0884; found 270.0887.
m.p. (EtOH): 190.1–190.5 °C.

3-Methyl-*N*'-(2-nitrophenyl)benzohydrazide (1c)

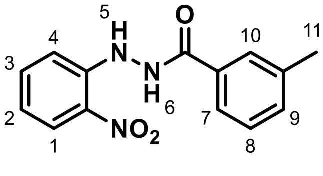

1c

$C_{14}H_{13}N_3O_3$
Mw = 271.28 g/mol

Synthesis of hydrazide **1c** was carried out according to **GPI** using (2-nitrophenyl)hydrazine hydrochloride (1.90 g, 10.00 mmol, 1.0 eq.) and 3-methylbenzoyl chloride (1.45 mL, 1.70 g, 11.00 mmol, 1.1 eq.). After recrystallization from ethanol **1c** was obtained as yellow solid (1.66 g, 6.12 mmol, 61%).

^{1}H NMR (400 MHz, DMSO-*d*$_6$) δ [ppm]: 10.74 (s, 1H, *H*-6), 9.41 (s, 1H, *H*-5), 8.13 (dd, *J* = 8.5, 1.7 Hz, 1H, *H*-1), 7.80 – 7.70 (m, 2H, *H*-7, *H*-10), 7.59 (td, *J* = 8.7, 1.7 Hz, 1H, *H*-3), 7.47 – 7.36 (m, 2H, *H*-8, *H*-9), 7.18 (dd, *J* = 8.7, 1.3 Hz, 1H, *H*-4), 6.89 (ddd, *J* = 8.5, 6.9, 1.3 Hz, 1H, *H*-2), 2.39 (s, 3H, *H*-11).
^{13}C{^{1}H} NMR (101 MHz, DMSO-*d*$_6$) δ [ppm]: 166.2, 145.4, 137.9, 136.4, 132.6, 132.3, 131.8, 128.4, 128.0, 125.8, 124.6, 117.9, 114.9, 20.9.
HRMS (ESI-): *m/z* calculated for $C_{14}H_{12}N_3O_3^-$ [M-H]$^-$ 270.0884; found 270.0888.
m.p. (EtOH): 148.0–148.4 °C.

***N*'-(2-Nitrophenyl)benzohydrazide (1d)**

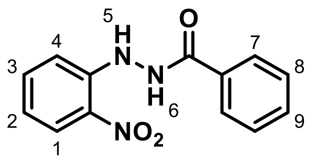

1d
$C_{13}H_{11}N_3O_3$
Mw = 257.25 g/mol

Synthesis of hydrazide **1d** was carried out according to **GPI** using (2-nitrophenyl)hydrazine hydrochloride (1.90 g, 10.00 mmol, 1.0 eq.) and benzoyl chloride (1.28 mL, 1.55 g, 11.00 mmol, 1.1 eq.). After recrystallization from ethanol **1d** was obtained as a yellow solid (1.89 g, 7.33 mmol, 73%).

^{1}H NMR (500 MHz, DMSO-*d_6*) *δ* [ppm]: 10.81 (s, 1H, *H*-6), 9.45 (s, 1H, *H*-5), 8.13 (dd, *J* = 8.5, 1.5 Hz, 1H, *H*-1), 7.99 – 7.93 (m, 2H, *H*-7), 7.66 – 7.55 (m, 2H, *H*-3, *H*-9), 7.57 – 7.52 (m, 2H, *H*-8), 7.18 (dd, *J* = 8.6, 1.3 Hz, 1H, *H*-4), 6.89 (ddd, *J* = 8.5, 6.9, 1.3 Hz, 1H, *H*-2).
^{13}C{^{1}H} NMR (126 MHz, DMSO-d_6) *δ* [ppm]: 166.1, 145.5, 136.5, 132.4, 132.1, 131.8, 128.6, 127.6, 125.9, 117.9, 114.9.
HRMS (ESI-): *m/z* calculated for $C_{13}H_{10}N_3O_3^-$ [M-H]$^-$ 256.0728; found 256.0731.
m.p. (EtOH): 171.9–172.3 °C.

4-Methoxy-*N*'-(2-nitrophenyl)benzohydrazide (1e)

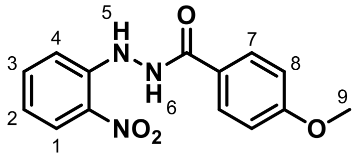

1e
$C_{14}H_{13}N_3O_4$
Mw = 287.28 g/mol

Synthesis of hydrazide **1e** was carried out according to **GPI** using (2-nitrophenyl)hydrazine hydrochloride (3.79 g, 20.00 mmol, 1.0 eq.) and 4-methoxybenzoyl chloride (2.98 mL, 3.75 g, 22.00 mmol, 1.1 eq.). After recrystallization from ethanol **1e** was obtained as red needles (5.19 g, 18.05 mmol, 90%).

^{1}H NMR (500 MHz, DMSO-*d_6*) *δ* [ppm]: 10.67 (s, 1H, *H*-6), 9.41 (s, 1H, *H*-5), 8.12 (dd, *J* = 8.5, 1.5 Hz, 1H, *H*-1), 7.97 – 7.90 (m, 2H, *H*-7), 7.58 (ddd, *J* = 8.7, 7.0, 1.4 Hz, 1H, *H*-3), 7.16 (dd, *J* = 8.7, 1.3 Hz, 1H, *H*-4), 7.10 – 7.04 (m, 2H, *H*-8), 6.88 (ddd, *J* = 8.5, 7.0, 1.3 Hz, 1H, *H*-2), 3.84 (s, 3H, *H*-9).
^{13}C{^{1}H} NMR (126 MHz, DMSO-*d_6*) *δ* [ppm]: 165.6, 162.2, 145.7, 136.5, 131.8, 129.5, 125.8, 124.4, 117.8, 114.9, 113.8, 55.5.
HRMS (ESI-): *m/z* calculated for $C_{14}H_{12}N_3O_4^-$ [M-H]$^-$ 286.0833; found 286.0837.
m.p. (EtOH): 180.1–180.5 °C.

4-(*tert*-Butyl)-*N*'-(2-nitrophenyl)benzohydrazide (1f)

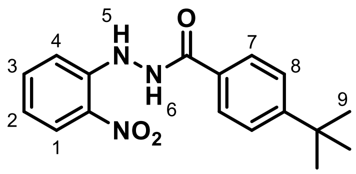

1f
$C_{17}H_{19}N_3O_3$
Mw = 313.36 g/mol

Synthesis of hydrazide **1f** was carried out according to **GPI** using (2-nitrophenyl)hydrazine hydrochloride (1.90 g, 10.00 mmol, 1.0 eq.) and 4-(*tert*-butyl)benzoyl chloride (2.15 mL, 2.16 g, 11.00 mmol, 1.1 eq.). After recrystallization from ethanol **1f** was obtained as yellow crystals (2.62 g, 8.36 mmol, 84%).

^{1}H NMR (500 MHz, DMSO-*d_6*) *δ* [ppm]: 10.74 (s, 1H, *H*-6), 9.43 (s, 1H, *H*-5), 8.13 (dd, *J* = 8.5, 1.5 Hz, 1H, *H*-1), 7.93 – 7.87 (m, 2H, *H*-7), 7.61 – 7.52 (m, 3H, *H*-3, *H*-8), 7.16 (dd, *J* = 8.7, 1.3 Hz, 1H, *H*-4), 6.89 (ddd, *J* = 8.5, 6.9, 1.3 Hz, 1H, *H*-2), 1.32 (s, 9H, *H*-9).
^{13}C{^{1}H} NMR (126 MHz, DMSO-d_6) *δ* [ppm]: 166.0, 155.0, 145.5, 136.5, 131.8, 129.5, 127.4, 125.9, 125.4, 117.9, 114.9, 34.8, 30.9.
HRMS (ESI-): *m/z* calculated for $C_{17}H_{18}N_3O_3^-$ [M-H]$^-$ 312.1354; found 312.1359.
m.p. (EtOH): 172.9–173.3 °C.

4-Fluoro-N'-(2-nitrophenyl)benzohydrazide (1g)

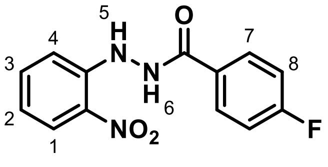

1g

$C_{13}H_{10}FN_3O_3$

Mw = 275.24 g/mol

Synthesis of hydrazide **1g** was carried out according to **GPI** using (2-nitrophenyl)hydrazine hydrochloride (1.90 g, 10.00 mmol, 1.0 eq.) and 4-fluorobenzoyl chloride (1.30 mL, 1.74 g, 11.00 mmol, 1.1 eq.). After recrystallization from ethanol **1g** was obtained as red platelets (1.43 g, 5.18 mmol, 52%).

^{1}H NMR (500 MHz, DMSO-*d*$_6$) δ [ppm]: 10.83 (s, 1H, *H*-6), 9.44 (s, 1H, *H*-5), 8.13 (dd, *J* = 8.5, 1.6 Hz, 1H, *H*-1), 8.06 – 8.00 (m, 2H, *H*-7), 7.59 (ddd, *J* = 8.6, 6.9, 1.6 Hz, 1H, *H*-3), 7.43 – 7.34 (m, 2H, *H*-8), 7.18 (dd, *J* = 8.6, 1.3 Hz, 1H, *H*-4), 6.89 (ddd, *J* = 8.5, 6.9, 1.3 Hz, 1H, *H*-2).

^{13}C{^{1}H} NMR (126 MHz, DMSO-*d*$_6$) δ [ppm]: 165.2, 164.4 (d, *J* = 249.6 Hz), 145.4, 136.5, 131.8, 130.3 (d, *J* = 9.2 Hz), 128.8 (d, *J* = 3.0 Hz), 125.9, 118.0, 115.6 (d, *J* = 22.0 Hz), 114.9.

^{19}F NMR (470 MHz, DMSO-*d*$_6$) δ [ppm]: -108.0 (tt, *J* = 9.4, 5.5 Hz).

HRMS (ESI-): *m/z* calculated for $C_{13}H_9FN_3O_3^-$ $[M-H]^-$ 274.0633; found 274.0637.

m.p. (EtOH): 191.2–191.6 °C.

3-Chloro-*N*'-(2-nitrophenyl)benzohydrazide (1h)

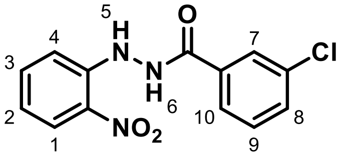

1h

$C_{13}H_{10}ClN_3O_3$

Mw = 291.69 g/mol

Synthesis of hydrazide **1h** was carried out according to **GPI** using (2-nitrophenyl)hydrazine hydrochloride (1.90 g, 10.00 mmol, 1.0 eq.) and 3-chlorobenzoyl chloride (1.41 mL, 1.93 g, 11.00 mmol, 1.1 eq.). After recrystallization from ethanol **1h** was obtained as orange crystals (1.49 g, 5.10 mmol, 51%).

^{1}H NMR (500 MHz, DMSO-*d*$_6$) δ [ppm]: 10.91 (s, 1H, *H*-6), 9.45 (s, 1H, *H*-5), 8.13 (dd, *J* = 8.5, 1.5 Hz, 1H, *H*-1), 8.00 (dd, *J* = 1.9, 1.9 Hz, 1H, *H*-7), 7.94 – 7.88 (m, 1H, *H*-10), 7.69 (ddd, *J* = 8.0, 1.9, 1.0 Hz, 1H, *H*-8), 7.62 – 7.55 (m, 2H, *H*-3, *H*-9), 7.21 (dd, *J* = 8.6, 1.3 Hz, 1H, *H*-4), 6.90 (ddd, *J* = 8.5, 6.9, 1.3 Hz, 1H, *H*-2).

^{13}C{^{1}H} NMR (126 MHz, DMSO-*d*$_6$) δ [ppm]: 164.9, 145.3, 136.6, 134.4, 133.4, 131.9, 131.9, 130.7, 127.4, 126.4, 125.9, 118.1, 115.0.

HRMS (ESI+): *m/z* calculated for $C_{13}H_{11}ClN_3O_3^+$ $[M+H]^+$ 292.0484 (^{35}Cl); found 292.0482.

m.p. (EtOH): 168.9–169.3 °C.

_N_'-(2-Nitrophenyl)-2-(trifluoromethyl)benzohydrazide (1i)

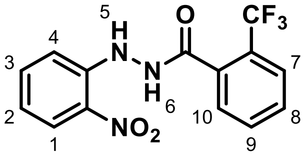

1i
$C_{14}H_{10}F_3N_3O_3$
Mw = 325.25 g/mol

Synthesis of hydrazide **1i** was carried out according to **GPI** using (2-nitrophenyl)hydrazine hydrochloride (0.38 g, 2.00 mmol, 1.0 eq.) and 2-(trifluoromethyl)benzoyl chloride (0.32 mL, 0.46 g, 2.20 mmol, 1.1 eq.). After recrystallization from ethanol **1i** was obtained as yellow needles (0.37 g, 1.13 mmol, 57%).

^{1}H NMR (500 MHz, DMSO-d_6) δ [ppm]: 10.79 (s, 1H, *H*-6), 9.44 (s, 1H, *H*-5), 8.14 (dd, *J* = 8.5, 1.5 Hz, 1H, *H*-1), 7.88 (d, *J* = 7.9 Hz, 1H, *H*-7), 7.87 – 7.80 (m, 2H, *H*-9, *H*-10), 7.75 (ddd, *J* = 7.9, 6.8, 0.9 Hz, 1H, *H*-8), 7.65 (ddd, *J* = 8.6, 6.9, 1.5 Hz, 1H, *H*-3), 7.20 (dd, *J* = 8.6, 1.3 Hz, 1H, *H*-4), 6.92 (ddd, *J* = 8.5, 6.9, 1.3 Hz, 1H, *H*-2).
^{13}C{^{1}H} NMR (126 MHz, DMSO-d_6) δ [ppm]: 166.5, 145.1, 136.5, 133.7, 132.6, 131.8, 130.7, 129.3, 126.6, 126.5, 126.3, 125.9, 118.1, 114.5.
^{19}F NMR (470 MHz, DMSO-d_6) δ [ppm]: -57.7
HRMS (ESI-): *m/z* calculated for $C_{14}H_9F_3N_3O_3^-$ [M-H]$^-$ 324.0602; found 324.0605.
m.p. (EtOH): 175.9–176.3 °C.

2,4,6-Trichloro-_N_'-(2-nitrophenyl)benzohydrazide (1j)

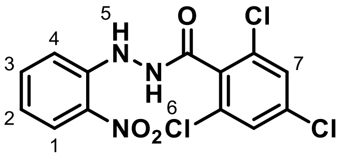

1j
$C_{13}H_8Cl_3N_3O_3$
Mw = 360.58 g/mol

Synthesis of hydrazide **1j** was carried out according to **GPI** using (2-nitrophenyl)hydrazine hydrochloride (0.95 g, 5.00 mmol, 1.0 eq.) and 2,4,6-trichlorobenzoyl chloride (0.86 mL, 1.34 g, 5.50 mmol, 1.1 eq.). After recrystallization from ethanol **1j** was obtained as yellow needles (0.20 g, 0.56 mmol, 11%).

^{1}H NMR (500 MHz, DMSO-d_6) δ [ppm]: 11.07 (s, 1H, *H*-6), 9.38 (s, 1H, *H*-5), 8.14 (dd, *J* = 8.5, 1.5 Hz, 1H, *H*-1), 7.86 (s, 2H, *H*-7), 7.73 – 7.64 (m, 1H, *H*-3), 7.38 (dd, *J* = 8.6, 1.2 Hz, 1H, *H*-4), 6.99 – 6.91 (m, 1H, *H*-2).
^{13}C{^{1}H} NMR (126 MHz, DMSO-d_6) δ [ppm]: 162.7, 144.5, 136.5, 135.4, 133.2, 132.7, 132.2, 128.3, 126.0, 118.6, 114.9.
HRMS (ESI-): *m/z* calculated for $C_{13}H_7Cl_3N_3O_3^-$ [M-H]$^-$ 357.9559 (^{35}Cl); found 357.9564.
m.p. (EtOH): 227.3–227.7 °C.

_N_'-(2-Nitrophenyl)-1-naphthohydrazide (1k)

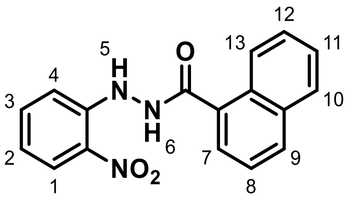

1k
$C_{17}H_{13}N_3O_3$
Mw = 307.31 g/mol

Synthesis of hydrazide **1k** was carried out according to GPI using (2-nitrophenyl)hydrazine hydrochloride (1.90 g, 10.00 mmol, 1.0 eq.) and 1-naphthoyl chloride (1.66 mL, 2.10 g, 11.00 mmol, 1.1 eq.). After recrystallization from ethanol **1k** was obtained as a yellow solid (1.16 g, 3.78 mmol, 38%).

^{1}H NMR (400 MHz, DMSO-d_6) δ [ppm]: 10.77 (s, 1H, *H*-6), 9.60 (s, 1H, *H*-5), 8.36 – 8.27 (m, 1H, *H*-13), 8.16 (dd, *J* = 8.5, 1.5 Hz, 1H, *H*-1), 8.11 (dd, *J* = 8.3, 1.1 Hz, 1H, *H*-9), 8.05 – 8.00 (m, 1H, *H*-10), 7.88 (dd, *J* = 7.0, 1.2 Hz, 1H, *H*-7), 7.72 – 7.56 (m, 4H, *H*-3, *H*-8, *H*-11, *H*-12), 7.32 (dd, *J* = 8.6, 1.3 Hz, 1H, *H*-4), 6.92 (ddd, *J* = 8.5, 7.0, 1.3 Hz, 1H, *H*-2).
^{13}C{^{1}H} NMR (101 MHz, DMSO-d_6) δ [ppm]: 168.1, 145.4, 136.6, 133.1, 132.0, 131.8, 130.7, 129.9, 128.3, 127.0, 126.5, 126.0, 125.9, 125.3, 125.0, 117.9, 114.7.
HRMS (ESI-): *m/z* calculated for $C_{17}H_{12}N_3O_3^-$ [M-H]$^-$ 306.0884; found 306.0887.
m.p. (EtOH): 231.4–231.8 °C.

N'-(2-Nitrophenyl)isobutyrohydrazide (1l)

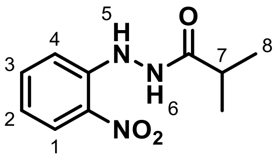

1l
$C_{10}H_{13}N_3O_3$
Mw = 223.23 g/mol

Synthesis of hydrazide **1l** was carried out according to **GPI** using (2-nitrophenyl)hydrazine hydrochloride (1.90 g, 10.00 mmol, 1.0 eq.) and isobutyryl chloride (1.15 mL, 1.17 g, 11.00 mmol, 1.1 eq.). After recrystallization from toluene **1l** was obtained as orange needles (1.60 g, 7.16 mmol, 72%).

^{1}H NMR (400 MHz, DMSO-d_6) δ [ppm]: 10.10 (d, *J* = 1.5 Hz, 1H, *H*-6), 9.18 (d, *J* = 1.5 Hz, 1H, *H*-5), 8.09 (dd, *J* = 8.5, 1.6 Hz, 1H, *H*-1), 7.59 (ddd, *J* = 8.6, 6.9, 1.6 Hz, 1H, *H*-3), 7.04 (dd, *J* = 8.6, 1.3 Hz, 1H, *H*-4), 6.86 (ddd, *J* = 8.5, 6.9, 1.3 Hz, 1H, *H*-2), 2.54 (hept, *J* = 6.8 Hz, 1H, *H*-7), 1.11 (d, *J* = 6.8 Hz, 6H, *H*-8).
^{13}C{^{1}H} NMR (101 MHz, DMSO-d_6) δ [ppm]: 175.7, 145.5, 136.5, 131.6, 125.8, 117.7, 114.5, 32.2, 19.2.
HRMS (ESI-): *m/z* calculated for $C_{10}H_{12}N_3O_3^-$ [M-H]$^-$ 222.0884; found 222.0885.
m.p. (Toluene): 154.8–155.2 °C.

N'-(2-Nitrophenyl)pivalohydrazide (1m)

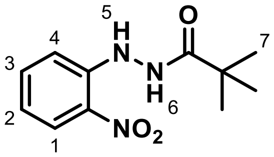

1m
$C_{11}H_{15}N_3O_3$
Mw = 237.26 g/mol

Synthesis of hydrazide **1m** was carried out according to **GPI** using (2-nitrophenyl)hydrazine hydrochloride (1.90 g, 10.00 mmol, 1.0 eq.) and pivaloyl chloride (1.36 mL, 1.33 g, 11.00 mmol, 1.1 eq.). After recrystallization from toluene **1m** was obtained as a yellow solid (1.48 g, 6.23 mmol, 62%).

^{1}H NMR (400 MHz, DMSO-d_6) δ [ppm]: 9.93 (s, 1H, *H*-6), 9.12 (s, 1H, *H*-5), 8.09 (dd, *J* = 8.5, 1.5 Hz, 1H, *H*-1), 7.59 (ddd, *J* = 8.6, 6.9, 1.6 Hz, 1H, *H*-3), 7.02 (dd, *J* = 8.6, 1.2 Hz, 1H, *H*-4), 6.86 (ddd, *J* = 8.5, 7.0, 1.3 Hz, 1H, *H*-2), 1.22 (s, 9H, *H*-7).
^{13}C{^{1}H} NMR (101 MHz, DMSO-d_6) δ [ppm]: 176.9, 145.9, 136.4, 131.8, 125.8, 117.8, 114.6, 37.6, 27.1.
HRMS (ESI-): *m/z* calculated for $C_{11}H_{14}N_3O_3^-$ [M-H]$^-$ 236.1041; found 236.1043.
m.p. (Toluene): 141.1–141.5 °C.

N'-(2-Nitrophenyl)-2-phenylacetohydrazide (1n)

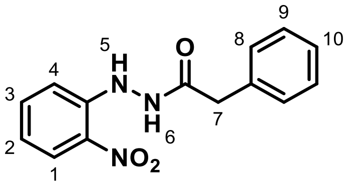

1n
$C_{14}H_{13}N_3O_3$
Mw = 271.28 g/mol

Synthesis of hydrazide **1n** was carried out according to **GPI** using (2-nitrophenyl)hydrazine hydrochloride (1.90 g, 10.00 mmol, 1.0 eq.) and 2-phenylacetyl chloride (1.45 mL, 1.70 g, 11.00 mmol, 1.1 eq.). After recrystallization from ethanol **1n** was obtained as orange needles (1.49 g, 5.47 mmol, 55%).

^{1}H NMR (400 MHz, DMSO-d_6) δ [ppm]: 10.37 (s, 1H, *H*-6), 9.24 (s, 1H, *H*-5), 8.08 (dd, *J* = 8.5, 1.6 Hz, 1H, *H*-1), 7.53 (ddd, *J* = 8.5, 6.9, 1.6 Hz, 1H, *H*-3), 7.38 – 7.31 (m, 4H, *H*-8, *H*-9), 7.28 – 7.23 (m, 1H, *H*-10), 7.01 (dd, *J* = 8.5, 1.3 Hz, 1H, *H*-4), 6.85 (ddd, *J* = 8.4, 6.9, 1.3 Hz, 1H, *H*-2), 3.58 (s, 2H, *H*-7).
^{13}C{^{1}H} NMR (101 MHz, DMSO-d_6) δ [ppm]: 169.8, 145.2, 136.3, 135.3, 131.7, 129.1, 128.3, 126.6, 125.8, 117.8, 114.5, 40.1.
HRMS (ESI-): *m/z* calculated for $C_{14}H_{12}N_3O_3^-$ [M-H]$^-$ 270.0884; found 270.0888.
m.p. (EtOH): 179.8–180.2 °C.

(*E*)-*N*'-(2-Nitrophenyl)but-2-enehydrazide (1o)

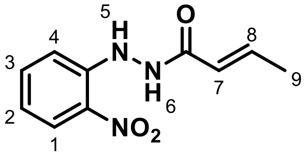

1o

$C_{10}H_{11}N_3O_3$

Mw = 221.22 g/mol

Synthesis of hydrazide **1o** was carried out according to **GPI** using (2-nitrophenyl)hydrazine hydrochloride (1.90 g, 10.00 mmol, 1.0 eq.) and crotonic acid chloride (1.13 mL, 1.15 g, 11.00 mmol, 1.1 eq.). After recrystallization from ethanol **1o** was obtained as red solid (1.01 g, 4.57 mmol, 46%).

^{1}H NMR (400 MHz, DMSO-d_6) δ [ppm]: 10.20 (s, 1H, *H*-6), 9.27 (s, 1H, *H*-5), 8.09 (dd, *J* = 8.5, 1.6 Hz, 1H, *H*-1), 7.58 (ddd, *J* = 8.6, 6.9, 1.6 Hz, 1H, *H*-3), 7.03 (dd, *J* = 8.6, 1.3 Hz, 1H, *H*-4), 6.86 (ddd, *J* = 8.5, 6.9, 1.6 Hz, 1H, *H*-2), 6.79 (dq, *J* = 15.3, 6.9 Hz, 1H, *H*-8), 6.05 (dq, *J* = 15.3, 1.7 Hz, 1H, *H*-7), 1.87 (dd, *J* = 6.9, 1.7 Hz, 3H, *H*-9).

^{13}C{^{1}H} NMR (101 MHz, DMSO-d_6) δ [ppm]: 164.5, 145.2, 140.5, 136.4, 131.7, 125.8, 122.9, 117.8, 114.7, 17.6.

HRMS (ESI-): *m/z* calculated for $C_{10}H_{10}N_3O_3^-$ [M-H]$^-$ 220.0728; found 220.0728.

m.p. (Toluene): 153.4–153.8 °C.

Ethyl 2-(2-(2-nitrophenyl)hydrazineyl)-2-oxoacetate (1p)

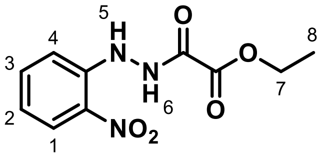

1p

$C_{10}H_{11}N_3O_5$

Mw = 253.21 g/mol

Synthesis of hydrazide **1p** was carried out according to **GPI** using (2-nitrophenyl)hydrazine hydrochloride (1.90 g, 10.00 mmol, 1.0 eq.) and ethyl 2-chloro-2-oxoacetate (1.23 mL, 1.50 g, 11.00 mmol, 1.1 eq.). After recrystallization from toluene **1p** was obtained as orange needles (0.69 g, 3.95 mmol, 27%).

^{1}H NMR (400 MHz, DMSO-d_6) δ [ppm]: 11.15 (s, 1H, *H*-6), 9.36 (s, 1H, *H*-5), 8.11 (dd, *J* = 8.5, 1.6 Hz, 1H, *H*-1), 7.60 (ddd, *J* = 8.6, 7.0, 1.6 Hz, 1H, *H*-3), 7.10 (d, *J* = 8.6 Hz, 1H, *H*-4), 6.90 (ddd, *J* = 8.5, 7.0, 1.2 Hz, 1H, *H*-2), 4.31 (q, *J* = 7.1 Hz, 2H, *H*-7), 1.31 (t, *J* = 7.1 Hz, 3H, *H*-8).

^{13}C{^{1}H} NMR (101 MHz, DMSO-d_6) δ [ppm]: 159.6, 156.8, 144.2, 136.4, 131.9, 125.8, 118.2, 114.8, 62.4, 13.8.

HRMS (ESI-): *m/z* calculated for $C_{10}H_{10}N_3O_5^-$ [M-H]$^-$ 252.0626; found 252.0628.

m.p. (Toluene): 144.6–145.0 °C.

1*H*-4-Methyl-*N*-(2-phenyl-benzo[*d*]imidazol-1-yl)benzamide (2a)

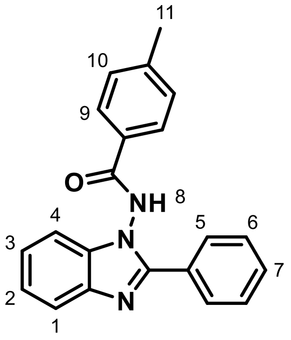

2a

$C_{21}H_{17}N_3O$

Mw = 327.39 g/mol

Synthesis of benzimidazole **2a** was carried out according to **GPIII** using **1a** (101.7 mg, 0.375 mmol, 1.0 eq.) and benzaldehyde (80 µL, 79.6 mg, 0.750 mmol, 2.0 eq.) as starting materials. Constant current electrolysis was performed at room temperature with a current density of 6.0 mA cm^{-2} until a charge of 5.0 *F* (181 C) was applied. After flash chromatography (Cy:EtOAc) **2a** was obtained as colorless solid (80.1 mg, 0.245 mmol, 65%).

^{1}H NMR (500 MHz, DMSO-*d*$_6$) δ [ppm]: 12.17 (s, 1H, *H*-8), 8.00 – 7.96 (m, 2H, *H*-5), 7.91 – 7.88 (m, 2H, *H*-9), 7.79 – 7.76 (m, 1H, *H*-1), 7.54 – 7.49 (m, 3H, *H*-6, *H*-7), 7.45 – 7.42 (m, 1H, *H*-4), 7.41 – 7.38 (m, 2H, *H*-10), 7.34 – 7.31 (m, 2H, *H*-2, *H*-3), 2.40 (s, 3H, *H*-11).
^{13}C{^{1}H} NMR (126 MHz, DMSO-*d*$_6$) δ [ppm]: 165.6, 151.9, 143.1, 140.4, 135.6, 130.2, 129.4, 128.9, 128.7, 128.4, 128.2, 127.7, 123.6, 122.9, 119.7, 109.8, 21.1.
HRMS (EI): *m/z* calculated for $C_{21}H_{17}N_3O^+$ [M]$^+$ 327.1366; found 327.1367.

1*H*-2-Methyl-*N*-(2-phenyl-benzo[*d*]imidazol-1-yl)benzamide (2b)

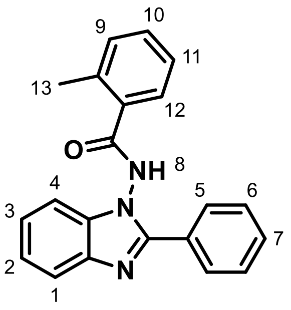

2b

$C_{21}H_{17}N_3O$

Mw = 327.39 g/mol

Synthesis of benzimidazole **2b** was carried out according to **GPIII** using **1b** (101.7 mg, 0.375 mmol, 1.0 eq.) and benzaldehyde (80 µL, 79.6 mg, 0.750 mmol, 2.0 eq.) as starting materials. Constant current electrolysis was performed at room temperature with a current density of 6.0 mA cm^{-2} until a charge of 5.0 *F* (181 C) was applied. After flash chromatography (Cy:EtOAc) **2b** was obtained as colorless solid (78.9 mg, 0.241 mmol, 64%).

^{1}H NMR (400 MHz, DMSO-*d*$_6$) δ [ppm]: 12.00 (s, 1H, *H*-8), 7.98 – 7.94 (m, 2H, *H*-5), 7.81 – 7.74 (m, 1H, *H*-1), 7.61 – 7.55 (m, 4H, *H*-6, *H*-7, *H*-12), 7.55 – 7.52 (m, 1H, *H*-9), 7.47 (td, *J* = 7.5, 1.5 Hz, 1H, *H*-10), 7.43 – 7.29 (m, 4H, *H*-2, *H*-3, *H*-4, *H*-11), 2.24 (s, 3H, *H*-13).
^{13}C{^{1}H} NMR (101 MHz, DMSO-*d*$_6$) δ [ppm]: 167.9, 152.2, 140.4, 136.2, 135.3, 133.0, 130.9, 130.8, 130.2, 128.8, 128.6, 128.4, 127.3, 125.9, 123.6, 122.9, 119.7, 109.6, 19.0.
HRMS (EI): *m/z* calculated for $C_{21}H_{17}N_3O^+$ [M]$^+$ 327.1366; found 327.1364.

1*H*-3-Methyl-*N*-(2-phenyl-benzo[*d*]imidazol-1-yl)benzamide (2c)

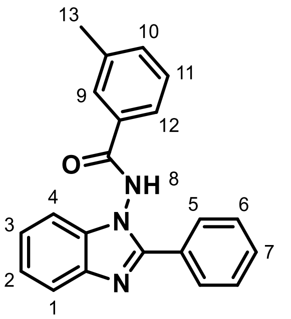

2c

$C_{21}H_{17}N_3O$

Mw = 327.39 g/mol

Synthesis of benzimidazole **2c** was carried out according to **GPIII** using **1c** (101.7 mg, 0.375 mmol, 1.0 eq.) and benzaldehyde (80 µL, 79.6 mg, 0.750 mmol, 2.0 eq.) as starting materials. Constant current electrolysis was performed at room temperature with a current density of 6.0 mA cm^{-2} until a charge of 5.0 *F* (181 C) was applied. After flash chromatography (Cy:EtOAc) **2c** was obtained as colorless solid (87.5 mg, 0.267 mmol, 71%).

^{1}H NMR (400 MHz, DMSO-*d*$_6$) δ [ppm]: 12.19 (s, 1H, *H*-8), 8.00 – 7.92 (m, 2H, *H*-5), 7.81 – 7.72 (m, 3H, *H*-1, *H*-9, *H*-12), 7.56 – 7.46 (m, 5H, *H*-6, *H*-7, *H*-10, *H*-11), 7.46 – 7.41 (m, 1H, *H*-4), 7.39 – 7.27 (m, 2H, *H*-2, *H*-3), 2.40 (s, 3H, *H*-13).
^{13}C{^{1}H} NMR (101 MHz, DMSO-*d*$_6$) δ [ppm]: 165.8, 151.9, 140.3, 138.4, 135.5, 133.4, 131.2, 130.2, 128.8, 128.8, 128.2, 128.2, 124.8, 123.6, 122.9, 119.7, 109.8, 20.9.
HRMS (ESI+): *m/z* calculated for $C_{21}H_{18}N_3O^+$ [M+H]$^+$ 328.1444; found 328.1443.

1*H*-*N*-(2-Phenyl-benzo[*d*]imidazol-1-yl)benzamide (2d)

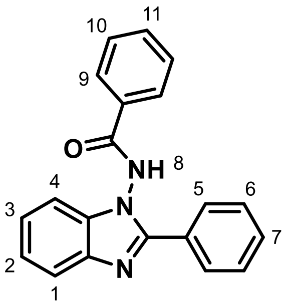

2d

$C_{20}H_{15}N_3O$

Mw = 313.36 g/mol

Synthesis of benzimidazole **2d** was carried out according to **GPIII** using **1d** (96.5 mg, 0.375 mmol, 1.0 eq.) and benzaldehyde (80 µL, 79.6 mg, 0.750 mmol, 2.0 eq.) as starting materials. Constant current electrolysis was performed at room temperature with a current density of 6.0 mA cm^{-2} until a charge of 5.0 *F* (181 C) was applied. After flash chromatography (Cy:EtOAc) **2d** was obtained as colorless solid (73.2 mg, 0.234 mmol, 62%).

^{1}H NMR (500 MHz, DMSO-*d*$_6$) *δ* [ppm]: 12.27 (s, 1H, *H*-8), 8.04 – 7.95 (m, 4H, *H*-5, *H*-9), 7.82 – 7.75 (m, 1H, *H*-1), 7.72 – 7.65 (m, 1H, *H*-11), 7.62 – 7.57 (m, 2H, *H*-10), 7.56 – 7.49 (m, 3H, *H*-6, *H*-7), 7.48 – 7.43 (m, 1H, *H*-4), 7.37 – 7.29 (m, 2H, *H*-2, *H*-3).

^{13}C{^{1}H} NMR (126 MHz, DMSO-*d*$_6$) *δ* [ppm]: 165.8, 151.9, 140.4, 135.5, 132.9, 131.2, 130.2, 129.0, 128.8, 128.8, 128.3, 127.7, 123.6, 123.0, 119.7, 109.8.

HRMS (EI): *m/z* calculated for $C_{20}H_{15}N_3O^+$ [M]$^+$ 313.1210; found 313.1212.

1*H*-4-Methoxy-*N*-(2-phenyl-benzo[*d*]imidazol-1-yl)benzamide (2e)

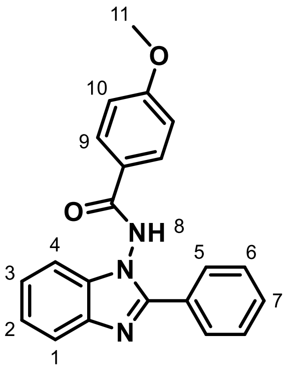

2e

$C_{21}H_{17}N_3O_2$

Mw = 343.39 g/mol

Synthesis of benzimidazole **2e** was carried out according to **GPIII** using **1e** (107.7 mg, 0.375 mmol, 1.0 eq.) and benzaldehyde (80 µL, 79.6 mg, 0.750 mmol, 2.0 eq.) as starting materials. Constant current electrolysis was performed at room temperature with a current density of 6.0 mA cm^{-2} until a charge of 5.0 *F* (181 C) was applied. After flash chromatography (Cy:EtOAc) **2e** was obtained as colorless solid (52.9 mg, 0.154 mmol, 41%).

^{1}H NMR (500 MHz, DMSO-*d*$_6$) *δ* [ppm]: 12.08 (s, 1H, *H*-8), 8.02 – 7.93 (m, 4H, *H*-5, *H*-9), 7.81 – 7.74 (m, 1H, *H*-1), 7.55 – 7.46 (m, 3H, *H*-6, *H*-7), 7.45 – 7.38 (m, 1H, *H*-4), 7.35 – 7.28 (m, 2H, *H*-2, *H*-3), 7.16 – 7.09 (m, 2H, *H*-10), 3.85 (s, 3H, *H*-11).

^{13}C{^{1}H} NMR (126 MHz, DMSO-*d*$_6$) *δ* [ppm]: 165.1, 162.8, 151.9, 140.3, 135.6, 130.2, 129.8, 128.9, 128.7, 128.2, 123.5, 123.3, 122.9, 119.7, 114.2, 109.8, 55.6.

HRMS (EI): *m/z* calculated for $C_{21}H_{17}N_3O_2^+$ [M]$^+$ 343.1315; found 343.1316.

1*H*-4-(*tert*-Butyl)-*N*-(2-phenyl-benzo[*d*]imidazol-1-yl)benzamide (2f)

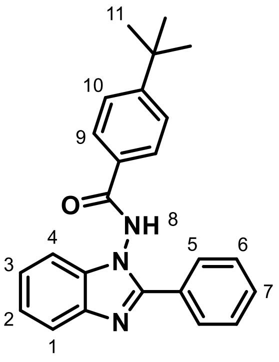

2f

$C_{24}H_{23}N_3O$

Mw = 369.47 g/mol

Synthesis of benzimidazole **2f** was carried out according to **GPIII** using **1f** (117.5 mg, 0.375 mmol, 1.0 eq.) and benzaldehyde (80 µL, 79.6 mg, 0.750 mmol, 2.0 eq.) as starting materials. Constant current electrolysis was performed at room temperature with a current density of 6.0 mA cm^{-2} until a charge of 5.0 *F* (181 C) was applied. After flash chromatography (Cy:EtOAc) **2f** was obtained as colorless solid (100.0 mg, 0.271 mmol, 72%).

^{1}H NMR (500 MHz, DMSO-*d*$_6$) *δ* [ppm]: 12.17 (s, 1H, *H*-8), 8.01 – 7.96 (m, 2H, *H*-5), 7.94 – 7.91 (m, 2H, *H*-9), 7.81 – 7.74 (m, 1H, *H*-1), 7.64 – 7.58 (m, 2H, *H*-10), 7.56 – 7.48 (m, 3H, *H*-6, *H*-7), 7.46 – 7.39 (m, 1H, *H*-4), 7.36 – 7.28 (m, 2H, *H*-2, *H*-3), 1.32 (s, 9H, *H*-11).
^{13}C{^{1}H} NMR (126 MHz, DMSO-*d*$_6$) *δ* [ppm]: 165.5, 155.9, 151.9, 140.3, 135.5, 130.2, 128.8, 128.7, 128.4, 128.2, 127.6, 125.8, 123.6, 122.9, 119.7, 109.8, 34.9, 30.9.
HRMS (EI): *m/z* calculated for $C_{24}H_{23}N_3O^+$ $[M]^+$ 369.1836; found 369.1833.

1*H*-4-Fluoro-*N*-(2-phenyl-benzo[*d*]imidazol-1-yl)benzamide (2g)

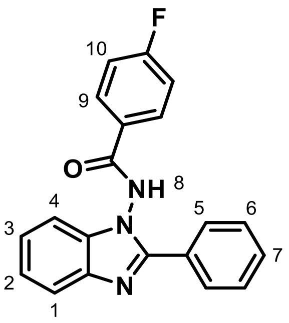

2g

$C_{20}H_{14}FN_3O$

Mw = 331.35 g/mol

Synthesis of benzimidazole **2g** was carried out according to GPIII using **1g** (103.2 mg, 0.375 mmol, 1.0 eq.) and benzaldehyde (80 µL, 79.6 mg, 0.750 mmol, 2.0 eq.) as starting materials. Constant current electrolysis was performed at room temperature with a current density of 6.0 mA cm^{-2} until a charge of 5.0 *F* (181 C) was applied. After flash chromatography (Cy:EtOAc) **2g** was obtained as colorless solid (81.4 mg, 0.246 mmol, 66%).

^{1}H NMR (400 MHz, DMSO-*d*$_6$) *δ* [ppm]: 12.24 (s, 1H, *H*-8), 8.08 – 8.02 (m, 2H, *H*-9), 7.99 – 7.94 (m, 2H, *H*-5), 7.81 – 7.72 (m, 1H, *H*-1), 7.60 – 7.49 (m, 3H, *H*-6, *H*-7), 7.48 – 7.40 (m, 3H, *H*-4, *H*-10), 7.38 – 7.28 (m, 2H, *H*-2, *H*-3).
^{13}C{^{1}H} NMR (101 MHz, DMSO-*d*$_6$) *δ* [ppm]: 164.7 (d, *J* = 250.8 Hz), 164.6, 151.8, 140.3, 135.4, 130.5 (d, *J* = 9.4 Hz), 130.1, 128.7, 128.7, 128.1, 127.7, 123.5, 122.9, 119.6, 115.9 (d, *J* = 22.1 Hz), 109.7.
^{19}F NMR (376 MHz, DMSO-*d*$_6$) *δ* [ppm]: -106.7 (tt, *J* = 9.5, 5.4 Hz).
HRMS (EI): *m/z* calculated for $C_{20}H_{14}FN_3O^+$ $[M]^+$ 331.1115; found 331.1114.

1*H*-3-Chloro-*N*-(2-phenyl-benzo[*d*]imidazol-1-yl)benzamide (2h)

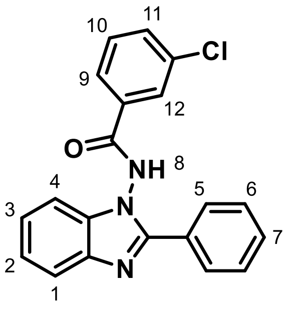

2h

$C_{20}H_{14}ClN_3O$

Mw = 347.80 g/mol

Synthesis of benzimidazole **2h** was carried out according to **GPIII** using **1h** (109.4 mg, 0.375 mmol, 1.0 eq.) and benzaldehyde (80 µL, 79.6 mg, 0.750 mmol, 2.0 eq.) as starting materials. Constant current electrolysis was performed at room temperature with a current density of 6.0 mA cm^{-2} until a charge of 5.0 *F* (181 C) was applied. After flash chromatography (Cy:EtOAc) **2h** was obtained as colorless solid (68.3 mg, 0.196 mmol, 52%).

^{1}H NMR (500 MHz, DMSO-*d*$_6$) *δ* [ppm]: 12.39 (s, 1H, *H*-8), 8.02 (t, *J* = 1.9 Hz, 1H, *H*-12), 7.98 – 7.94 (m, 2H, *H*-5), 7.92 (dt, *J* = 7.9, 1.9 Hz, 1H, *H*-9), 7.81 – 7.74 (m, 2H, *H*-1, *H*-11), 7.63 (t, *J* = 7.9 Hz, 1H, *H*-10), 7.57 – 7.47 (m, 4H, *H*-4, *H*-6, *H*-7), 7.37 – 7.28 (m, 2H, *H*-2, *H*-3).

^{13}C{^{1}H} NMR (126 MHz, DMSO-*d*$_6$) *δ* [ppm]: 164.5, 151.8, 140.3, 135.4, 133.7, 133.2, 132.7, 131.0, 130.3, 128.8, 128.7, 128.2, 127.5, 126.5, 123.6, 123.0, 119.7, 109.9.

HRMS (EI): *m/z* calculated for $C_{20}H_{14}ClN_3O^+$ $[M]^+$ 347.0820 (^{35}Cl); found 347.0818.

1*H*-*N*-(2-Phenyl-benzo[*d*]imidazol-1-yl)-2-(trifluoromethyl)benzamide (2i)

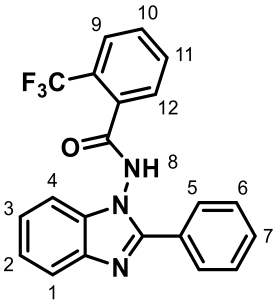

2i

$C_{21}H_{14}F_3N_3O$

Mw = 381.36 g/mol

Synthesis of benzimidazole **2i** was carried out according to **GPIII** using **1i** (122.0 mg, 0.375 mmol, 1.0 eq.) and benzaldehyde (80 µL, 79.6 mg, 0.750 mmol, 2.0 eq.) as starting materials. Constant current electrolysis was performed at room temperature with a current density of 6.0 mA cm^{-2} until a charge of 5.0 *F* (181 C) was applied. After flash chromatography (Cy:EtOAc) **2i** was obtained as colorless solid (90.1 mg, 0.236 mmol, 63%).

^{1}H NMR (400 MHz, DMSO-*d*$_6$) *δ* [ppm]: 12.36 (s, 1H, *H*-8), 8.02 – 7.73 (m, 6H, *H*-1, *H*-5, *H*-9, *H*-10, *H*-11), 7.70 – 7.66 (m, 1H, *H*-12), 7.65 – 7.58 (m, 3H, *H*-6, *H*-7), 7.50 – 7.46 (m, 1H, *H*-4), 7.40 (td, *J* = 7.6, 1.3 Hz, 1H, *H*-3), 7.35 (td, *J* = 7.6, 1.3 Hz, 1H, *H*-2).

^{13}C{^{1}H} NMR (101 MHz, DMSO-*d*$_6$) *δ* [ppm]: 165.8, 152.1, 140.4, 135.1, 132.9, 131.4, 130.3, 128.8, 128.7, 128.5, 128.4, 126.9 (q, *J* = 4.7 Hz), 126.6 (d, *J* = 31.8 Hz), 124.8, 123.7, 123.0, 122.0, 119.8, 109.3.

^{19}F NMR (376 MHz, DMSO-*d*$_6$) *δ* [ppm]: -57.6.

HRMS (EI): *m/z* calculated for $C_{21}H_{14}F_3N_3O^+$ $[M]^+$ 381.1083; found 381.1081.

1*H*-2,4,6-Trichloro-*N*-(2-phenyl-benzo[*d*]imidazol-1-yl)benzamide (2j)

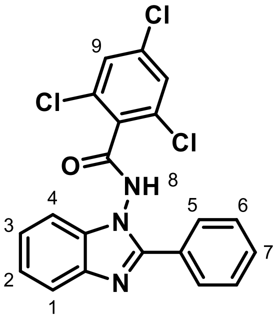

2j
$C_{20}H_{12}Cl_3N_3O$
Mw = 416.69 g/mol

Synthesis of benzimidazole **2j** was carried out according to **GPIII** using **1j** (135.2 mg, 0.375 mmol, 1.0 eq.) and benzaldehyde (80 µL, 79.6 mg, 0.750 mmol, 2.0 eq.) as starting materials. Constant current electrolysis was performed at room temperature with a current density of 6.0 mA cm^{-2} until a charge of 5.0 *F* (181 C) was applied. After flash chromatography (Cy:EtOAc) **2j** was obtained as colorless solid (104.7 mg, 0.251 mmol, 67%).

^{1}H NMR (400 MHz, DMSO-*d*$_6$) *δ* [ppm]: 12.57 (s, 1H, *H*-8), 7.94 – 7.85 (m, 4H, *H*-5, *H*-9), 7.77 (dt, *J* = 7.7, 0.9 Hz, 1H, *H*-1), 7.65 – 7.61 (m, 1H, *H*-4), 7.55 (dd, *J* = 5.2, 1.9 Hz, 3H, *H*-6, *H*-7), 7.44 – 7.30 (m, 2H, *H*-2, *H*-3).
^{13}C{^{1}H} NMR (101 MHz, DMSO-*d*$_6$) *δ* [ppm]: 161.9, 152.4, 140.4, 136.0, 135.1, 132.5, 130.1, 129.2, 128.9, 128.4, 128.4, 126.4, 123.8, 123.0, 119.8, 110.0.
HRMS (ESI-): *m/z* calculated for $C_{20}H_{12}Cl_3N_3O^+$ [M]$^+$ 416.0119 (^{35}Cl); found 416.0120.

1*H*-*N*-(2-Phenyl-benzo[*d*]imidazol-1-yl)-1-naphthamide (2k)

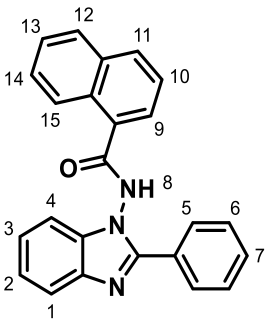

2k
$C_{24}H_{17}N_3O$
Mw = 363.42 g/mol

Synthesis of benzimidazole **2k** was carried out according to **GPIII** using **1k** (115.2 mg, 0.375 mmol, 1.0 eq.) and benzaldehyde (80 µL, 79.6 mg, 0.750 mmol, 2.0 eq.) as starting materials. Constant current electrolysis was performed at room temperature with a current density of 6.0 mA cm^{-2} until a charge of 5.0 *F* (181 C) was applied. After flash chromatography (Cy:EtOAc) **2k** was obtained as colorless solid (79.0 mg, 0.217 mmol, 58%).

^{1}H NMR (500 MHz, DMSO-*d*$_6$) *δ* [ppm]: 12.31 (s, 1H, *H*-8), 8.17 (d, *J* = 8.3 Hz, 1H, *H*-11), 8.07 – 7.98 (m, 3H, *H*-5, *H*-12), 7.94 (dd, *J* = 7.0, 1.1 Hz, 1H, *H*-9), 7.84 – 7.78 (m, 2H, *H*-1, *H*-15), 7.68 (dd, *J* = 8.3, 7.0 Hz, 1H, *H*-10), 7.65 – 7.63 (m, 1H, *H*-14), 7.62 – 7.57 (m, 4H, *H*-6, *H*-7, *H*-13), 7.53 (ddd, *J* = 8.3, 6.8, 1.4 Hz, 1H, *H*-4), 7.44 – 7.34 (m, 2H, *H*-2, *H*-3).
^{13}C{^{1}H} NMR (126 MHz, DMSO-*d*$_6$) *δ* [ppm]: 167.6, 152.3, 140.5, 135.4, 133.2, 131.5, 130.6, 130.3, 129.6, 128.8, 128.8, 128.5, 128.5, 127.4, 126.7, 126.2, 125.0, 124.5, 123.8, 123.0, 119.8, 109.7.
HRMS (ESI+): *m/z* calculated for $C_{24}H_{18}N_3O^+$ [M+H]$^+$ 364.1444; found 364.1445.

1*H*-*N*-(2-Phenyl-benzo[*d*]imidazol-1-yl)isobutyramide (2l)

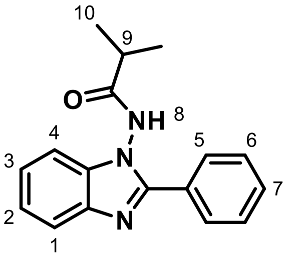

2l
$C_{17}H_{17}N_3O$
Mw = 279.34 g/mol

Synthesis of benzimidazole **2l** was carried out according to **GPIII** using **1l** (83.7 mg, 0.375 mmol, 1.0 eq.) and benzaldehyde (80 µL, 79.6 mg, 0.750 mmol, 2.0 eq.) as starting materials. Constant current electrolysis was performed at room temperature with a current density of 6.0 mA cm^{-2} until a charge of 5.0 *F* (181 C) was applied. After flash chromatography (Cy:EtOAc) **2l** was obtained as colorless solid (54.0 mg, 0.193 mmol, 52%).

^{1}H NMR (500 MHz, DMSO-*d*$_6$) *δ* [ppm]: 11.56 (s, 1H, *H*-8), 7.94 – 7.86 (m, 2H, *H*-5), 7.76 – 7.70 (m, 1H, *H*-1), 7.60 – 7.50 (m, 3H, *H*-6, *H*-7), 7.37 – 7.27 (m, 3H, *H*-2, *H*-3, *H*-4), 2.67 (h, *J* = 6.9 Hz, 1H, *H*-9), 1.18 (d, *J* = 6.9 Hz, 3H, *H*-10), 1.09 – 1.03 (d, *J* = 6.9 Hz, 3H, *H*-10).
^{13}C{^{1}H} NMR (126 MHz, DMSO-*d*$_6$) *δ* [ppm]: 175.5, 151.8, 140.3, 135.3, 130.2, 128.7, 128.6, 128.4, 123.5, 122.8, 119.6, 109.5, 32.5, 19.1, 19.0.
HRMS (EI): *m/z* calculated for $C_{17}H_{17}N_3O^+$ [M]$^+$ 279.1366; found 279.1365.

1*H*-*N*-(2-Phenyl-benzo[*d*]imidazol-1-yl)pivalamide (2m)

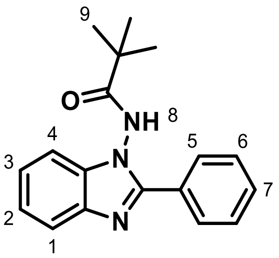

2m
$C_{18}H_{19}N_3O$
Mw = 293.37 g/mol

Synthesis of benzimidazole **2m** was carried out according to **GPIII** using **1m** (115.2 mg, 0.375 mmol, 1.0 eq.) and benzaldehyde (80 µL, 79.6 mg, 0.750 mmol, 2.0 eq.) as starting materials. Constant current electrolysis was performed at room temperature with a current density of 6.0 mA cm^{-2} until a charge of 5.0 *F* (181 C) was applied. After flash chromatography (Cy:EtOAc) **2m** was obtained as colorless solid (79.0 mg, 0.217 mmol, 58%).

^{1}H NMR (500 MHz, DMSO-*d*$_6$) *δ* [ppm]: 11.34 (s, 1H, *H*-8), 7.96 – 7.88 (m, 2H, *H*-5), 7.77 – 7.71 (m, 1H, *H*-1), 7.59 – 7.50 (m, 3H, *H*-6, *H*-7), 7.37 – 7.25 (m, 3H, *H*-2, *H*-3, *H*-4), 1.25 (s, 9H, *H*-9).

^{13}C{^{1}H} NMR (126 MHz, DMSO-*d*$_6$) *δ* [ppm]: 176.6, 152.0, 140.3, 135.4, 130.2, 128.7, 128.6, 128.4, 123.4, 122.8, 119.6, 109.4, 38.0, 26.9.

HRMS (EI): *m/z* calculated for $C_{18}H_{19}N_3O^+$ [M]$^+$ 293.1523; found 293.1524.

1*H*-2-Phenyl-*N*-(2-phenyl-benzo[*d*]imidazol-1-yl)acetamide (2n)

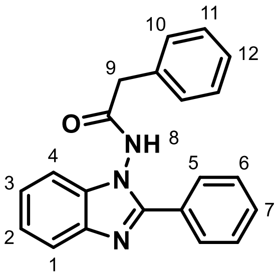

2n
$C_{21}H_{17}N_3O$
Mw = 327.39 g/mol

Synthesis of benzimidazole **2n** was carried out according to **GPIII** using **1n** (101.7 mg, 0.375 mmol, 1.0 eq.) and benzaldehyde (80 µL, 79.6 mg, 0.750 mmol, 2.0 eq.) as starting materials. Constant current electrolysis was performed at room temperature with a current density of 6.0 mA cm^{-2} until a charge of 5.0 *F* (181 C) was applied. After flash chromatography (Cy:EtOAc) **2n** was obtained as colorless solid (51.7 mg, 0.158 mmol, 42%).

^{1}H NMR (400 MHz, DMSO-*d*$_6$) *δ* [ppm]: 11.82 (s, 1H, *H*-8), 7.89 – 7.82 (m, 2H, *H*-5), 7.75 – 7.70 (m, 1H, *H*-1), 7.53 – 7.48 (m, 1H, *H*-4), 7.47 – 7.41 (m, 2H, *H*-2, *H*-3), 7.38 – 7.26 (m, 8H, *H*-6, *H*-7, *H*-10, *H*-11, *H*-12), 3.74 (s, 2H, *H*-9).

^{13}C{^{1}H} NMR (101 MHz, DMSO-*d*$_6$) *δ* [ppm]: 169.7, 151.3, 140.1, 135.2, 134.5, 130.0, 129.1, 128.5, 128.5, 128.5, 128.2, 126.9, 123.4, 122.8, 119.6, 109.5, 40.3.

HRMS (ESI+): *m/z* calculated for $C_{21}H_{18}N_3O^+$ [M+H]$^+$ 328.1444; found 328.1444.

1*H*-(*E*)-*N*-(2-Phenyl-benzo[*d*]imidazol-1-yl)but-2-enamide (2o)

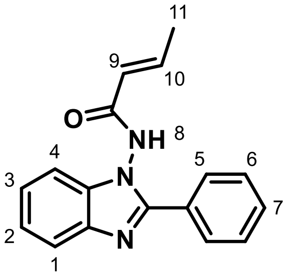

2o
$C_{17}H_{15}N_3O$
Mw = 277.33 g/mol

Synthesis of benzimidazole **2o** was carried out according to **GPIII** using **1o** (83.0 mg, 0.375 mmol, 1.0 eq.) and benzaldehyde (80 µL, 79.6 mg, 0.750 mmol, 2.0 eq.) as starting materials. Constant current electrolysis was performed at room temperature with a current density of 6.0 mA cm^{-2} until a charge of 5.0 *F* (181 C) was applied. After flash chromatography (Cy:EtOAc) **2o** was obtained as colorless solid (75.6 mg, 0.273 mmol, 73%).

^{1}H NMR (400 MHz, DMSO-*d*$_6$) *δ* [ppm]: 11.65 (s, 1H, *H*-8), 7.97 – 7.88 (m, 2H, *H*-5), 7.79 – 7.70 (m, 1H, *H*-1), 7.60 – 7.48 (m, 3H, *H*-6, *H*-7), 7.40 – 7.25 (m, 3H, *H*-2, *H*-3, *H*-4), 6.88 (dq, *J* = 15.5, 6.9 Hz, 1H, *H*-10), 6.16 (dd, *J* = 15.5, 1.7 Hz, 1H, *H*-9), 1.90 (dd, *J* = 6.9, 1.7 Hz, 3H, *H*-11).

^{13}C{^{1}H} NMR (101 MHz, DMSO-*d*$_6$) *δ* [ppm]: 164.2, 151.6, 142.9, 140.2, 135.4, 130.1, 128.8, 128.6, 128.2, 123.4, 122.8, 121.9, 119.6, 109.5, 17.7.

HRMS (ESI+): *m/z* calculated for $C_{17}H_{16}N_3O^+$ [M+H]$^+$ 278.1288; found 278.1287.

Ethyl 1*H*-2-oxo-2-((2-phenyl-benzo[*d*]imidazol-1-yl)amino)acetate (2p) & 1*H*-2,3,4,5,6-pentafluoro-*N*-(2-phenyl-benzo[*d*]imidazol-1-yl)benzamide (2q)

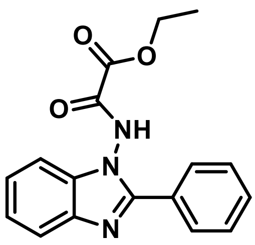

2p
$C_{17}H_{15}N_3O_3$
Mw = 309.33 g/mol

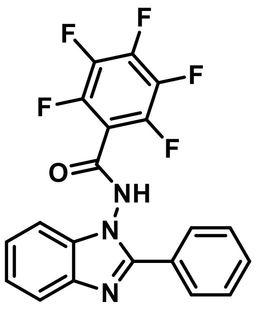

2q
$C_{20}H_{10}F_5N_3O$
Mw = 403.31 g/mol

The ester-substituted derivative **1p** did not yield benzimidazole **2p** and ^{1}H NMR did not show the presence of the ethyl group in the isolated product but rather the carbonyl aldehyde. Molecule **2q** could also not be obtained due to laborious separation from side products while forming **1q**.

1*H*-4-Methyl-*N*-(2-(*p*-tolyl)-benzo[*d*]imidazol-1-yl)benzamide (3a)

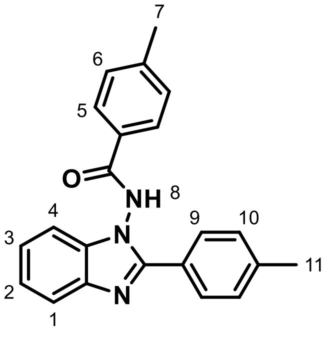

3a
$C_{22}H_{19}N_3O$
Mw = 341.41 g/mol

Synthesis of benzimidazole **3a** was carried out according to **GPIII** using **1a** (101.9 mg, 0.375 mmol, 1.0 eq.) and 4-methylbenzaldehyde (88 µL, 90.1 mg, 0.750 mmol, 2.0 eq.) as starting materials. Constant current electrolysis was performed at room temperature with a current density of 6.0 mA cm^{-2} until a charge of 5.0 *F* (181 C) was applied. After flash chromatography (Cy:EtOAc) **3a** was obtained as colorless solid (96.1 mg, 0.281 mmol, 75%).

^{1}H NMR (400 MHz, DMSO-*d*$_6$) *δ* [ppm]: 12.12 (s, 1H, *H*-8), 7.91 – 7.83 (m, 4H, *H*-5, *H*-9), 7.78 – 7.69 (m, 1H, *H*-1), 7.44 – 7.35 (m, 3H, *H*-4, *H*-10), 7.35 – 7.25 (m, 4H, *H*-2, *H*-3, *H*-6), 2.41 (s, 3H, *H*-11), 2.34 (s, 3H, *H*-7).
^{13}C{^{1}H} NMR (101 MHz, DMSO-*d*$_6$) *δ* [ppm]: 165.5, 152.0, 143.1, 140.4, 140.0, 135.5, 129.4, 129.3, 128.5, 128.1, 127.7, 126.0, 123.3, 122.8, 119.5, 109.7, 21.1, 21.0.
HRMS (EI): *m/z* calculated for $C_{22}H_{19}N_3O^+$ [M]$^+$ 341.1523; found 341.1521.

1*H*-*N*-(2-(4-Chlorophenyl)-benzo[*d*]imidazol-1-yl)-4-methylbenzamide (3b)

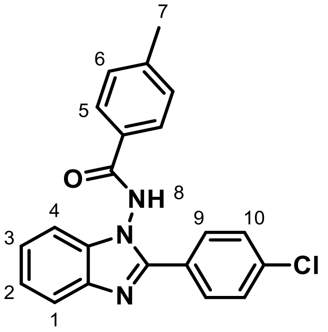

3b
$C_{21}H_{16}ClN_3O$
Mw = 361.83 g/mol

Synthesis of benzimidazole **3b** was carried out according to **GPIII** using **1a** (101.5 mg, 0.375 mmol, 1.0 eq.) and 4-chlorobenzaldehyde (105.0 mg, 0.750 mmol, 2.0 eq.) as starting materials. Constant current electrolysis was performed at room temperature with a current density of 6.0 mA cm^{-2} until a charge of 5.0 *F* (181 C) was applied. After flash chromatography (Cy:EtOAc) **3b** was obtained as colorless solid (105.9 mg, 0.293 mmol, 78%).

^{1}H NMR (400 MHz, DMSO-*d*$_6$) *δ* [ppm]: 12.17 (s, 1H, *H*-8), 8.02 – 7.92 (m, 2H, *H*-9), 7.91 – 7.84 (m, 2H, *H*-5), 7.82 – 7.73 (m, 1H, *H*-1), 7.65 – 7.57 (m, 2H, *H*-10), 7.46 – 7.37 (m, 3H, *H*-4, *H*-6), 7.36 – 7.29 (m, 2H, *H*-2, *H*-3), 2.41 (s, 3H, *H*-7).
^{13}C{^{1}H} NMR (101 MHz, DMSO-*d*$_6$) *δ* [ppm]: 165.6, 150.8, 143.2, 140.3, 135.5, 135.1, 129.9, 129.5, 129.0, 128.3, 127.8, 127.7, 123.8, 123.1, 119.8, 109.9, 21.1.
HRMS (ESI+): *m/z* calculated for $C_{21}H_{17}ClN_3O^+$ [M+H]$^+$ 362.1055 (^{35}Cl); found 362.1053.

1*H*-*N*-(2-(4-Bromophenyl)-benzo[*d*]imidazol-1-yl)-4-methylbenzamide (3c)

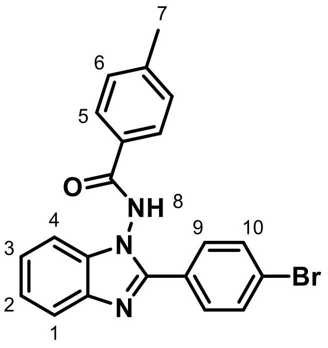

3c
$C_{21}H_{16}BrN_3O$
Mw = 406.28 g/mol

Synthesis of benzimidazole **3c** was carried out according to **GPIII** using **1a** (101.9 mg, 0.375 mmol, 1.0 eq.) and 4-bromobenzaldehyde (138.8 mg, 0.750 mmol, 2.0 eq.) as starting materials. Constant current electrolysis was performed at room temperature with a current density of 6.0 mA cm^{-2} until a charge of 5.0 *F* (181 C) was applied. After flash chromatography (Cy:EtOAc) **3c** was obtained as colorless solid (116.2 mg, 0.286 mmol, 76%).

^{1}H NMR (400 MHz, DMSO-*d*$_6$) δ [ppm]: 12.17 (s, 1H, *H*-8), 7.94 – 7.85 (m, 4H, *H*-5, *H*-9), 7.79 – 7.72 (m, 3H, *H*-1, *H*-10), 7.47 – 7.38 (m, 3H, *H*-4, *H*-6), 7.37 – 7.30 (m, 2H, *H*-2, *H*-3), 2.41 (s, 3H, *H*-7).
^{13}C{^{1}H} NMR (101 MHz, DMSO-*d*$_6$) δ [ppm]: 165.6, 150.9, 143.2, 140.3, 135.5, 131.9, 130.1, 129.5, 128.3, 128.0, 127.8, 123.9, 123.8, 123.1, 119.8, 109.9, 21.1.
HRMS (ESI+): *m/z* calculated for $C_{21}H_{17}N_3OBr^+$ [M+H]$^+$ 406.0550 (^{79}Br); found 406.0550.

1*H*-*N*-(2-(4-Iodophenyl)-benzo[*d*]imidazol-1-yl)-4-methylbenzamide (3d)

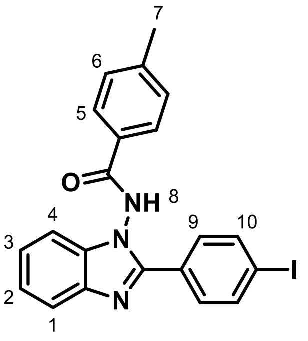

3d
$C_{21}H_{16}IN_3O$
Mw = 453.28 g/mol

Synthesis of benzimidazole **3d** was carried out according to **GPIII** using **1a** (101.8 mg, 0.375 mmol, 1.0 eq.) and 4-iodobenzaldehyde (173.3 mg, 0.750 mmol, 2.0 eq.) as starting materials. Constant current electrolysis was performed at room temperature with a current density of 6.0 mA cm^{-2} until a charge of 5.0 *F* (181 C) was applied. After flash chromatography (Cy:EtOAc) **3d** was obtained as colorless solid (127.2 mg, 0.280 mmol, 75%).

^{1}H NMR (400 MHz, DMSO-*d*$_6$) δ [ppm]: 12.18 (s, 1H, *H*-8), 7.92 – 7.88 (m, 2H, *H*-9), 7.87 (d, 2H, *J* = 7.9 Hz, *H*-5), 7.78 – 7.72 (m, 3H, *H*-1, *H*-10), 7.43 – 7.37 (m, 3H, *H*-4, *H*-6), 7.35 – 7.30 (m, 2H, *H*-2, *H*-3), 2.41 (s, 3H, *H*-7).
^{13}C{^{1}H} NMR (101 MHz, DMSO-*d*$_6$) δ [ppm]: 165.7, 151.2, 143.3, 140.3, 137.7, 135.6, 130.0, 129.5, 128.3, 128.3, 127.8, 123.9, 123.2, 119.8, 109.9, 97.6, 21.2.
HRMS (ESI+): *m/z* calculated for $C_{21}H_{17}N_3OI^+$ [M+H]$^+$ 454.0411; found 454.0414.

Methyl 1*H*-4-(1-(4-methylbenzamido)-benzo[*d*]imidazol-2-yl)benzoate (3e)

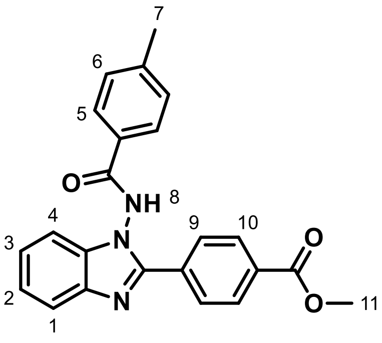

3e
$C_{23}H_{19}N_3O_3$
Mw = 385.42 g/mol

Synthesis of benzimidazole **3e** was carried out according to **GPIII** using **1a** (101.9 mg, 0.375 mmol, 1.0 eq.) and methyl 4-formylbenzoate (123.2 mg, 0.750 mmol, 2.0 eq.) as starting materials. Constant current electrolysis was performed at room temperature with a current density of 6.0 mA cm^{-2} until a charge of 5.0 *F* (181 C) was applied. After flash chromatography (Cy:EtOAc) **3e** was obtained as colorless solid (105.9 mg, 0.275 mmol, 73%).

^{1}H NMR (400 MHz, DMSO-*d*$_6$) δ [ppm]: 12.23 (s, 1H, *H*-8), 8.15 – 8.12 (m, 2H, *H*-9), 8.10 – 8.06 (m, 2H, *H*-10), 7.88 (d, *J* = 8.2 Hz, 2H, *H*-5), 7.82 – 7.78 (m, 1H, *H*-1), 7.49 – 7.44 (m, 1H, *H*-4), 7.40 (d, *J* = 8.2 Hz, 2H, *H*-6), 7.39 – 7.31 (m, 2H, *H*-2, *H*-3), 3.86 (s, 3H, *H*-11), 2.40 (s, 3H, *H*-7).
^{13}C{^{1}H} NMR (101 MHz, DMSO-*d*$_6$) δ [ppm]: 165.7, 165.7, 150.7, 143.2, 140.4, 135.6, 133.1, 130.7, 129.5, 129.4, 128.5, 128.3, 127.8, 124.1, 123.2, 119.9, 110.0, 52.4, 21.1.
HRMS (ESI+): *m/z* calculated for $C_{23}H_{20}N_3O_3^+$ $[M+H]^+$ 386.1499; found 386.1501.

1*H*-*N*-(2-(2-Fluorophenyl)-benzo[*d*]imidazol-1-yl)-4-methylbenzamide (3f)

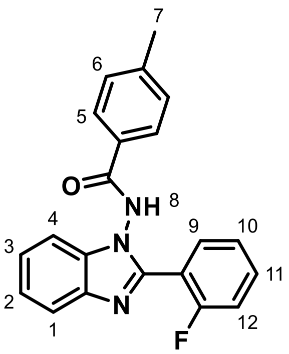

3f
$C_{21}H_{16}FN_3O$
Mw = 345.38 g/mol

Synthesis of benzimidazole **3f** was carried out according to **GPIII** using **1a** (101.5 mg, 0.375 mmol, 1.0 eq.) and 2-fluorobenzaldehyde (80 µL, 93.1 mg, 0.750 mmol, 2.0 eq.) as starting materials. Constant current electrolysis was performed at room temperature with a current density of 6.0 mA cm^{-2} until a charge of 5.0 *F* (181 C) was applied. After flash chromatography (Cy:EtOAc) **3f** was obtained as colorless solid (85.5 mg, 0.248 mmol, 66%).

^{1}H NMR (400 MHz, DMSO-*d*$_6$) δ [ppm]: 12.03 (s, 1H, *H*-8), 7.84 – 7.76 (m, 3H, *H*-1, *H*-5), 7.71 (td, *J* = 7.5, 1.8 Hz, 1H, *H*-10), 7.62 – 7.52 (m, 1H, *H*-9), 7.48 – 7.30 (m, 7H, *H*-2, *H*-3, *H*-4, *H*-6, *H*-11, *H*-12), 2.37 (s, 3H, *H*-7).
^{13}C{^{1}H} NMR (101 MHz, DMSO-*d*$_6$) δ [ppm]: 165.6, 159.7 (d, *J* = 250.7 Hz), 148.4 (d, *J* = 2.1 Hz), 143.0, 140.6, 132.6 (d, *J* = 8.3 Hz), 131.7 (d, *J* = 2.2 Hz), 129.3, 128.4, 127.7, 124.6 (d, *J* = 3.5 Hz), 123.8, 122.9, 119.9, 117.0 (d, *J* = 13.8 Hz), 116.3 (d, *J* = 21.3 Hz), 109.9, 21.1.
^{19}F NMR (376 MHz, DMSO-*d*$_6$) δ [ppm]: -110.26 – -115.51 (m).
HRMS (ESI+): *m/z* calculated for $C_{21}H_{17}N_3OF^+$ $[M+H]^+$ 346.1350; found 346.1350.

1*H*-*N*-(2-(3-Fluorophenyl)-benzo[*d*]imidazol-1-yl)-4-methylbenzamide (3g)

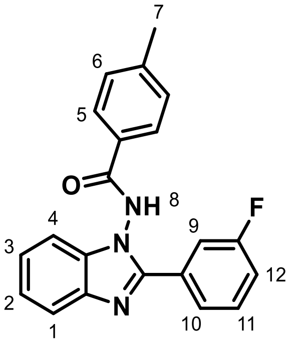

3g
$C_{21}H_{16}FN_3O$
Mw = 345.38 g/mol

Synthesis of benzimidazole **3g** was carried out according to **GPIII** using **1a** (101.9 mg, 0.375 mmol, 1.0 eq.) and 3-fluorobenzaldehyde (80 µL, 93.1 mg, 0.750 mmol, 2.0 eq.) as starting materials. Constant current electrolysis was performed at room temperature with a current density of 6.0 mA cm^{-2} until a charge of 5.0 *F* (181 C) was applied. After flash chromatography (Cy:EtOAc) **3g** was obtained as colorless solid (92.4 mg, 0.268 mmol, 71%).

^{1}H NMR (400 MHz, DMSO-*d*$_6$) *δ* [ppm]: 12.17 (s, 1H, *H*-8), 7.92 – 7.86 (m, 2H, *H*-5), 7.83 (dq, *J* = 8.0, 1.3 Hz, 1H, *H*-10), 7.81 – 7.74 (m, 2H, *H*-1, *H*-9), 7.58 (dd, *J* = 8.0, 6.0 Hz, 1H, *H*-11), 7.49 – 7.43 (m, 1H, *H*-4), 7.41 (d, *J* = 7.8 Hz, 2H, *H*-6), 7.37 – 7.32 (m, 3H, *H*-2, *H*-3, *H*-12), 2.41 (s, 3H, *H*-7).
^{13}C{^{1}H} NMR (101 MHz, DMSO-*d*$_6$) *δ* [ppm]: 165.6, 161.9 (d, *J* = 243.8 Hz), 150.4, 150.4, 143.2, 140.2, 135.5, 131.0 (d, *J* = 8.4 Hz), 130.9 (d, *J* = 8.7 Hz), 129.4, 128.3, 127.6, 124.3 (d, *J* = 2.8 Hz), 123.9, 123.1, 119.8, 117.0 (d, *J* = 20.8 Hz), 114.7 (d, *J* = 23.7 Hz), 109.8, 21.1.
^{19}F NMR (376 MHz, DMSO-*d*$_6$) *δ* [ppm]: -112.3 (td, *J* = 9.5, 6.1 Hz).
HRMS (ESI+): *m/z* calculated for $C_{21}H_{17}N_3OF^+$ $[M+H]^+$ 346.1350; found 346.1350.

***N*-(2-(4-Fluorophenyl)-1*H*-benzo[*d*]imidazol-1-yl)-4-methylbenzamide (3h)**

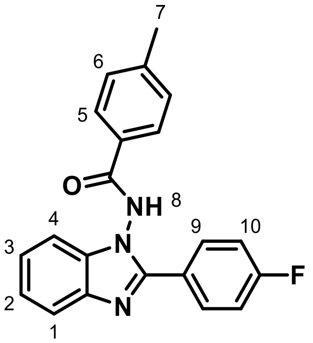

3h
$C_{21}H_{16}FN_3O$
Mw = 345.38 g/mol

Synthesis of benzimidazole **3h** was carried out according to **GPIII** using **1a** (101.9 mg, 0.375 mmol, 1.0 eq.) and 4-fluorobenzaldehyde (80 µL, 93.1 mg, 0.750 mmol, 2.0 eq.) as starting materials. Constant current electrolysis was performed at room temperature with a current density of 6.0 mA cm^{-2} until a charge of 5.0 *F* (181 C) was applied. After flash chromatography (Cy:EtOAc) **3h** was obtained as colorless solid (82.2 mg, 0.238 mmol, 63%).

^{1}H NMR (400 MHz, DMSO-*d*$_6$) *δ* [ppm]: 12.12 (s, 1H, *H*-8), 8.06 – 7.97 (m, 2H, *H*-9), 7.94 – 7.84 (m, 2H, *H*-5), 7.83 – 7.71 (m, 1H, *H*-1), 7.46 – 7.35 (m, 5H, *H*-4, *H*-6, *H*-10), 7.34 – 7.27 (m, 2H, *H*-2, *H*-3), 2.41 (s, 3H, *H*-7).
^{13}C{^{1}H} NMR (101 MHz, DMSO-*d*$_6$) *δ* [ppm]: 165.6, 163.1 (d, *J* = 248.4 Hz), 151.0, 143.1, 140.2, 135.4, 130.5 (d, *J* = 8.9 Hz), 129.4, 128.3, 127.7, 125.4, 125.3 (d, *J* = 3.2 Hz), 122.9, 119.6, 115.8 (d, *J* = 21.9 Hz), 109.7, 21.1.
^{19}F NMR (376 MHz, DMSO-*d*$_6$) *δ* [ppm]: -110.3 (tt, *J* = 8.8, 5.5 Hz).
HRMS (ESI+): *m/z* calculated for $C_{21}H_{17}N_3OF^+$ $[M+H]^+$ 346.1350; found 346.1350.

1*H*-*N*-(2-(3-Hydroxyphenyl)-benzo[*d*]imidazol-1-yl)-4-methylbenzamide (3i)

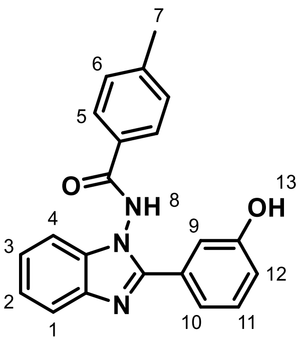

3i
$C_{21}H_{17}N_3O_2$
Mw = 343.39 g/mol

Synthesis of benzimidazole **3i** was carried out according to **GPIII** using **1a** (101.6 mg, 0.375 mmol, 1.0 eq.) and 3-hydroxybenzaldehyde (92.5 mg, 0.750 mmol, 2.0 eq.) as starting materials. Constant current electrolysis was performed at room temperature with a current density of 6.0 mA cm^{-2} until a charge of 5.0 *F* (181 C) was applied. After flash chromatography (CH_2Cl_2:MeOH) **3i** was obtained as colorless solid (92.4 mg, 0.269 mmol, 72%).

^{1}H NMR (400 MHz, DMSO-*d*$_6$) δ [ppm]: 12.10 (s, 1H, *H*-8), 9.74 (s, 1H, *H*-13), 7.91 – 7.86 (m, 2H, *H*-5), 7.77 – 7.73 (m, 1H, *H*-1), 7.42 – 7.36 (m, 5H, *H*-4, *H*-6, *H*-9, *H*-10), 7.35 – 7.24 (m, 3H, *H*-2, *H*-3, *H*-11), 6.90 – 6.86 (m, 1H, *H*-12), 2.41 (s, 3H, *H*-7).
^{13}C{^{1}H} NMR (101 MHz, DMSO-*d*$_6$) δ [ppm]: 165.6, 157.5, 151.8, 143.1, 140.3, 135.5, 130.0, 129.7, 129.4, 128.5, 127.8, 123.5, 122.8, 119.6, 118.8, 117.2, 115.2, 109.7, 21.1.
HRMS (ESI+): *m/z* calculated for $C_{21}H_{18}N_3O_2^+$ $[M+H]^+$ 344.1394; found 344.1396.

1*H*-*N*-(2-(3-Methoxyphenyl)-benzo[*d*]imidazol-1-yl)-4-methylbenzamide (3j)

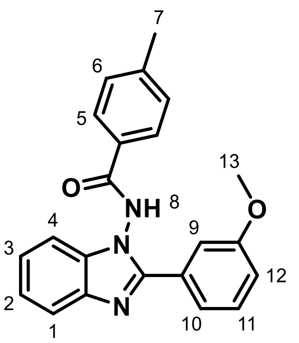

3j
$C_{22}H_{19}N_3O_2$
Mw = 357.41 g/mol

Synthesis of benzimidazole **3j** was carried out according to **GPIII** using **1a** (101.8 mg, 0.375 mmol, 1.0 eq.) and 3-methoxybenzaldehyde (91 µL, 102.0 mg, 0.750 mmol, 2.0 eq.) as starting materials. Constant current electrolysis was performed at room temperature with a current density of 6.0 mA cm^{-2} until a charge of 5.0 *F* (181 C) was applied. After flash chromatography (Cy:EtOAc) **3j** was obtained as colorless solid (110.7 mg, 0.332 mmol, 89%).

^{1}H NMR (400 MHz, DMSO-*d*$_6$) δ [ppm]: 12.17 (s, 1H, *H*-8), 7.92 – 7.85 (m, 2H, *H*-5), 7.79 – 7.74 (m, 1H, *H*-1), 7.57 – 7.50 (m, 2H, *H*-9, *H*-10), 7.45 – 7.38 (m, 4H, *H*,-4, *H*-6, *H*-11), 7.34 – 7.30 (m, 2H, *H*-2, *H*-3), 7.07 (ddd, *J* = 8.3, 2.6, 1.0 Hz, 1H, *H*-12), 3.76 (s, 3H, *H*-13), 2.40 (s, 3H, *H*-7).
^{13}C{^{1}H} NMR (101 MHz, DMSO-*d*$_6$) δ [ppm]: 165.5, 159.2, 151.6, 143.2, 140.2, 135.6, 130.0, 129.9, 129.5, 128.4, 127.7, 123.6, 123.0, 120.5, 119.7, 116.1, 113.3, 109.8, 55.2, 21.1.
HRMS (EI): *m/z* calculated for $C_{22}H_{19}N_3O_2^+$ $[M]^+$ 357.1472; found 357.1469.

1*H*-*N*-(2-(3-Cyanophenyl)-benzo[*d*]imidazol-1-yl)-4-methylbenzamide (3k)

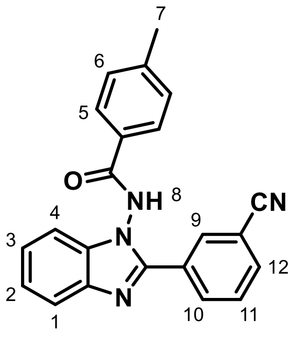

3k
$C_{22}H_{16}N_4O$
Mw = 352.40 g/mol

Synthesis of benzimidazole **3k** was carried out according to **GPIII** using **1a** (101.7 mg, 0.375 mmol, 1.0 eq.) and 3-formylbenzonitrile (97.7 mg, 0.750 mmol, 2.0 eq.) as starting materials. Constant current electrolysis was performed at room temperature with a current density of 6.0 mA cm^{-2} until a charge of 5.0 *F* (181 C) was applied. After flash chromatography (Cy:EtOAc) **3k** was obtained as colorless solid (106.6 mg, 0.302 mmol, 81%).

^{1}H NMR (400 MHz, DMSO-*d*$_6$) δ [ppm]: 12.22 (s, 1H, *H*-8), 8.37 (t, *J* = 1.4 Hz, 1H, *H*-9), 8.27 (dt, *J* = 7.9, 1.4 Hz, 1H, *H*-10), 7.99 (dt, *J* = 7.9, 1.4 Hz, 1H, *H*-12), 7.90 – 7.85 (m, 2H, *H*-5), 7.83 – 7.78 (m, 1H, *H*-1), 7.76 (t, *J* = 7.9 Hz, 1H, *H*-11), 7.54 – 7.44 (m, 1H, *H*-4), 7.46 – 7.32 (m, 4H, *H*-2, *H*-3), 2.41 (s, 3H, *H*-7).

^{13}C{^{1}H} NMR (101 MHz, DMSO-*d*$_6$) δ [ppm]: 165.7, 150.0, 143.3, 140.2, 135.5, 133.7, 132.7, 131.4, 130.3, 130.0, 129.5, 128.2, 127.7, 124.2, 123.3, 120.0, 118.2, 112.0, 110.1, 21.1.

HRMS (ESI+): *m/z* calculated for $C_{22}H_{17}N_4O^+$ $[M+H]^+$ 353.1397; found 353.1398.

1*H*-*N*-(2-(2-Methoxyphenyl)-benzo[*d*]imidazol-1-yl)-4-methylbenzamide (3l)

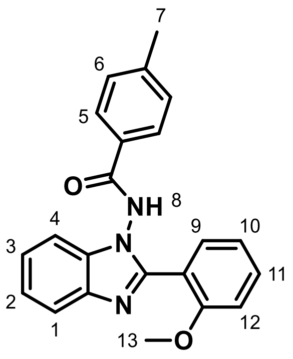

3l
$C_{22}H_{19}N_3O_2$
Mw = 357.41 g/mol

Synthesis of benzimidazole **3l** was carried out according to **GPIII** using **1a** (101.8 mg, 0.375 mmol, 1.0 eq.) and 2-methoxybenzaldehyde (102.1 mg, 0.750 mmol, 2.0 eq.) as starting materials. Constant current electrolysis was performed at room temperature with a current density of 6.0 mA cm^{-2} until a charge of 5.0 *F* (181 C) was applied. After flash chromatography (Cy:EtOAc) **3l** was obtained as colorless solid (91.6 mg, 0.256 mmol, 68%).

^{1}H NMR (400 MHz, DMSO-*d*$_6$) δ [ppm]: 11.63 (s, 1H, *H*-8), 7.80 – 7.68 (m, 3H, *H*-1, *H*-5), 7.52 – 7.43 (m, 2H, *H*-9, *H*-11), 7.40 – 7.21 (m, 5H, *H*-2, *H*-3, *H*-4, *H*-6), 7.14 (d, *J* = 8.3 Hz, 1H, *H*-12), 7.04 (td, *J* = 7.5, 1.0 Hz, 1H, *H*-10), 3.77 (s, 3H, *H*-13), 2.37 (s, 3H, *H*-7).

^{13}C{^{1}H} NMR (101 MHz, DMSO-*d*$_6$) δ [ppm]: 165.2, 157.3, 151.2, 142.6, 140.6, 134.8, 131.7, 129.2, 128.7, 127.5, 123.0, 122.2, 120.2, 119.5, 118.1, 111.5, 109.6, 55.6, 21.0.

HRMS (ESI+): *m/z* calculated for $C_{22}H_{20}N_3O_2^+$ $[M+H]^+$ 358.1550; found 358.1549.

1*H*-4-Methyl-*N*-(2-(thien-2-yl)-benzo[*d*]imidazol-1-yl)benzamide (3m)

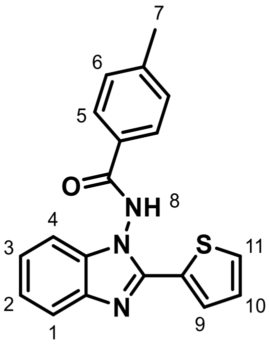

3m

$C_{19}H_{15}N_3OS$

Mw = 333.41 g/mol

Synthesis of benzimidazole **3m** was carried out according to **GPIII** using **1a** (101.7 mg, 0.375 mmol, 1.0 eq.) and thiophene-2-carbaldehyde (80 µL, 84.1 mg, 0.750 mmol, 2.0 eq.) as starting materials. Constant current electrolysis was performed at room temperature with a current density of 6.0 mA cm^{-2} until a charge of 5.0 *F* (181 C) was applied. After flash chromatography (Cy:EtOAc) **3m** was obtained as off-white solid (87.5 mg, 0.262 mmol, 70%).

^{1}H NMR (400 MHz, DMSO-*d*$_6$) *δ* [ppm]: 12.26 (s, 1H, *H*-8), 8.03 – 7.96 (m, 2H, *H*-5), 7.82 (dd, *J* = 3.7, 1.2 Hz, 1H, *H*-9), 7.78 (dd, *J* = 5.1, 1.2 Hz, 1H, *H*-11), 7.75 – 7.69 (m, 1H, *H*-1), 7.45 (d, *J* = 8.0 Hz, 2H, *H*-6), 7.41 – 7.35 (m, 1H, *H*-4), 7.34 – 7.26 (m, 2H, *H*-2, *H*-3), 7.21 (dd, *J* = 5.1, 3.7 Hz, 1H, *H*-10), 2.44 (s, 3H, *H*-7).

^{13}C{^{1}H} NMR (101 MHz, DMSO-*d*$_6$) *δ* [ppm]: 165.7, 146.8, 143.3, 140.4, 135.1, 130.3, 130.0, 129.5, 128.4, 128.3, 128.2, 127.9, 123.5, 123.1, 119.3, 109.5, 21.2.

HRMS (ESI+): *m/z* calculated for $C_{19}H_{16}N_3OS^+$ [M+H]$^+$ 334.1009; found 334.1007.

1*H*-*N*-(2-(Furan-2-yl)-benzo[*d*]imidazol-1-yl)-4-methylbenzamide (3n)

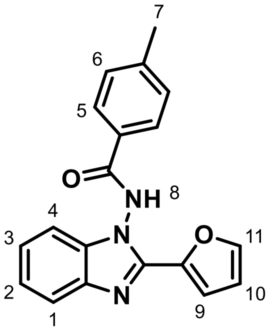

3n

$C_{19}H_{15}N_3O_2$

Mw = 317.35 g/mol

Synthesis of benzimidazole **3n** was carried out according to **GPIII** using **1a** (101.7 mg, 0.375 mmol, 1.0 eq.) and furan-2-carbaldehyde (63 µL, 72.1 mg, 0.750 mmol, 2.0 eq.) as starting materials. Constant current electrolysis was performed at room temperature with a current density of 6.0 mA cm^{-2} until a charge of 5.0 *F* (181 C) was applied. After flash chromatography (Cy:EtOAc) **3n** was obtained as off-white solid (83.5 mg, 0.263 mmol, 70%).

^{1}H NMR (400 MHz, DMSO-*d*$_6$) *δ* [ppm]: 12.16 (s, 1H, *H*-8), 8.02 – 7.95 (m, 2H, *H*-5), 7.92 (dd, *J* = 1.8, 0.7 Hz, 1H, *H*-11), 7.77 – 7.68 (m, 1H, *H*-1), 7.47 – 7.36 (m, 3H, *H*-4, *H*-6), 7.35 – 7.26 (m, 2H, *H*-2, *H*-3), 7.11 (dd, *J* = 3.5, 0.7 Hz, 1H, *H*-9), 6.69 (dd, *J* = 3.5, 1.8 Hz, 1H, *H*-10), 2.43 (s, 3H, *H*-7).

^{13}C{^{1}H} NMR (101 MHz, DMSO-*d*$_6$) *δ* [ppm]: 165.8, 145.4, 143.5, 143.4, 143.2, 140.4, 134.9, 129.5, 128.5, 127.9, 123.7, 123.1, 119.6, 112.5, 112.2, 109.5, 21.2.

HRMS (ESI+): *m/z* calculated for $C_{19}H_{15}N_3O_2^+$ [M]$^+$ 317.1159; found 317.1154.

1*H*-4-Methyl-*N*-(2-(5-methylfuran-2-yl)-benzo[*d*]imidazol-1-yl)benzamide (3o)

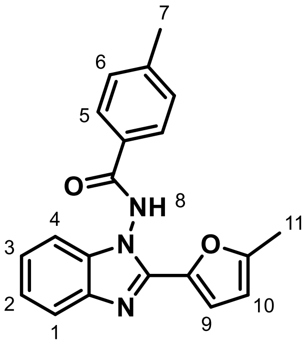

3o

$C_{20}H_{17}N_3O_2$

Mw = 331.38 g/mol

Synthesis of benzimidazole **3o** was carried out according to **GPIII** using **1a** (101.9 mg, 0.375 mmol, 1.0 eq.) and 5-methylfuran-2-carbaldehyde (80 µL, 82.6 mg, 0.750 mmol, 2.0 eq.) as starting materials. Constant current electrolysis was performed at room temperature with a current density of 6.0 mA cm^{-2} until a charge of 5.0 *F* (181 C) was applied. After flash chromatography (Cy:EtOAc) **3o** was obtained as off-white solid (84.9 mg, 0.256 mmol, 68%).

^{1}H NMR (400 MHz, DMSO-*d*$_6$) *δ* [ppm]: 12.12 (s, 1H, *H*-8), 8.02 – 7.94 (m, 2H, *H*-5), 7.74 – 7.65 (m, 1H, *H*-1), 7.44 (d, *J* = 8.0 Hz, 2H, *H*-6), 7.41 – 7.37 (m, 1H, *H*-7), 7.34 – 7.24 (m, 2H, *H*-2, *H*-3), 7.00 (d, *J* = 3.4 Hz, 1H, *H*-9), 6.31 (d, *J* = 3.4 Hz, 1H, *H*-10), 2.43 (s, 3H, *H*-7), 2.29 (s, 3H, *H*-11).
^{13}C{^{1}H} NMR (101 MHz, DMSO-*d*$_6$) *δ* [ppm]: 165.9, 154.4, 143.6, 143.1, 141.9, 140.5, 134.9, 129.5, 128.6, 127.8, 123.4, 122.9, 119.4, 113.6, 109.4, 108.5, 21.2, 13.3.
HRMS (ESI+): *m/z* calculated for $C_{20}H_{18}N_3O_2^+$ $[M]^+$ 332.1394; found 332.1392.

1*H*-4-Methyl-*N*-(2-(thien-3-yl)-benzo[*d*]imidazol-1-yl)benzamide (3p)

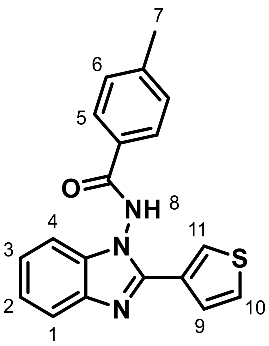

3p

$C_{19}H_{15}N_3OS$

Mw = 333.41 g/mol

Synthesis of benzimidazole **3p** was carried out according to **GPIII** using **1a** (101.7 mg, 0.375 mmol, 1.0 eq.) and thiophene-2-carbaldehyde (66 µL, 84.0 mg, 0.750 mmol, 2.0 eq.) as starting materials. Constant current electrolysis was performed at room temperature with a current density of 6.0 mA cm^{-2} until a charge of 5.0 *F* (181 C) was applied. After flash chromatography (Cy:EtOAc) **3p** was obtained as off-white solid (102.6 mg, 0.287 mmol, 77%).

^{1}H NMR (400 MHz, DMSO-*d*$_6$) *δ* [ppm]: 12.19 (s, 1H, *H*-8), 8.17 (t, *J* = 2.1 Hz, 1H, *H*-11), 7.98 – 7.93 (m, 2H, *H*-5), 7.74 – 7.70 (m, 3H, *H*-1, *H*-9, *H*-10), 7.44 – 7.40 (m, 2H, *H*-6), 7.39 – 7.35 (m, 1H, *H*-4), 7.32 – 7.27 (m, 2H, *H*-2, *H*-3), 2.42 (s, 3H, *H*-7).
^{13}C{^{1}H} NMR (101 MHz, DMSO-*d*$_6$) *δ* [ppm]: 165.7, 148.0, 143.2, 140.3, 135.1, 129.8, 129.5, 128.4, 127.9, 127.4, 127.3, 126.7, 123.4, 122.9, 119.5, 109.6, 21.2.
HRMS (ESI+): *m/z* calculated for $C_{19}H_{16}N_3OS^+$ $[M+H]^+$ 334.1009; found 334.1010.

1*H*-*N*-(2-(4-Hydroxy-3-methoxyphenyl)-benzo[*d*]imidazol-1-yl)-4-methylbenzamide (3q)

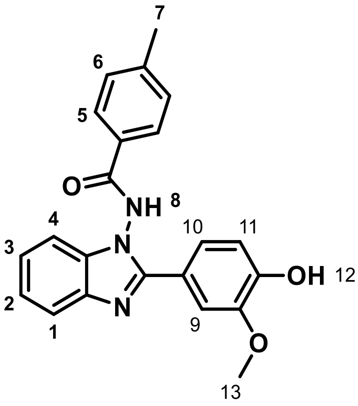

3q
$C_{22}H_{19}N_3O_3$
Mw = 373.41 g/mol

Synthesis of benzimidazole **3q** was carried out according to **GPIII** using **1a** (101.6 mg, 0.375 mmol, 1.0 eq.) and 4-hydroxy-3-methoxybenzaldehyde (114.2 mg, 0.750 mmol, 2.0 eq.) as starting materials. Constant current electrolysis was performed at room temperature with a current density of 6.0 mA cm^{-2} until a charge of 5.0 *F* (181 C) was applied. After flash chromatography (CH_2Cl_2/MeOH) **3q** was obtained as colorless solid (80.4 mg, 0.215 mmol, 58%).

^{1}H NMR (400 MHz, DMSO-*d*$_6$) δ [ppm]: 12.11 (s, 1H, *H*-8), 9.62 (s, 1H, *H*-12), 7.91 (d, *J* = 8.2 Hz, 2H, *H*-5), 7.75 – 7.69 (m, 1H, *H*-1), 7.54 (d, *J* = 2.0 Hz, 1H, *H*-9), 7.45 (dd, *J* = 8.3, 2.0 Hz, 1H, *H*-10), 7.42 – 7.36 (m, 3H, *H*-6, *H*-4), 7.31 – 7.25 (m, 2H, *H*-2, *H*-3), 6.88 (d, *J* = 8.3 Hz, 1H, *H*-11), 3.74 (s, 3H, *H*-13), 2.41 (s, 3H, *H*-7).
^{13}C{^{1}H} NMR (101 MHz, DMSO-*d*$_6$) δ [ppm]: 165.5, 152.1, 148.7, 147.5, 143.1, 140.3, 135.7, 129.5, 128.5, 127.7, 123.0, 122.7, 121.5, 119.8, 119.2, 115.6, 112.0, 109.5, 55.6, 21.2.
HRMS (EI): *m/z* calculated for $C_{22}H_{19}N_3O_3^+$ $[M]^+$ 373.1421; found 373.1420.

1*H*-*N*-(2-((1*R*,5*S*)-6,6-Dimethylbicyclo[3.1.1]hept-2-en-2-yl)-benzo[*d*]imidazol-1-yl)-4-methylbenzamide (3r)

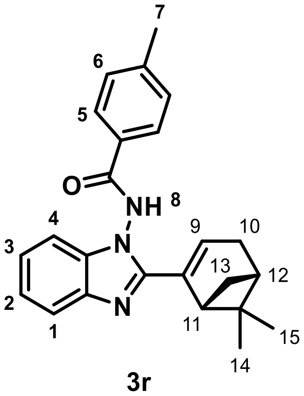

3r
$C_{24}H_{25}N_3O$
Mw = 371.48 g/mol

Synthesis of benzimidazole **3r** was carried out according to **GPIII** using **1a** (101.6 mg, 0.375 mmol, 1.0 eq.) and (1*R*)-(-)-myrtenal (109 µL, 113.0 mg, 0.750 mmol, 2.0 eq.) as starting materials. Constant current electrolysis was performed at room temperature with a current density of 6.0 mA cm^{-2} until a charge of 5.0 *F* (181 C) was applied. After flash chromatography (Cy/EtOAc) **3r** was obtained as pale-yellow solid (81.2 mg, 0.219 mmol, 58%).

^{1}H NMR (400 MHz, DMSO-*d*$_6$) δ [ppm]: 11.91 (s, 1H, *H*-8), 7.90 (d, *J* = 7.9 Hz, 2H, *H*-5), 7.66 – 7.61 (m, 1H, *H*-1), 7.41 (d, *J* = 7.9 Hz, 2H, *H*-6), 7.31 – 7.21 (m, 3H, *H*-2, *H*-3, *H*-4), 6.49 (d, *J* = 11.5 Hz, 1H, *H*-9), 3.10 – 3.01 (m, 1H, *H*-11), 2.46 – 2.26 (s, 5H, *H*-7, *H*-10), 2.17 – 2.08 (m, 1H, *H*-12), 1.39 – 1.12 (m, 5H, *H*-13, *H*-14 or *H*-15), 0.79 (d, *J* = 24.7 Hz, 3H, *H*-14 or *H*-15).
^{13}C{^{1}H} NMR (101 MHz, DMSO-*d*$_6$) δ [ppm]: 165.3, 151.6, 142.9, 140.0, 136.8, 135.5, 129.4, 128.7, 127.9, 127.6, 123.2, 122.4, 119.3, 109.2, 43.7, 37.2, 31.9, 31.0, 25.9, 21.1, 20.9.
HRMS (ESI+): *m/z* calculated for $C_{24}H_{26}N_3O^+$ $[M+H]^+$ 372.2070; found 372.2073.

1*H*-*N*-(Benzo[*d*]imidazol-1-yl)-4-methylbenzamide (3s)

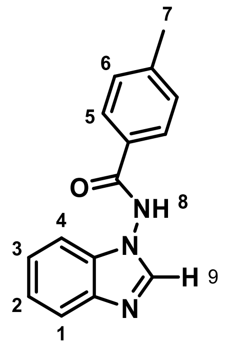

3s
$C_{15}H_{13}N_3O$
Mw = 251.29 g/mol

Synthesis of benzimidazole **3s** was carried out according to **GPIII** using **1a** (101.5 mg, 0.375 mmol, 1.0 eq.) and formaldehyde (40% in H_2O, 57 µL, 23.0 mg, 0.750 mmol, 2.0 eq.) as starting materials. Constant current electrolysis was performed at room temperature with a current density of 6.0 mA cm^{-2} until a charge of 5.0 *F* (181 C) was applied. After flash chromatography (Cy/EtOAc) **3s** was obtained as pale-yellow solid (26.3 mg, 0.105 mmol, 28%).

^{1}H NMR (400 MHz, DMSO-*d*$_6$) *δ* [ppm]: 12.00 (s, 1H, *H*-8), 8.40 (s, 1H, *H*-9), 7.94 (d, *J* = 8.1 Hz, 2H, H-5), 7.75 – 7.71 (m, 1H, *H*-1), 7.43 – 7.39 (m, 3H, *H*-4, *H*-6), 7.33 – 7.25 (m, 2H, *H*-2, *H*-3), 2.42 (s, 3H, *H*-7).
^{13}C{^{1}H} NMR (101 MHz, DMSO-*d*$_6$) *δ* [ppm]: 166.0, 144.4, 143.0, 140.9, 133.5, 129.3, 128.5, 127.9, 123.4, 122.3, 119.9, 109.5, 21.1.
HRMS (ESI+): *m/z* calculated for $C_{15}H_{14}N_3O^+$ [M+H]$^+$ 252.1131; found 252.1130.

1*H*-*N*-(2-Cyclohexyl-benzo[*d*]imidazol-1-yl)-4-methylbenzamide (3t)

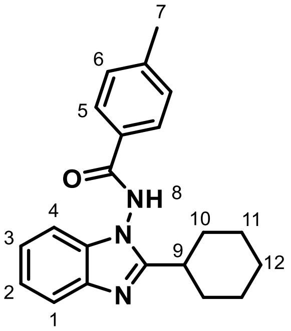

3t
$C_{21}H_{23}N_3O$
Mw = 333.44 g/mol

Synthesis of benzimidazole **3t** was carried out according to **GPIII** using **1a** (101.9 mg, 0.375 mmol, 1.0 eq.) and cyclohexanecarbaldehyde (91 µL, 84.1 mg, 0.750 mmol, 2.0 eq.) as starting materials. Constant current electrolysis was performed at room temperature with a current density of 6.0 mA cm^{-2} until a charge of 5.0 *F* (181 C) was applied. After flash chromatography (Cy:EtOAc) **3t** was obtained as colorless solid (98.4 mg, 0.295 mmol, 79%).

^{1}H NMR (400 MHz, DMSO-*d*$_6$) *δ* [ppm]: 11.81 (s, 1H, *H*-8), 7.98 – 7.90 (m, 2H, *H*-5), 7.66 – 7.57 (m, 1H, *H*-1), 7.42 (d, *J* = 8.0 Hz, 2H, *H*-6), 7.33 – 7.24 (m, 1H, *H*-4), 7.24 – 7.16 (m, 2H, *H*-2, *H*-3), 2.76 (tt, *J* = 11.5, 3.5 Hz, 1H, *H*-9), 2.42 (s, 3H, *H*-7), 2.01 – 1.21 (m, 10H, *H*-10, *H*-11, *H*-12).
^{13}C{^{1}H} NMR (101 MHz, DMSO-*d*$_6$) *δ* [ppm]: 165.8, 158.6, 143.1, 140.1, 134.2, 129.4, 128.6, 127.8, 122.5, 122.0, 119.1, 109.0, 34.8, 31.3, 25.5, 22.1, 21.2.
HRMS (EI): *m/z* calculated for $C_{21}H_{23}N_3O^+$ [M]$^+$ 333.1836; found 333.1836.

1*H*-4-Methyl-*N*-(2-neopentyl-benzo[*d*]imidazol-1-yl)benzamide (3u)

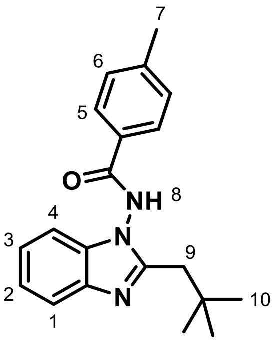

3u
$C_{20}H_{23}N_3O$
Mw = 321.42 g/mol

Synthesis of benzimidazole **3u** was carried out according to **GPIII** using **1a** (101.6 mg, 0.375 mmol, 1.0 eq.) and 3,3-dimethylbutyraldehyde (94 µL, 75.0 mg, 0.750 mmol, 2.0 eq.) as starting materials. Constant current electrolysis was performed at room temperature with a current density of 6.0 mA cm^{-2} until a charge of 5.0 *F* (181 C) was applied. After flash chromatography (Cy:EtOAc) **3u** was obtained as colorless solid (88.3 mg, 0.274 mmol, 73%).

^{1}H NMR (400 MHz, DMSO-*d*$_6$) *δ* [ppm]: 11.83 (s, 1H, *H*-8), 7.94 (d, *J* = 8.3 Hz, 2H, *H*-5), 7.67 – 7.61 (m, 1H, *H*-1), 7.42 (d, *J* = 8.3 Hz, 2H, *H*-6), 7.27 – 7.18 (m, 3H, *H*-2, *H*-3, *H*-4), 2.64 (d, *J* = 6.5 Hz, 2H, *H*-9), 2.42 (s, 3H, *H*-7), 1.03 (s, 9H, *H*-10).
^{13}C{^{1}H} NMR (101 MHz, DMSO-*d*$_6$) *δ* [ppm]: 165.6, 153.2, 143.0, 140.2, 134.0, 129.4, 128.6, 127.8, 122.4, 122.0, 119.0, 109.3, 38.6, 31.8, 29.6, 21.2.
HRMS (CI): *m/z* calculated for $C_{20}H_{24}N_3O^+$ [M+H]$^+$ 322.1914; found 322.1919.

1*H*-*N*-(2-Benzyl-benzo[*d*]imidazol-1-yl)-4-methylbenzamide (3v)

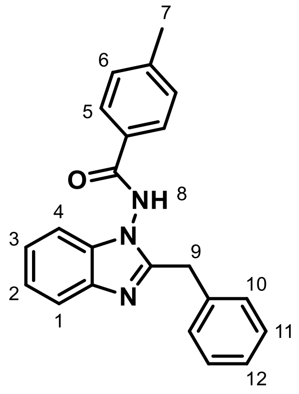

3v
$C_{22}H_{19}N_3O$
Mw = 341.41 g/mol

Synthesis of benzimidazole **3v** was carried out according to **GPIII** using **1a** (101.5 mg, 0.375 mmol, 1.0 eq.) and phenylacetaldehyde (87 µL, 90.0 mg, 0.750 mmol, 2.0 eq.) as starting materials. Constant current electrolysis was performed at room temperature with a current density of 6.0 mA cm^{-2} until a charge of 5.0 *F* (181 C) was applied. After flash chromatography (Cy:EtOAc) **3v** was obtained as colorless film (50.8 mg, 0.149 mmol, 40%).

^{1}H NMR (400 MHz, DMSO-*d*$_6$) δ [ppm]: 11.88 (s, 1H, *H*-8), 7.90 (d, *J* = 8.2 Hz, 2H, *H*-5), 7.65 – 7.59 (m, 1H, *H*-1), 7.41 (d, *J* = 8.2 Hz, 2H, *H*-6), 7.31 – 7.26 (m, 5H, *H*-10, *H*-11, *H*-12), 7.26 – 7.19 (m, 3H, *H*-2, *H*-3, *H*-4), 4.13 (d, *J* = 10.9 Hz, 2H, *H*-9), 2.42 (s, 3H, *H*-7).
^{13}C{^{1}H} NMR (101 MHz, DMSO-*d*$_6$) δ [ppm]: 165.8, 153.8, 143.1, 140.2, 136.5, 134.3, 129.4, 129.1, 128.6, 128.5, 127.9, 126.6, 122.8, 122.3, 119.2, 109.3, 32.2, 21.2.
HRMS (ESI+): *m/z* calculated for $C_{22}H_{20}N_3O^+$ [M+H]$^+$ 342.1601; found 342.1602.

1*H*-4-Methyl-*N*-(2-propyl-benzo[*d*]imidazol-1-yl)benzamide (3w)

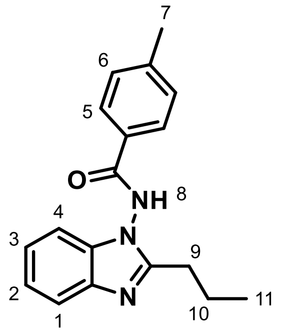

3w
$C_{18}H_{19}N_3O$
Mw = 293.37 g/mol

Synthesis of benzimidazole **3w** was carried out according to **GPIII** using **1a** (101.5 mg, 0.375 mmol, 1.0 eq.) and butyraldehyde (68 µL, 54.0 mg, 0.750 mmol, 2.0 eq.) as starting materials. Constant current electrolysis was performed at room temperature with a current density of 6.0 mA cm^{-2} until a charge of 5.0 *F* (181 C) was applied. After flash chromatography (Cy:EtOAc) **3w** was obtained as colorless film/solid (93.1 mg, 0.317 mmol, 85%).

^{1}H NMR (400 MHz, DMSO-*d*$_6$) δ [ppm]: 11.83 (s, 1H, *H*-8), 7.94 (d, *J* = 8.2 Hz, 2H, *H*-5), 7.65 – 7.59 (m, 1H, *H*-1), 7.42 (d, *J* = 8.0 Hz, 2H, *H*-6), 7.31 – 7.26 (m, 1H, *H*-4), 7.25 – 7.18 (m, 2H, *H*-2, *H*-3), 2.69 (t, *J* = 7.4 Hz, 2H, *H*-9), 2.42 (s, 3H, *H*-7), 1.78 (h, *J* = 7.4 Hz, 2H, *H*-10), 0.96 (t, *J* = 7.4 Hz, 3H, *H*-11).
^{13}C{^{1}H} NMR (101 MHz, DMSO-*d*$_6$) δ [ppm]: 165.7, 155.0, 143.0, 140.2, 134.4, 129.4, 128.5, 127.8, 122.4, 122.0, 119.0, 109.0, 27.7, 21.2, 19.9, 13.8.
HRMS (EI): *m/z* calculated for $C_{18}H_{19}N_3O^+$ [M]$^+$ 293.1523; found 293.1522.

Scale-Up

The scale-up experiment was performed in a divided glass cell equipped with PTFE stoppers and sleeves, electrode holders, electrodes, and a cross-shaped stirring bar. Benzaldehyde was dissolved in 150 mL of electrolyte solution in an AcOH/MeOH mixture at room temperature in the cathode compartment. The anode compartment was filled with an equal volume of electrolyte solution. Under stirring (cross stirring bar 120 rpm), **1a** was added to the cathode compartment and dispersed in the mixture. The mixture was subjected to galvanostatic electrolysis using electrodes with a relevant surface area of 6.8 cm^2 (surface: 60 mm x 20 mm, immersion depth: 3.4 mm). After electrolysis, the catholyte was removed under reduced pressure and the crude product purified via flash chromatography (SiO_2, 6% EtOAc in Cy to 94% EtOAc).

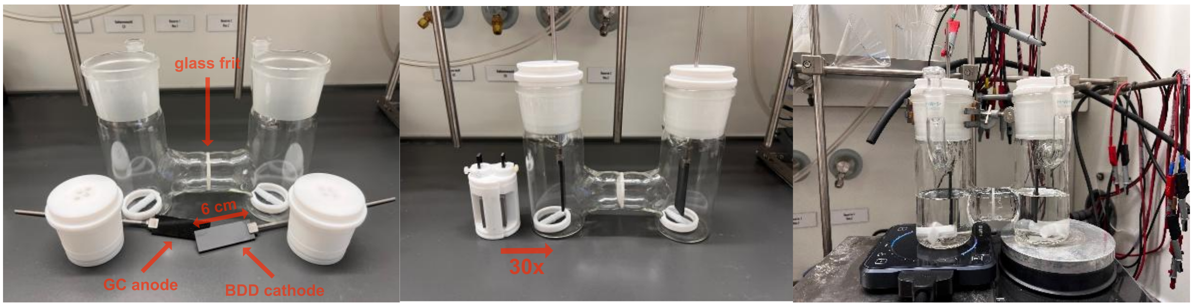

***Figure S5**: Divided glass cell with a GC anode and BDD electrode (left), the assembled divided PTFE screening cell in comparison to the undivided glass cell (middle), and the assembled divided glass cell with electrolyte and mounted electrodes (right).*

Scale-up of 2a

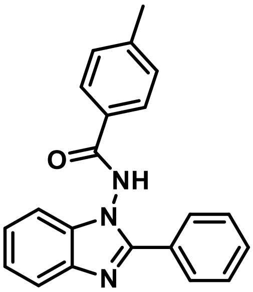

2a
$C_{21}H_{17}N_3O$
Mw = 327.39 g/mol

Synthesis of benzimidazole **2a** was carried out using **1a** (3.050 g, 11.25 mmol, 1.0 eq.) and benzaldehyde (2.32 mL, 2.388 g, 22.50 mmol, 2.0 eq.) as starting materials. Constant current electrolysis was performed at room temperature with a current density of 6.0 mA cm^{-2} until a charge of 5.0 *F* (5427 C) was applied. After flash chromatography (Cy:EtOAc) **2a** was obtained as off-white solid (2.734 g, 8.35 mmol, 74%).

The analytical data matches the results of the divided PTFE screening cells.

References

(1) Gütz, C.; Klöckner, B.; Waldvogel, S. R. Electrochemical Screening for Electroorganic Synthesis. *Org. Process Res. Dev.* **2016**, *20* (1), 26–32. https://doi.org/10.1021/acs.oprd.5b00377.

(2) Pollok, D.; Gleede, B.; Stenglein, A.; Waldvogel, S. R. Preparative Batch-Type Electrosynthesis: A Tutorial. *Aldrichim. Acta* **2021**, *54*, 3–15.

(3) Dörr, M.; Hielscher, M. M.; Proppe, J.; Waldvogel, S. R. Electrosynthetic Screening and Modern Optimization Strategies for Electrosynthesis of Highly Value-Added Products. *ChemElectroChem* **2021**, *8* (14), 2621–2629. https://doi.org/10.1002/celc.202100318.

Appendix

Crystallographic Data

3s was crystallized from slow diffusion of cyclohexane into EtOAc.

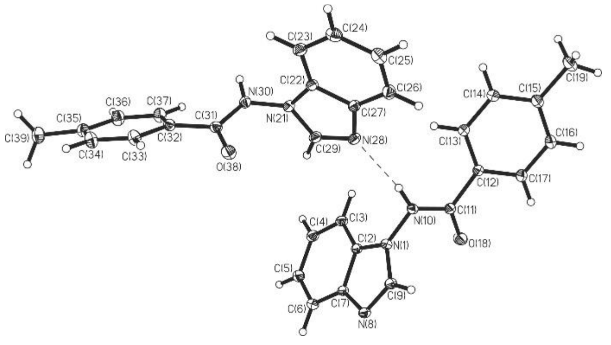

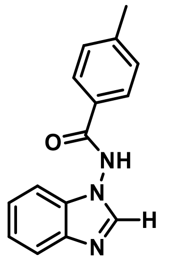

3s

Figure S6: *Thermal ellipsoid plot of the two crystallographically independent molecules in* ***3s*** *drawn at the 50% probability level.*

Table S3: *Crystallographic data for* ***3s****.*

CCDC number	2514730
Empirical formula	$C_{15}H_{13}N_3O$
Formula weight/g mol^{-1}	251.28
Temperature/K	100(2)
Wavelength/Å	0.71073
Crystal system	monoclinic
Space group	P2(1); No. 4
a/Å	9.8852(3)
b/Å	10.1151(3)
c/Å	12.3690(3)
α/°	90
β/°	90.8849(12)
γ/°	90
Volume/Å^3	1236.63(6)
Z	4
ρ_{calc}/g cm^{-3}	1.350
μ/mm^{-1}	0.088
F(000)	528
Crystal size/mm^3	0.268 x 0.243 x 0.060
Θ range for data collection/°	2.06 to 45.33
Index ranges	-19≤h≤19, -20≤k≤19, -24≤l≤24
Reflections collected	200269
Independent reflections	20667 [R_{int} = 0.0382]
Absorption correction	Numerical Mu from formula
Max. and min. transmission	0.99 and 0.94
Refinement method	Full-matrix least-squares on F^2
Data/restraints/parameters	20667/1/354
Goodness-of-fit on F^2	1.059
Final R indices [I>2σ (I)]	R_1 = 0.0320, wR_2 = 0.0897
R indices (all data)	R_1 = 0.0366, wR_2 = 0.0937
Largest diff. peak and hole/e Å^{-3}	0.481 and -0.224

NMR Spectra

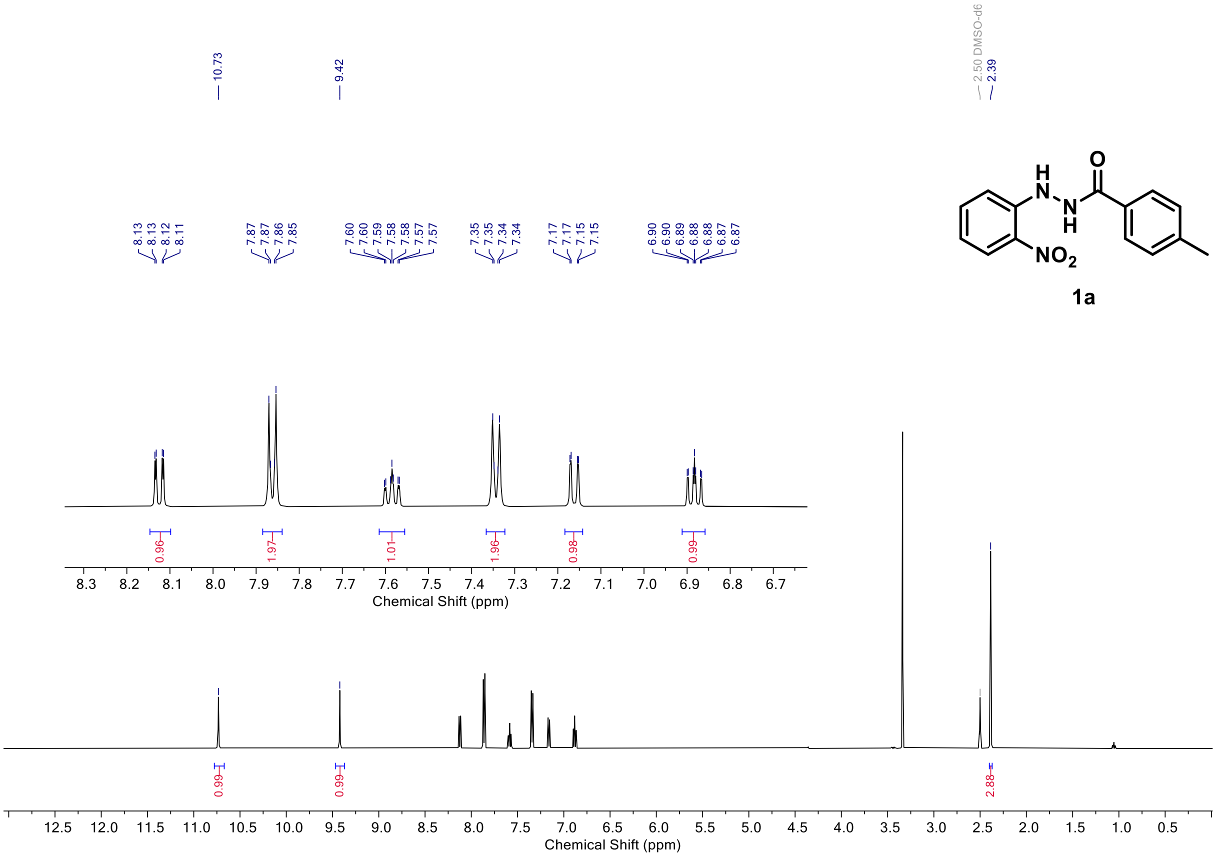

Figure S7: ^{1}H NMR (500 MHz, DMSO-d_6): 4-Methyl-*N*'-(2-nitrophenyl)benzohydrazide (**1a**).

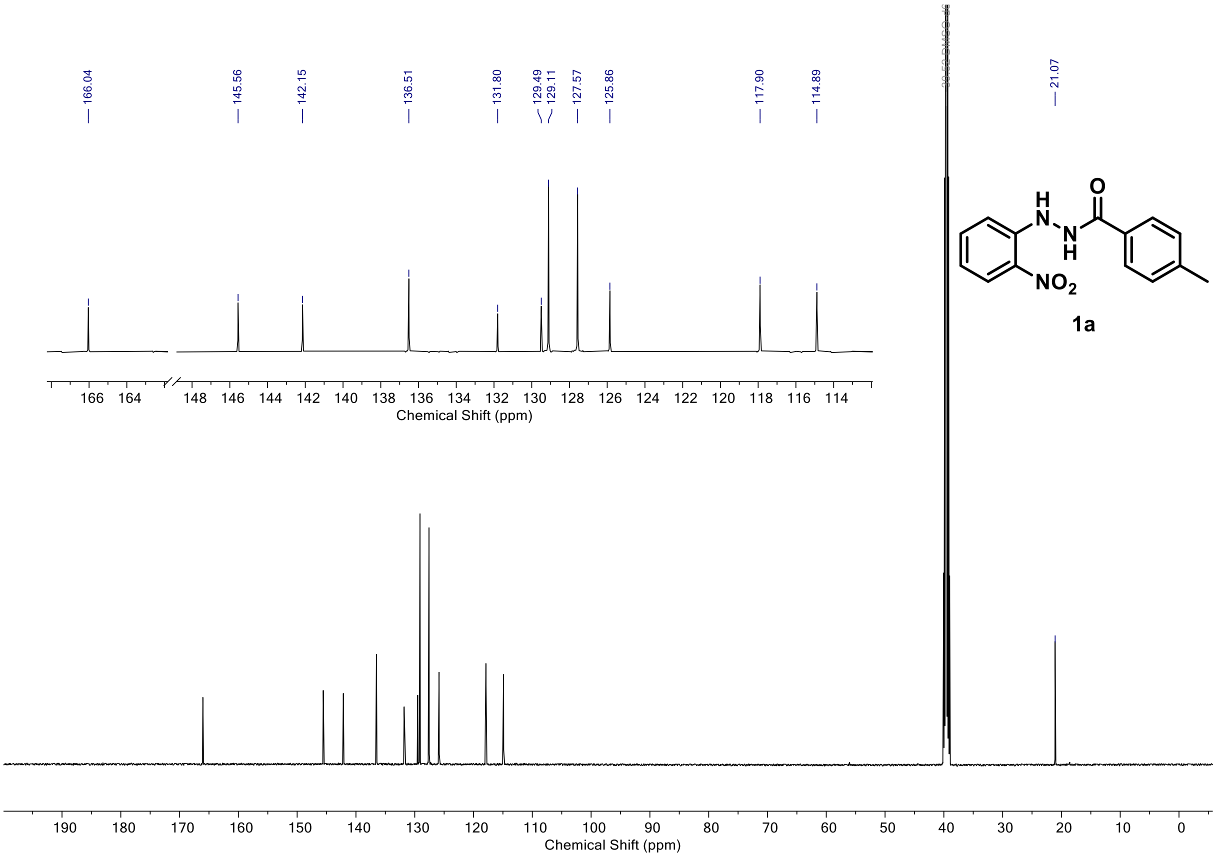

Figure S8: $^{13}C\{^{1}H\}$ NMR (126 MHz, DMSO-d_6): 4-Methyl-*N*'-(2-nitrophenyl)benzohydrazide (**1a**).

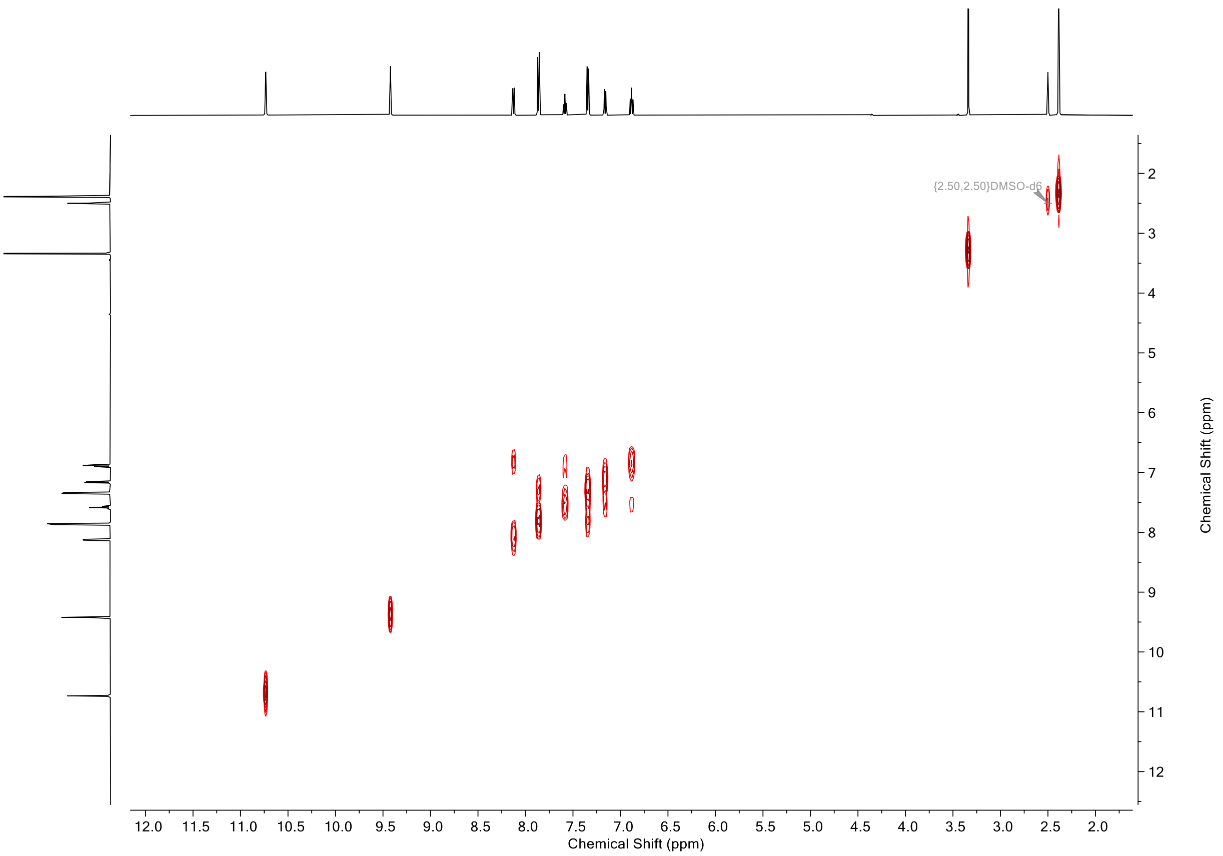

Figure S9: COSY (DMSO-d_6): 4-Methyl-*N*'-(2-nitrophenyl)benzohydrazide (**1a**).

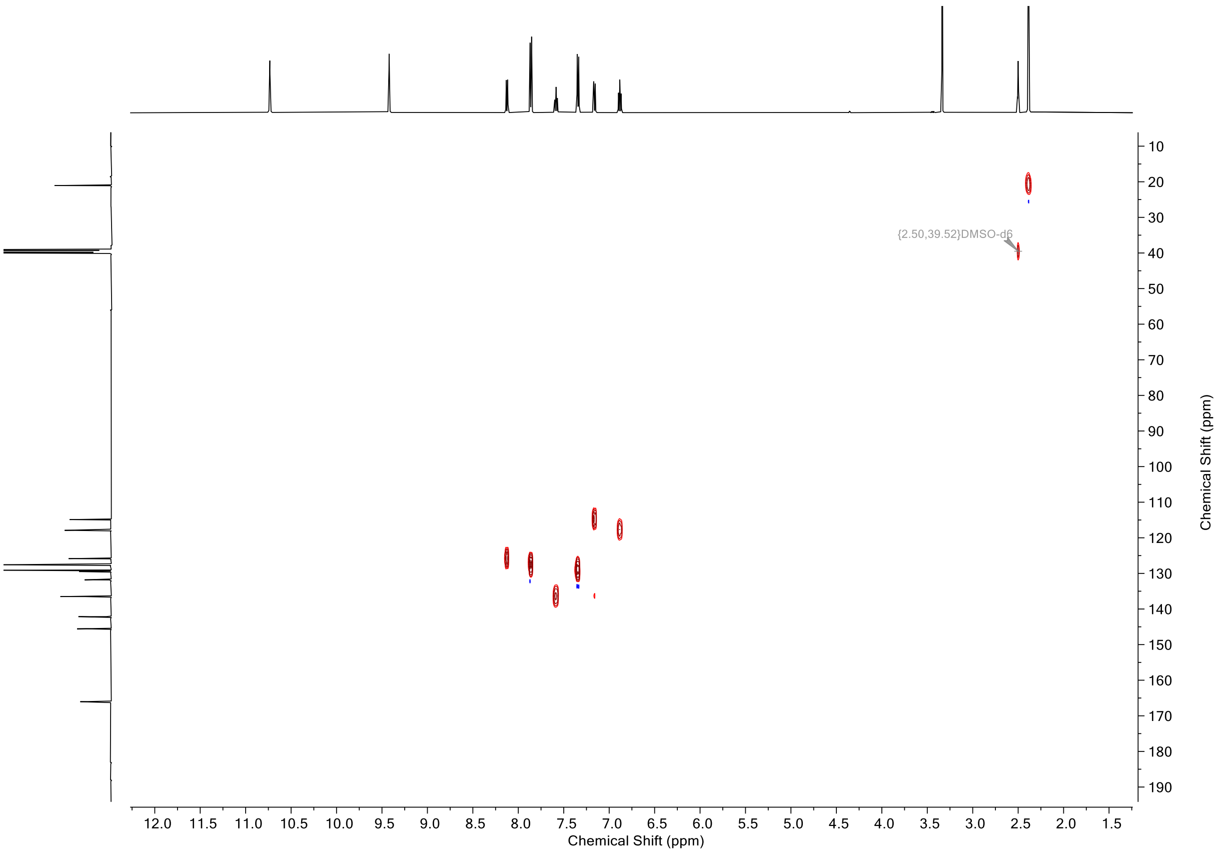

Figure S10: HSQC (DMSO-d_6): 4-Methyl-*N*'-(2-nitrophenyl)benzohydrazide (**1a**).

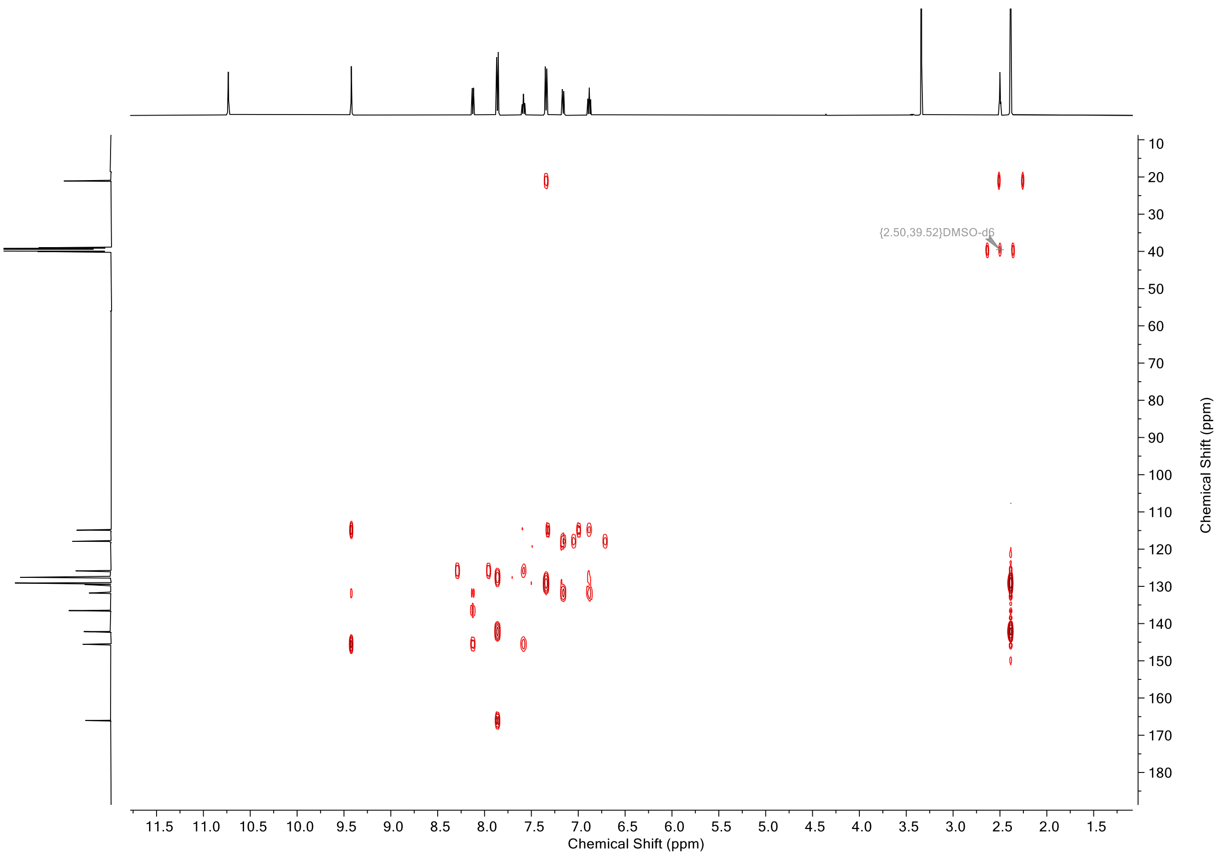

Figure S11: HMBC (DMSO-d_6): 4-Methyl-*N*'-(2-nitrophenyl)benzohydrazide (**1a**).

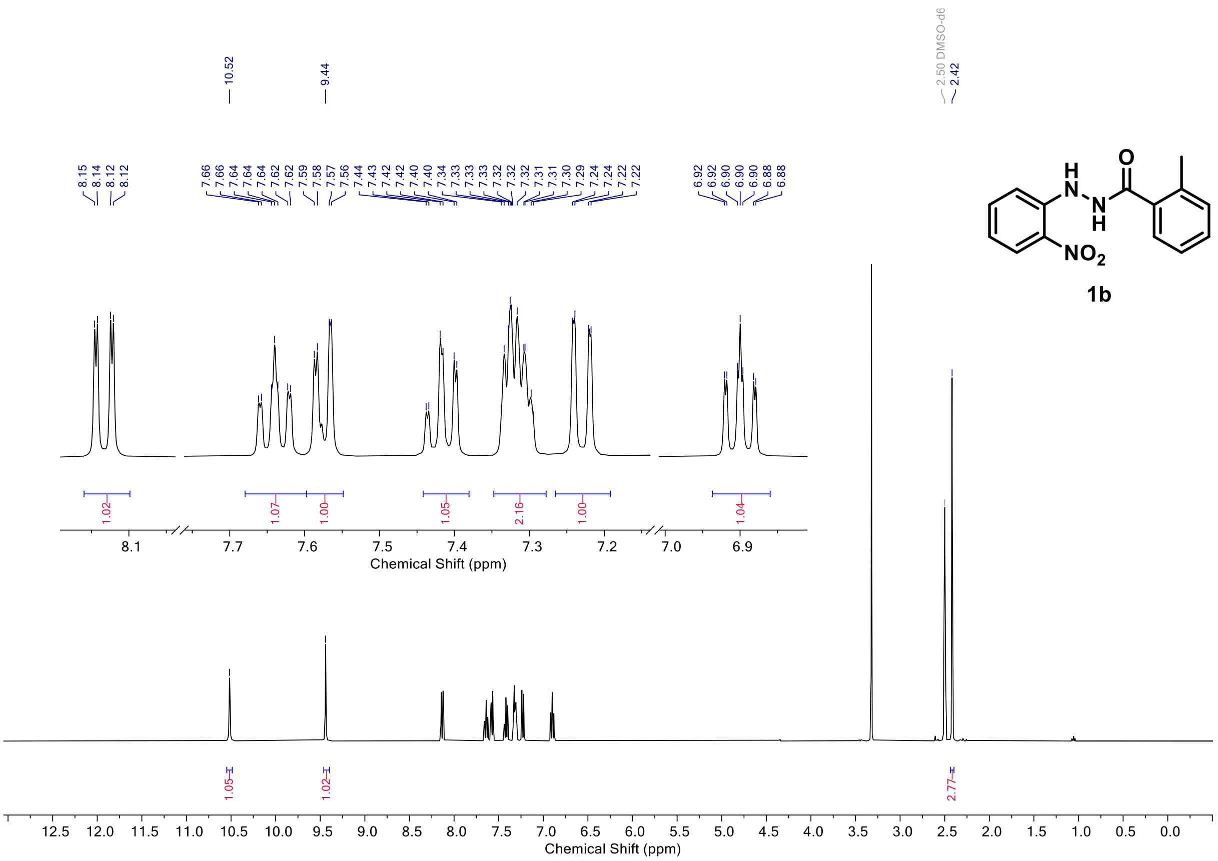

Figure S12: ^{1}H NMR (400 MHz, DMSO-d_6): 2-Methyl-*N*'-(2-nitrophenyl)benzohydrazide (**1b**).

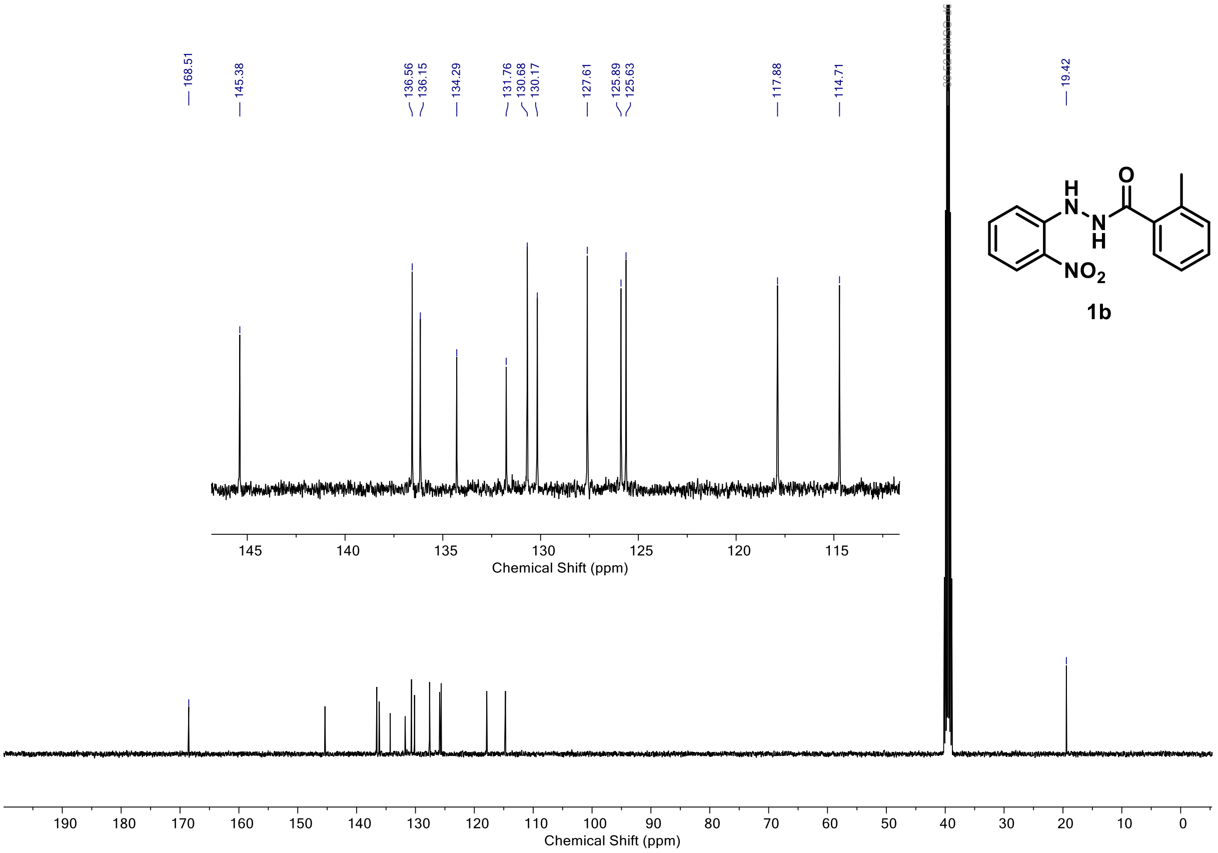

Figure S13: $^{13}C\{^{1}H\}$ NMR (101 MHz, DMSO-d_6): 2-Methyl-*N*'-(2-nitrophenyl)benzohydrazide (**1b**).

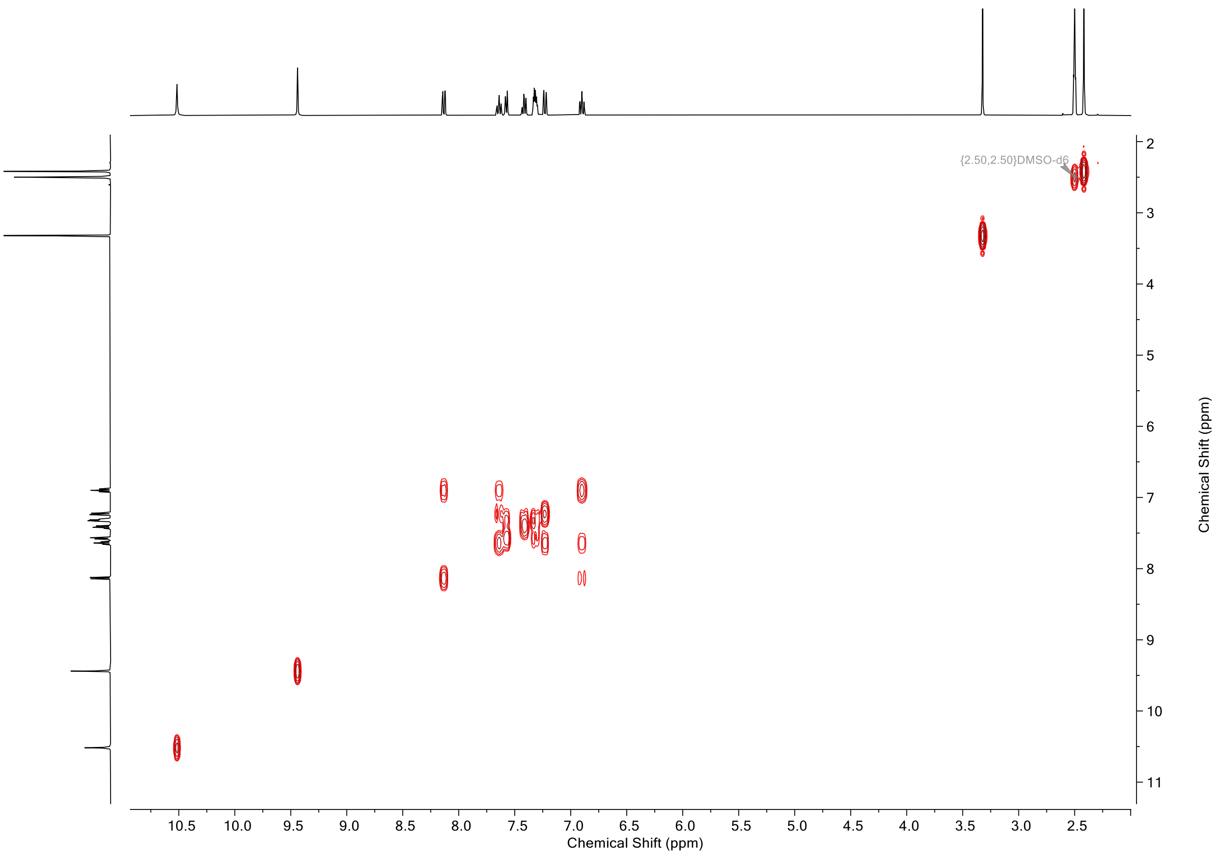

Figure S14: COSY (DMSO-d_6): 2-Methyl-*N*'-(2-nitrophenyl)benzohydrazide (**1b**).

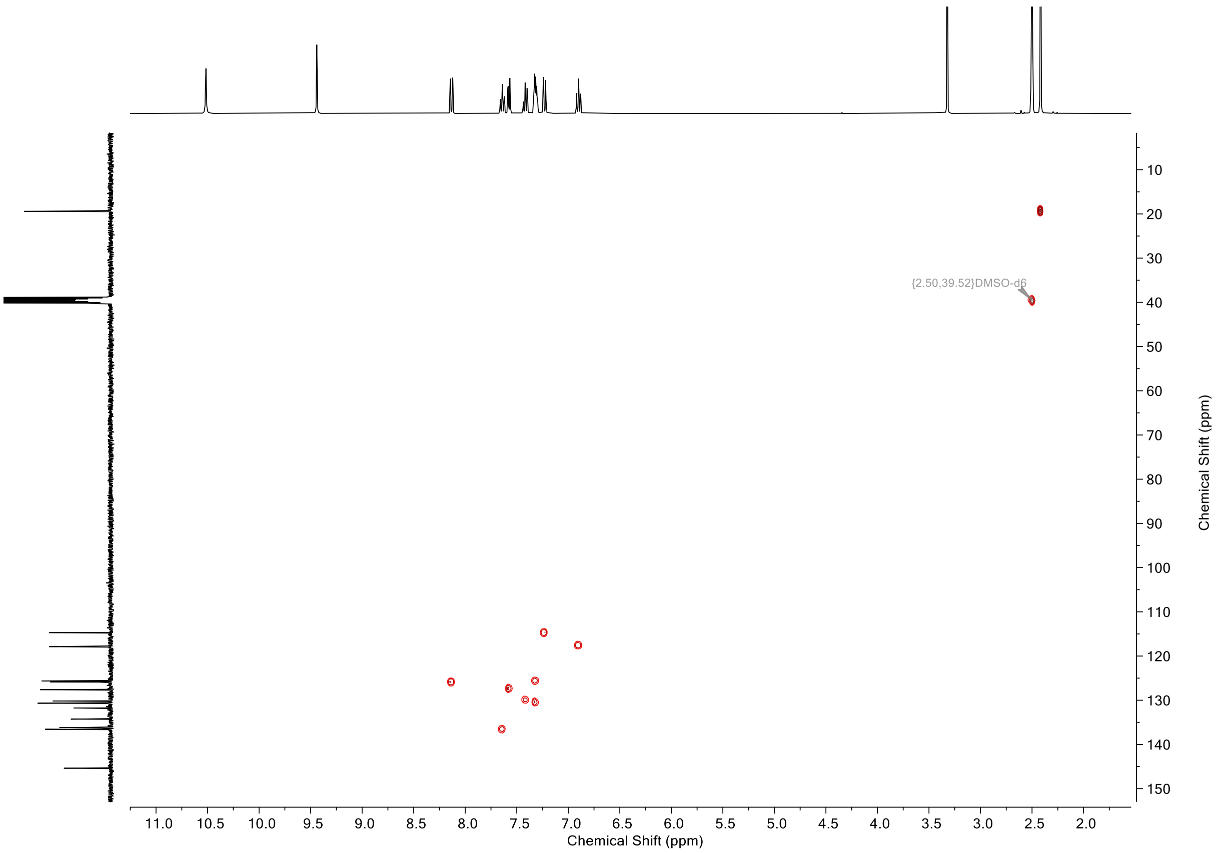

Figure S15: HSQC (DMSO-d_6): 2-Methyl-*N*'-(2-nitrophenyl)benzohydrazide (**1b**).

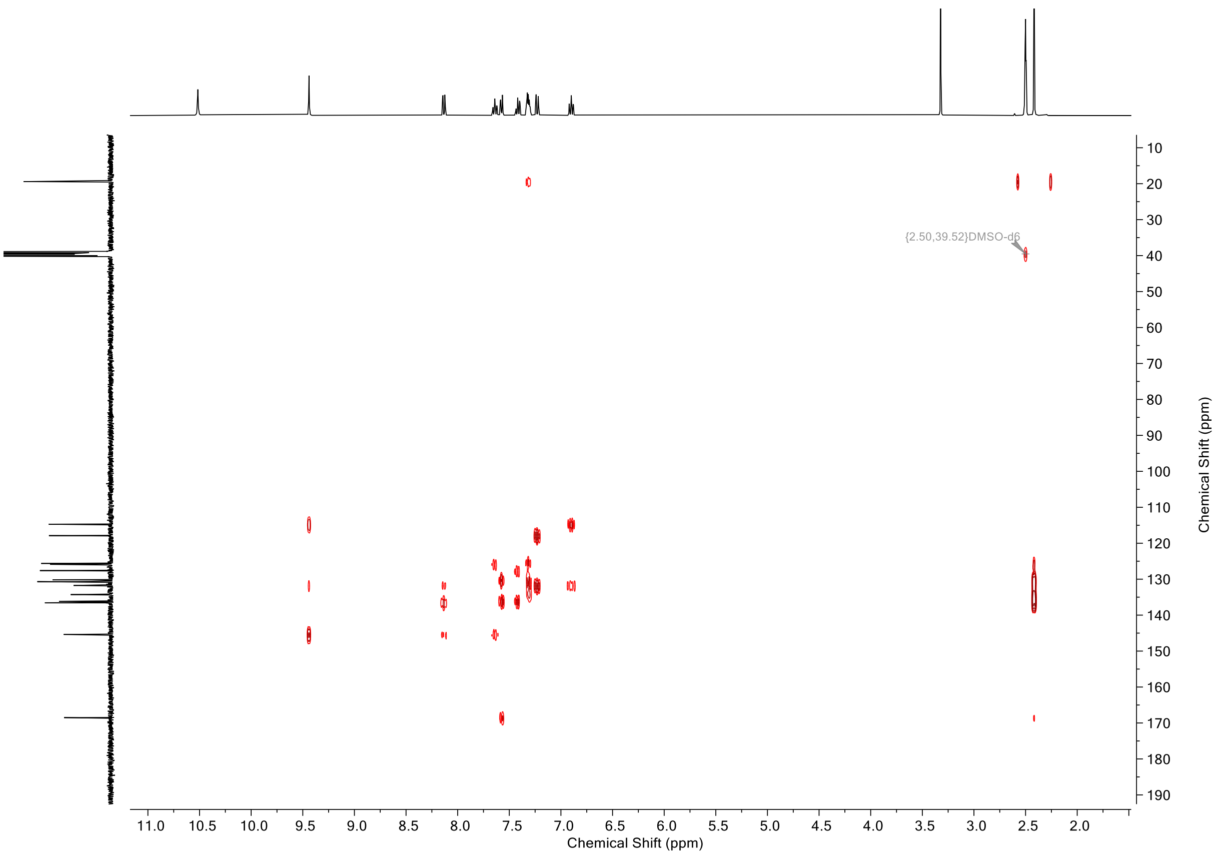

Figure S16: HMBC (DMSO-d_6): 2-Methyl-*N*'-(2-nitrophenyl)benzohydrazide (**1b**).

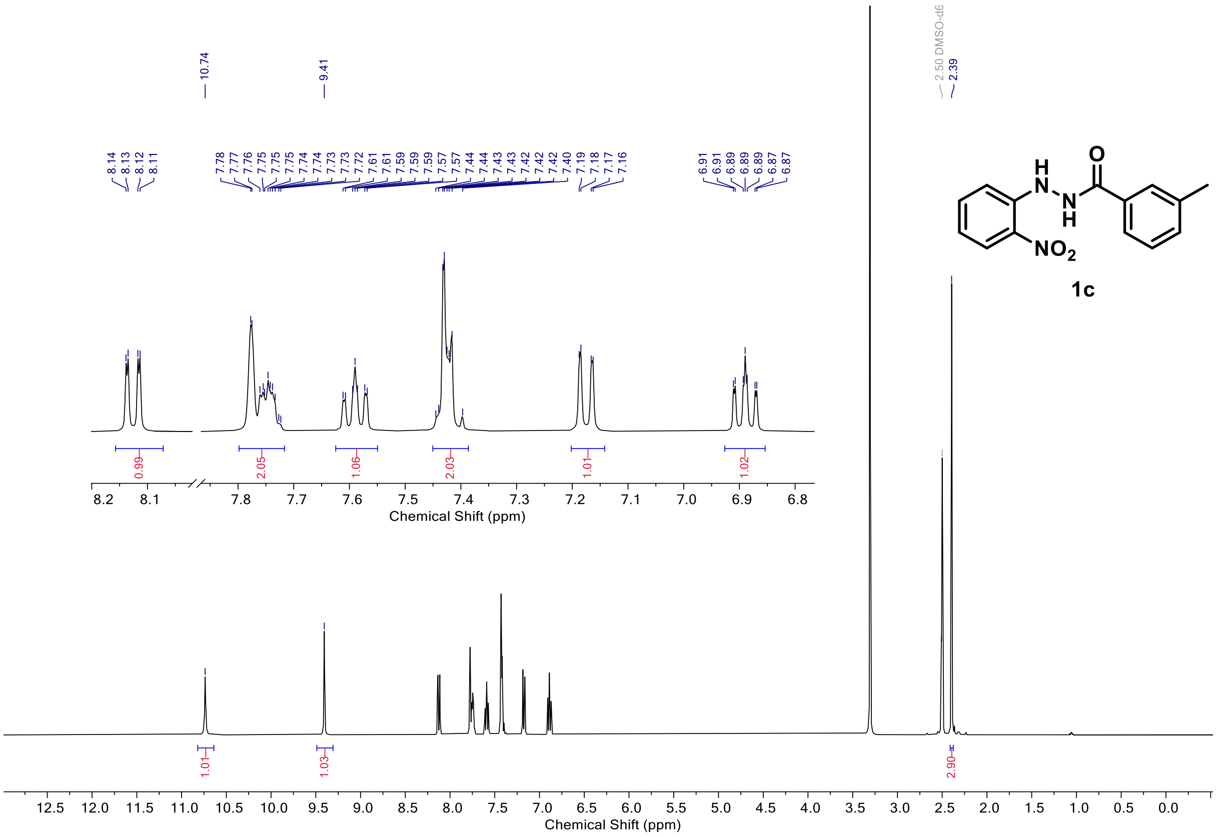

Figure S17: ^{1}H NMR (400 MHz, DMSO-d_6): 3-Methyl-*N*'-(2-nitrophenyl)benzohydrazide (**1c**).

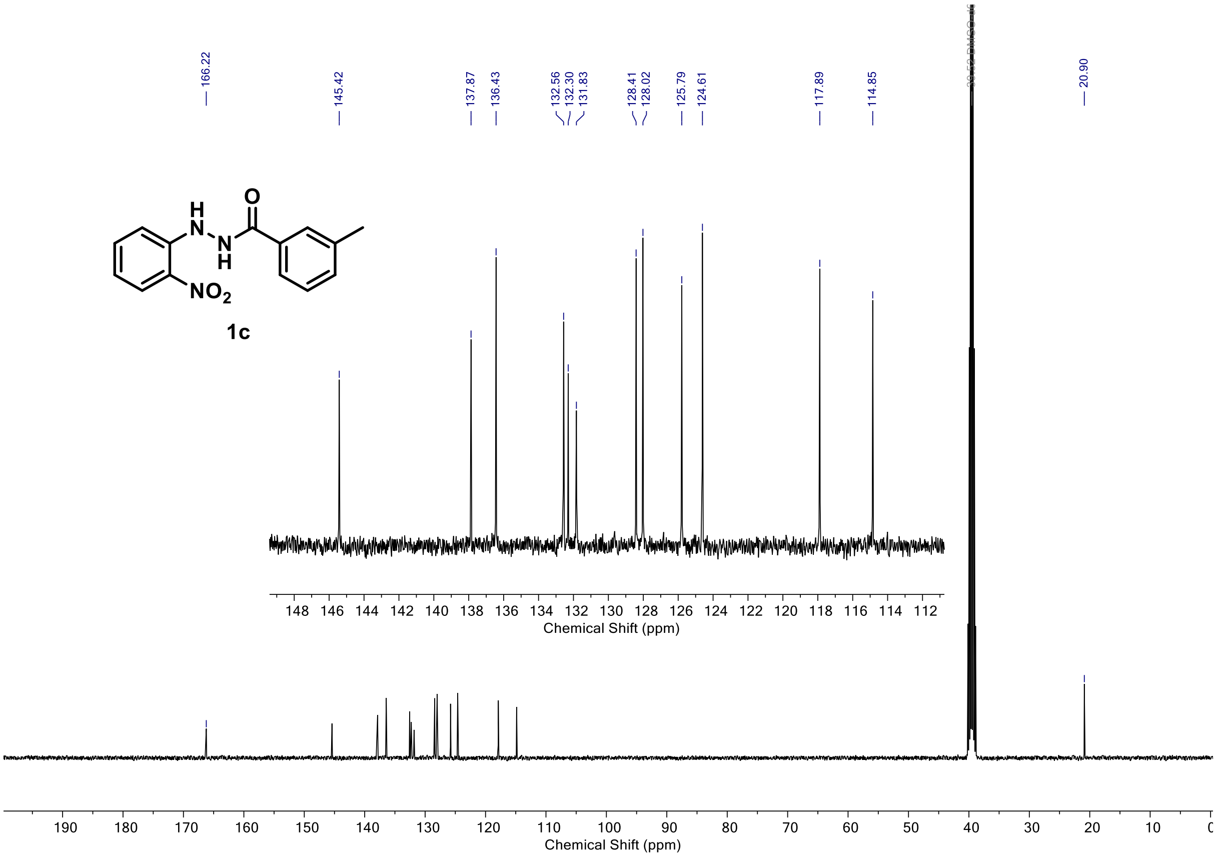

Figure S18: $^{13}C\{^{1}H\}$ NMR (101 MHz, DMSO-d_6): 3-Methyl-*N*'-(2-nitrophenyl)benzohydrazide (**1c**).

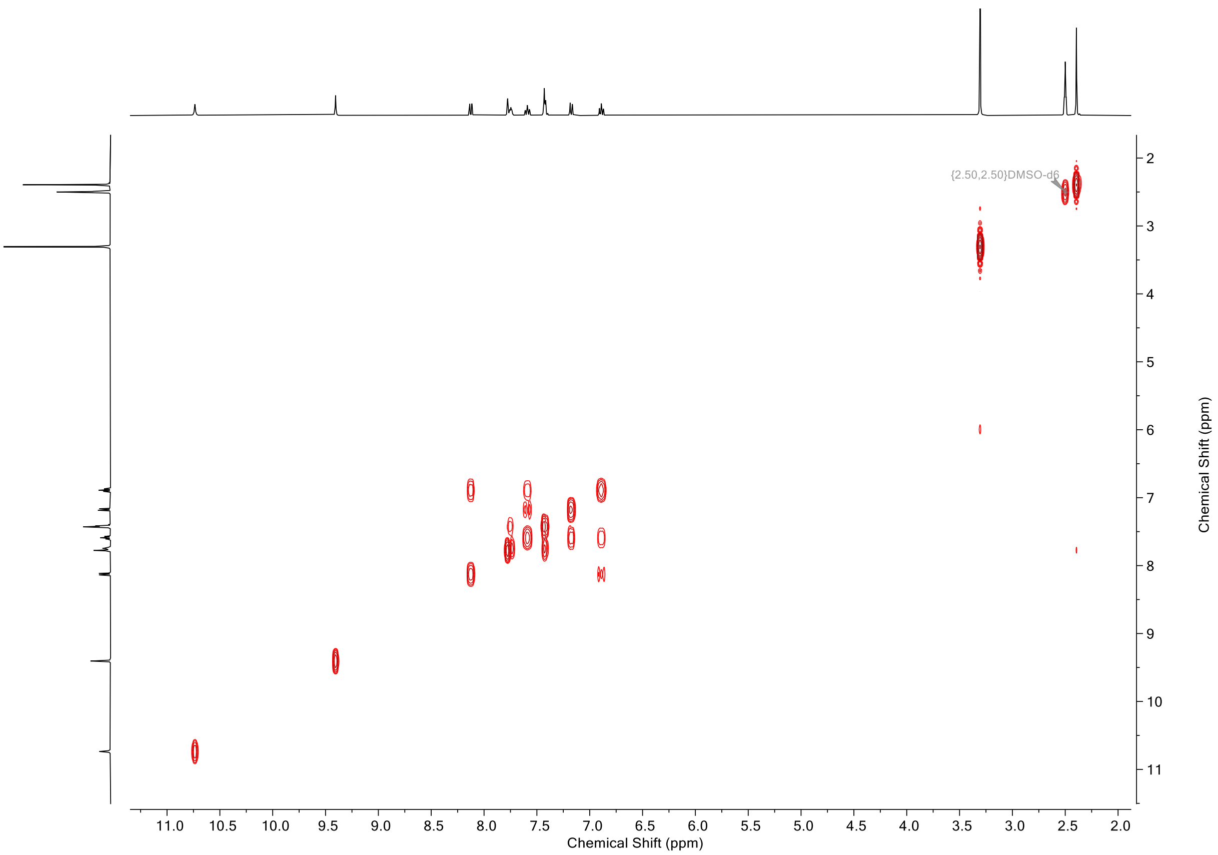

Figure S19: COSY (DMSO-d_6): 3-Methyl-*N*'-(2-nitrophenyl)benzohydrazide (**1c**).

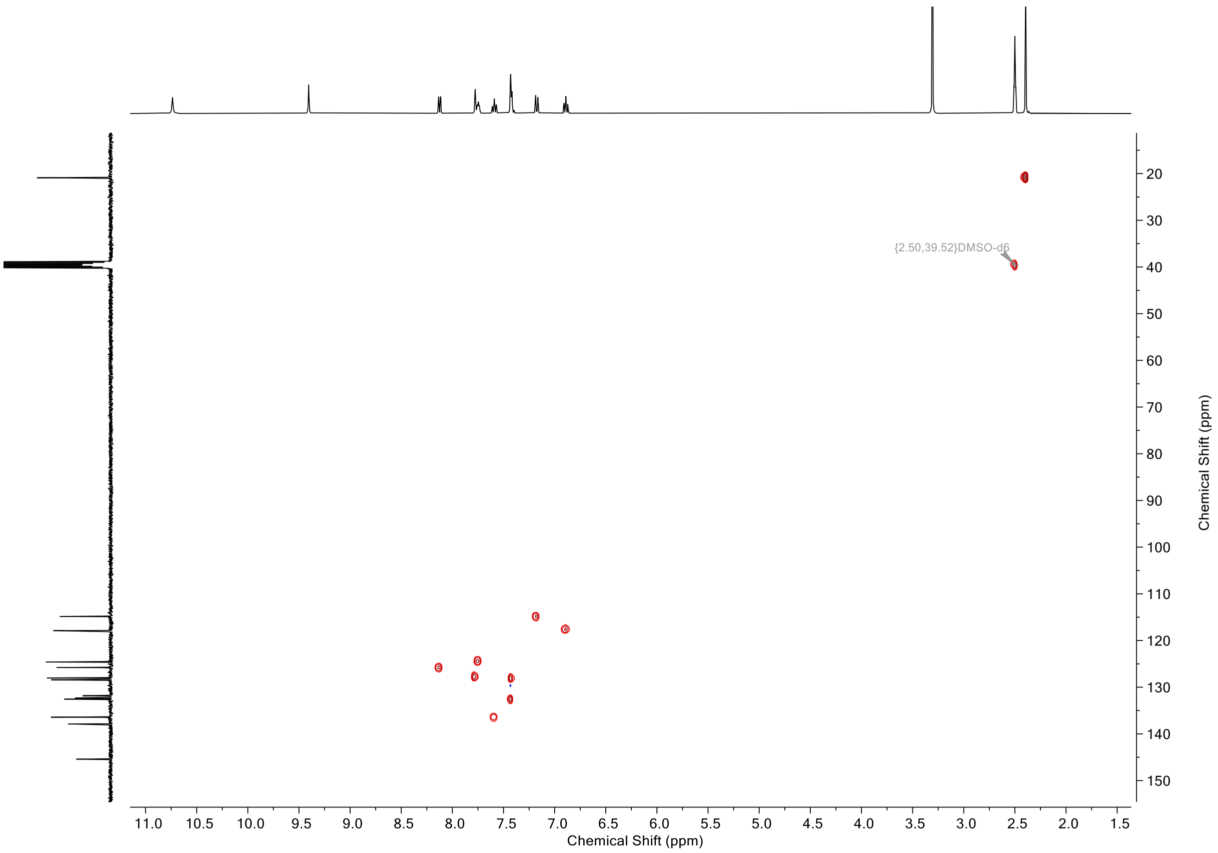

Figure S20: HSQC (DMSO-d_6): 3-Methyl-*N*'-(2-nitrophenyl)benzohydrazide (**1c**).

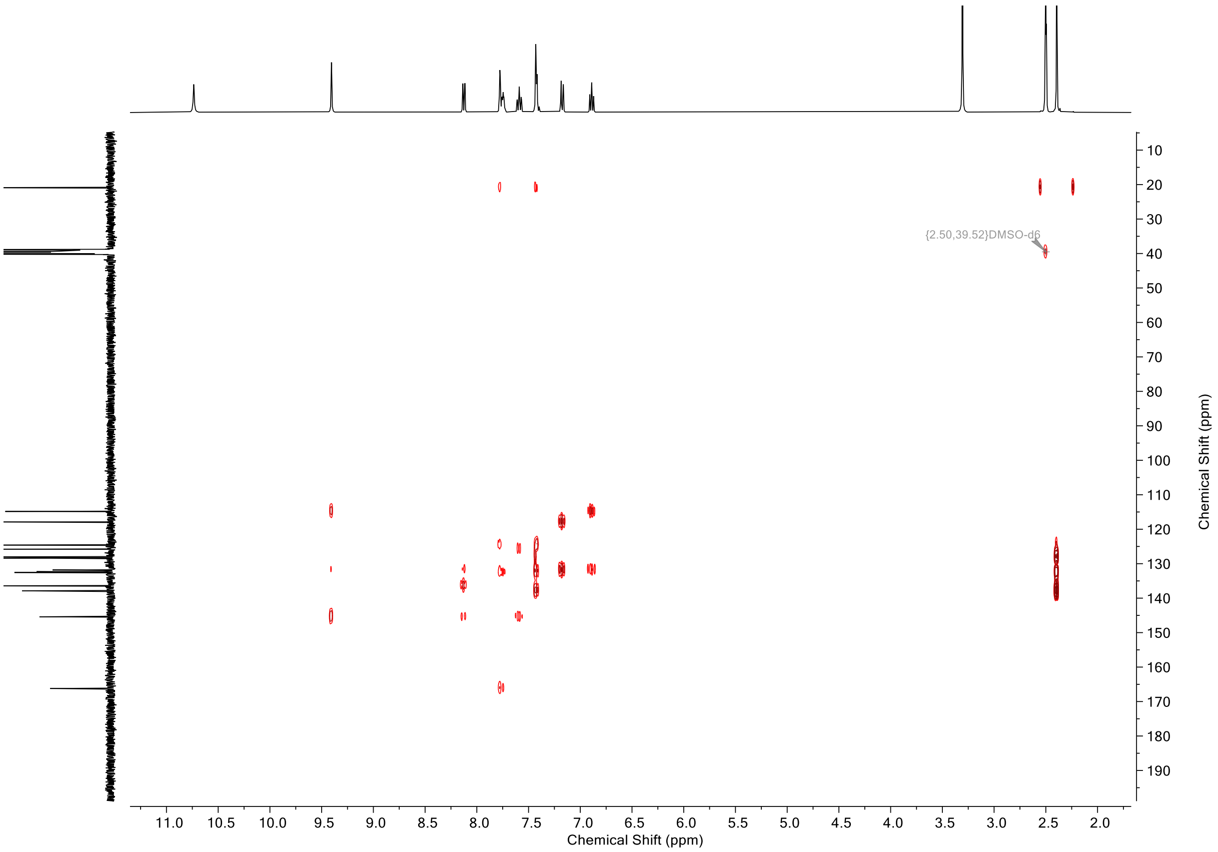

Figure S21: HMBC (DMSO-d_6): 3-Methyl-*N*'-(2-nitrophenyl)benzohydrazide (**1c**).

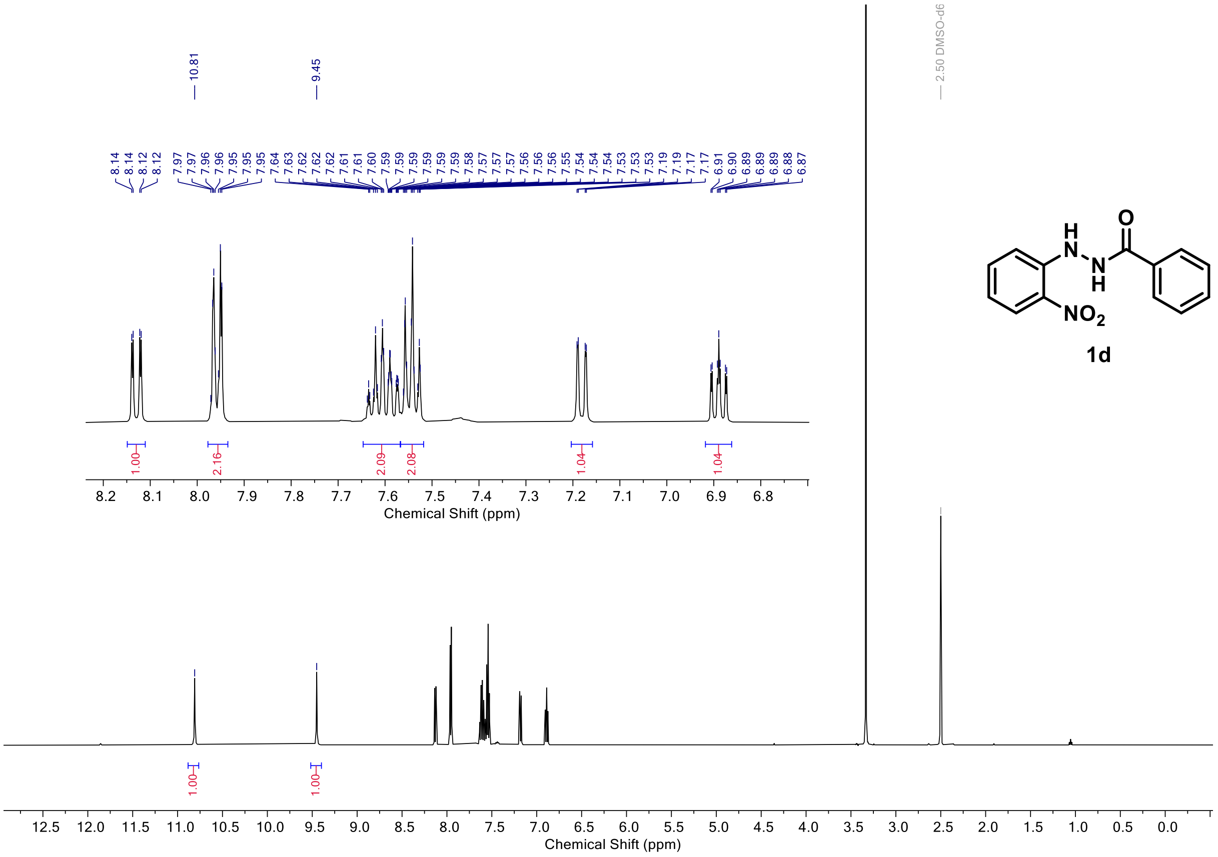

Figure S22: ^{1}H NMR (500 MHz, DMSO-d_6): *N*'-(2-Nitrophenyl)benzohydrazide (**1d**).

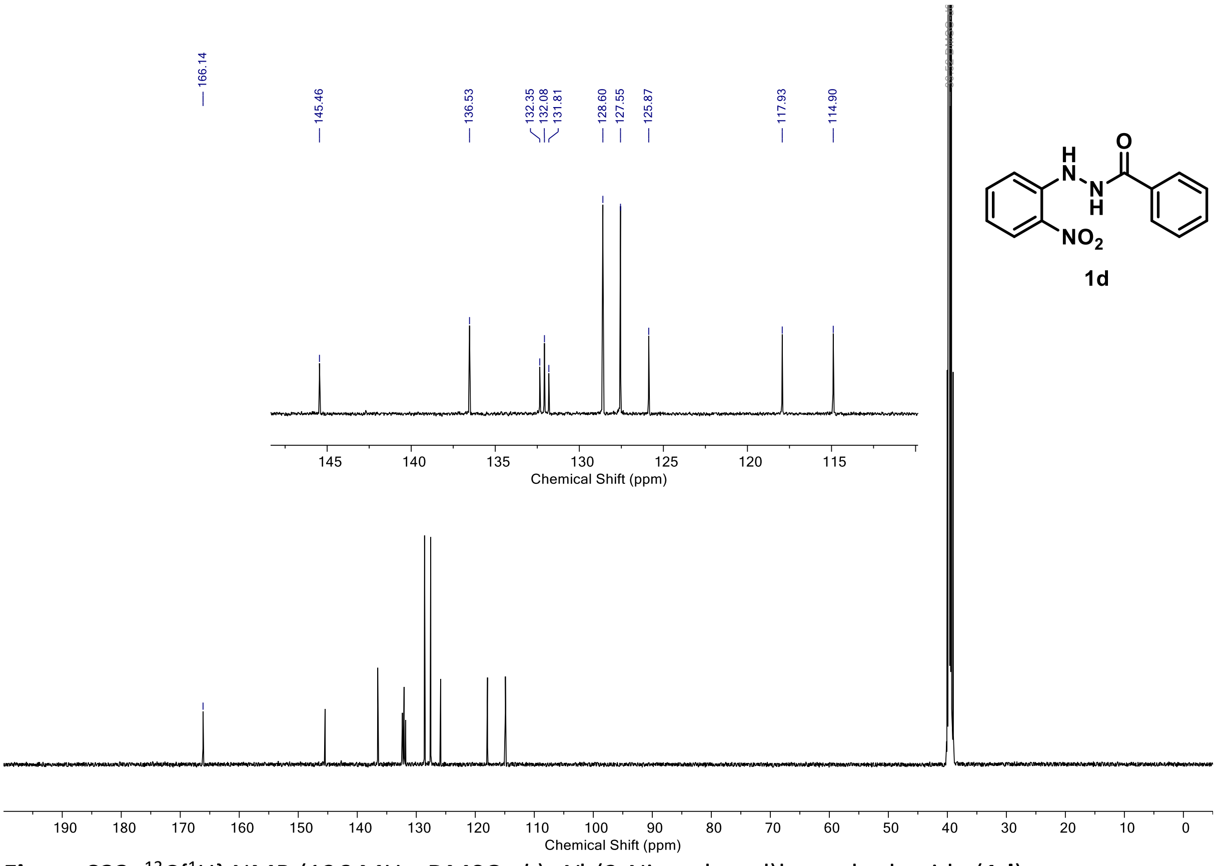

Figure S23: ^{13}C{^{1}H} NMR (126 MHz, DMSO-d_6): *N*'-(2-Nitrophenyl)benzohydrazide (**1d**).

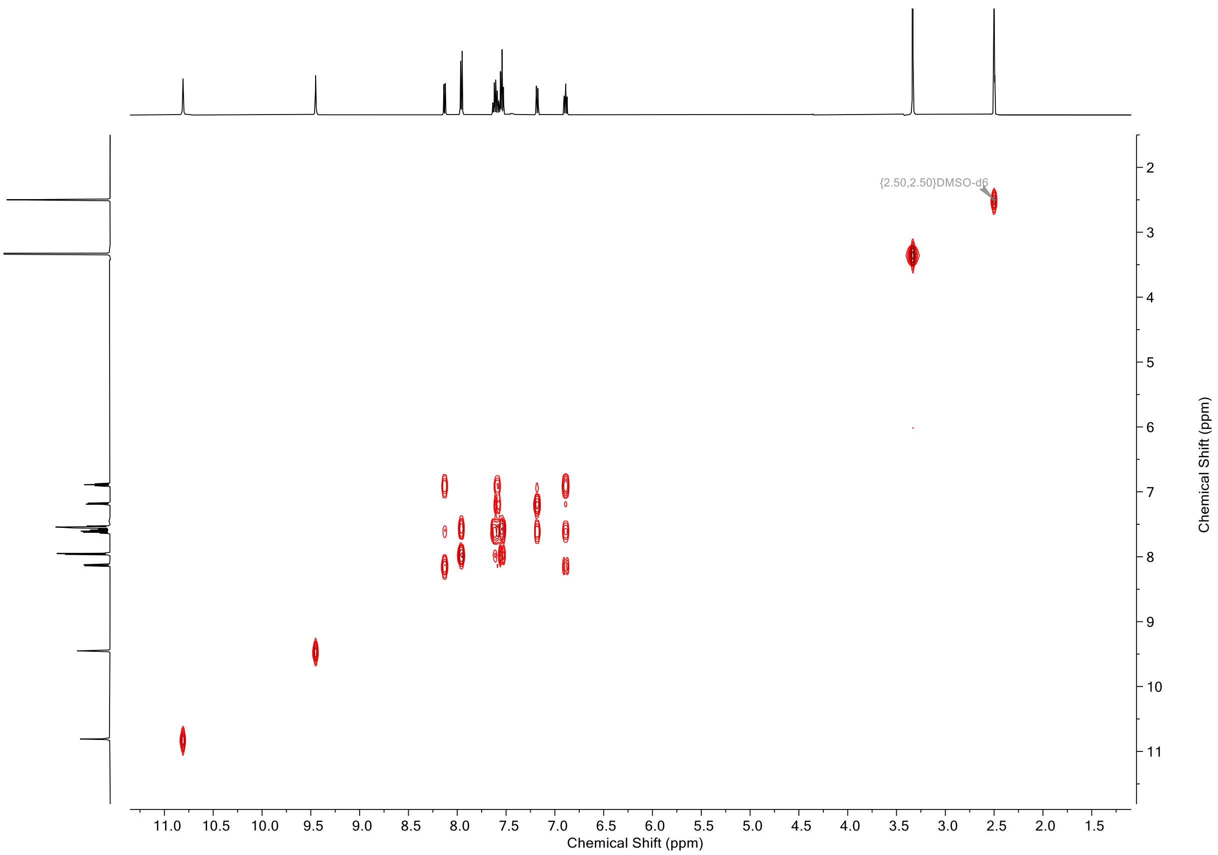

Figure S24: COSY (DMSO-d_6): *N*'-(2-Nitrophenyl)benzohydrazide (**1d**).

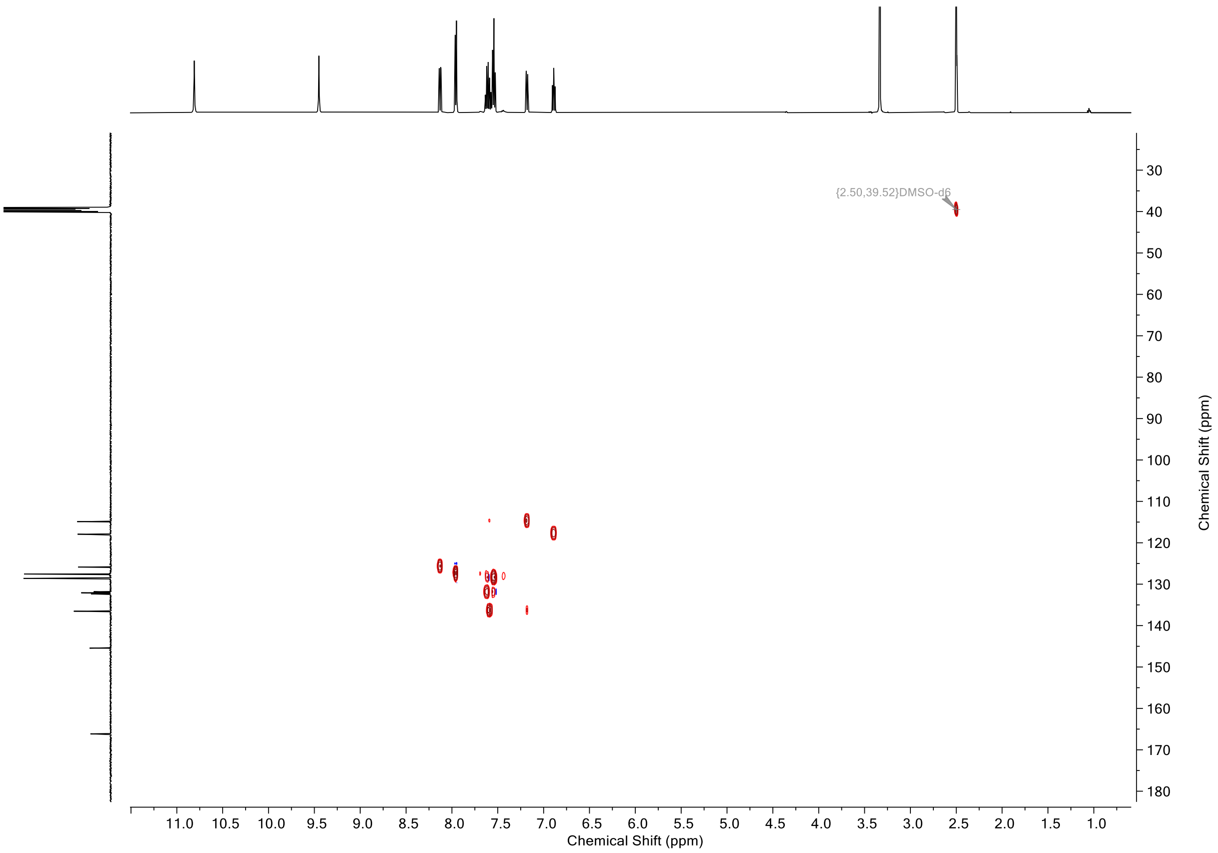

Figure S25: HSQC (DMSO-d_6): *N*'-(2-Nitrophenyl)benzohydrazide (**1d**).

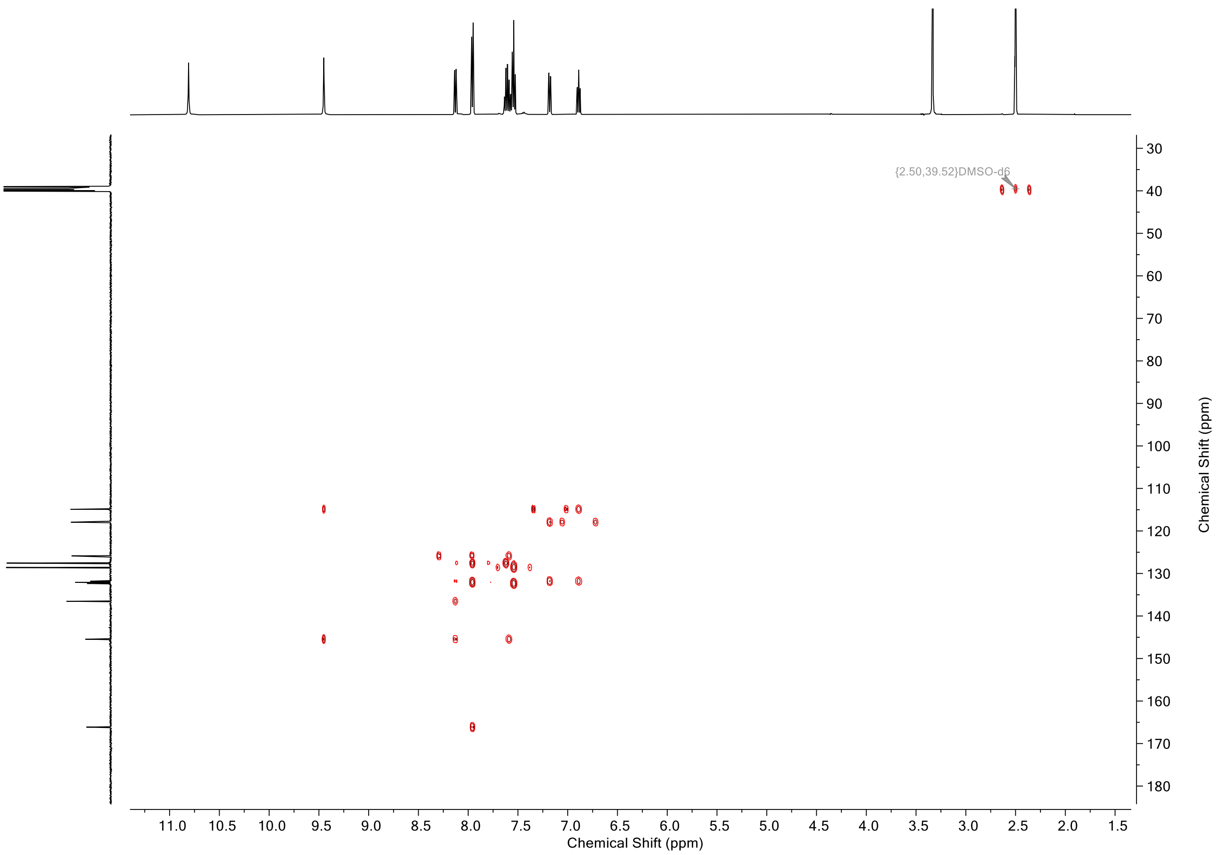

Figure S26: HMBC (DMSO-d_6): *N*'-(2-Nitrophenyl)benzohydrazide (**1d**).

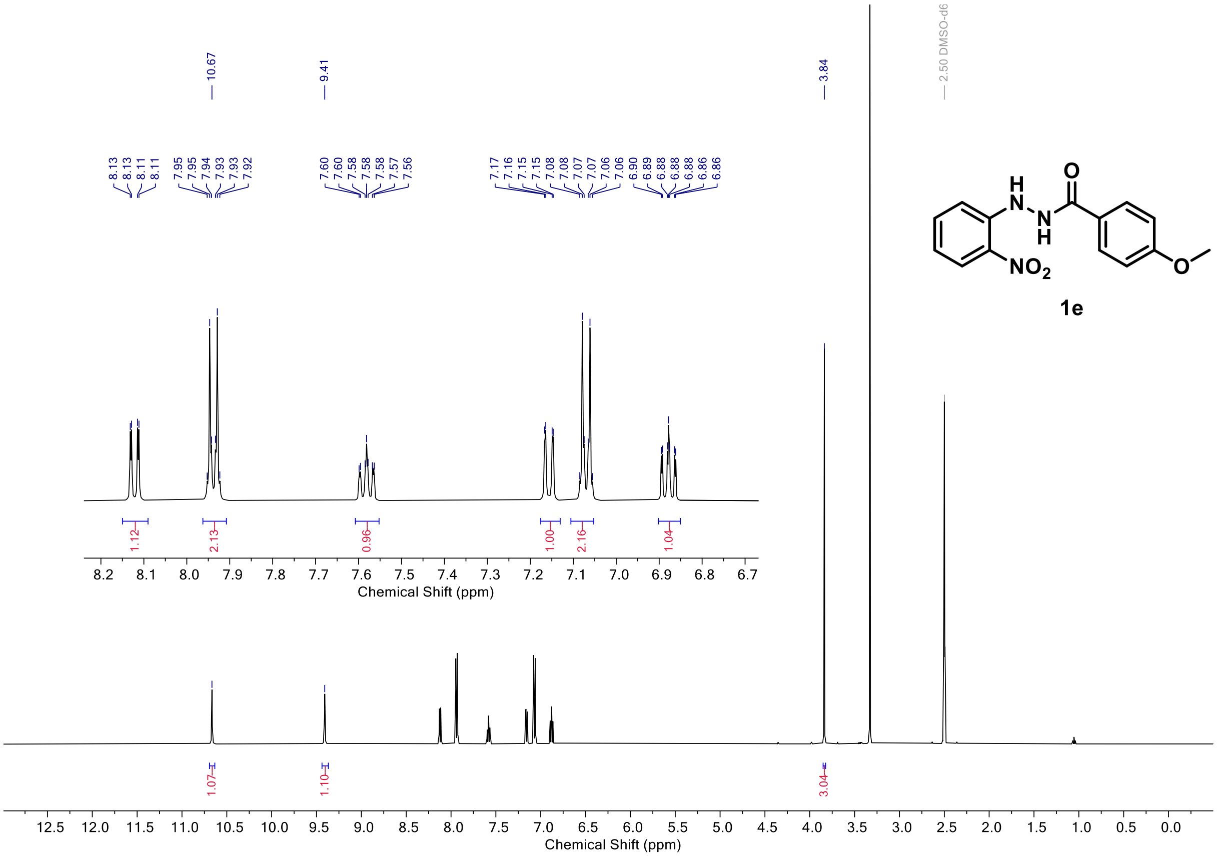

Figure S27: ^{1}H NMR (500 MHz, DMSO-d_6): 4-Methoxy-*N*'-(2-nitrophenyl)benzohydrazide (**1e**).

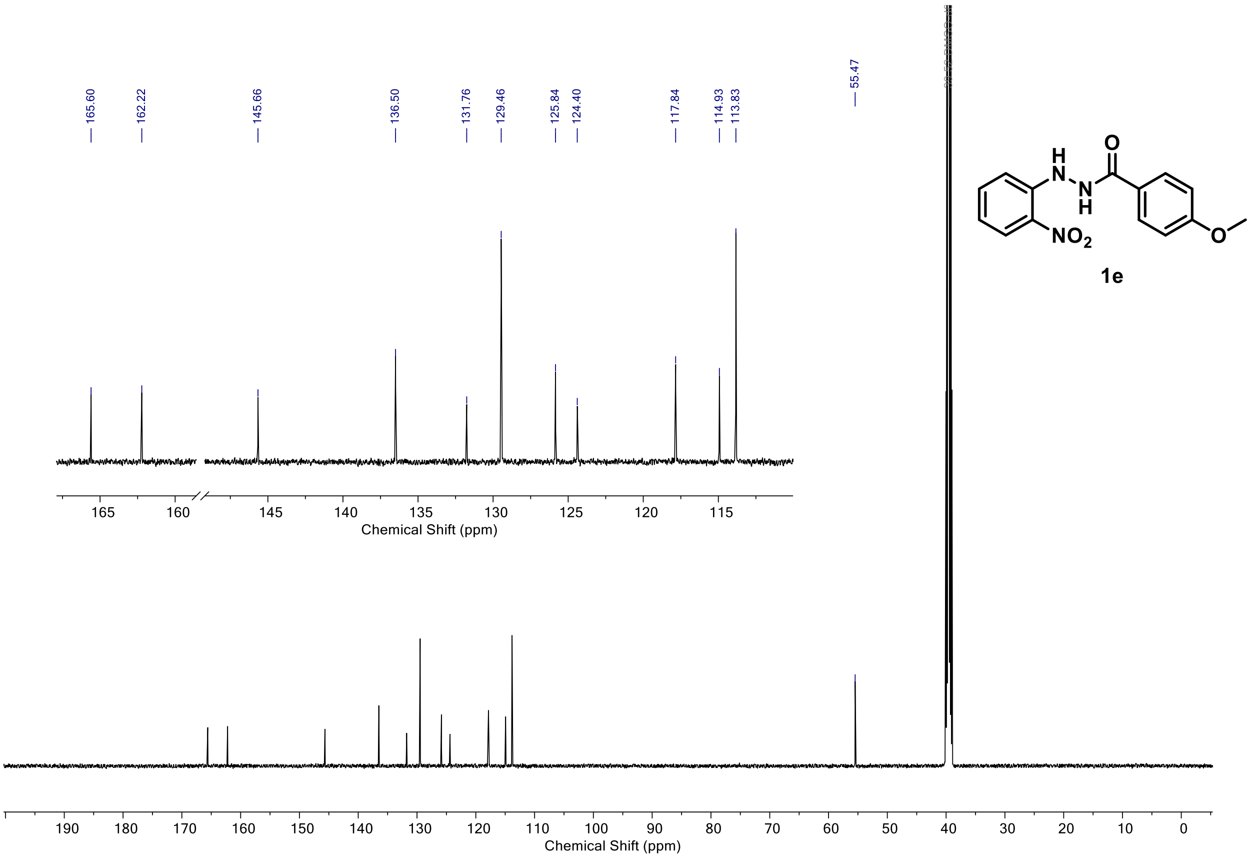

Figure S28: ^{13}C{^{1}H} NMR (126 MHz, DMSO-d_6): 4-Methoxy-*N*'-(2-nitrophenyl)benzohydrazide (**1e**).

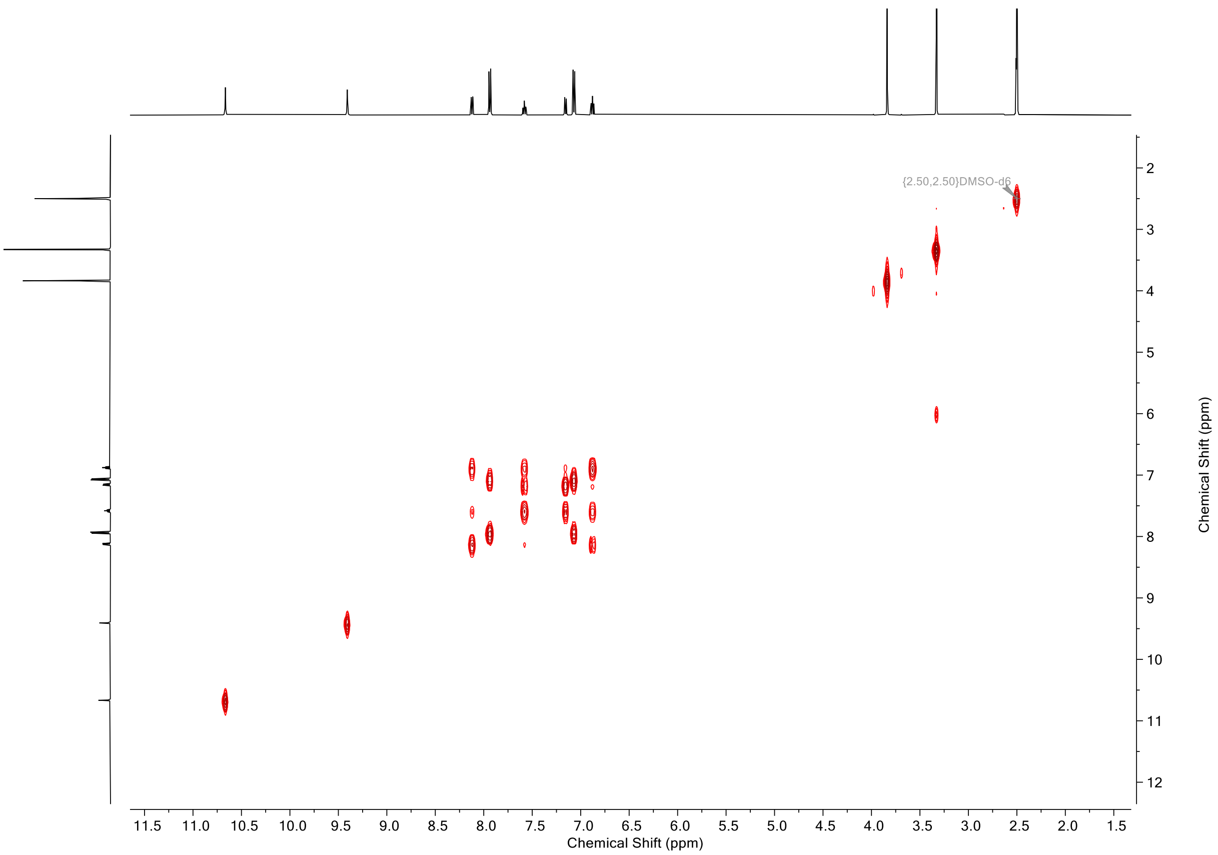

Figure S29: COSY (DMSO-d_6): 4-Methoxy-*N*'-(2-nitrophenyl)benzohydrazide (**1e**).

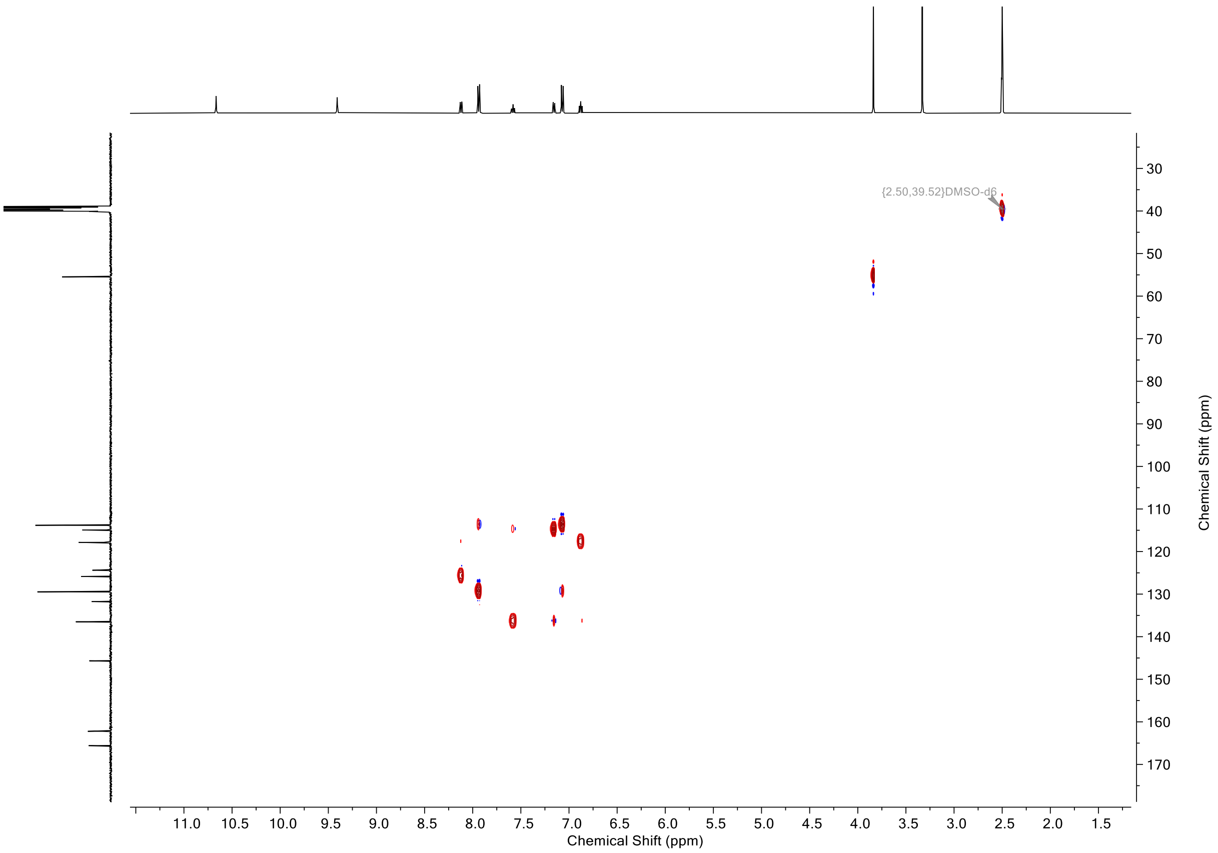

Figure S30: HSQC (DMSO-d_6): 4-Methoxy-*N*'-(2-nitrophenyl)benzohydrazide (**1e**).

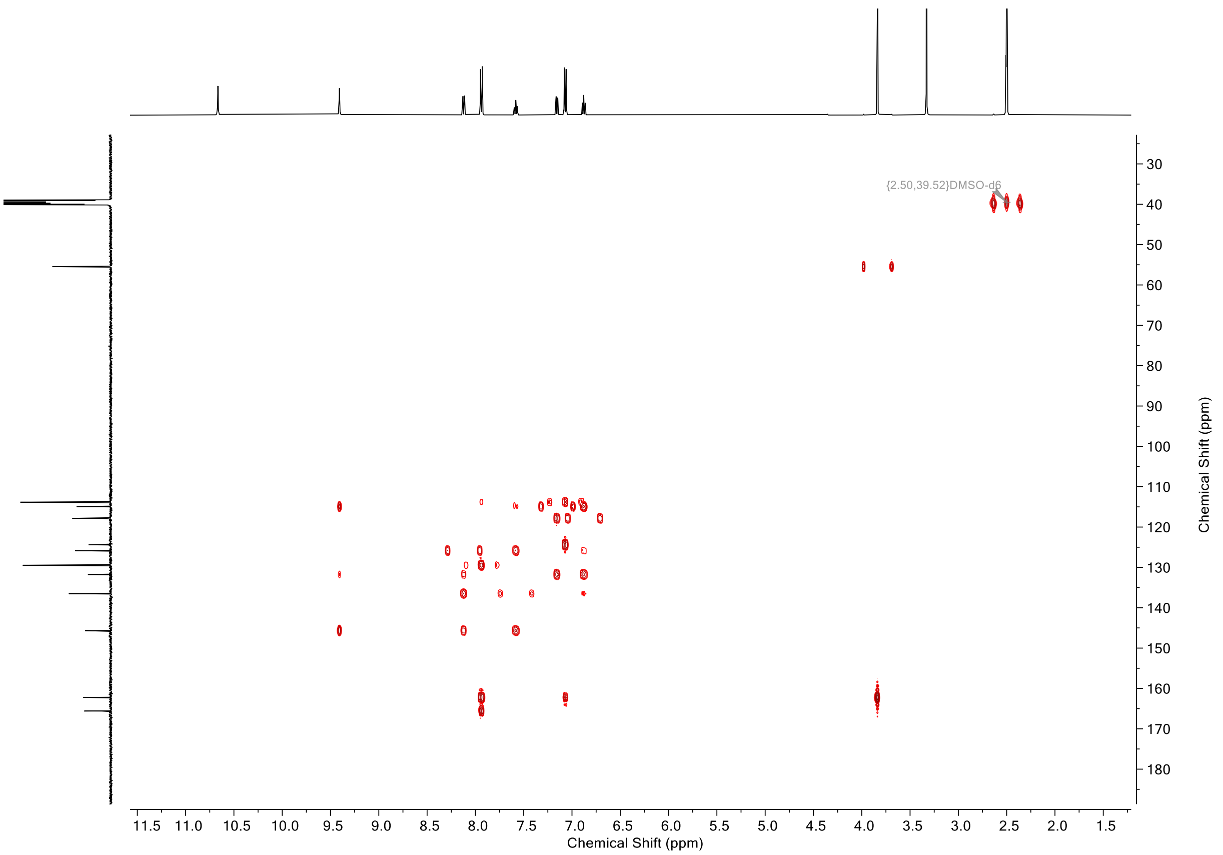

Figure S31: HMBC (DMSO-d_6): 4-Methoxy-*N*'-(2-nitrophenyl)benzohydrazide (**1e**).

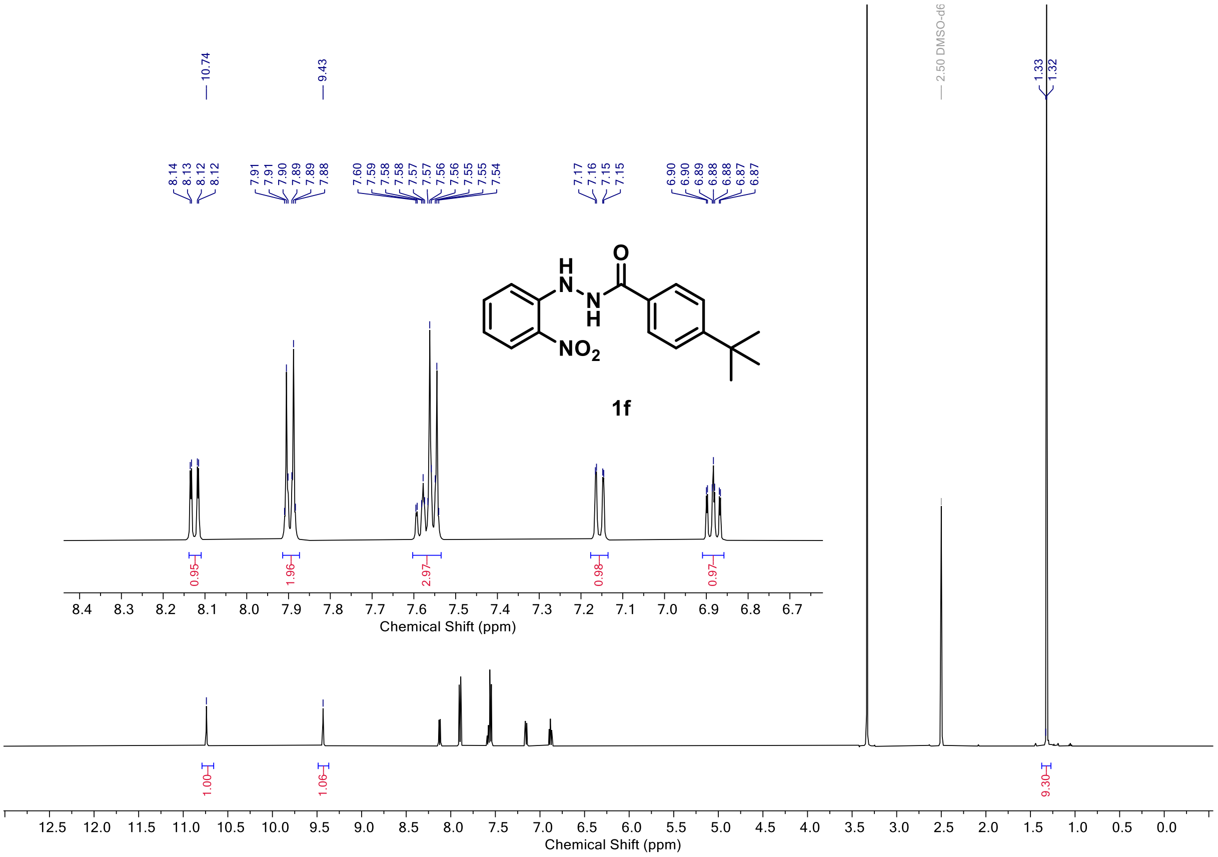

Figure S32: ^{1}H NMR (500 MHz, DMSO-d_6): 4-(*tert*-Butyl)-*N*'-(2-nitrophenyl)benzohydrazide (**1f**).

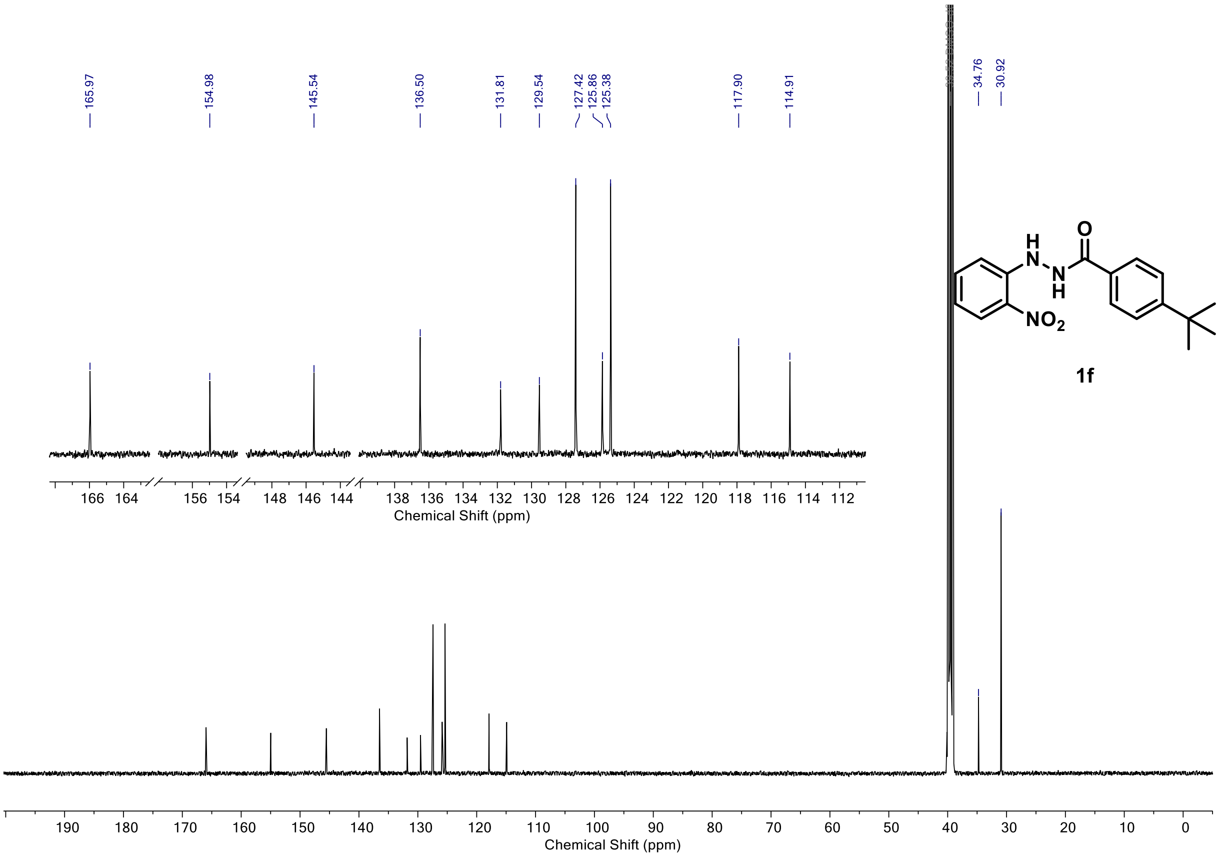

Figure S33: ^{13}C{^{1}H} NMR (126 MHz, DMSO-d_6): 4-(*tert*-Butyl)-*N*'-(2-nitrophenyl)benzohydrazide (**1f**).

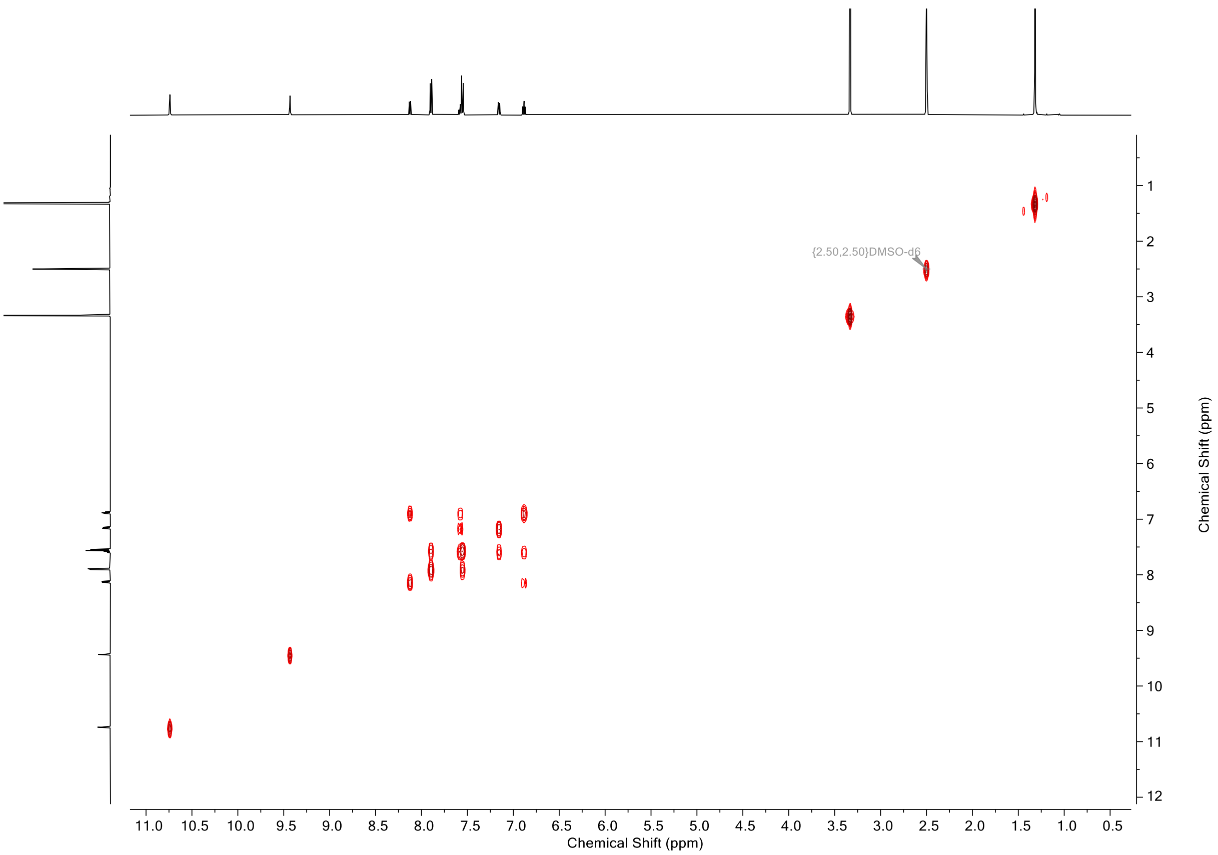

Figure S34: COSY (DMSO-d_6): 4-(*tert*-Butyl)-*N*'-(2-nitrophenyl)benzohydrazide (**1f**).

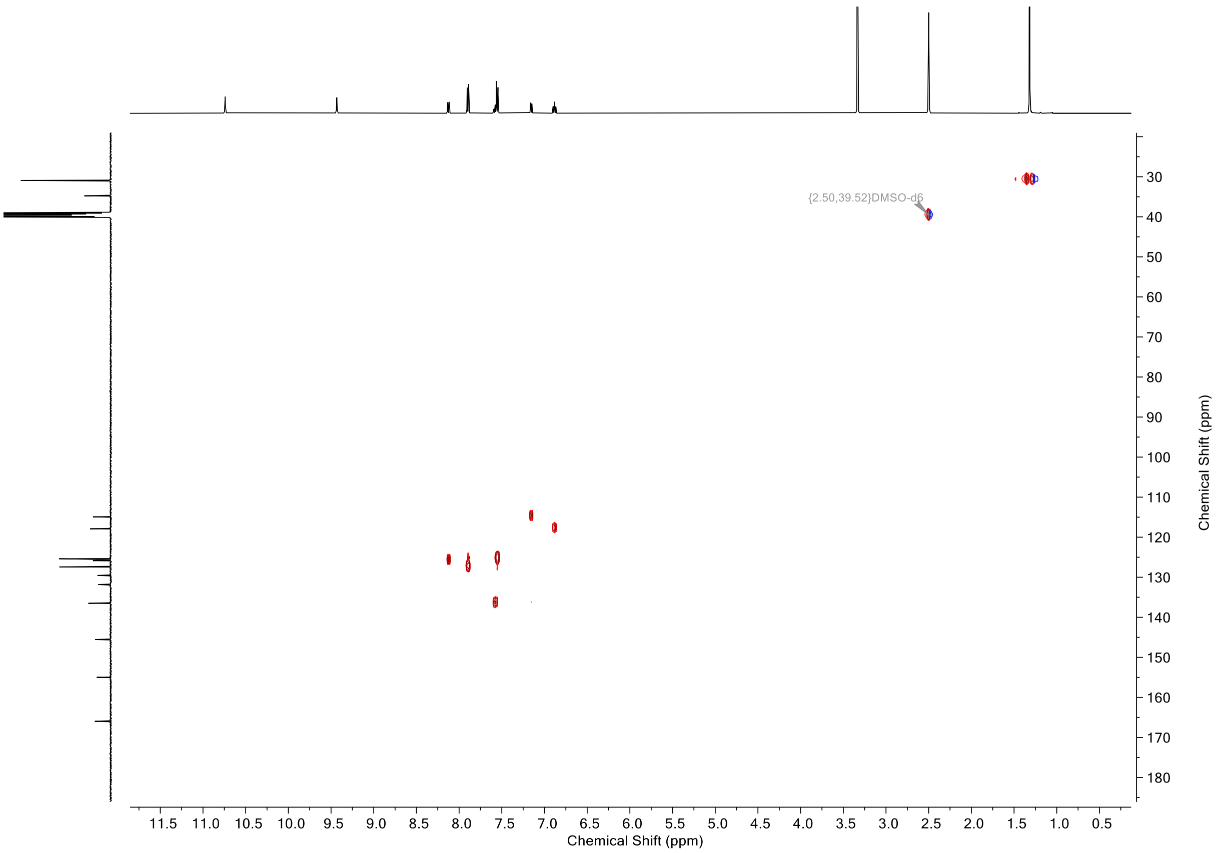

Figure S35: HSQC (DMSO-d_6): 4-(*tert*-Butyl)-*N*'-(2-nitrophenyl)benzohydrazide (**1f**).

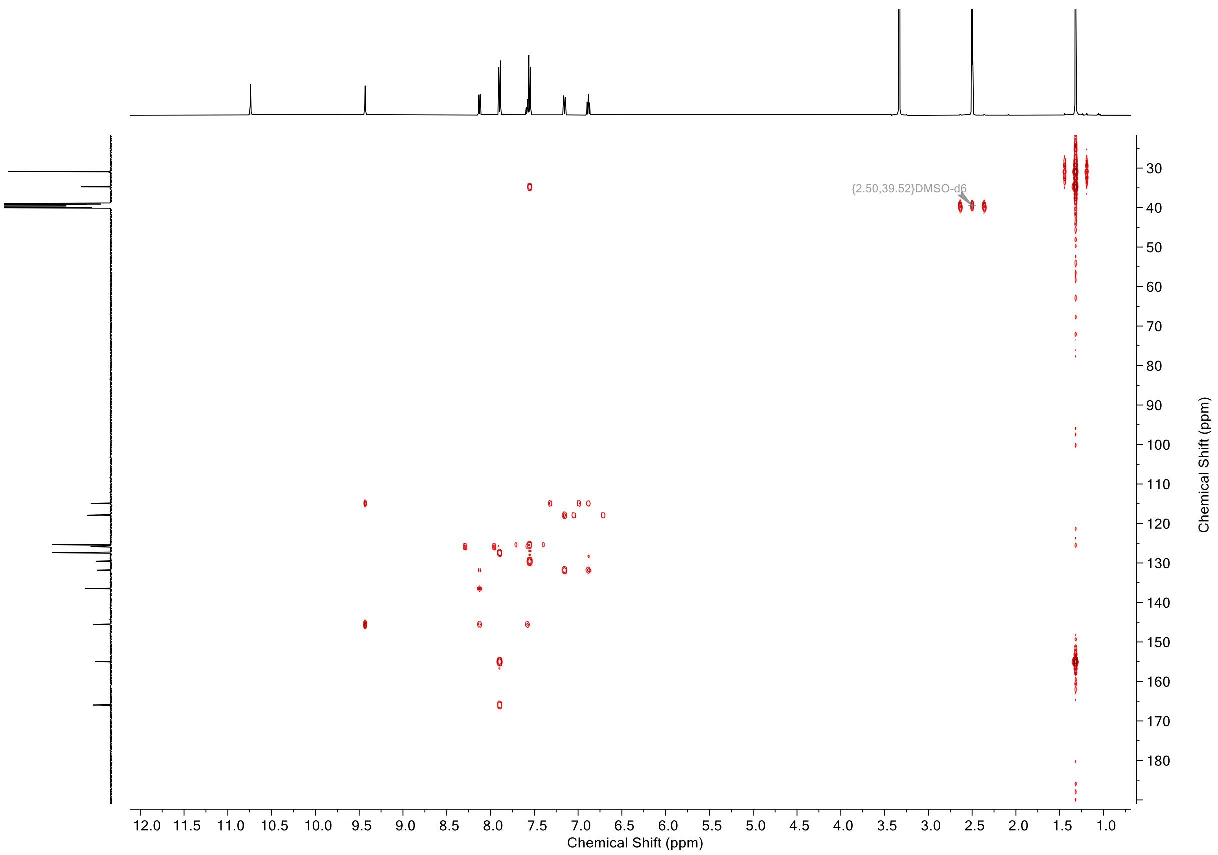

Figure S36: HMBC (DMSO-d_6): 4-(*tert*-Butyl)-*N*'-(2-nitrophenyl)benzohydrazide (**1f**).

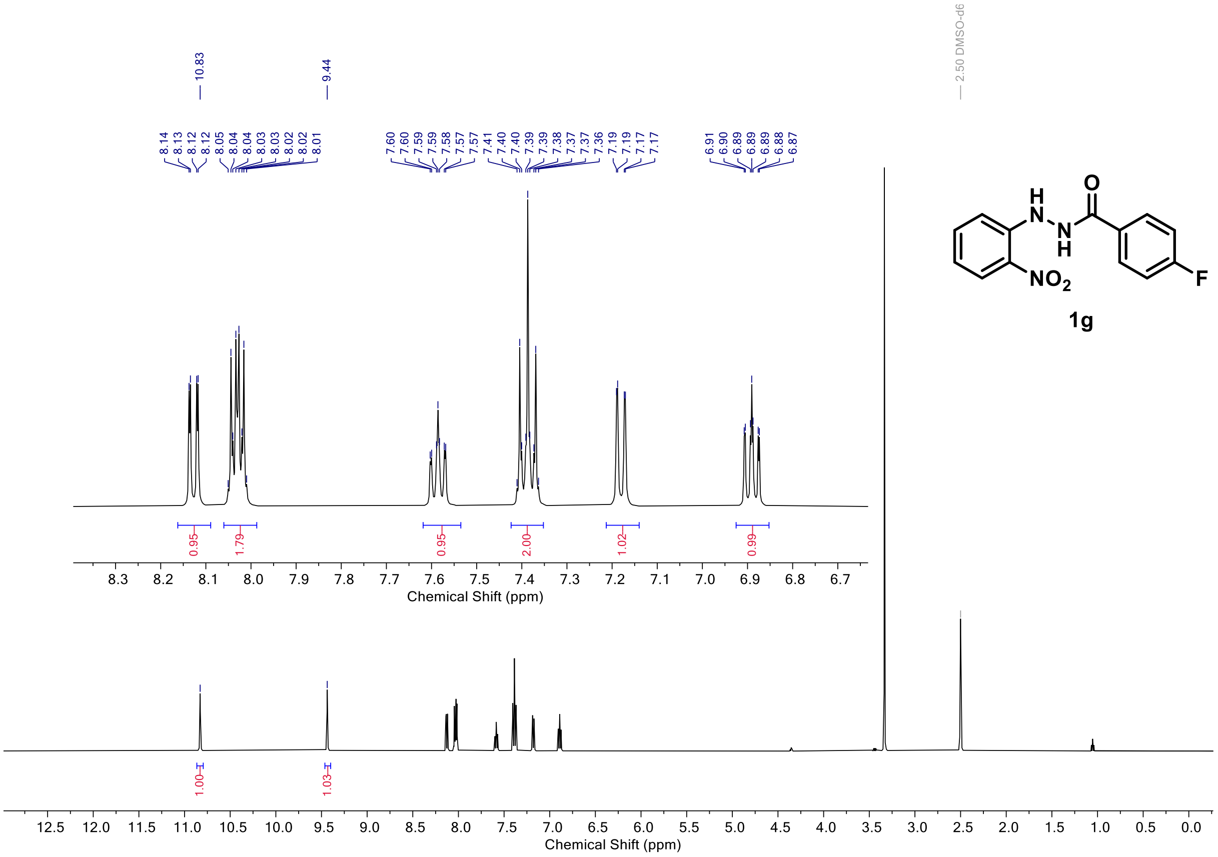

Figure S37: ^{1}H NMR (500 MHz, DMSO-d_6): 4-Fluoro-*N*'-(2-nitrophenyl)benzohydrazide (**1g**).

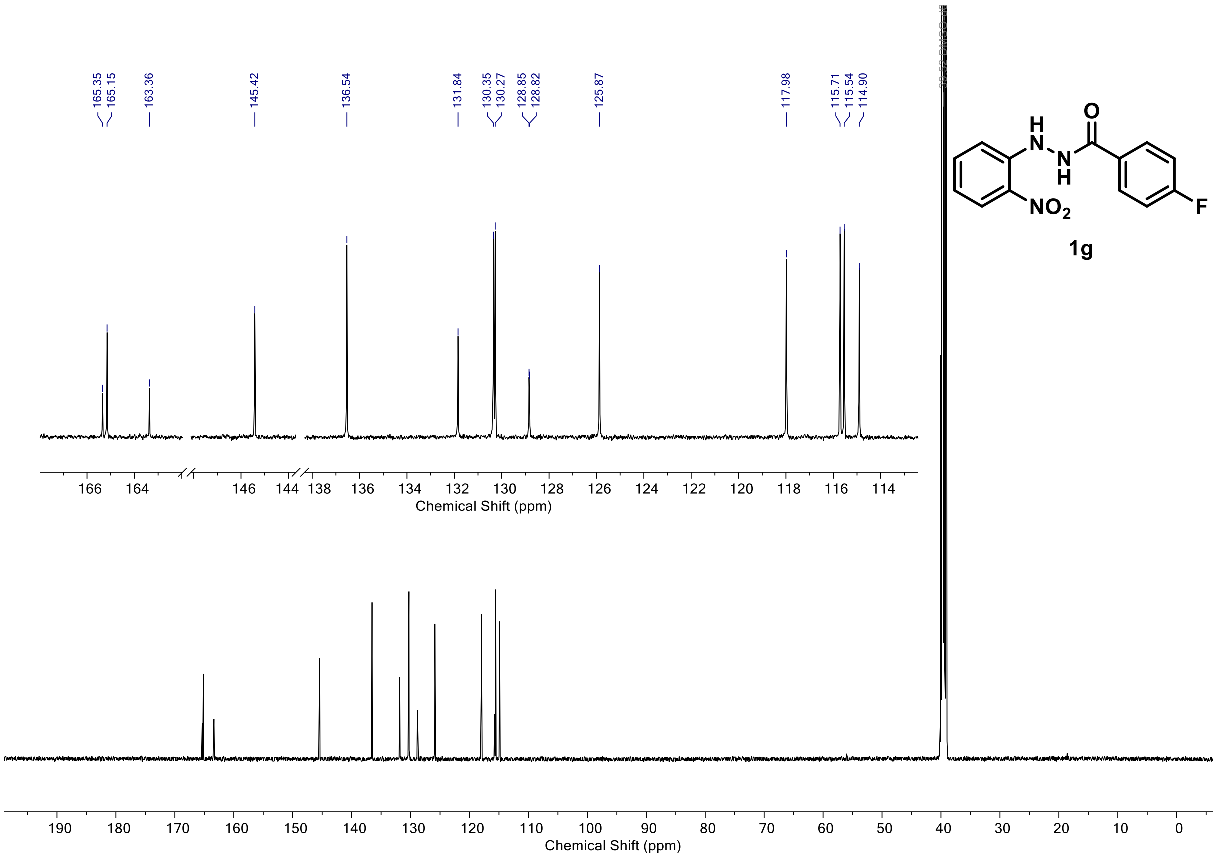

Figure S38: ^{13}C{^{1}H} NMR (126 MHz, DMSO-d_6): 4-Fluoro-*N*'-(2-nitrophenyl)benzohydrazide (**1g**).

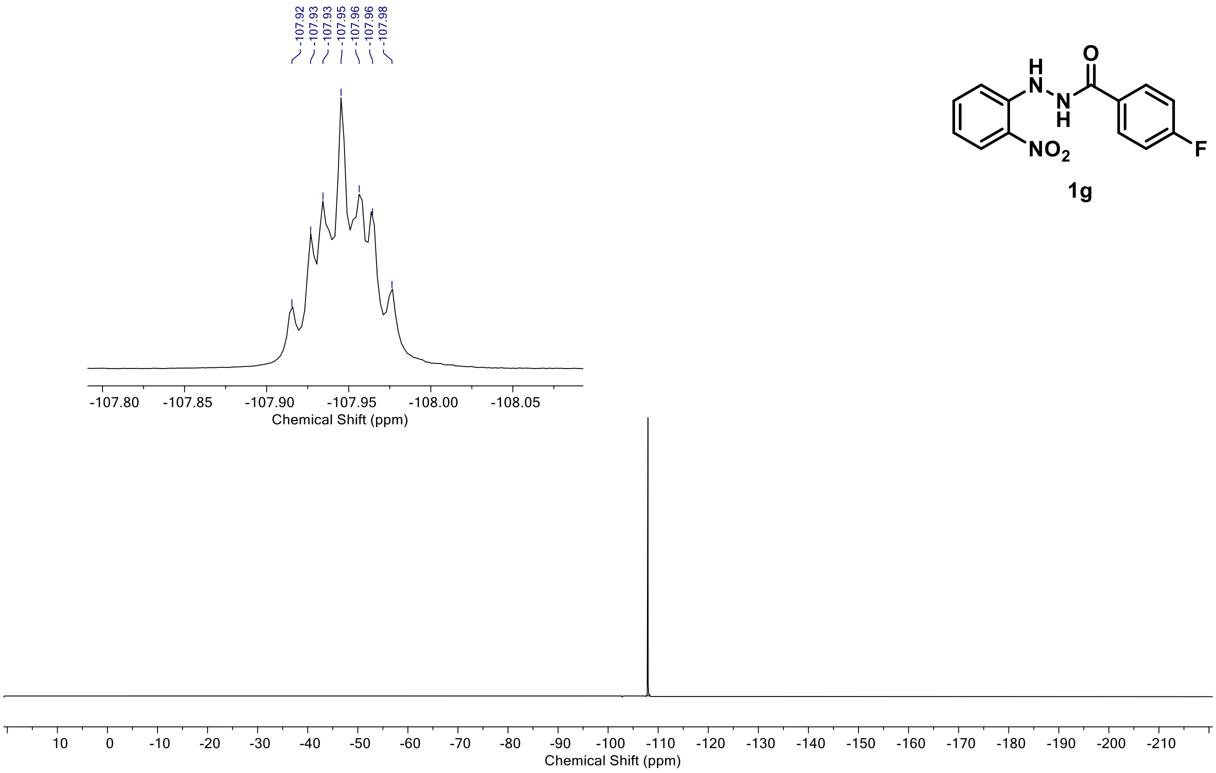

1g

Figure S39: ^{19}F NMR (470 MHz, DMSO-d_6): 4-Fluoro-*N*'-(2-nitrophenyl)benzohydrazide (**1g**).

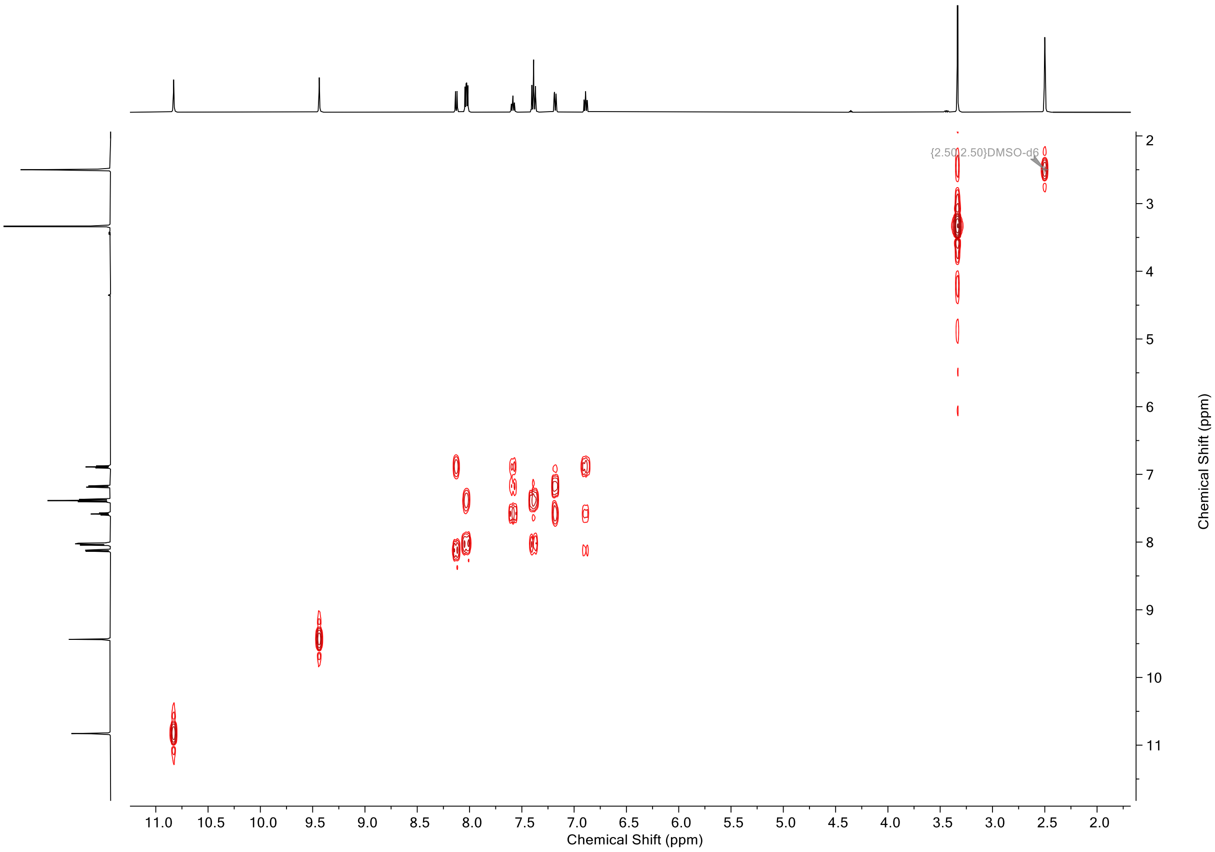

Figure S40: COSY (DMSO-d_6): 4-Fluoro-*N*'-(2-nitrophenyl)benzohydrazide (**1g**).

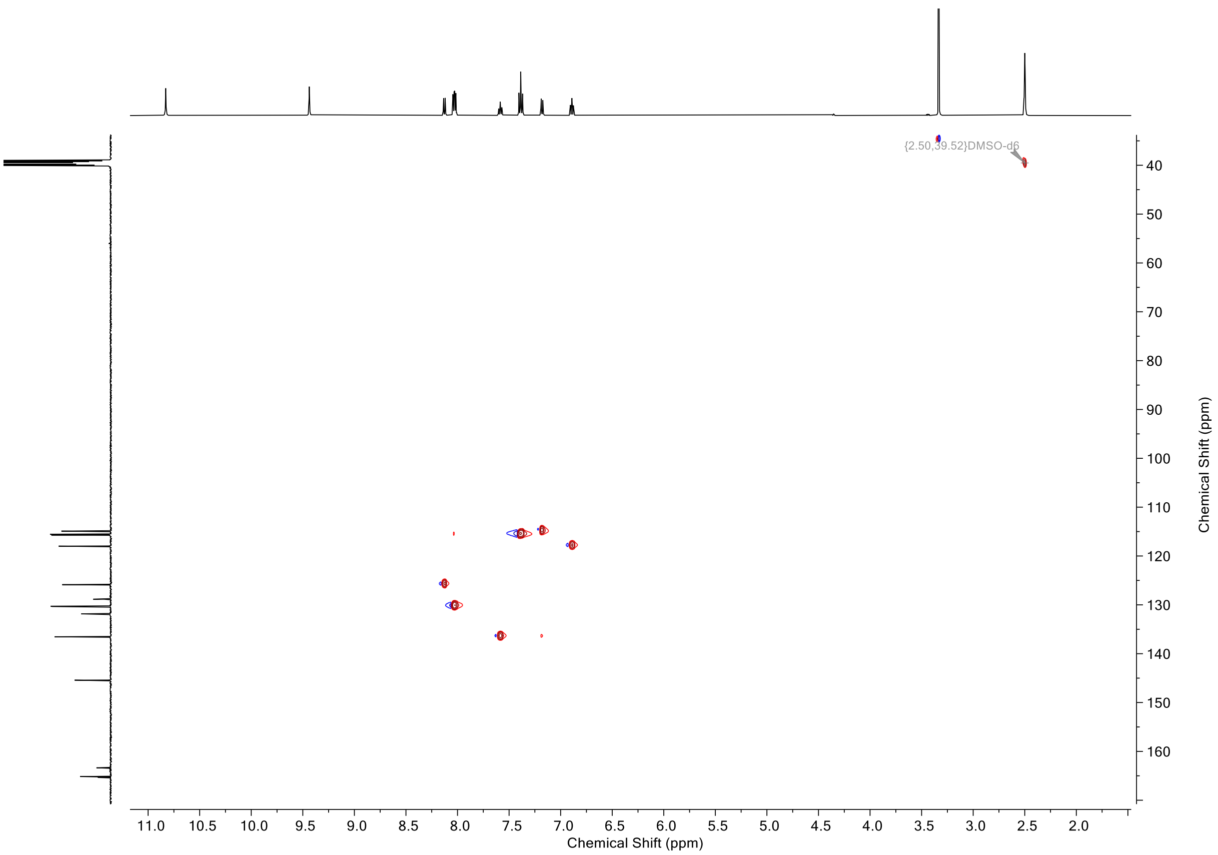

Figure S41: HSQC (DMSO-d_6): 4-Fluoro-*N*'-(2-nitrophenyl)benzohydrazide (**1g**).

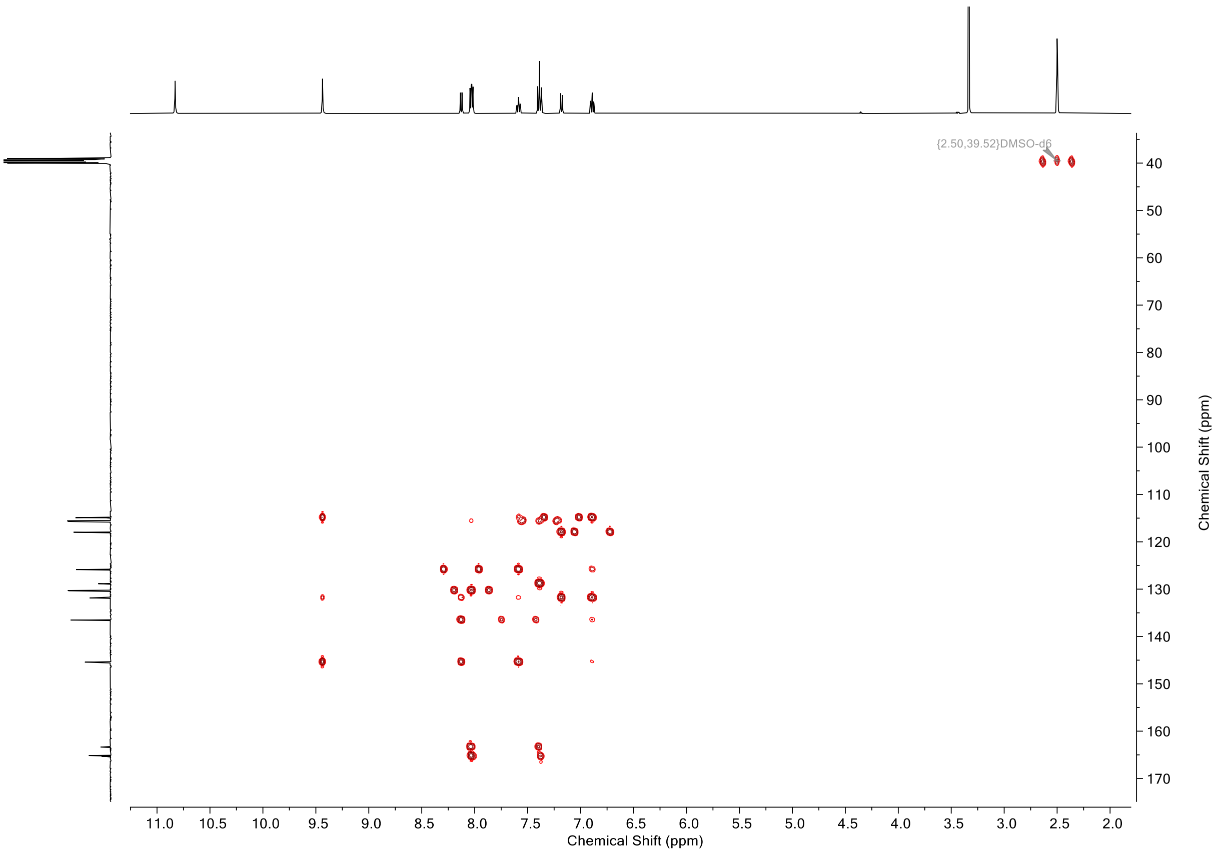

Figure S42: HMBC (DMSO-d_6): 4-Fluoro-*N*'-(2-nitrophenyl)benzohydrazide (**1g**).

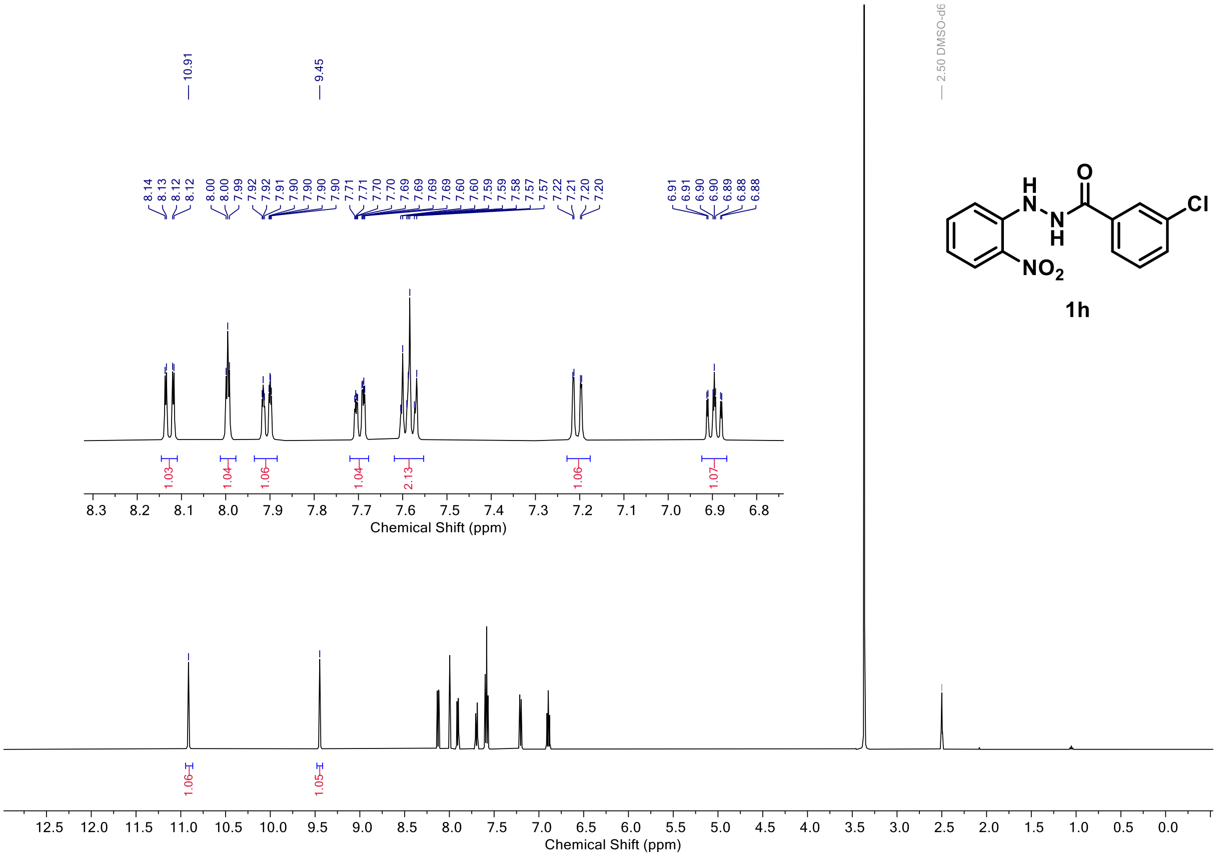

Figure S43: ^{1}H NMR (500 MHz, DMSO-d_6): 3-Chloro-*N*'-(2-nitrophenyl)benzohydrazide (**1h**).

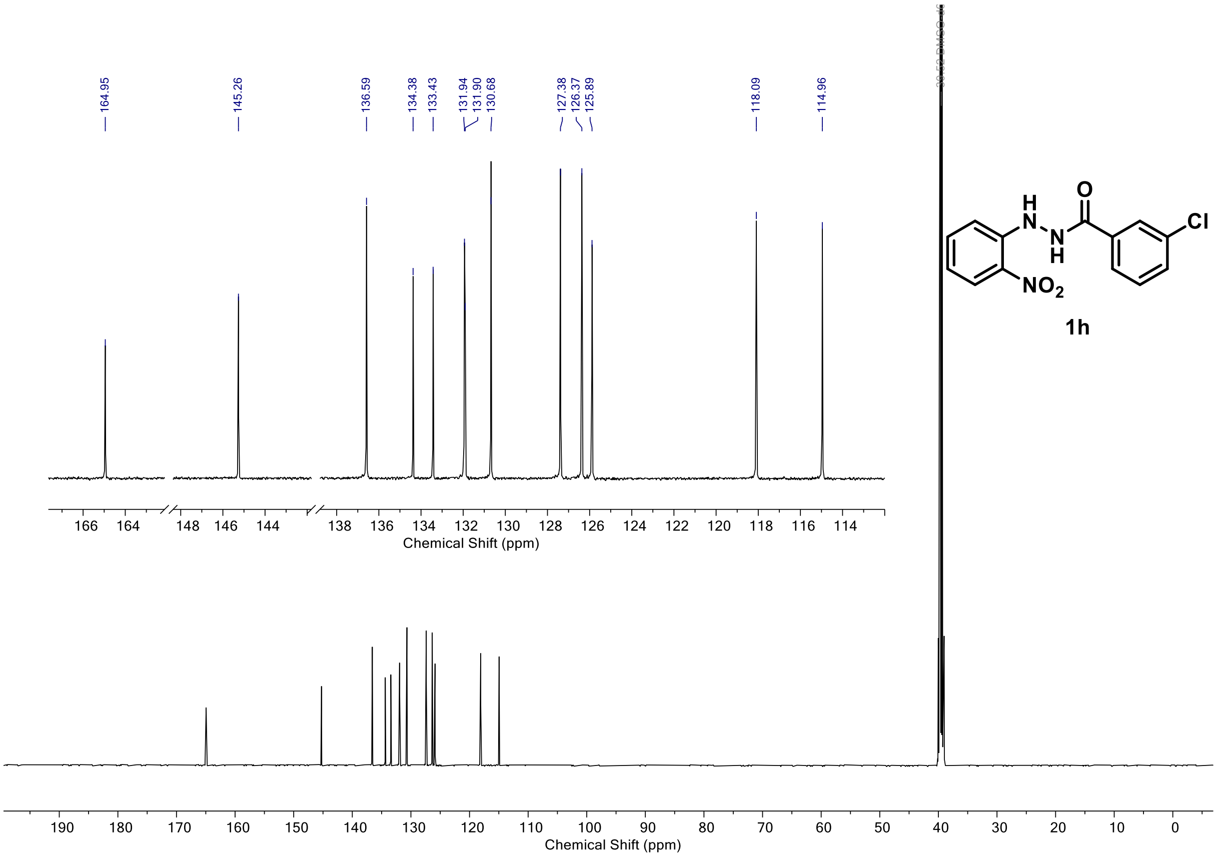

Figure S44: $^{13}C\{^{1}H\}$ NMR (126 MHz, DMSO-d_6): 3-Chloro-*N*'-(2-nitrophenyl)benzohydrazide (**1h**).

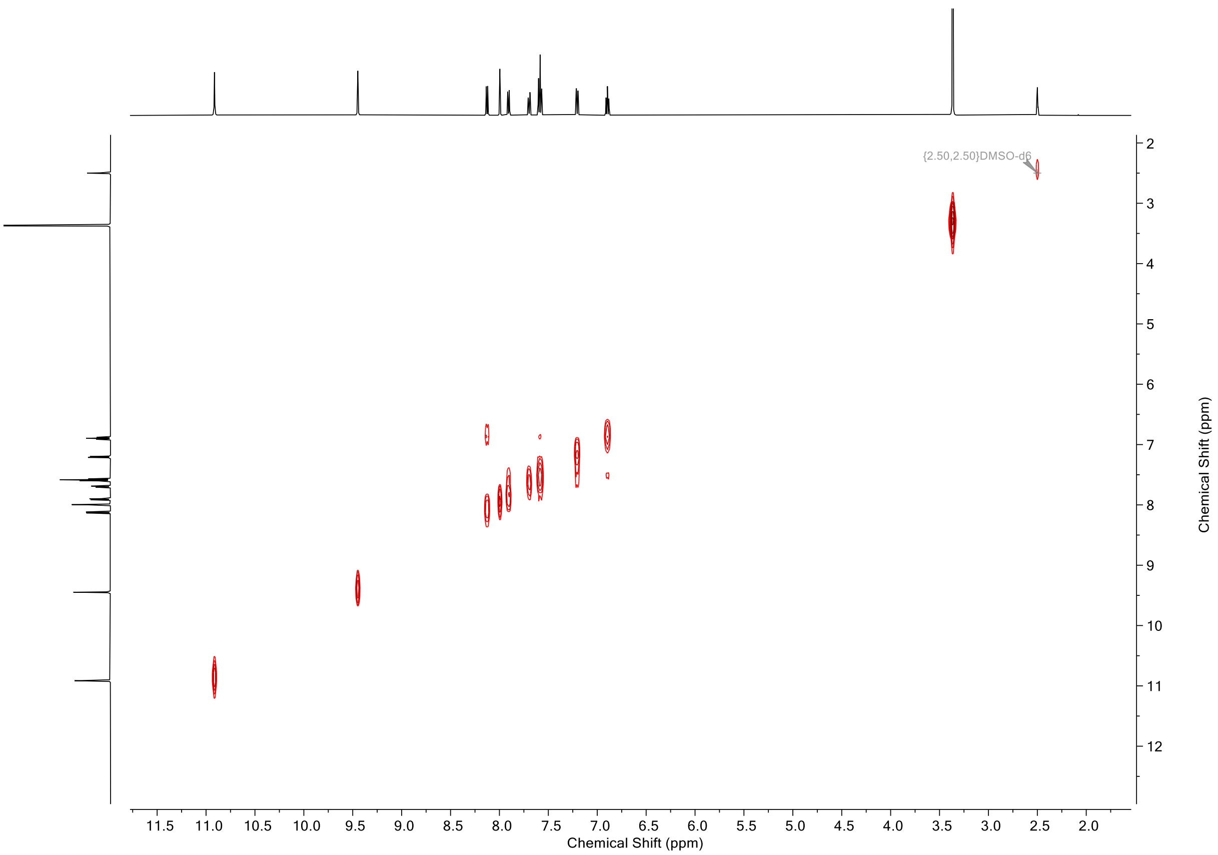

Figure S45: COSY (DMSO-d_6): 3-Chloro-*N*'-(2-nitrophenyl)benzohydrazide (**1h**).

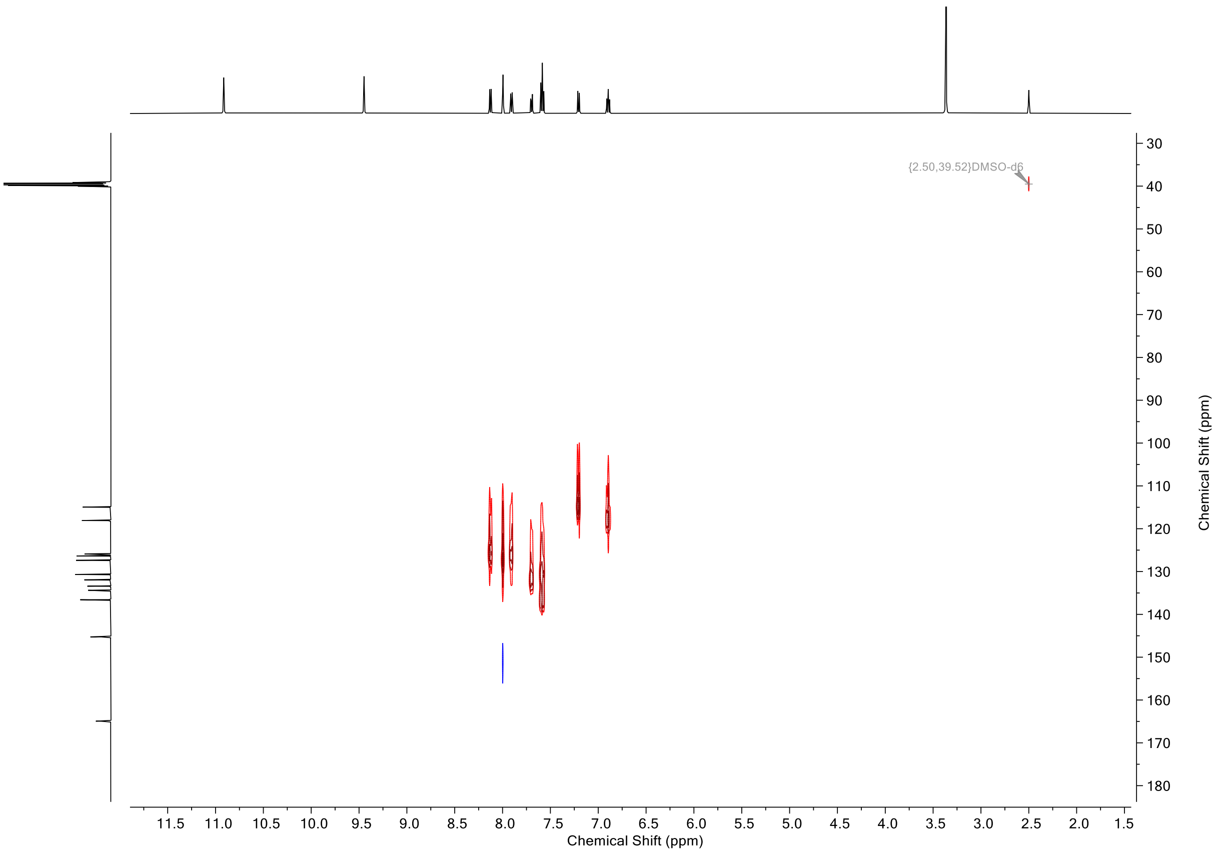

Figure S46: HSQC (DMSO-d_6): 3-Chloro-*N*'-(2-nitrophenyl)benzohydrazide (**1h**).

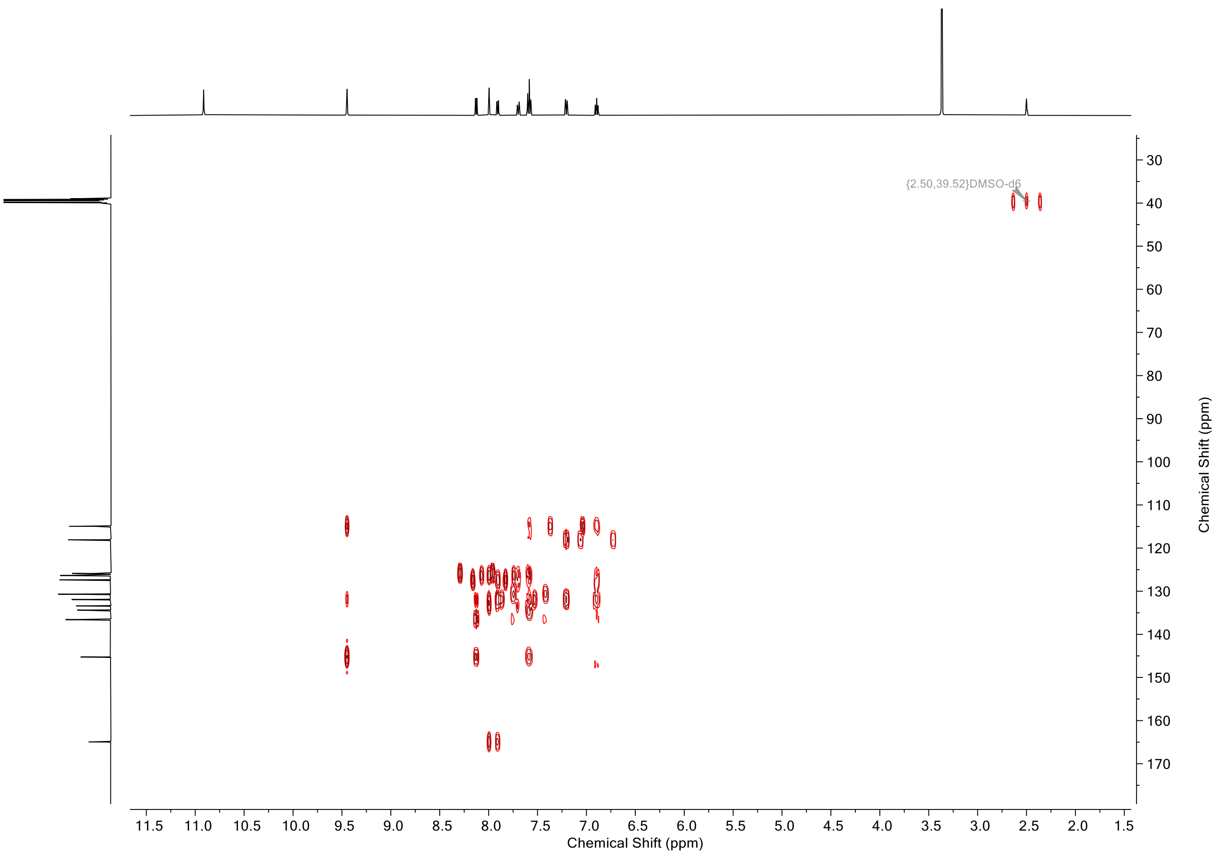

Figure S47: HMBC (DMSO-d_6): 3-Chloro-*N*'-(2-nitrophenyl)benzohydrazide (**1h**).

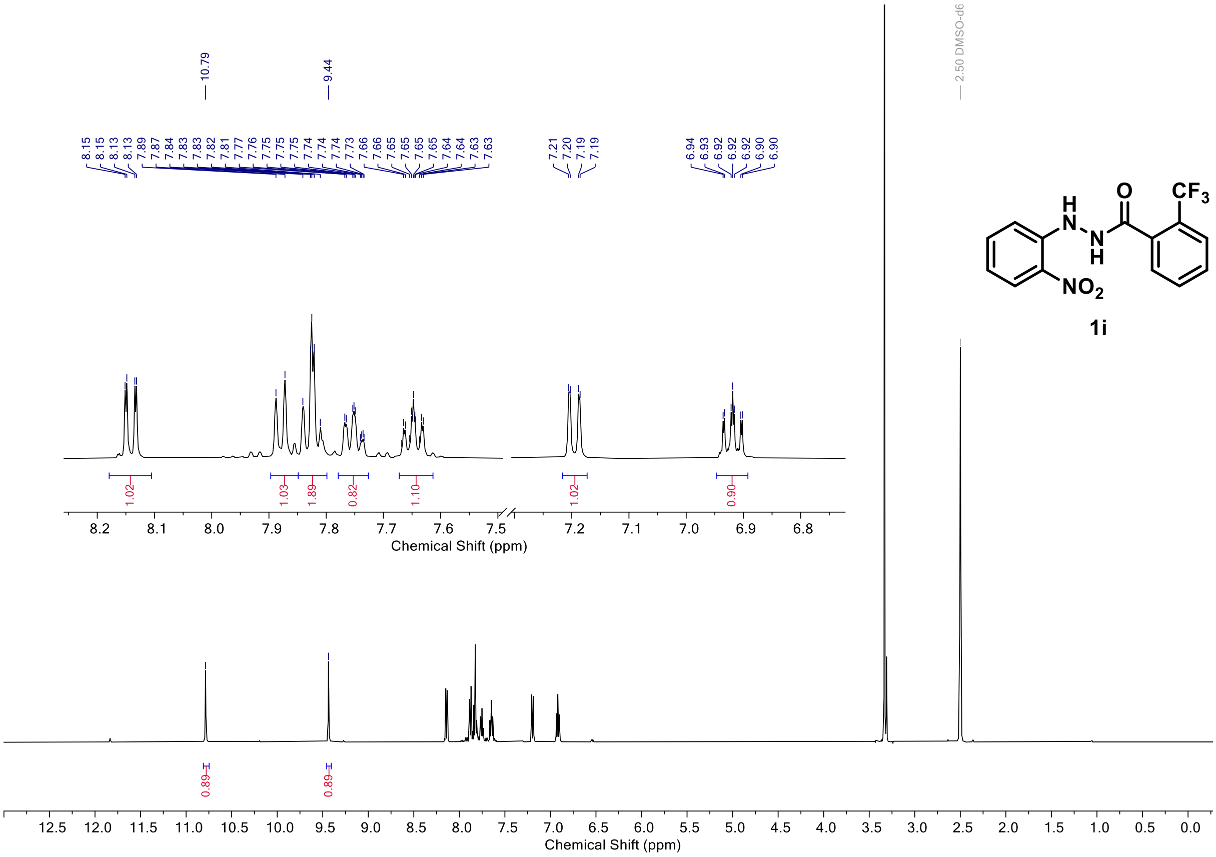

Figure S48: ^{1}H NMR (500 MHz, DMSO-d_6): *N*'-(2-Nitrophenyl)-2-(trifluoromethyl)benzohydrazide (**1i**).

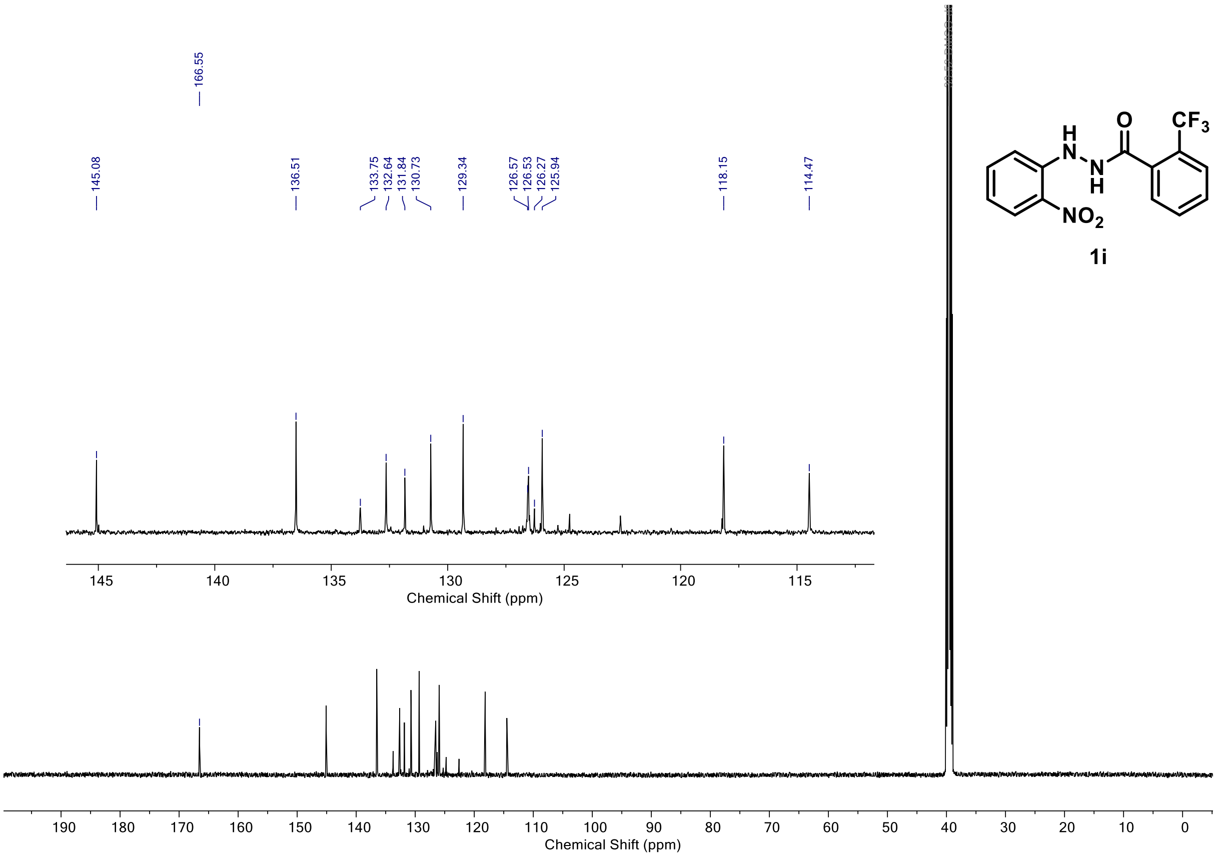

Figure S49: $^{13}C\{^{1}H\}$ NMR (126 MHz, DMSO-d_6): *N*'-(2-Nitrophenyl)-2-(trifluoromethyl)benzohydrazide (**1i**).

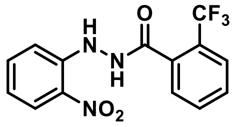

1i

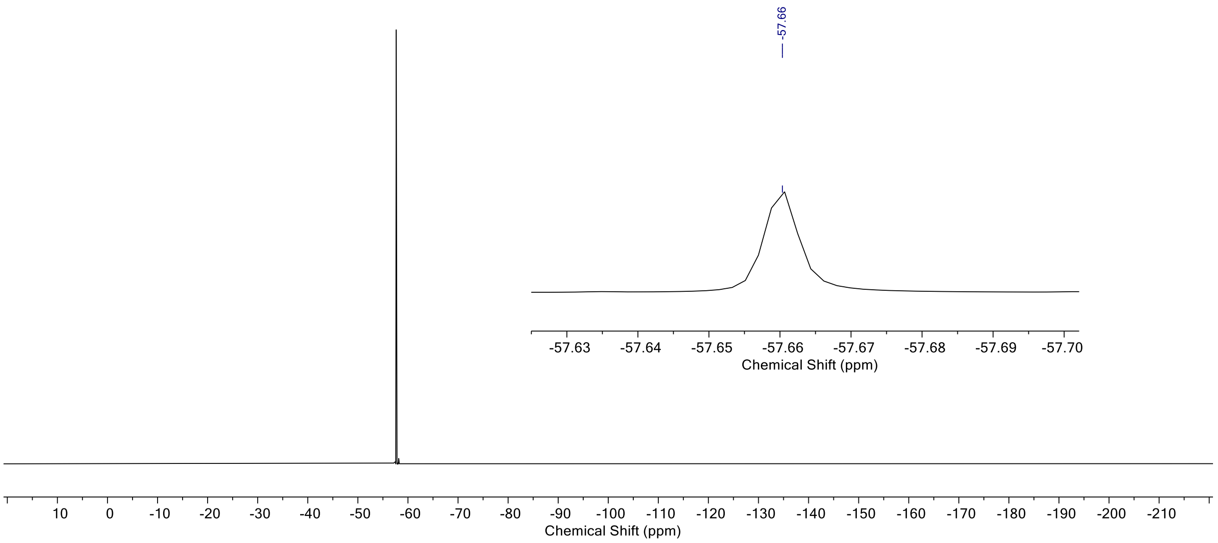

Figure S50: ^{19}F NMR (470 MHz, DMSO-d_6): *N*'-(2-Nitrophenyl)-2-(trifluoromethyl)benzohydrazide (**1i**).

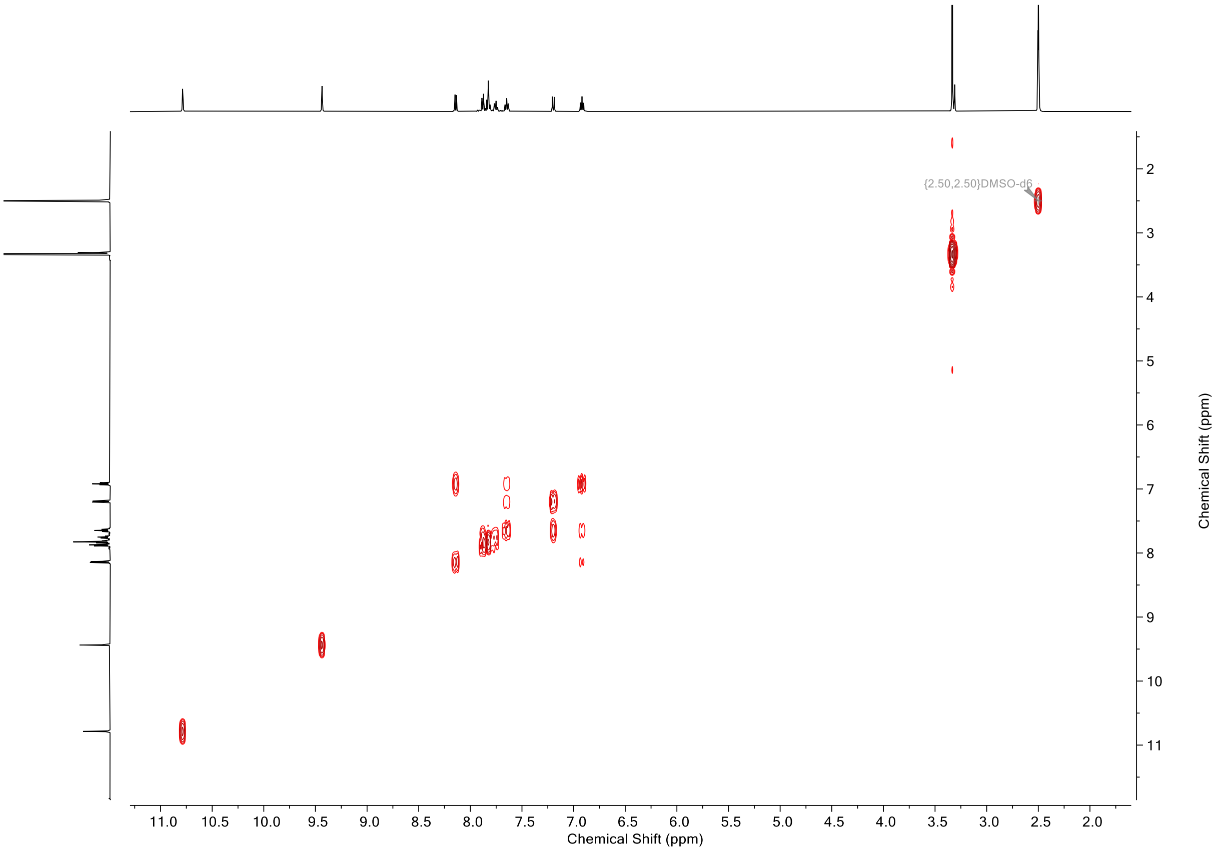

Figure S51: COSY (DMSO-d_6): *N*'-(2-Nitrophenyl)-2-(trifluoromethyl)benzohydrazide (**1i**).

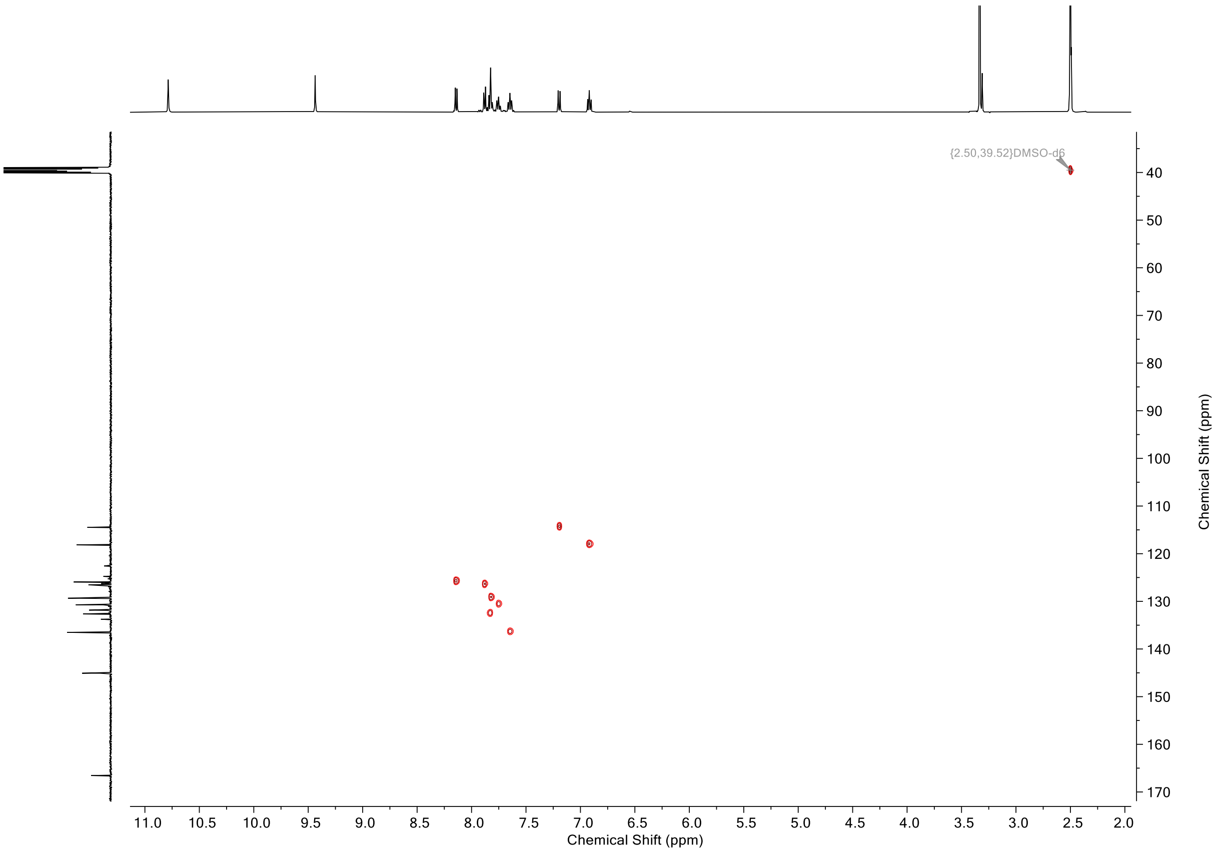

Figure S52: HSQC (DMSO-d_6): *N*'-(2-Nitrophenyl)-2-(trifluoromethyl)benzohydrazide (**1i**).

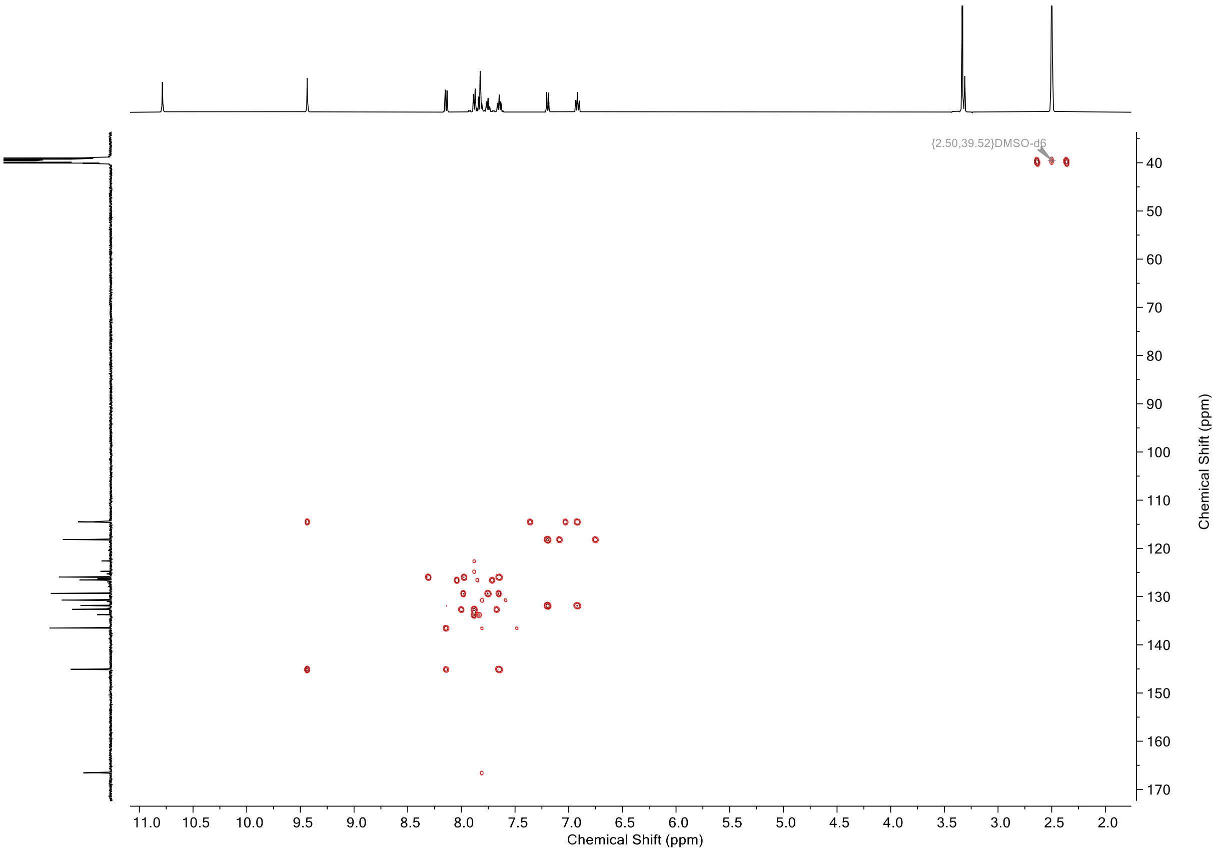

Figure S53: HMBC (DMSO-d_6): *N*'-(2-Nitrophenyl)-2-(trifluoromethyl)benzohydrazide (**1i**).

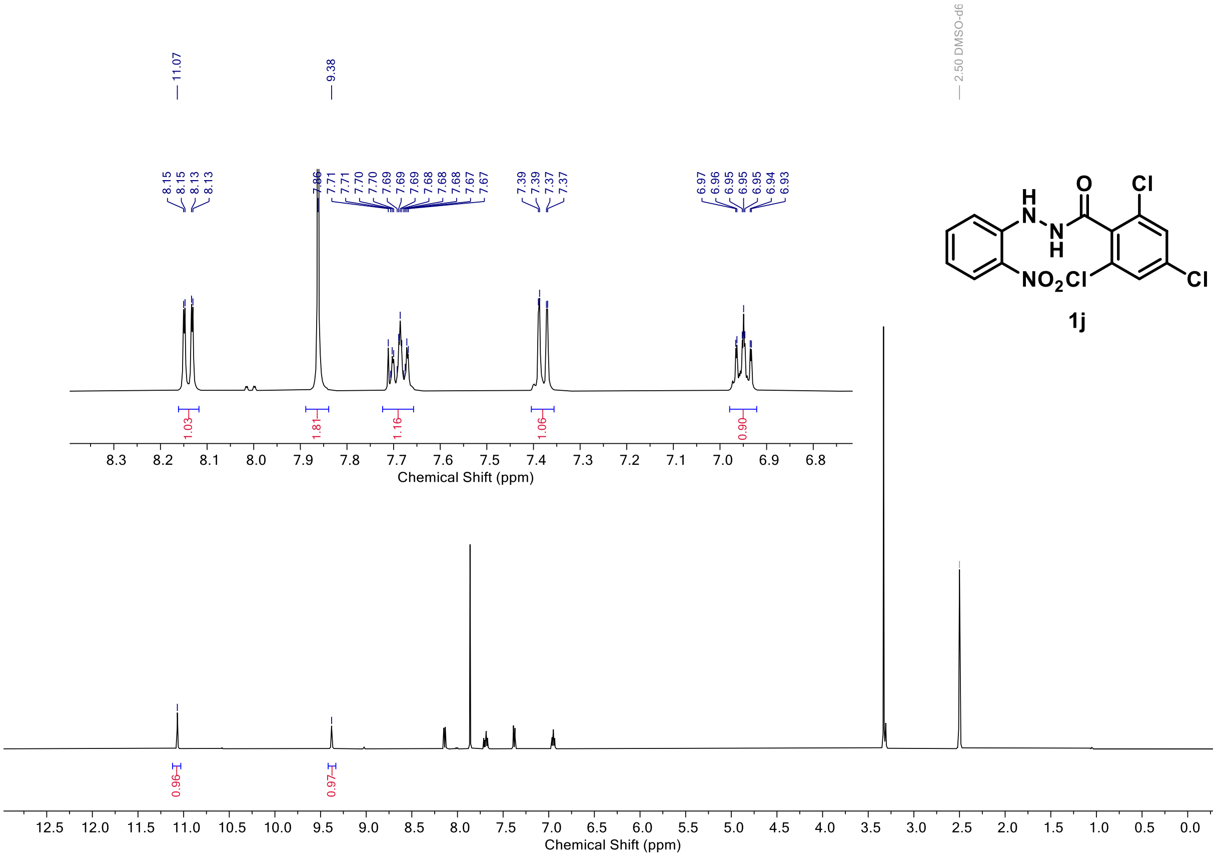

Figure S54: ^{1}H NMR (500 MHz, DMSO-d_6): 2,4,6-Trichloro-*N*'-(2-nitrophenyl)benzohydrazide (**1j**).

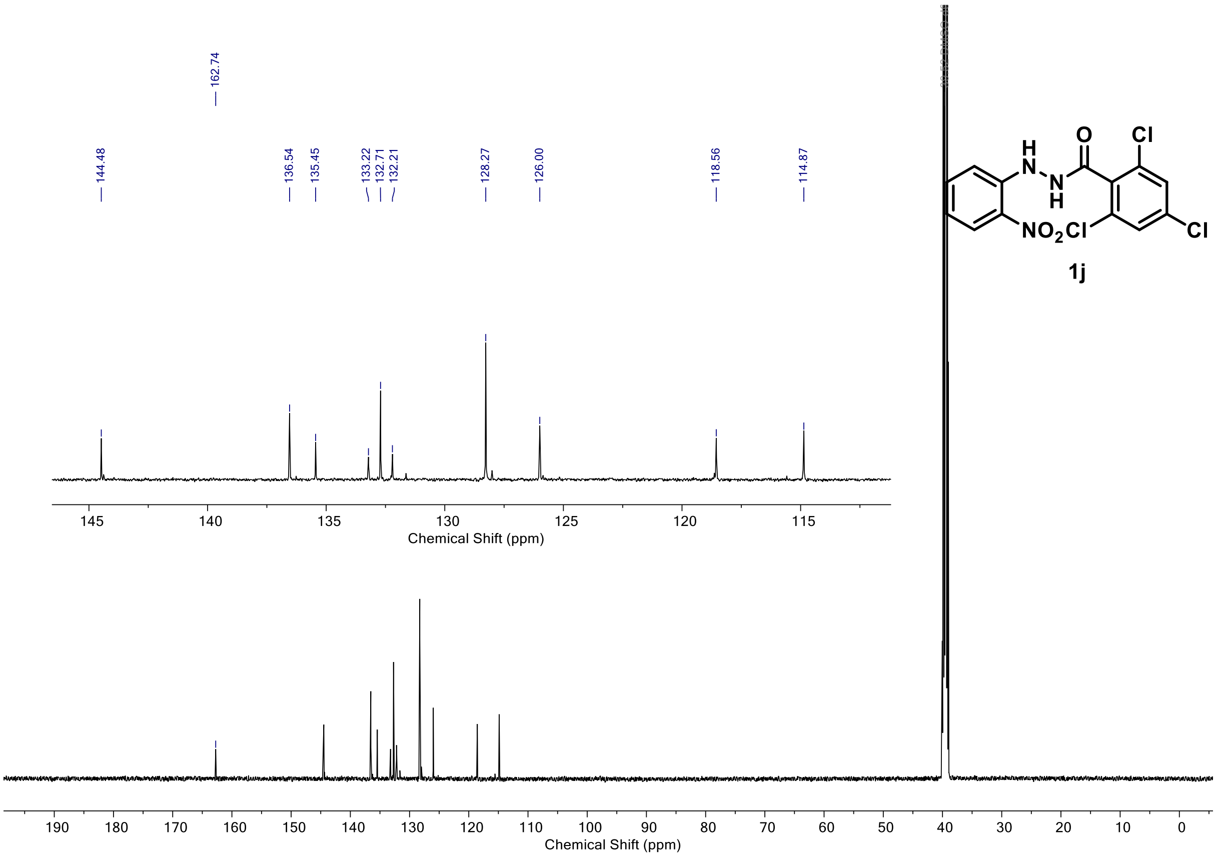

Figure S55: $^{13}C\{^{1}H\}$ NMR (126 MHz, DMSO-d_6): 2,4,6-Trichloro-*N*'-(2-nitrophenyl)benzohydrazide (**1j**).

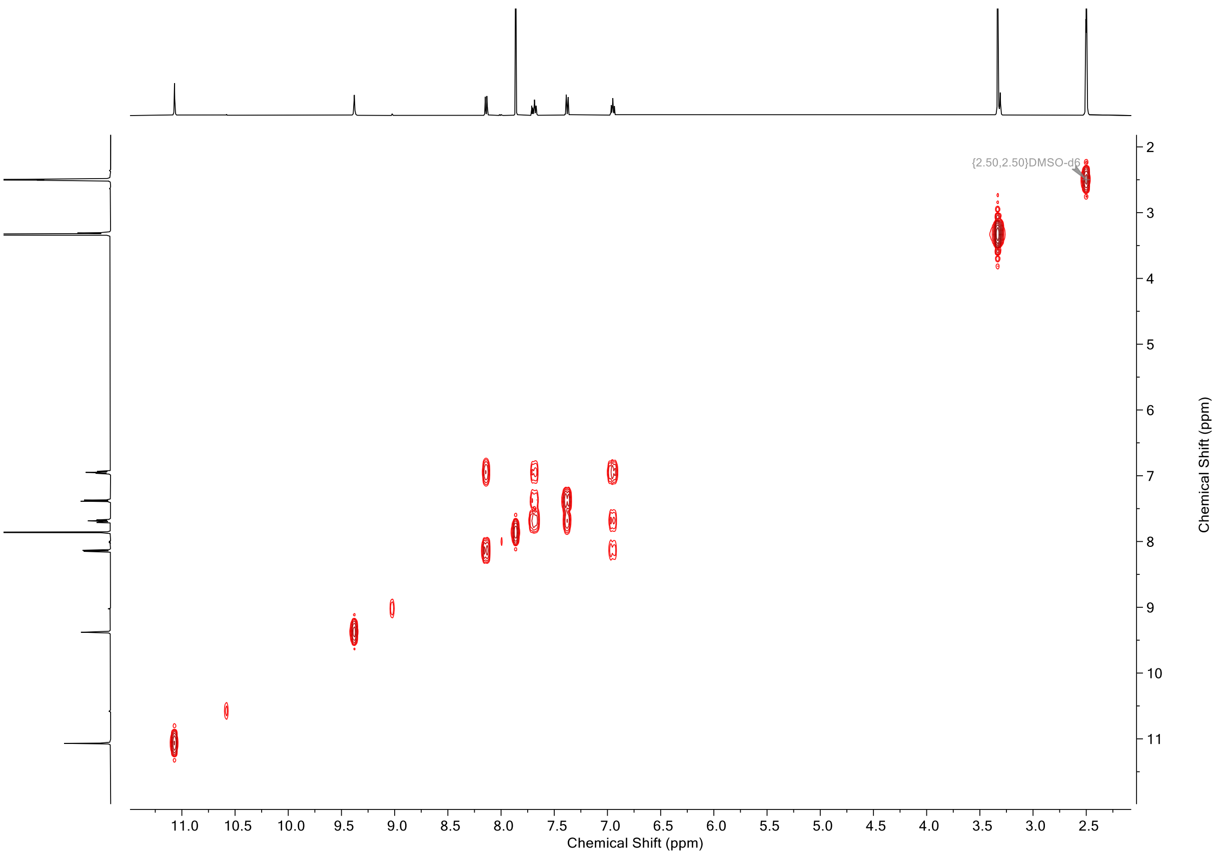

Figure S56: COSY (DMSO-d_6): 2,4,6-Trichloro-*N*'-(2-nitrophenyl)benzohydrazide (**1j**).

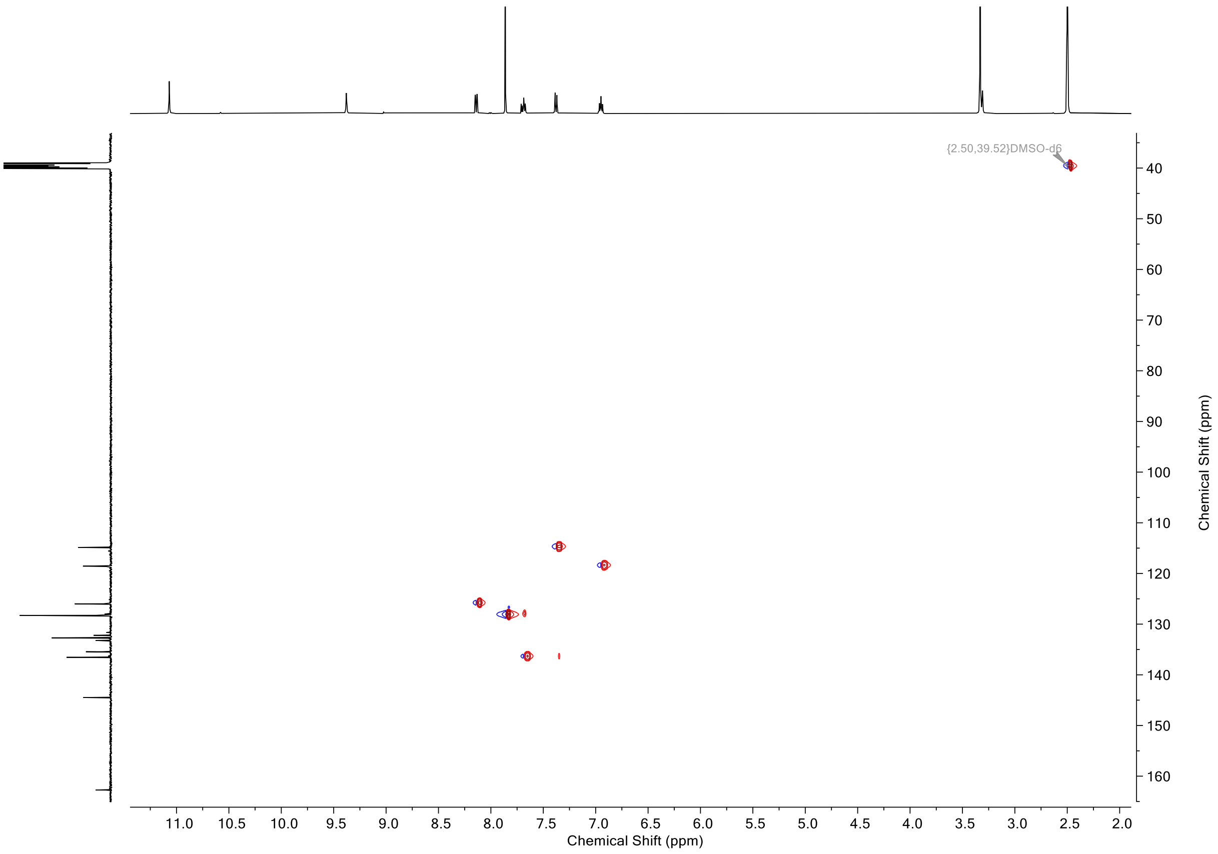

Figure S57: HSQC (DMSO-d_6): 2,4,6-Trichloro-*N*'-(2-nitrophenyl)benzohydrazide (**1j**).

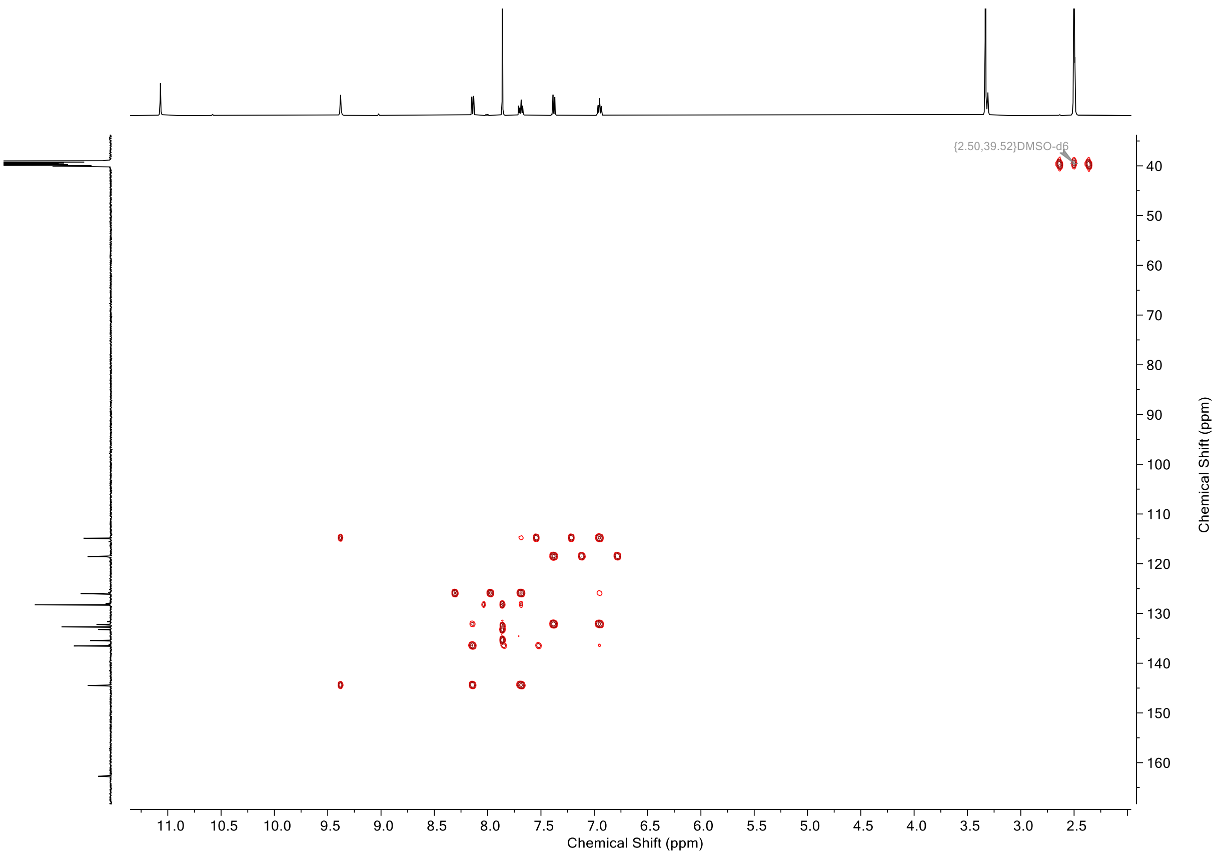

Figure S58: HMBC (DMSO-d_6): 2,4,6-Trichloro-*N*'-(2-nitrophenyl)benzohydrazide (**1j**).

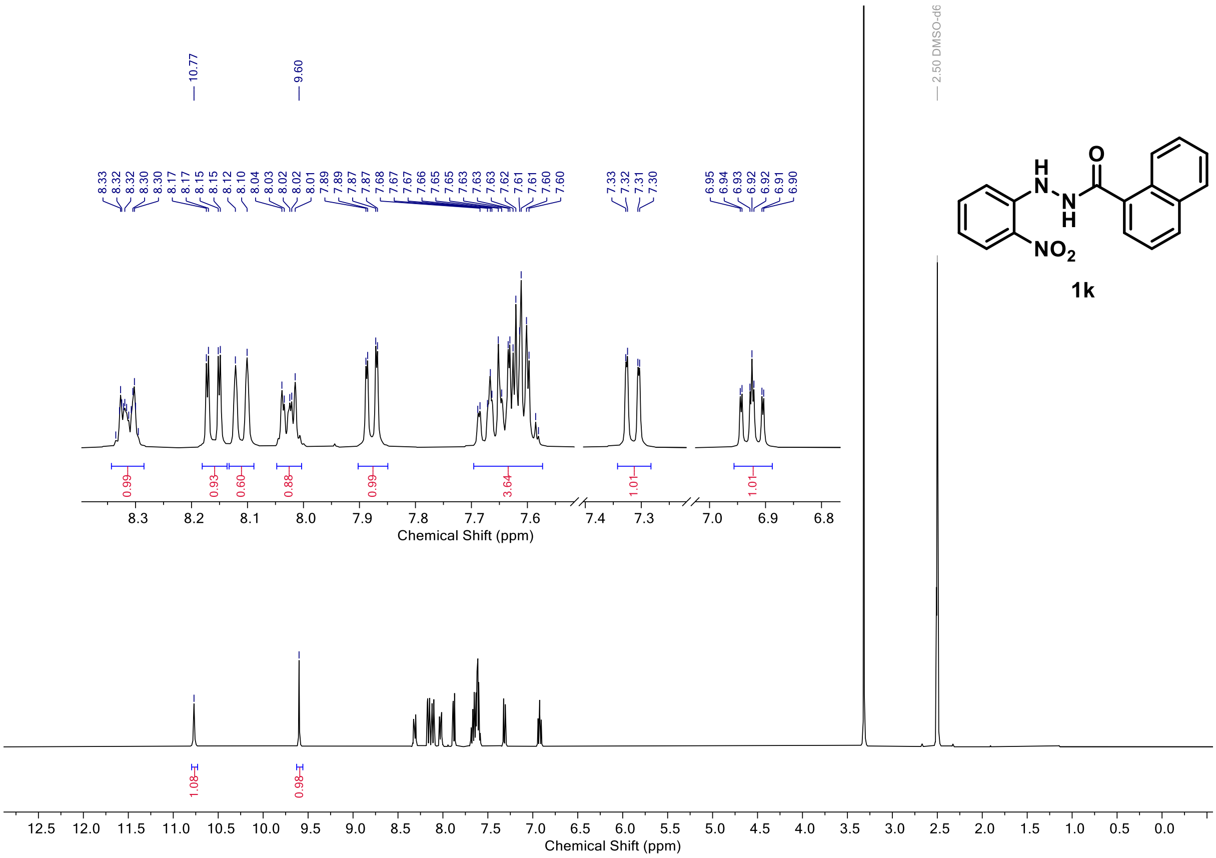

Figure S59: ^{1}H NMR (400 MHz, DMSO-d_6): *N*'-(2-Nitrophenyl)-1-naphthohydrazide (**1k**).

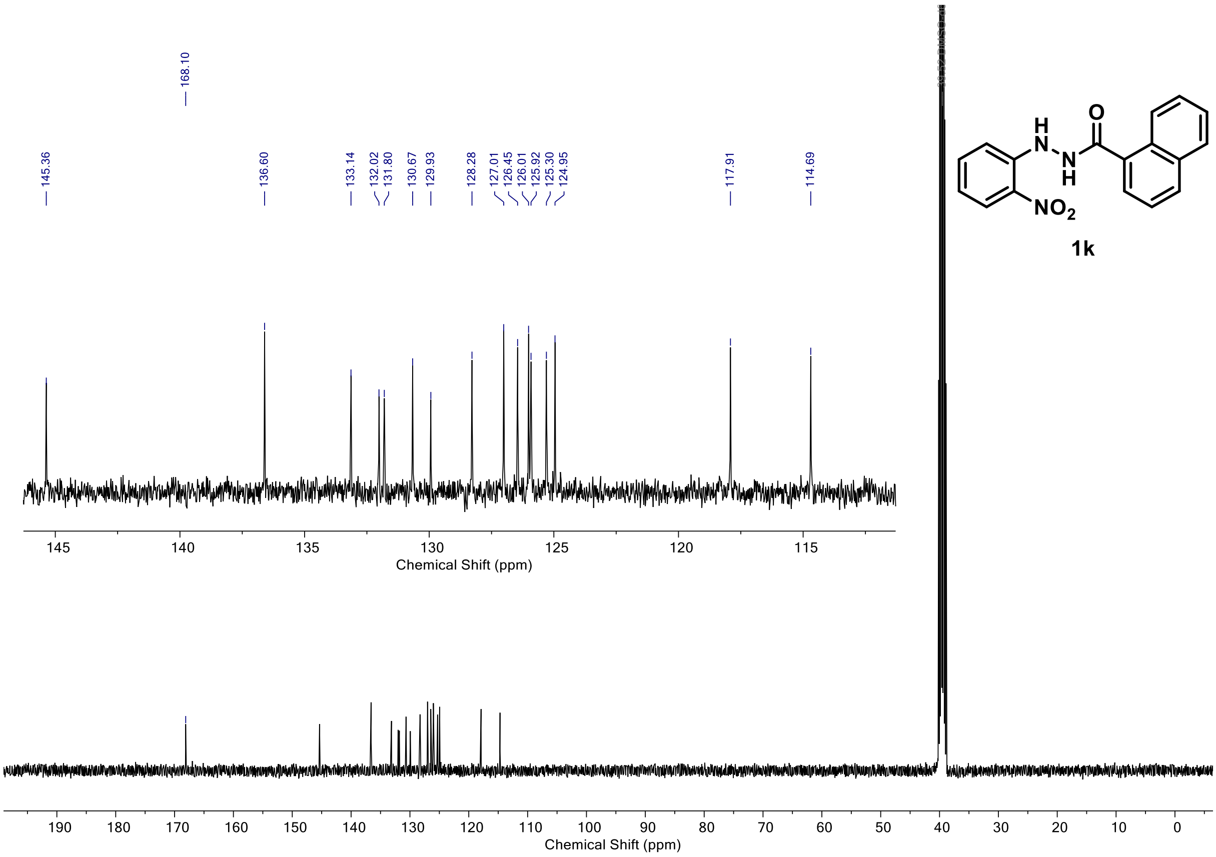

Figure S60: ^{13}C{^{1}H} NMR (101 MHz, DMSO-d_6): *N*'-(2-Nitrophenyl)-1-naphthohydrazide (**1k**).

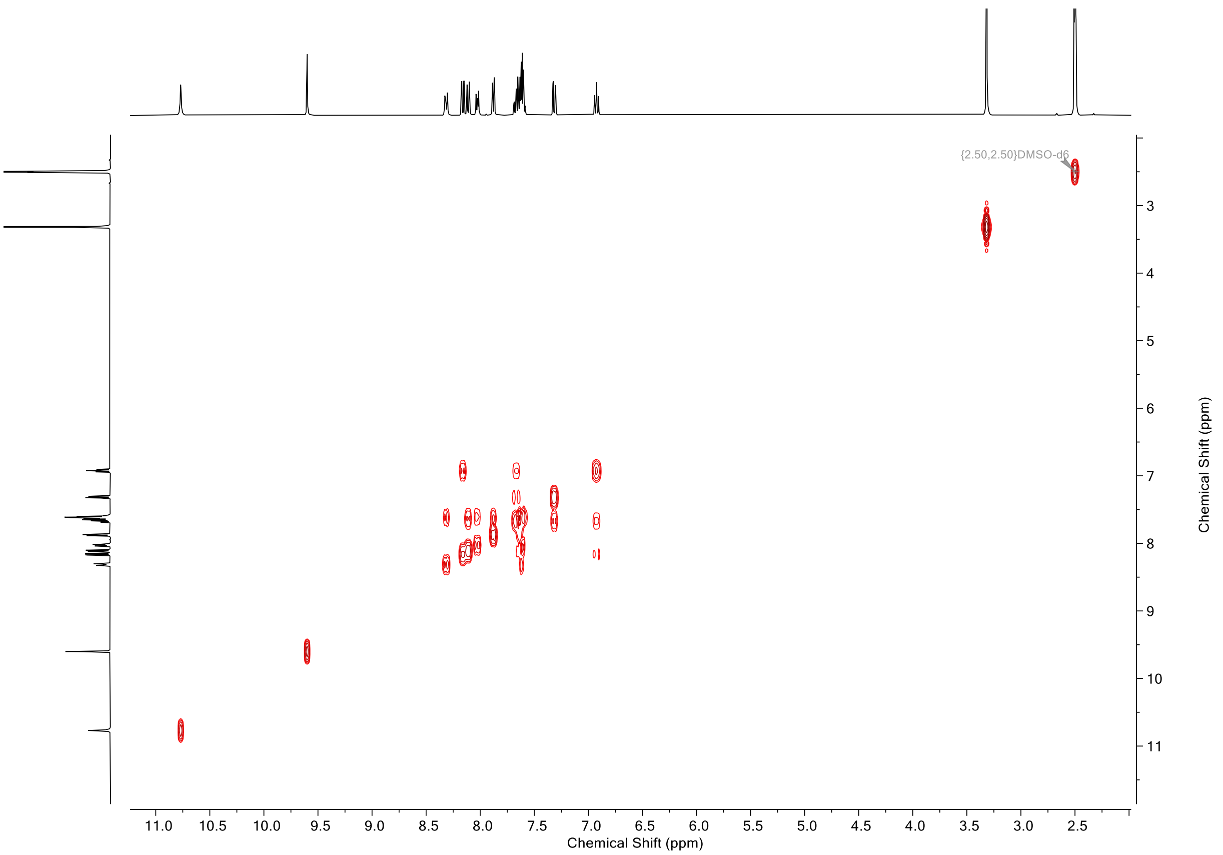

Figure S61: COSY (DMSO-d_6): *N*'-(2-Nitrophenyl)-1-naphthohydrazide (**1k**).

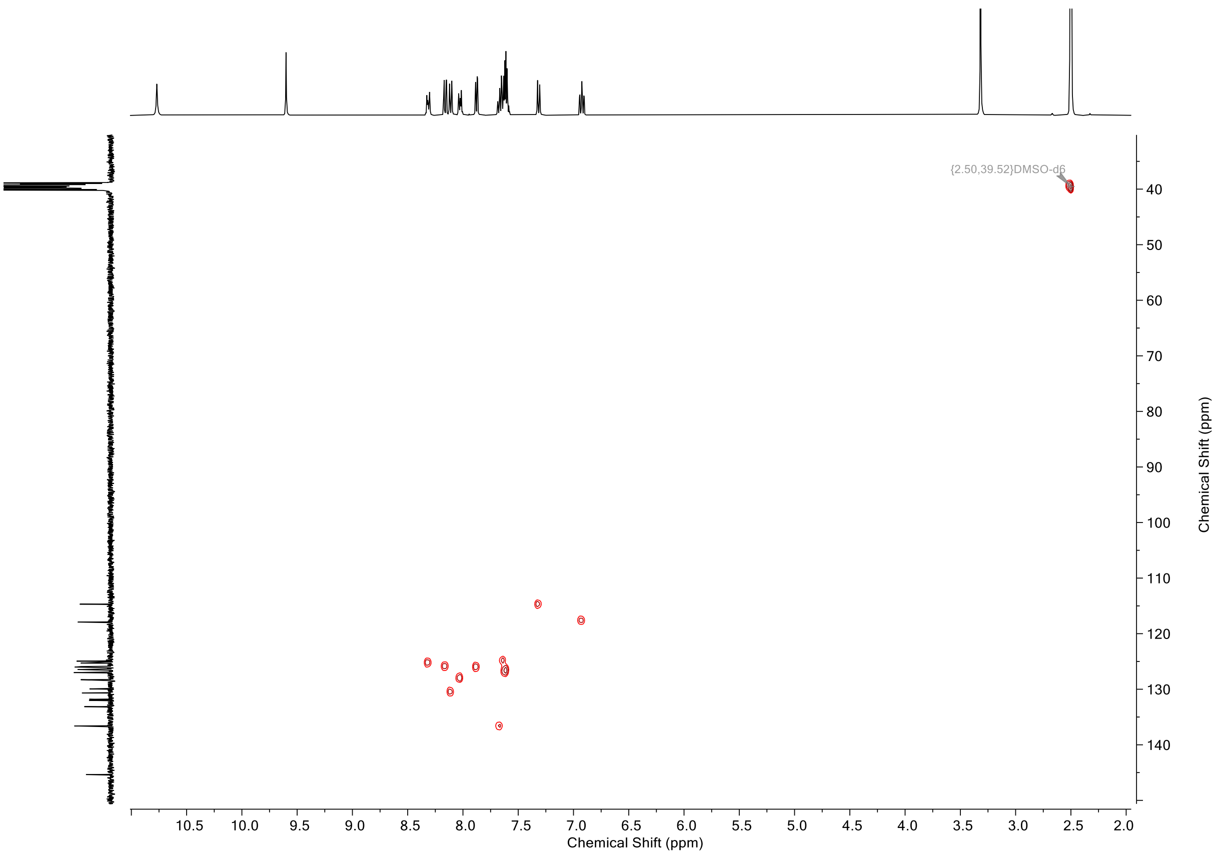

Figure S62: HSQC (DMSO-d_6): *N*'-(2-Nitrophenyl)-1-naphthohydrazide (**1k**).

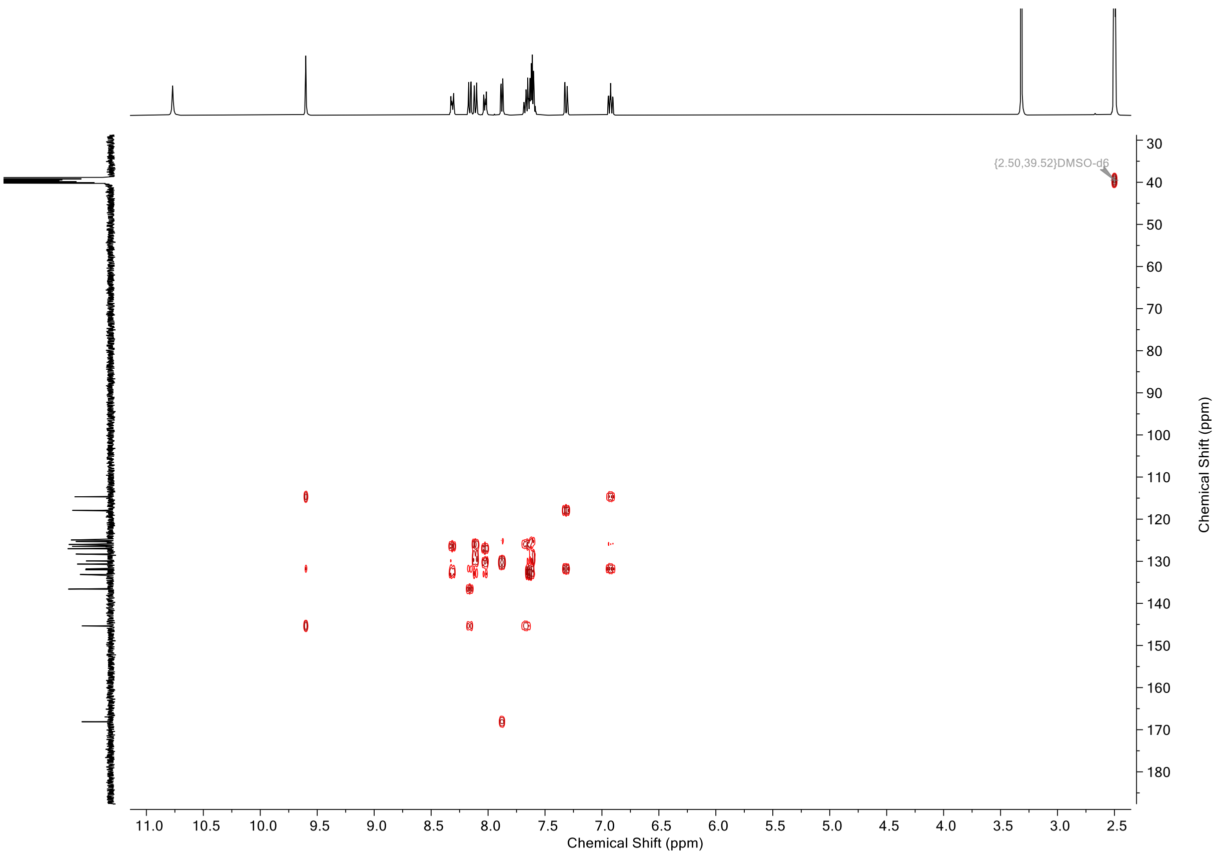

Figure S63: HMBC (DMSO-d_6): *N*'-(2-Nitrophenyl)-1-naphthohydrazide (**1k**).

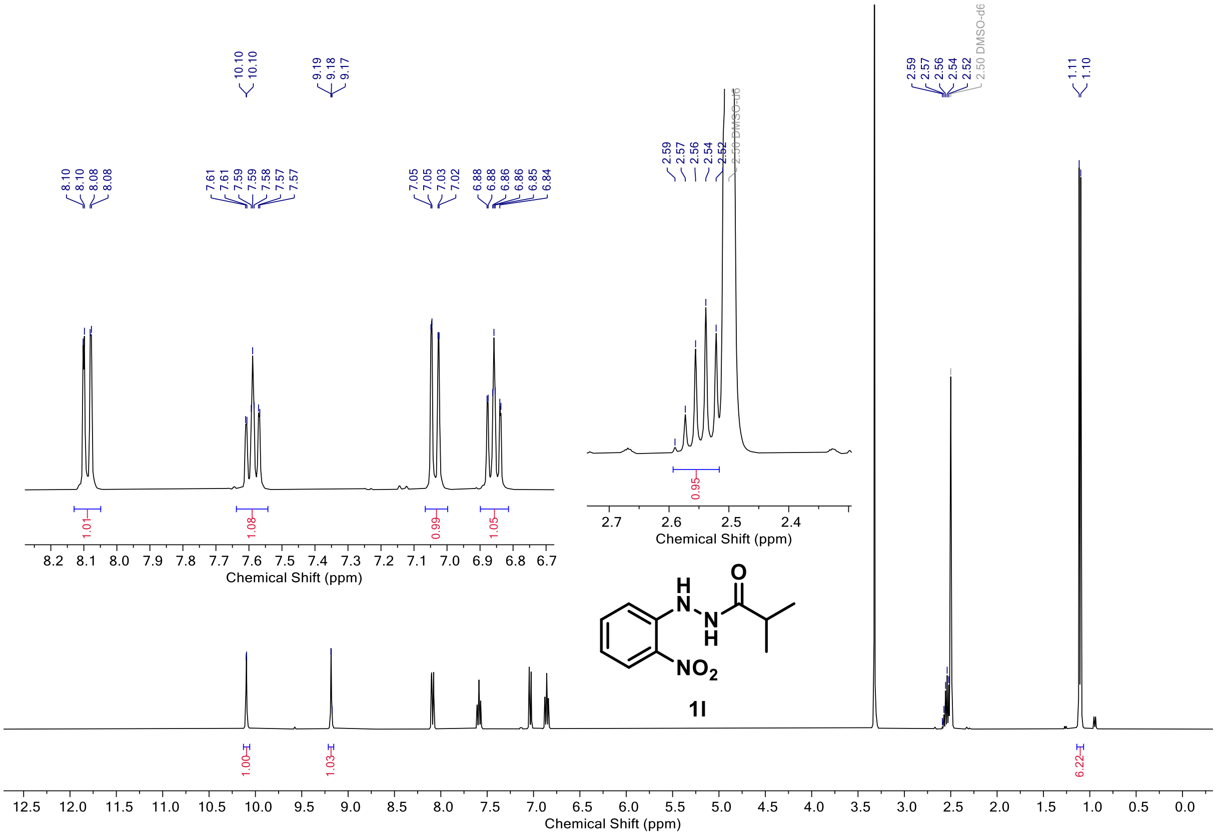

Figure S64: ^{1}H NMR (400 MHz, DMSO-d_6): *N*'-(2-Nitrophenyl)isobutyrohydrazide (**1l**).

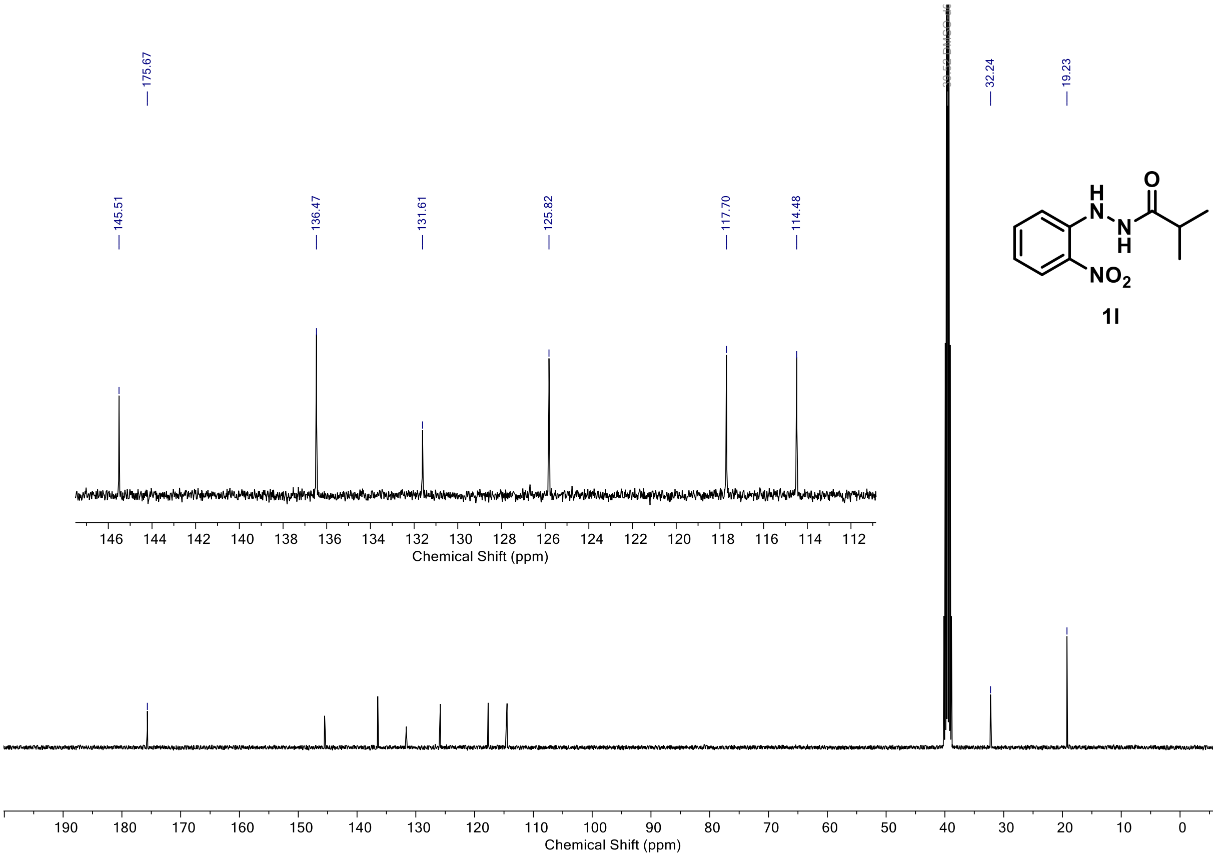

Figure S65: $^{13}C\{^{1}H\}$ NMR (101 MHz, DMSO-d_6): *N*'-(2-Nitrophenyl)isobutyrohydrazide (**1l**).

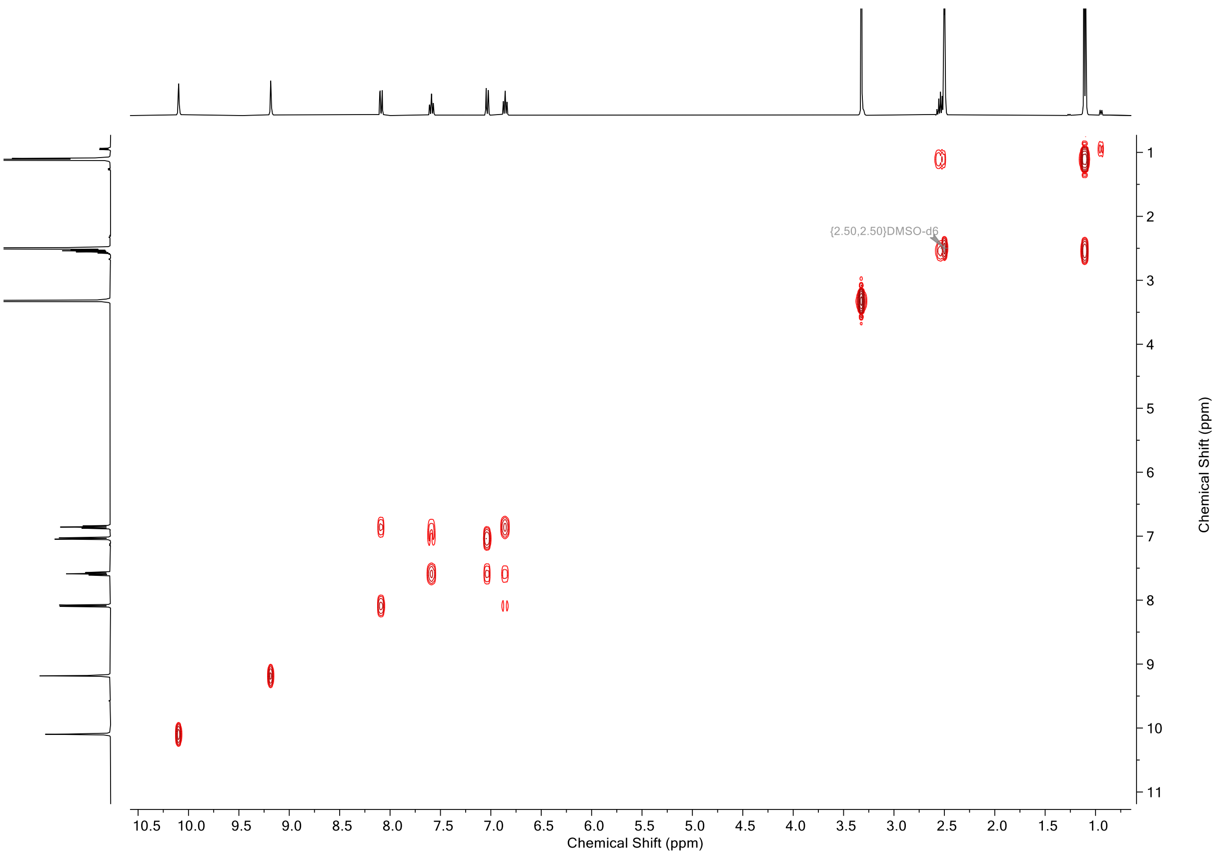

Figure S66: COSY (DMSO-d_6): *N*'-(2-Nitrophenyl)isobutyrohydrazide (**1l**).

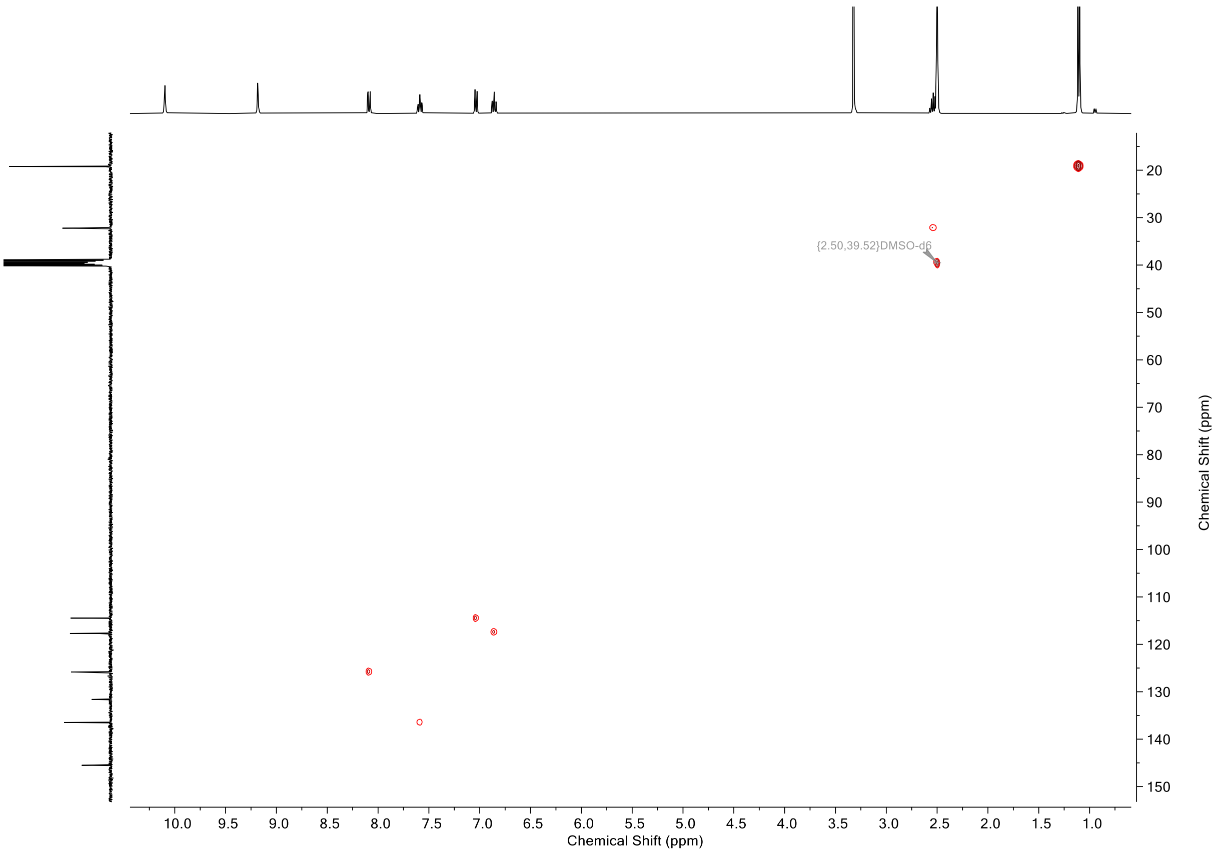

Figure S67: HSQC (DMSO-d_6): *N*'-(2-Nitrophenyl)isobutyrohydrazide (**1l**).

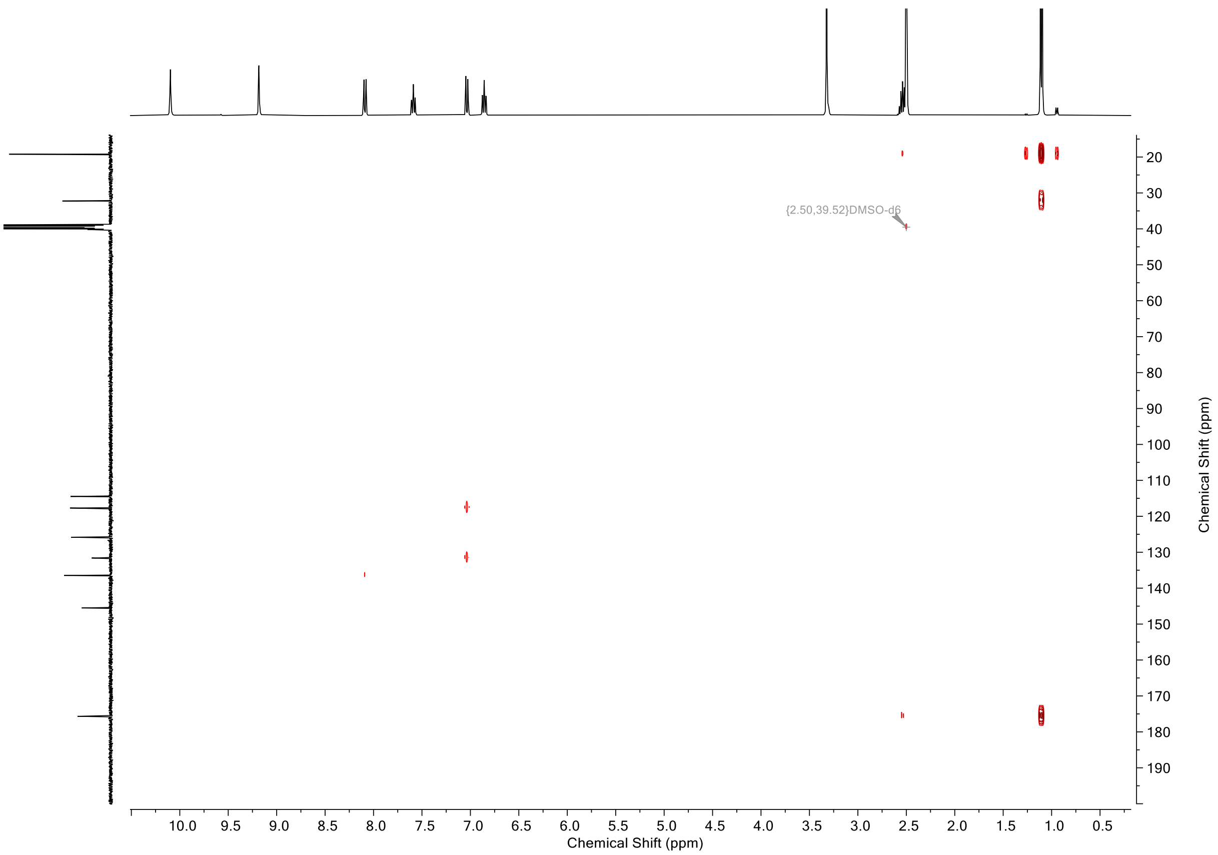

Figure S68: HMBC (DMSO-d_6): *N*'-(2-Nitrophenyl)isobutyrohydrazide (**1l**).

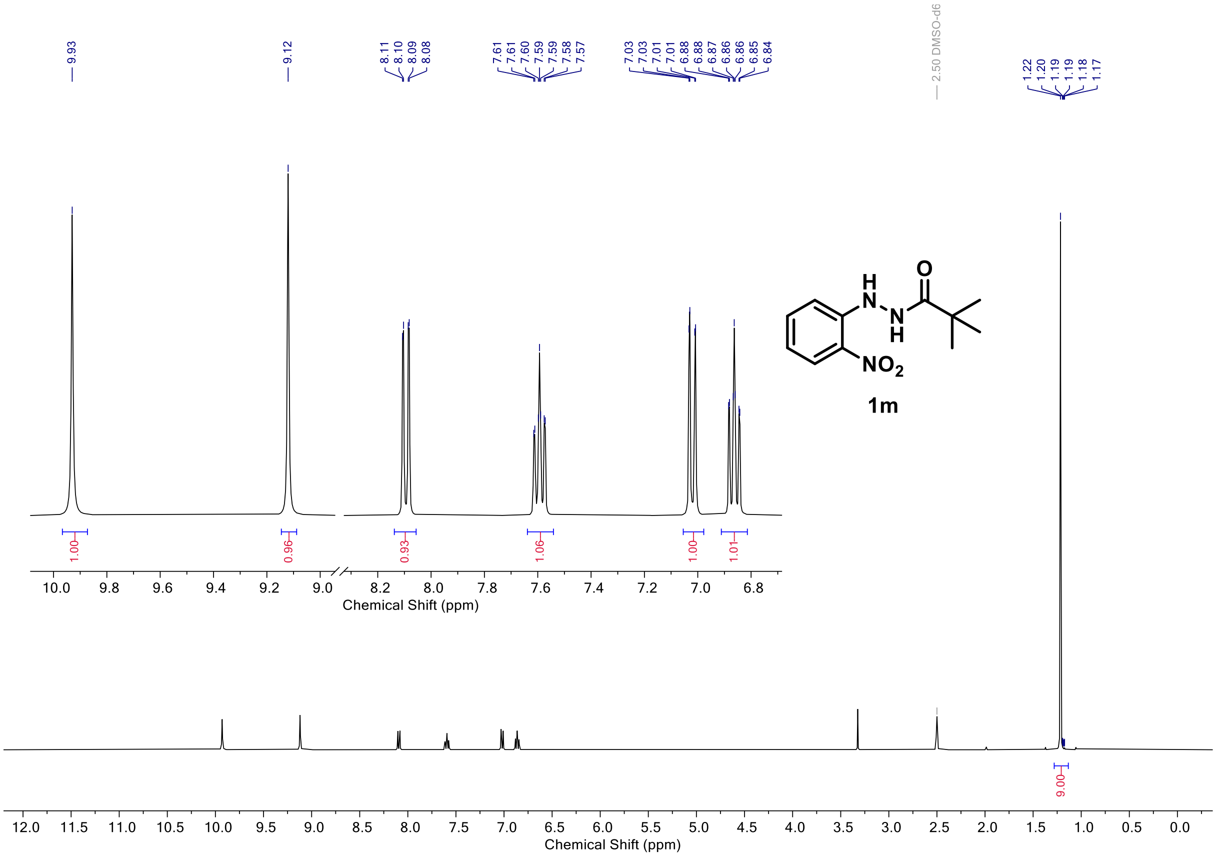

Figure S69: ^{1}H NMR (400 MHz, DMSO-d_6): *N*'-(2-Nitrophenyl)pivalohydrazide (**1m**).

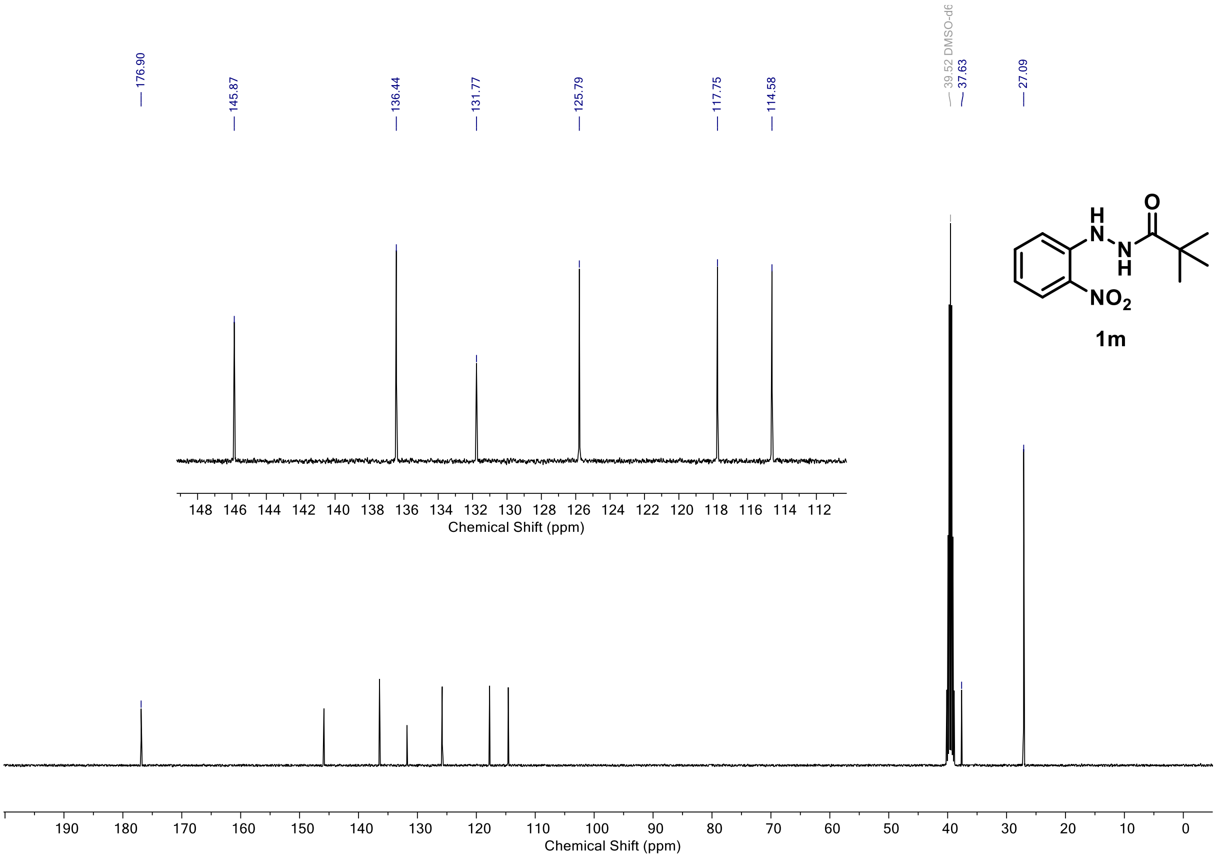

Figure S70: $^{13}C\{^{1}H\}$ NMR (101 MHz, DMSO-d_6): *N*'-(2-Nitrophenyl)pivalohydrazide (**1m**).

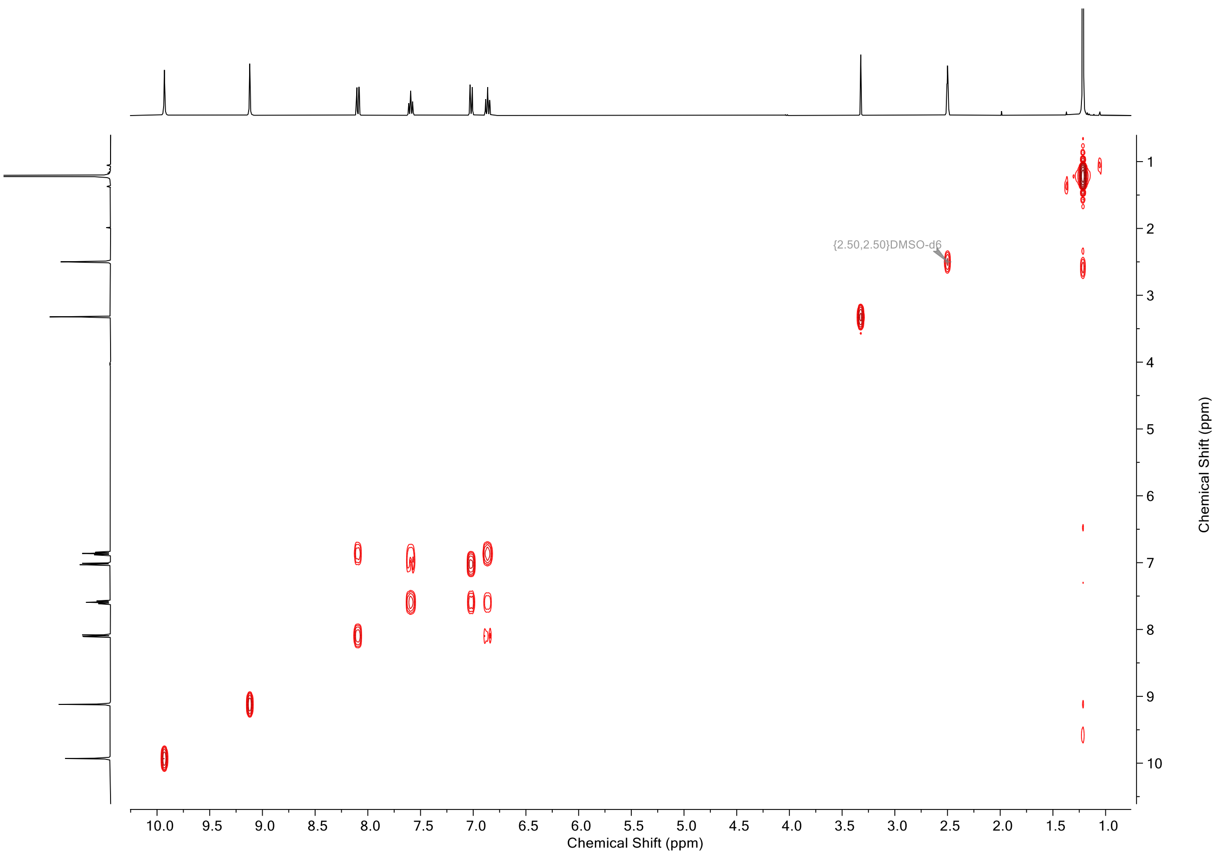

Figure S71: COSY (DMSO-d_6): *N*'-(2-Nitrophenyl)pivalohydrazide (**1m**).

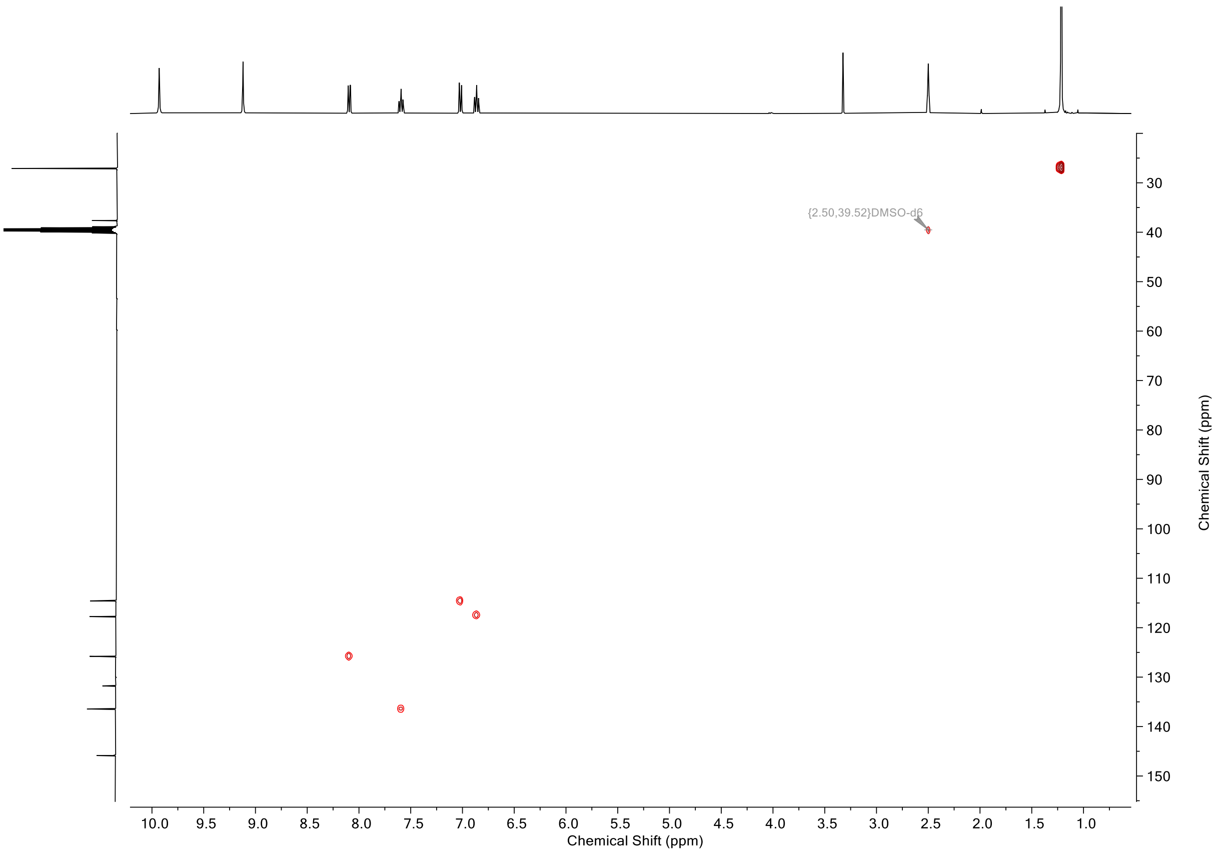

Figure S72: HSQC (DMSO-d_6): *N*'-(2-Nitrophenyl)pivalohydrazide (**1m**).

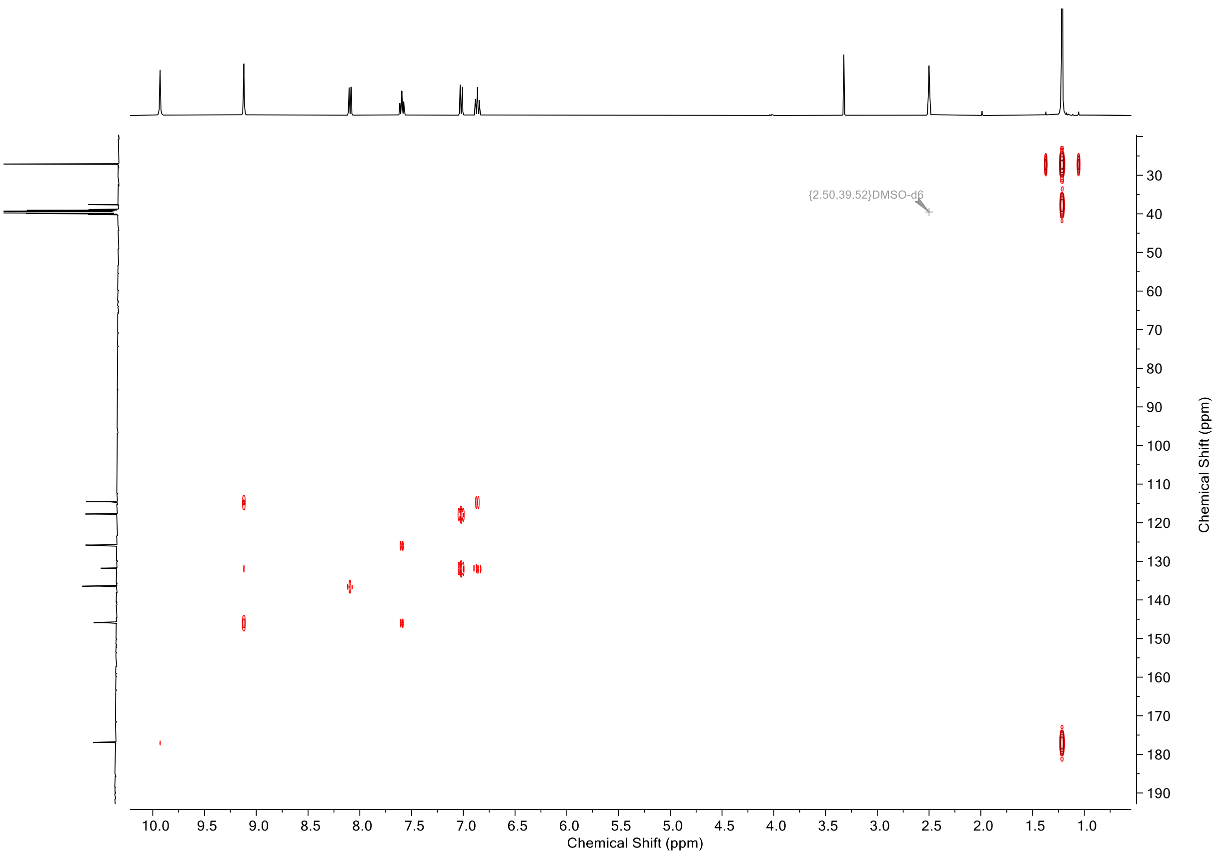

Figure S73: HMBC (DMSO-d_6): *N*'-(2-Nitrophenyl)pivalohydrazide (**1m**).

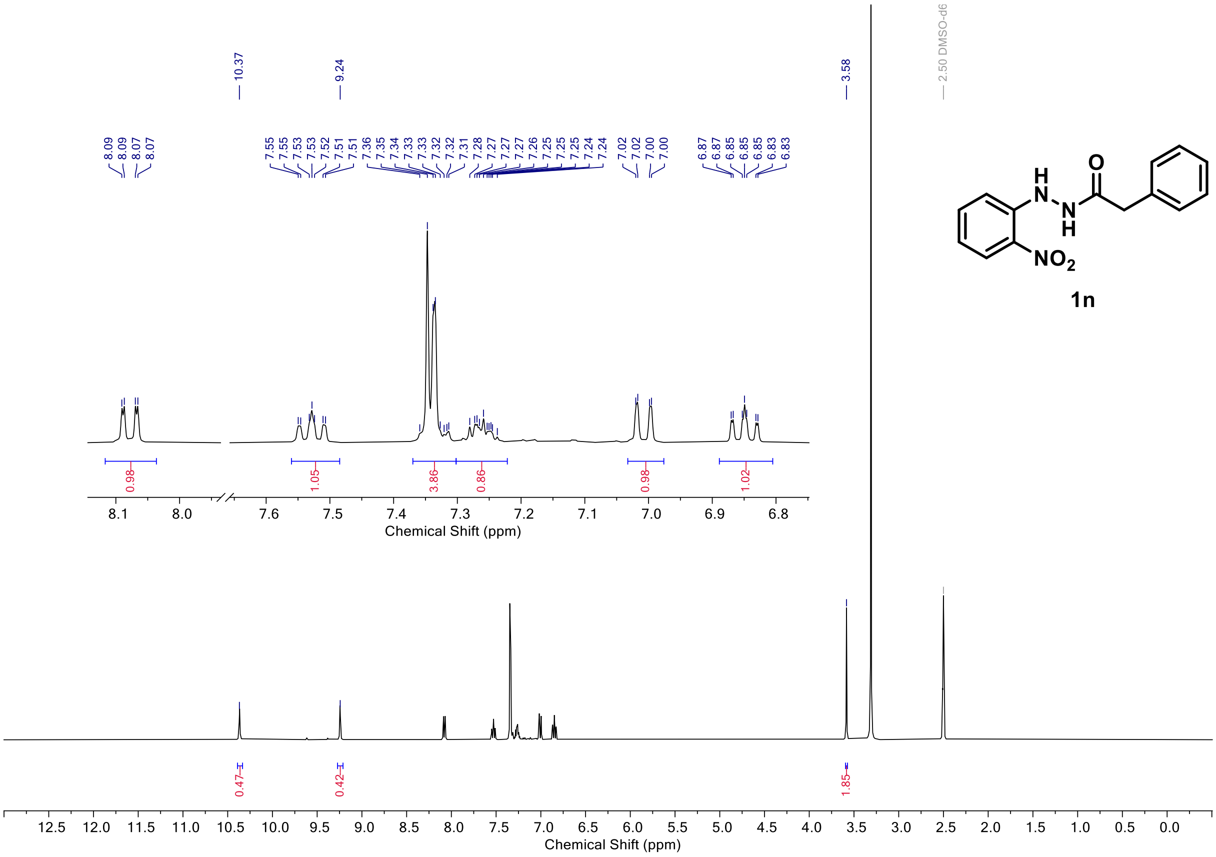

Figure S74: ^{1}H NMR (400 MHz, DMSO-d_6): *N*'-(2-Nitrophenyl)-2-phenylacetohydrazide (**1n**).

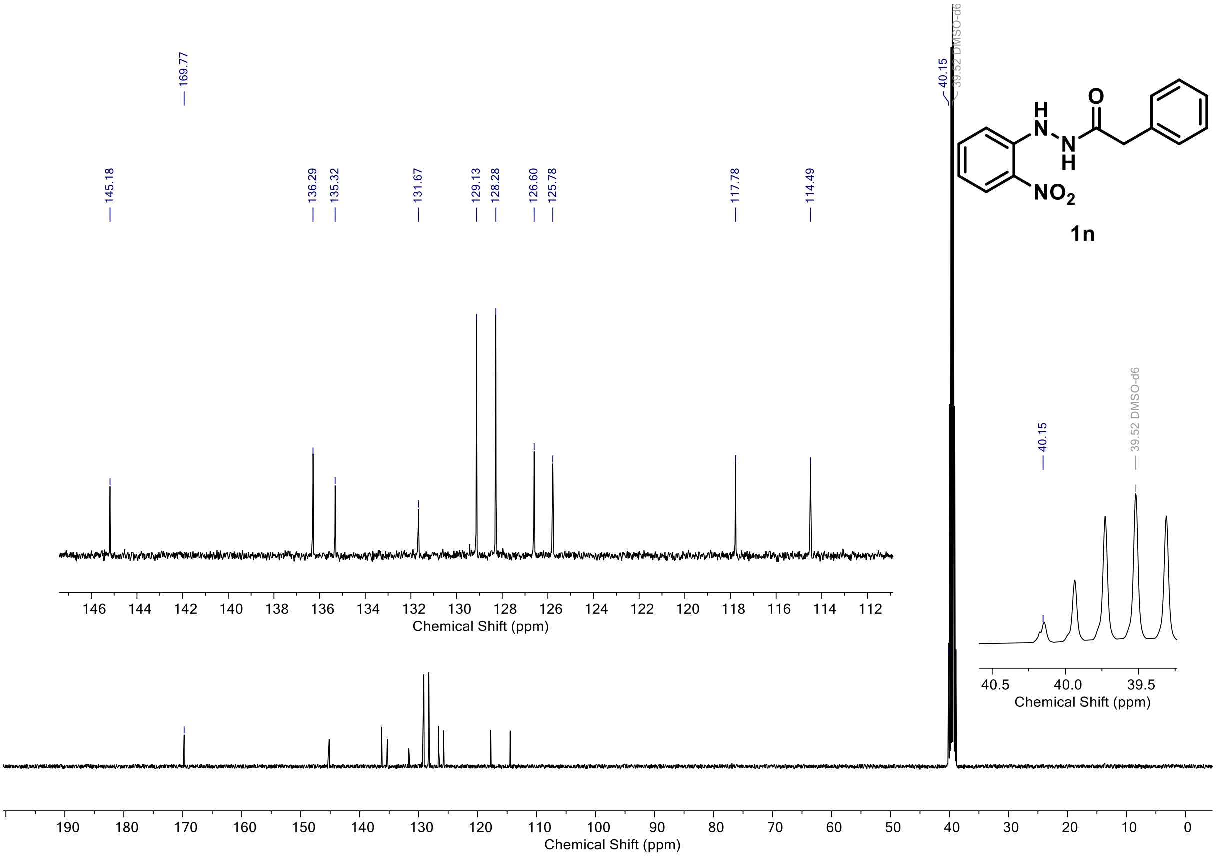

Figure S75: ^{13}C{^{1}H} NMR (101 MHz, DMSO-d_6): *N*'-(2-Nitrophenyl)-2-phenylacetohydrazide (**1n**).

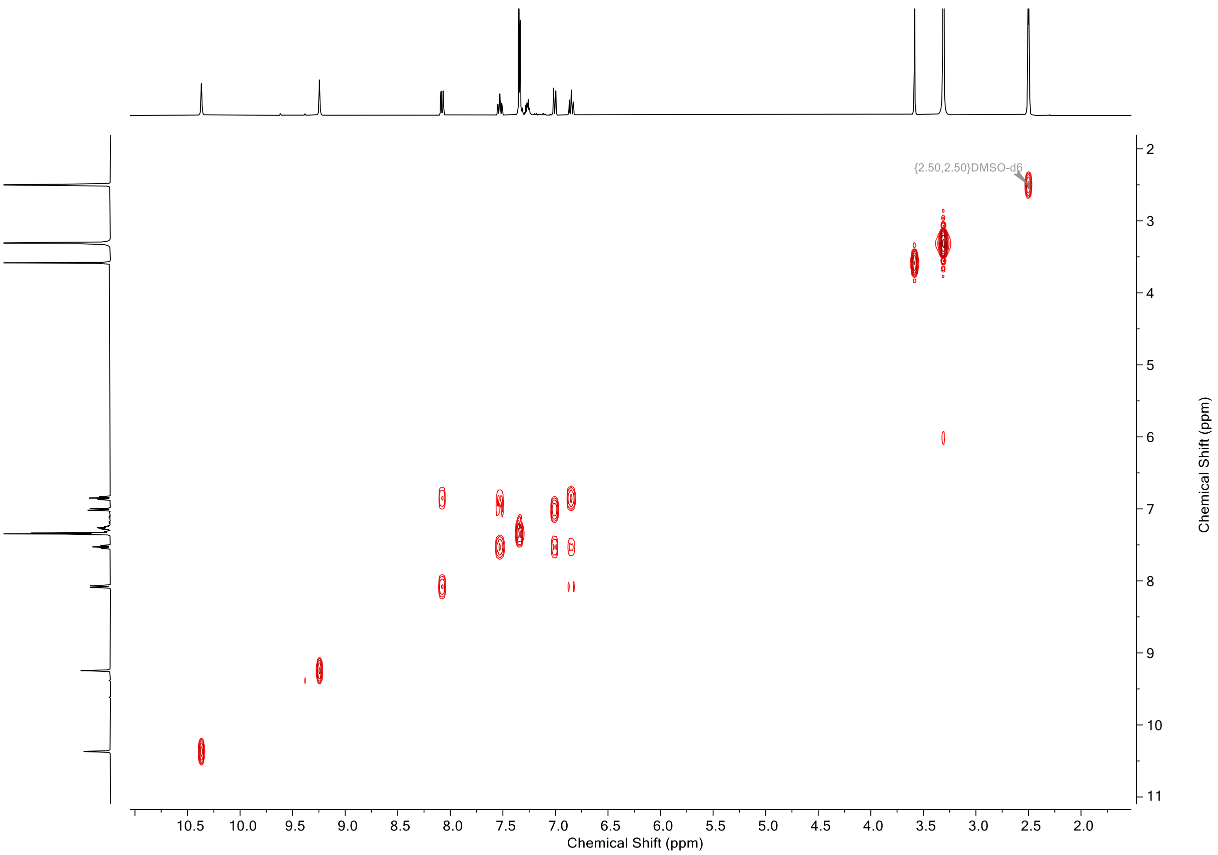

Figure S76: COSY (DMSO-d_6): *N*'-(2-Nitrophenyl)-2-phenylacetohydrazide (**1n**).

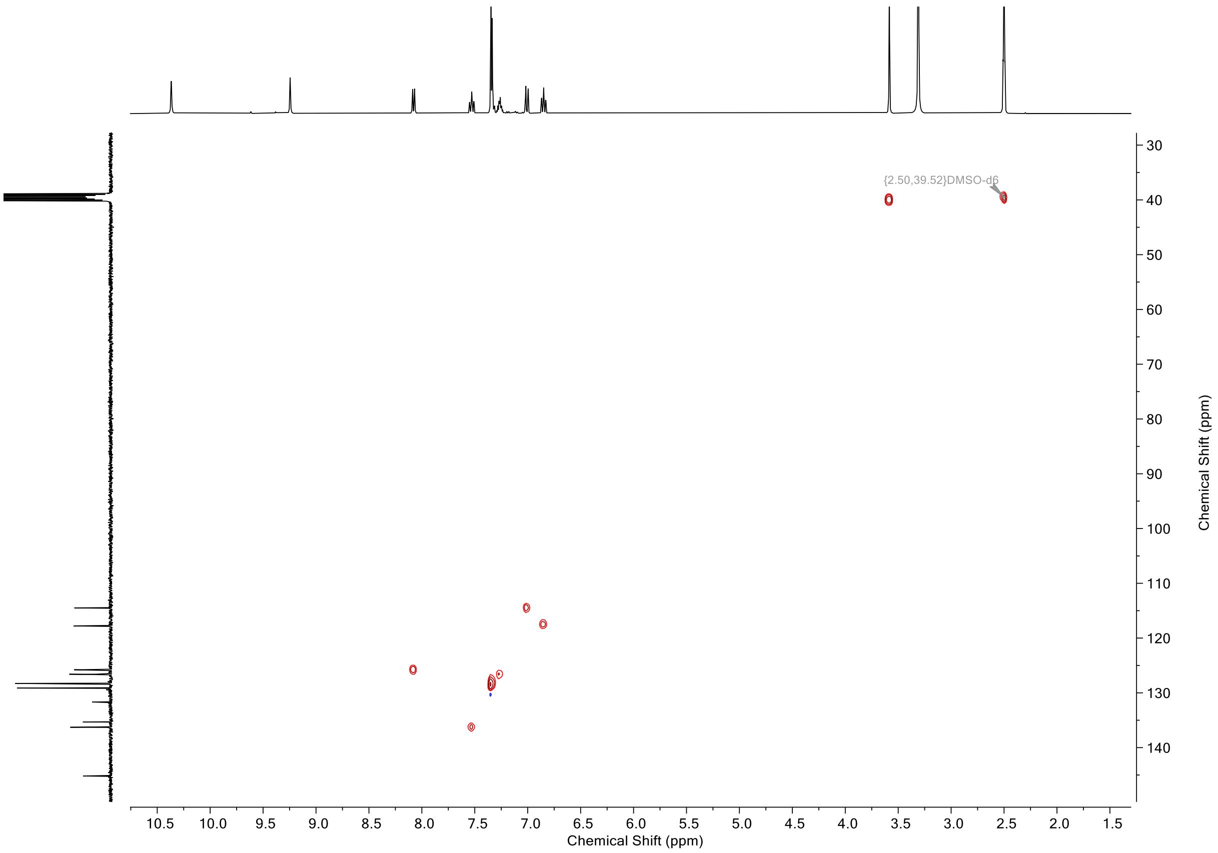

Figure S77: HSQC (DMSO-d_6): *N*'-(2-Nitrophenyl)-2-phenylacetohydrazide (**1n**).

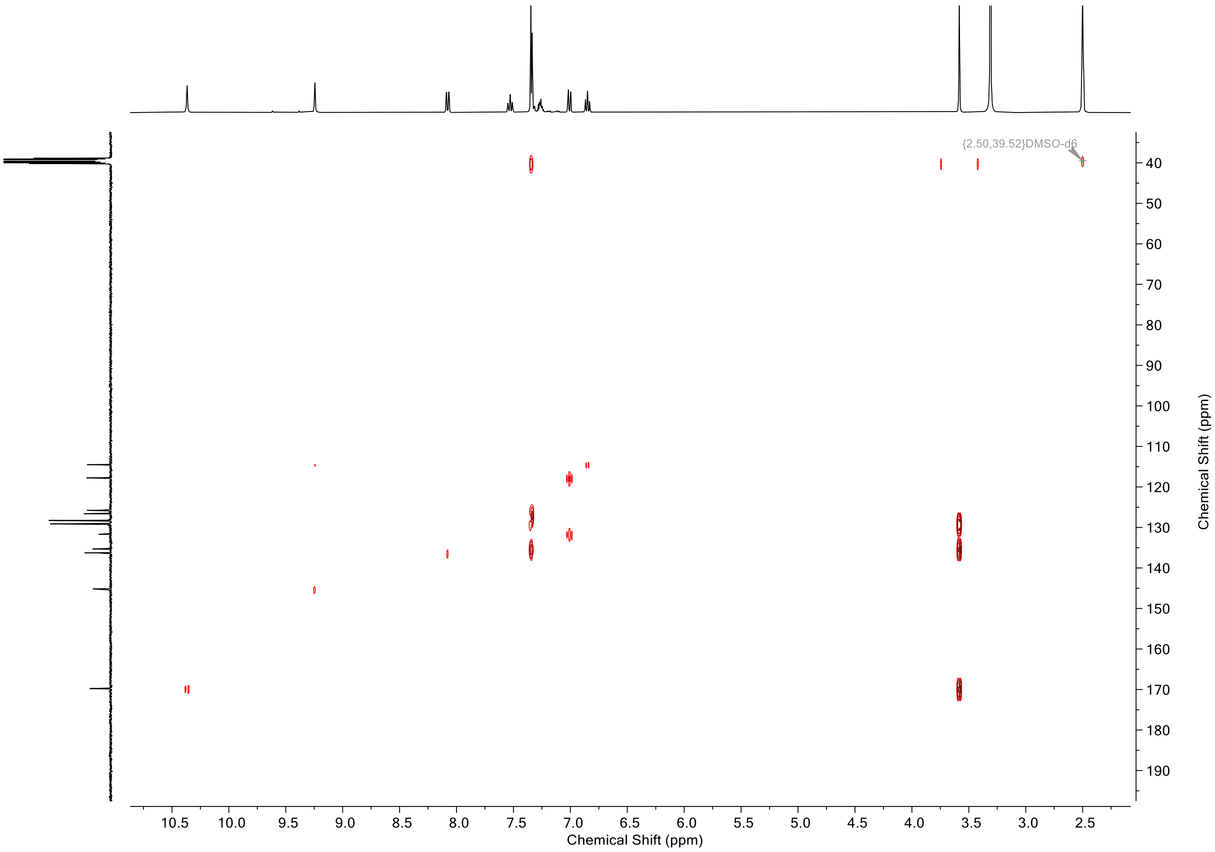

Figure S78: HMBC (DMSO-d_6): *N*'-(2-Nitrophenyl)-2-phenylacetohydrazide (**1n**).

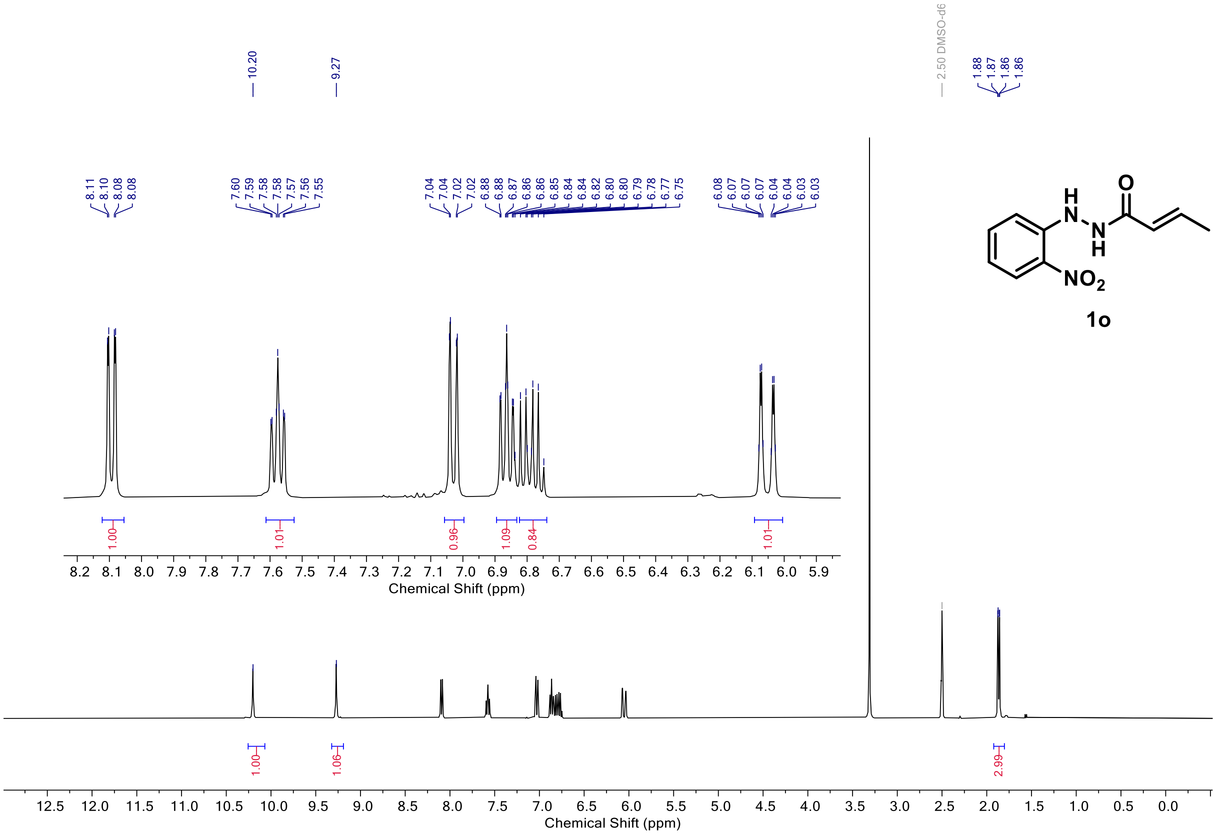

Figure S79: ^{1}H NMR (400 MHz, DMSO-d_6): (*E*)-*N*'-(2-Nitrophenyl)but-2-enehydrazide (**1o**).

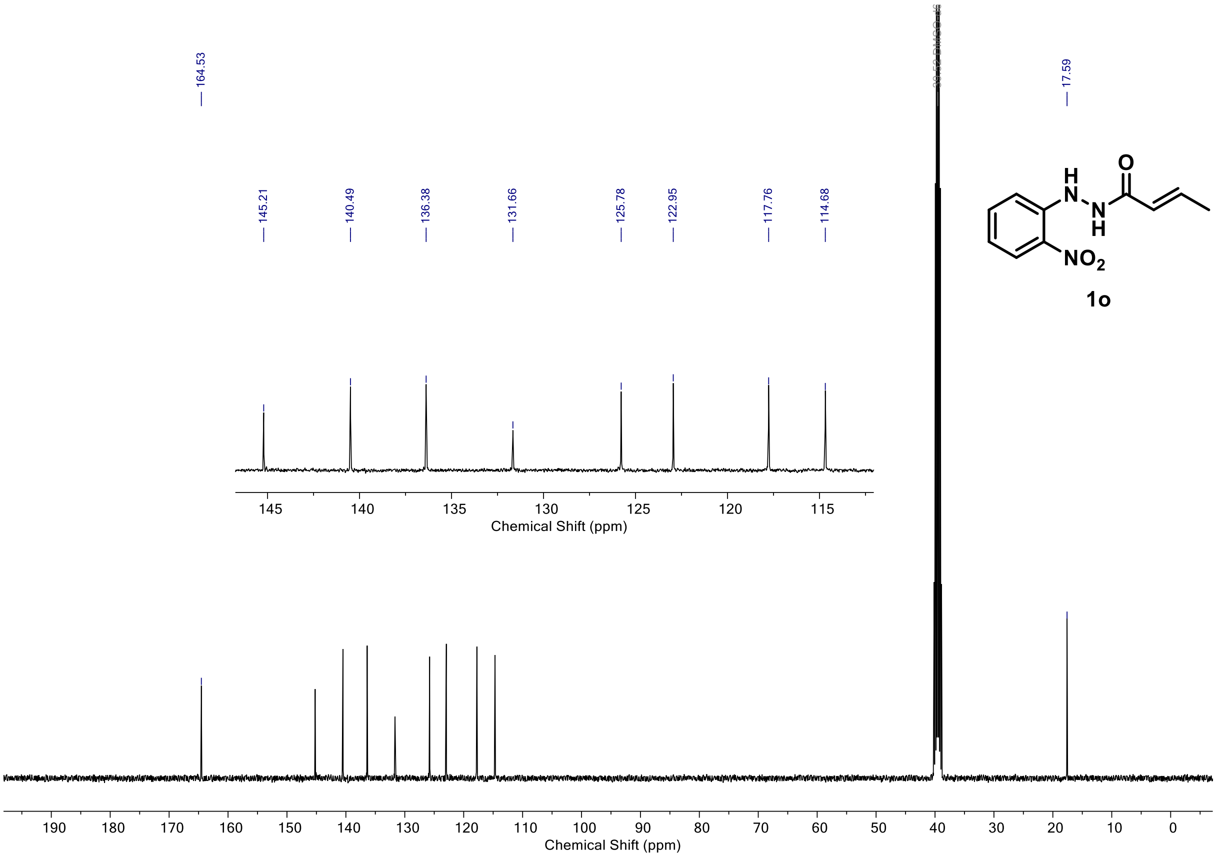

Figure S80: ^{13}C{^{1}H} NMR (101 MHz, DMSO-d_6): (*E*)-*N*'-(2-Nitrophenyl)but-2-enehydrazide (**1o**).

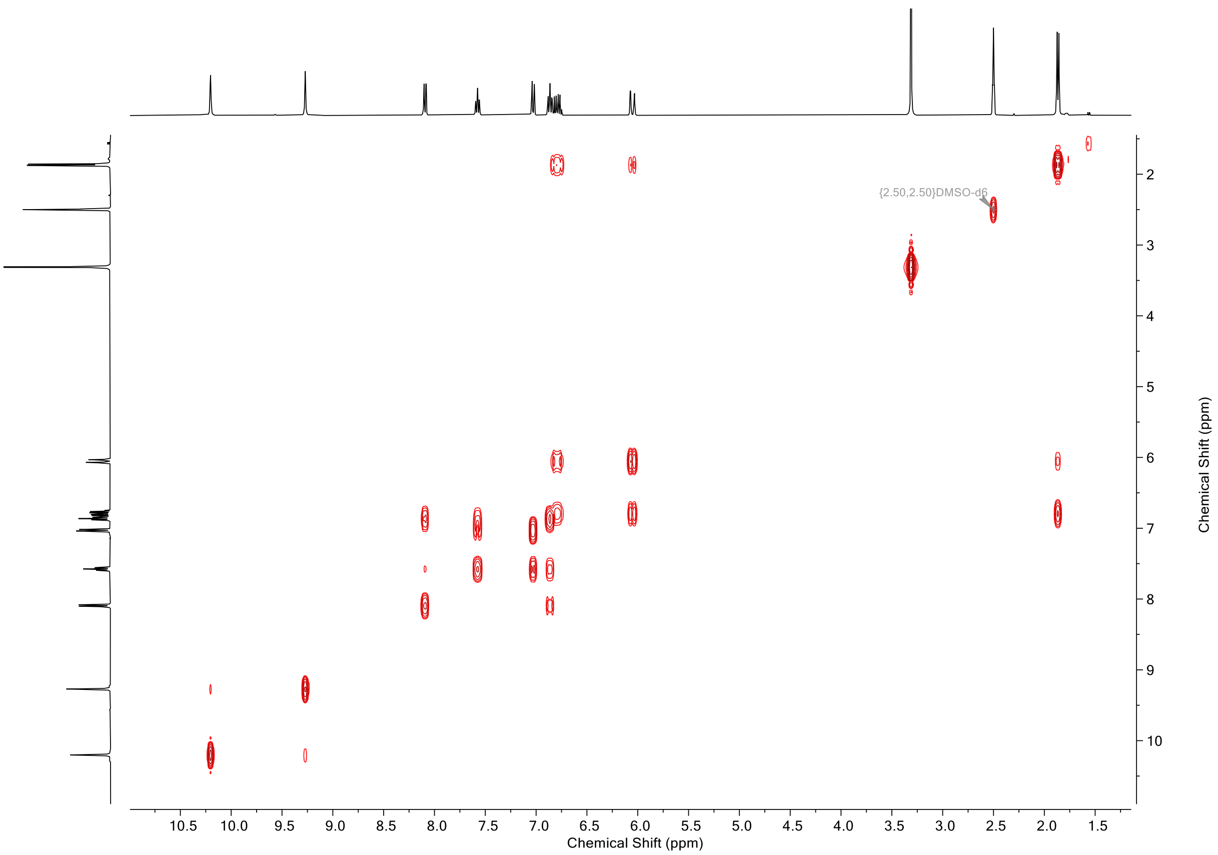

Figure S81: COSY (DMSO-d_6): (*E*)-*N*'-(2-Nitrophenyl)but-2-enehydrazide (**1o**).

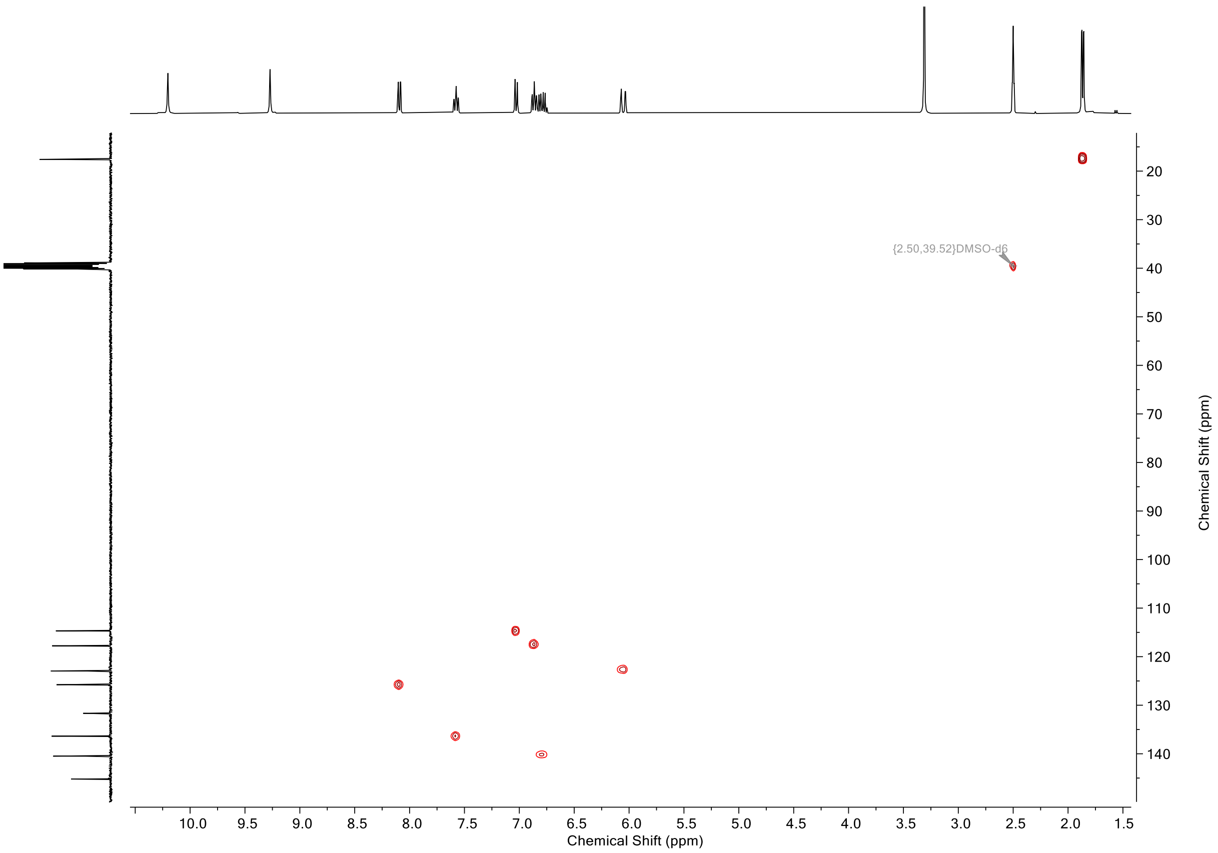

Figure S82: HSQC (DMSO-d_6): (*E*)-*N*'-(2-Nitrophenyl)but-2-enehydrazide (**1o**).

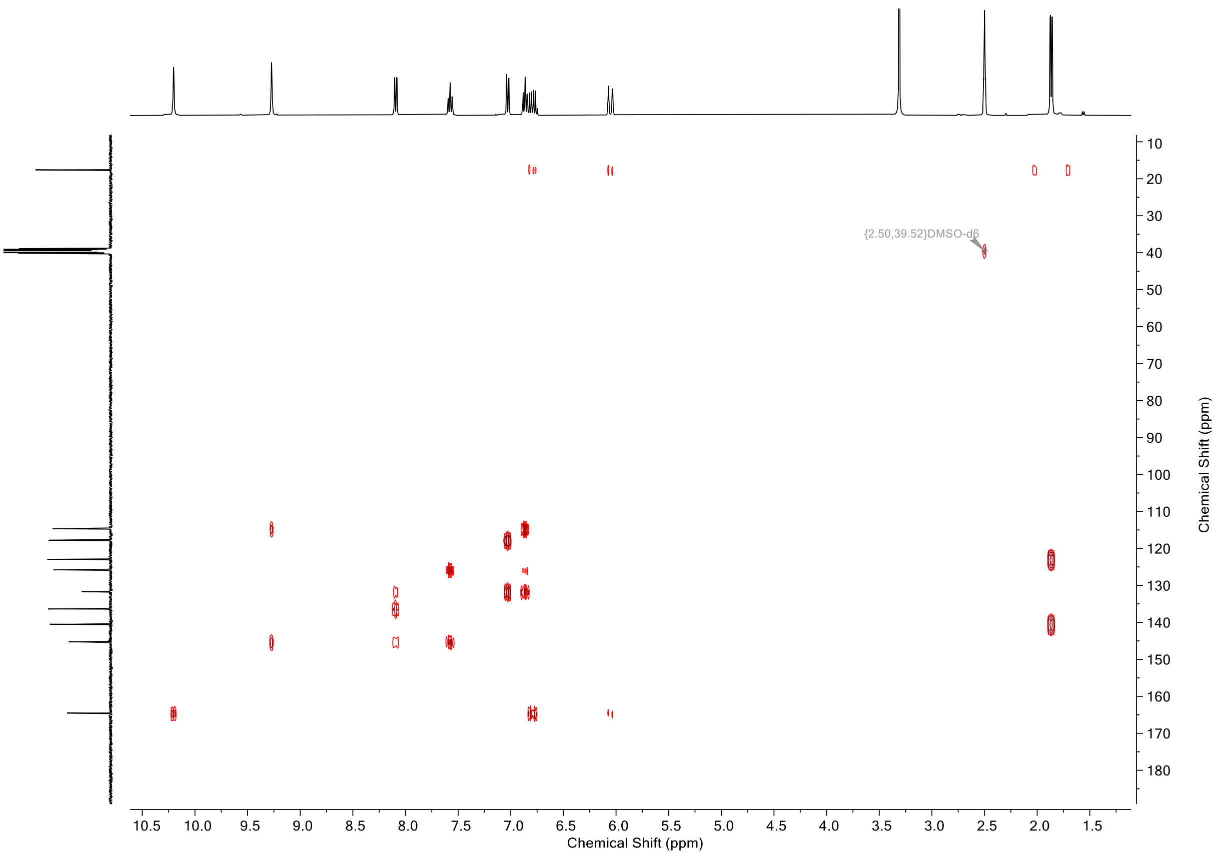

Figure S83: HMBC (DMSO-d_6): (*E*)-*N*'-(2-Nitrophenyl)but-2-enehydrazide (**1o**).

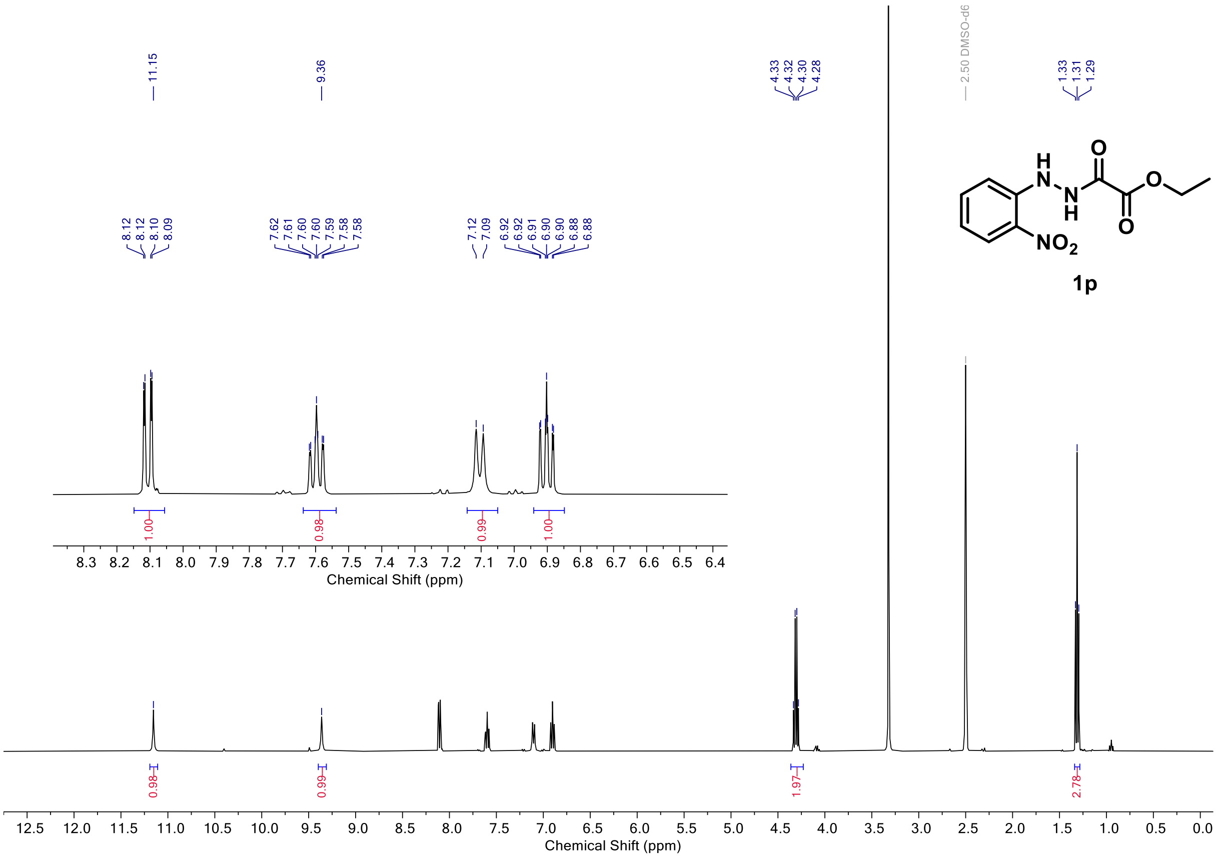

Figure S84: ^{1}H NMR (400 MHz, DMSO-d_6): Ethyl 2-(2-(2-nitrophenyl)hydrazineyl)-2-oxoacetate (**1p**).

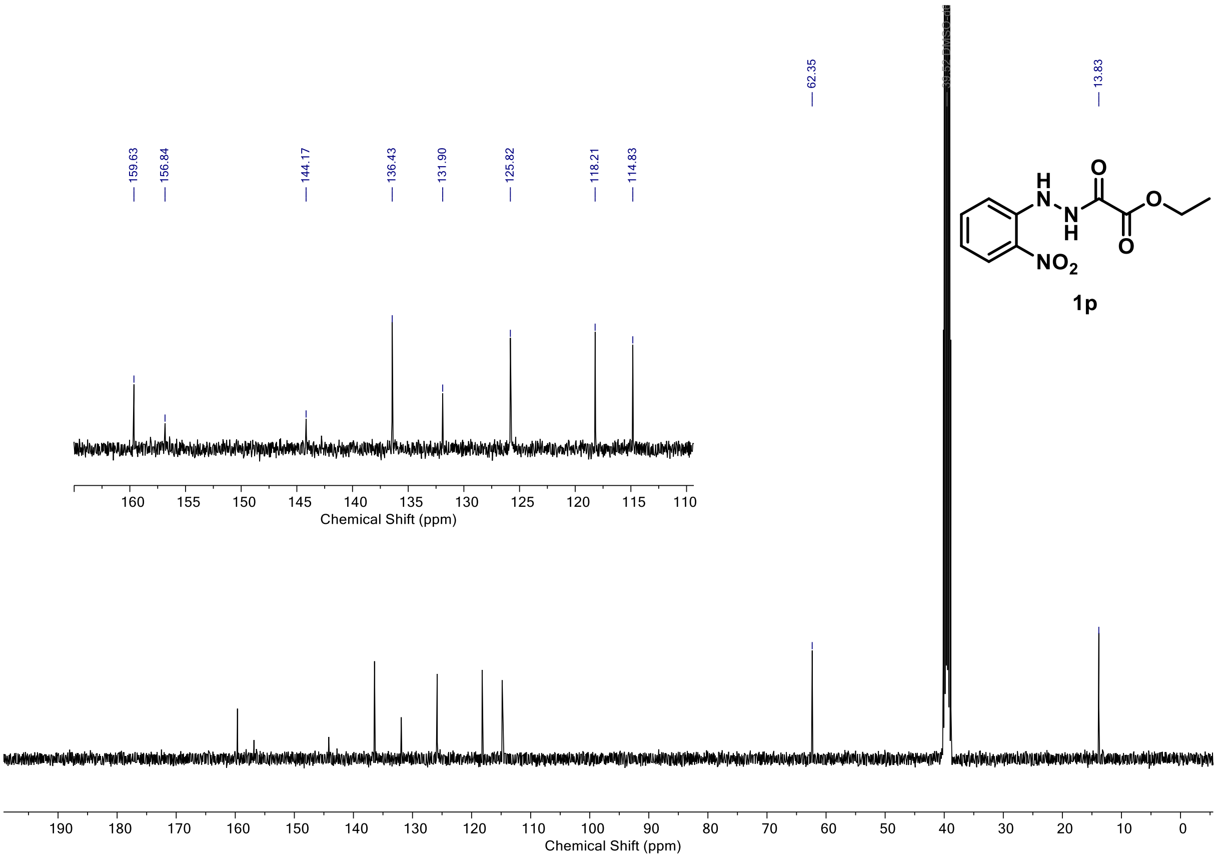

Figure S85: $^{13}C\{^{1}H\}$ NMR (101 MHz, DMSO-d_6): Ethyl 2-(2-(2-nitrophenyl)hydrazineyl)-2-oxoacetate (**1p**).

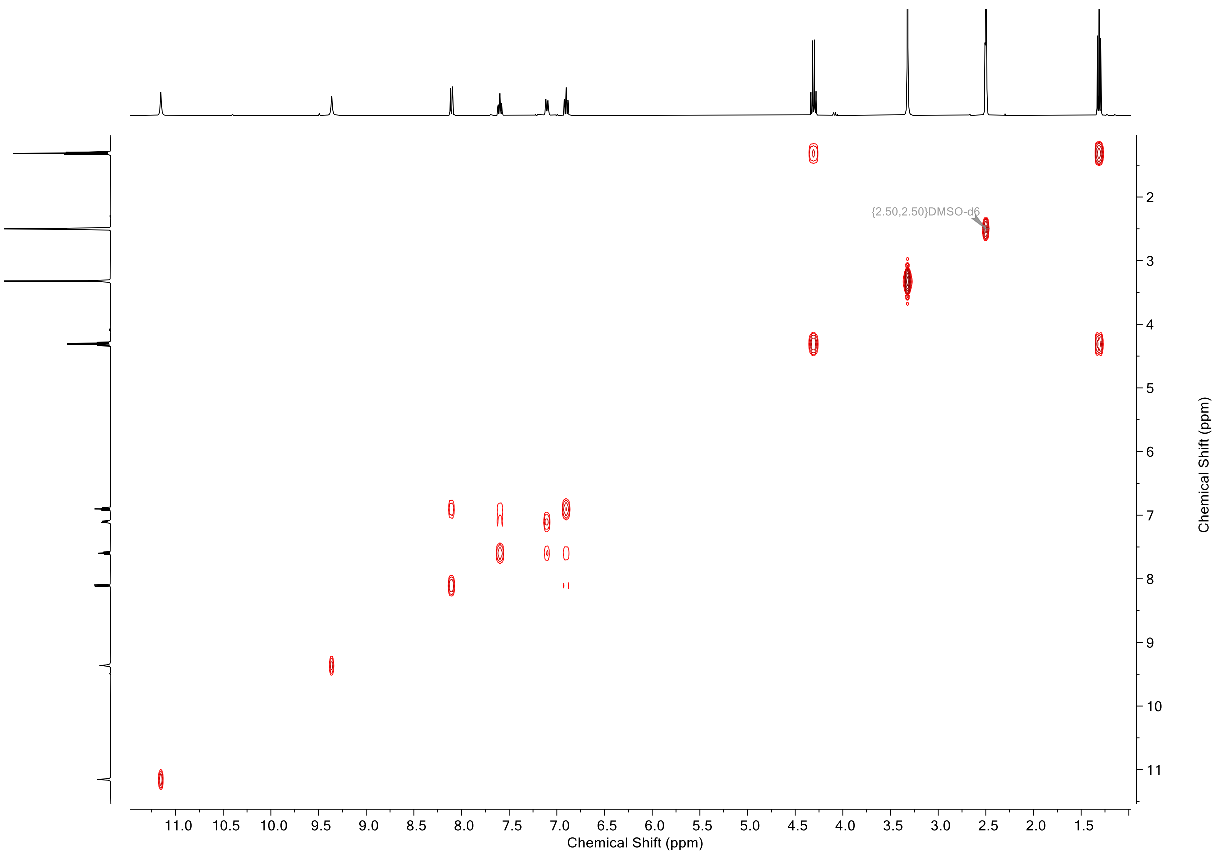

Figure S86: COSY (DMSO-d_6): Ethyl 2-(2-(2-nitrophenyl)hydrazineyl)-2-oxoacetate (**1p**).

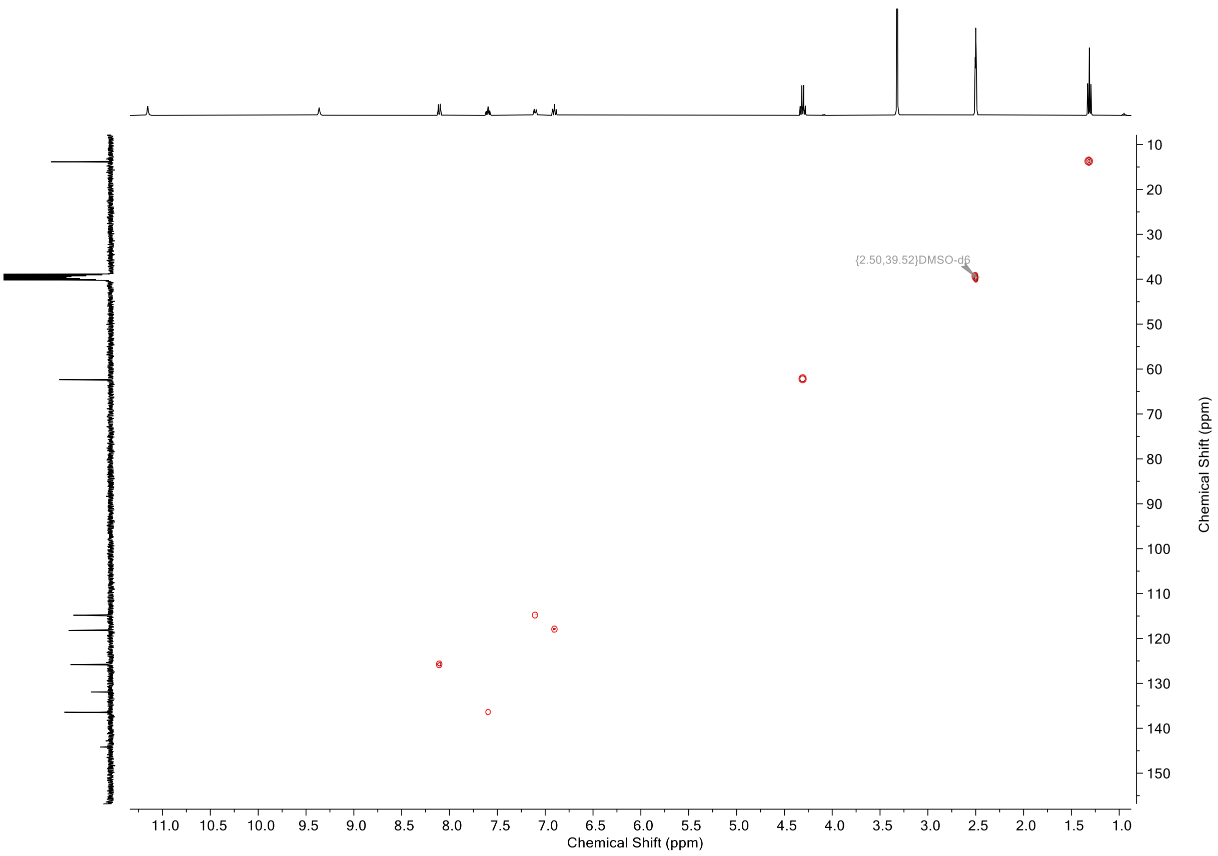

Figure S87: HSQC (DMSO-d_6): Ethyl 2-(2-(2-nitrophenyl)hydrazineyl)-2-oxoacetate (**1p**).

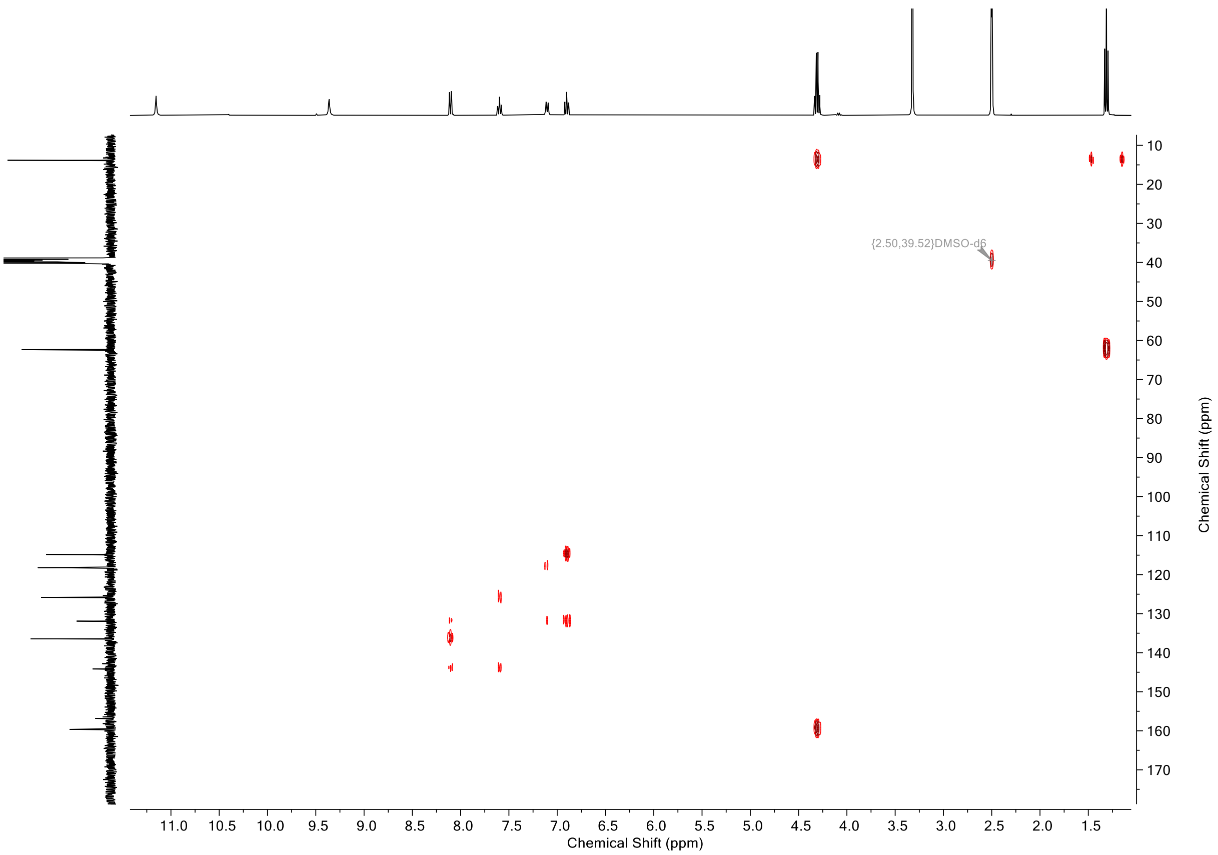

Figure S88: HMBC (DMSO-d_6): Ethyl 2-(2-(2-nitrophenyl)hydrazineyl)-2-oxoacetate (**1p**).

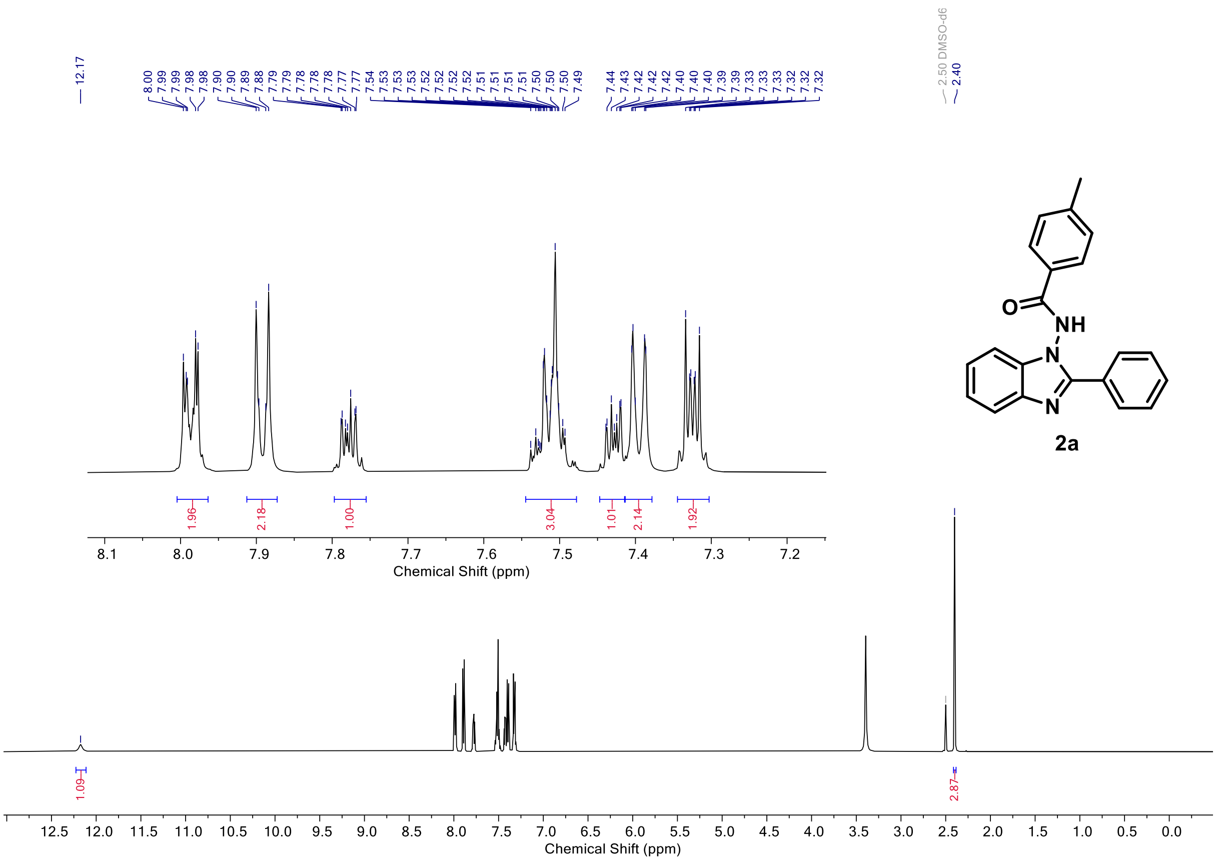

Figure S89: ^{1}H NMR (500 MHz, DMSO-d_6): 1*H*-4-Methyl-*N*-(2-phenyl-benzo[*d*]imidazol-1-yl)benzamide (**2a**).

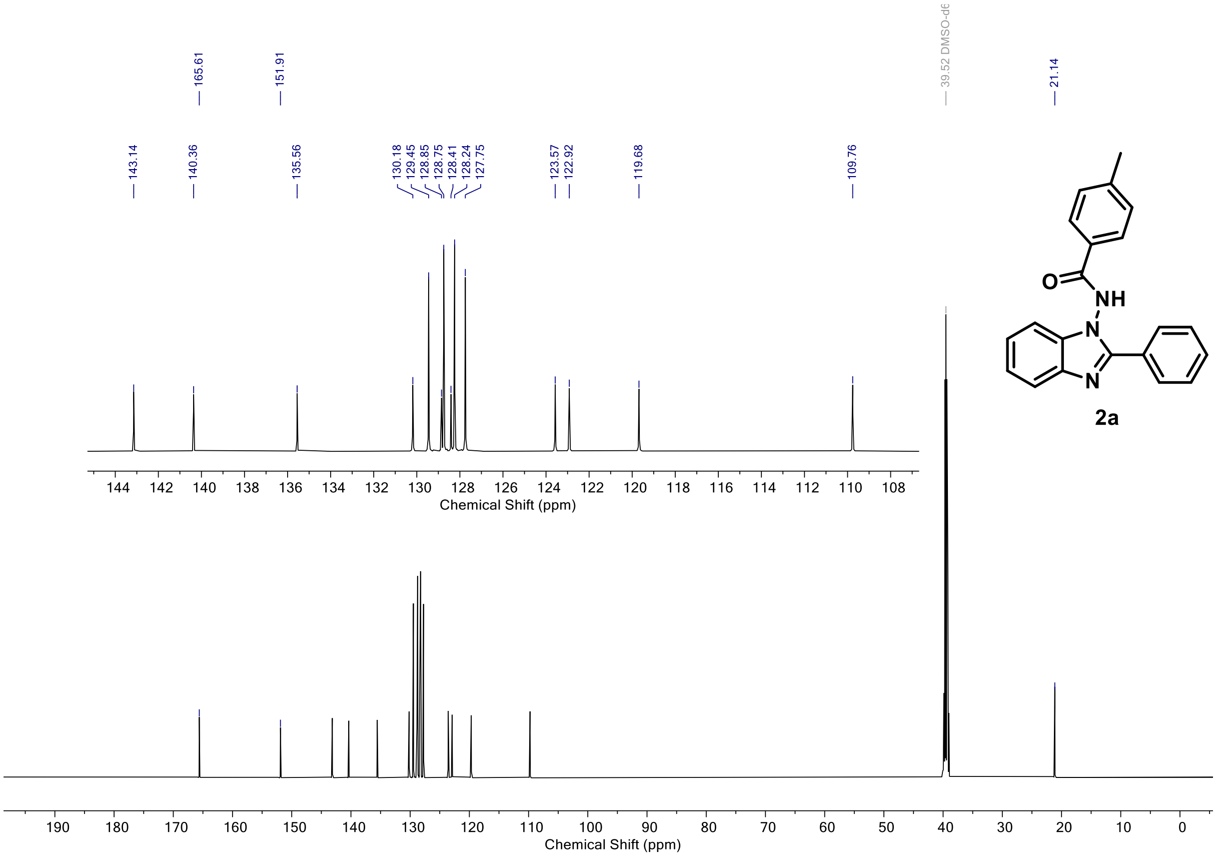

Figure S90: ^{13}C{^{1}H} NMR (126 MHz, DMSO-d_6): 1*H*-4-Methyl-*N*-(2-phenyl-benzo[*d*]imidazol-1-yl)benzamide (**2a**).

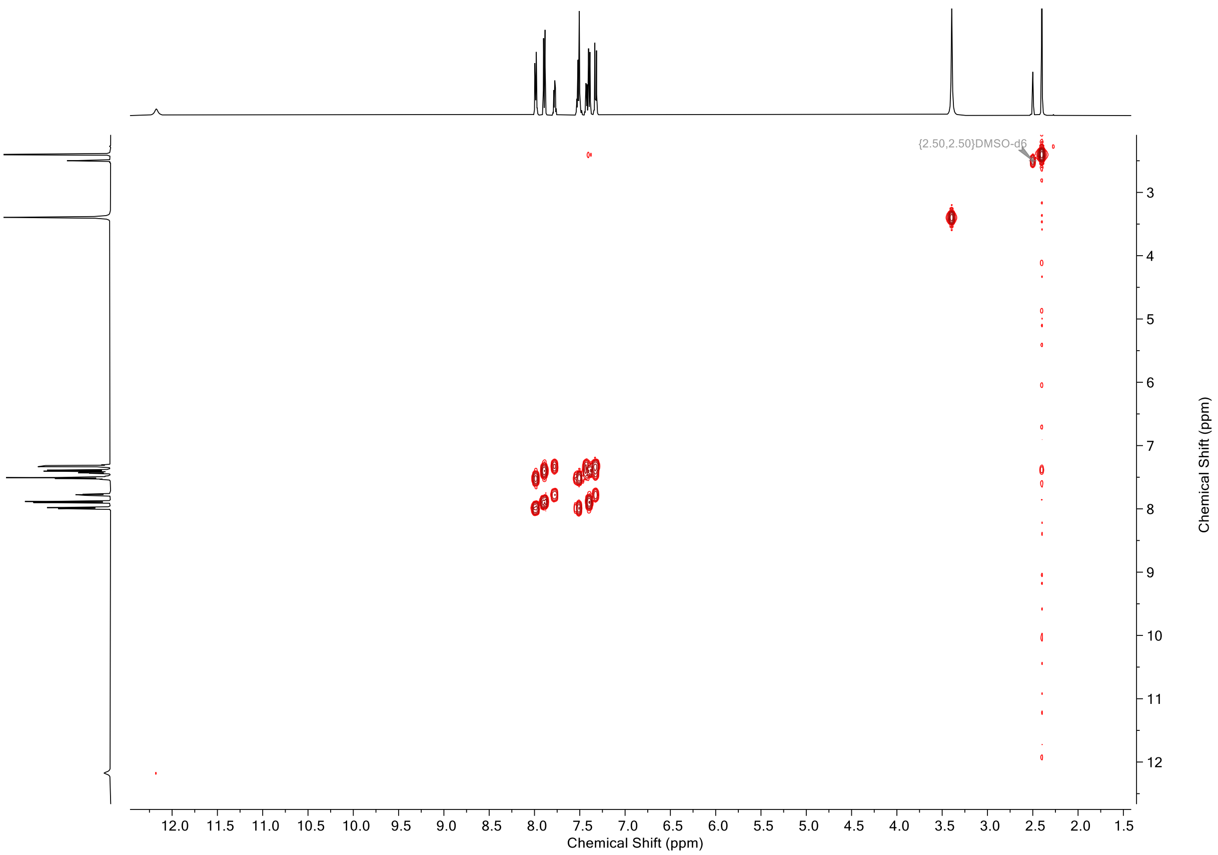

Figure S91: COSY (DMSO-d_6): 1*H*-4-Methyl-*N*-(2-phenyl-benzo[*d*]imidazol-1-yl)benzamide (**2a**).

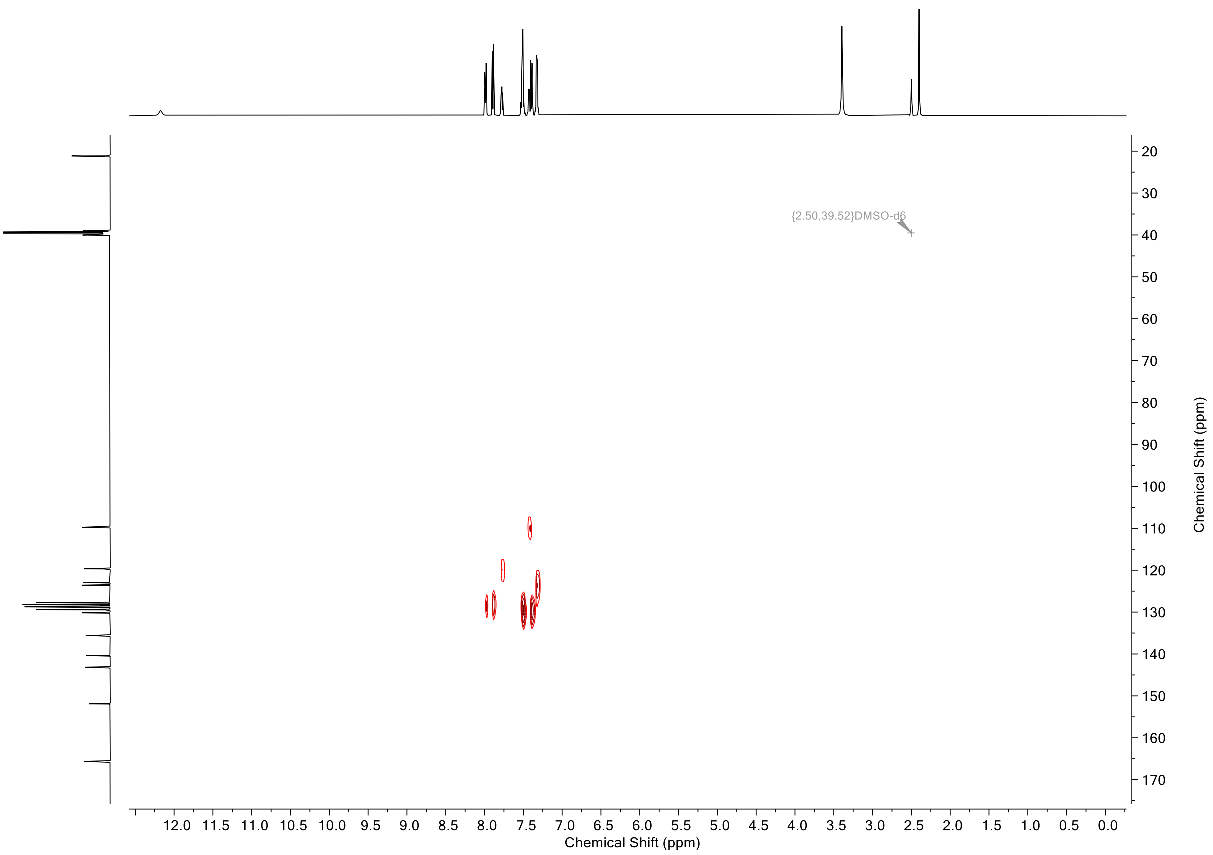

Figure S92: HSQC (DMSO-d_6): 1*H*-4-Methyl-*N*-(2-phenyl-benzo[*d*]imidazol-1-yl)benzamide (**2a**).

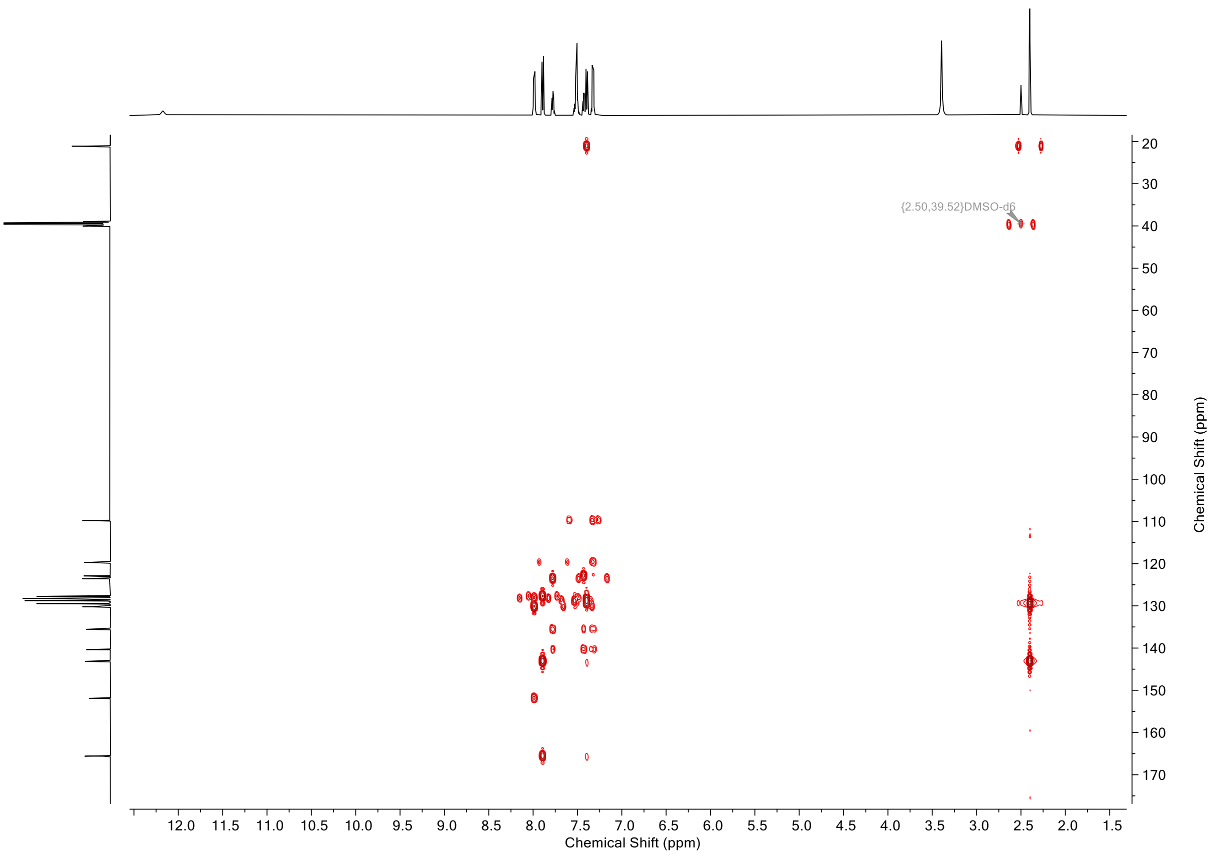

Figure S93: HMBC (DMSO-d_6): 1*H*-4-Methyl-*N*-(2-phenyl-benzo[*d*]imidazol-1-yl)benzamide (**2a**).

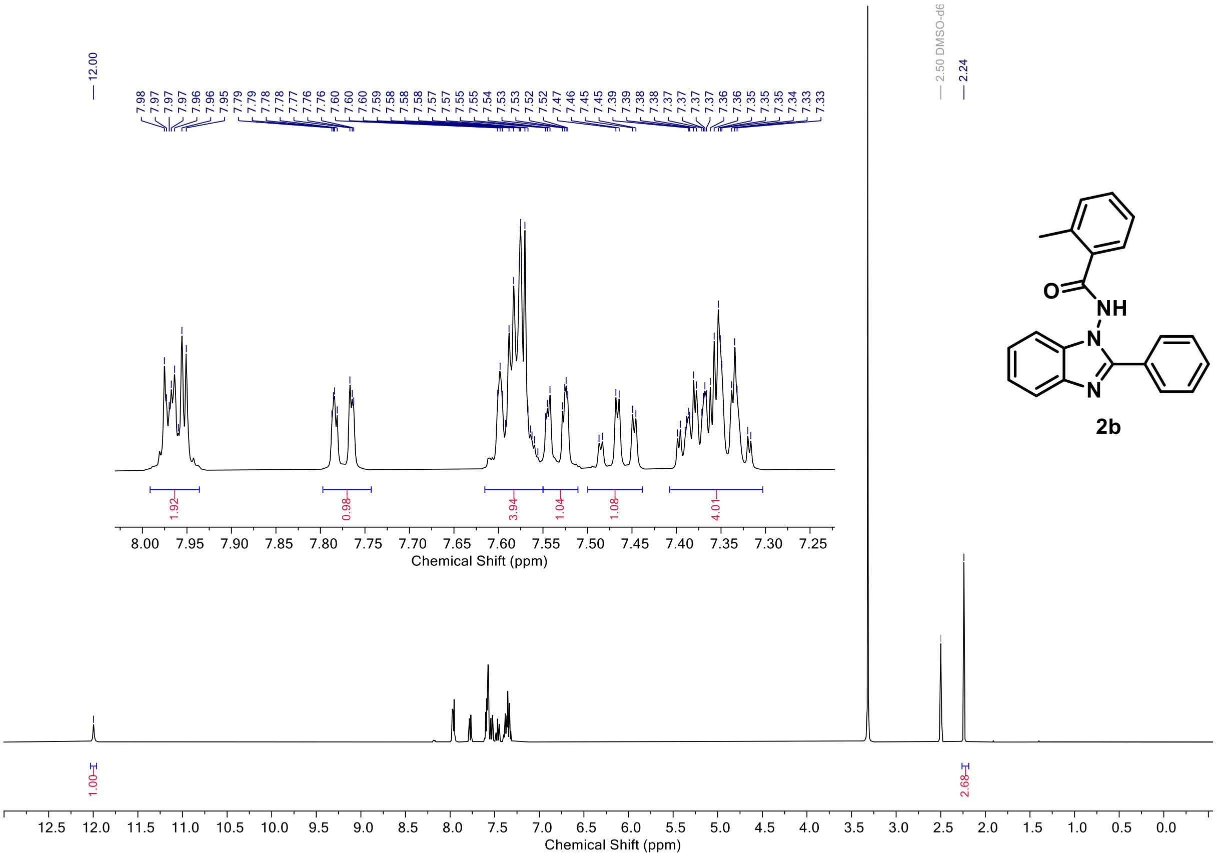

Figure S94: ^{1}H NMR (400 MHz, DMSO-d_6): 1*H*-2-Methyl-*N*-(2-phenyl-benzo[*d*]imidazol-1-yl)benzamide (**2b**).

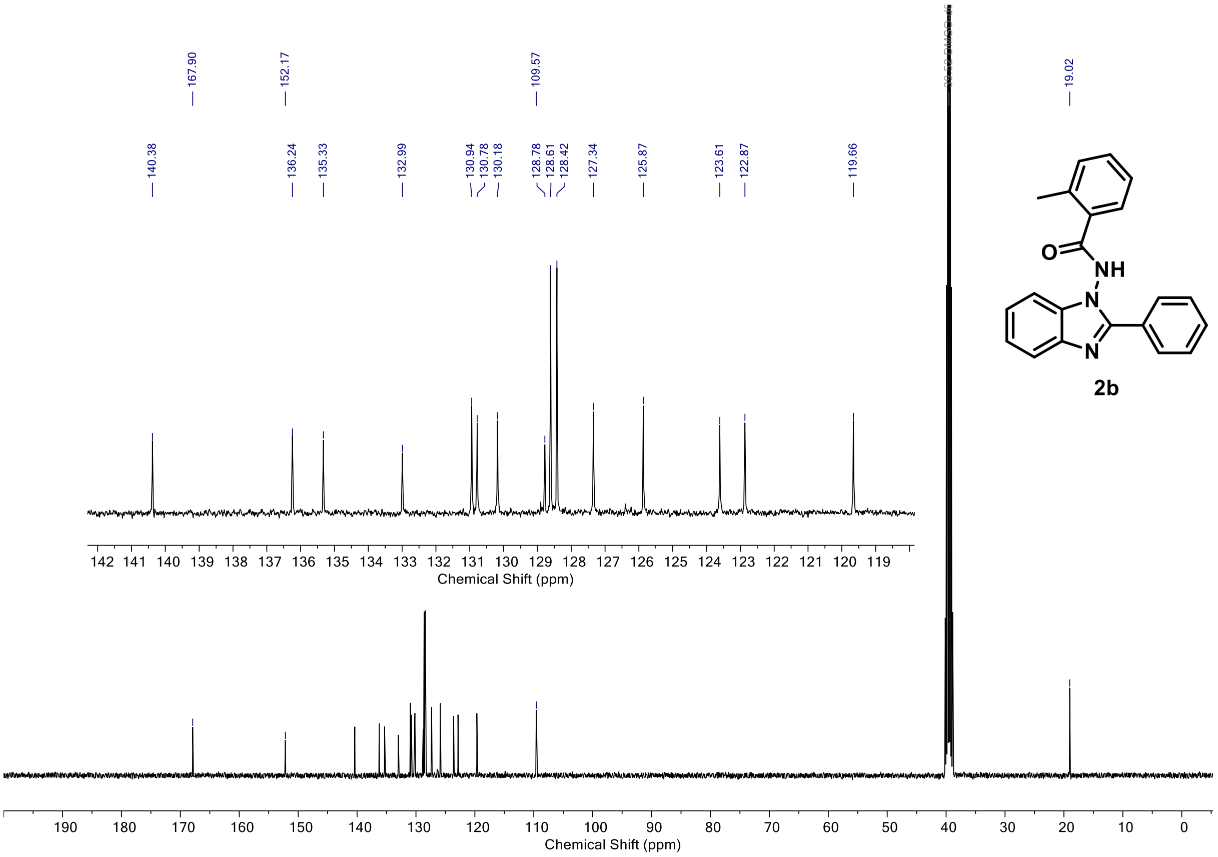

Figure S95: ^{13}C{^{1}H} NMR (101 MHz, DMSO-d_6): 1*H*-2-Methyl-*N*-(2-phenyl-benzo[*d*]imidazol-1-yl)benzamide (**2b**).

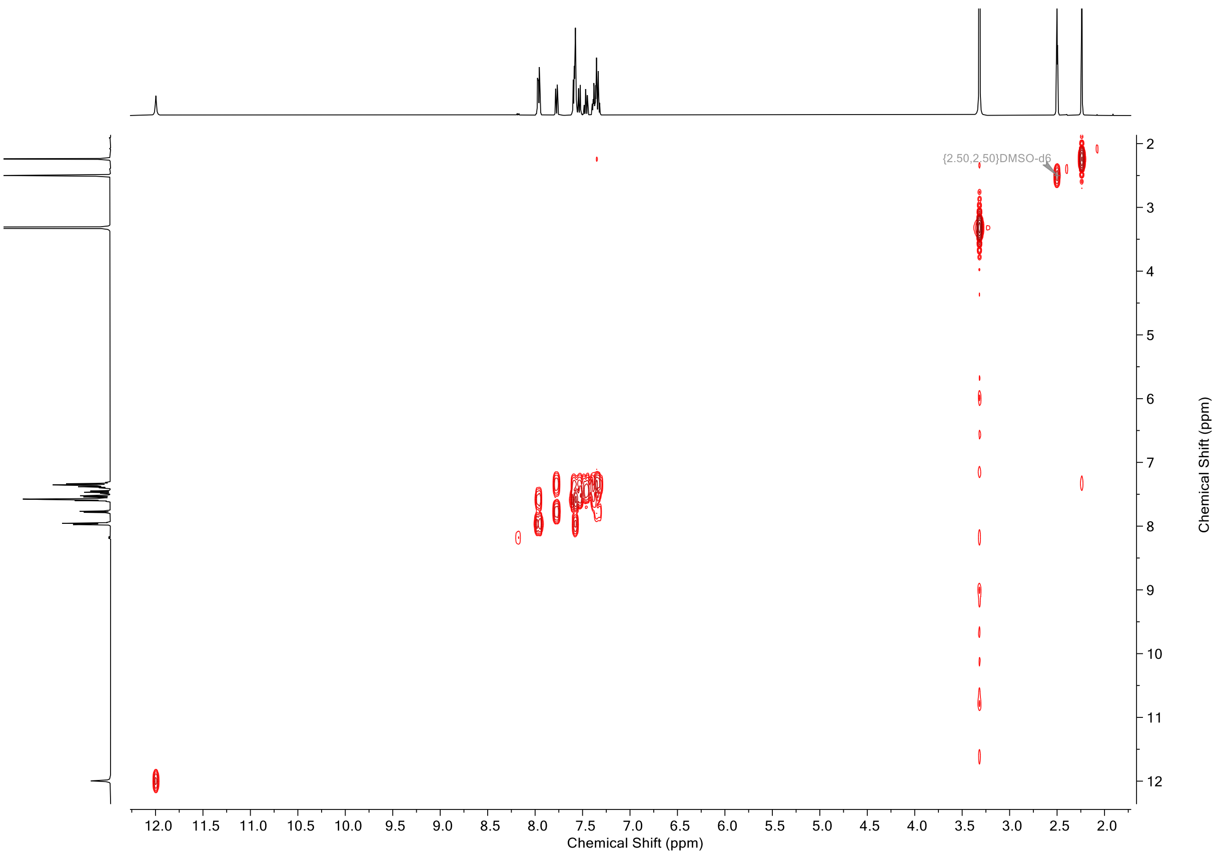

Figure S96: COSY (DMSO-*d*$_6$): 1*H*-2-Methyl-*N*-(2-phenyl-benzo[*d*]imidazol-1-yl)benzamide (**2b**).

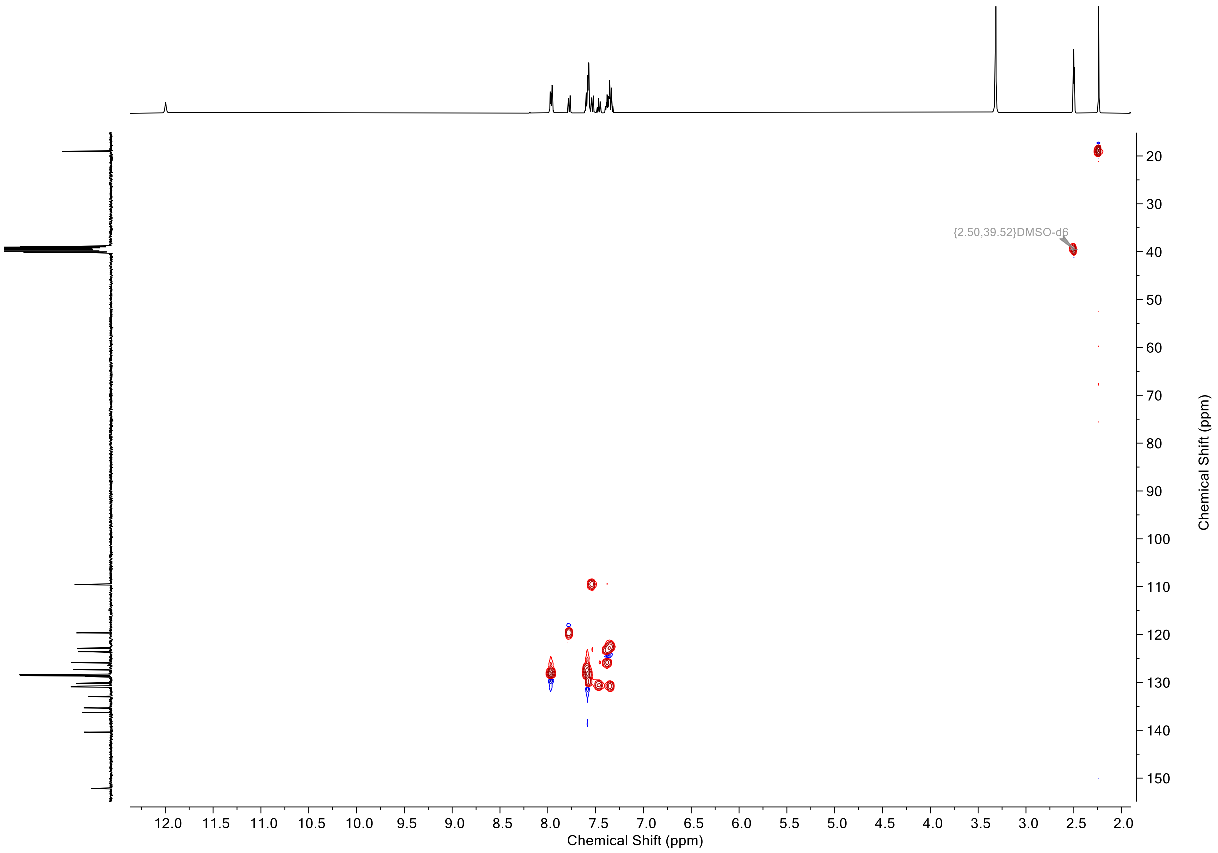

Figure S97: HSQC (DMSO-*d*$_6$): 1*H*-2-Methyl-*N*-(2-phenyl-benzo[*d*]imidazol-1-yl)benzamide (**2b**).

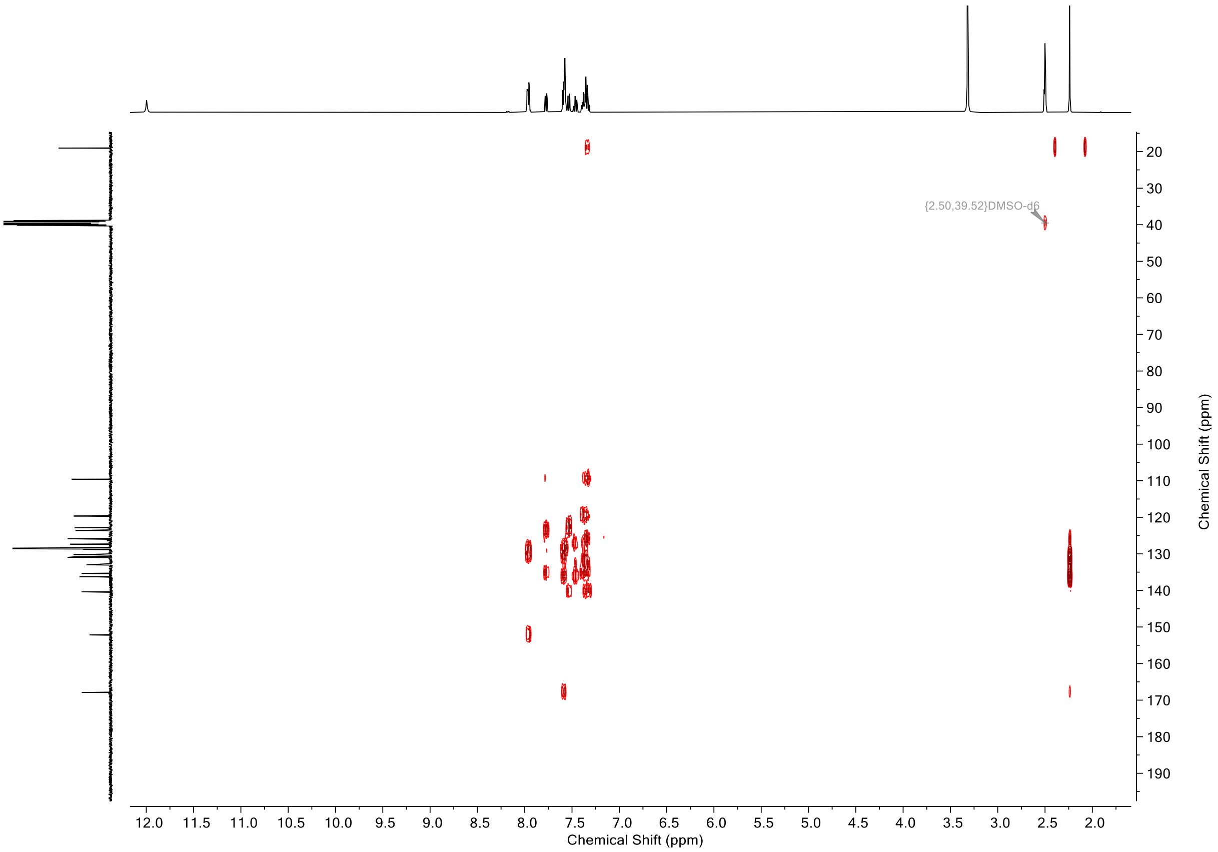

Figure S98: HMBC (DMSO-d_6): 1*H*-2-Methyl-*N*-(2-phenyl-benzo[*d*]imidazol-1-yl)benzamide (**2b**).

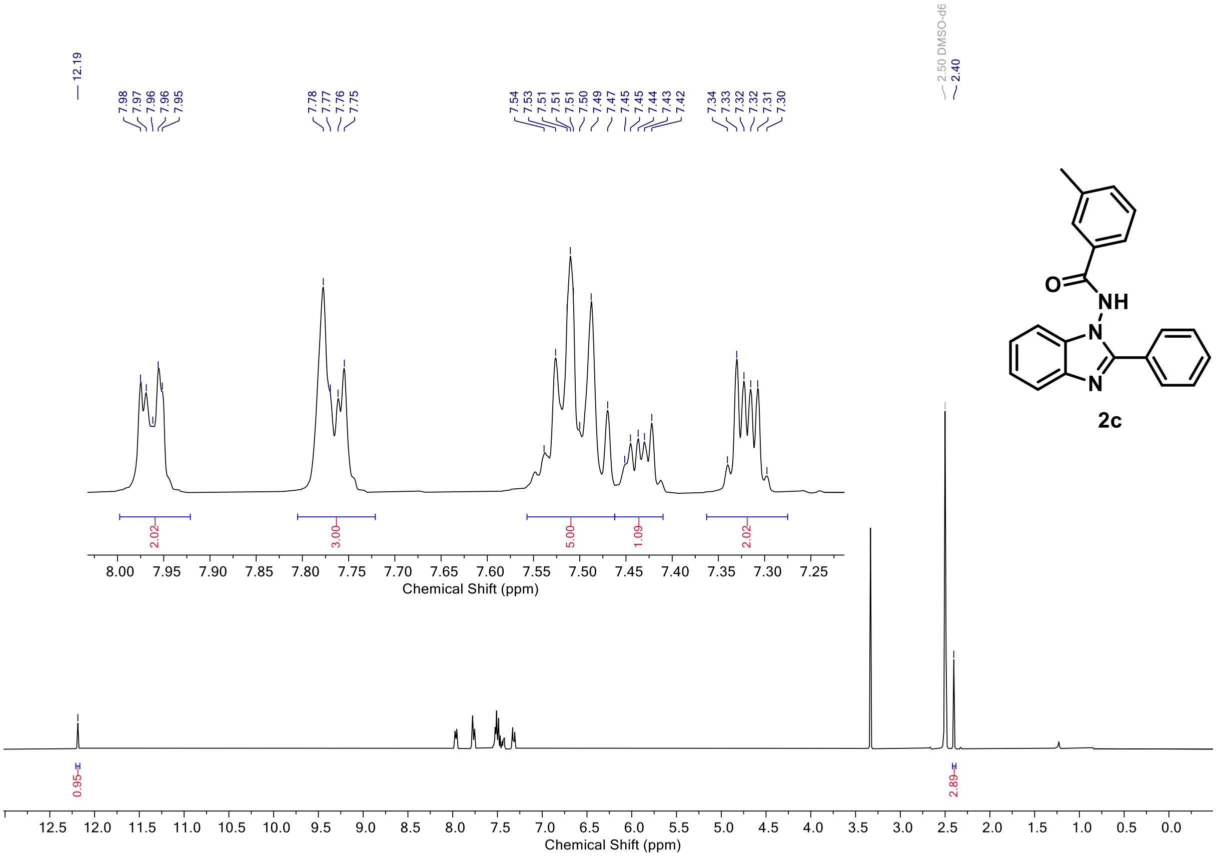

Figure S99: ^{1}H NMR (400 MHz, DMSO-d_6): 1*H*-3-Methyl-*N*-(2-phenyl-benzo[*d*]imidazol-1-yl)benzamide (**2c**).

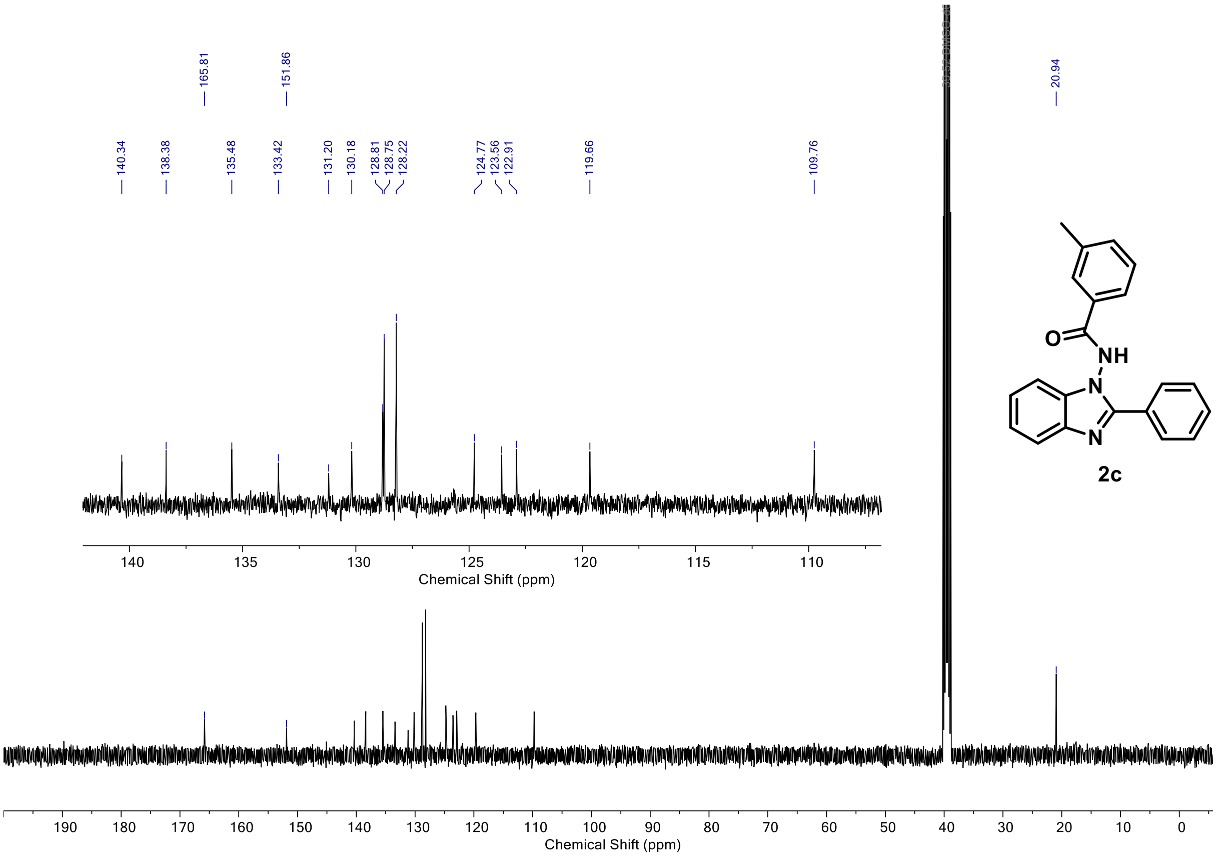

Figure S100: ^{13}C{^{1}H} NMR (101 MHz, DMSO-d_6): 1*H*-3-Methyl-*N*-(2-phenyl-benzo[*d*]imidazol-1-yl)benzamide (**2c**).

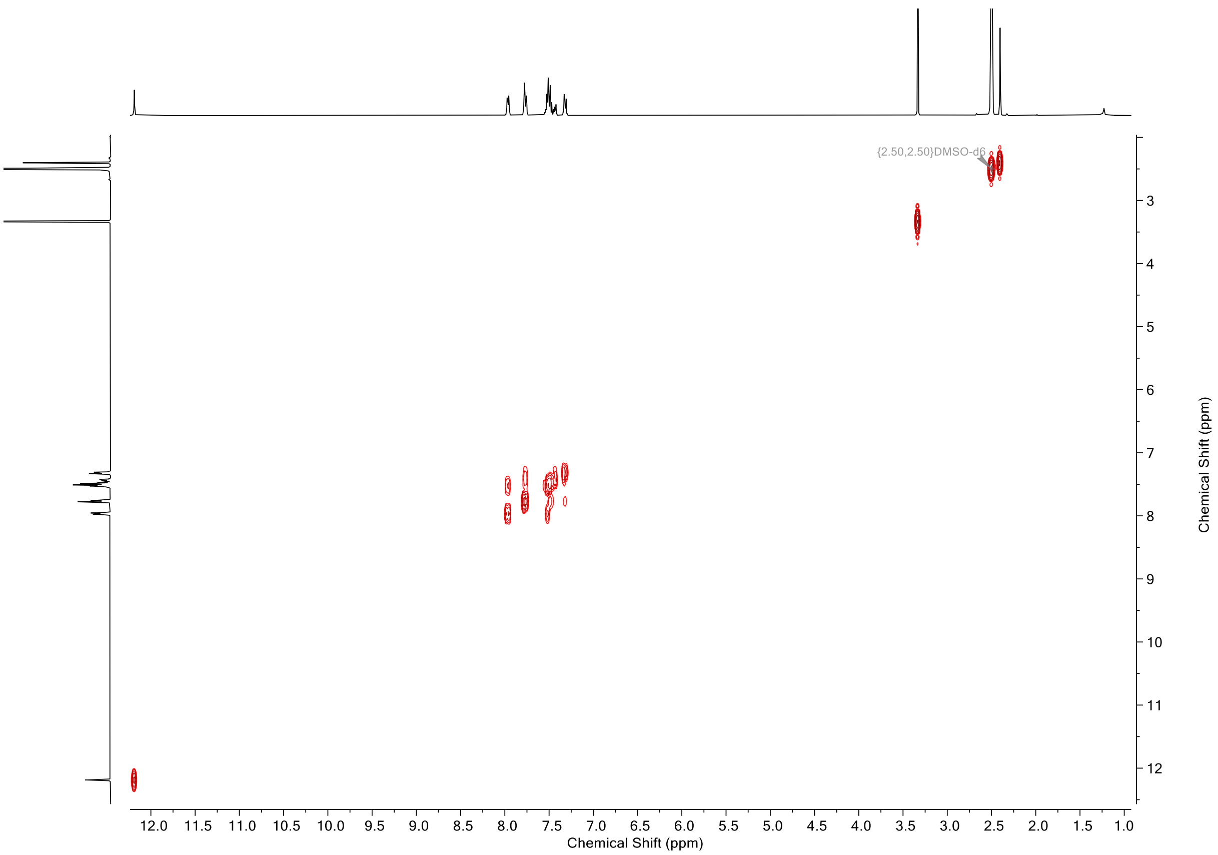

Figure S101: COSY (DMSO-d_6): 1*H*-3-Methyl-*N*-(2-phenyl-benzo[*d*]imidazol-1-yl)benzamide (**2c**).

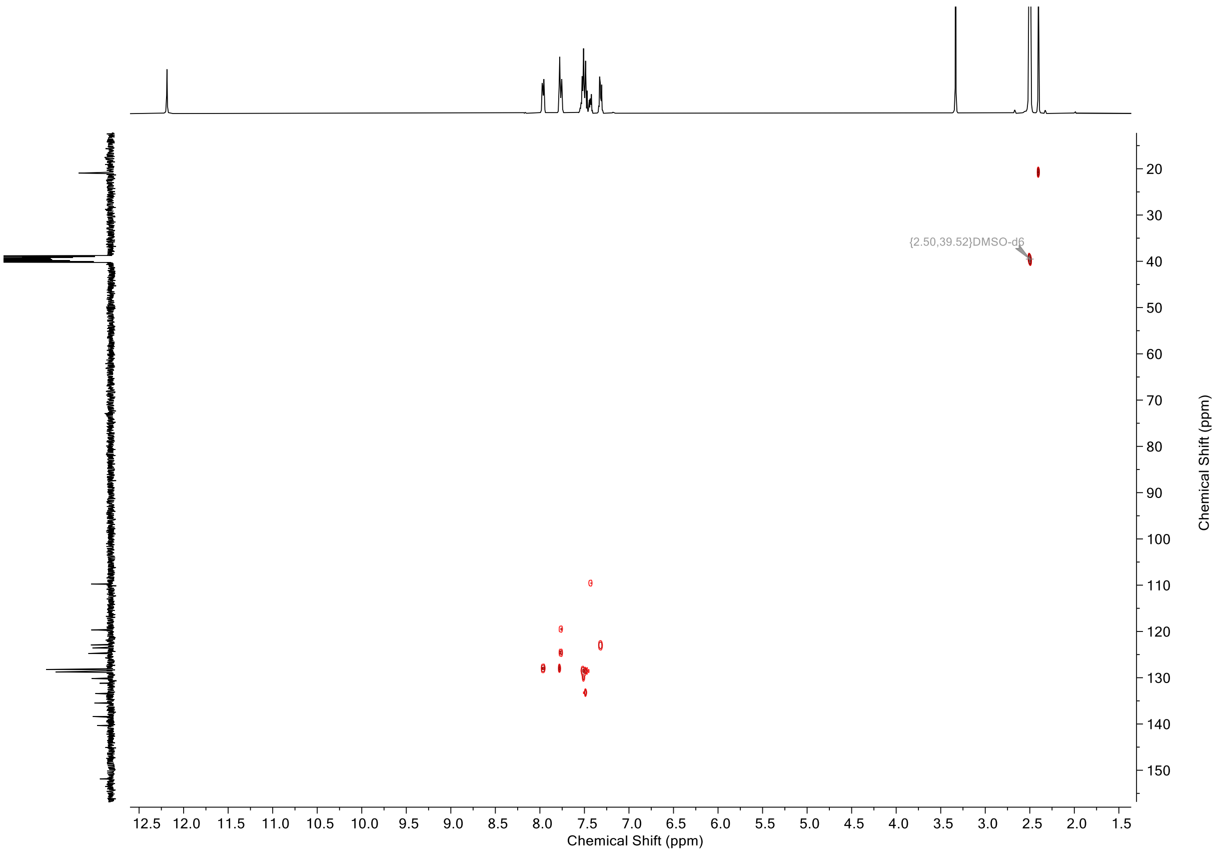

Figure S102: HSQC (DMSO-d_6): 1*H*-3-Methyl-*N*-(2-phenyl-benzo[*d*]imidazol-1-yl)benzamide (**2c**).

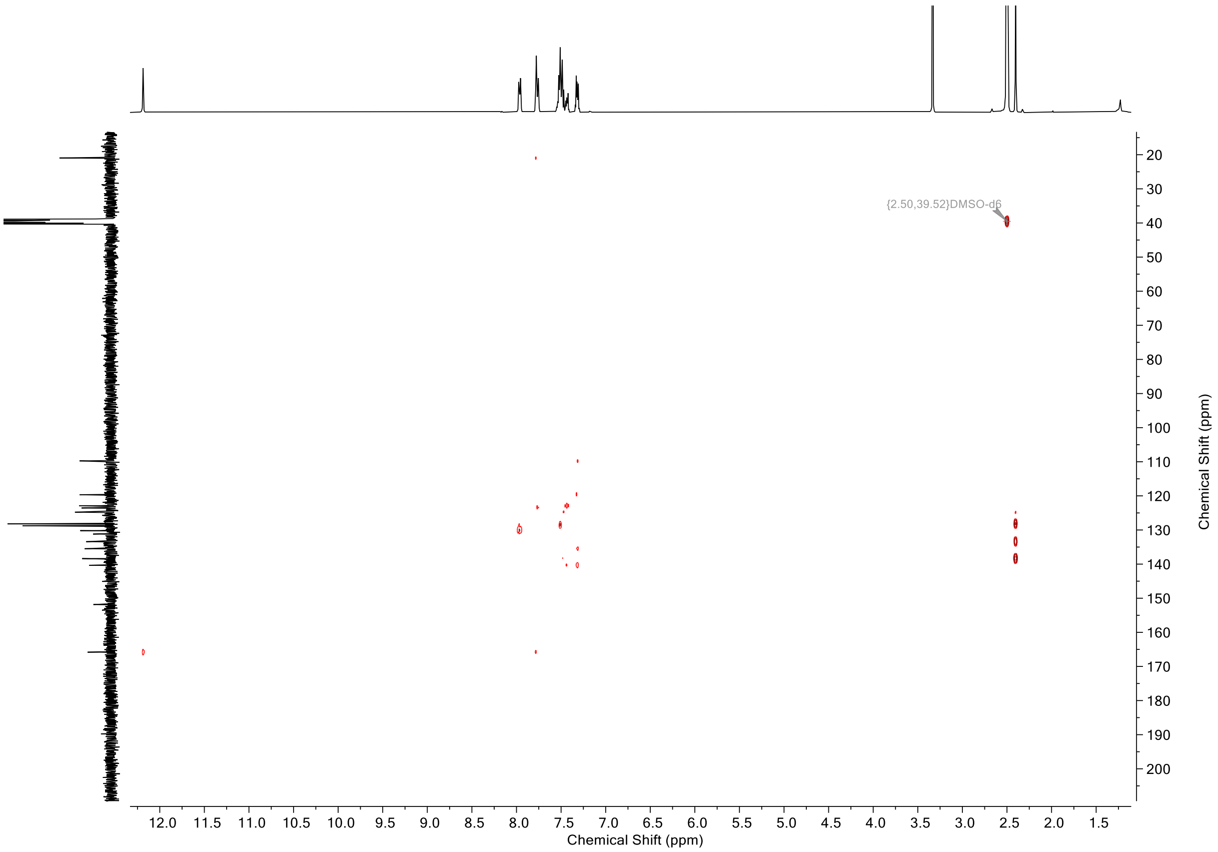

Figure S103: HMBC (DMSO-d_6): 1*H*-3-Methyl-*N*-(2-phenyl-benzo[*d*]imidazol-1-yl)benzamide (**2c**).

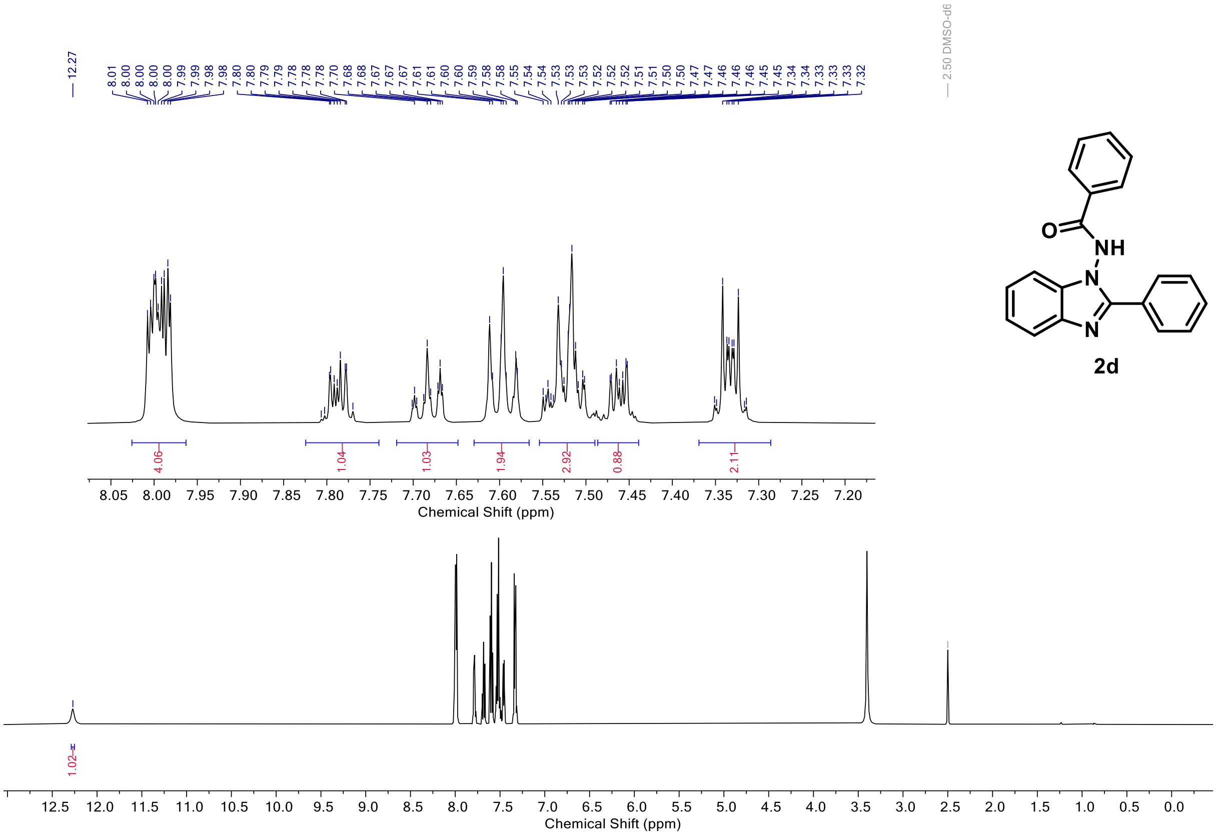

Figure S104: ^{1}H NMR (500 MHz, DMSO-d_6): 1*H*-*N*-(2-Phenyl-benzo[*d*]imidazol-1-yl)benzamide (**2d**).

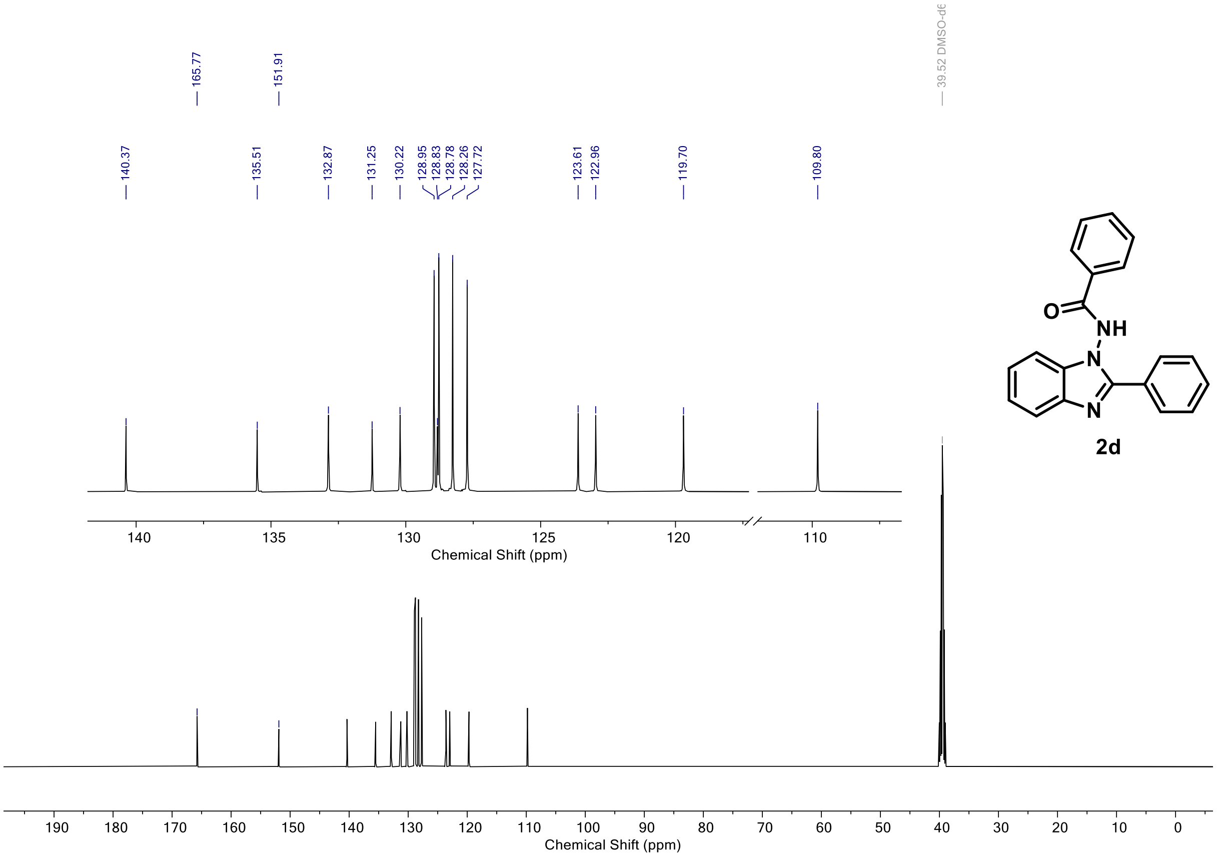

Figure S105: $^{13}C\{^{1}H\}$ NMR (126 MHz, DMSO-d_6): 1*H*-*N*-(2-Phenyl-benzo[*d*]imidazol-1-yl)benzamide (**2d**).

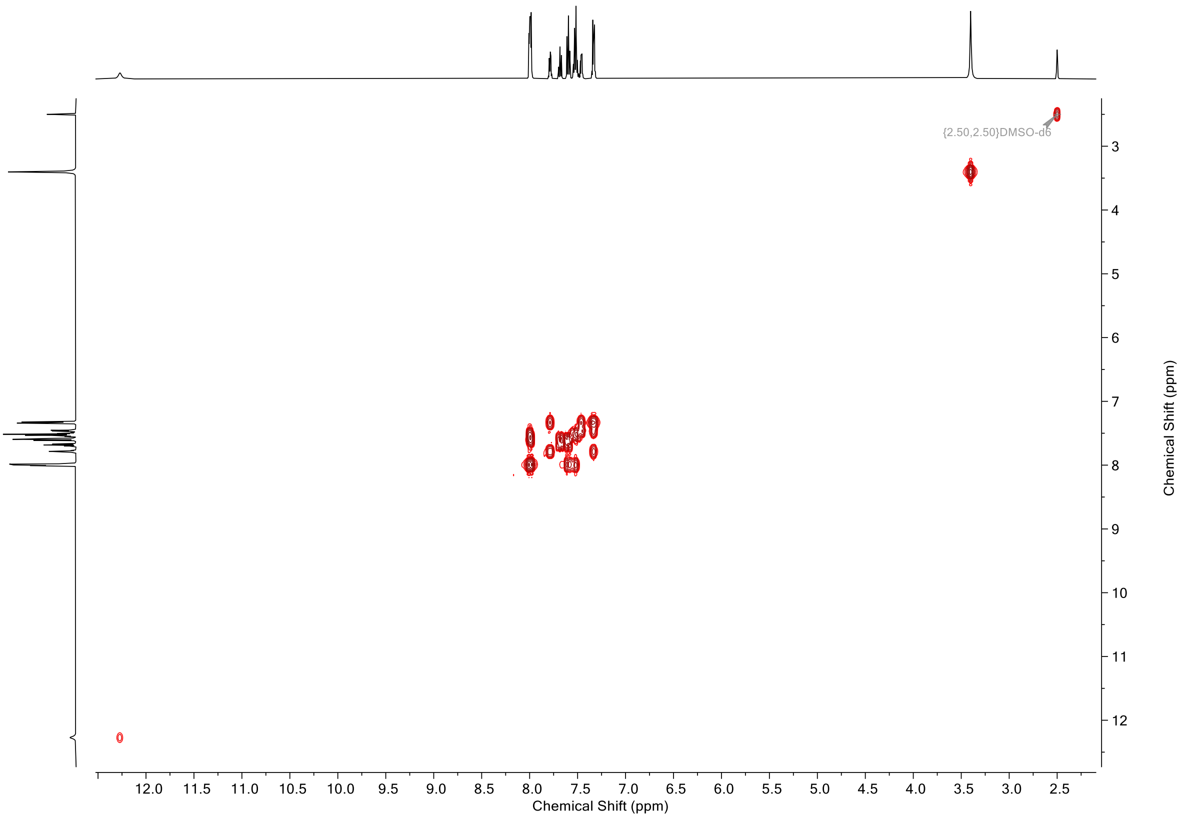

Figure S106: COSY (DMSO-d_6): 1*H*-*N*-(2-Phenyl-benzo[*d*]imidazol-1-yl)benzamide (**2d**).

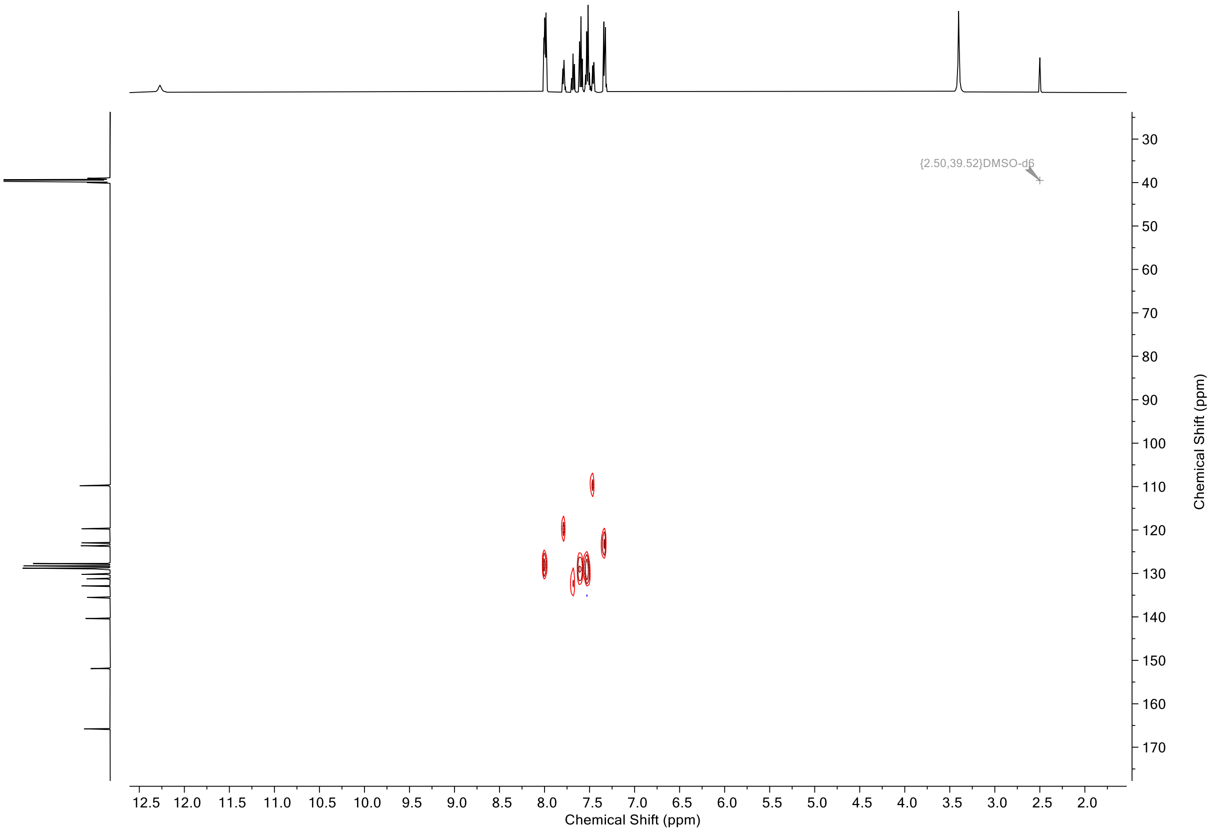

Figure S107: HSQC (DMSO-d_6): 1*H*-*N*-(2-Phenyl-benzo[*d*]imidazol-1-yl)benzamide (**2d**).

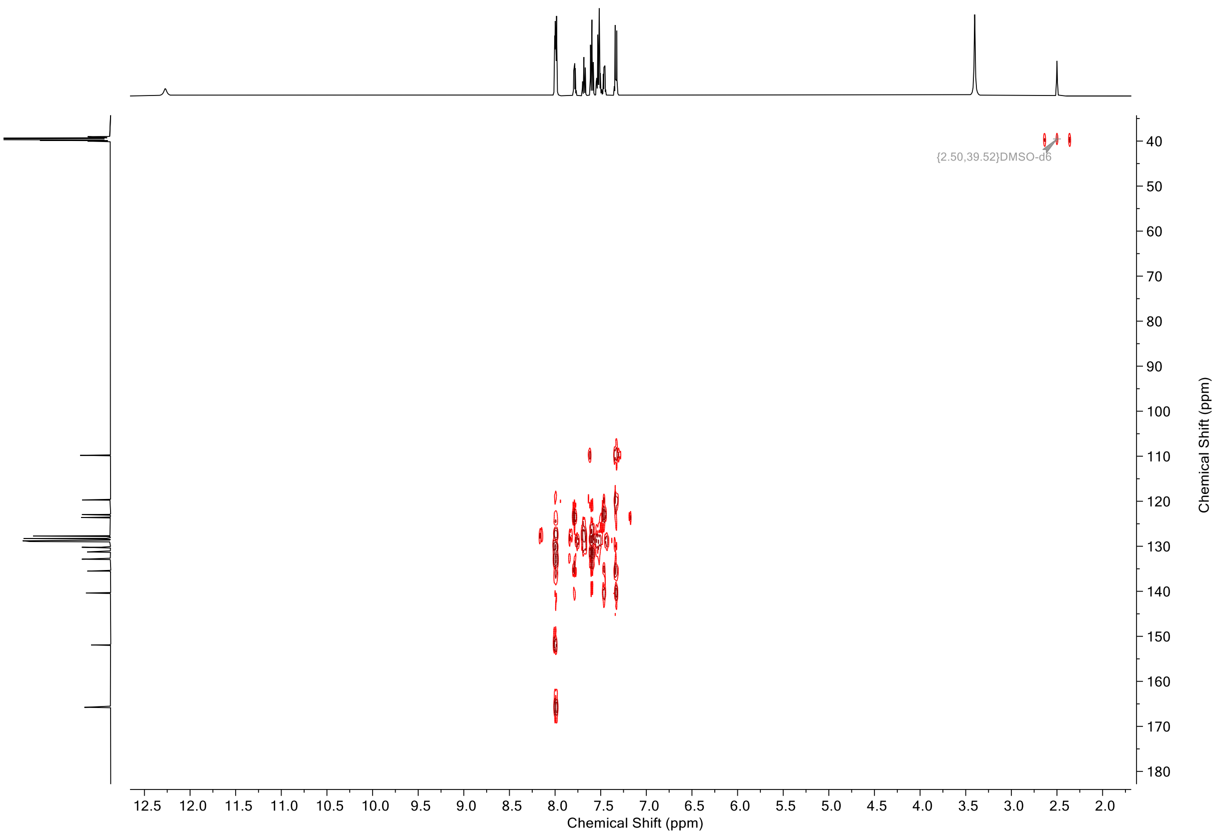

Figure S108: HMBC (DMSO-d_6): 1*H*-*N*-(2-Phenyl-benzo[*d*]imidazol-1-yl)benzamide (**2d**).

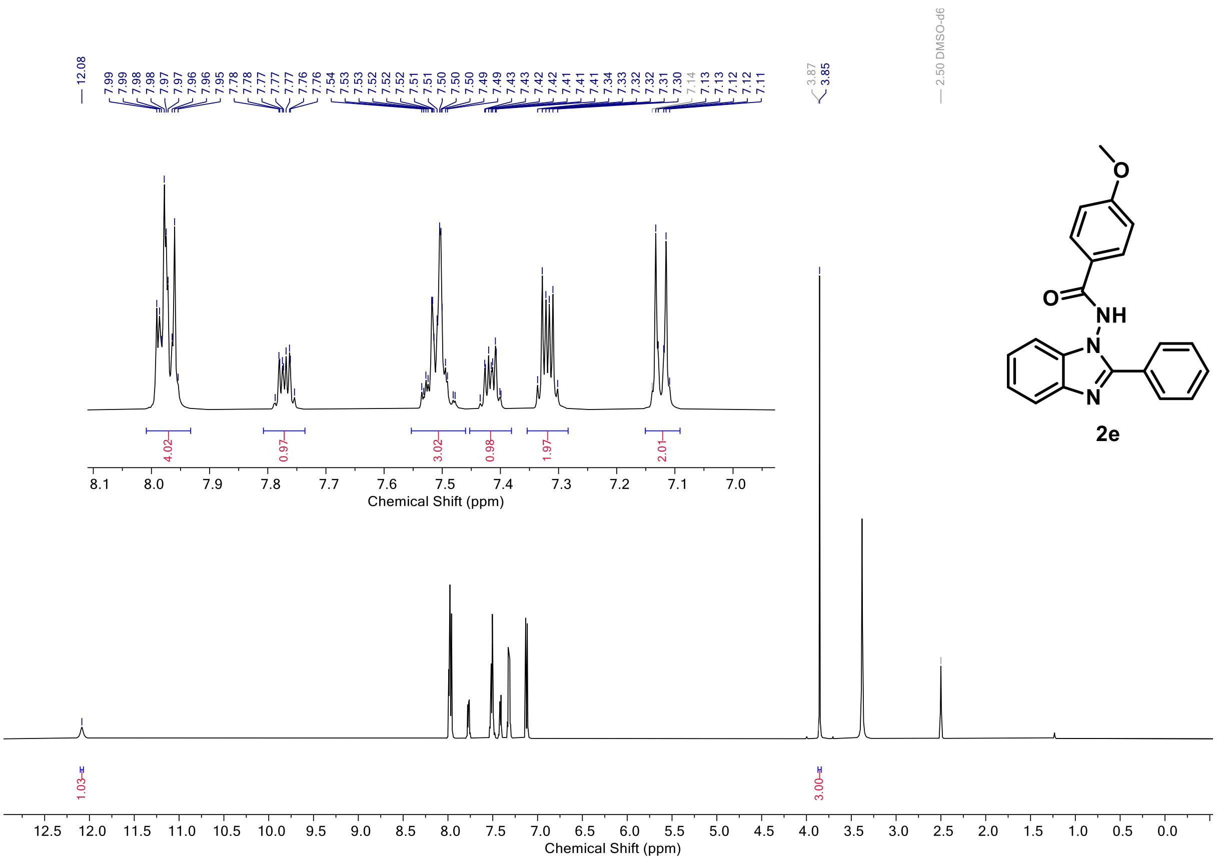

Figure S109: ^{1}H NMR (500 MHz, DMSO-d_6): 1*H*-4-Methoxy-*N*-(2-phenyl-benzo[*d*]imidazol-1-yl)benzamide (**2e**).

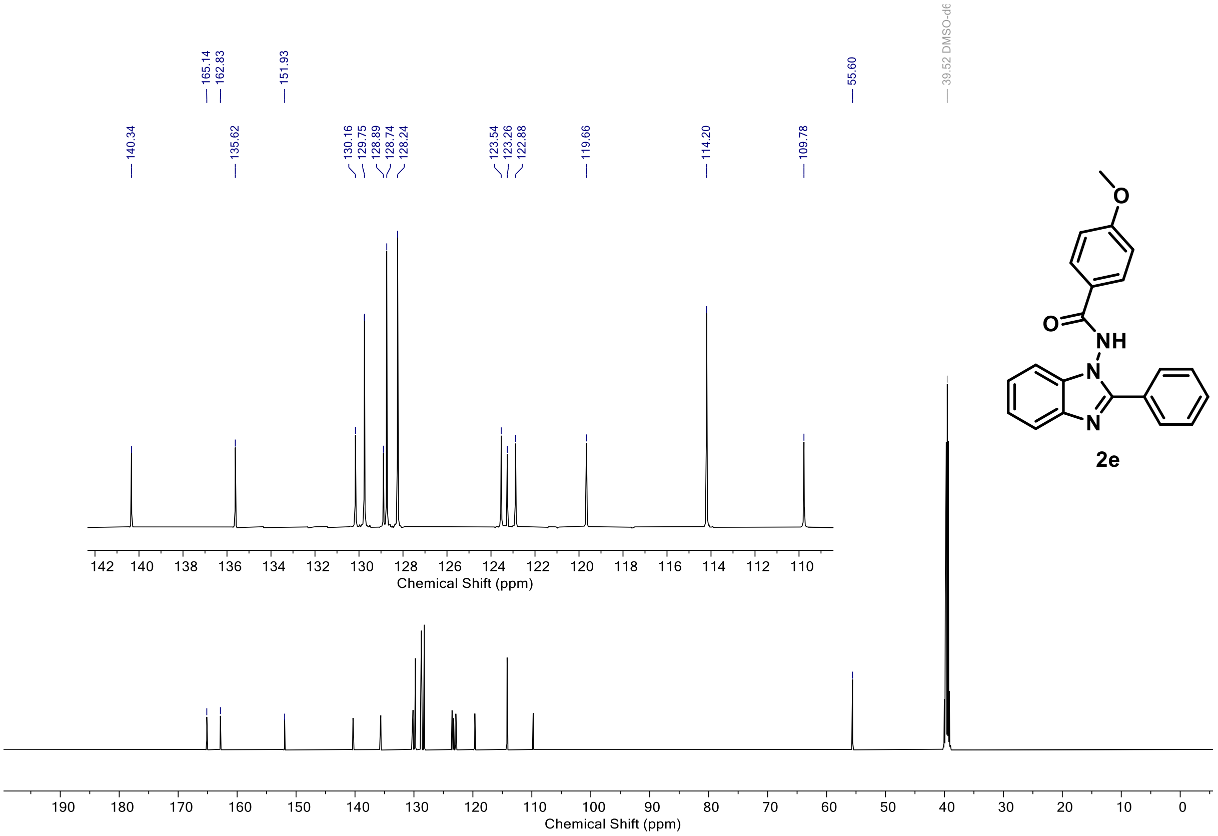

Figure S110: ^{13}C{^{1}H} NMR (126 MHz, DMSO-d_6): 1*H*-4-Methoxy-*N*-(2-phenyl-benzo[*d*]imidazol-1-yl)benzamide (**2e**).

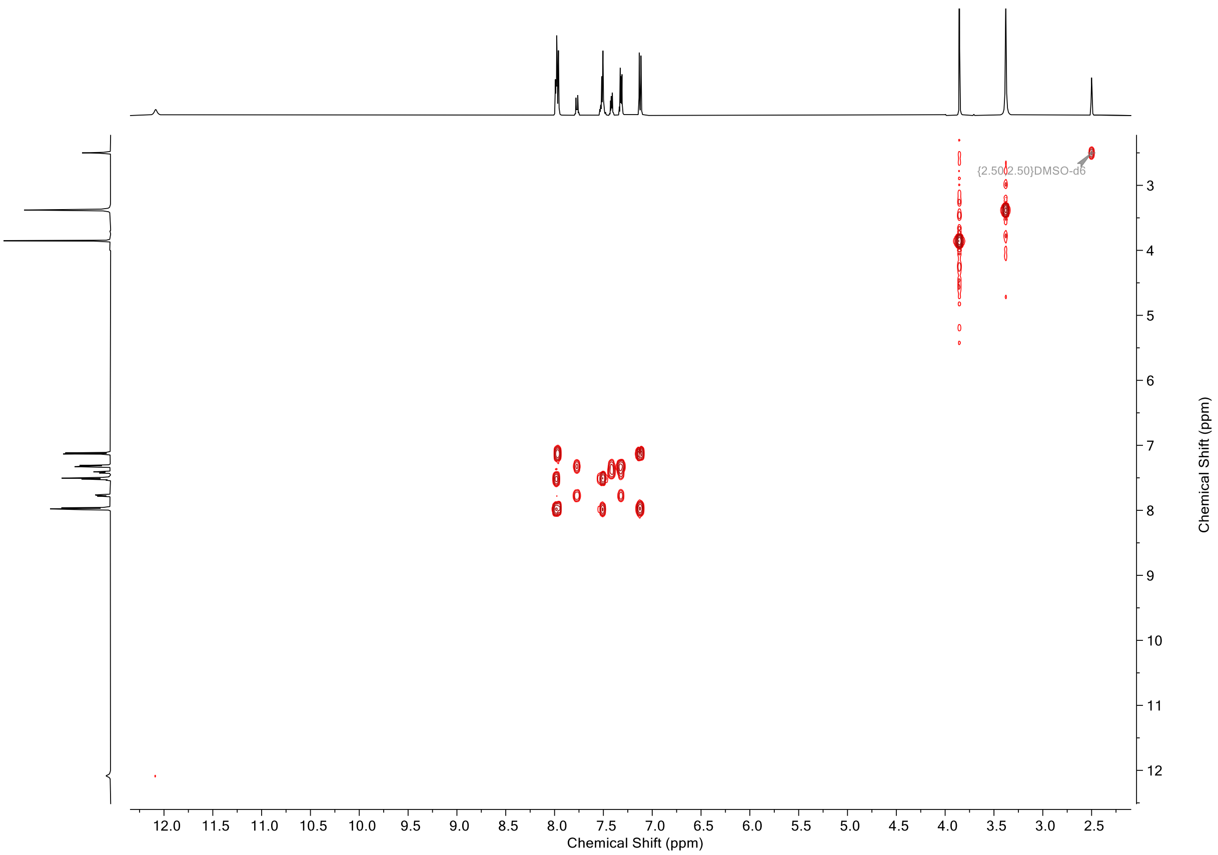

Figure S111: COSY (DMSO-d_6): 1*H*-4-Methoxy-*N*-(2-phenyl-benzo[*d*]imidazol-1-yl)benzamide (**2e**).

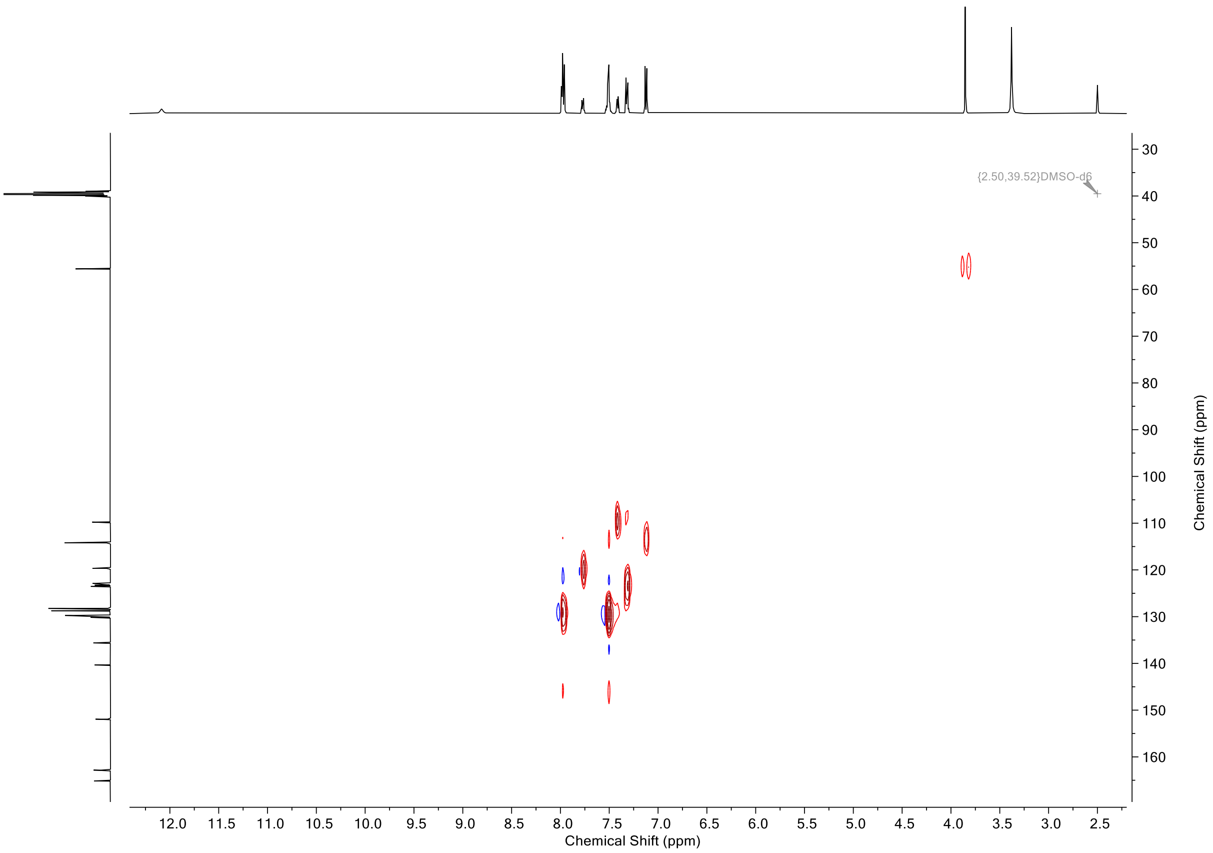

Figure S112: HSQC (DMSO-d_6): 1*H*-4-Methoxy-*N*-(2-phenyl-benzo[*d*]imidazol-1-yl)benzamide (**2e**).

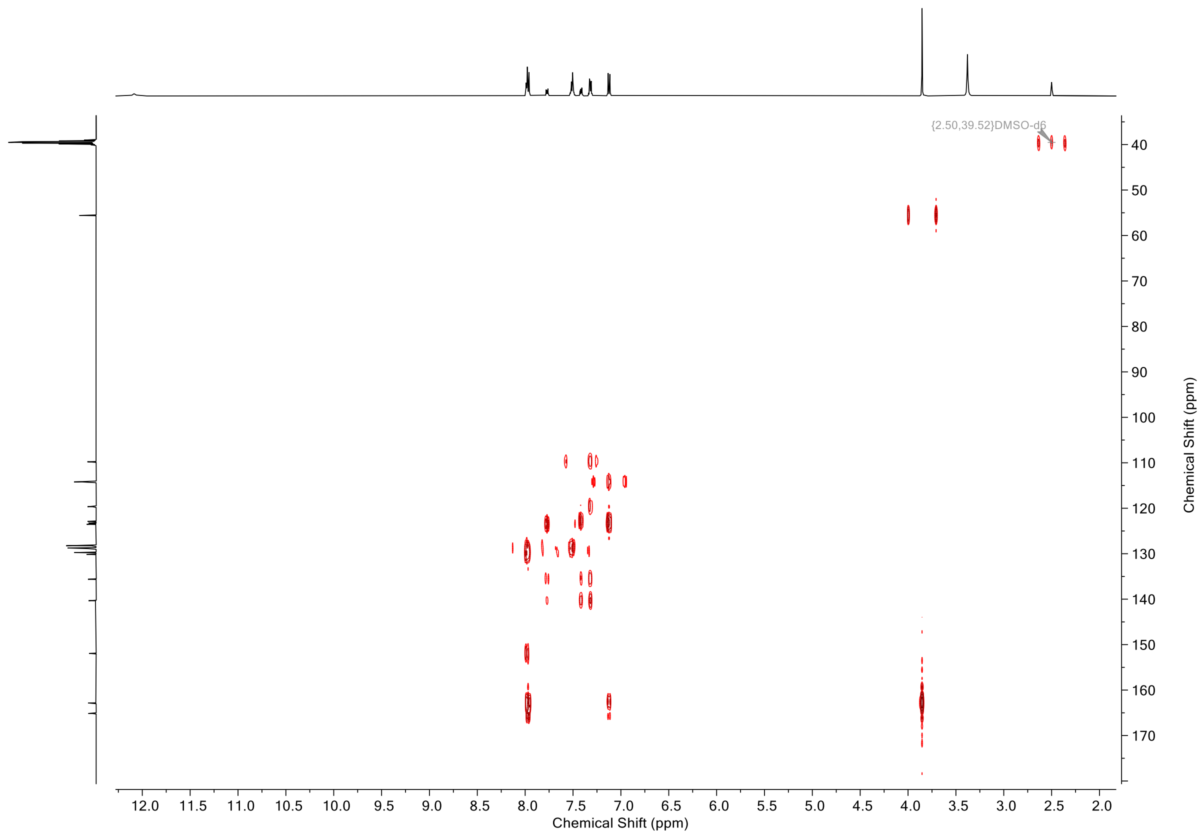

Figure S113: HMBC (DMSO-d_6): 1*H*-4-Methoxy-*N*-(2-phenyl-benzo[*d*]imidazol-1-yl)benzamide (**2e**).

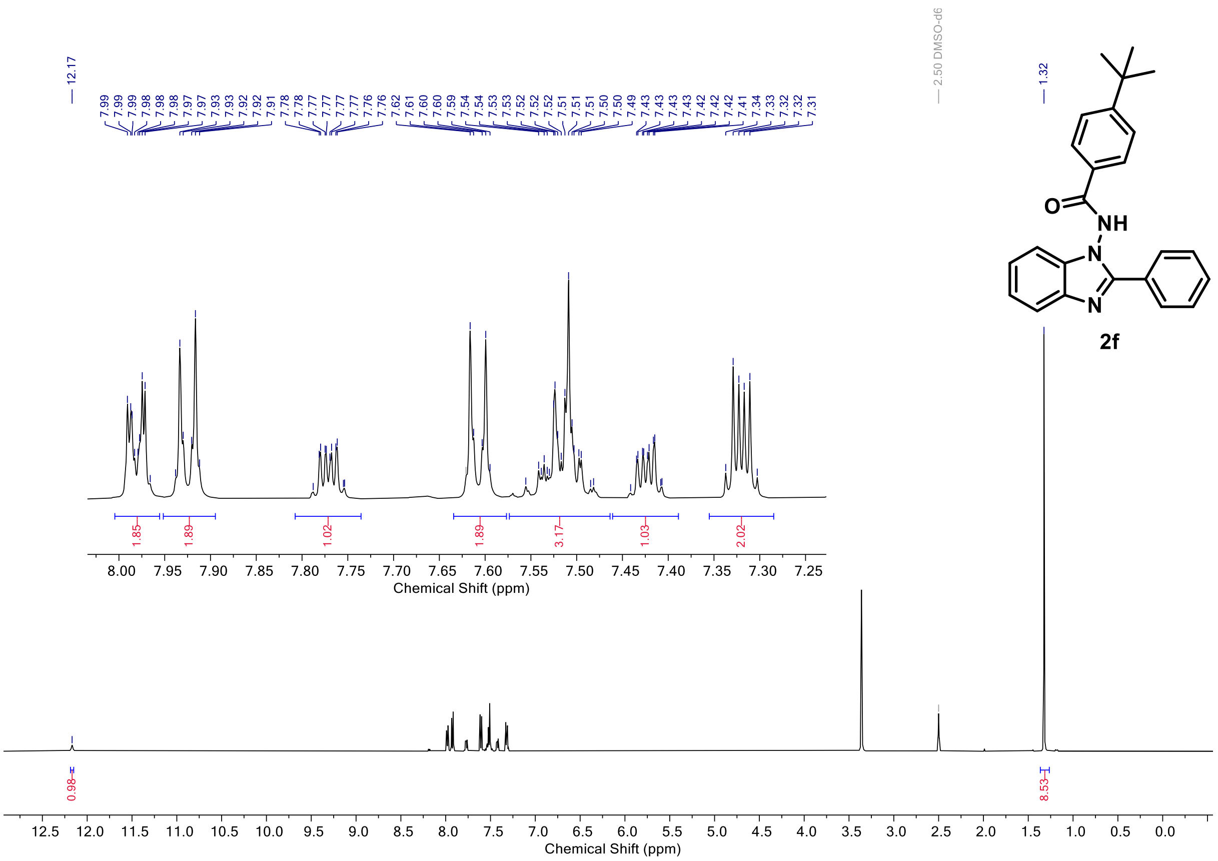

Figure S114: ^{1}H NMR (500 MHz, DMSO-d_6): 1*H*-4-(*tert*-Butyl)-*N*-(2-phenyl-benzo[*d*]imidazol-1-yl)benzamide (**2f**).

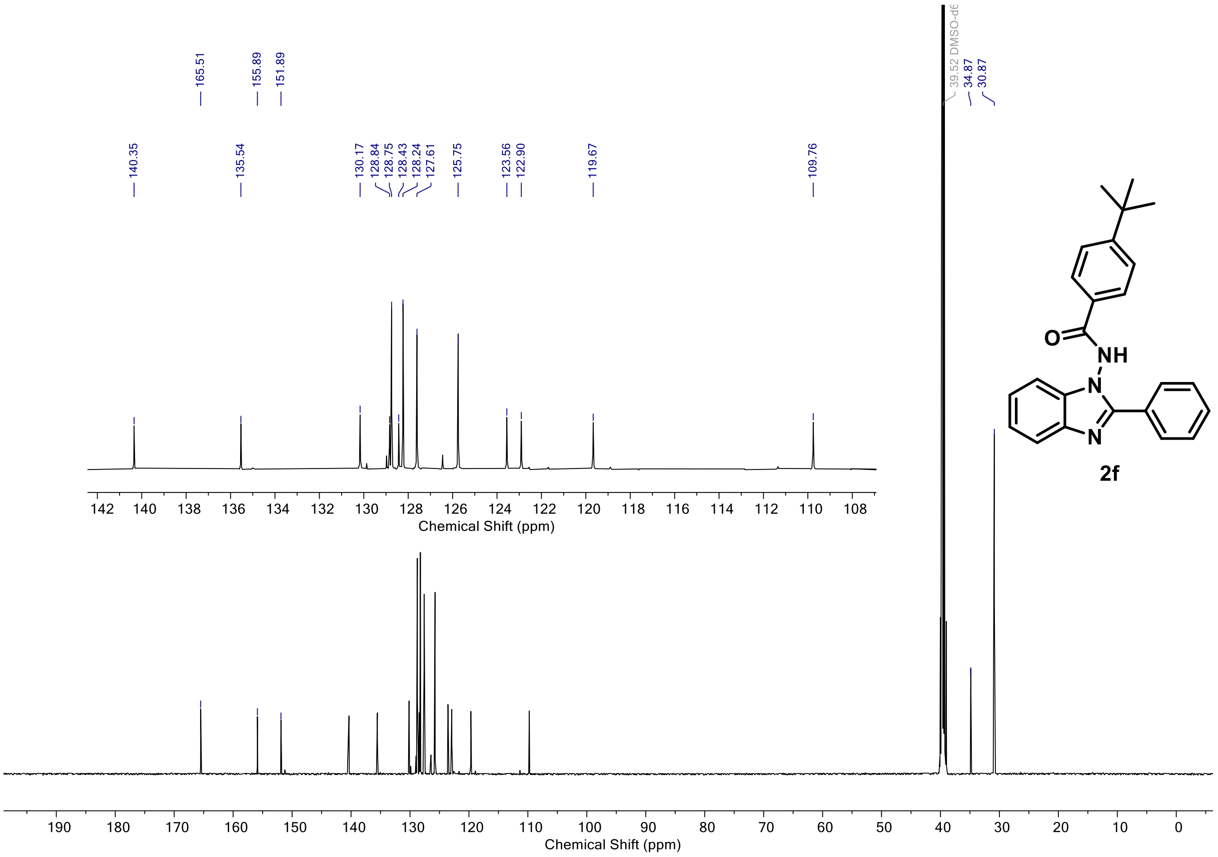

Figure S115: ^{13}C{^{1}H} NMR (126 MHz, DMSO-d_6): 1*H*-4-(*tert*-Butyl)-*N*-(2-phenyl-benzo[*d*]imidazol-1-yl)benzamide (**2f**).

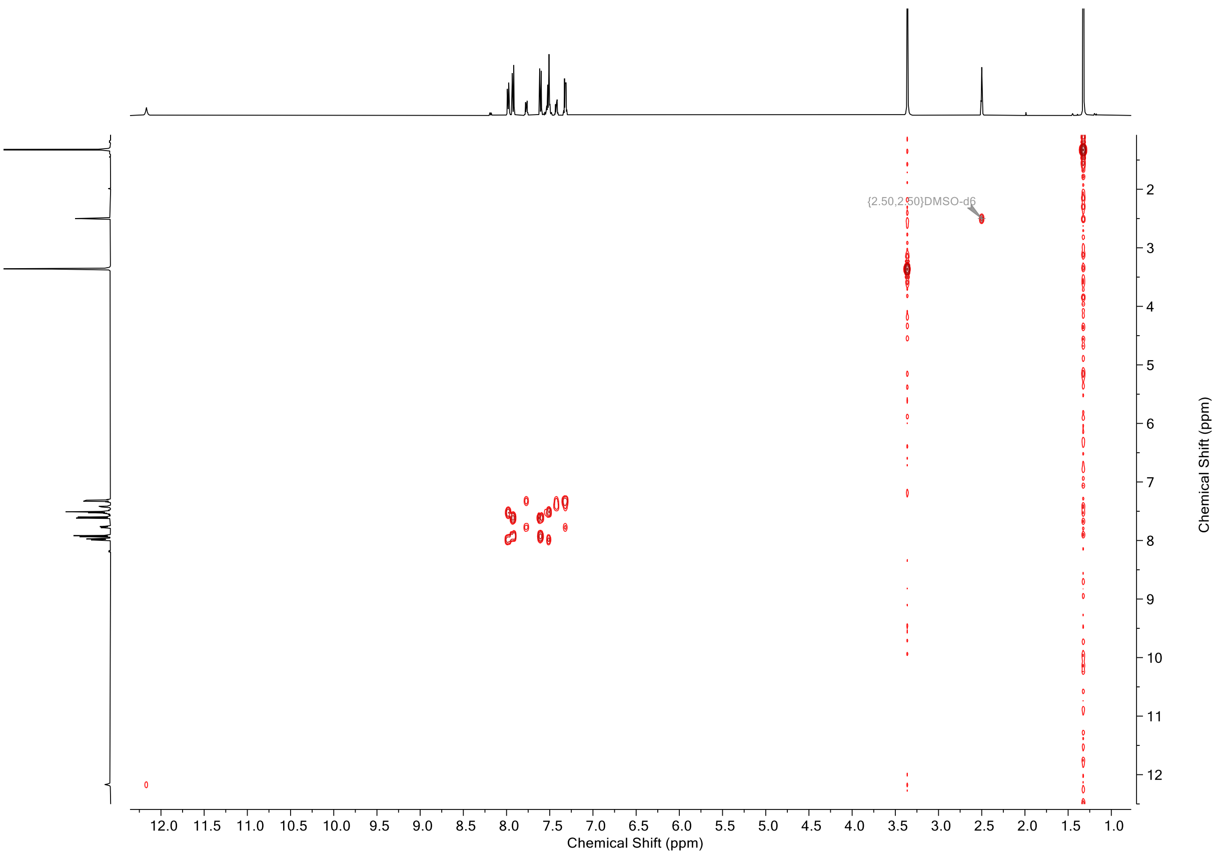

Figure S116: COSY (DMSO-d_6): 1*H*-4-(*tert*-Butyl)-*N*-(2-phenyl-benzo[*d*]imidazol-1-yl)benzamide (**2f**).

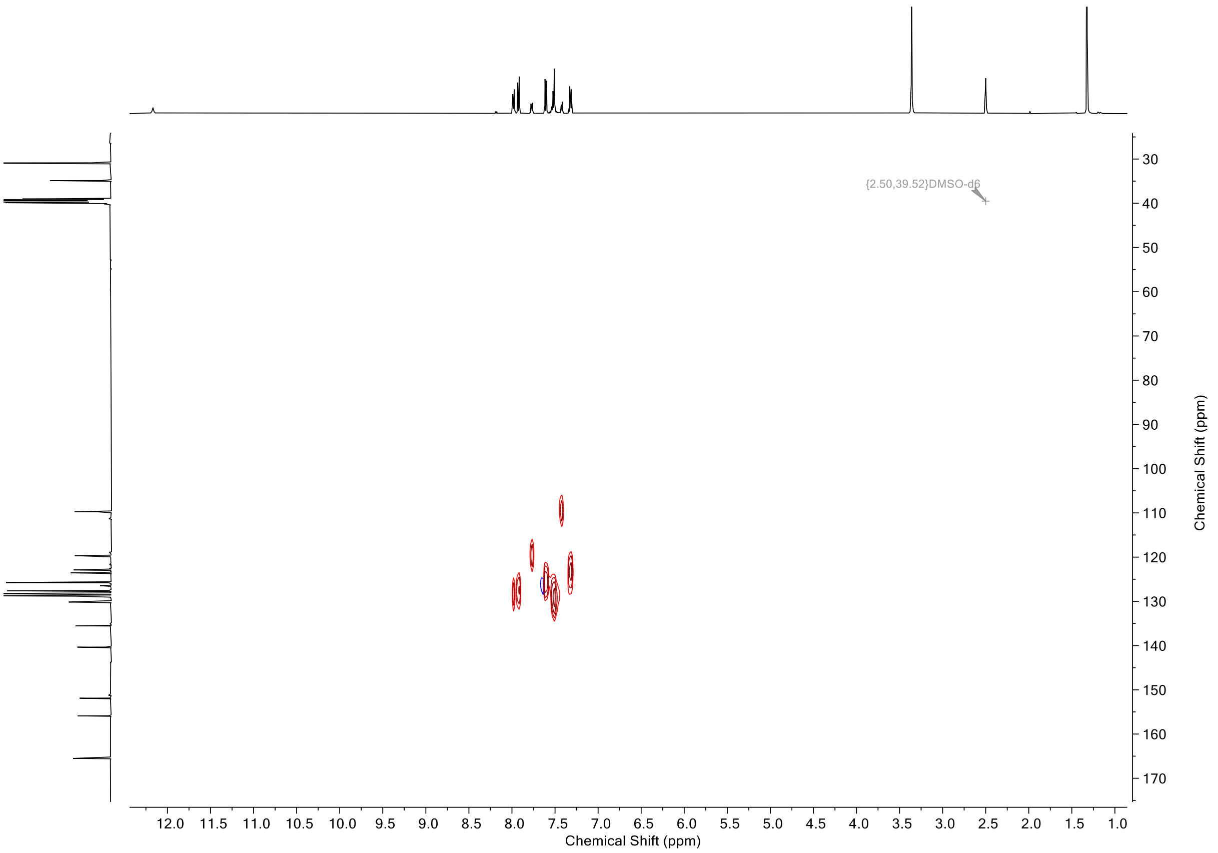

Figure S117: HSQC (DMSO-d_6): 1*H*-4-(*tert*-Butyl)-*N*-(2-phenyl-benzo[*d*]imidazol-1-yl)benzamide (**2f**).

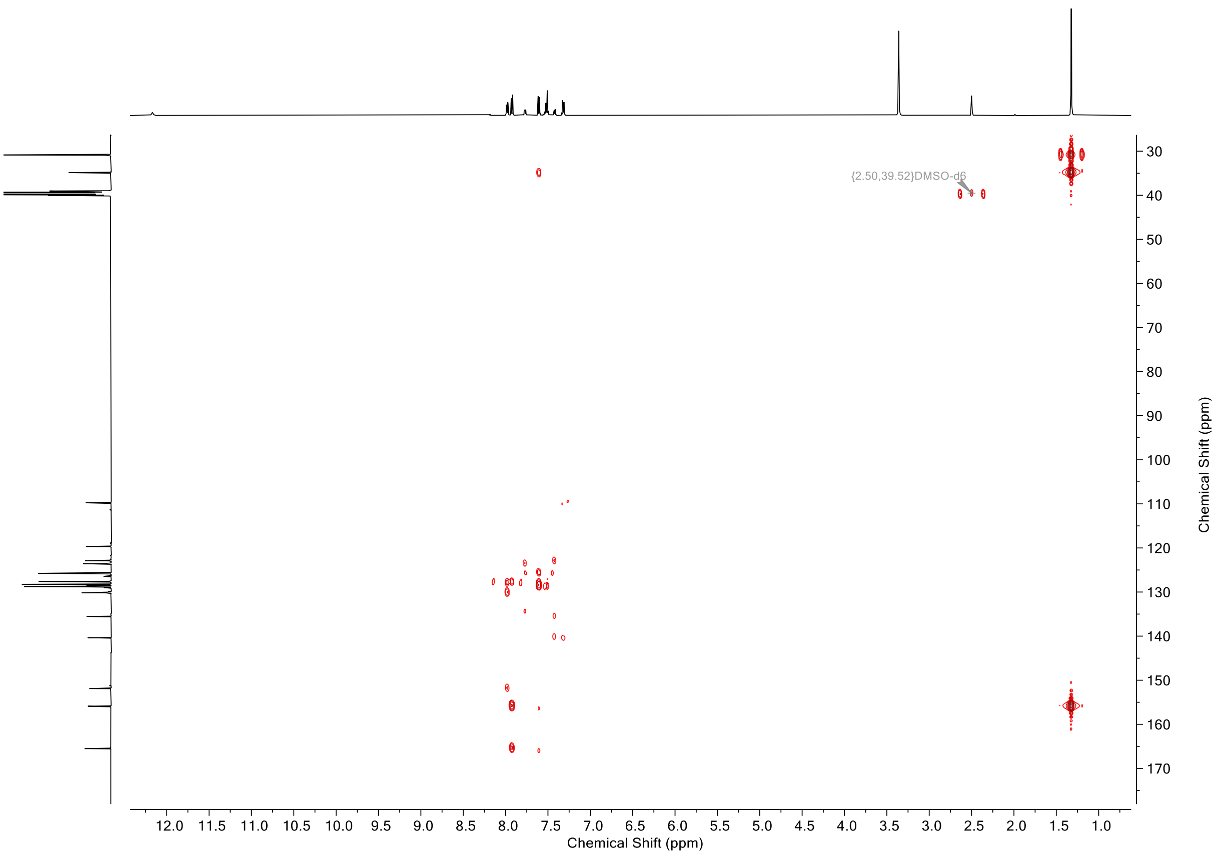

Figure S118: HMBC (DMSO-d_6): 1*H*-4-(*tert*-Butyl)-*N*-(2-phenyl-benzo[*d*]imidazol-1-yl)benzamide (**2f**).

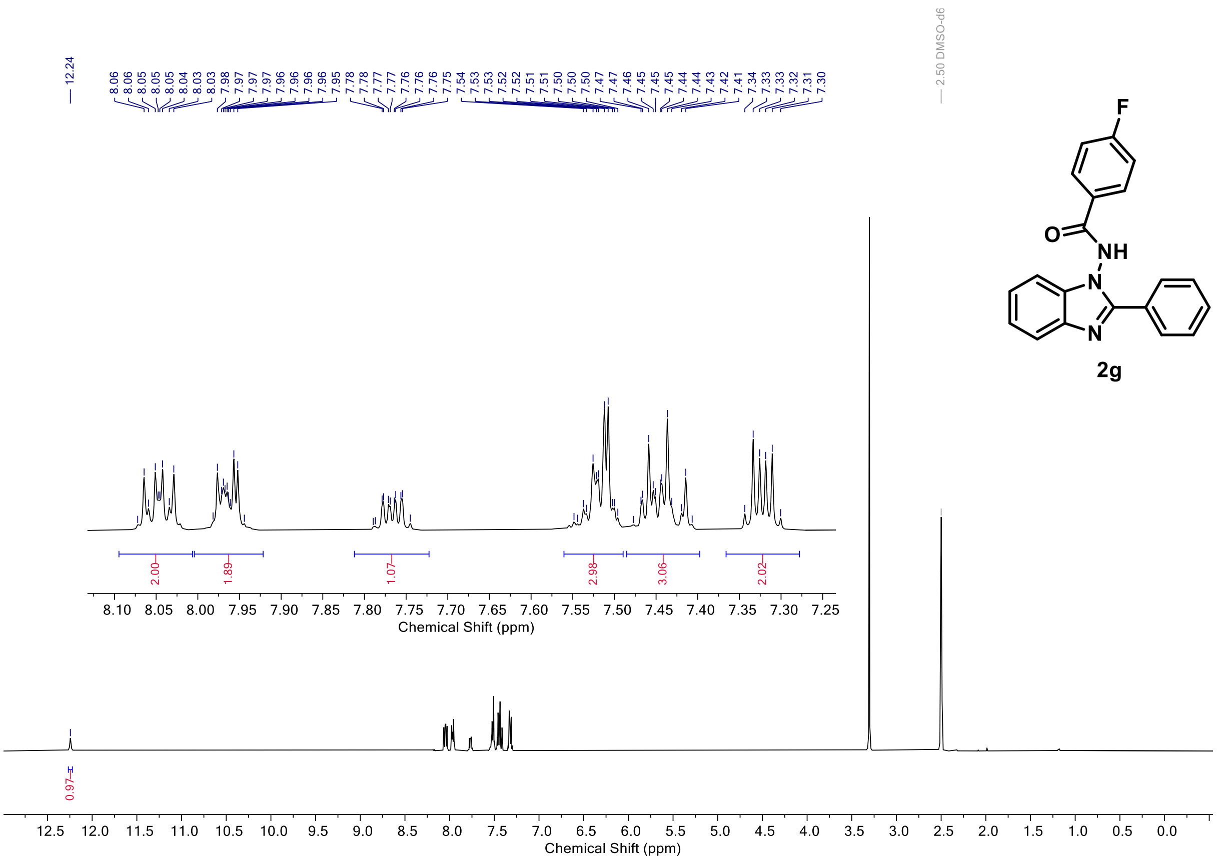

Figure S119: ^{1}H NMR (400 MHz, DMSO-d_6): 1*H*-4-Fluoro-*N*-(2-phenyl-benzo[*d*]imidazol-1-yl)benzamide (**2g**).

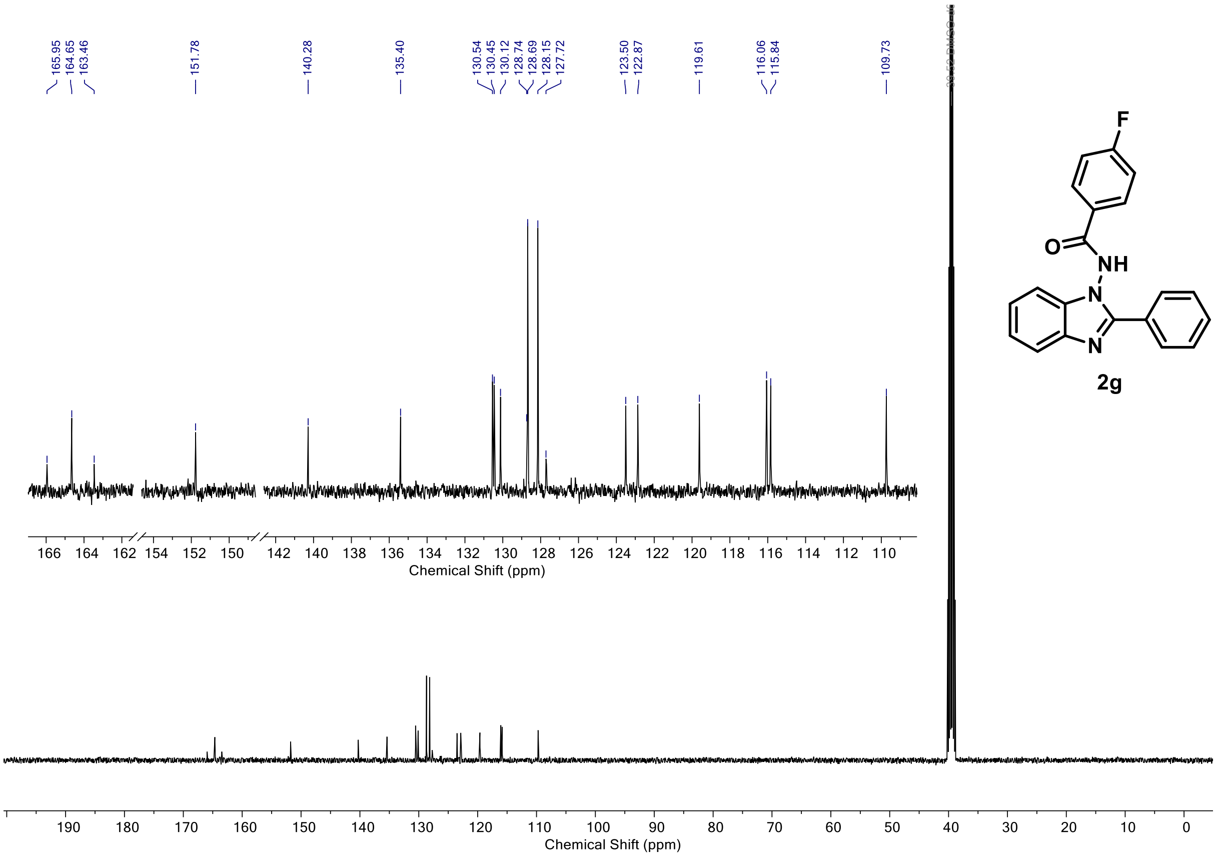

Figure S120: ^{13}C{^{1}H} NMR (101 MHz, DMSO-d_6): 1*H*-4-Fluoro-*N*-(2-phenyl-benzo[*d*]imidazol-1-yl)benzamide (**2g**).

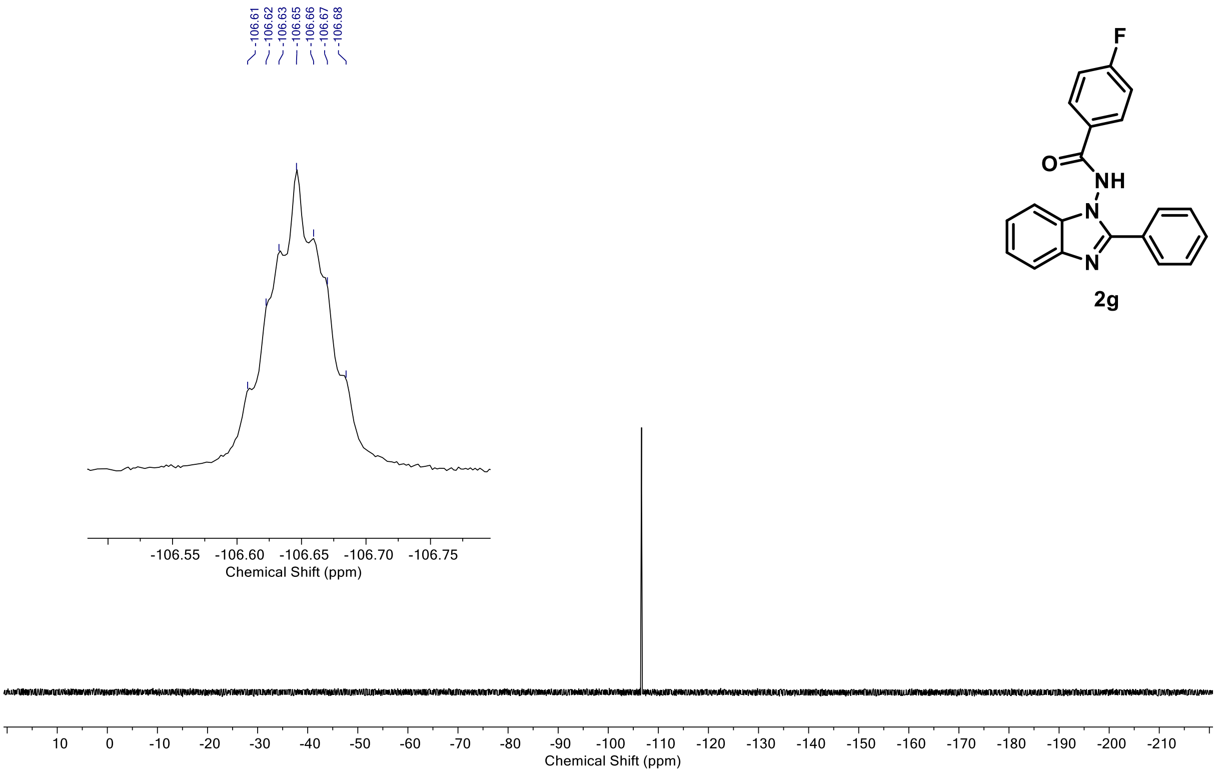

Figure S121: ^{19}F NMR (376 MHz, DMSO-d_6): 1*H*-4-Fluoro-*N*-(2-phenyl-benzo[*d*]imidazol-1-yl)benzamide (**2g**).

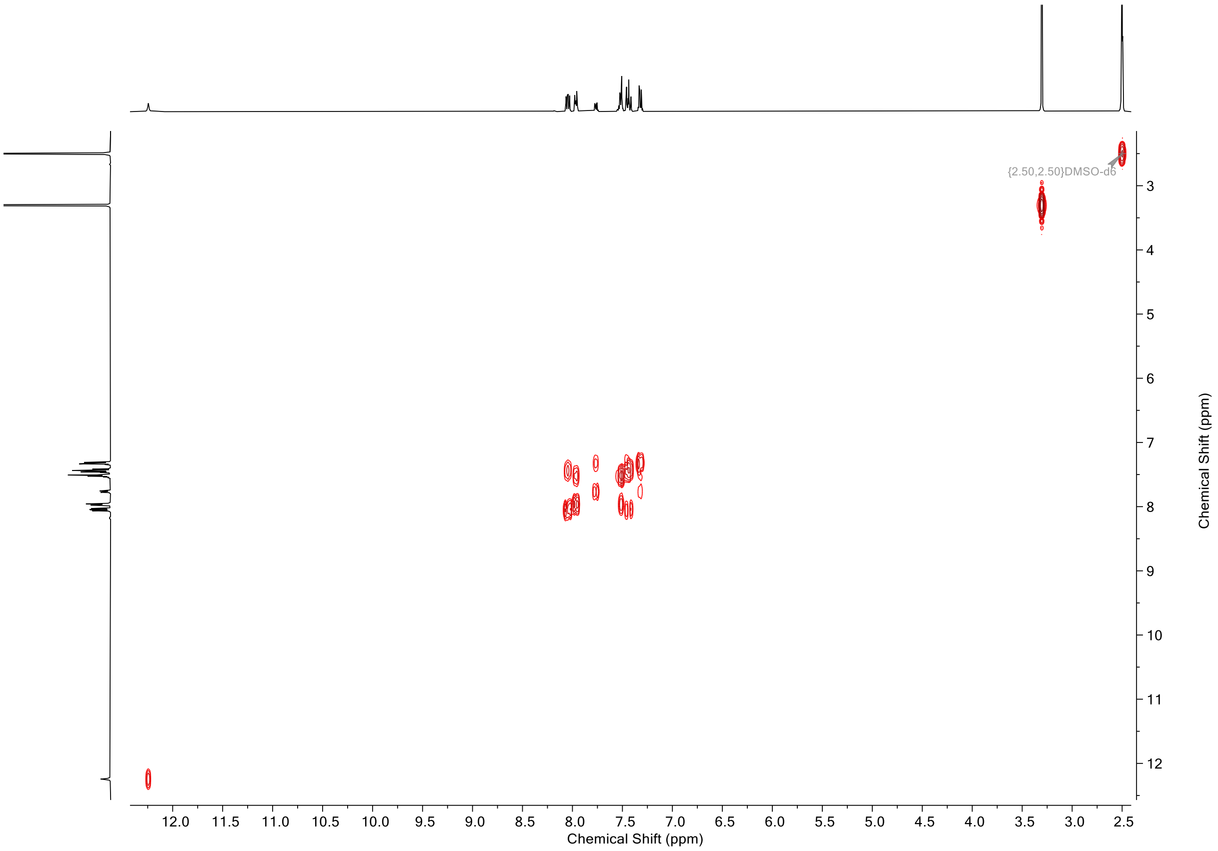

Figure S122: COSY (DMSO-d_6): 1*H*-4-Fluoro-*N*-(2-phenyl-benzo[*d*]imidazol-1-yl)benzamide (**2g**).

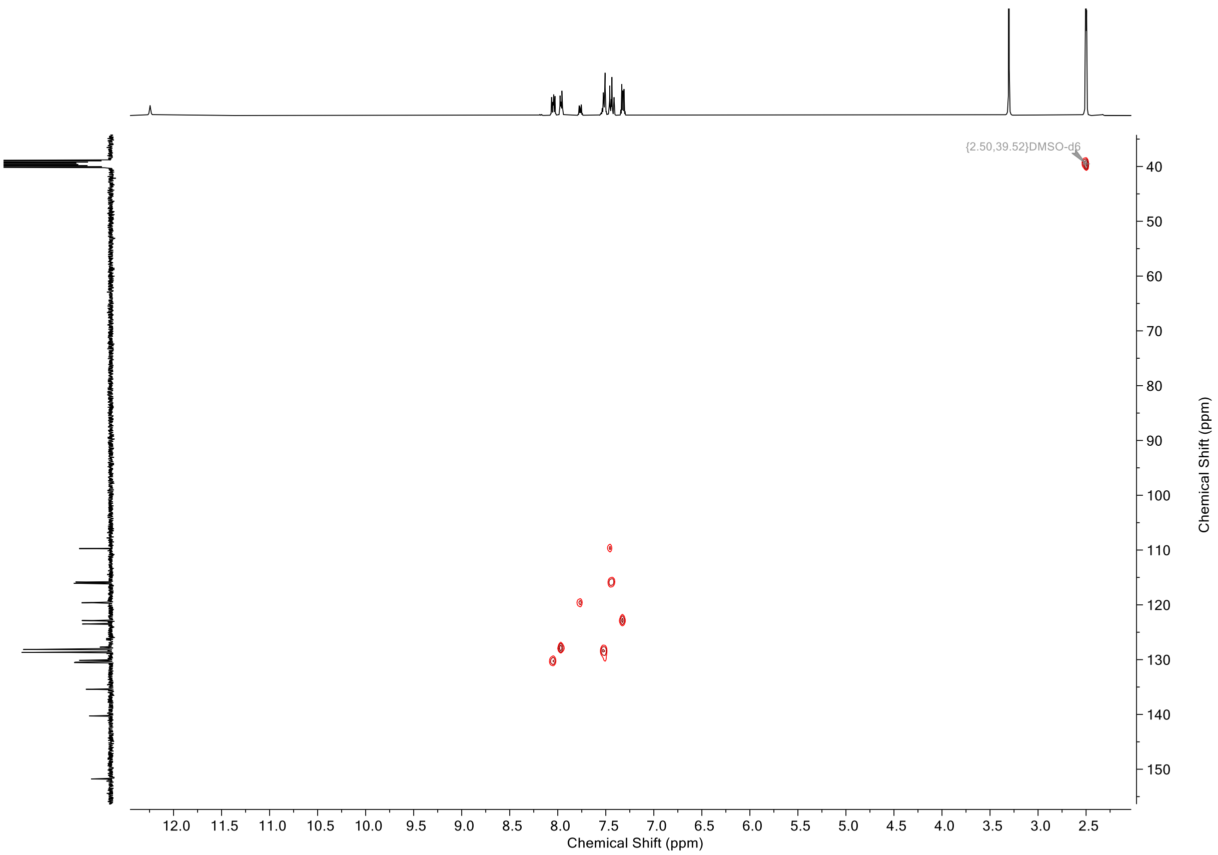

Figure S123: HSQC (DMSO-d_6): 1*H*-4-Fluoro-*N*-(2-phenyl-benzo[*d*]imidazol-1-yl)benzamide (**2g**).

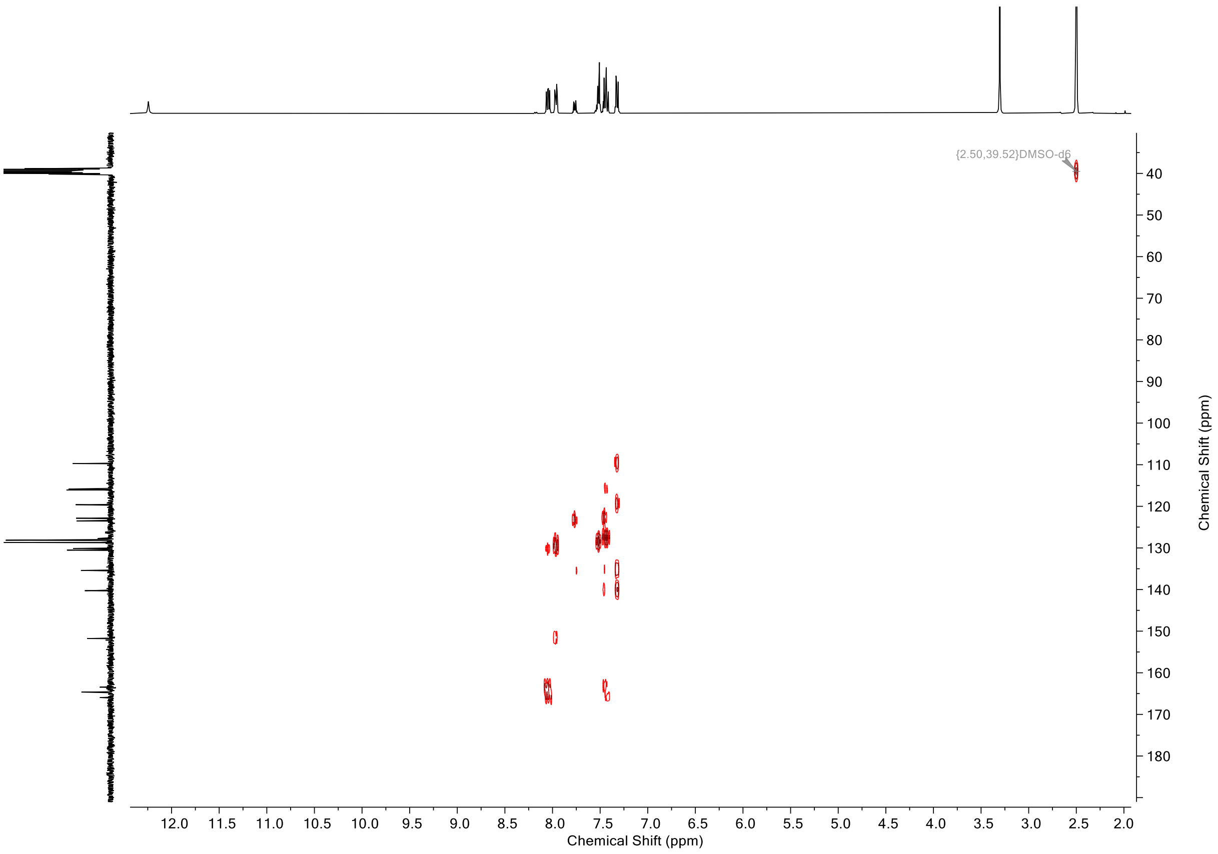

Figure S124: HMBC (DMSO-d_6): 1*H*-4-Fluoro-*N*-(2-phenyl-benzo[*d*]imidazol-1-yl)benzamide (**2g**).

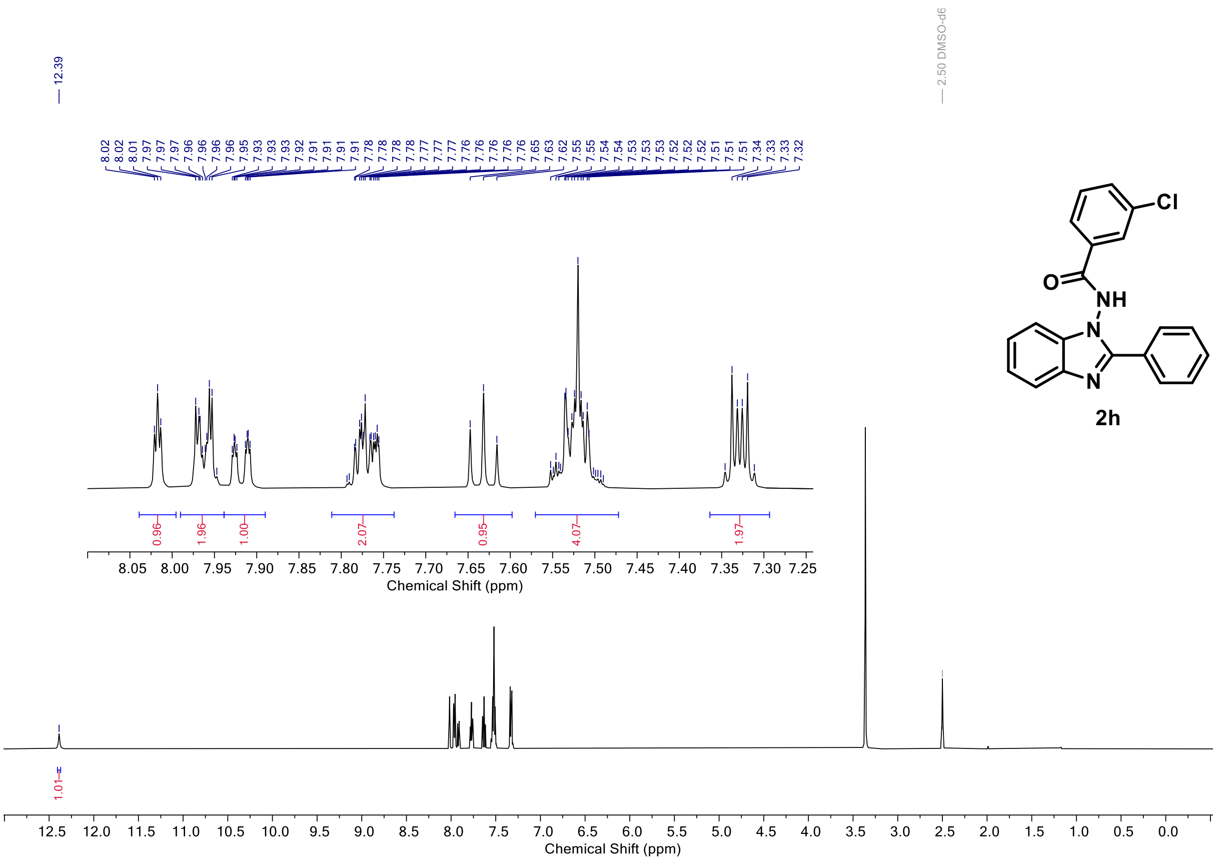

Figure S125: ^{1}H NMR (500 MHz, DMSO-d_6): 1*H*-3-Chloro-*N*-(2-phenyl-benzo[*d*]imidazol-1-yl)benzamide (**2h**).

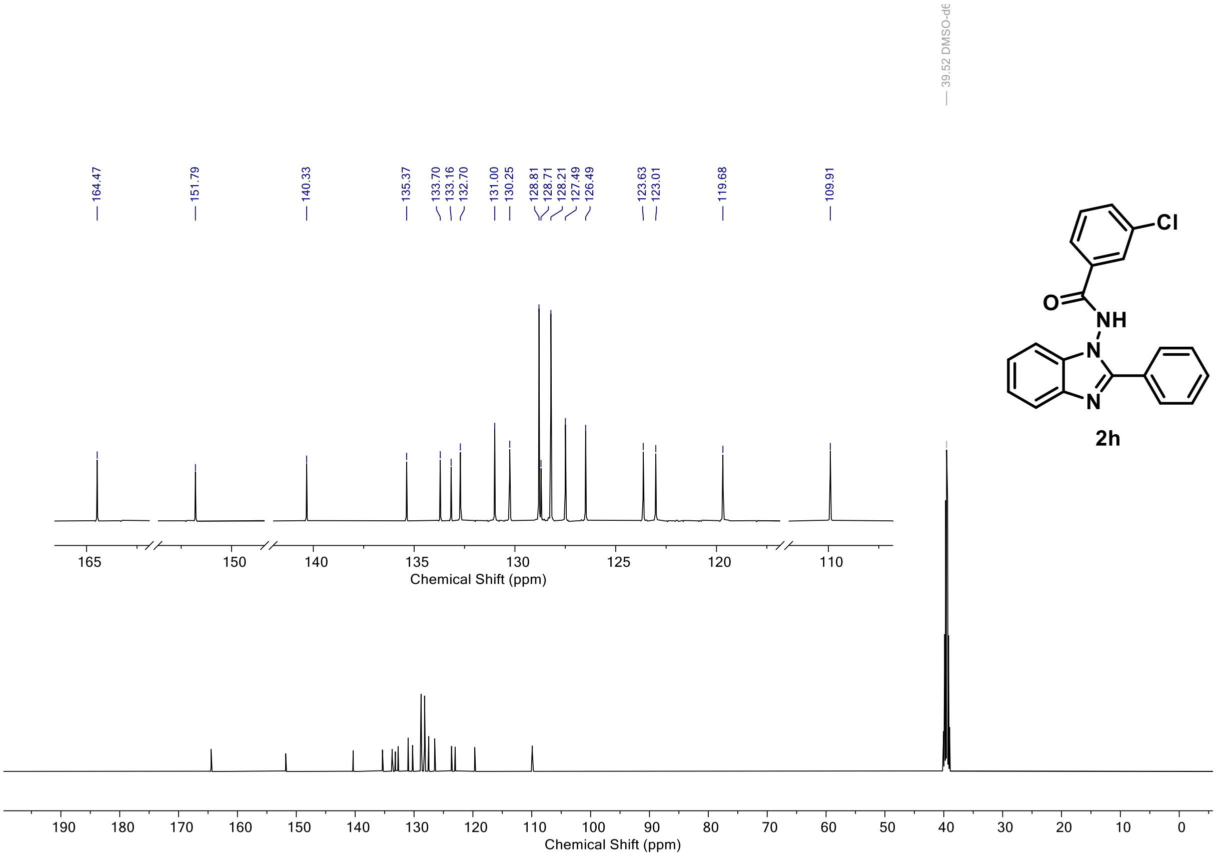

Figure S126: ^{13}C{^{1}H} NMR (126 MHz, DMSO-d_6): 1*H*-3-Chloro-*N*-(2-phenyl-benzo[*d*]imidazol-1-yl)benzamide (**2h**).

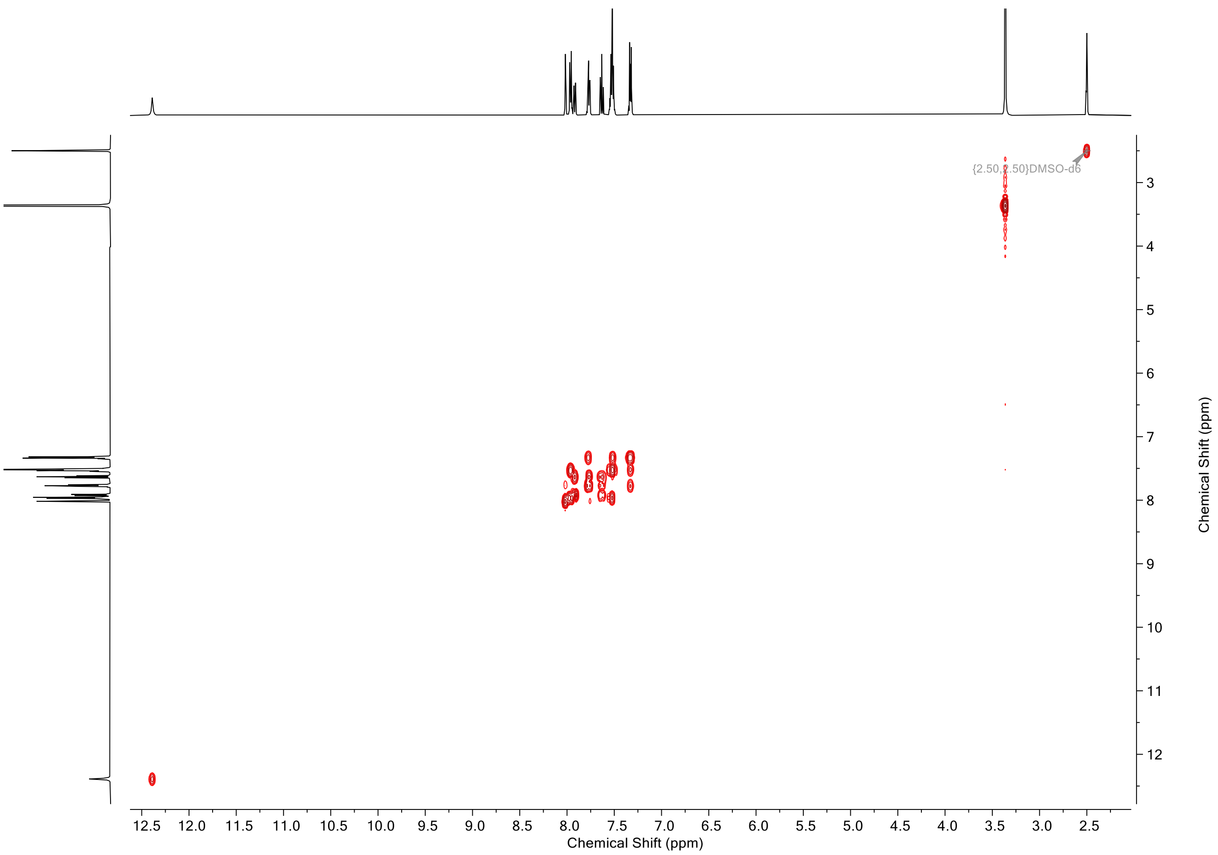

Figure S127: COSY (DMSO-d_6): 1*H*-3-Chloro-*N*-(2-phenyl-benzo[*d*]imidazol-1-yl)benzamide (**2h**).

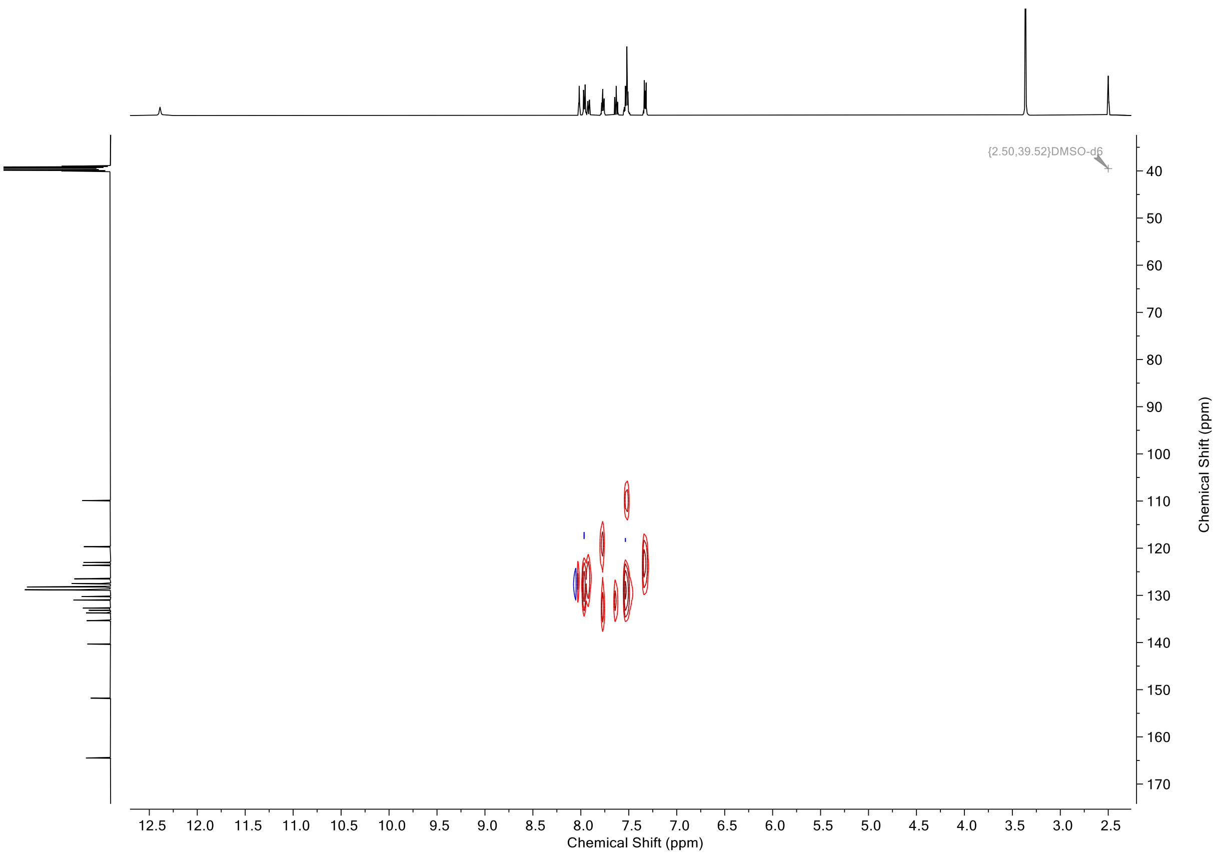

Figure S128: HSQC (DMSO-d_6): 1*H*-3-Chloro-*N*-(2-phenyl-benzo[*d*]imidazol-1-yl)benzamide (**2h**).

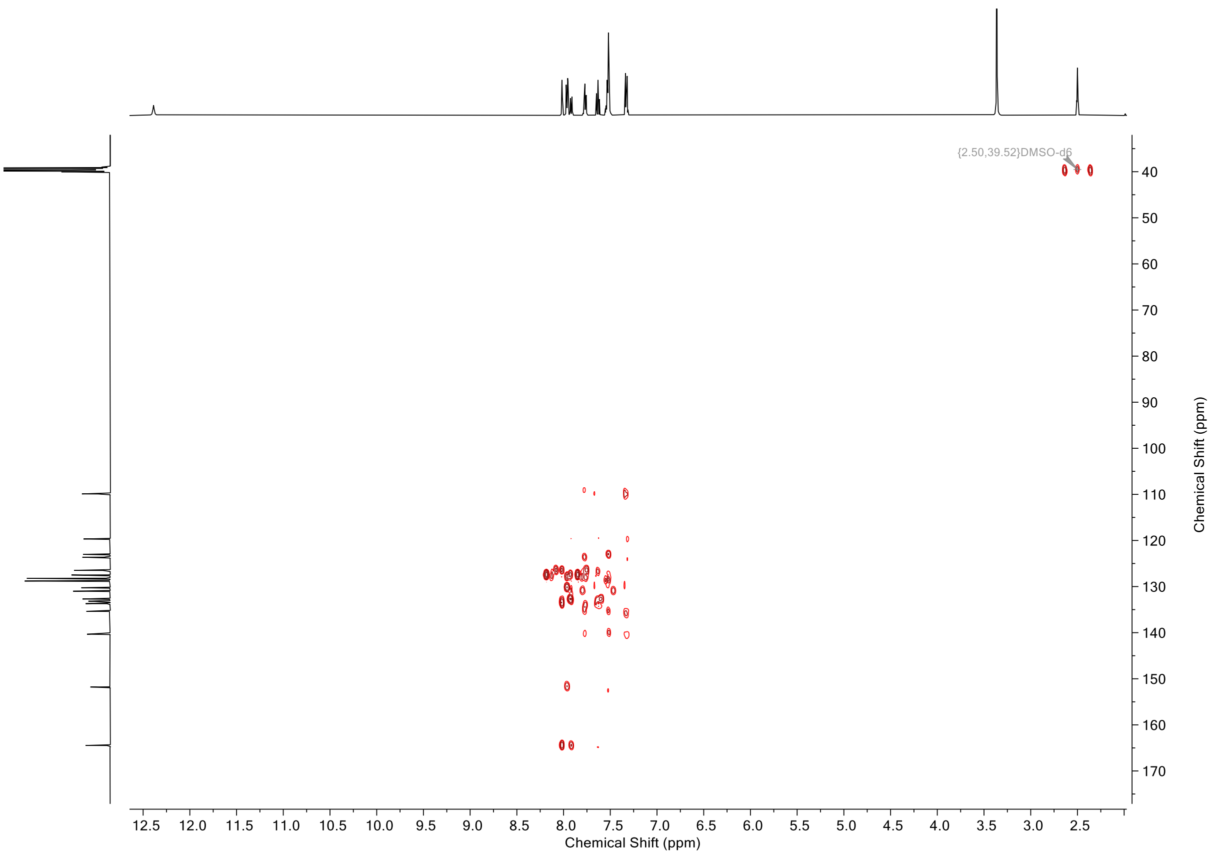

Figure S129: HMBC (DMSO-d_6): 1*H*-3-Chloro-*N*-(2-phenyl-benzo[*d*]imidazol-1-yl)benzamide (**2h**).

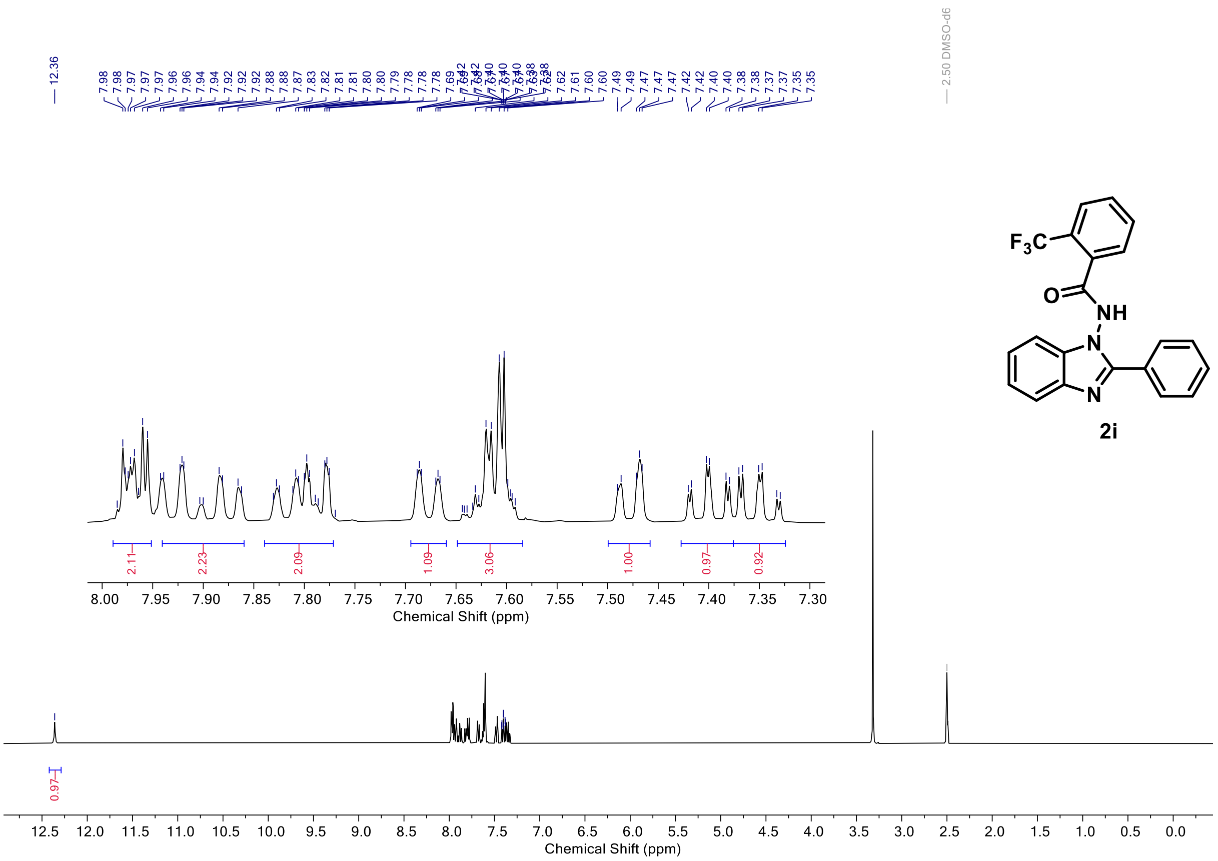

Figure S130: ^{1}H NMR (400 MHz, DMSO-d_6): 1*H*-*N*-(2-Phenyl-benzo[*d*]imidazol-1-yl)-2-(trifluoromethyl)benzamide (**2i**).

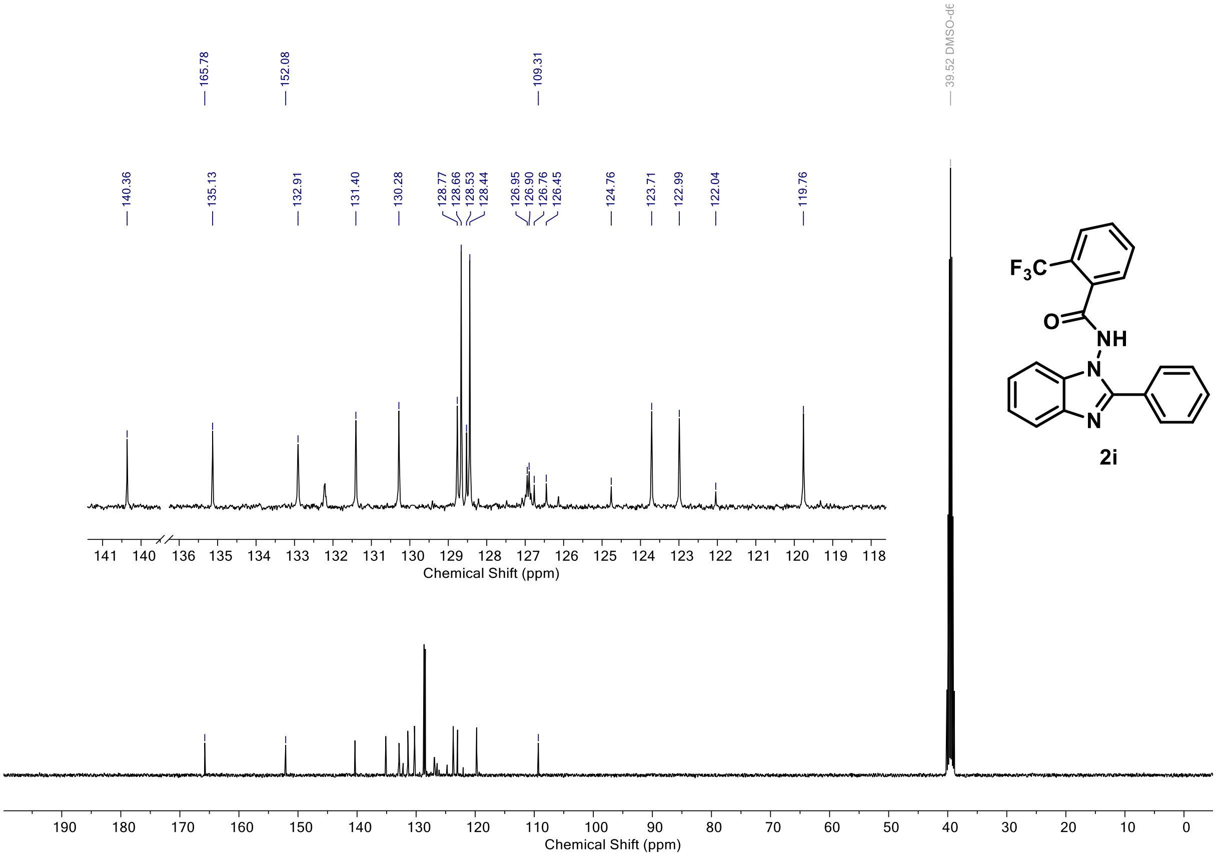

Figure S131: ^{13}C{^{1}H} NMR (101 MHz, DMSO-d_6): 1*H*-*N*-(2-Phenyl-benzo[*d*]imidazol-1-yl)-2-(trifluoromethyl)benz-amide (**2i**).

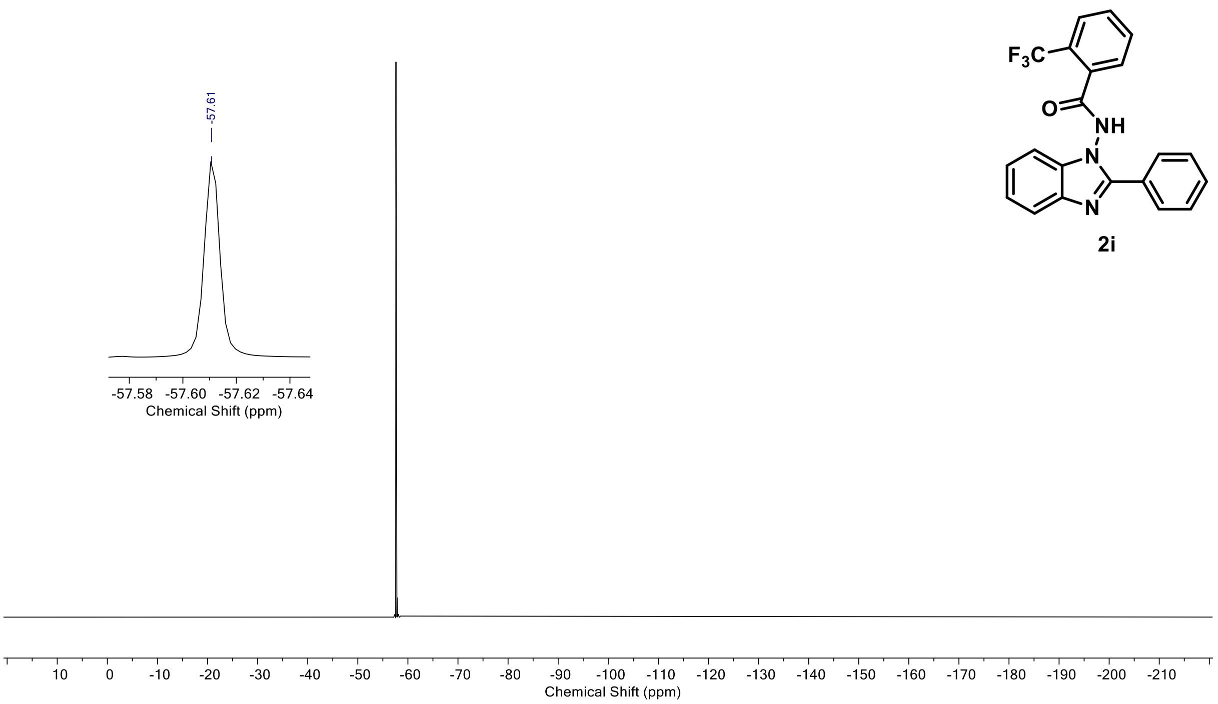

Figure S132: ^{19}F NMR (376 MHz, DMSO-d_6): 1*H*-*N*-(2-Phenyl-benzo[*d*]imidazol-1-yl)-2-(trifluoromethyl)benzamide (**2i**).

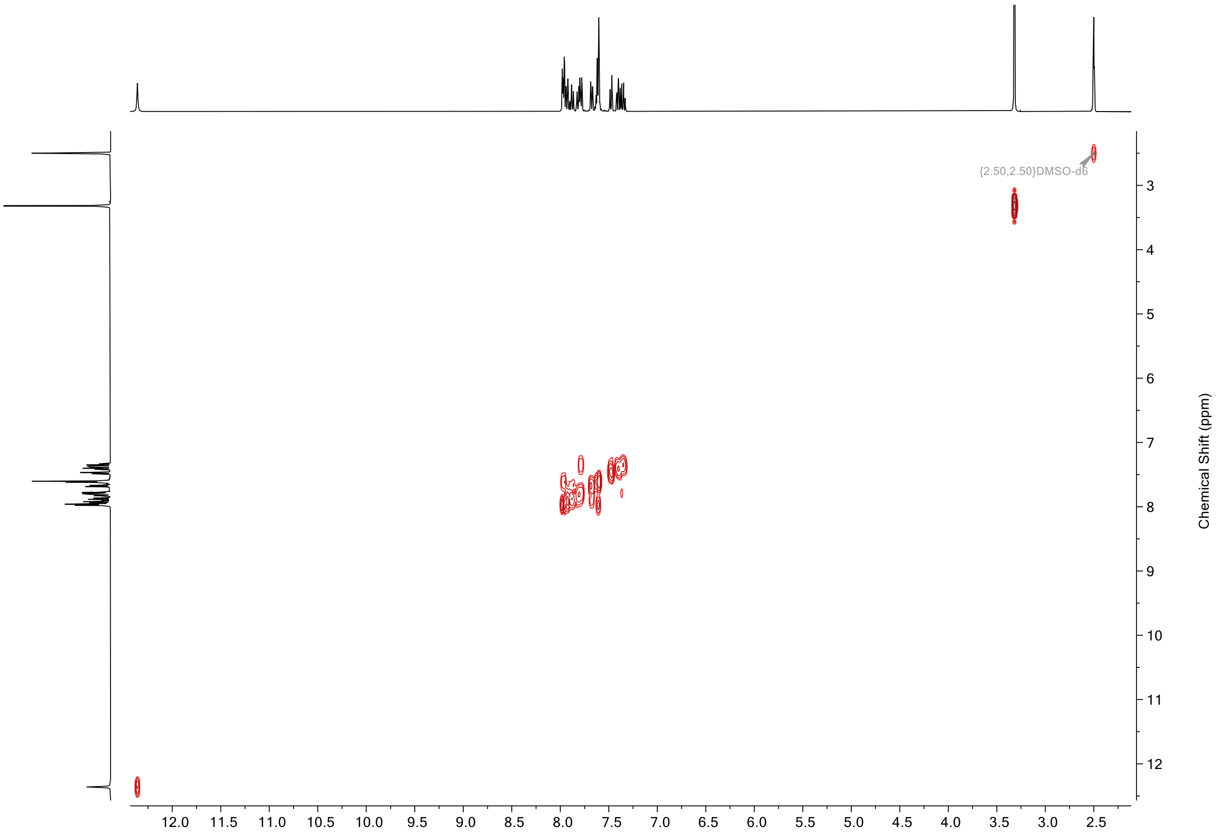

Figure S133: COSY (DMSO-d_6): 1*H*-*N*-(2-Phenyl-benzo[*d*]imidazol-1-yl)-2-(trifluoromethyl)benzamide (**2i**).

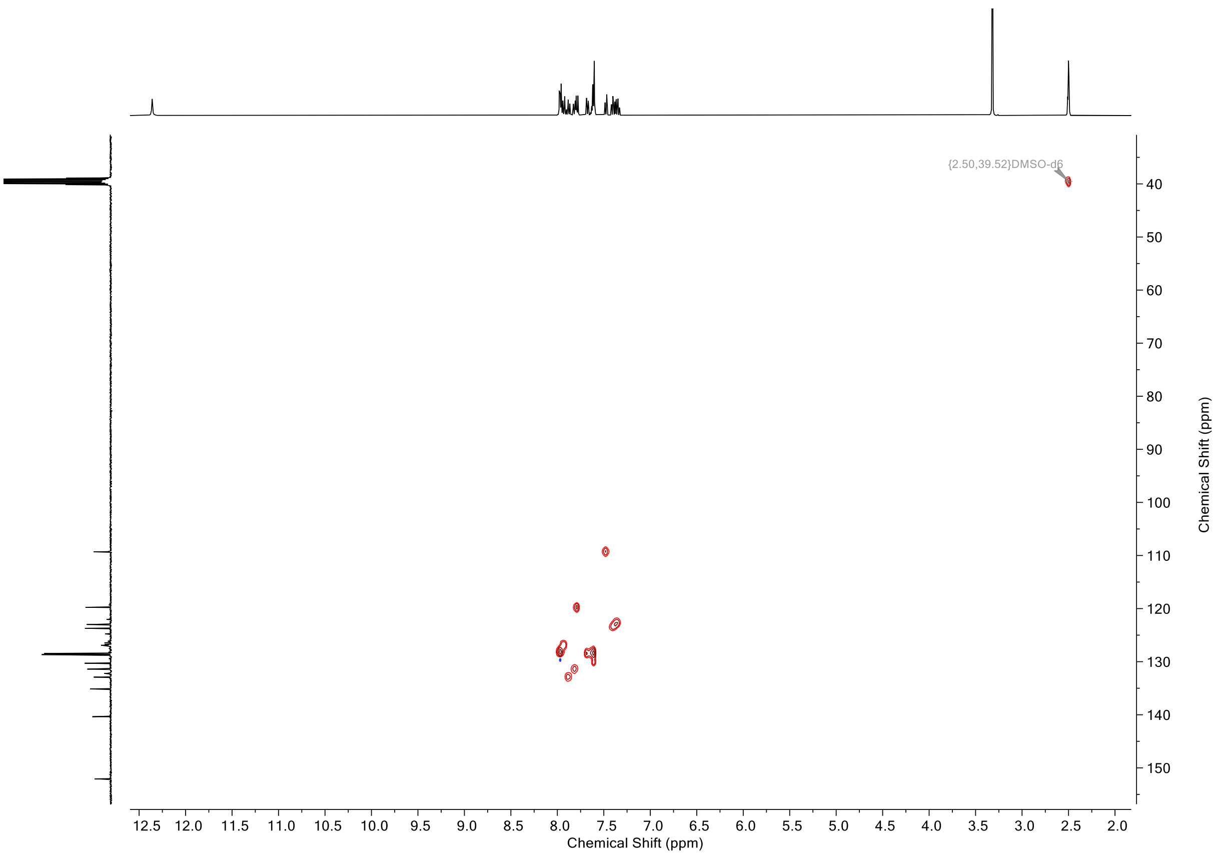

Figure S134: HSQC (DMSO-d_6): 1*H*-*N*-(2-Phenyl-benzo[*d*]imidazol-1-yl)-2-(trifluoromethyl)benzamide (**2i**).

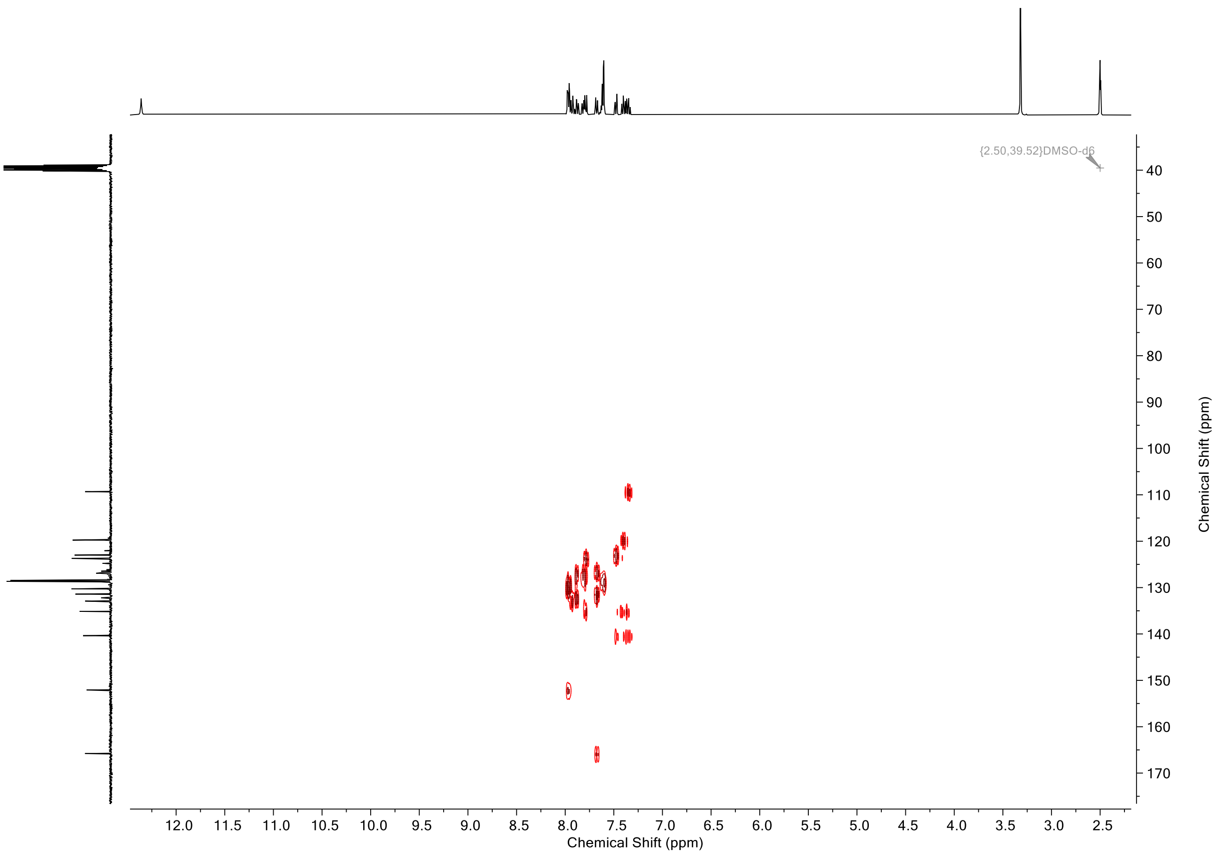

Figure S135: HMBC (DMSO-d_6): 1*H*-*N*-(2-Phenyl-benzo[*d*]imidazol-1-yl)-2-(trifluoromethyl)benzamide (**2i**).

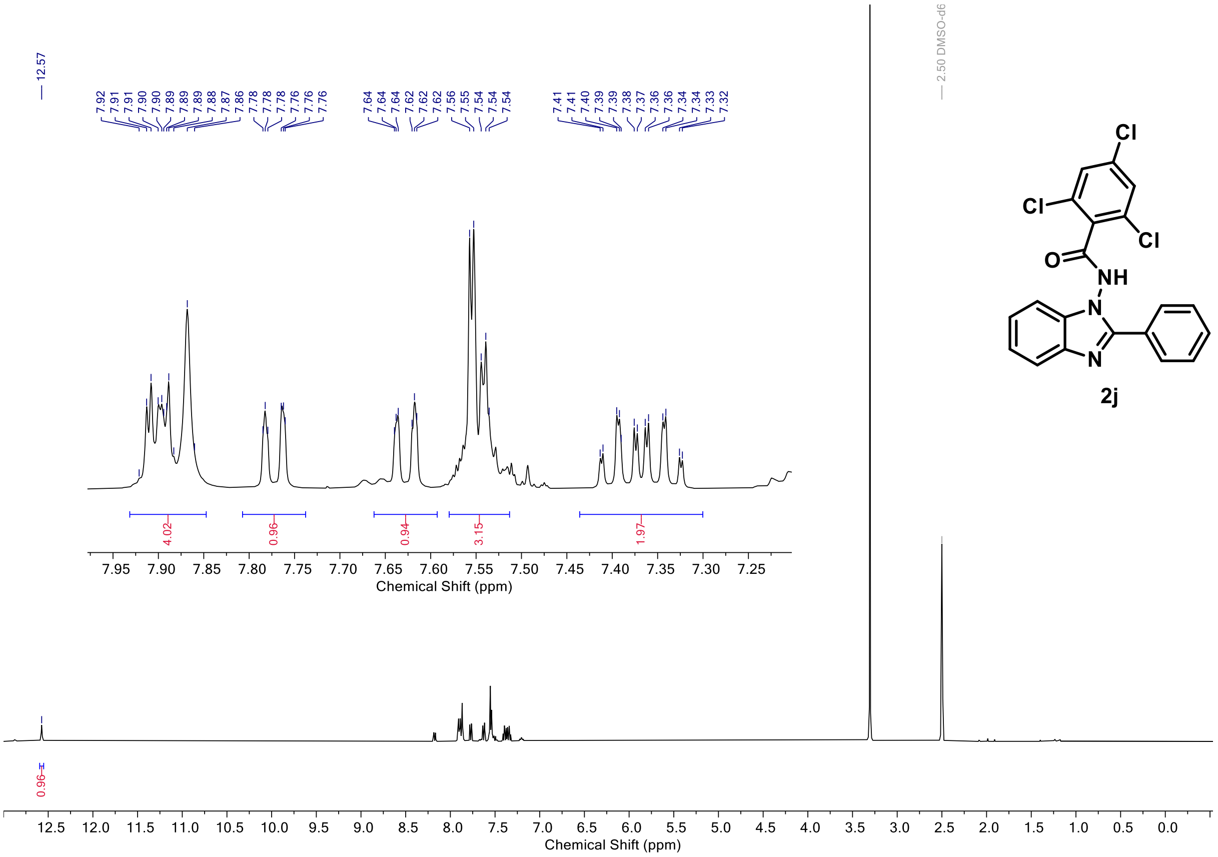

Figure S136: ^{1}H NMR (400 MHz, DMSO-d_6): 1*H*-2,4,6-Trichloro-*N*-(2-phenyl-benzo[*d*]imidazol-1-yl)benzamide (**2j**).

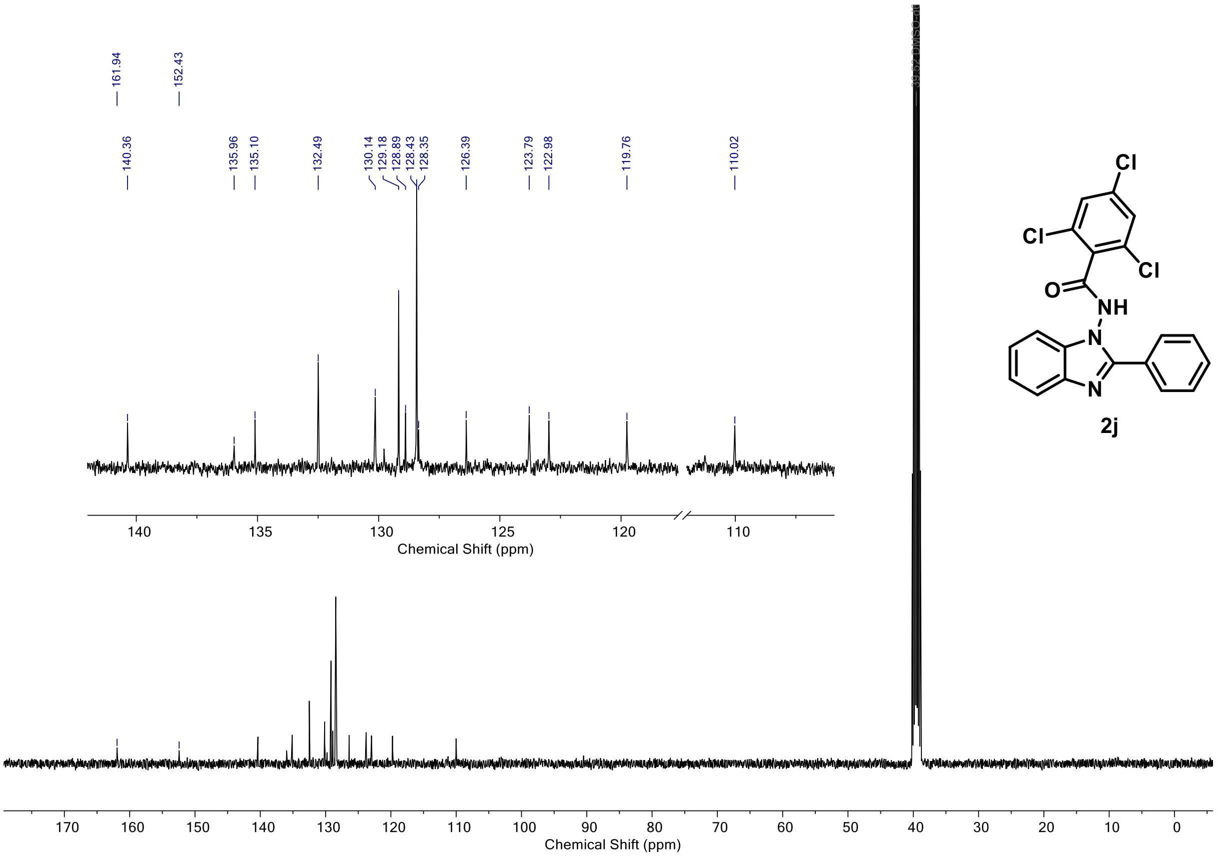

Figure S137: ^{13}C{^{1}H} NMR (101 MHz, DMSO-d_6): 1*H*-2,4,6-Trichloro-*N*-(2-phenyl-benzo[*d*]imidazol-1-yl)benzamide (**2j**).

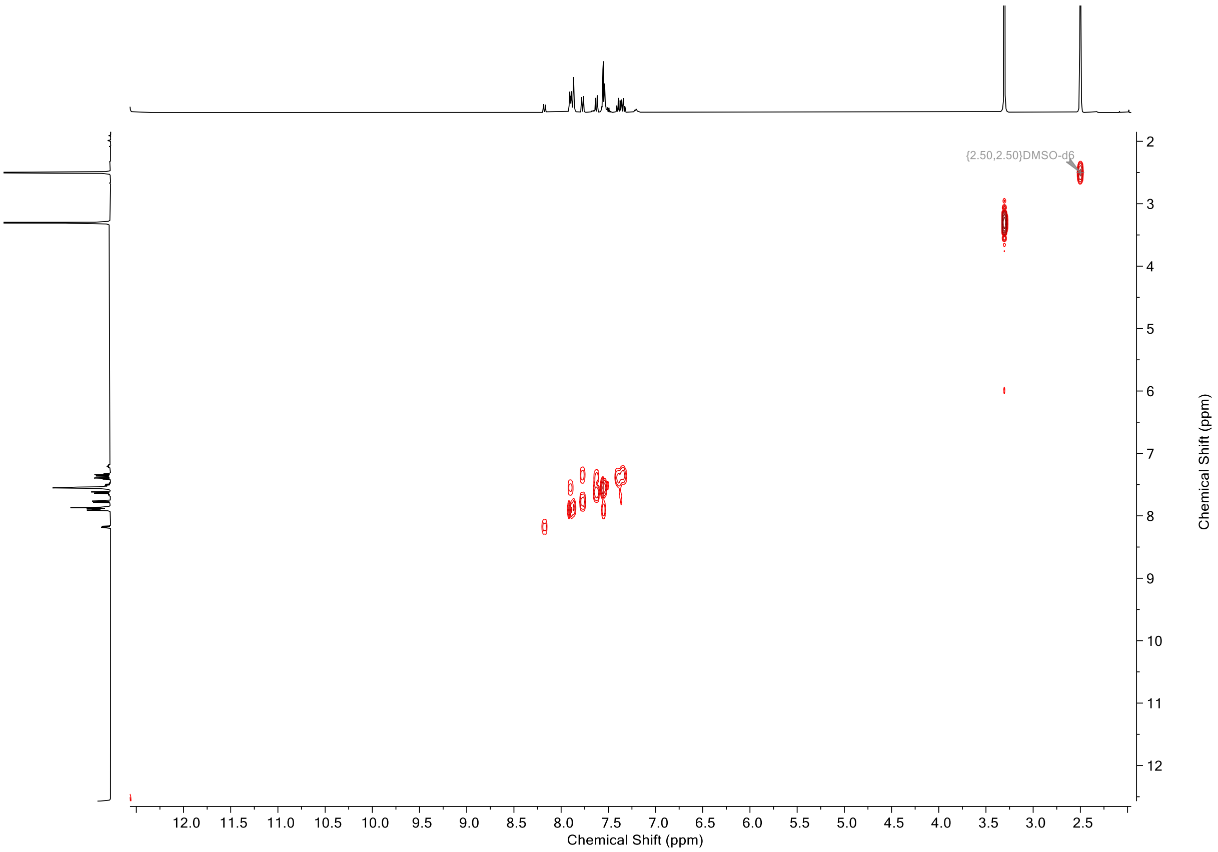

Figure S138: COSY (DMSO-d_6): 1*H*-2,4,6-Trichloro-*N*-(2-phenyl-benzo[*d*]imidazol-1-yl)benzamide (**2j**).

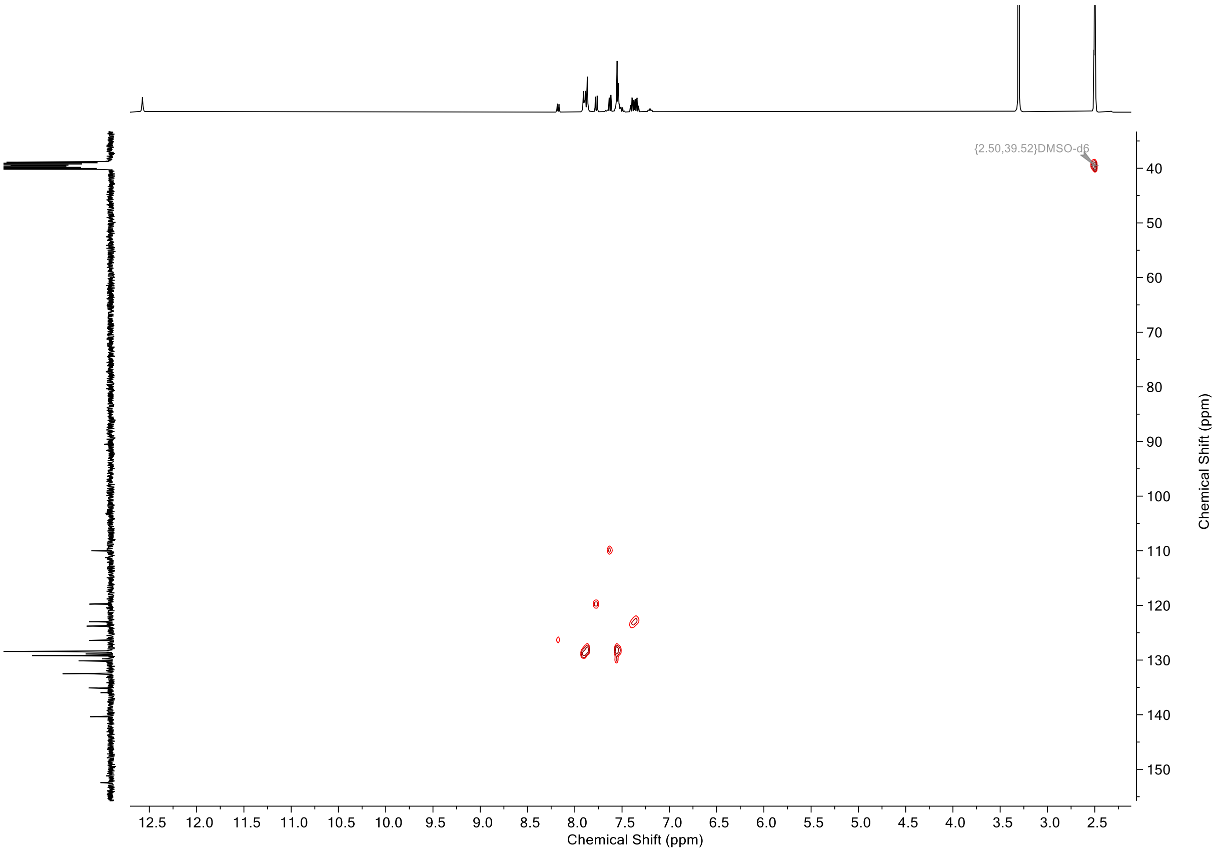

Figure S139: HSQC (DMSO-d_6): 1*H*-2,4,6-Trichloro-*N*-(2-phenyl-benzo[*d*]imidazol-1-yl)benzamide (**2j**).

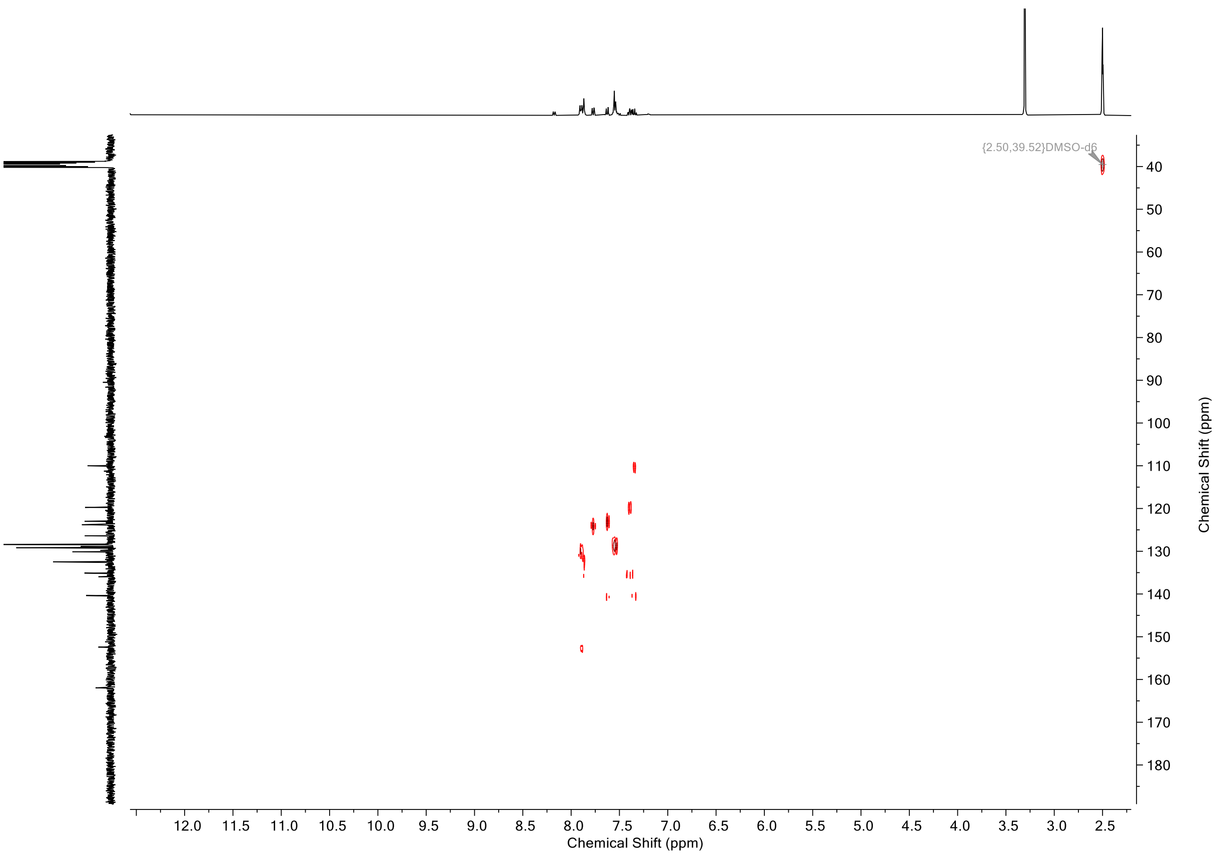

Figure S140: HMBC (DMSO-d_6): 1*H*-2,4,6-Trichloro-*N*-(2-phenyl-benzo[*d*]imidazol-1-yl)benzamide (**2j**).

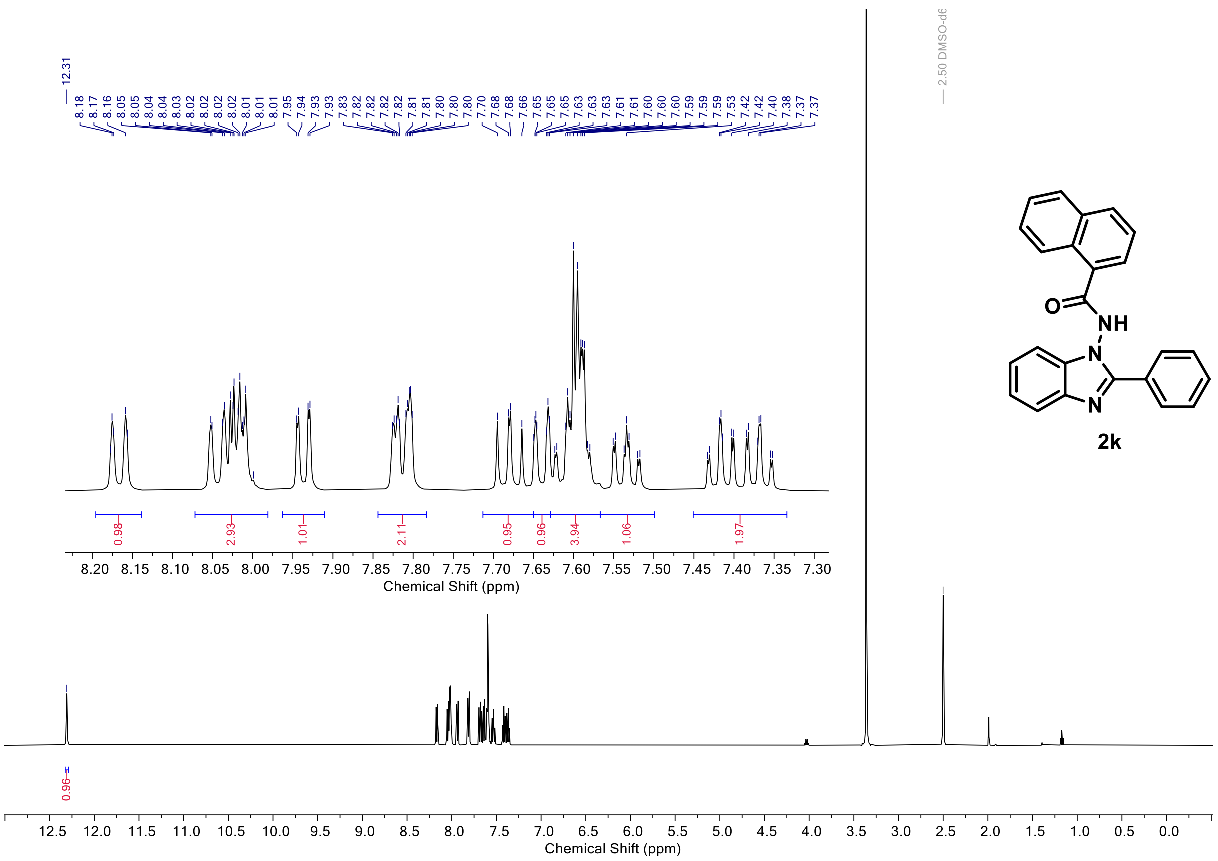

Figure S141: ^{1}H NMR (500 MHz, DMSO-d_6): 1*H*-*N*-(2-Phenyl-benzo[*d*]imidazol-1-yl)-1-naphthamide (**2k**).

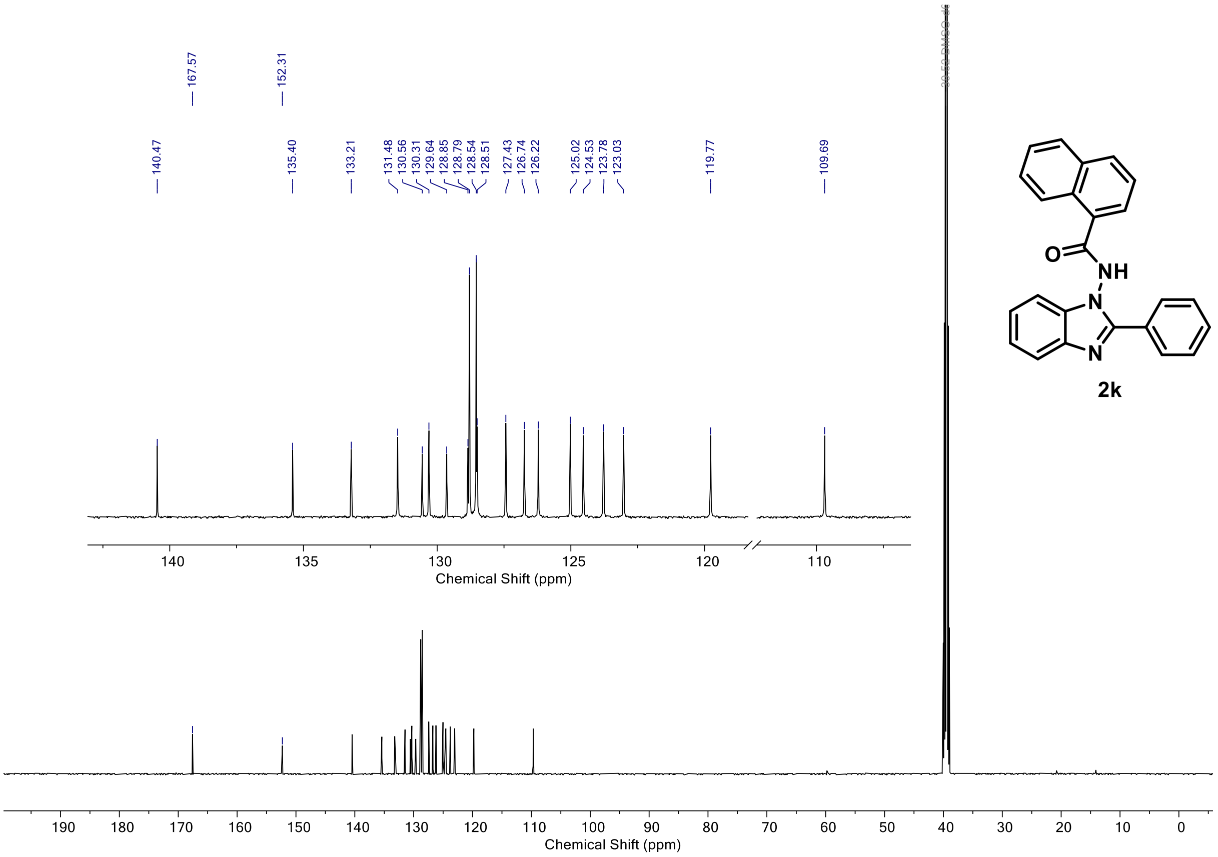

Figure S142: ^{13}C{^{1}H} NMR (126 MHz, DMSO-d_6): 1*H*-*N*-(2-Phenyl-benzo[*d*]imidazol-1-yl)-1-naphthamide (**2k**).

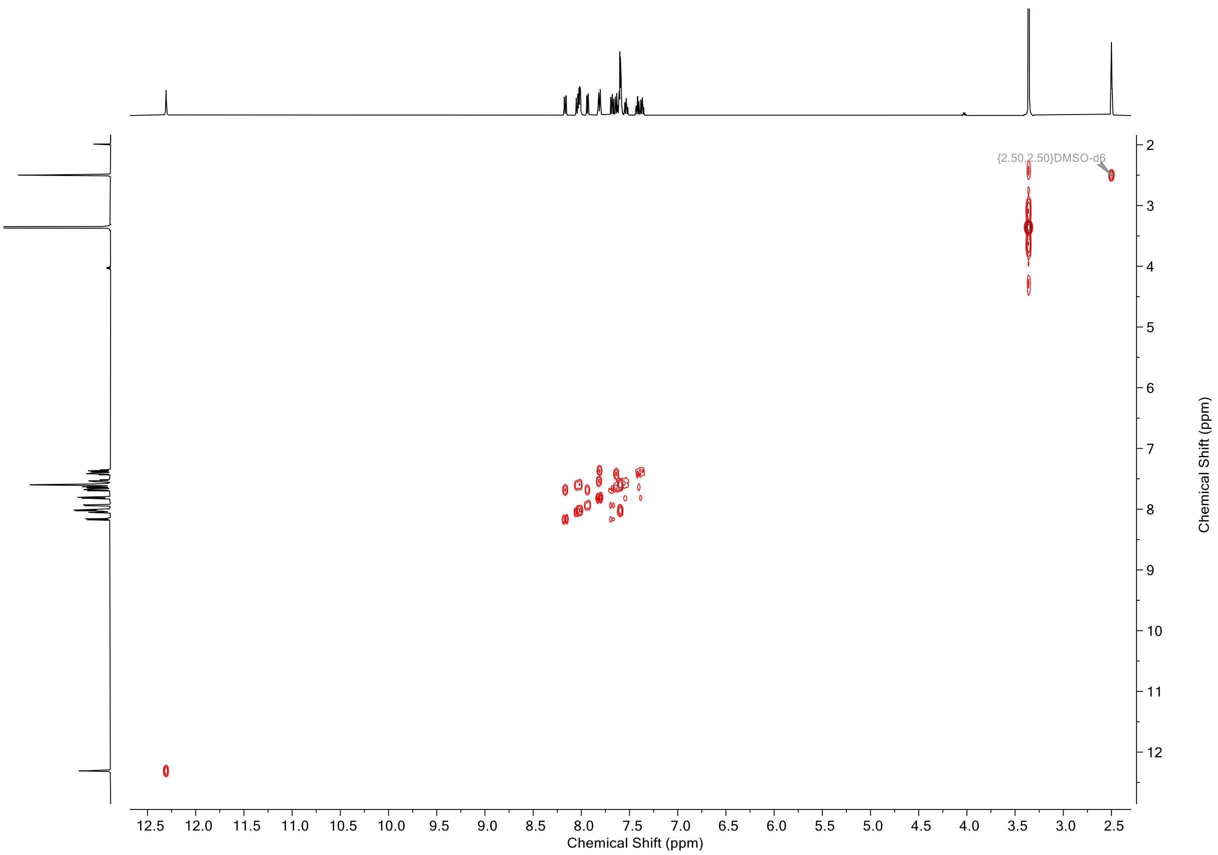

Figure S143: COSY (DMSO-d_6): 1*H*-*N*-(2-Phenyl-benzo[*d*]imidazol-1-yl)-1-naphthamide (**2k**).

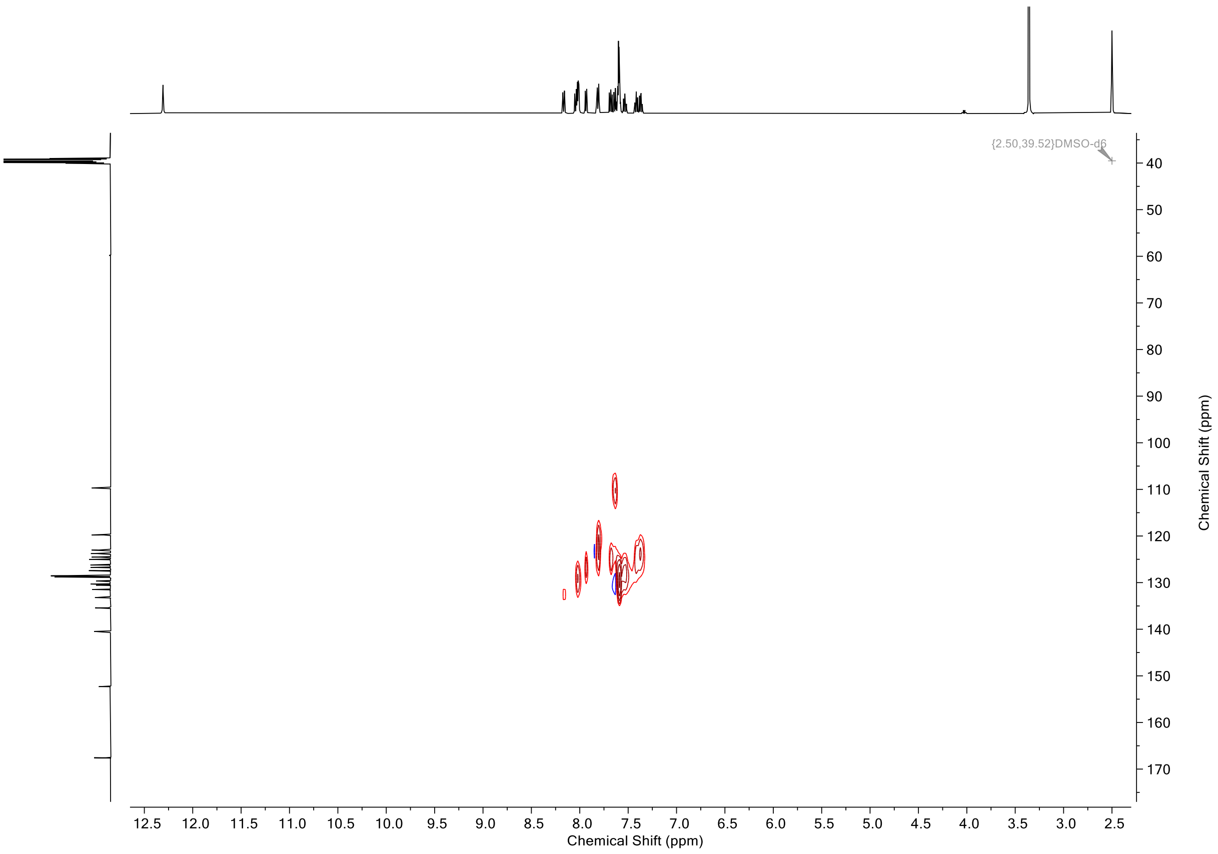

Figure S144: HSQC (DMSO-d_6): 1*H*-*N*-(2-Phenyl-benzo[*d*]imidazol-1-yl)-1-naphthamide (**2k**).

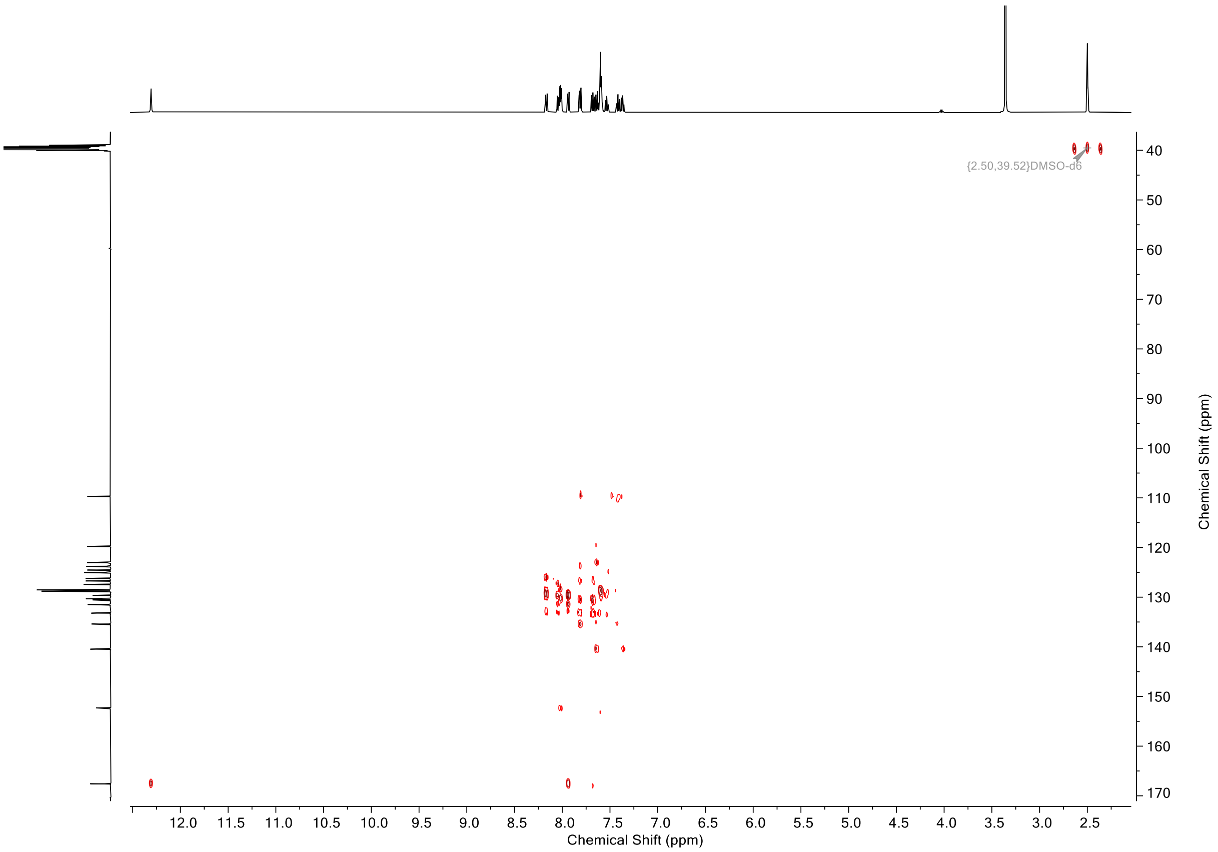

Figure S145: HMBC (DMSO-d_6): 1*H*-*N*-(2-Phenyl-benzo[*d*]imidazol-1-yl)-1-naphthamide (**2k**).

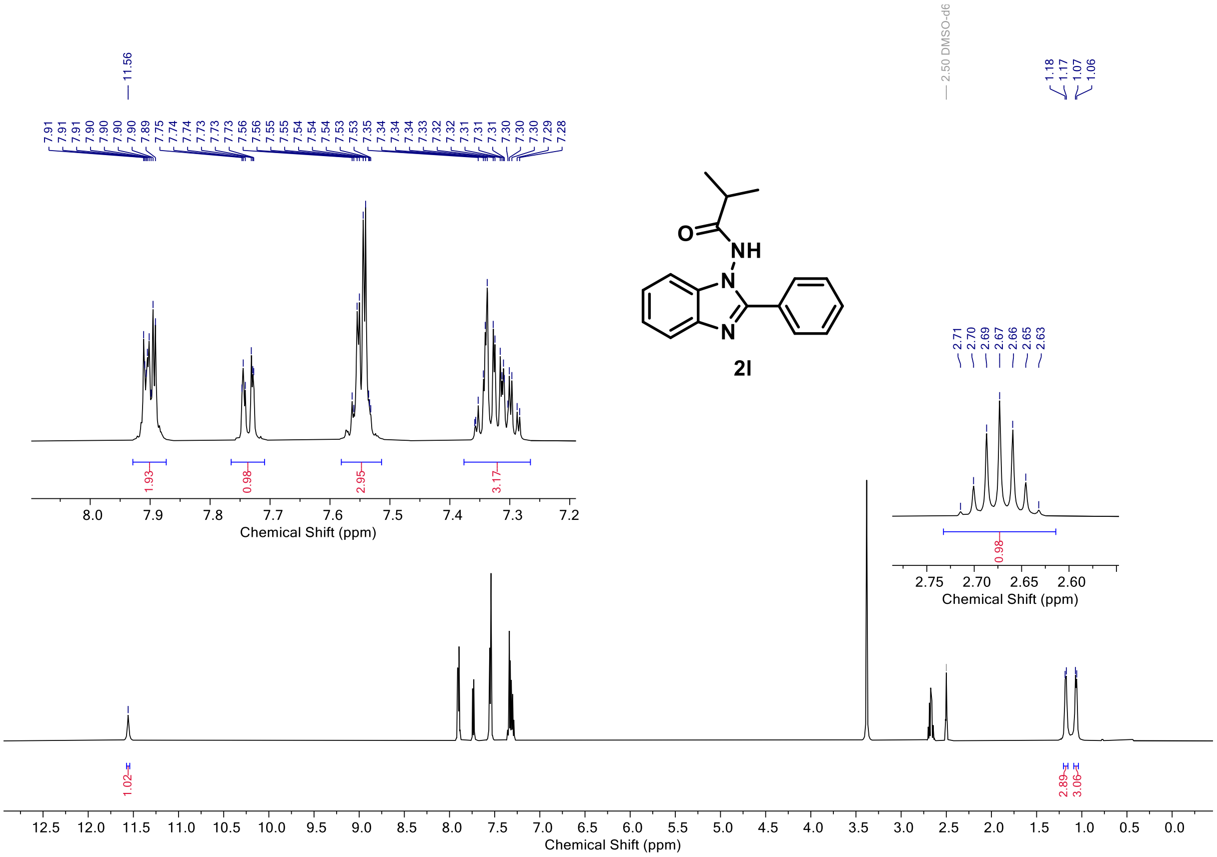

Figure S146: ^{1}H NMR (500 MHz, DMSO-d_6): 1*H*-*N*-(2-Phenyl-benzo[*d*]imidazol-1-yl)isobutyramide (**2l**).

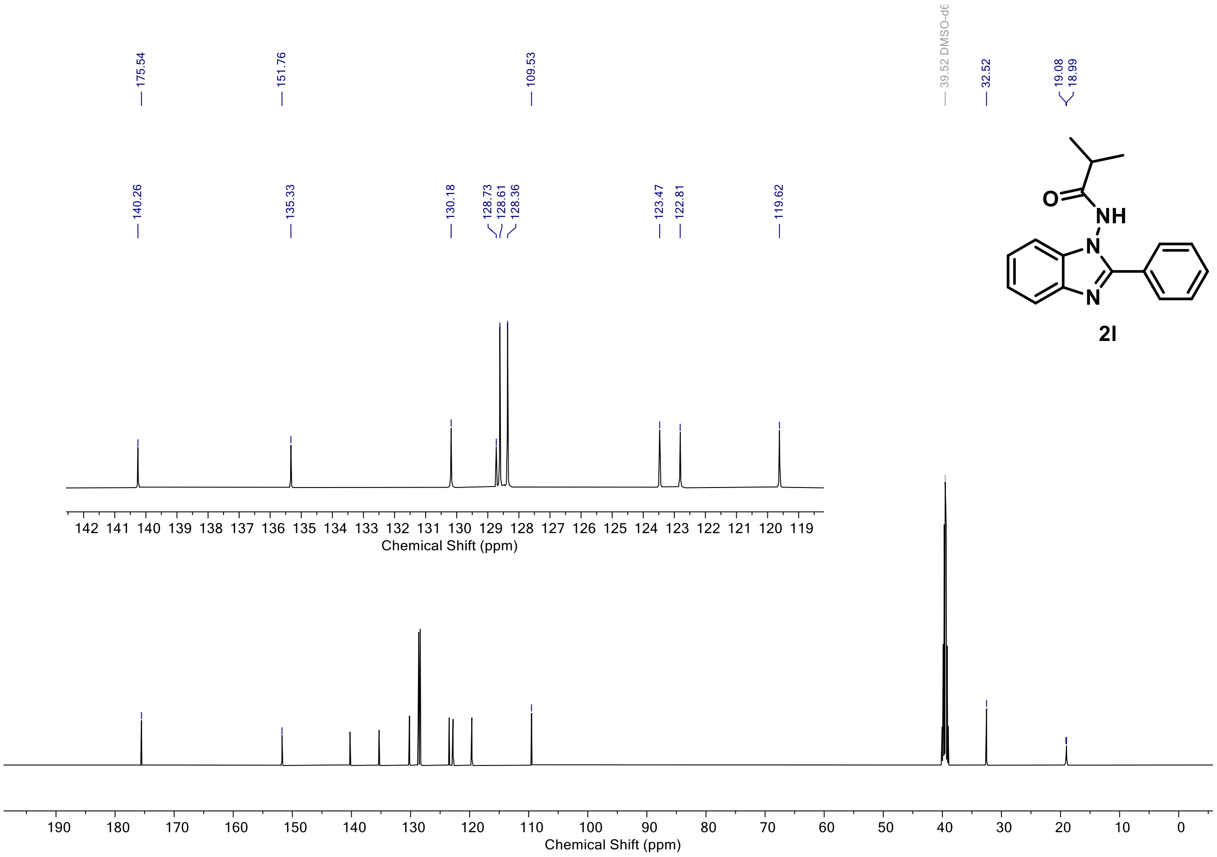

Figure S147: ^{13}C{^{1}H} NMR (126 MHz, DMSO-d_6): 1*H*-*N*-(2-Phenyl-benzo[*d*]imidazol-1-yl)isobutyramide (**2l**).

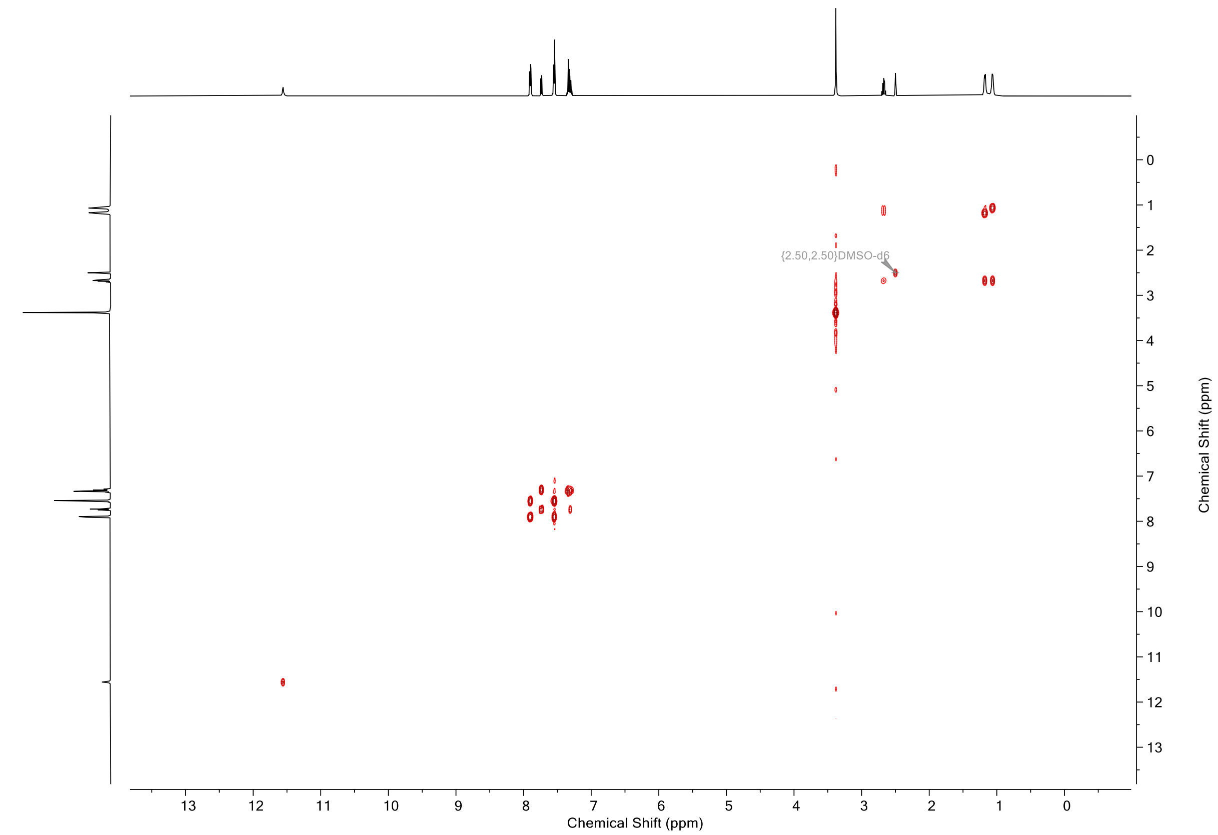

Figure S148: COSY (DMSO-d_6): 1*H*-*N*-(2-Phenyl-benzo[*d*]imidazol-1-yl)isobutyramide (**2l**).

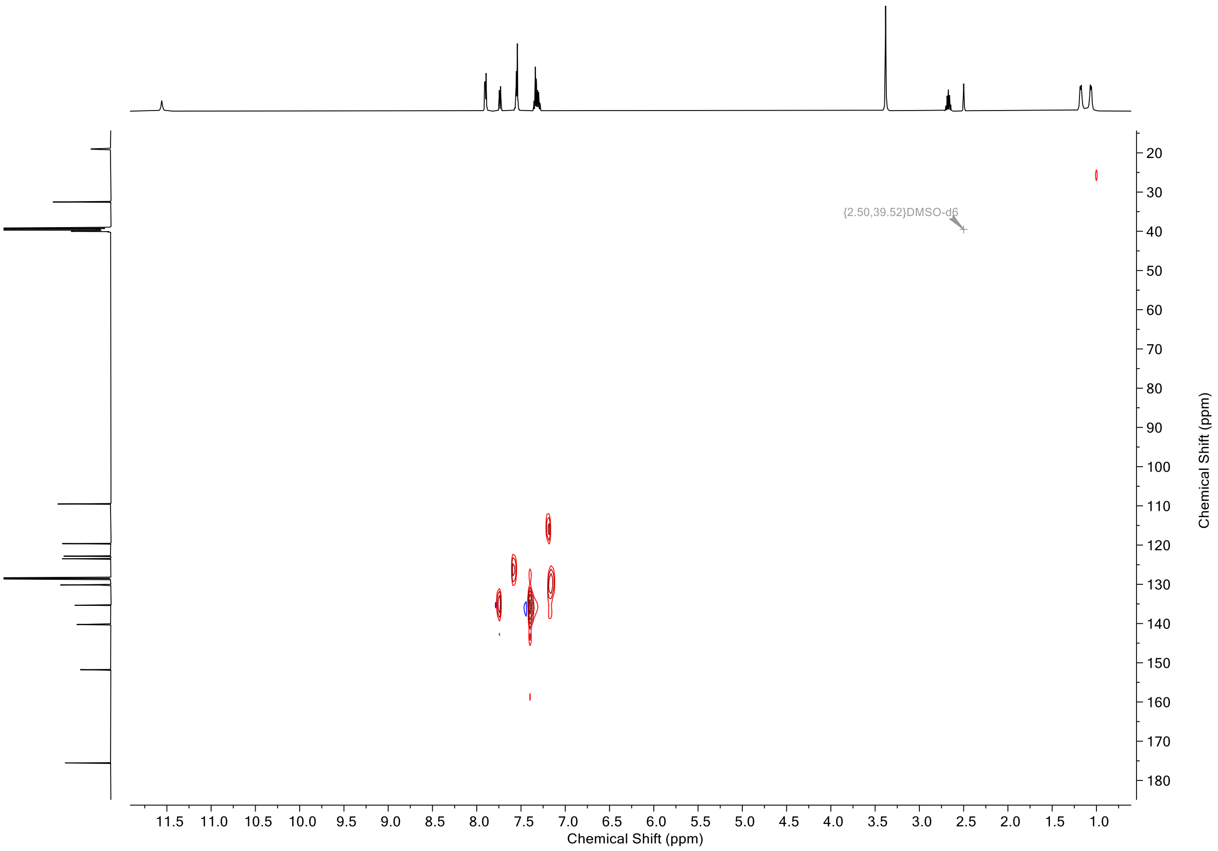

Figure S149: HSQC (DMSO-d_6): 1*H*-*N*-(2-Phenyl-benzo[*d*]imidazol-1-yl)isobutyramide (**2l**).

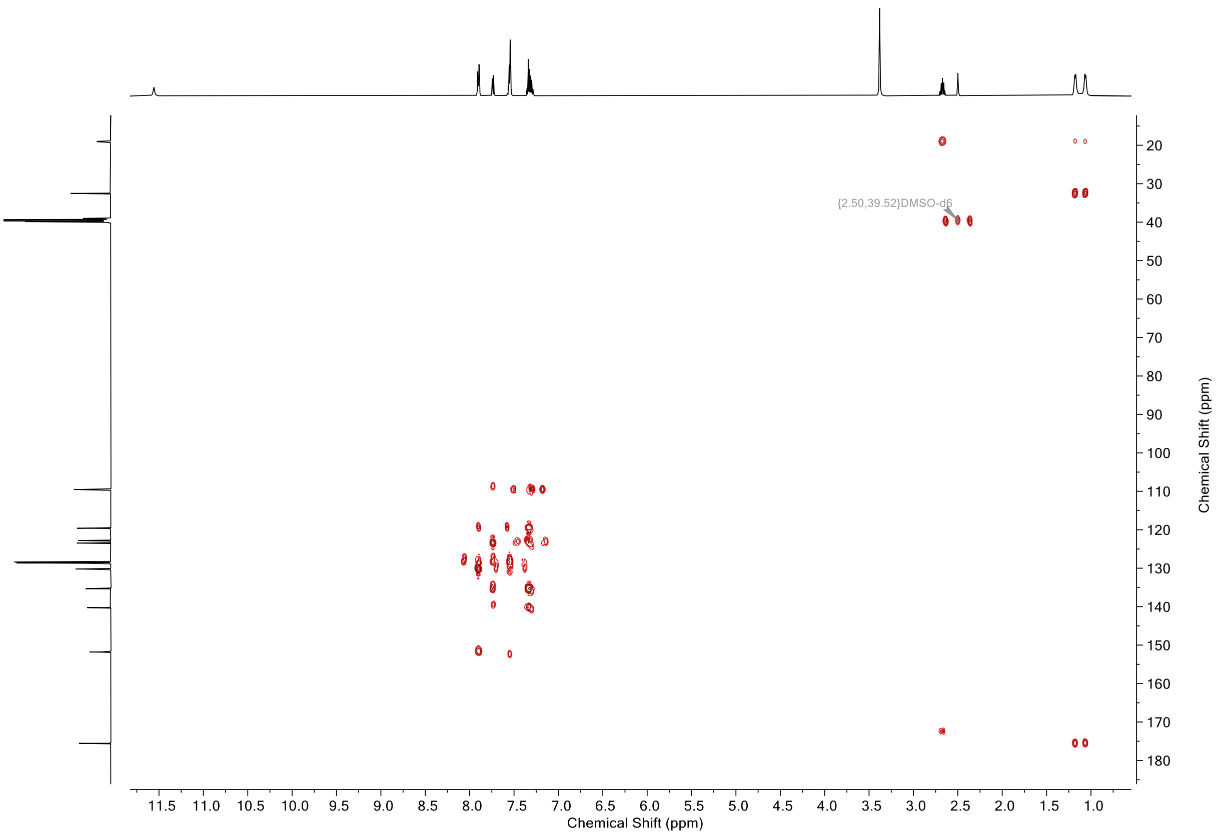

Figure S150: HMBC (DMSO-d_6): 1*H*-*N*-(2-Phenyl-benzo[*d*]imidazol-1-yl)isobutyramide (**2l**).

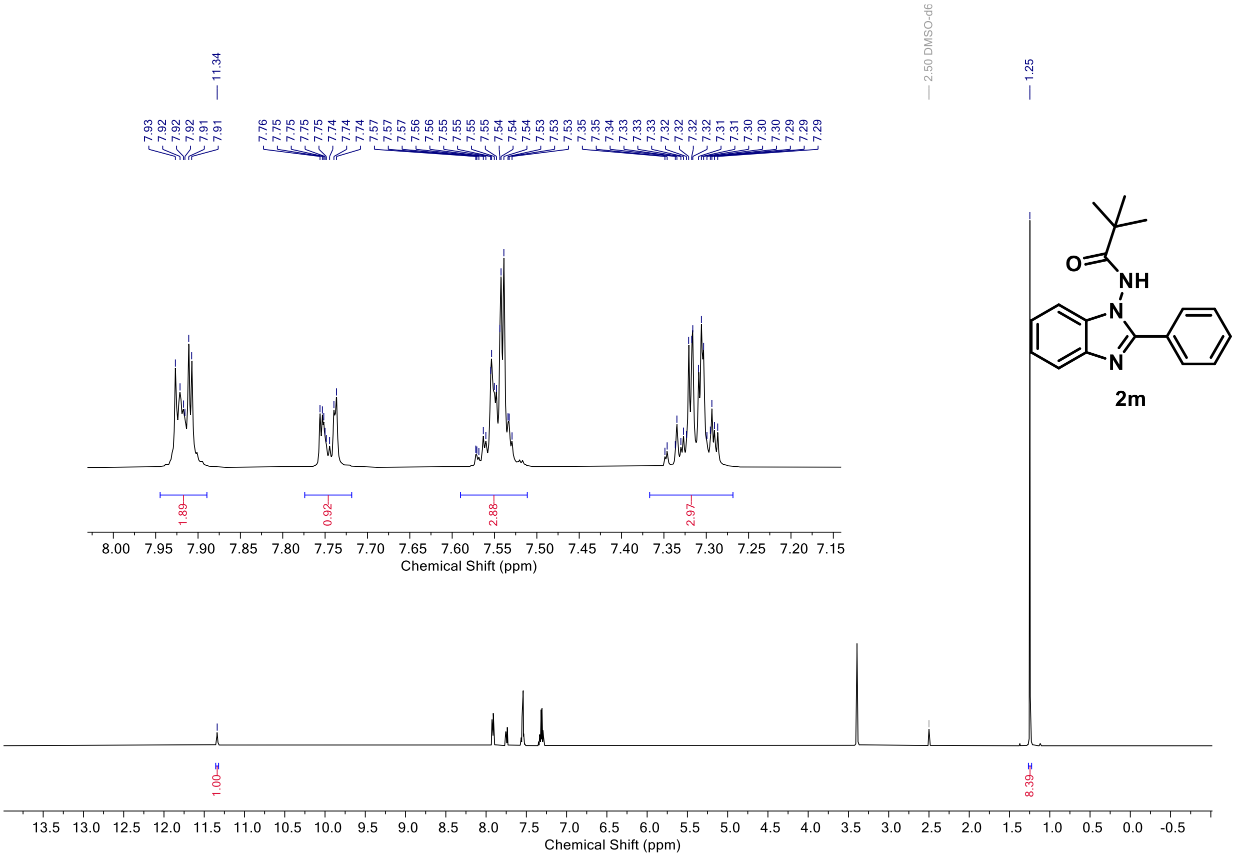

Figure S151: ^{1}H NMR (500 MHz, DMSO-d_6): 1*H*-*N*-(2-Phenyl-benzo[*d*]imidazol-1-yl)pivalamide (**2m**).

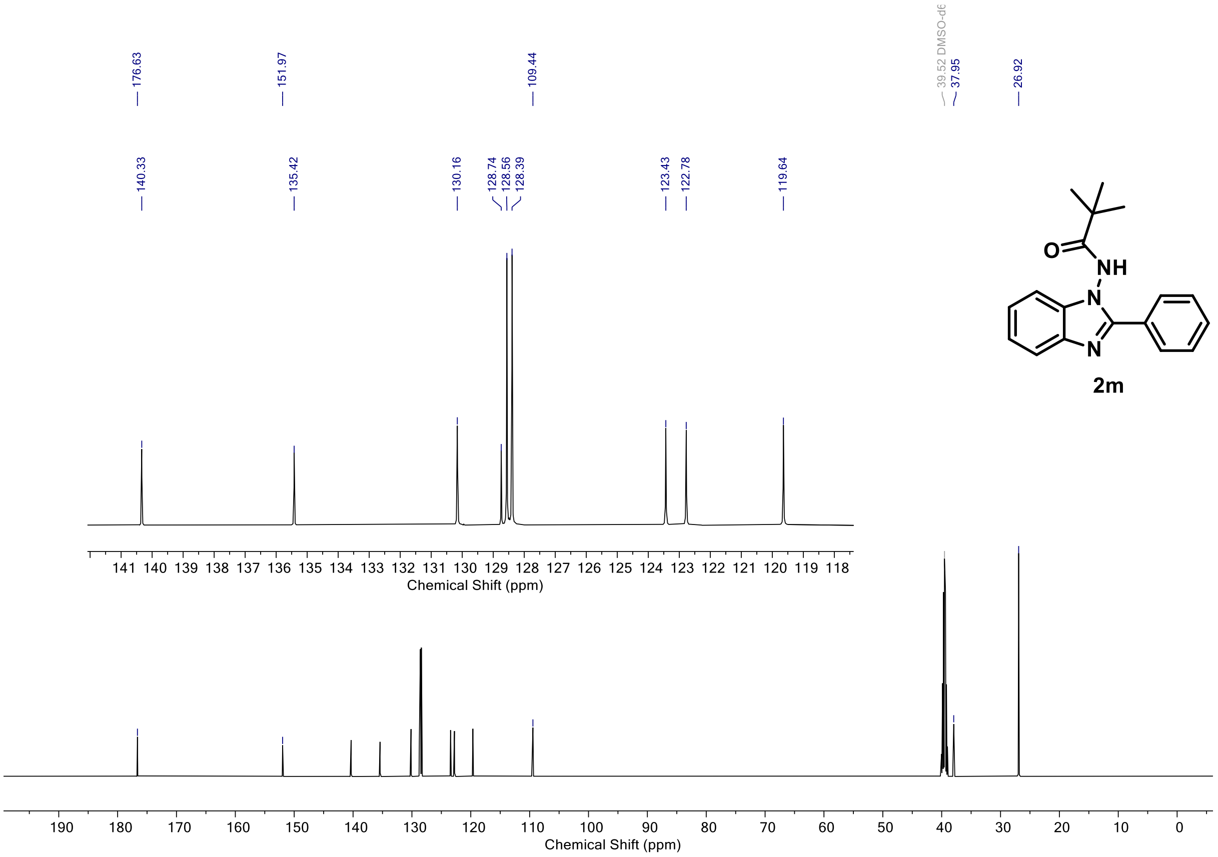

Figure S152: ^{13}C{^{1}H} NMR (126 MHz, DMSO-d_6): 1*H*-*N*-(2-Phenyl-benzo[*d*]imidazol-1-yl)pivalamide (**2m**).

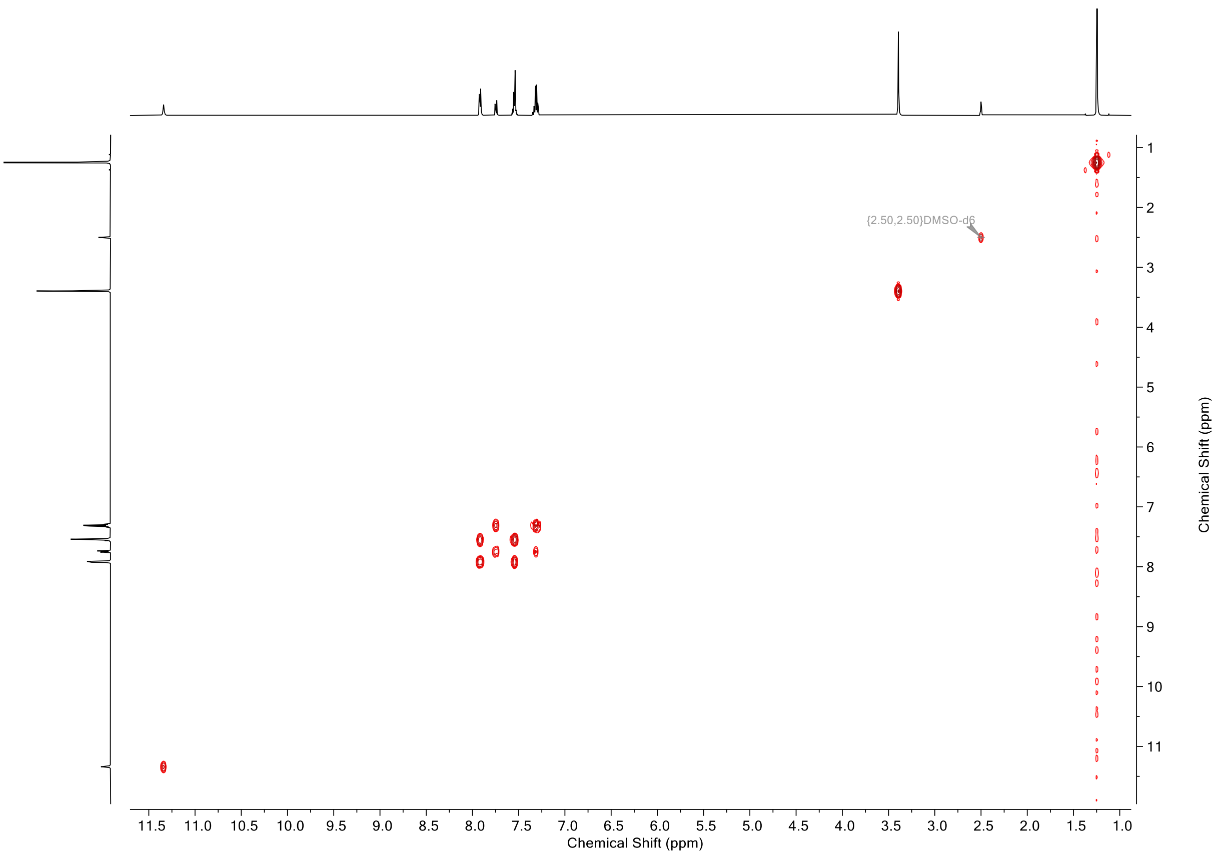

Figure S153: COSY (DMSO-d_6): 1*H*-*N*-(2-Phenyl-benzo[*d*]imidazol-1-yl)pivalamide (**2m**).

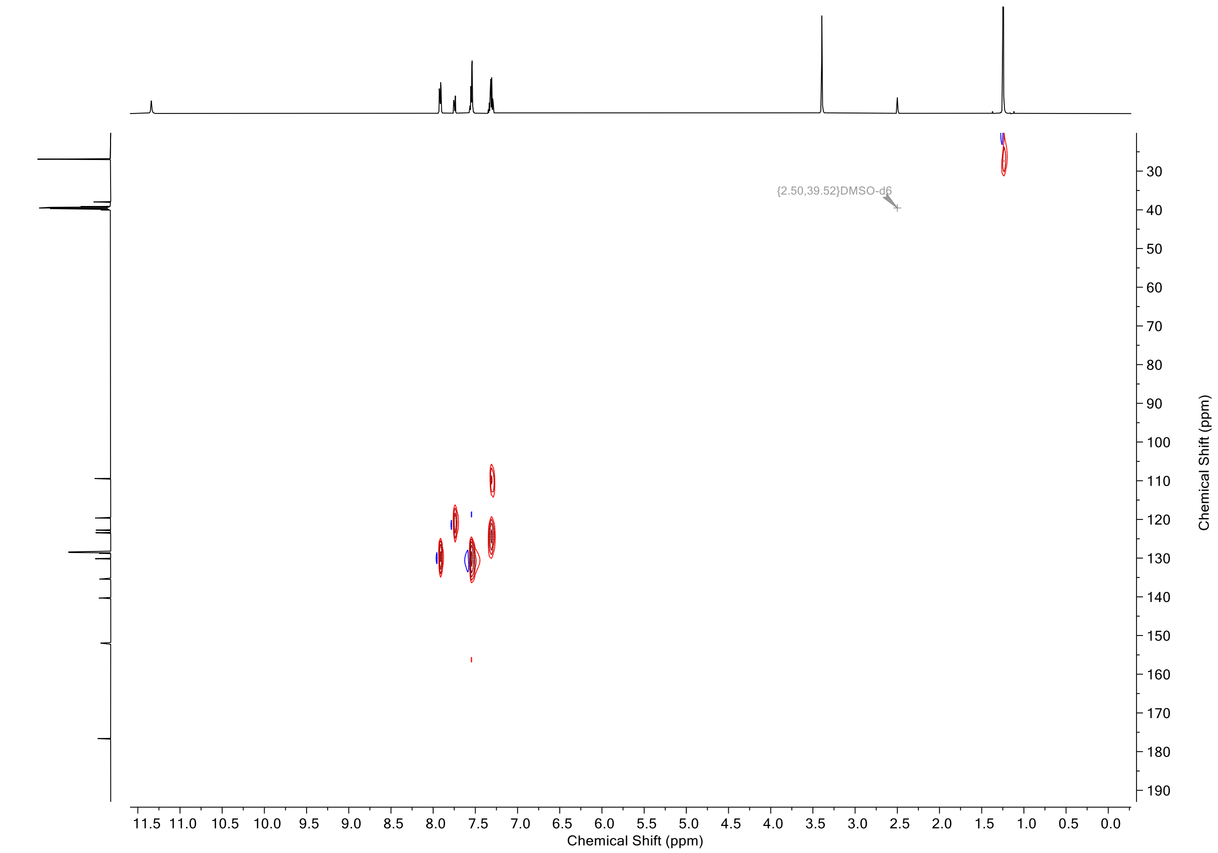

Figure S154: HSQC (DMSO-d_6): 1*H*-*N*-(2-Phenyl-benzo[*d*]imidazol-1-yl)pivalamide (**2m**).

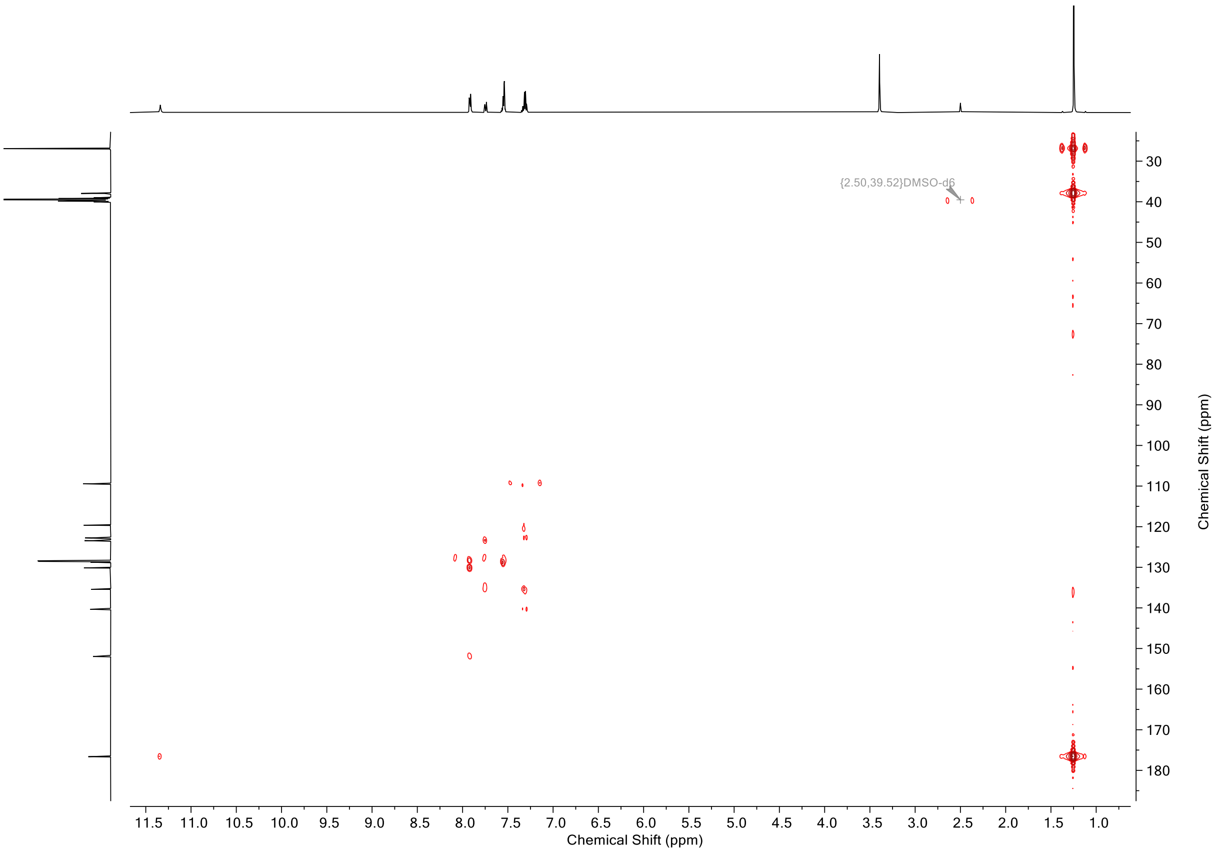

Figure S155: HMBC (DMSO-d_6): 1*H*-*N*-(2-Phenyl-benzo[*d*]imidazol-1-yl)pivalamide (**2m**).

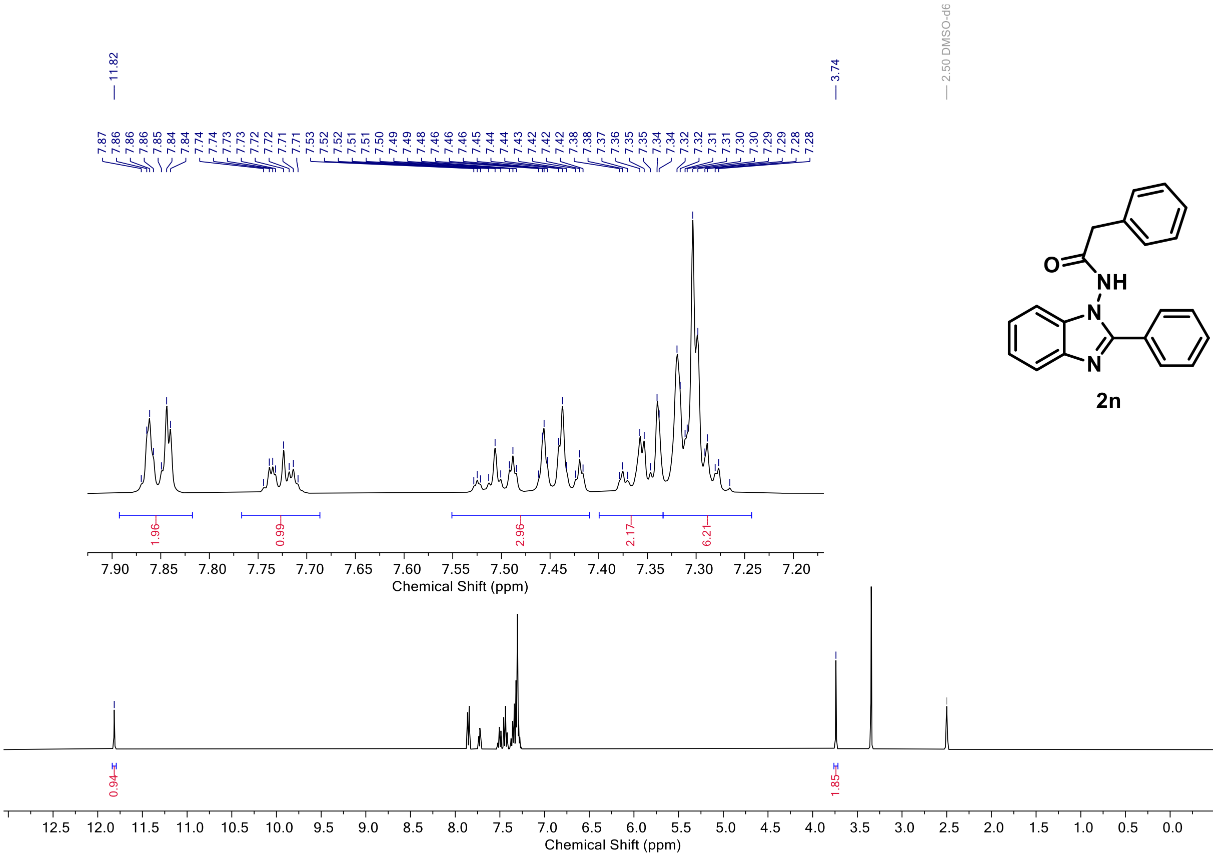

Figure S156: ^{1}H NMR (400 MHz, DMSO-d_6): 1*H*-2-Phenyl-*N*-(2-phenyl-benzo[*d*]imidazol-1-yl)acetamide (**2n**).

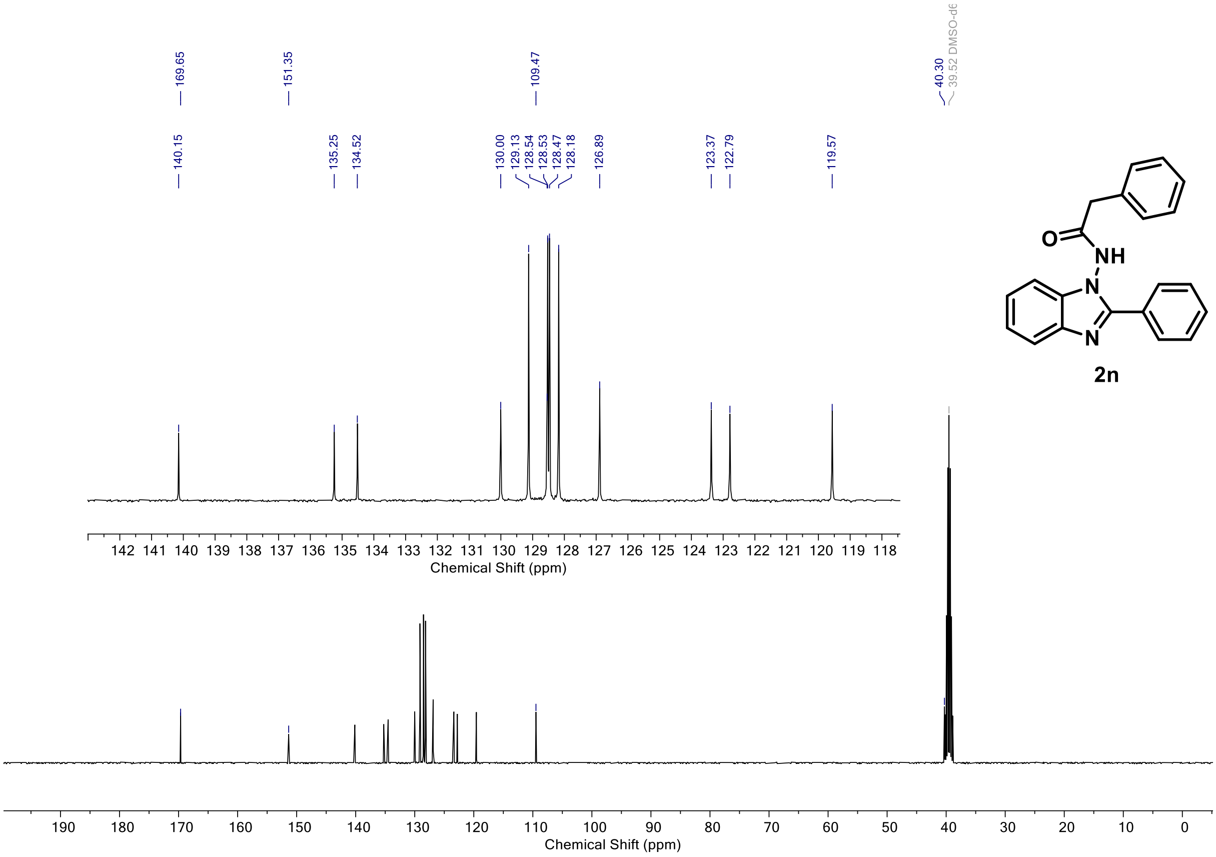

Figure S157: ^{13}C{^{1}H} NMR (101 MHz, DMSO-d_6): 1*H*-2-Phenyl-*N*-(2-phenyl-benzo[*d*]imidazol-1-yl)acetamide (**2n**).

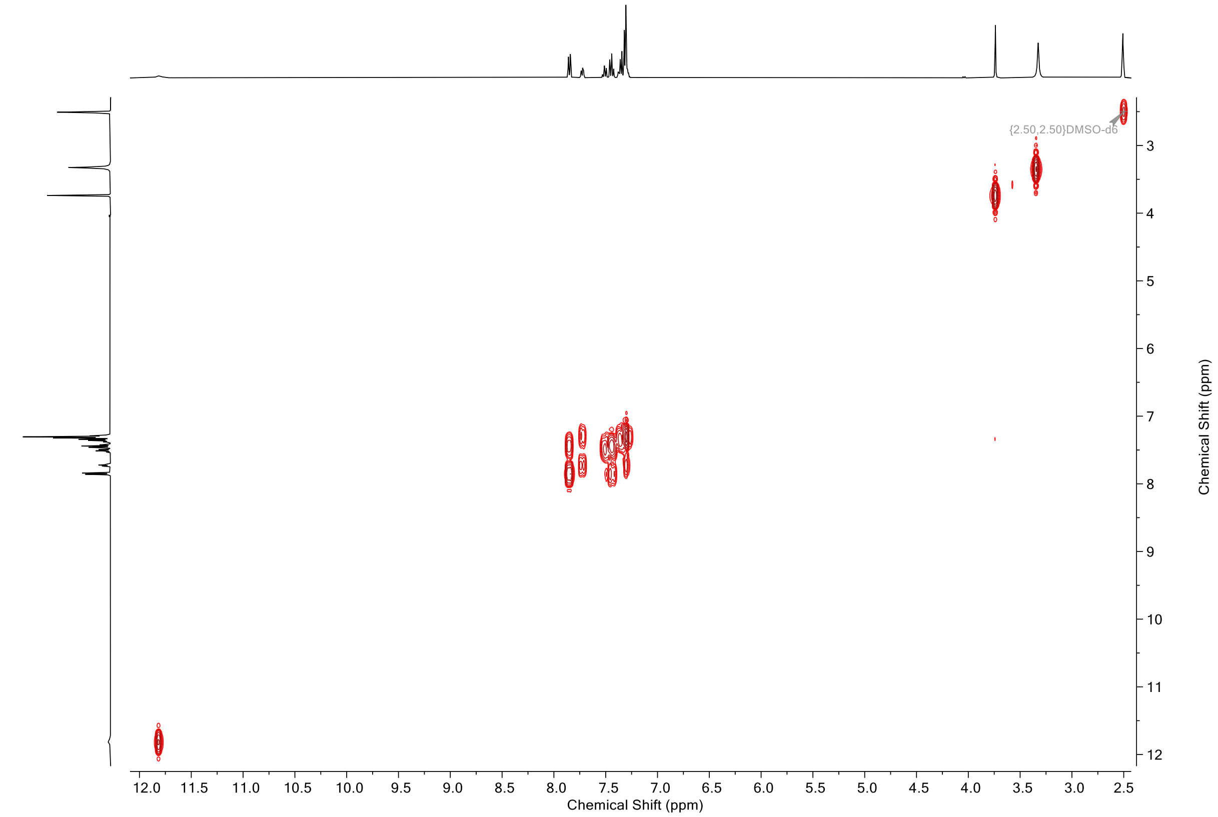

Figure S158: COSY (DMSO-d_6): 1*H*-2-Phenyl-*N*-(2-phenyl-benzo[*d*]imidazol-1-yl)acetamide (**2n**).

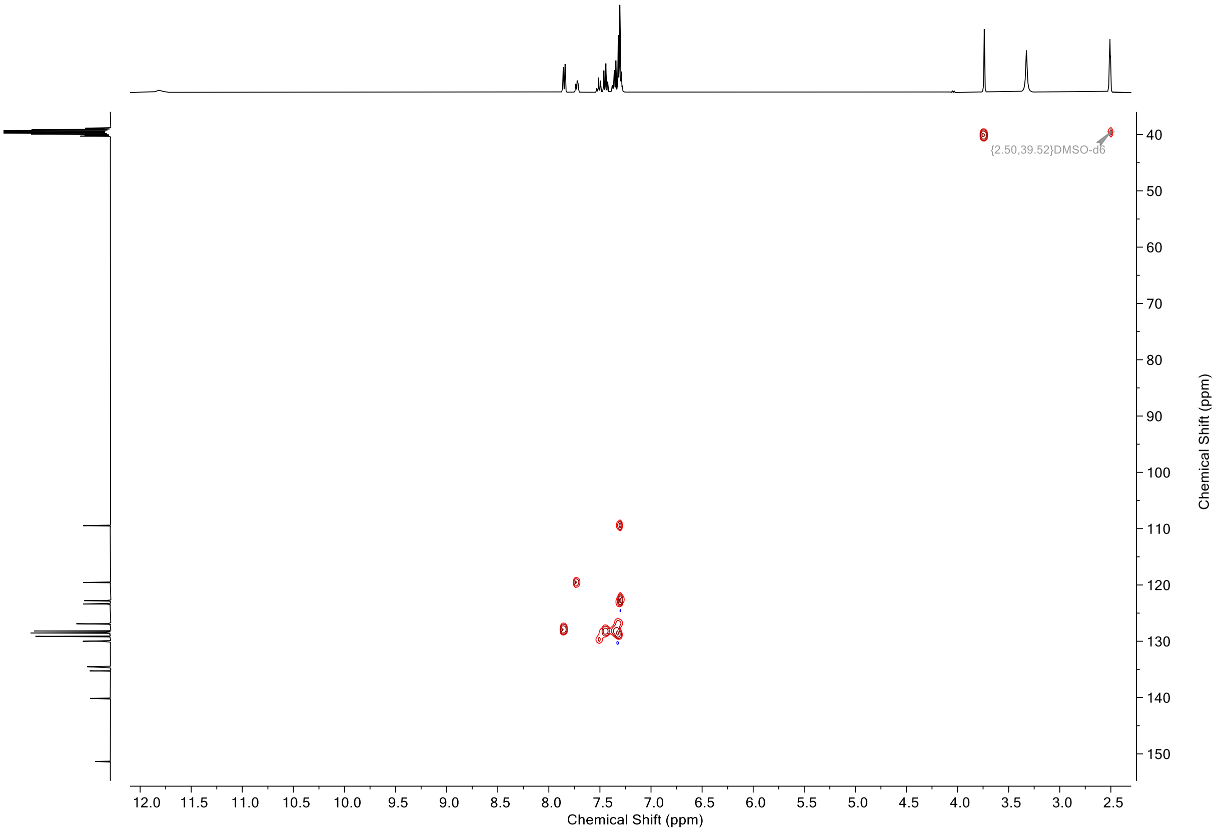

Figure S159: HSQC (DMSO-d_6): 1*H*-2-Phenyl-*N*-(2-phenyl-benzo[*d*]imidazol-1-yl)acetamide (**2n**).

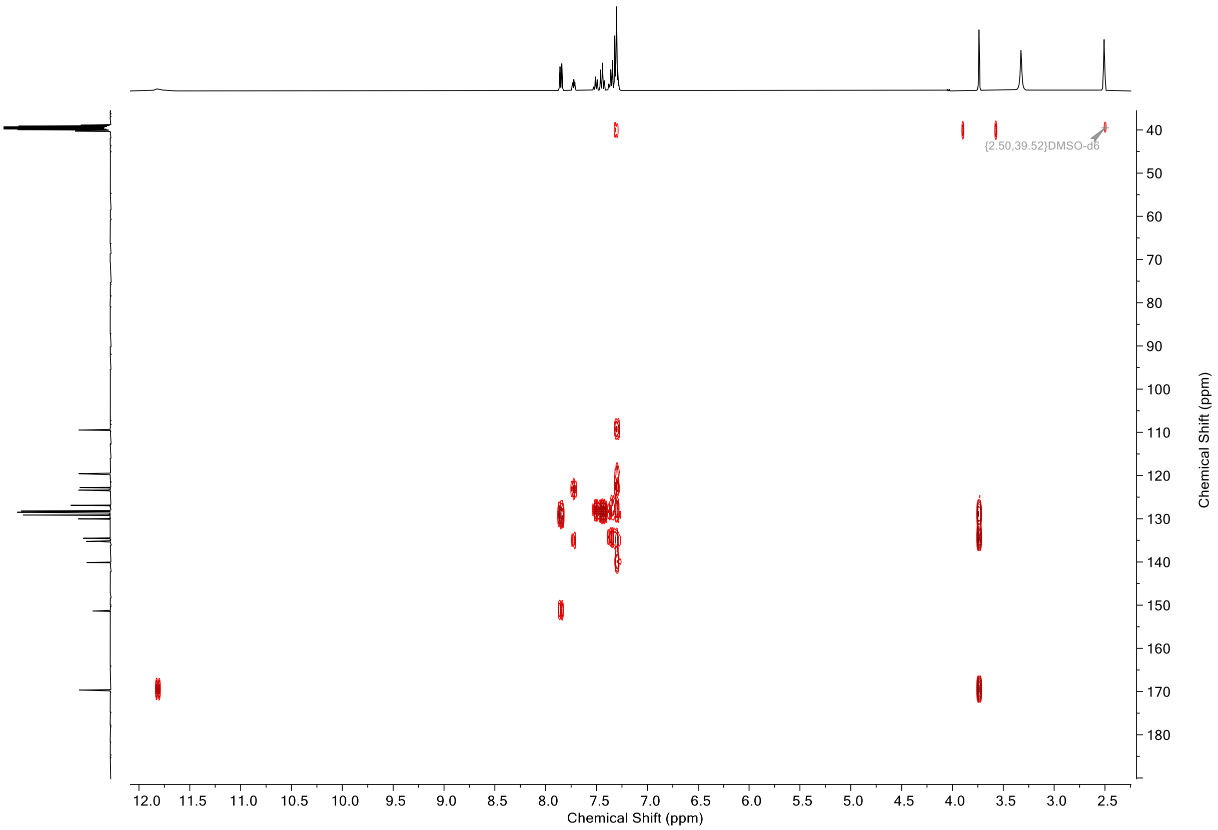

Figure S160: HMBC (DMSO-d_6): 1*H*-2-Phenyl-*N*-(2-phenyl-benzo[*d*]imidazol-1-yl)acetamide (**2n**).

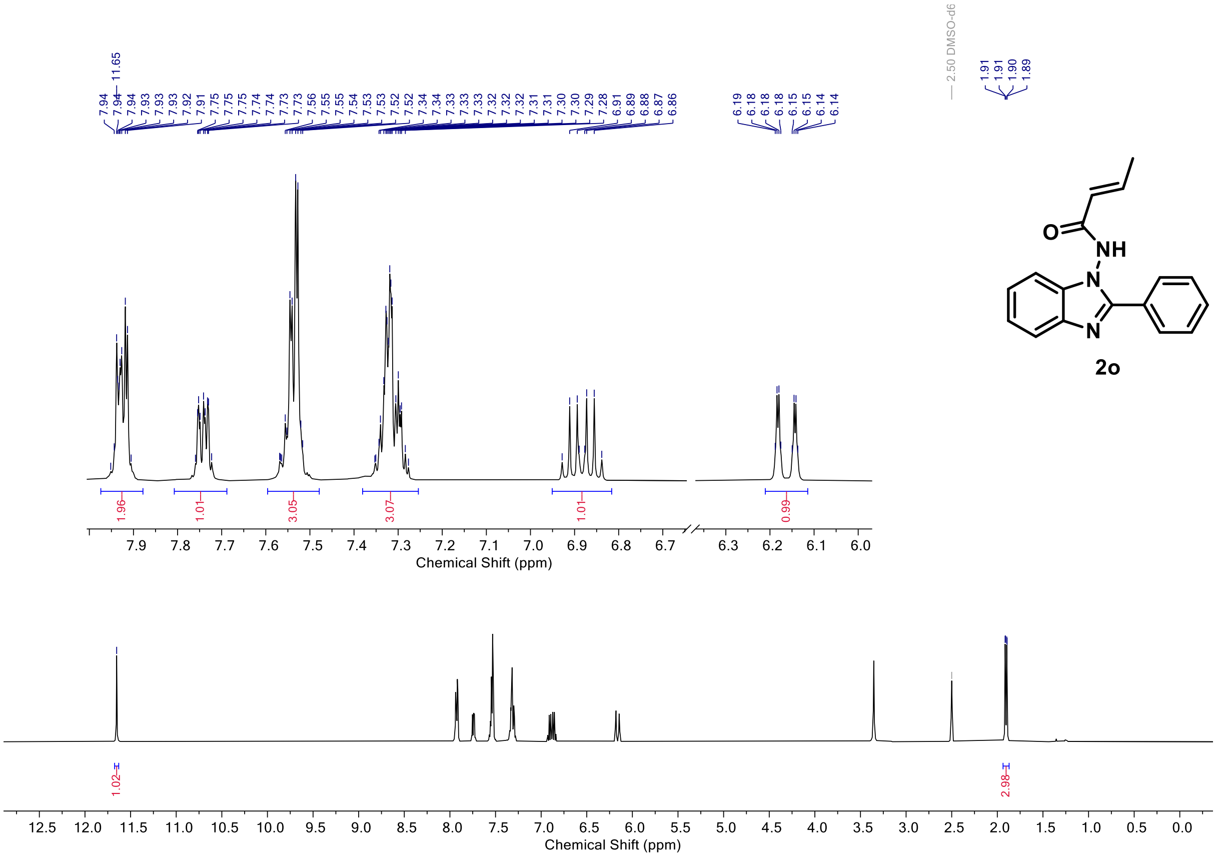

Figure S161: ^{1}H NMR (400 MHz, DMSO-d_6): 1*H*-(*E*)-*N*-(2-Phenyl-benzo[*d*]imidazol-1-yl)but-2-enamide (**2o**).

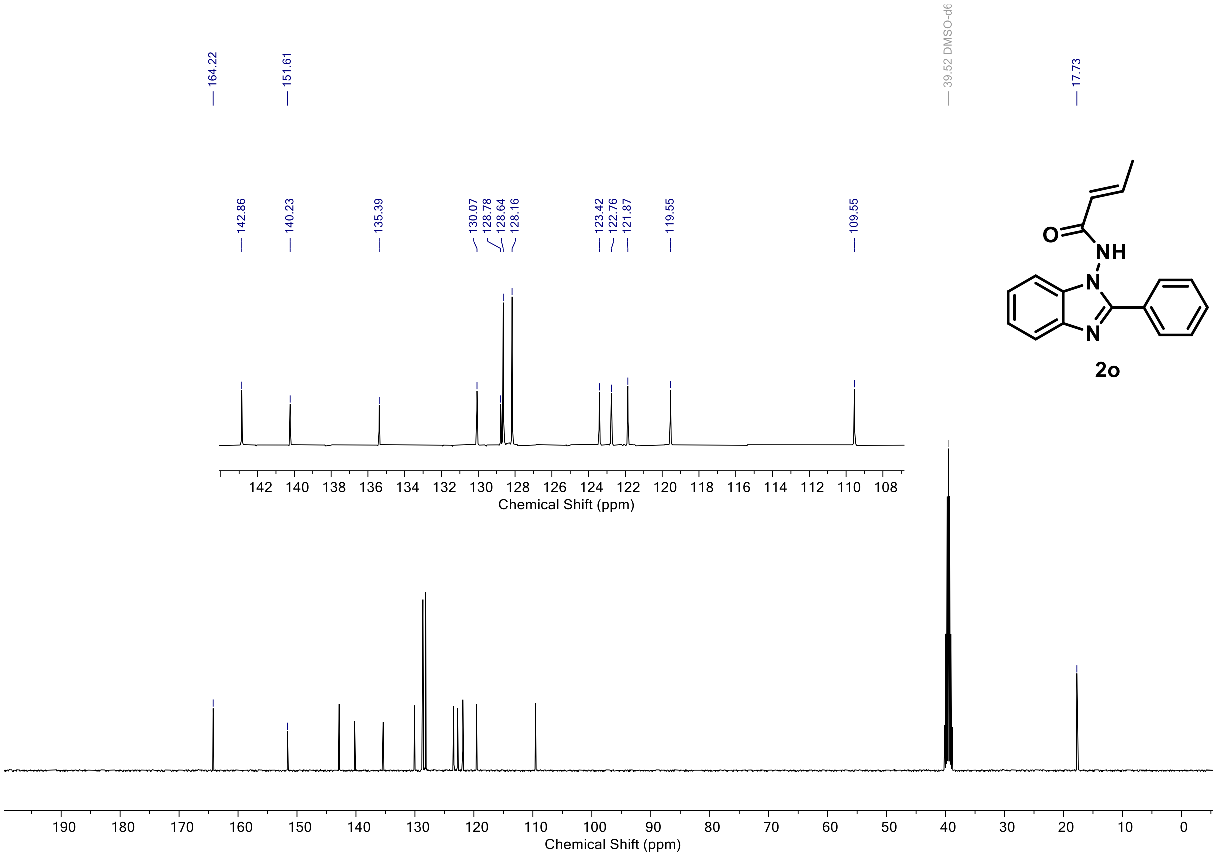

Figure S162: ^{13}C{^{1}H} NMR (101 MHz, DMSO-d_6): 1*H*-(*E*)-*N*-(2-Phenyl-benzo[*d*]imidazol-1-yl)but-2-en-amide (**2o**).

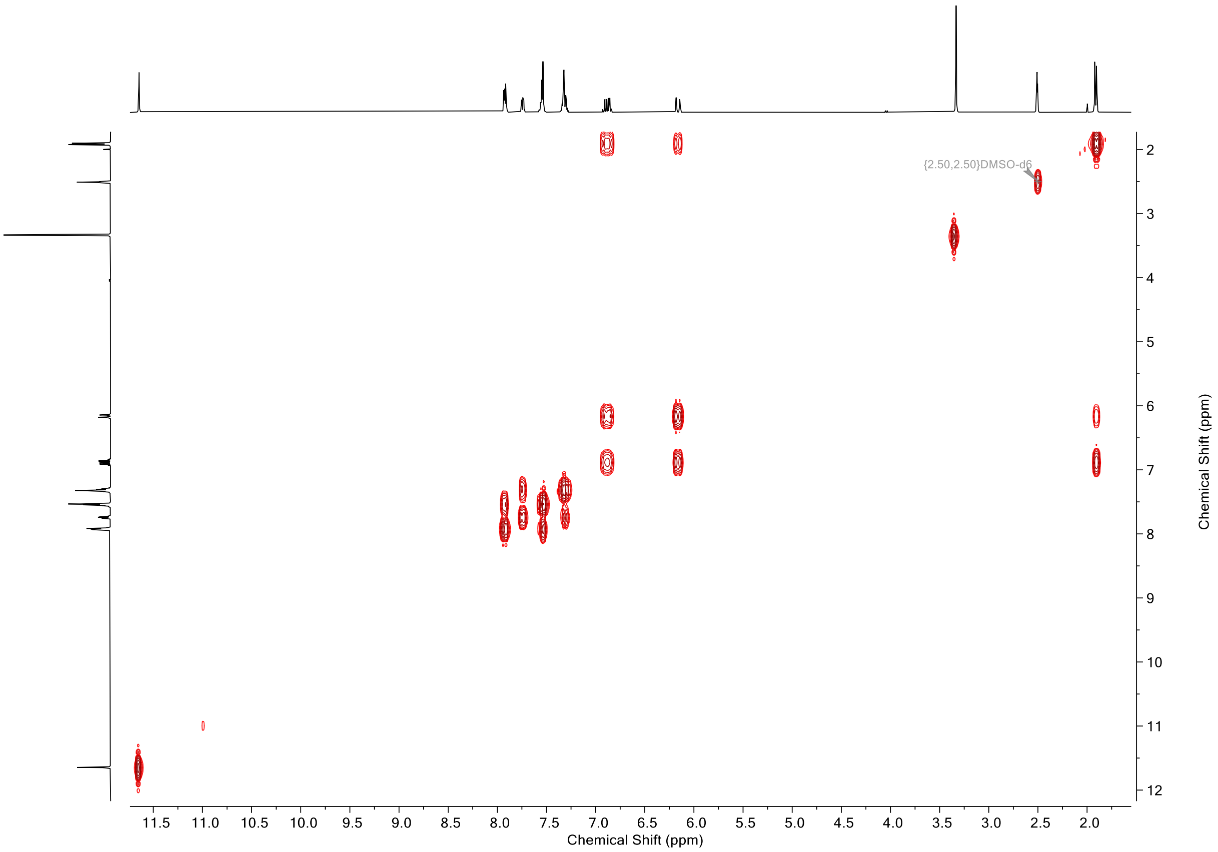

Figure S163: COSY (DMSO-d_6): 1*H*-(*E*)-*N*-(2-Phenyl-benzo[*d*]imidazol-1-yl)but-2-enamide (**2o**).

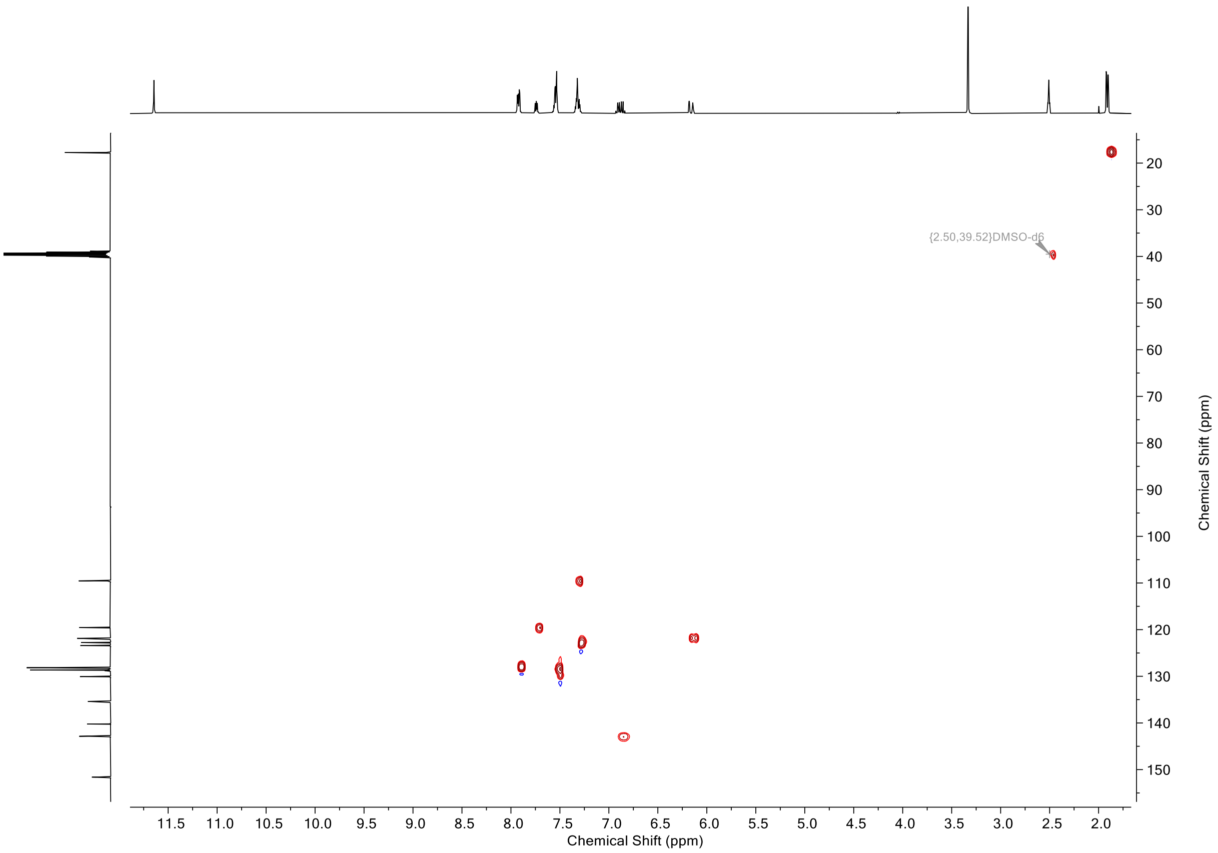

Figure S164: HSQC (DMSO-d_6): 1*H*-(*E*)-*N*-(2-Phenyl-benzo[*d*]imidazol-1-yl)but-2-enamide (**2o**).

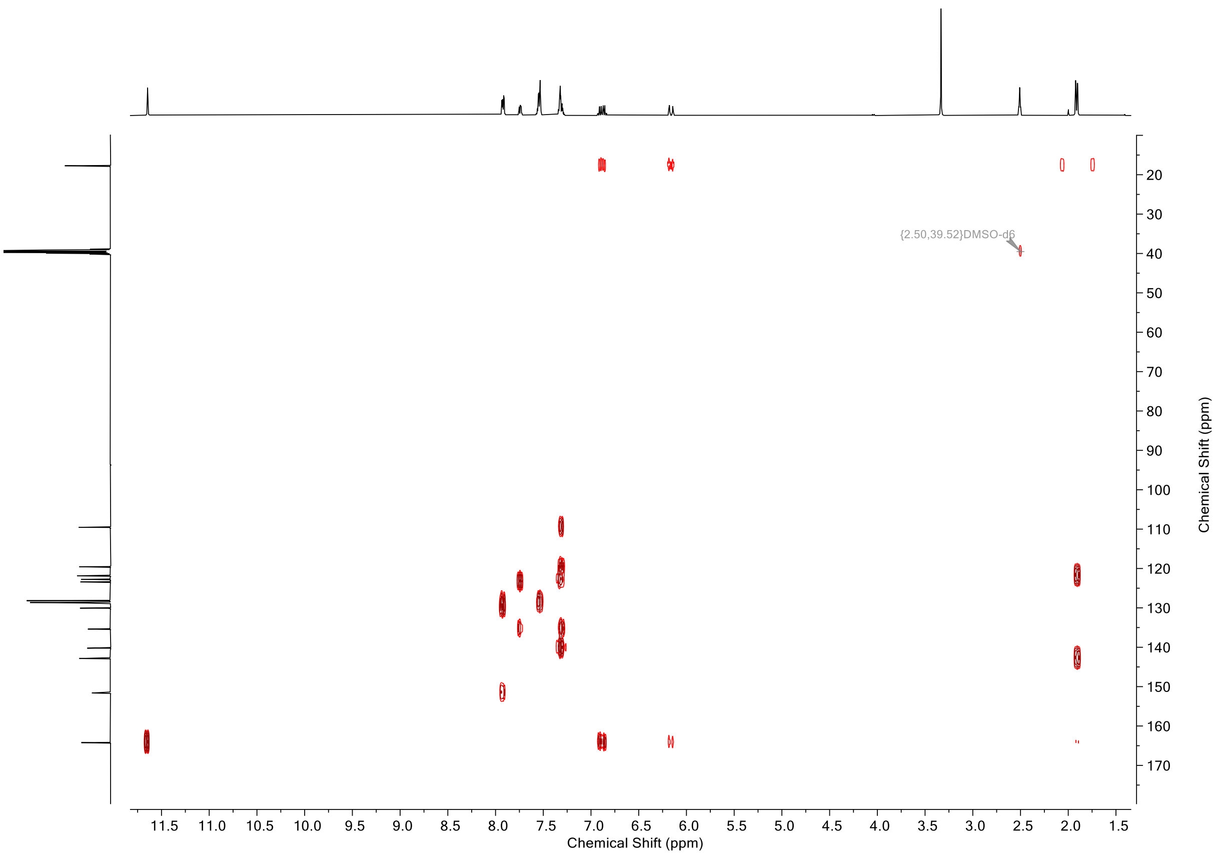

Figure S165: HMBC (DMSO-d_6): 1*H*-(*E*)-*N*-(2-Phenyl-benzo[*d*]imidazol-1-yl)but-2-enamide (**2o**).

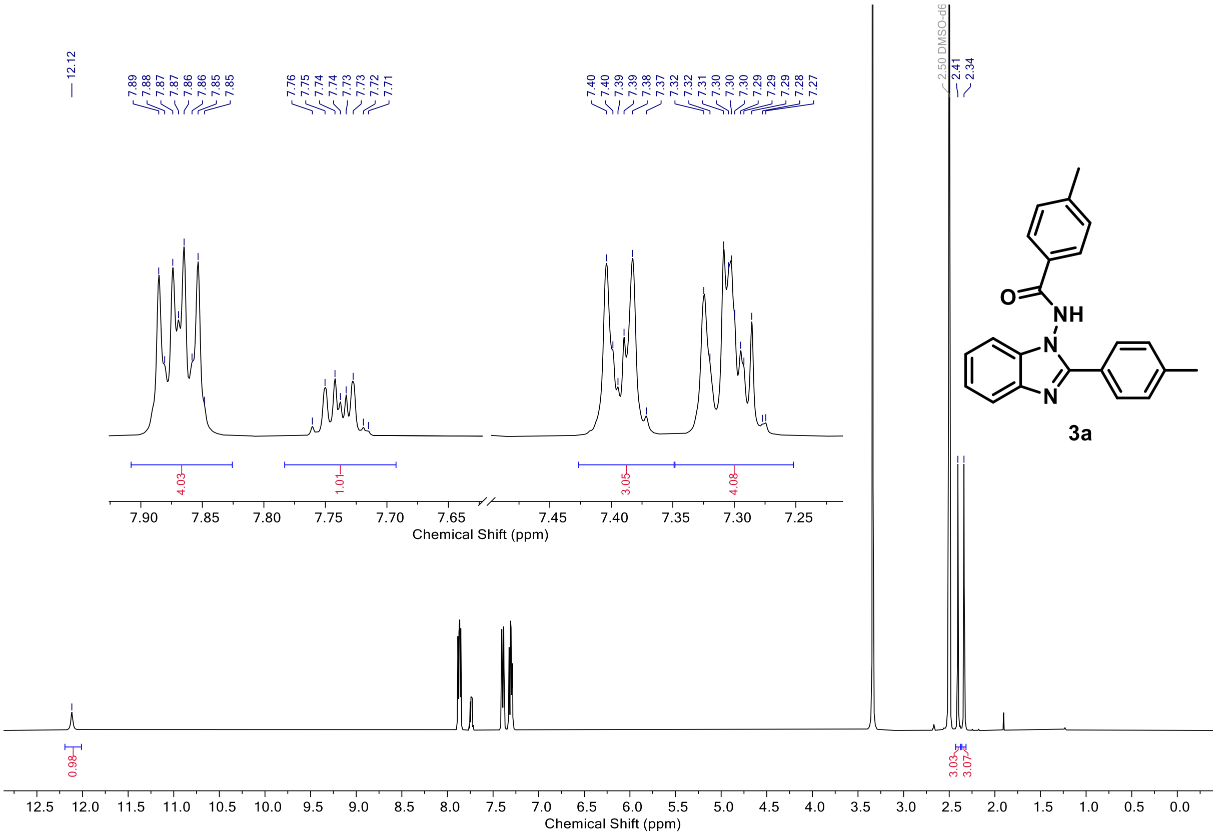

Figure S166: ^{1}H NMR (400 MHz, DMSO-d_6): 1*H*-4-Methyl-*N*-(2-(*p*-tolyl)-benzo[*d*]imidazol-1-yl)benzamide (**3a**).

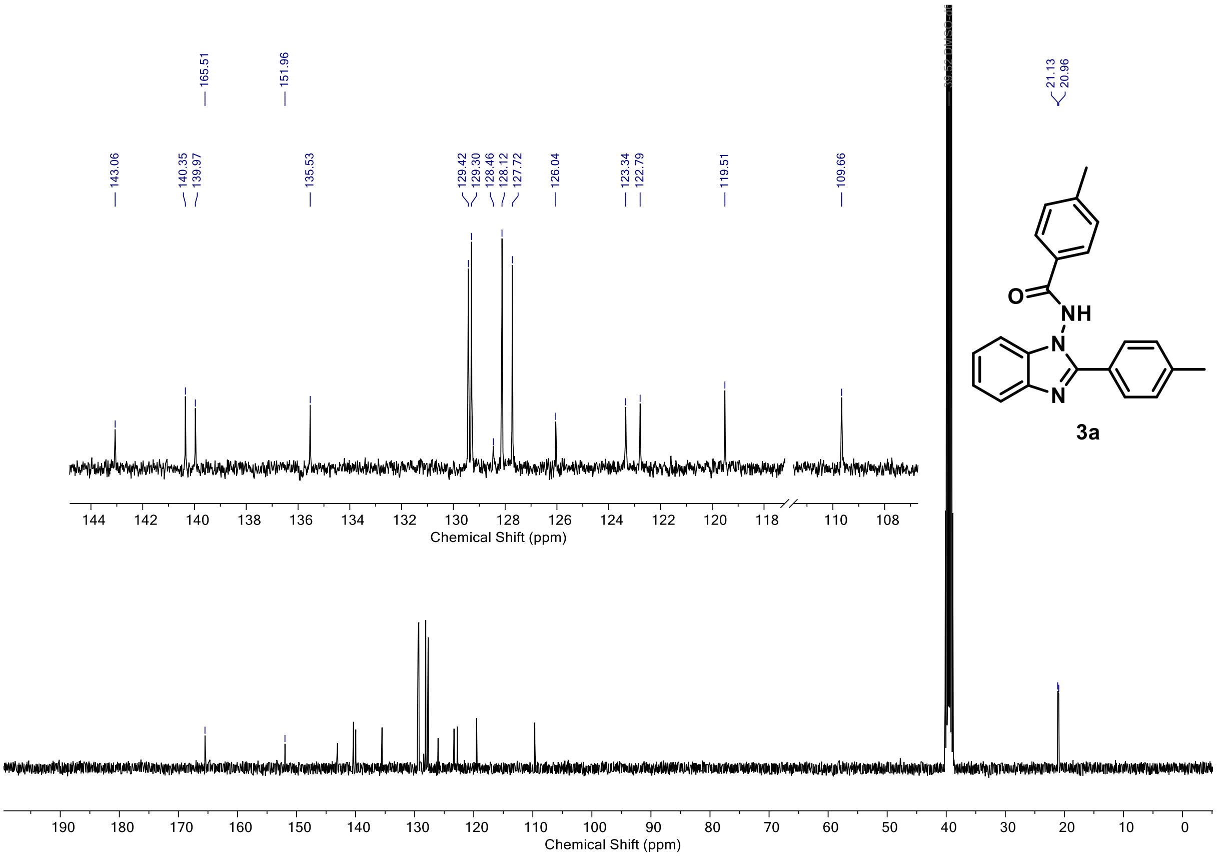

Figure S167: ^{13}C{^{1}H} NMR (101 MHz, DMSO-d_6): 1*H*-4-Methyl-*N*-(2-(*p*-tolyl)-benzo[*d*]imidazol-1-yl)benzamide (**3a**).

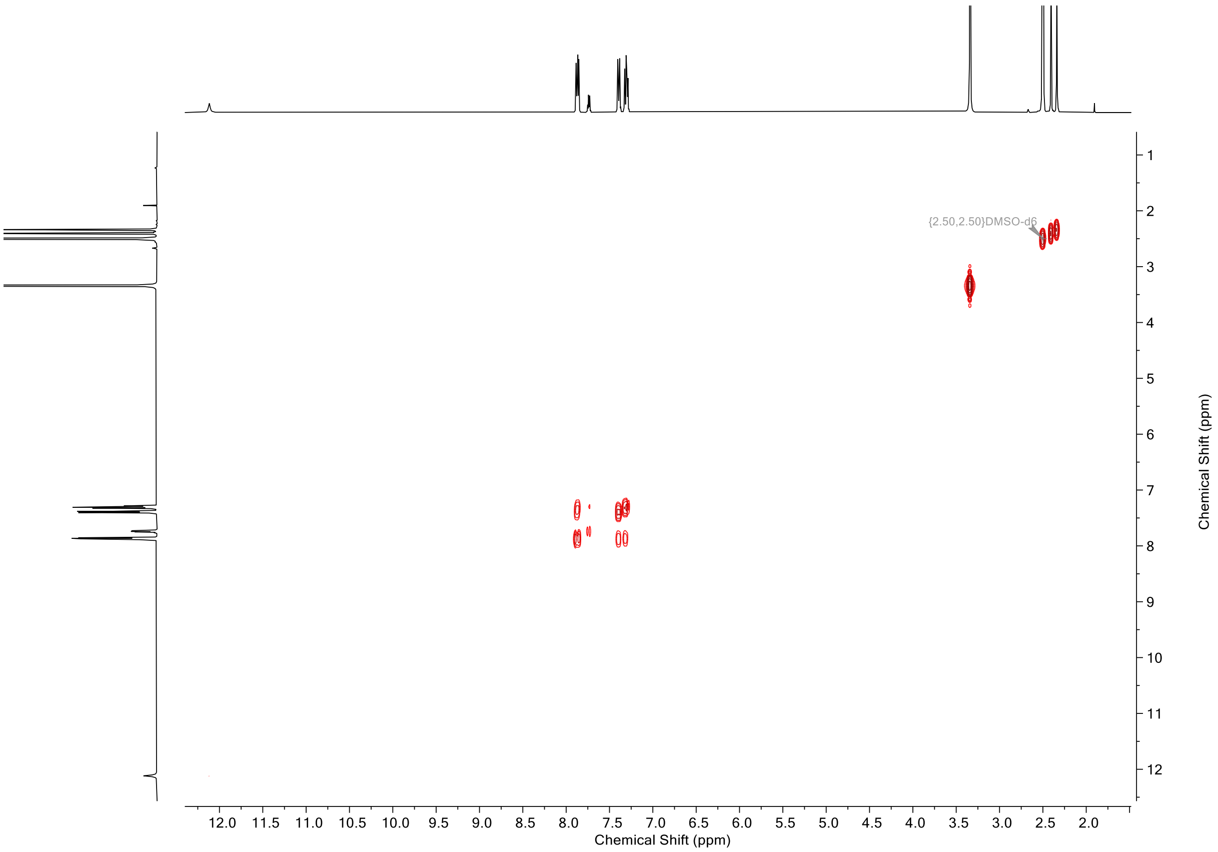

Figure S168: COSY (DMSO-d_6): 1*H*-4-Methyl-*N*-(2-(*p*-tolyl)-benzo[*d*]imidazol-1-yl)benzamide (**3a**).

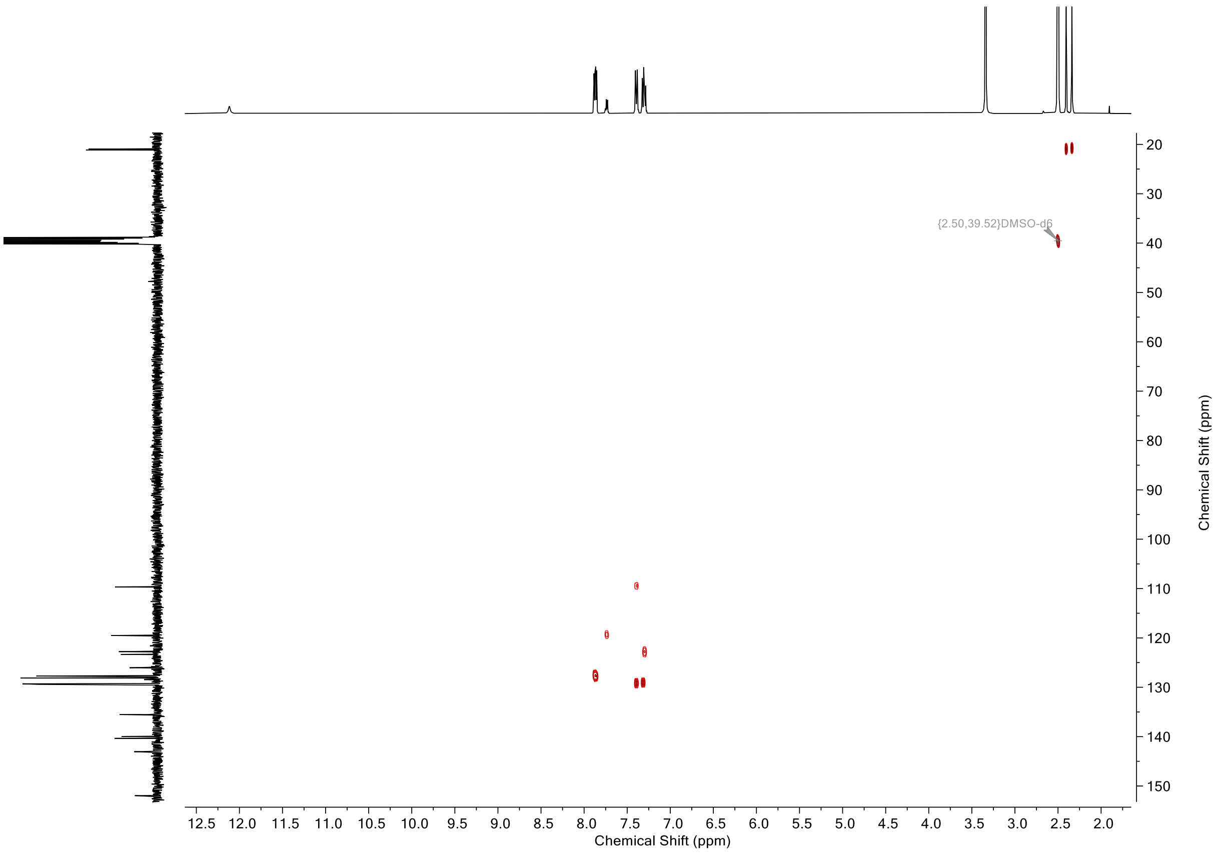

Figure S169: HSQC (DMSO-d_6): 1*H*-4-Methyl-*N*-(2-(*p*-tolyl)-benzo[*d*]imidazol-1-yl)benzamide (**3a**).

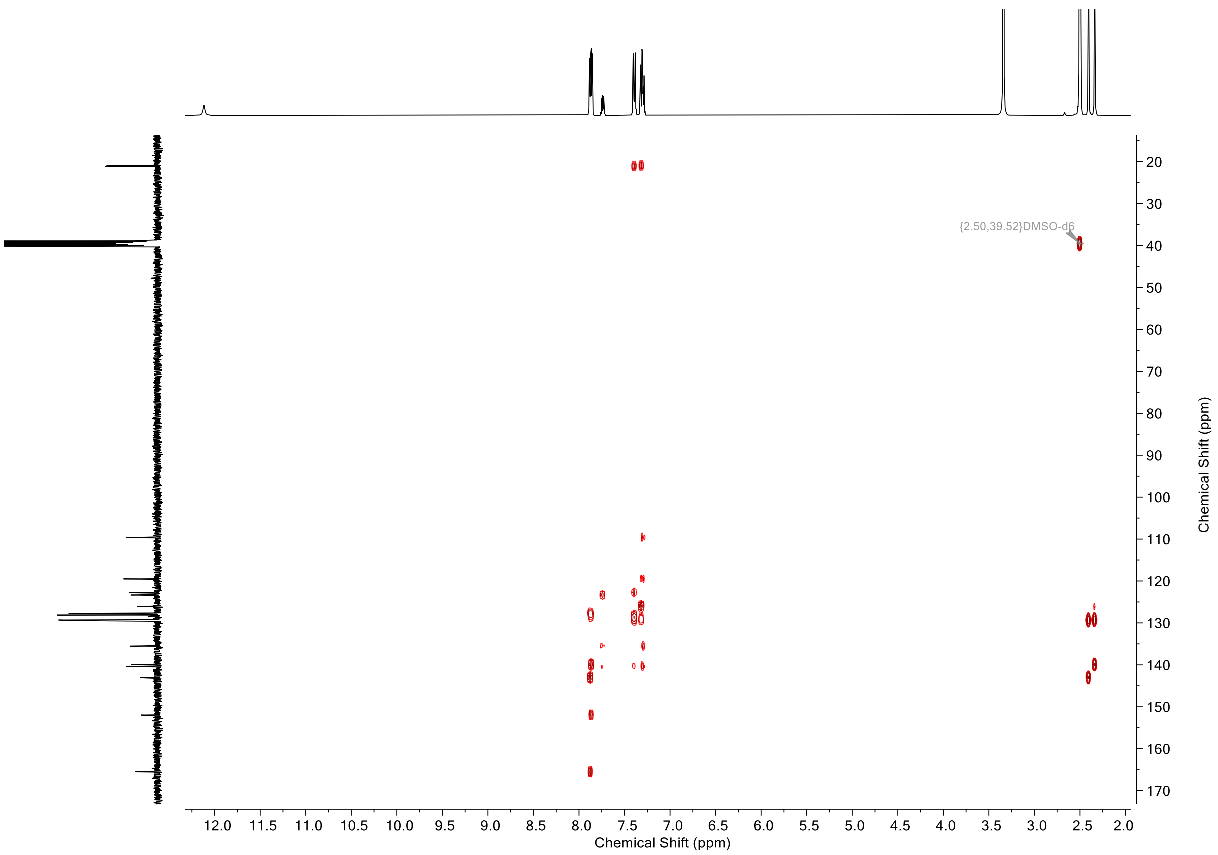

Figure S170: HMBC (DMSO-d_6): 1*H*-4-Methyl-*N*-(2-(*p*-tolyl)-benzo[*d*]imidazol-1-yl)benzamide (**3a**).

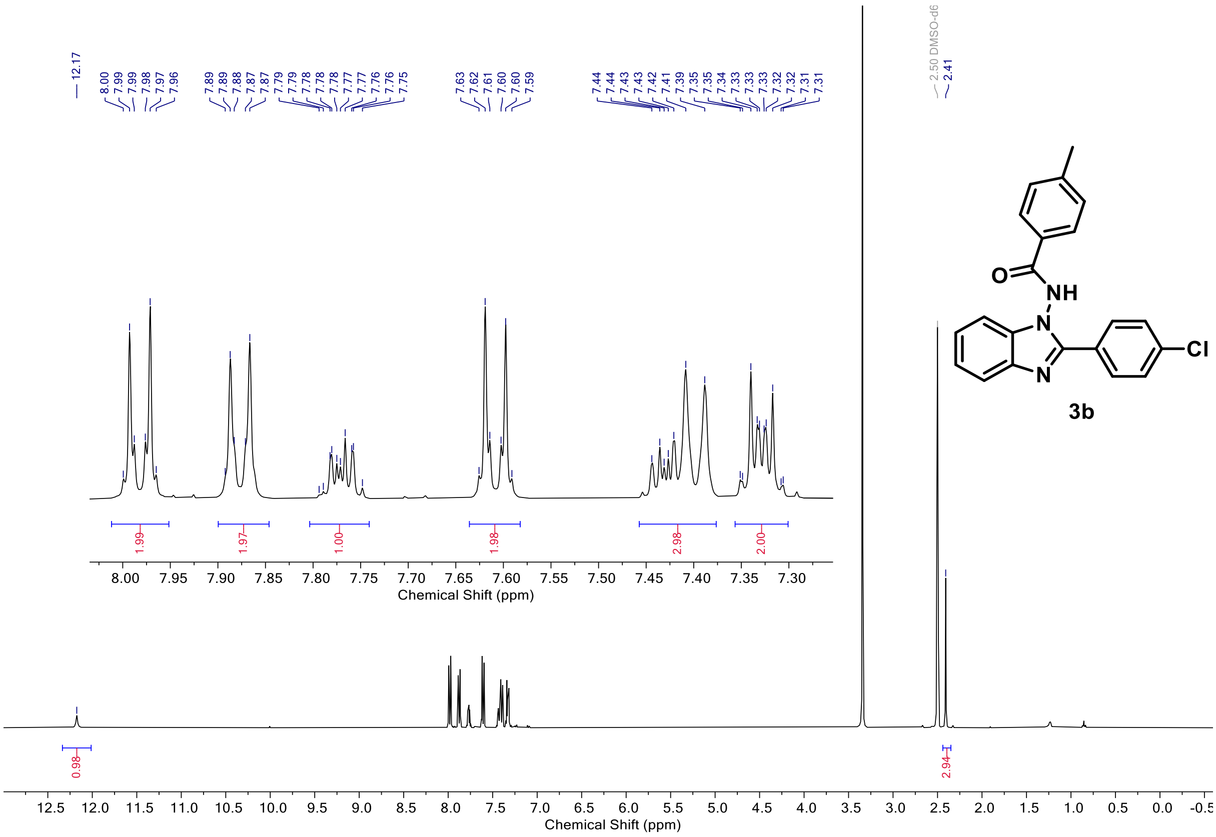

Figure S171: ^{1}H NMR (400 MHz, DMSO-d_6): 1*H*-*N*-(2-(4-Chlorophenyl)-benzo[*d*]imidazol-1-yl)-4-methylbenzamide (**3b**).

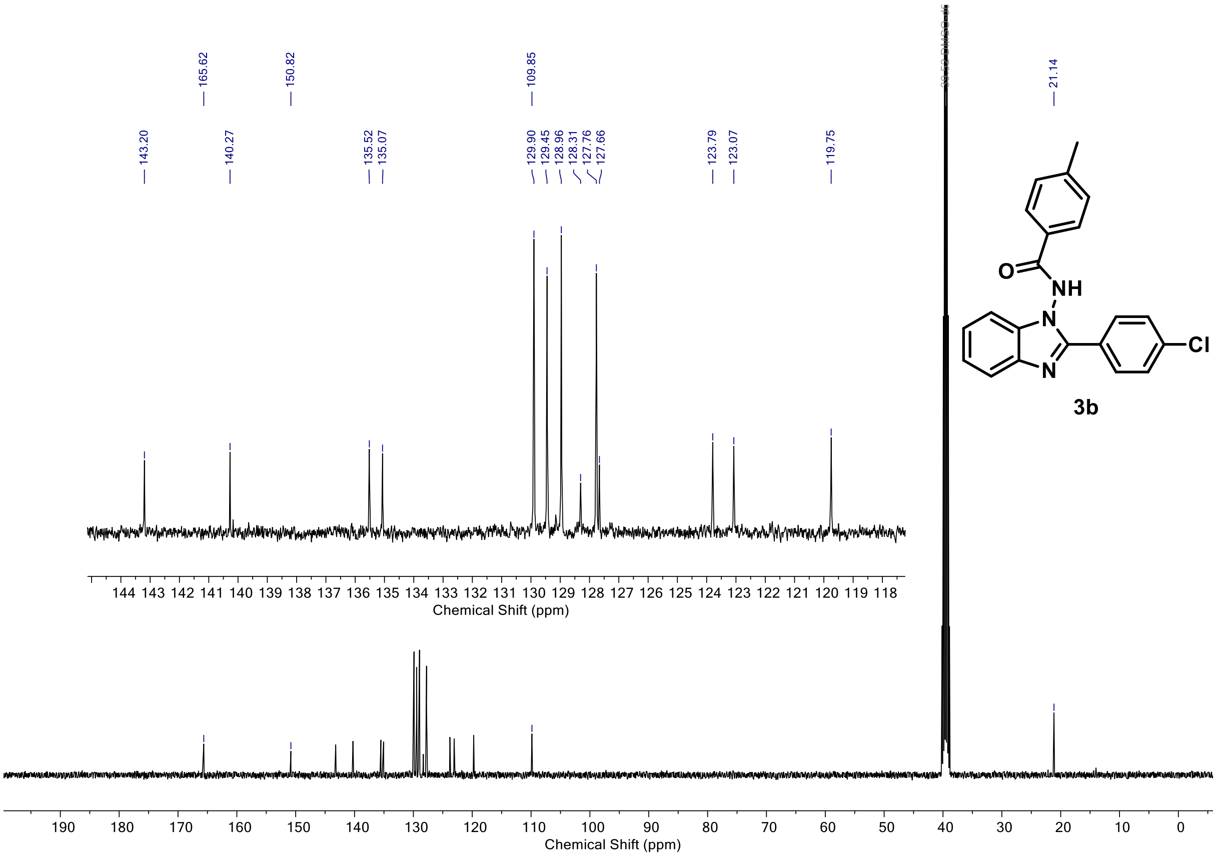

Figure S172: ^{13}C{^{1}H} NMR (101 MHz, DMSO-d_6): 1*H*-*N*-(2-(4-Chlorophenyl)-benzo[*d*]imidazol-1-yl)-4-methylbenz-amide (**3b**).

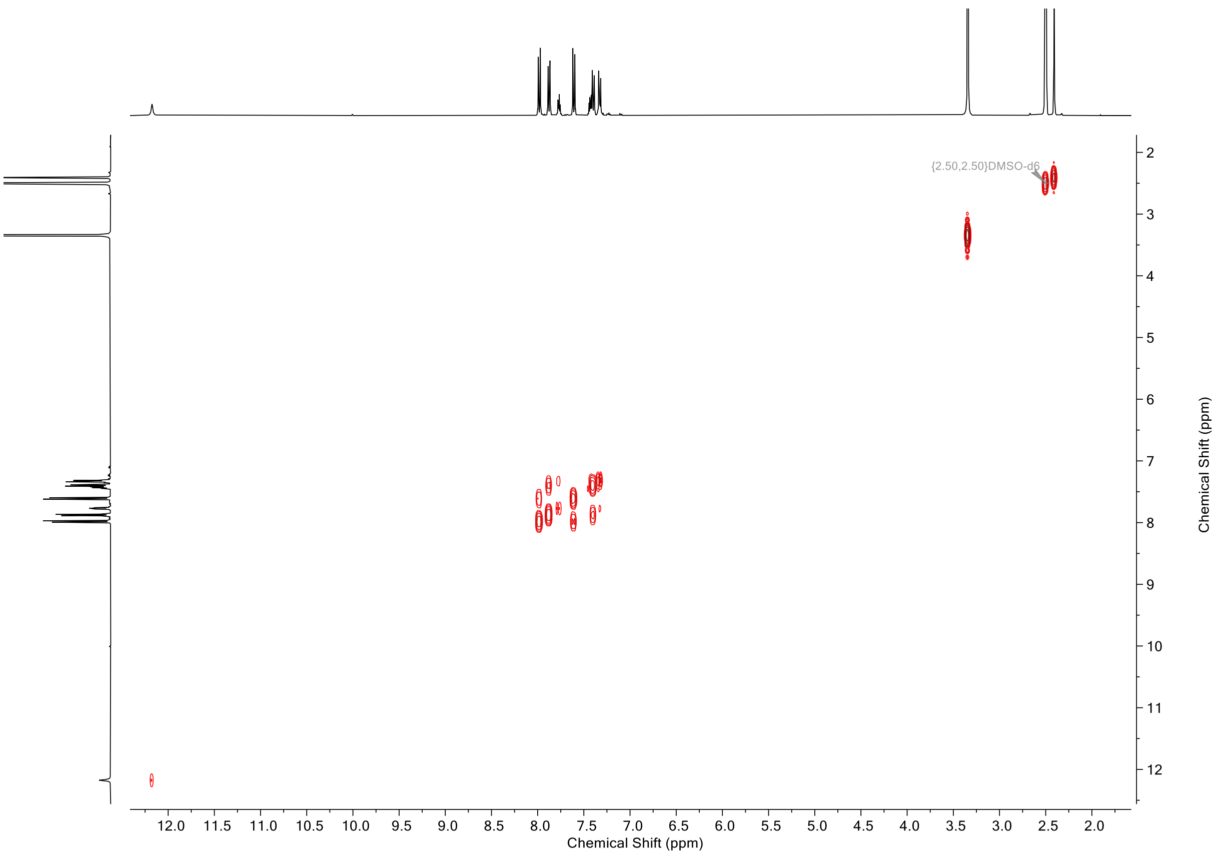

Figure S173: COSY (DMSO-d_6): 1*H*-*N*-(2-(4-Chlorophenyl)-benzo[*d*]imidazol-1-yl)-4-methylbenz-amide (**3b**).

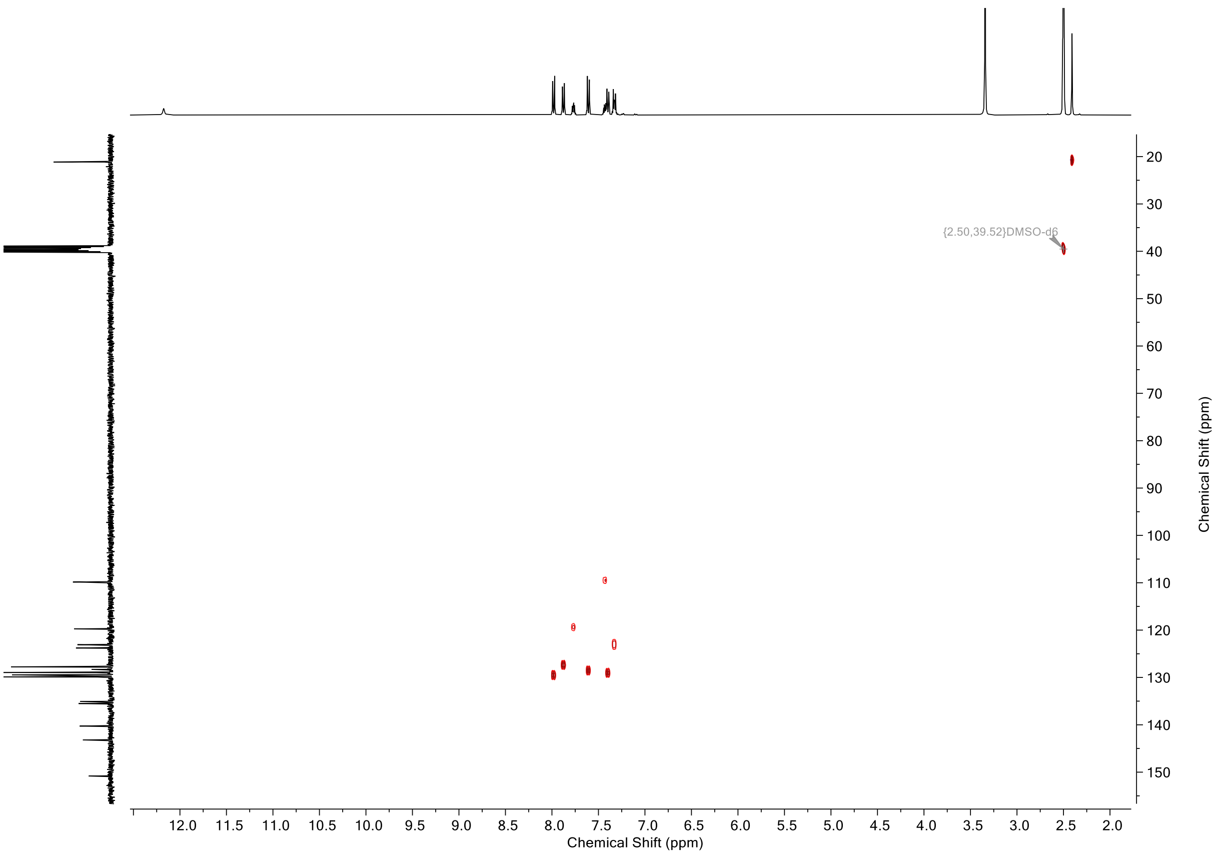

Figure S174: HSQC (DMSO-d_6): 1*H*-*N*-(2-(4-Chlorophenyl)-benzo[*d*]imidazol-1-yl)-4-methylbenz-amide (**3b**).

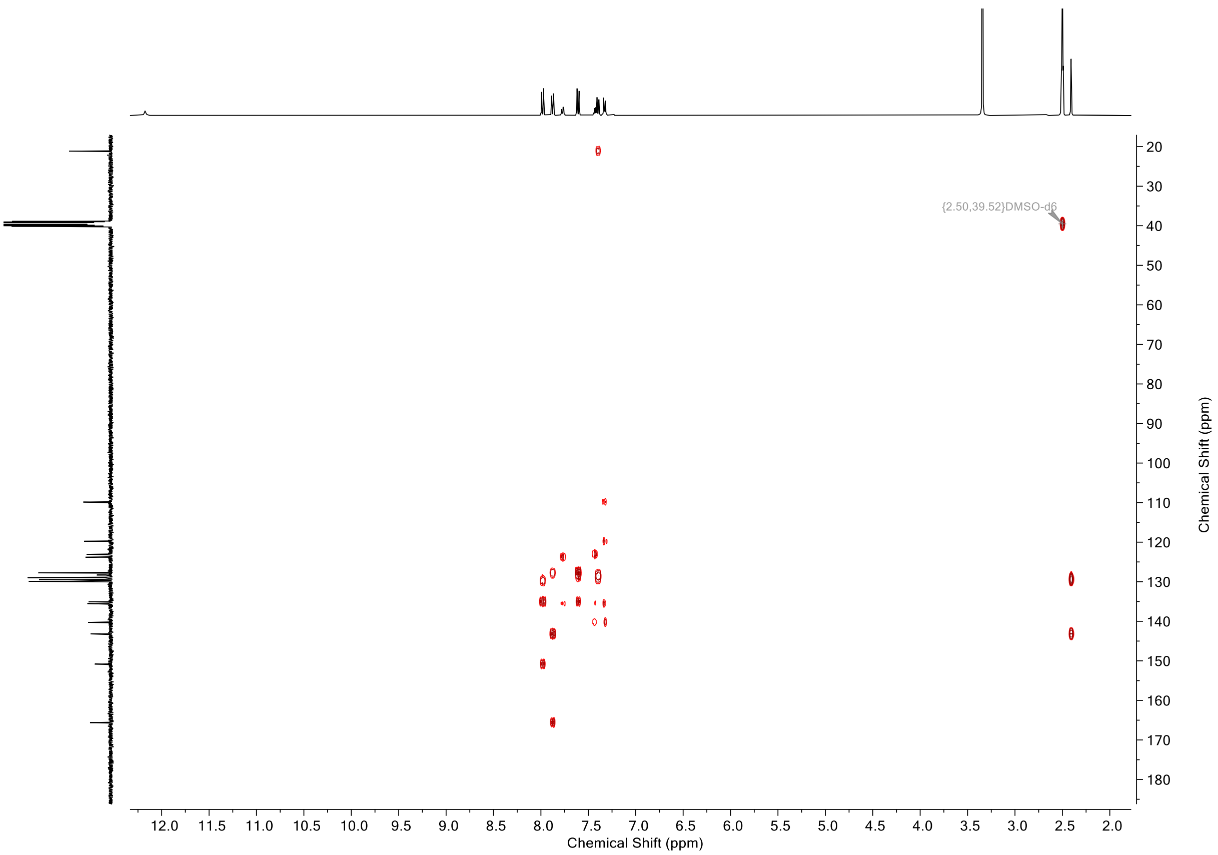

Figure S175: HMBC (DMSO-d_6): 1*H*-*N*-(2-(4-Chlorophenyl)-benzo[*d*]imidazol-1-yl)-4-methylbenz-amide (**3b**).

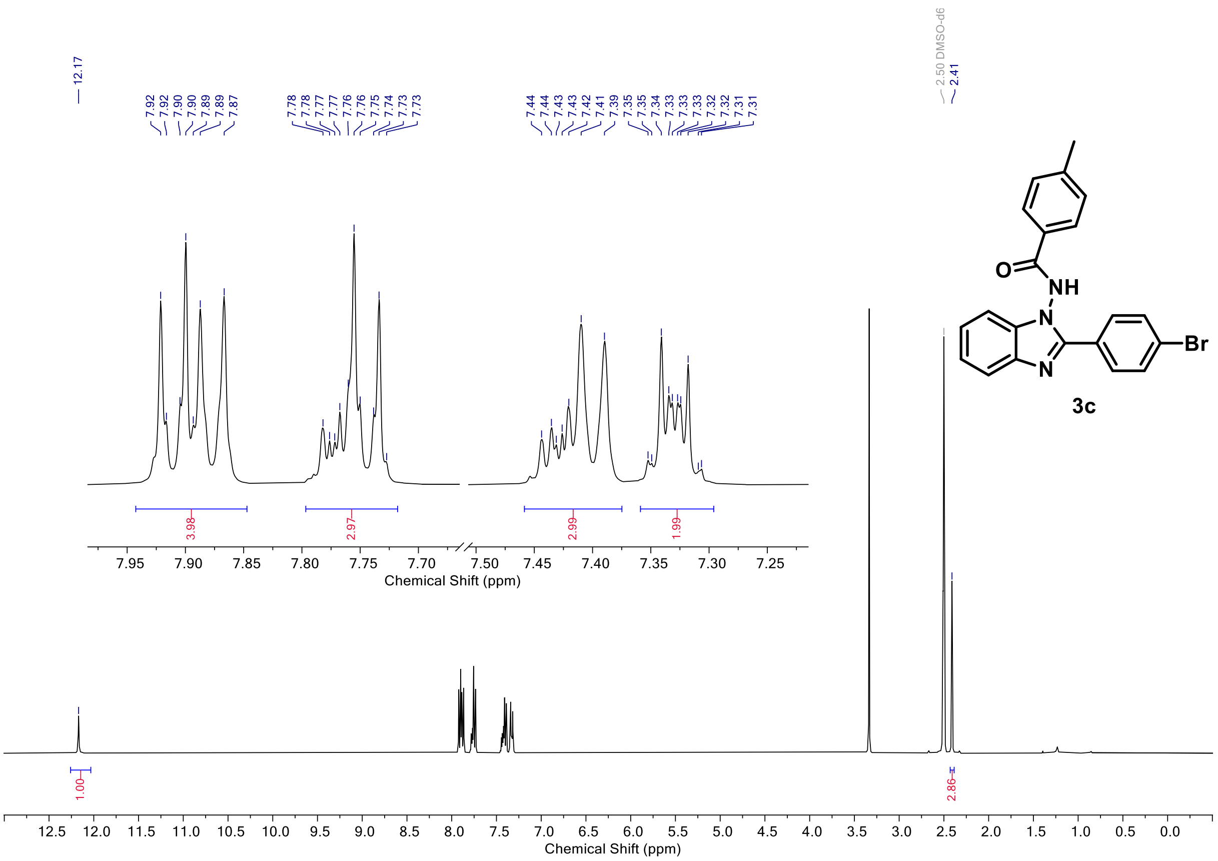

Figure S176: ^{1}H NMR (400 MHz, DMSO-d_6): 1*H*-*N*-(2-(4-Bromophenyl)-benzo[*d*]imidazol-1-yl)-4-methylbenzamide (**3c**).

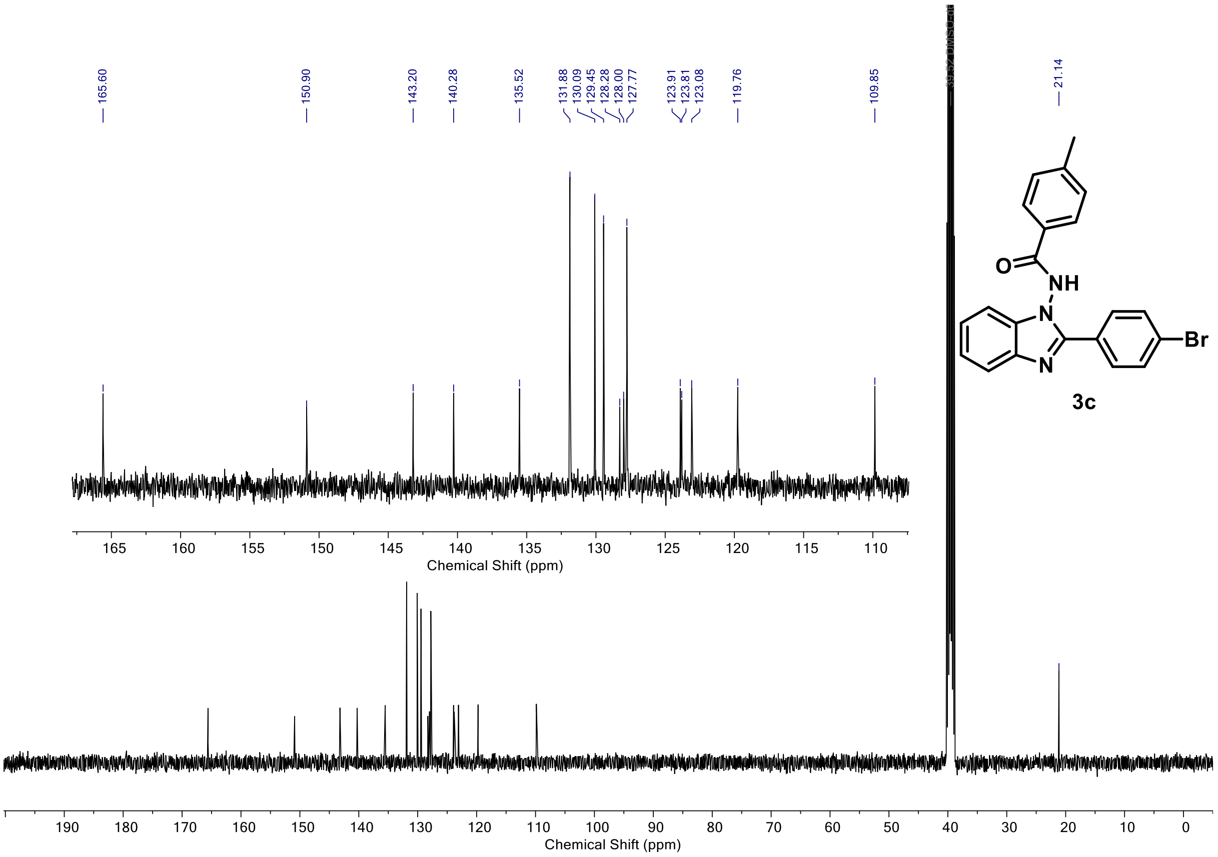

Figure S177: ^{13}C{^{1}H} NMR (101 MHz, DMSO-d_6): 1*H*-*N*-(2-(4-Bromophenyl)-benzo[*d*]imidazol-1-yl)-4-methylbenzamide (**3c**).

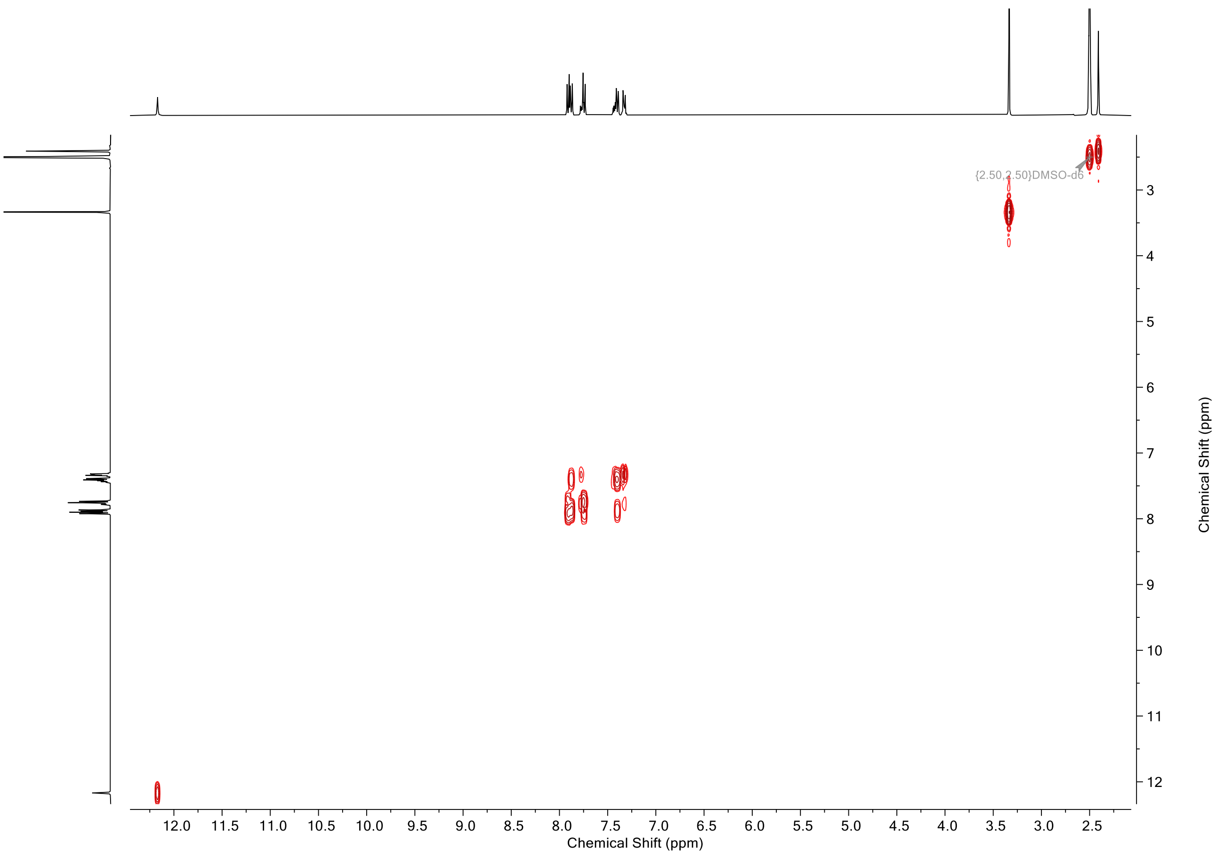

Figure S178: COSY (DMSO-d_6): 1*H*-*N*-(2-(4-Bromophenyl)-benzo[*d*]imidazol-1-yl)-4-methylbenzamide (**3c**).

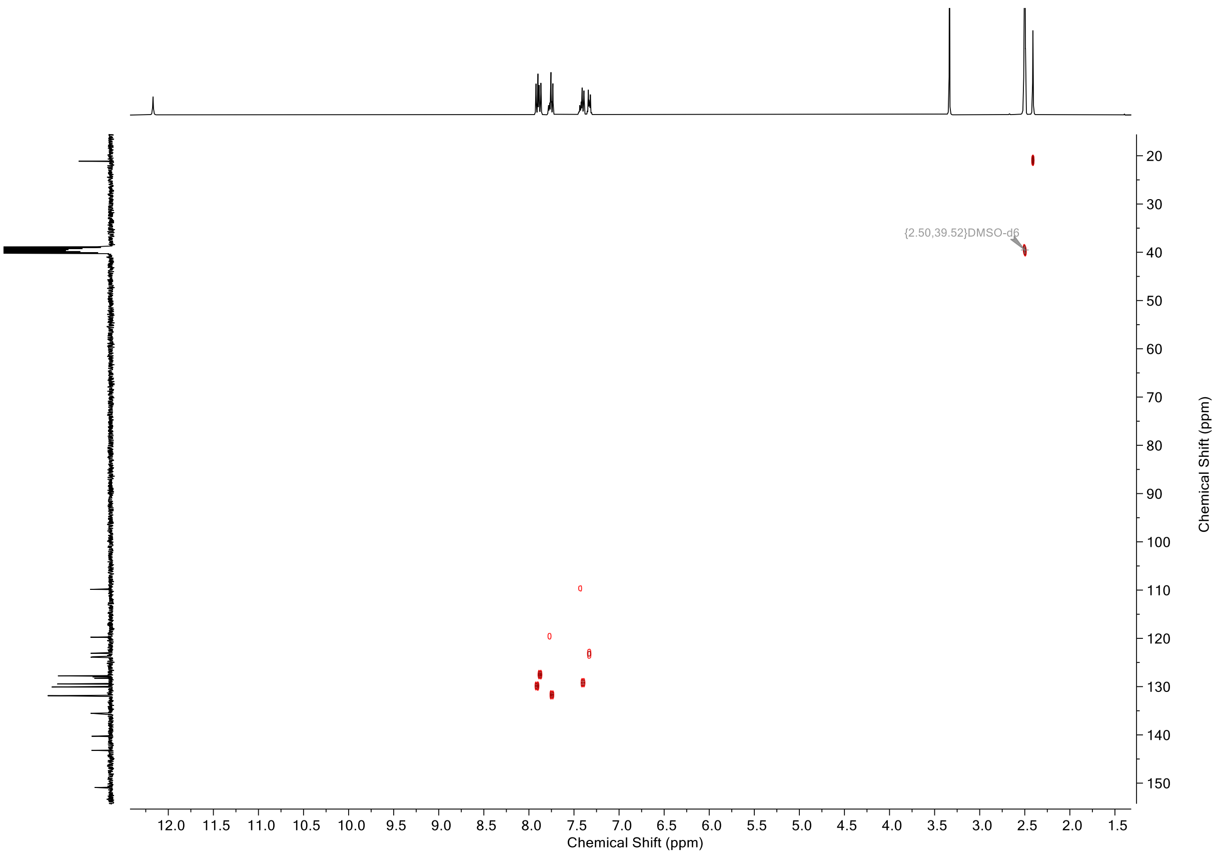

Figure S179: HSQC (DMSO-d_6): 1*H*-*N*-(2-(4-Bromophenyl)-benzo[*d*]imidazol-1-yl)-4-methylbenzamide (**3c**).

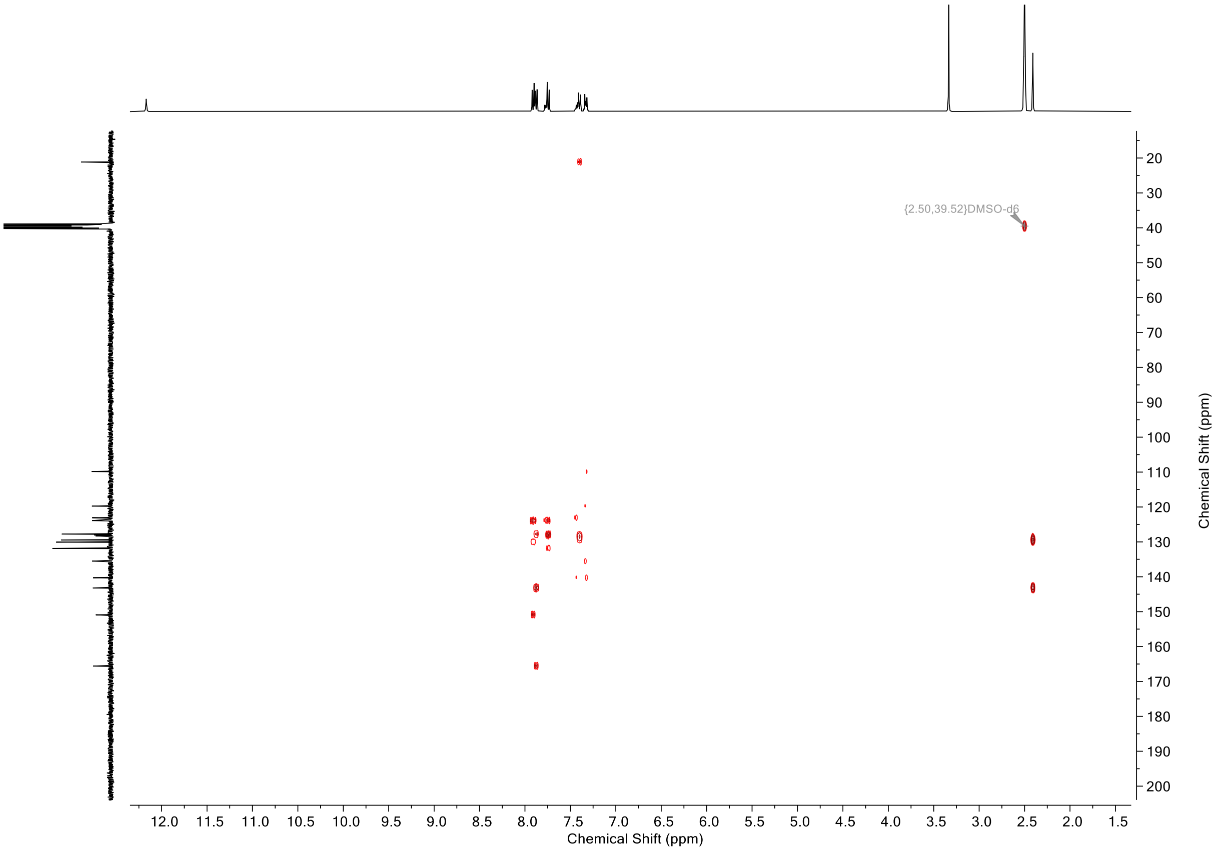

Figure S180: HMBC (DMSO-d_6): 1*H*-*N*-(2-(4-Bromophenyl)-benzo[*d*]imidazol-1-yl)-4-methylbenzamide (**3c**).

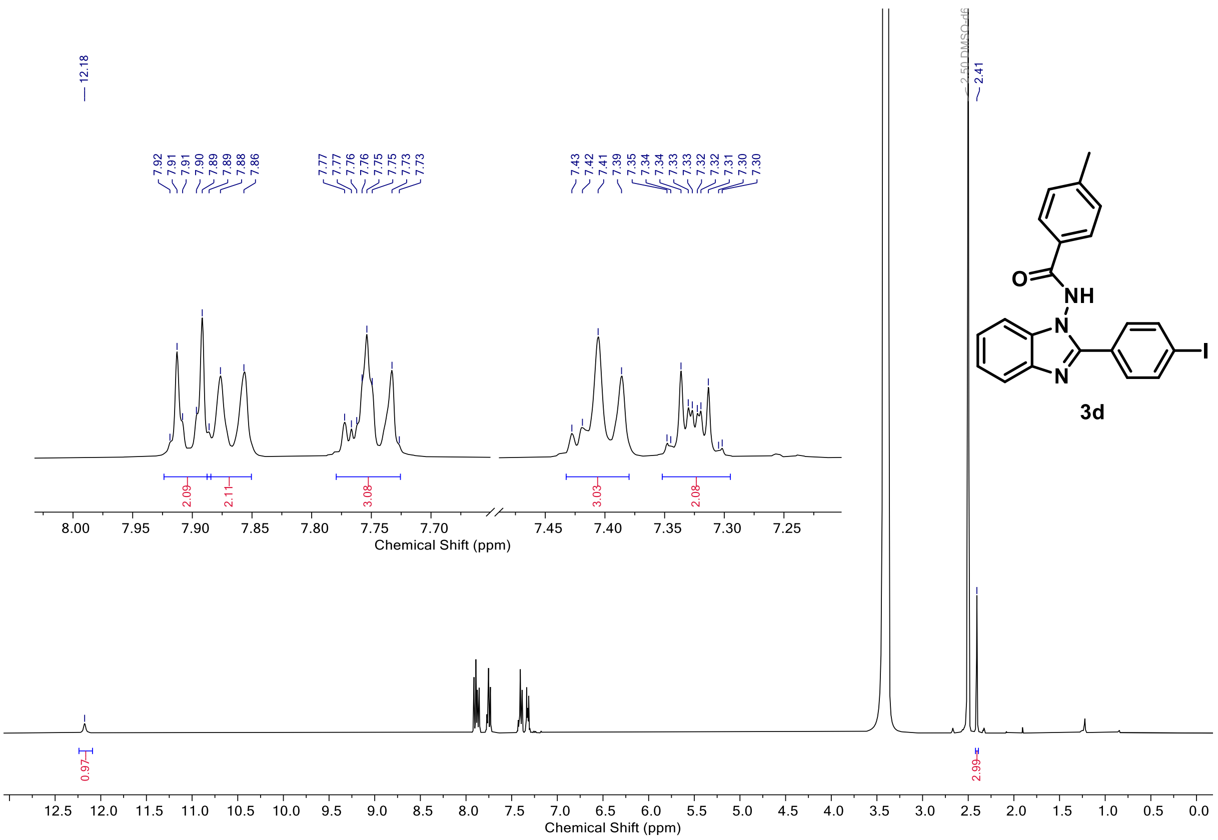

Figure S181: ^{1}H NMR (400 MHz, DMSO-d_6): 1*H*-*N*-(2-(4-Iodophenyl)-benzo[*d*]imidazol-1-yl)-4-methylbenzamide (**3d**).

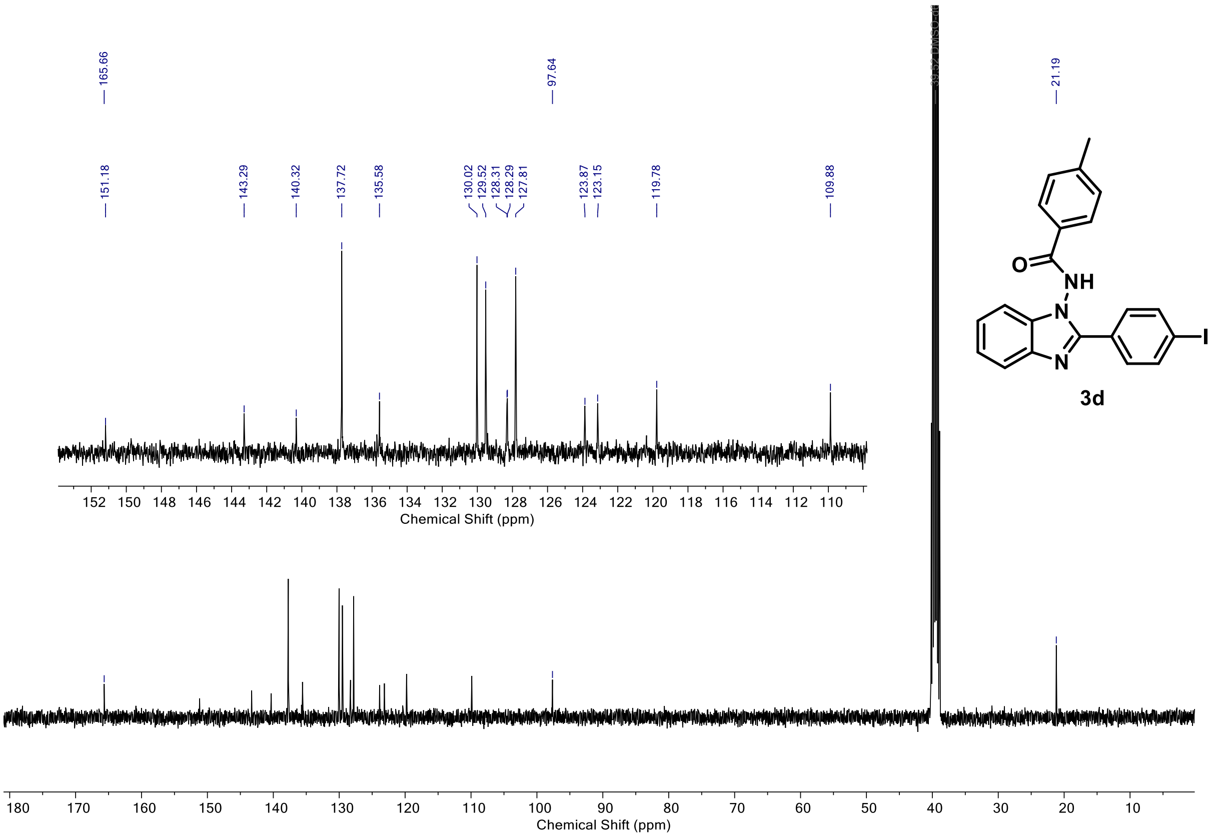

Figure S182: ^{13}C{^{1}H} NMR (101 MHz, DMSO-d_6): 1*H*-*N*-(2-(4-Iodophenyl)-benzo[*d*]imidazol-1-yl)-4-methylbenzamide (**3d**).

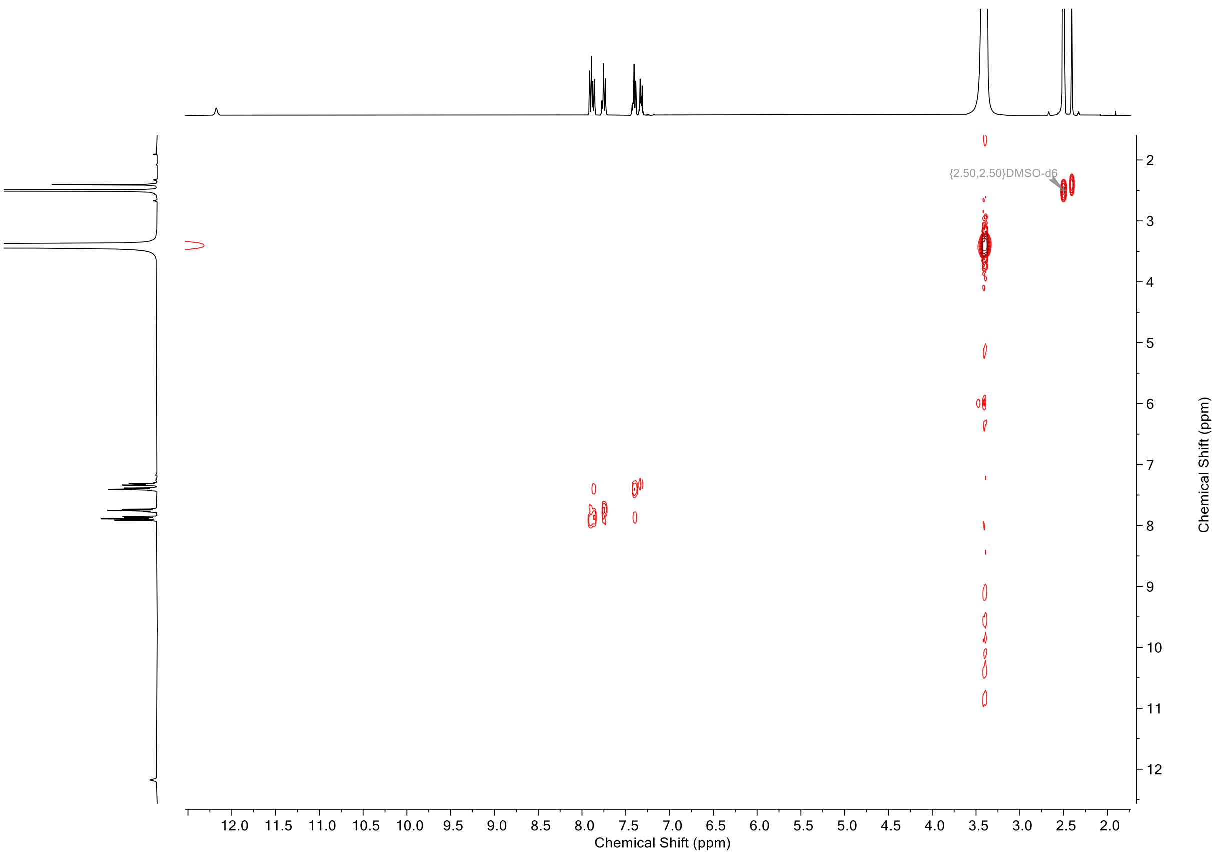

Figure S183: COSY (DMSO-d_6): 1*H*-*N*-(2-(4-Iodophenyl)-benzo[*d*]imidazol-1-yl)-4-methylbenzamide (**3d**).

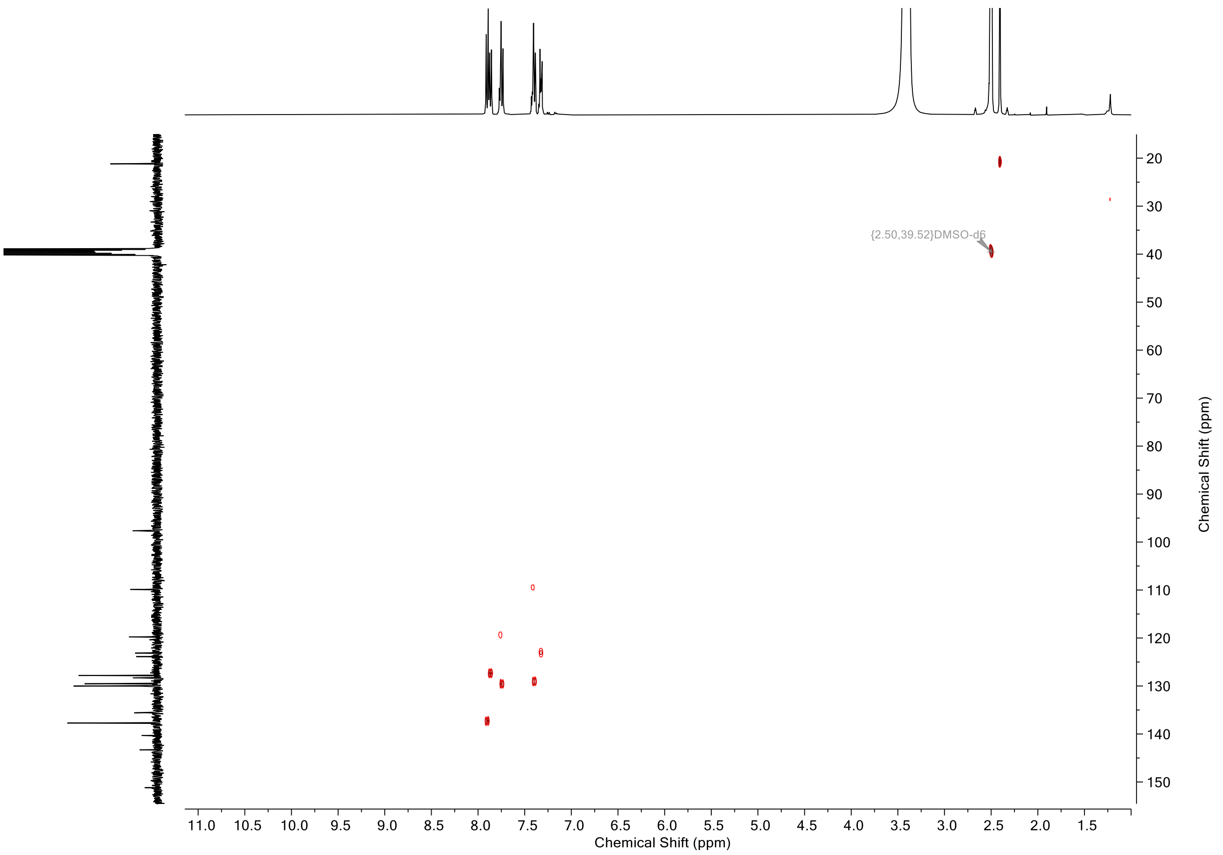

Figure S184: HSQC (DMSO-d_6): 1*H*-*N*-(2-(4-Iodophenyl)-benzo[*d*]imidazol-1-yl)-4-methylbenzamide (**3d**).

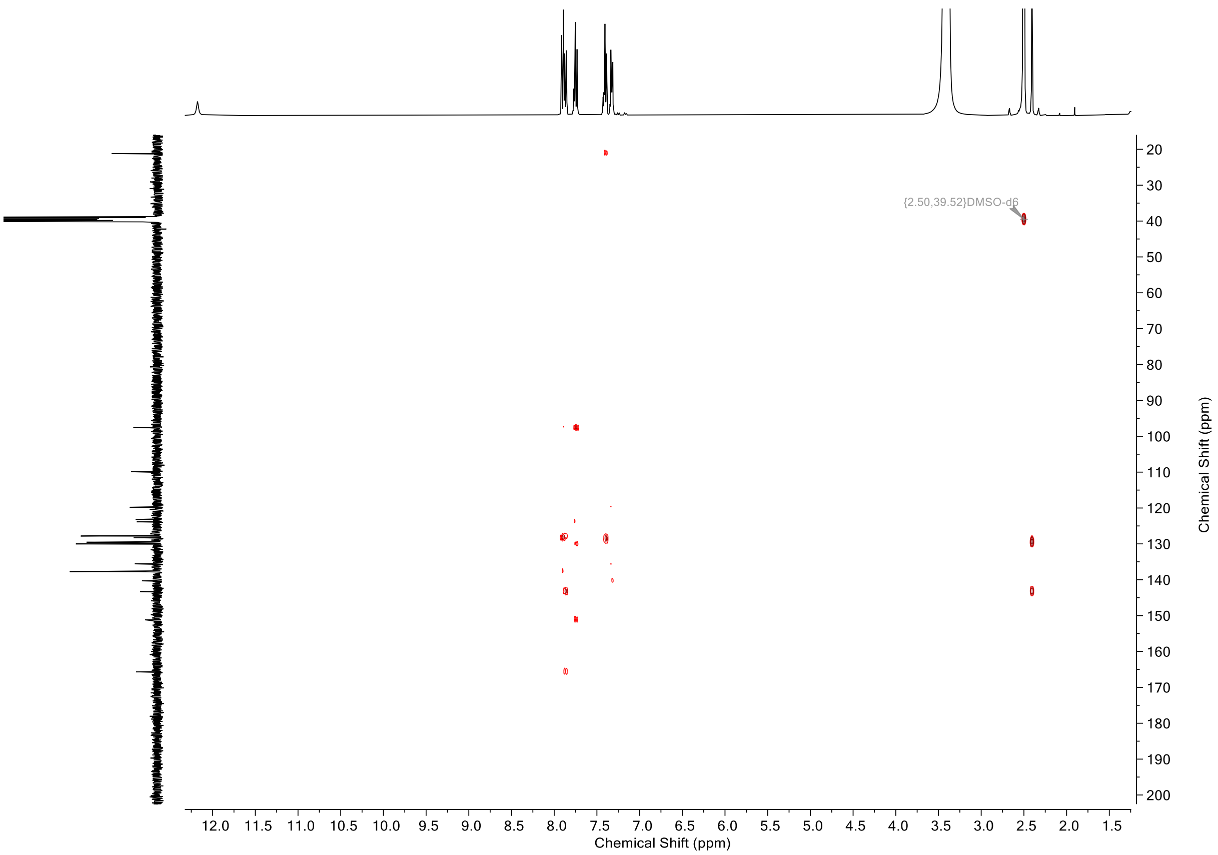

Figure S185: HMBC (DMSO-d_6): 1*H*-*N*-(2-(4-Iodophenyl)-benzo[*d*]imidazol-1-yl)-4-methylbenzamide (**3d**).

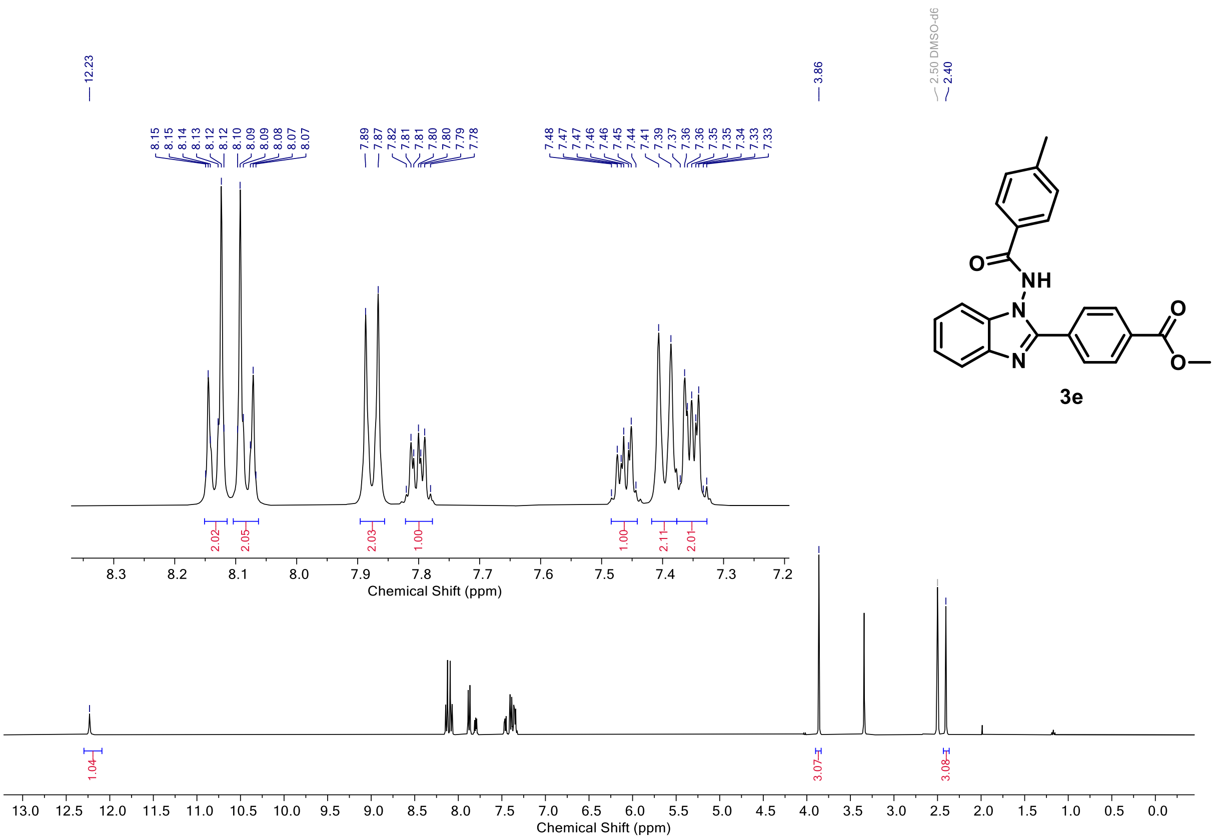

Figure S186: ^{1}H NMR (400 MHz, DMSO-d_6): Methyl 1*H*-4-(1-(4-methylbenzamido)-benzo[*d*]imidazol-2-yl)benzoate (**3e**).

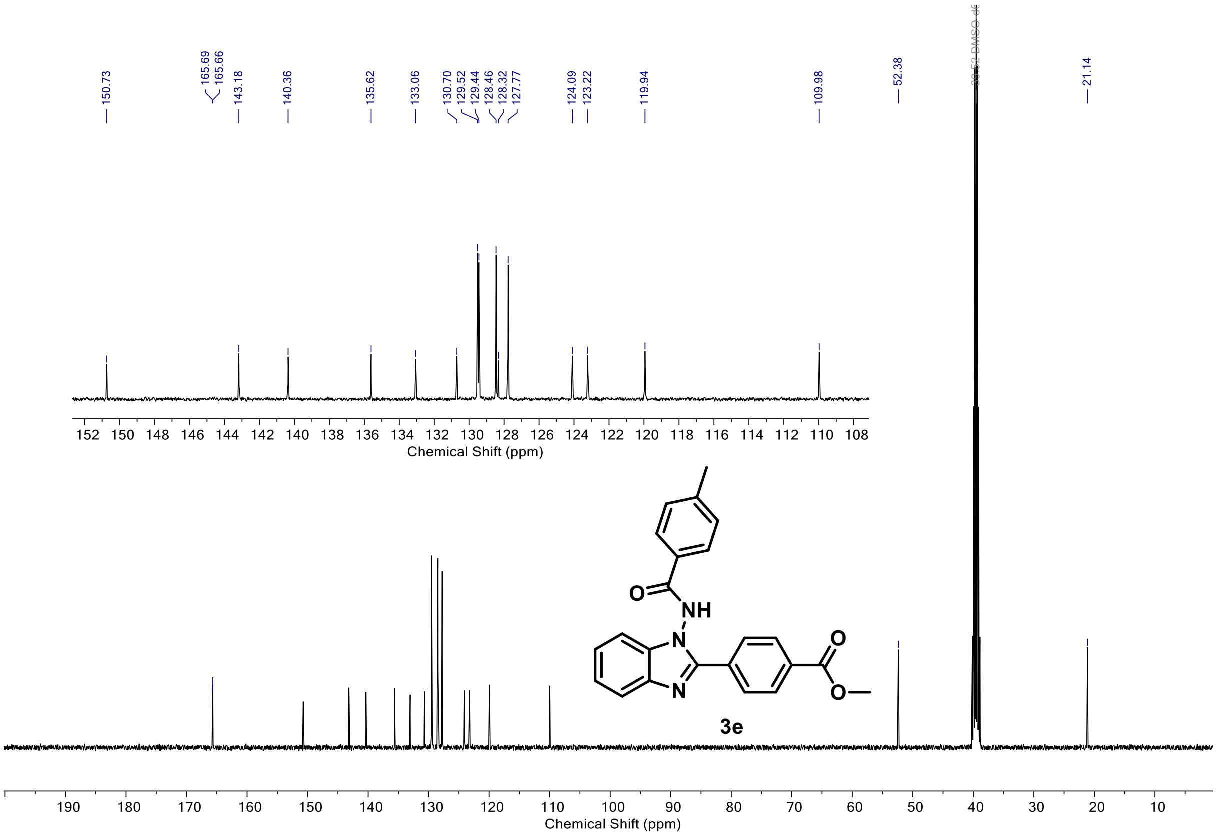

Figure S187: $^{13}C\{^{1}H\}$ NMR (101 MHz, DMSO-d_6): Methyl 1*H*-4-(1-(4-methylbenzamido)-benzo[*d*]imidazol-2-yl)benzoate (**3e**).

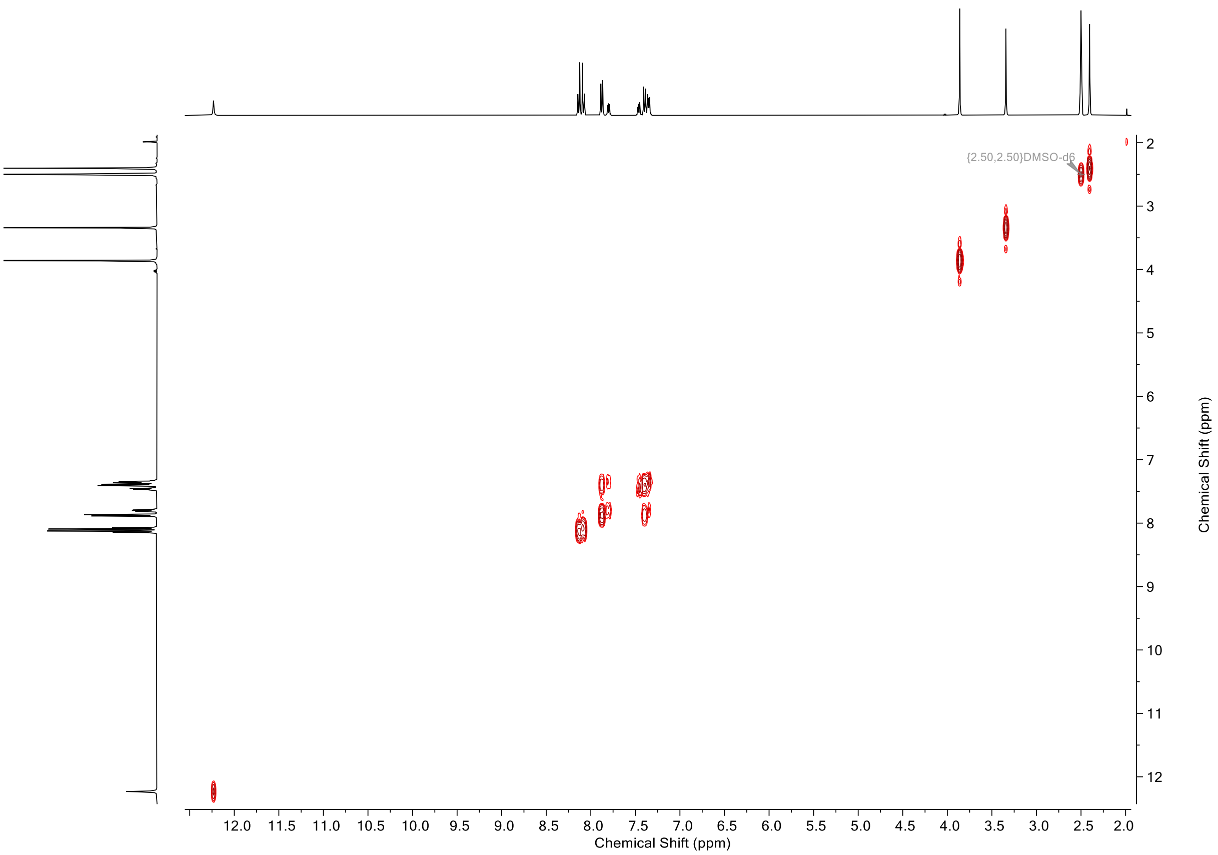

Figure S188: COSY (DMSO-d_6): Methyl 1*H*-4-(1-(4-methylbenzamido)-benzo[*d*]imidazol-2-yl)benzoate (**3e**).

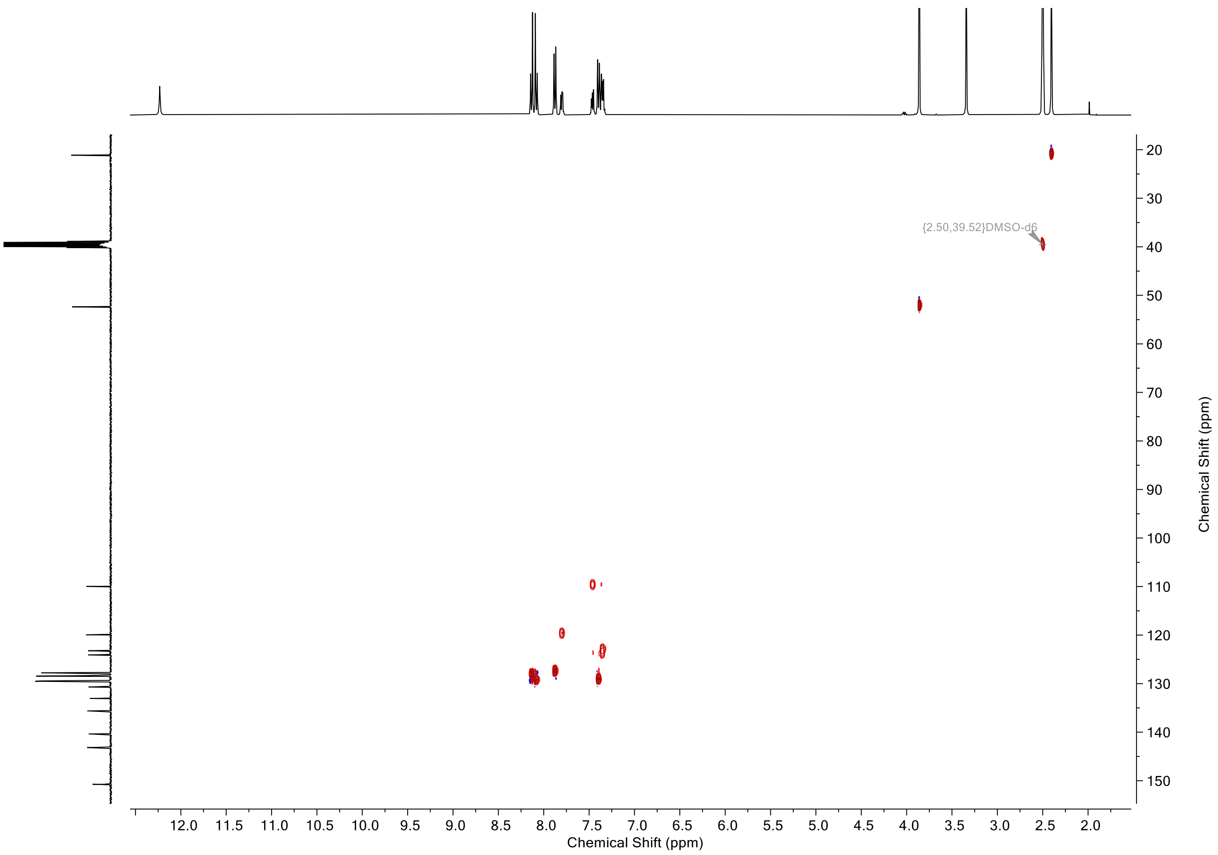

Figure S189: HSQC (DMSO-d_6): Methyl 1*H*-4-(1-(4-methylbenzamido)-benzo[*d*]imidazol-2-yl)benzoate (**3e**).

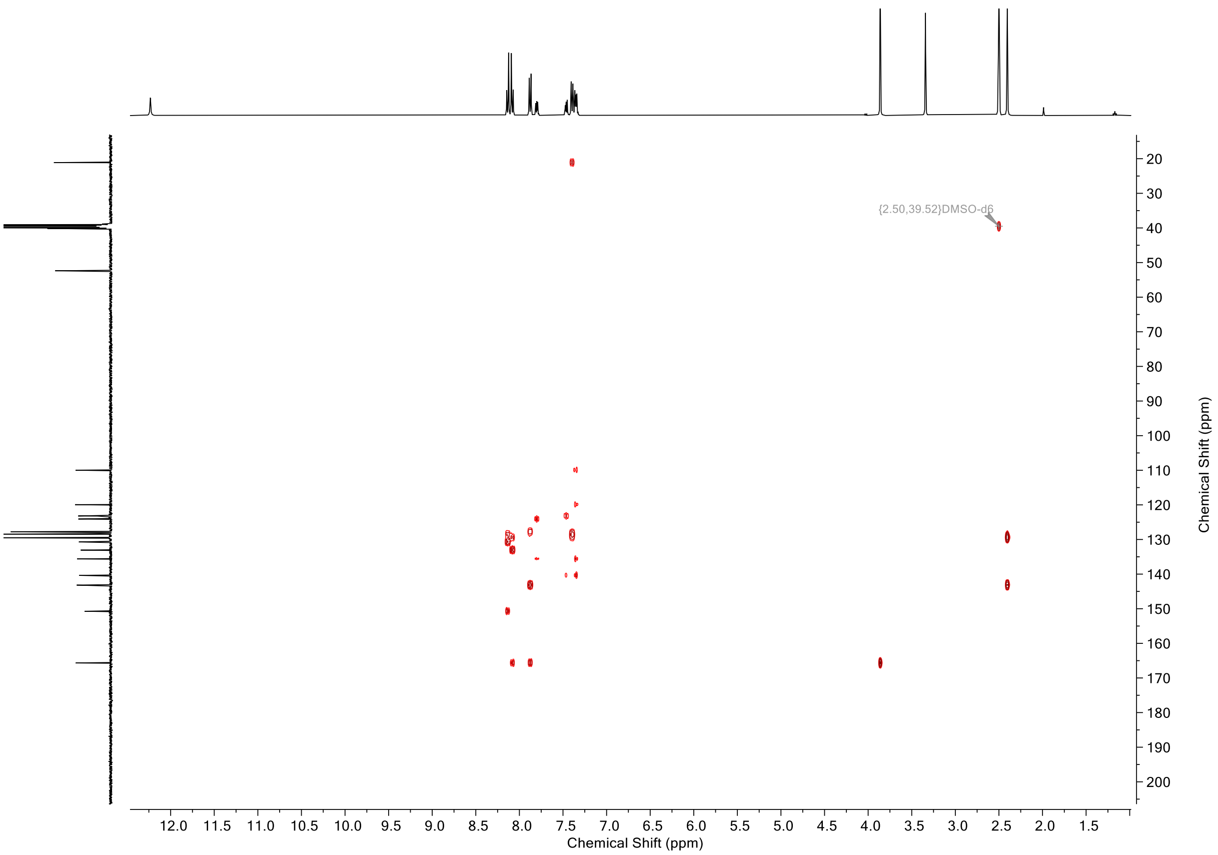

Figure S190: HMBC (DMSO-d_6): Methyl 1*H*-4-(1-(4-methylbenzamido)-benzo[*d*]imidazol-2-yl)benzoate (**3e**).

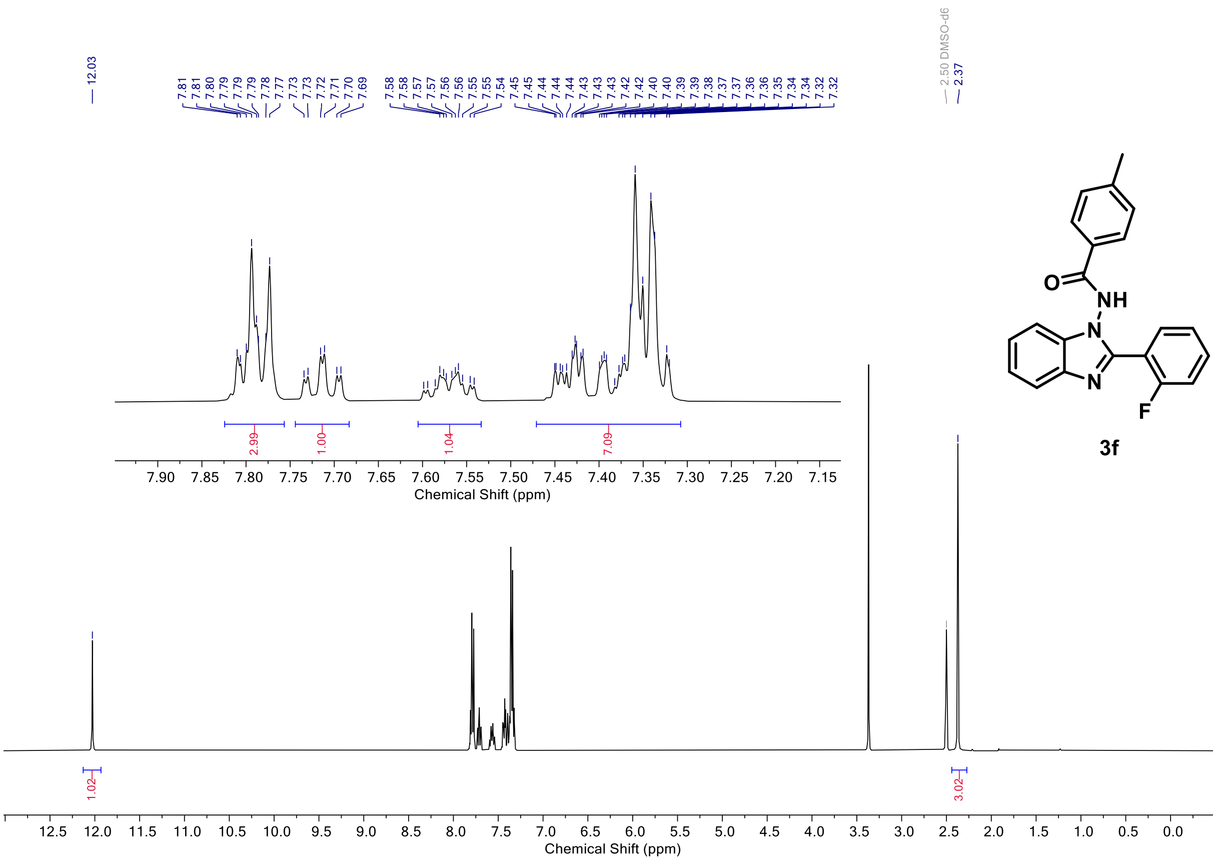

Figure S191: ^{1}H NMR (400 MHz, DMSO-d_6): 1*H*-*N*-(2-(2-Fluorophenyl)-benzo[*d*]imidazol-1-yl)-4-methylbenzamide (**3f**).

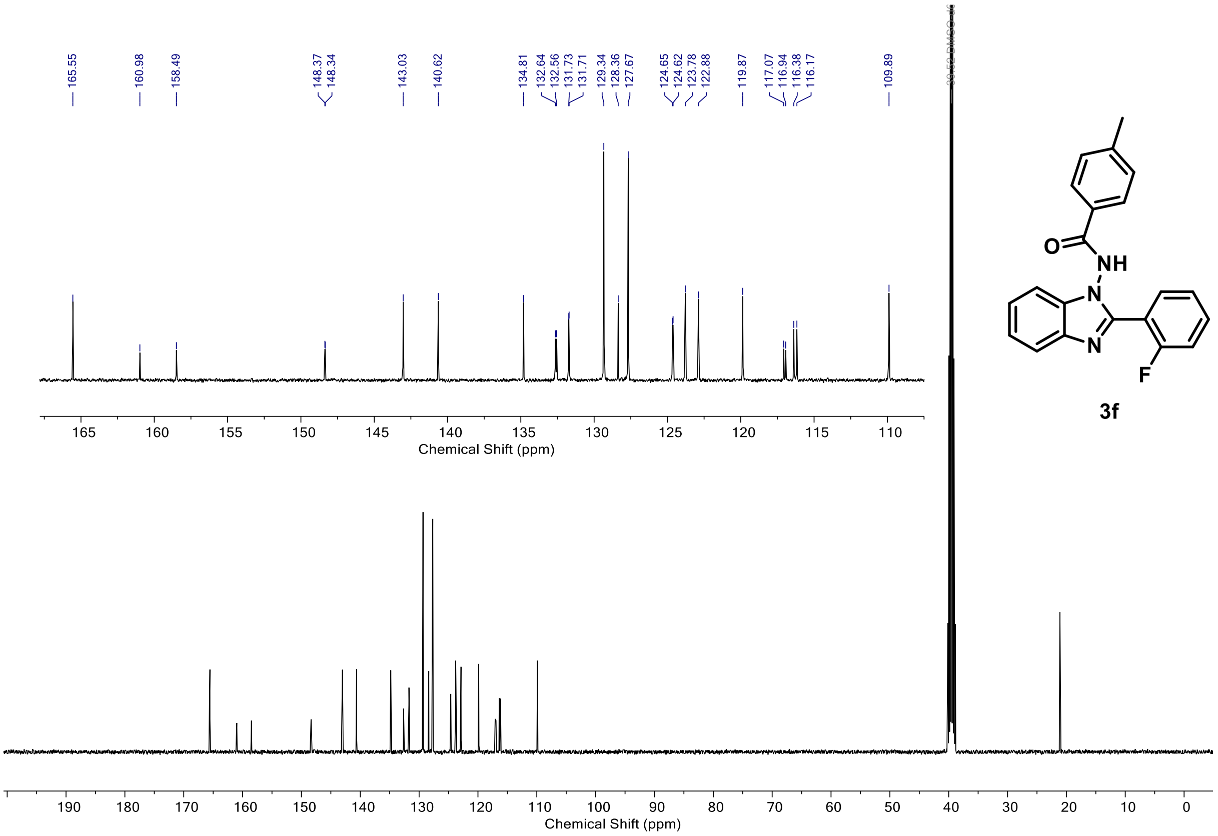

Figure S191: ^{13}C{^{1}H} NMR (101 MHz, DMSO-d_6): 1*H*-*N*-(2-(2-Fluorophenyl)-benzo[*d*]imidazol-1-yl)-4-methylbenzamide (**3f**).

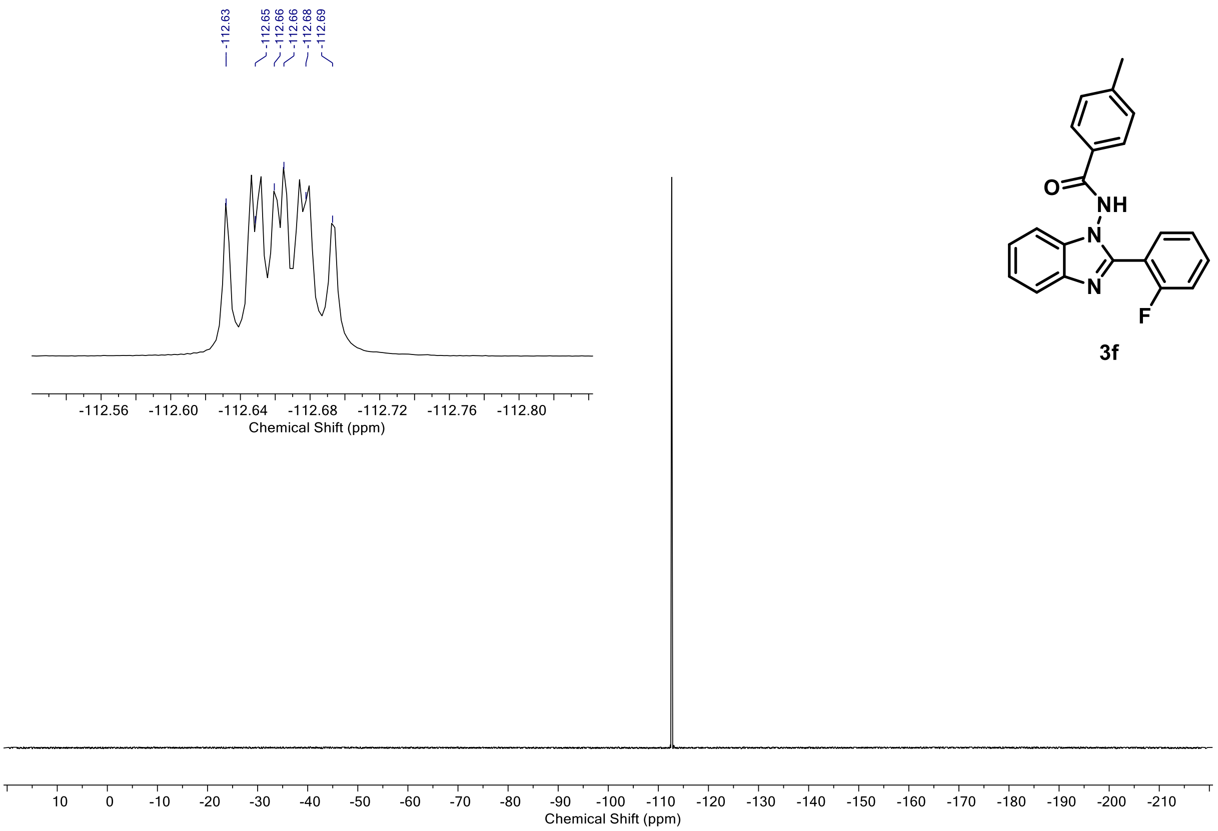

Figure S192: ^{19}F NMR (376 MHz, DMSO-d_6): 1*H*-*N*-(2-(2-Fluorophenyl)-benzo[*d*]imidazol-1-yl)-4-methylbenzamide (**3f**).

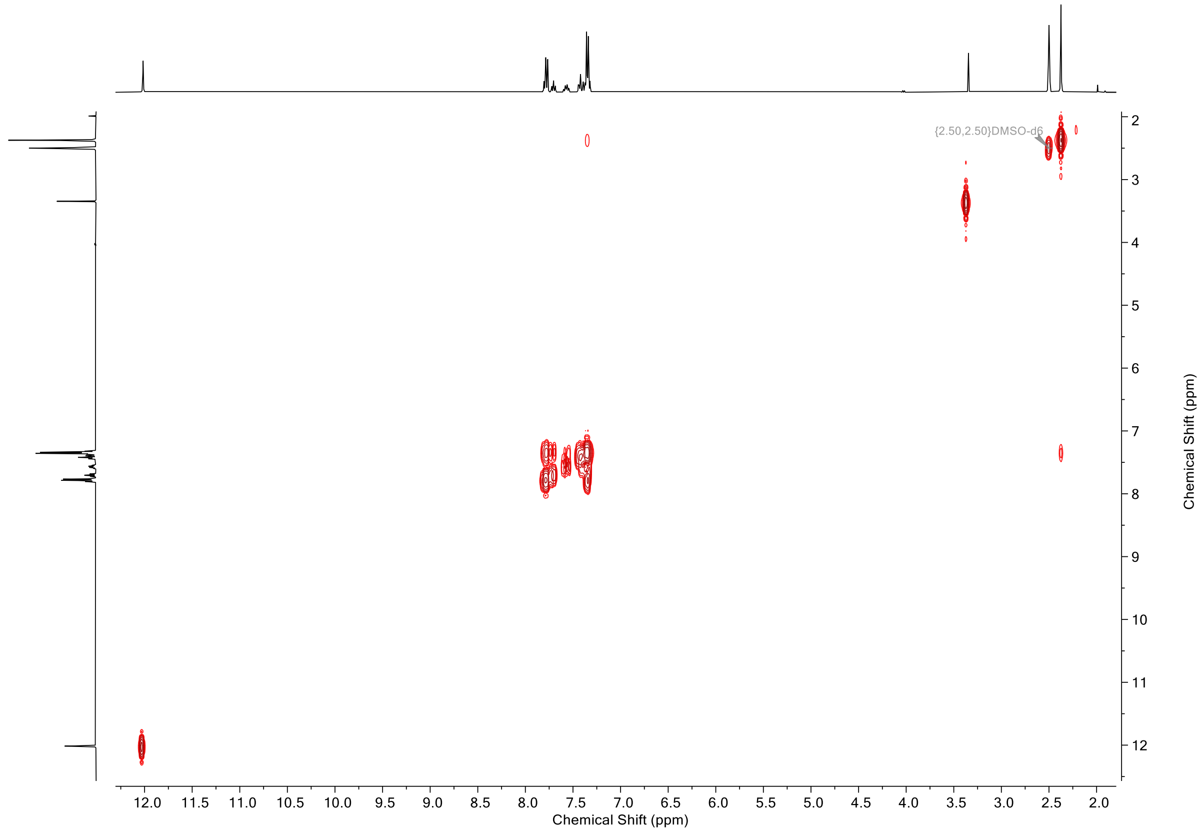

Figure S193: COSY (DMSO-d_6): 1*H*-*N*-(2-(2-Fluorophenyl)-benzo[*d*]imidazol-1-yl)-4-methylbenzamide (**3f**).

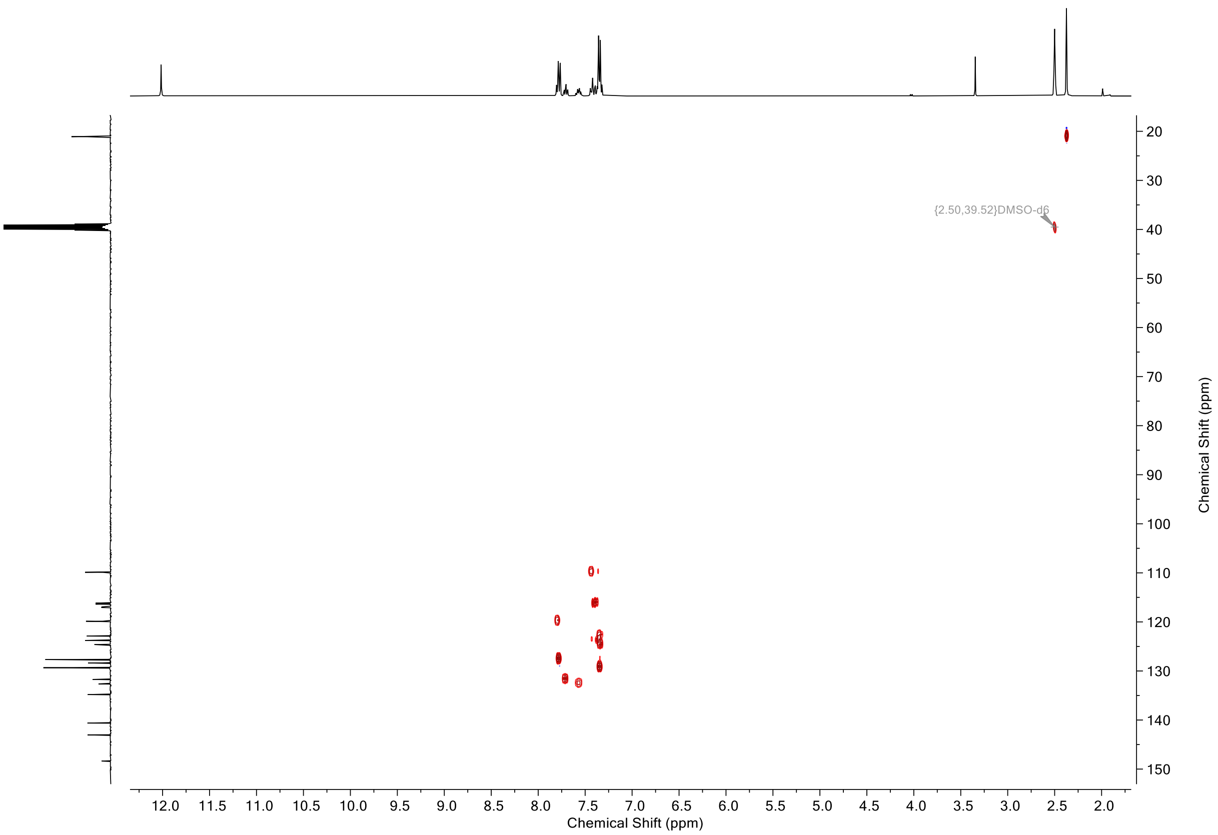

Figure S194: HSQC (DMSO-d_6): 1*H*-*N*-(2-(2-Fluorophenyl)-benzo[*d*]imidazol-1-yl)-4-methylbenzamide (**3f**).

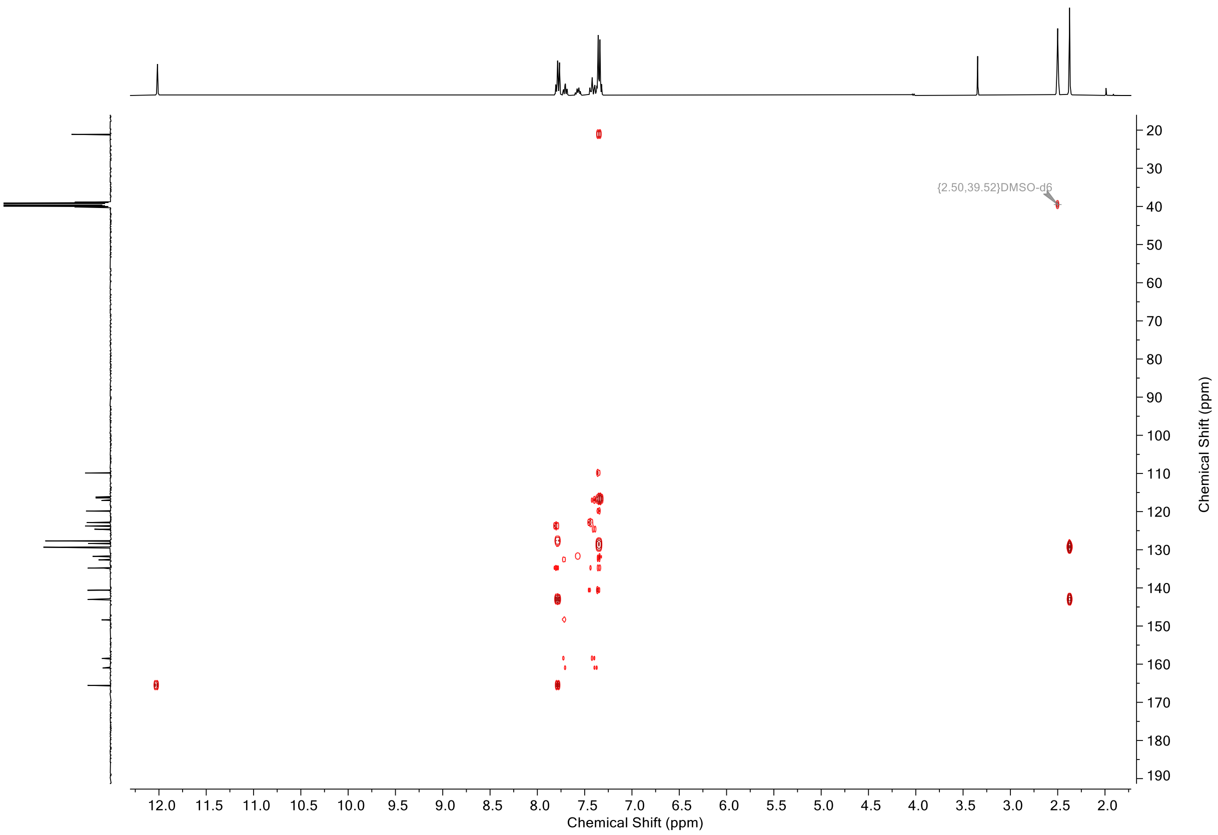

Figure S195: HMBC (DMSO-d_6): 1*H*-*N*-(2-(2-Fluorophenyl)-benzo[*d*]imidazol-1-yl)-4-methylbenzamide (**3f**).

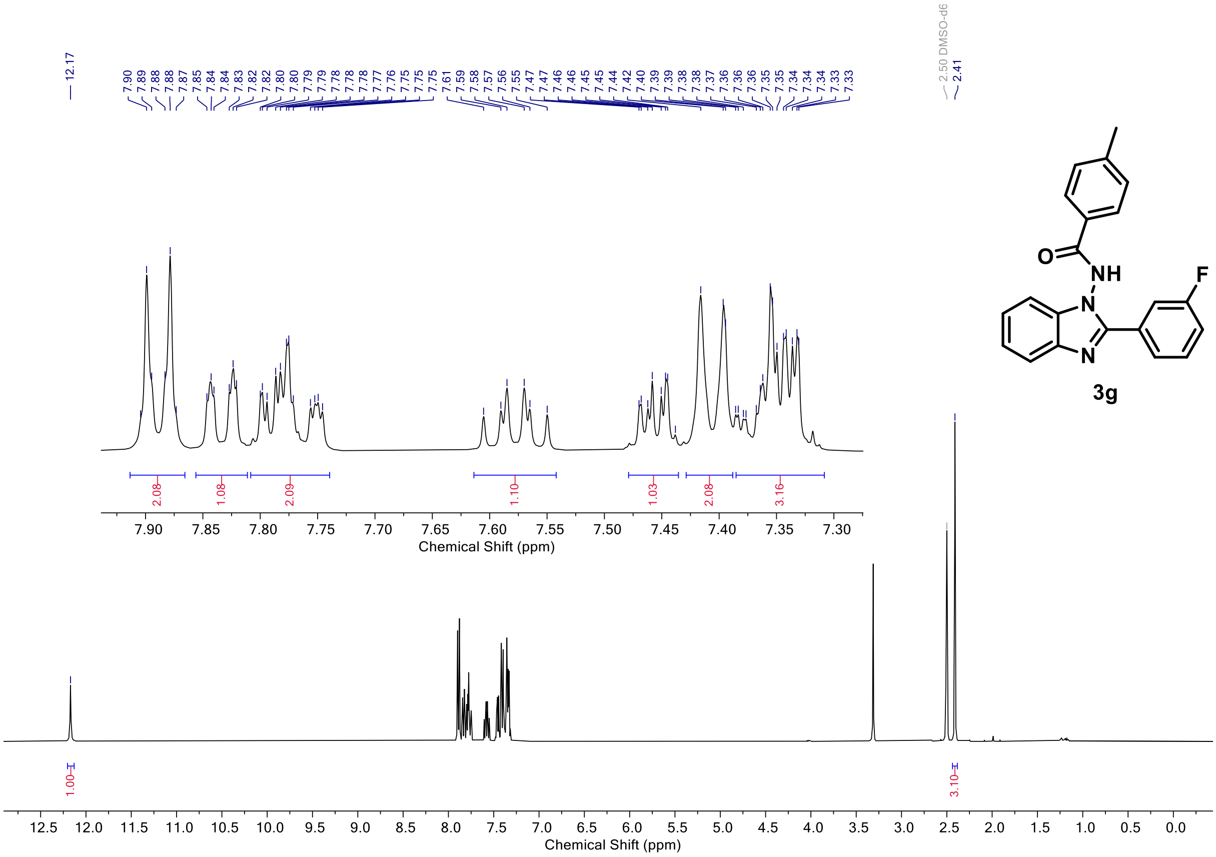

Figure S196: ^{1}H NMR (400 MHz, DMSO-d_6): 1*H*-*N*-(2-(3-Fluorophenyl)-benzo[*d*]imidazol-1-yl)-4-methylbenzamide (**3g**).

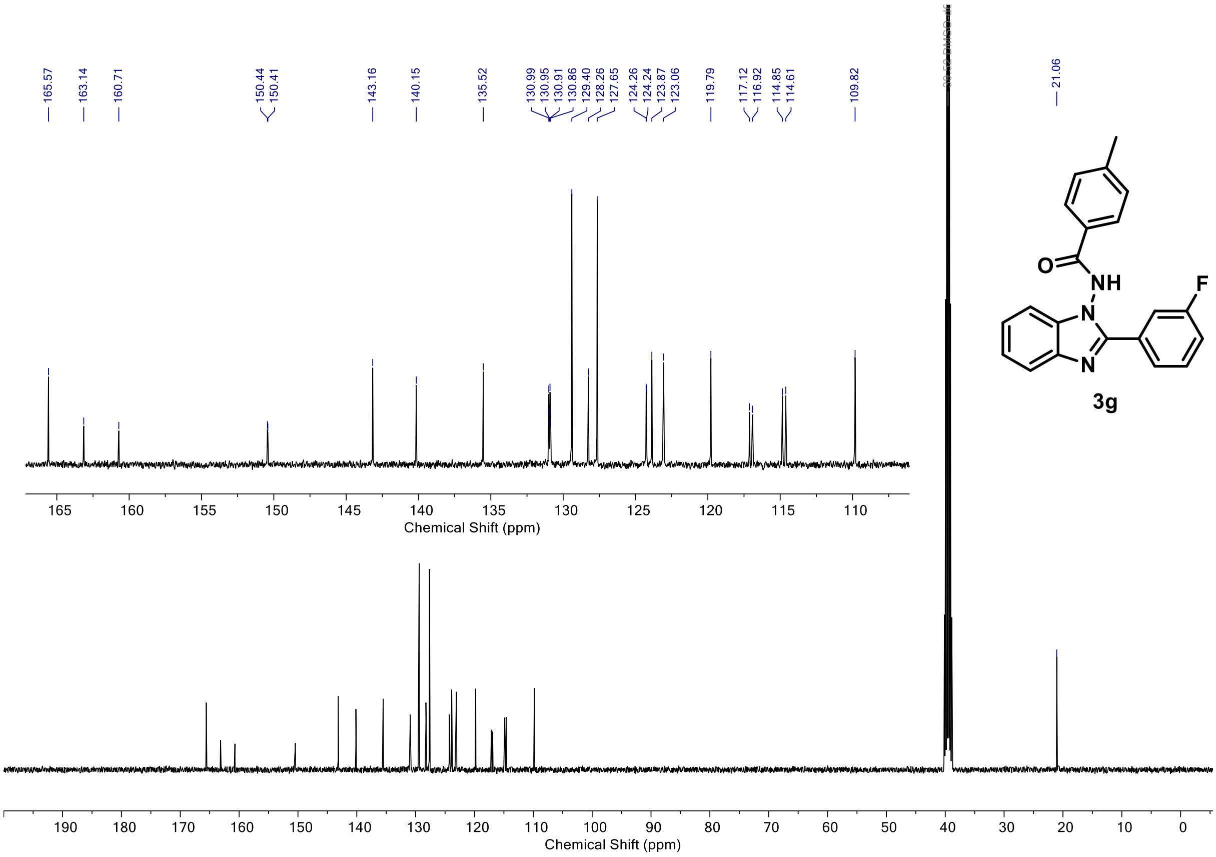

Figure S197: ^{13}C{^{1}H} NMR (101 MHz, DMSO-d_6): 1*H*-*N*-(2-(3-Fluorophenyl)-benzo[*d*]imidazol-1-yl)-4-methylbenzamide (**3g**).

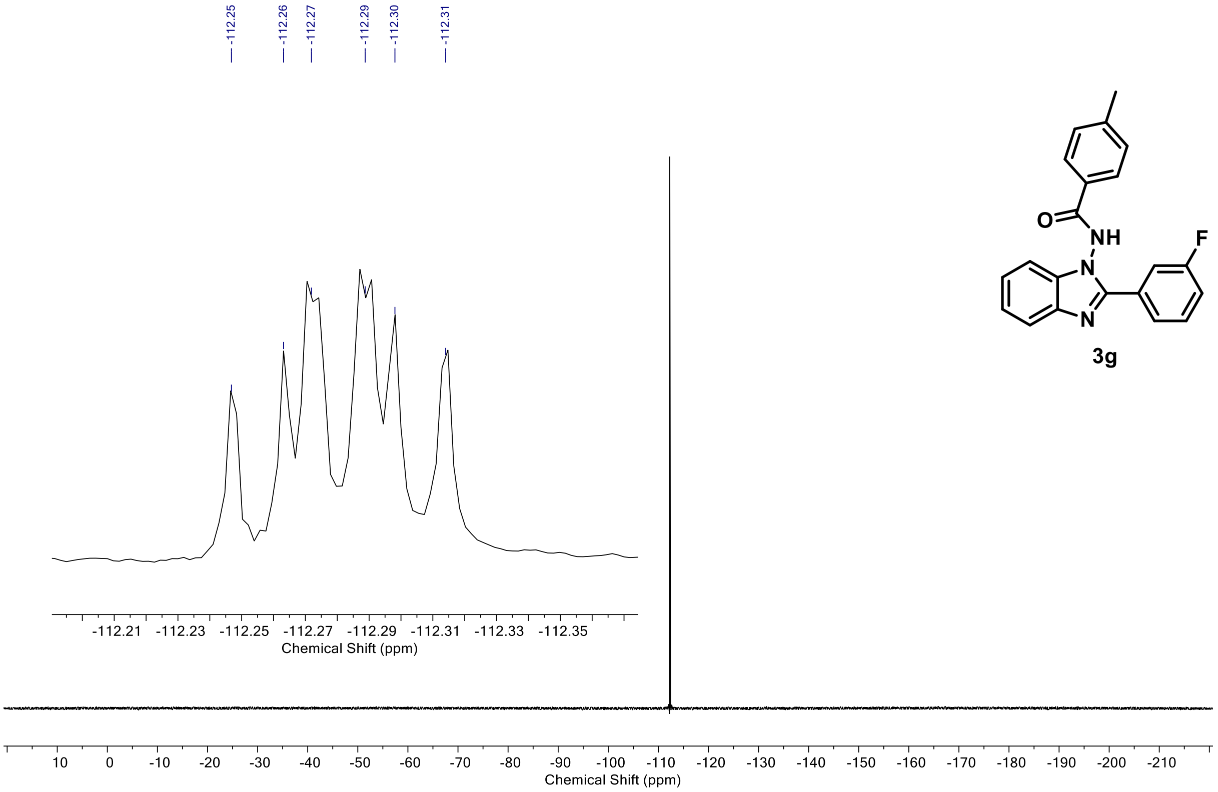

Figure S198: ^{19}F NMR (376 MHz, DMSO-d_6): 1*H*-*N*-(2-(3-Fluorophenyl)-benzo[*d*]imidazol-1-yl)-4-methylbenzamide (**3g**).

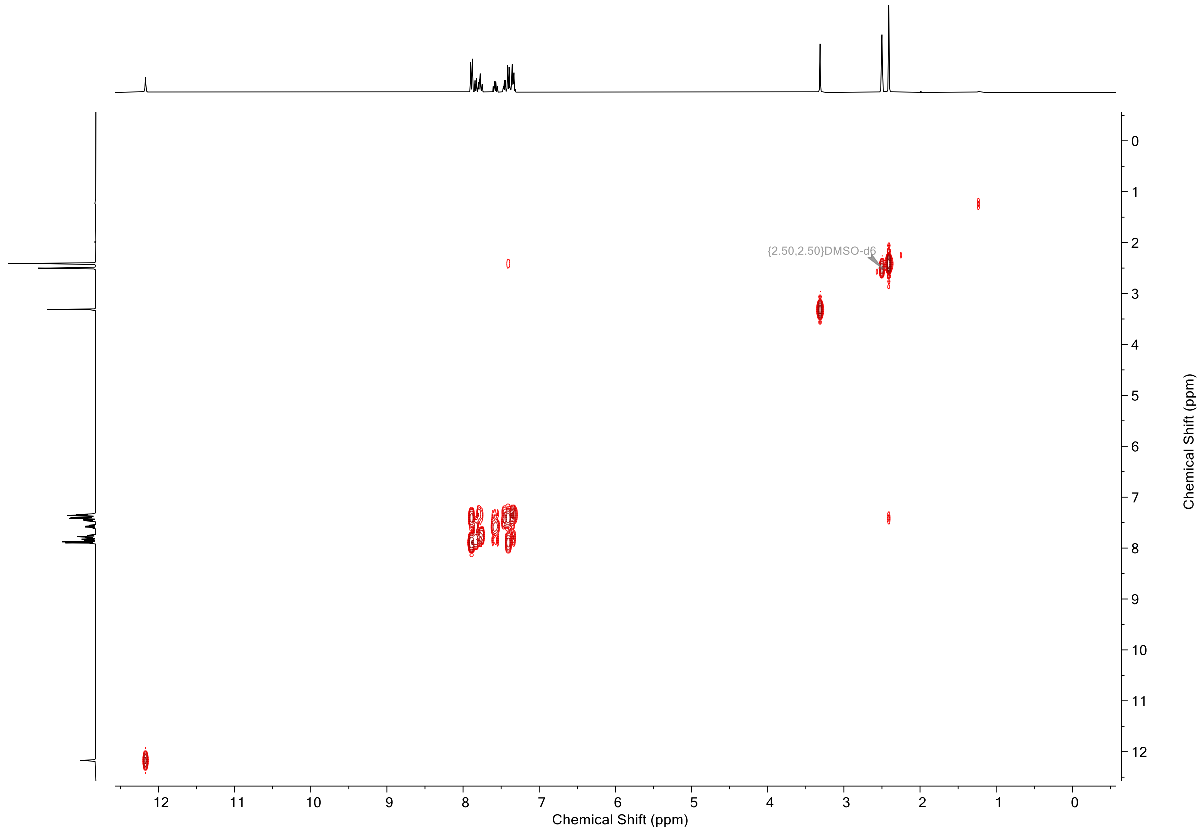

Figure S199: COSY (DMSO-d_6): 1*H*-*N*-(2-(3-Fluorophenyl)-benzo[*d*]imidazol-1-yl)-4-methylbenzamide (**3g**).

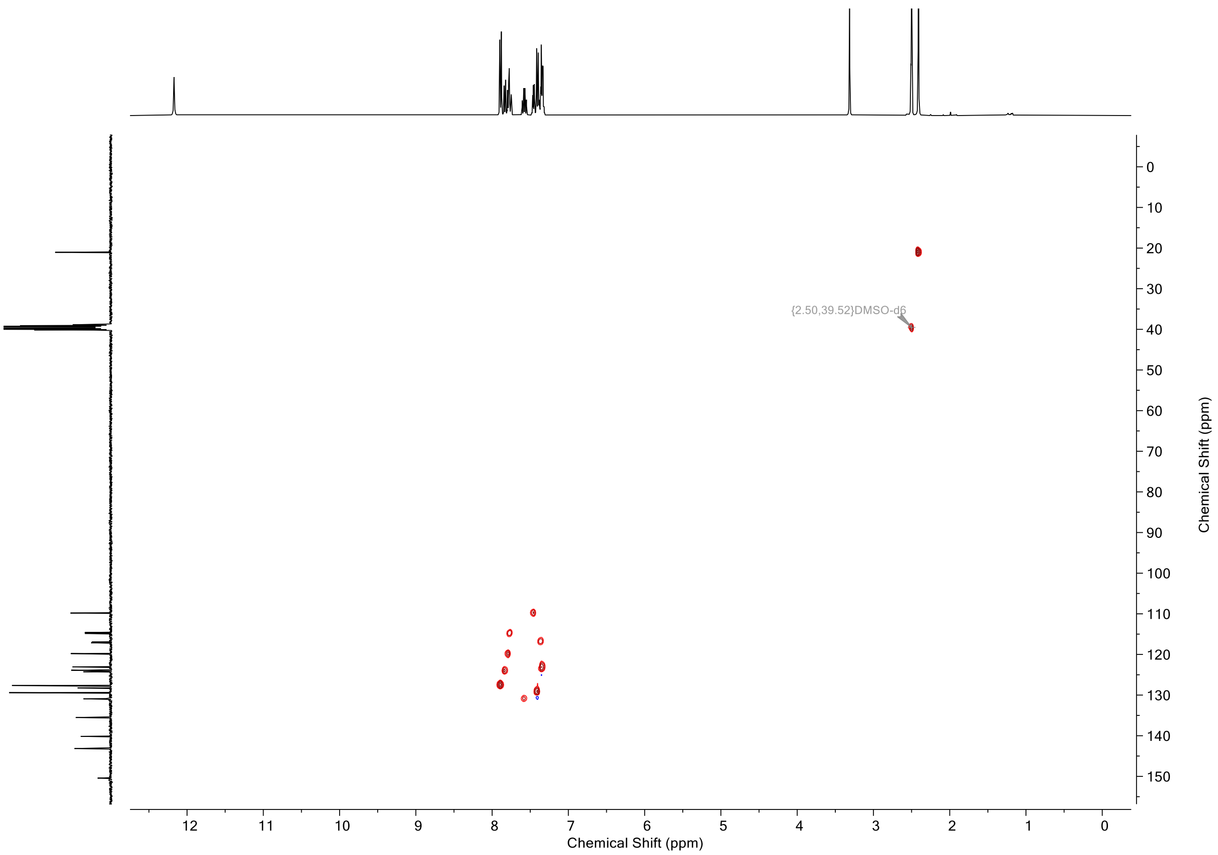

Figure S200: HSQC (DMSO-d_6): 1*H*-*N*-(2-(3-Fluorophenyl)-benzo[*d*]imidazol-1-yl)-4-methylbenzamide (**3g**).

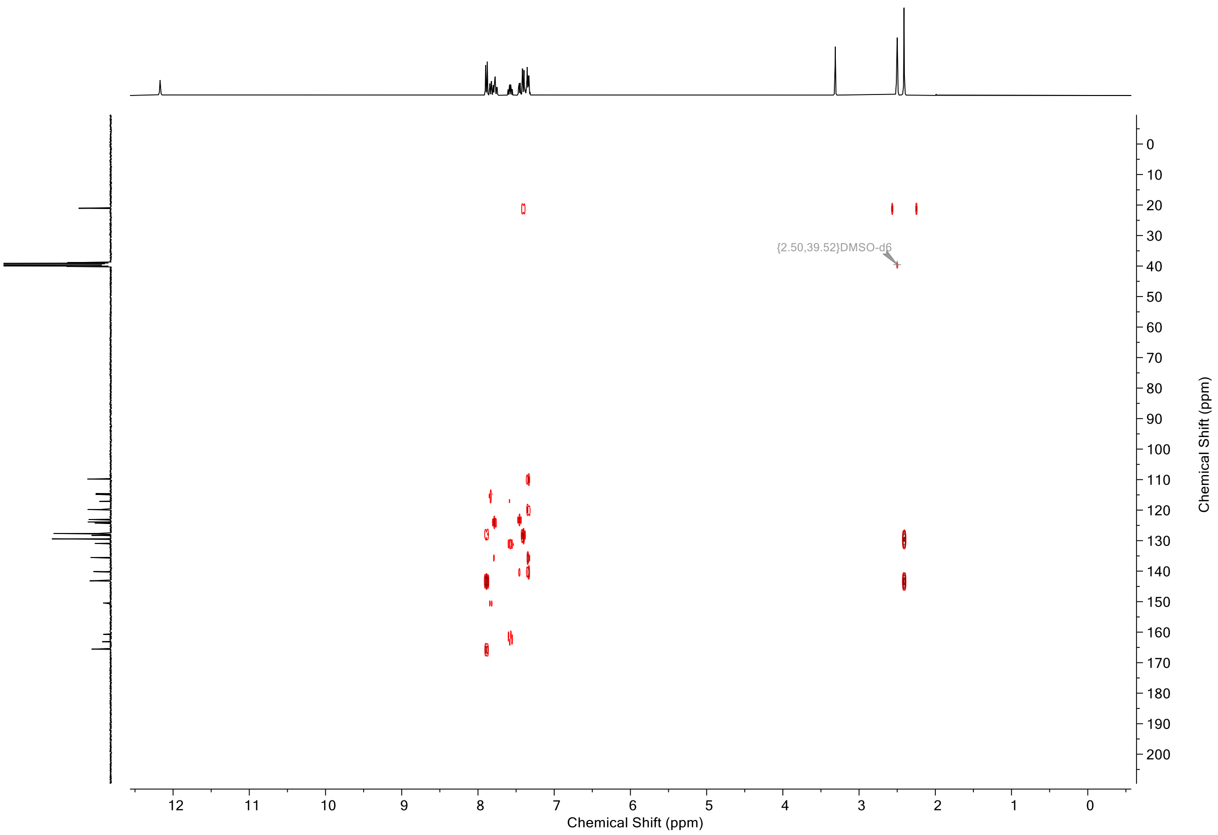

Figure S201: HMBC (DMSO-d_6): 1*H*-*N*-(2-(3-Fluorophenyl)-benzo[*d*]imidazol-1-yl)-4-methylbenzamide (**3g**).

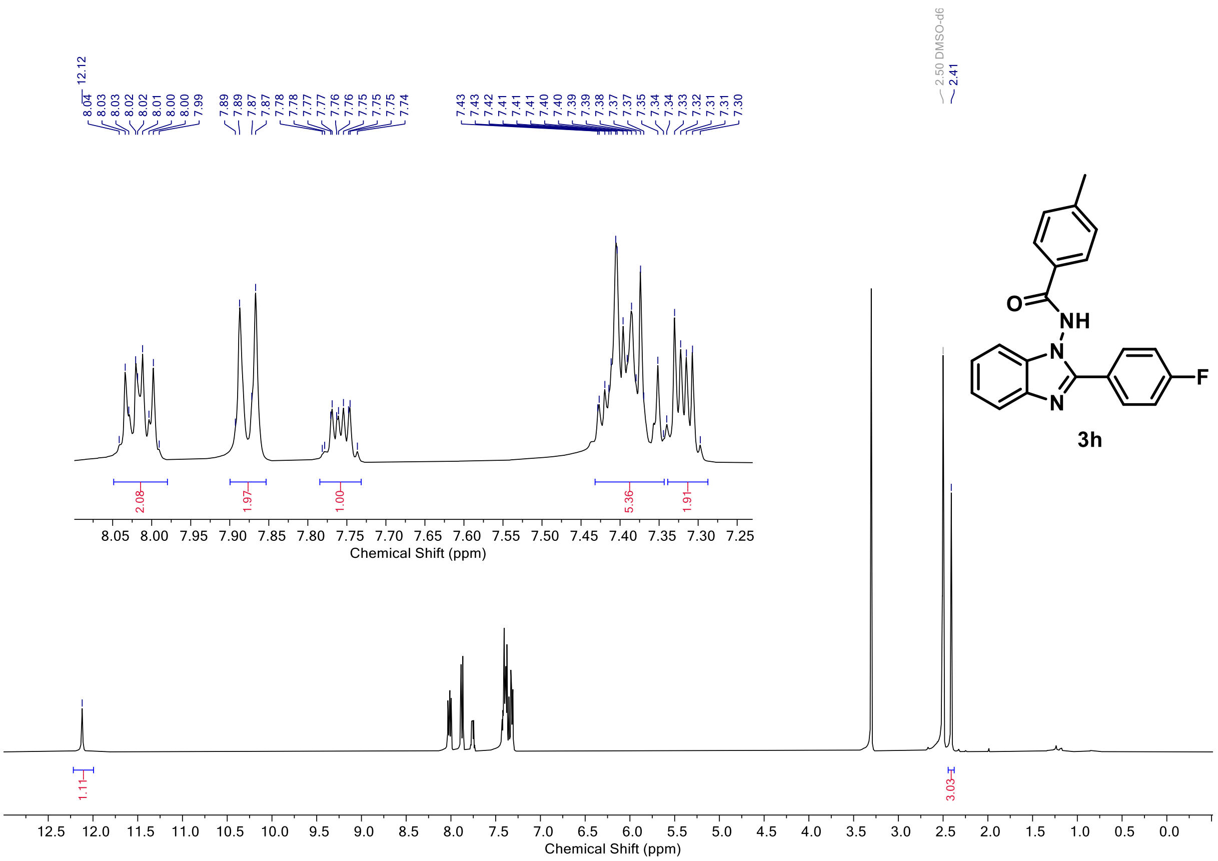

Figure S202: ^{1}H NMR (400 MHz, DMSO-d_6): 1*H*-*N*-(2-(4-Fluorophenyl)-benzo[*d*]imidazol-1-yl)-4-methylbenzamide (**3h**).

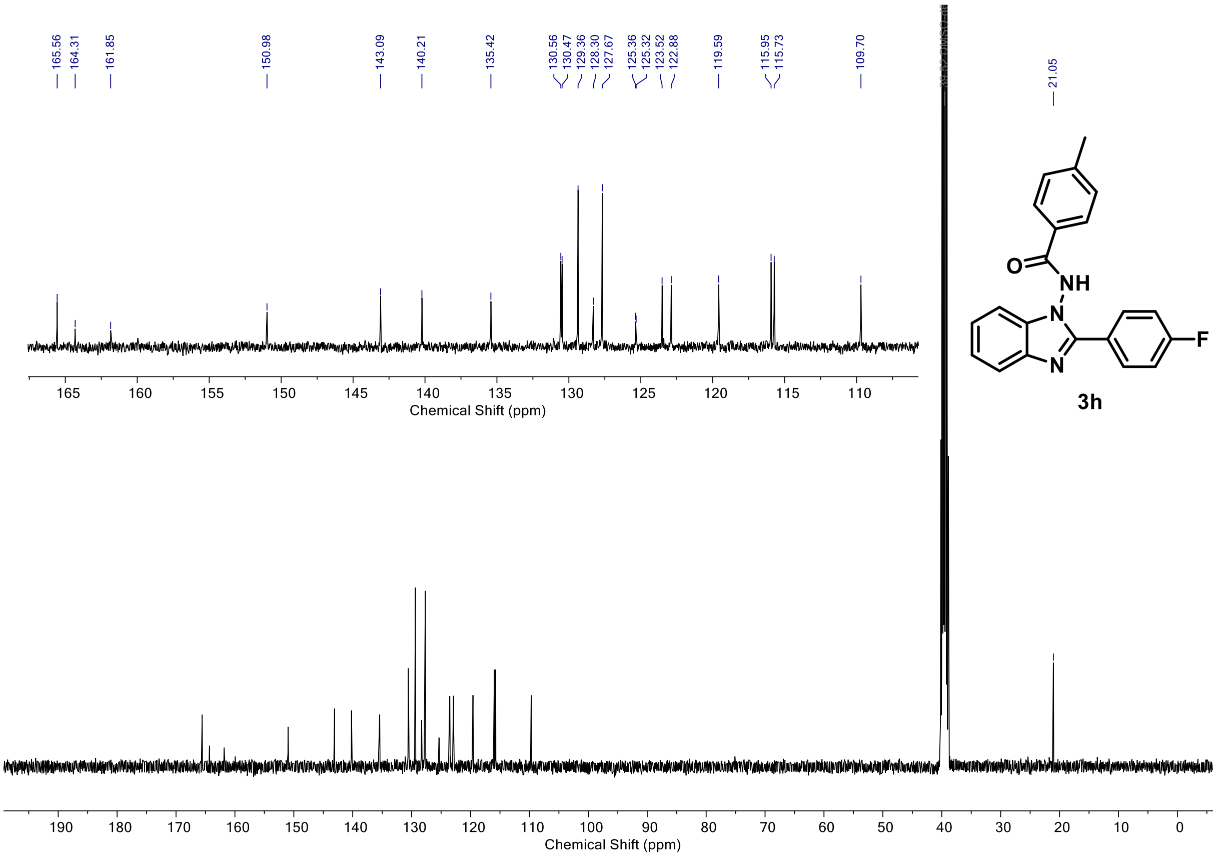

Figure S203: ^{13}C{^{1}H} NMR (101 MHz, DMSO-d_6): 1*H*-*N*-(2-(4-Fluorophenyl)-benzo[*d*]imidazol-1-yl)-4-methylbenzamide (**3h**).

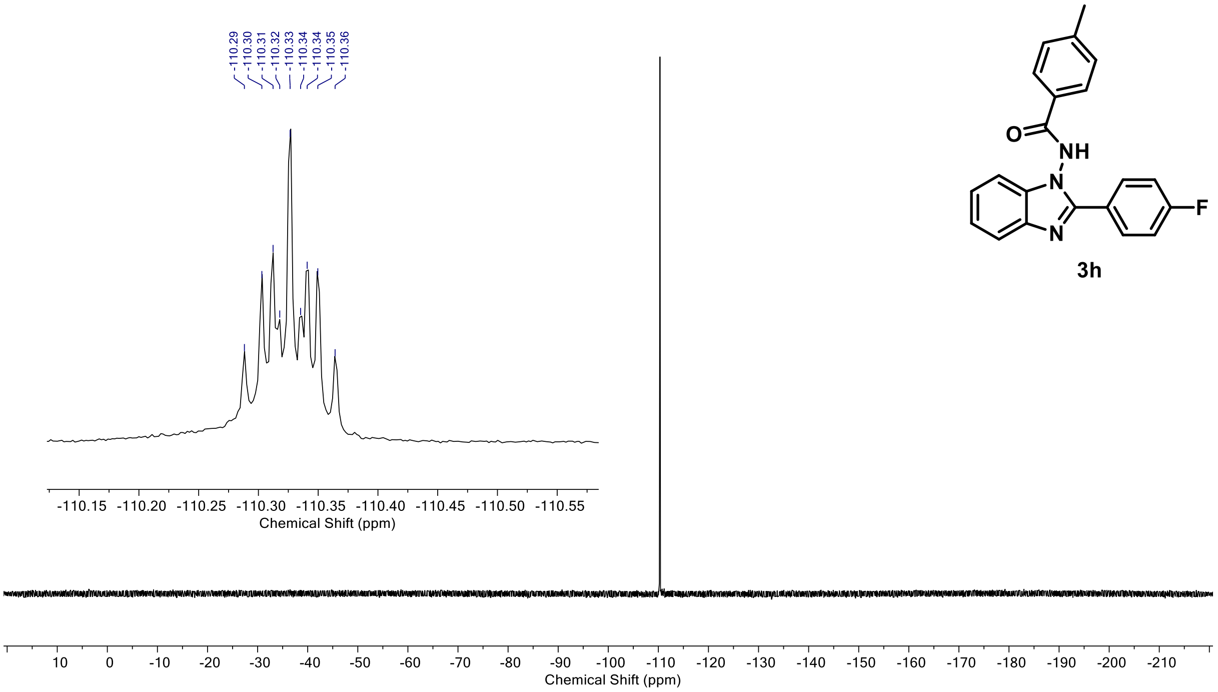

3h

Figure S204: ^{19}F NMR (376 MHz, DMSO-d_6): 1*H*-*N*-(2-(4-Fluorophenyl)-benzo[*d*]imidazol-1-yl)-4-methylbenzamide (**3h**).

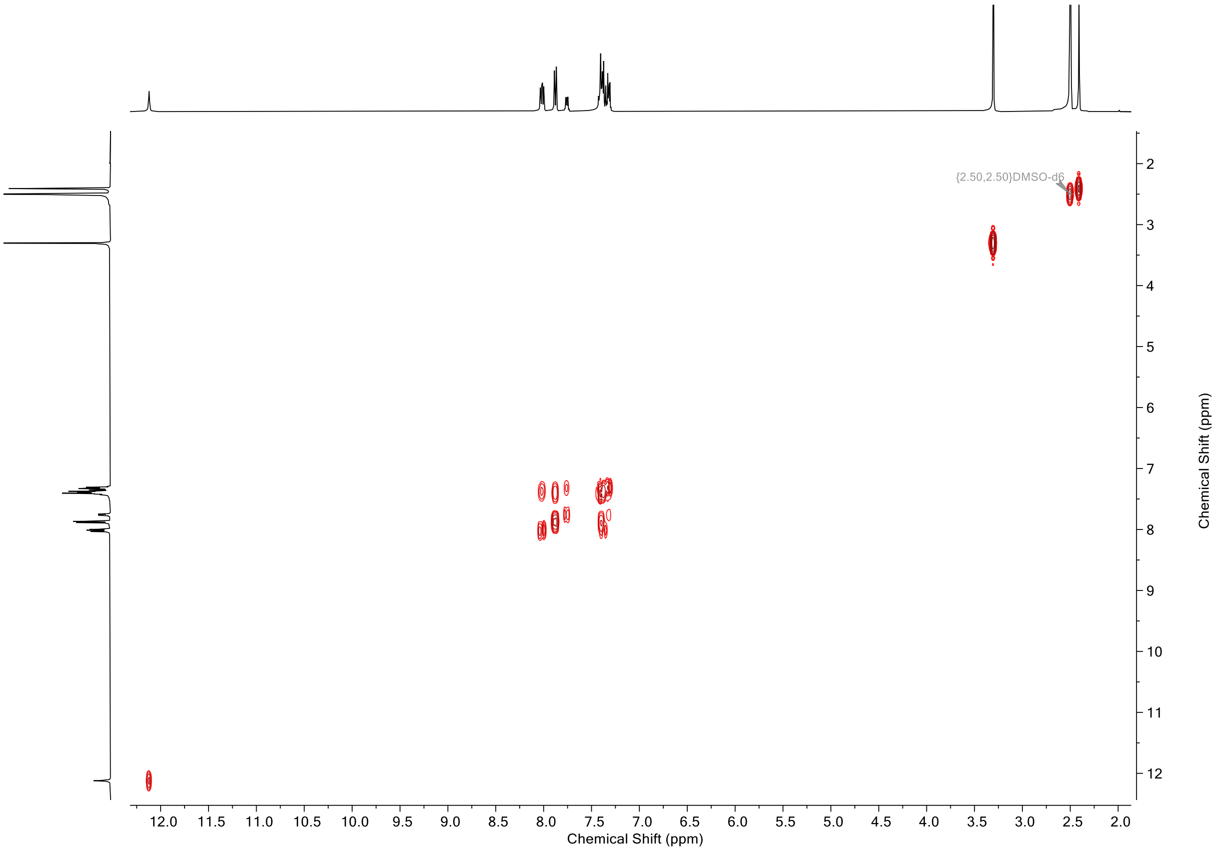

Figure S205: COSY (DMSO-d_6): 1*H*-*N*-(2-(4-Fluorophenyl)-benzo[*d*]imidazol-1-yl)-4-methylbenzamide (**3h**).

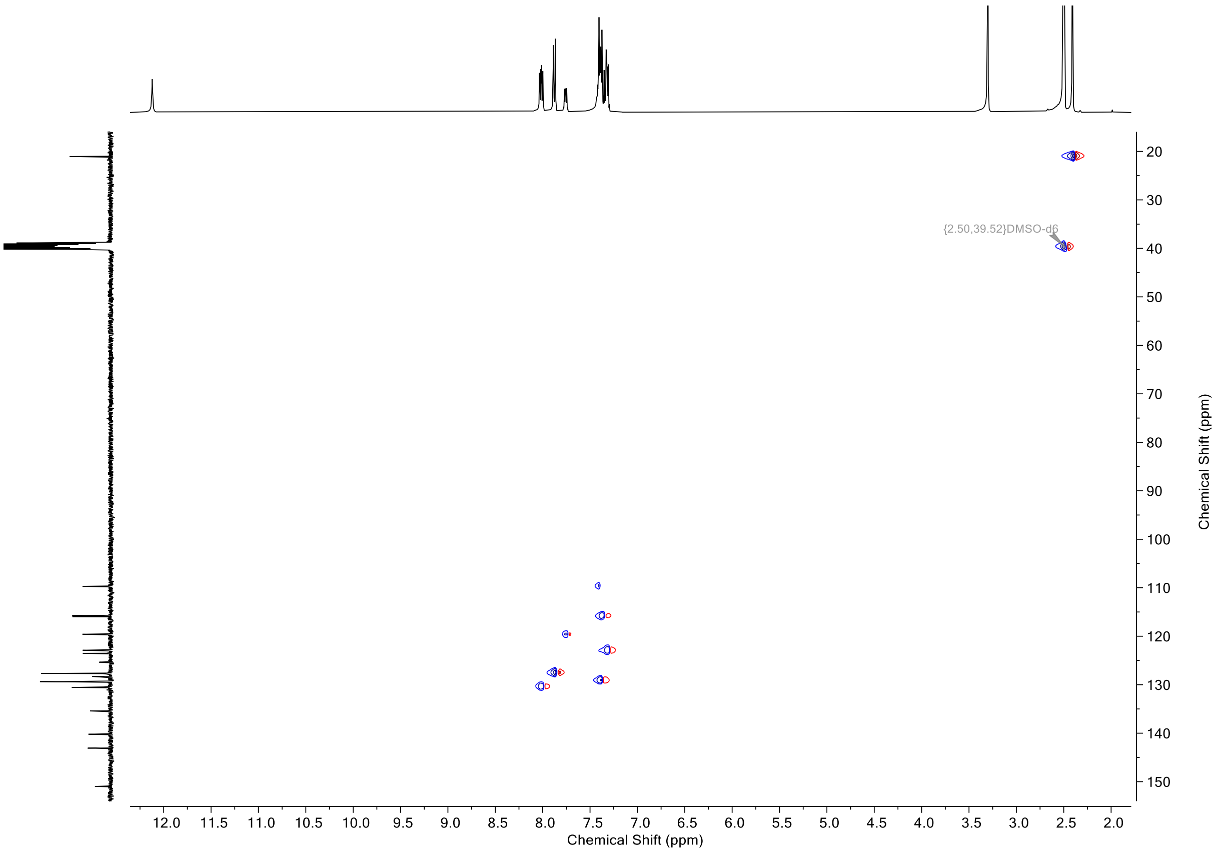

Figure S206: HSQC (DMSO-d_6): 1*H*-*N*-(2-(4-Fluorophenyl)-benzo[*d*]imidazol-1-yl)-4-methylbenzamide (**3h**).

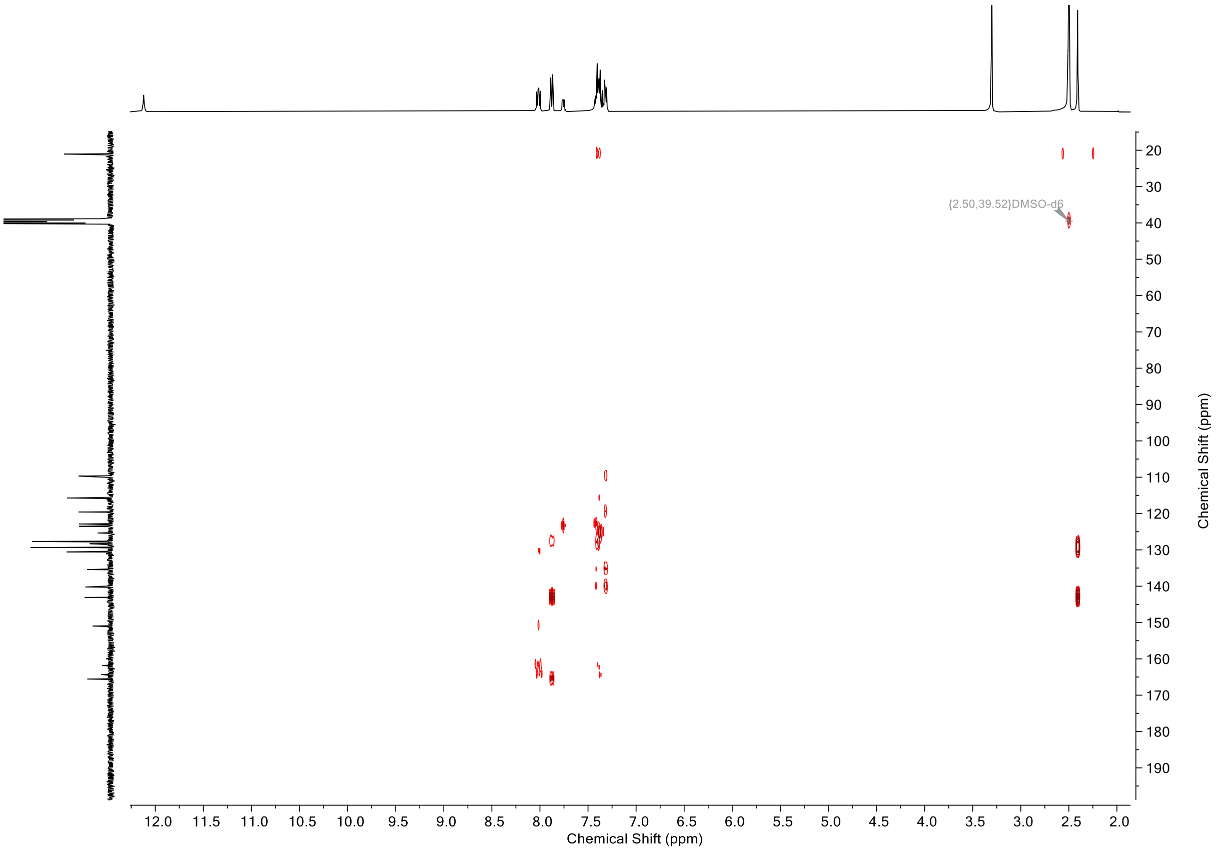

Figure S207: HMBC (DMSO-d_6): 1*H*-*N*-(2-(4-Fluorophenyl)-benzo[*d*]imidazol-1-yl)-4-methylbenzamide (**3h**).

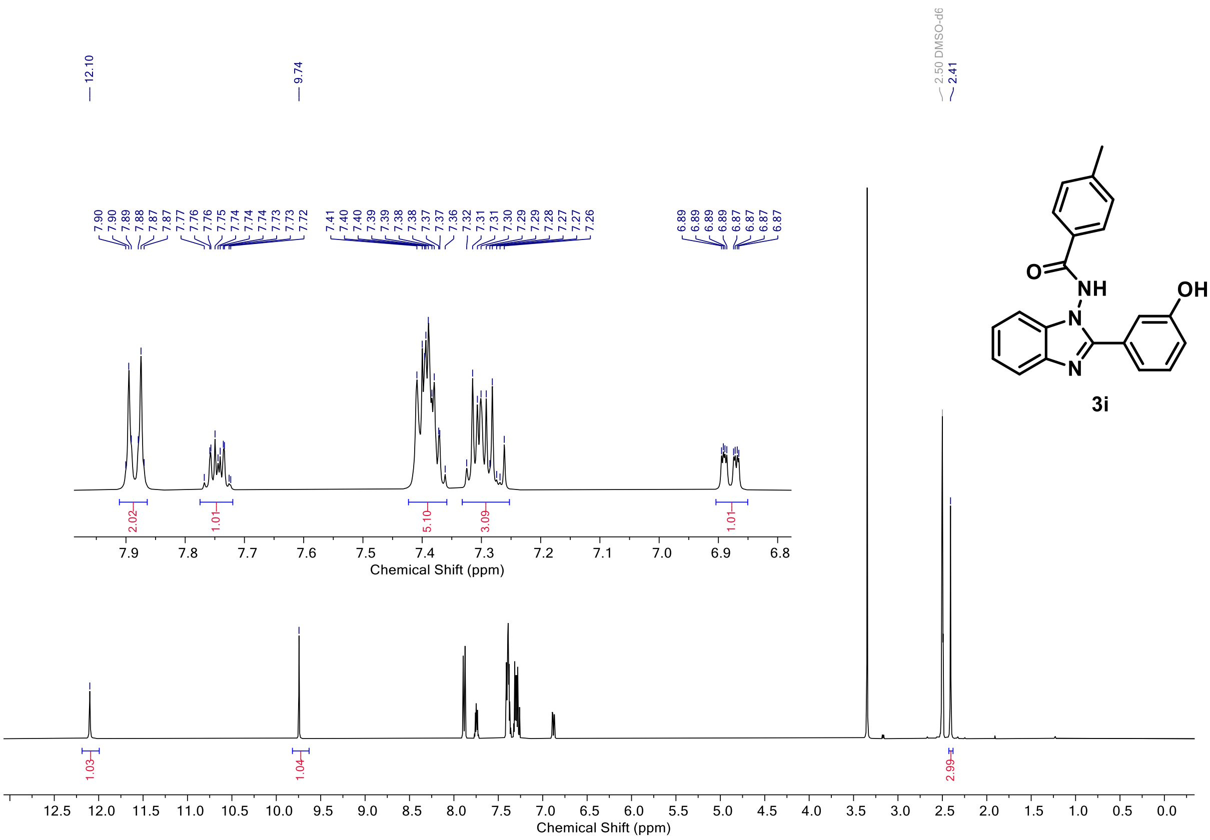

Figure S208: ^{1}H NMR (400 MHz, DMSO-d_6): 1*H*-*N*-(2-(3-Hydroxyphenyl)-benzo[*d*]imidazol-1-yl)-4-methylbenzamide (**3i**).

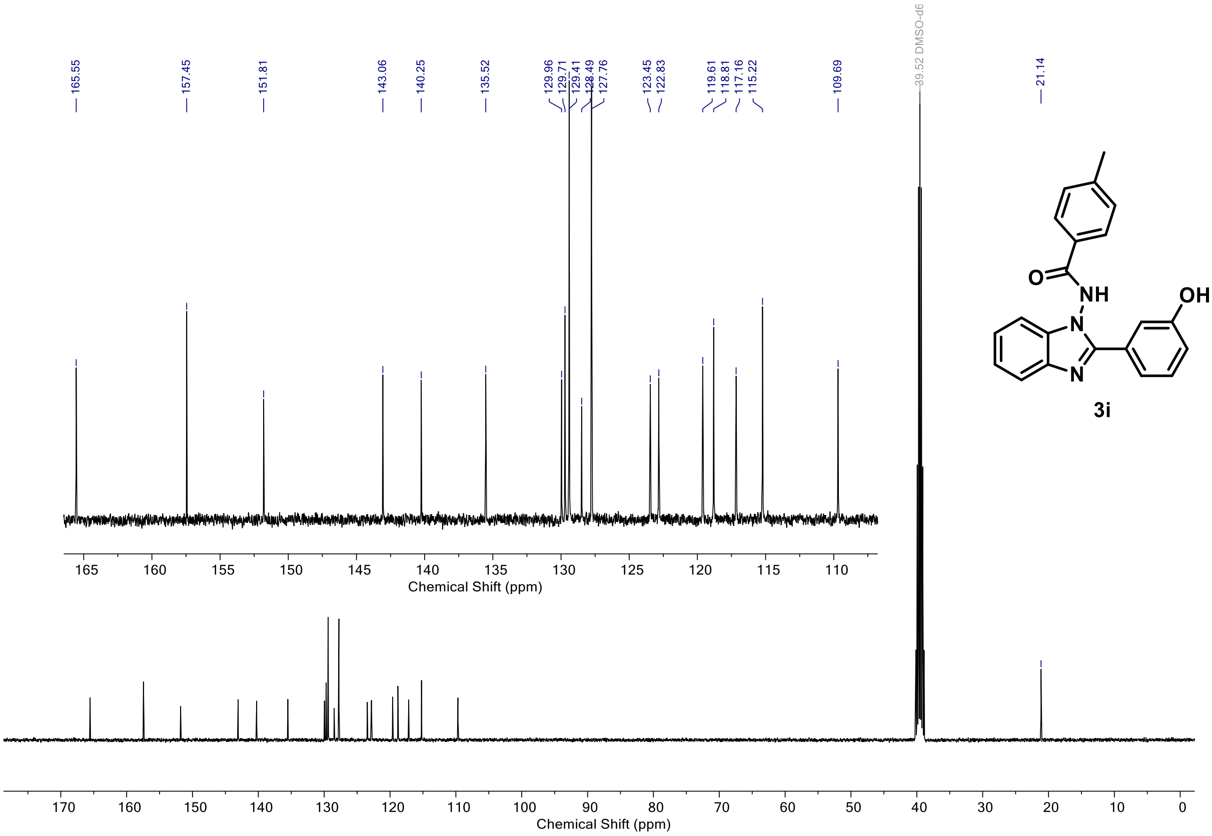

Figure S209: ^{13}C{^{1}H} NMR (101 MHz, DMSO-d_6): 1*H*-*N*-(2-(3-Hydroxyphenyl)-benzo[*d*]imidazol-1-yl)-4-methylbenzamide (**3i**).

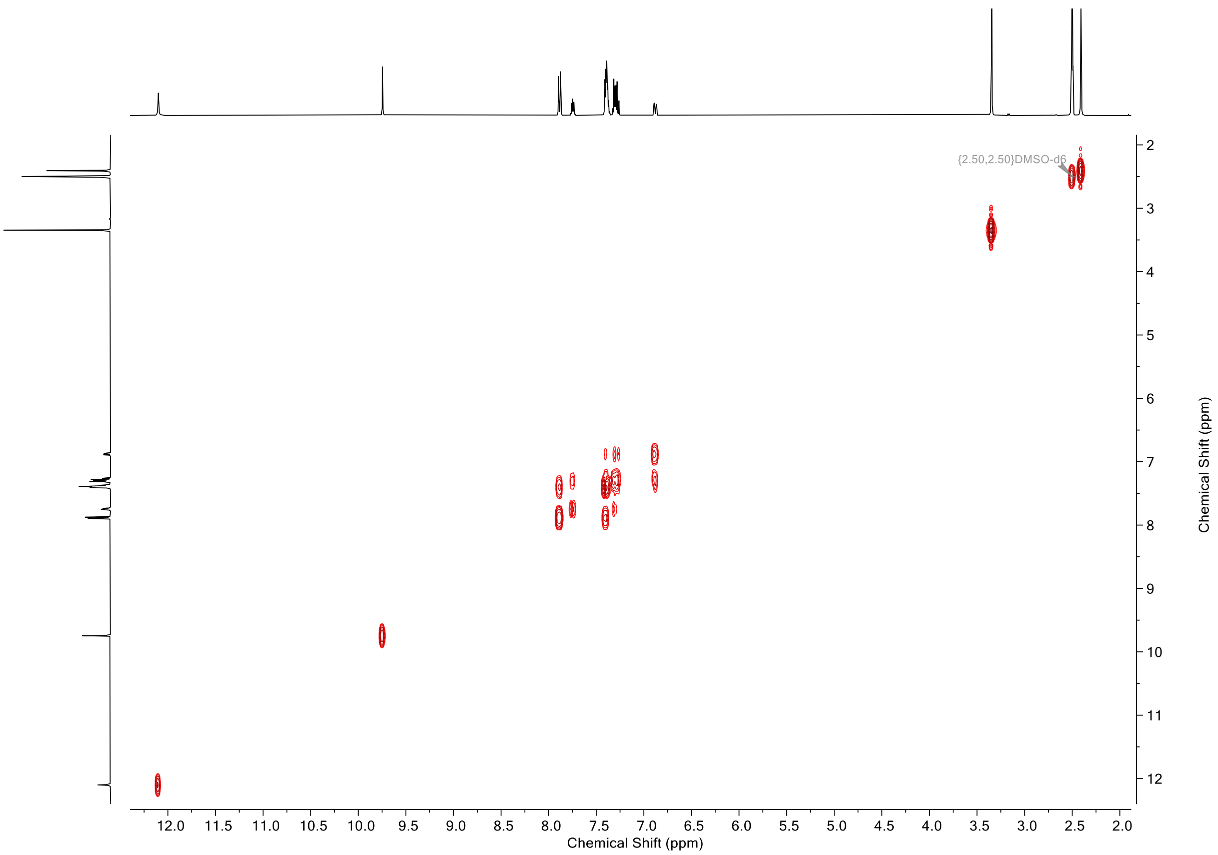

Figure S210: COSY (DMSO-d_6): 1*H*-*N*-(2-(3-Hydroxyphenyl)-benzo[*d*]imidazol-1-yl)-4-methylbenzamide (**3i**).

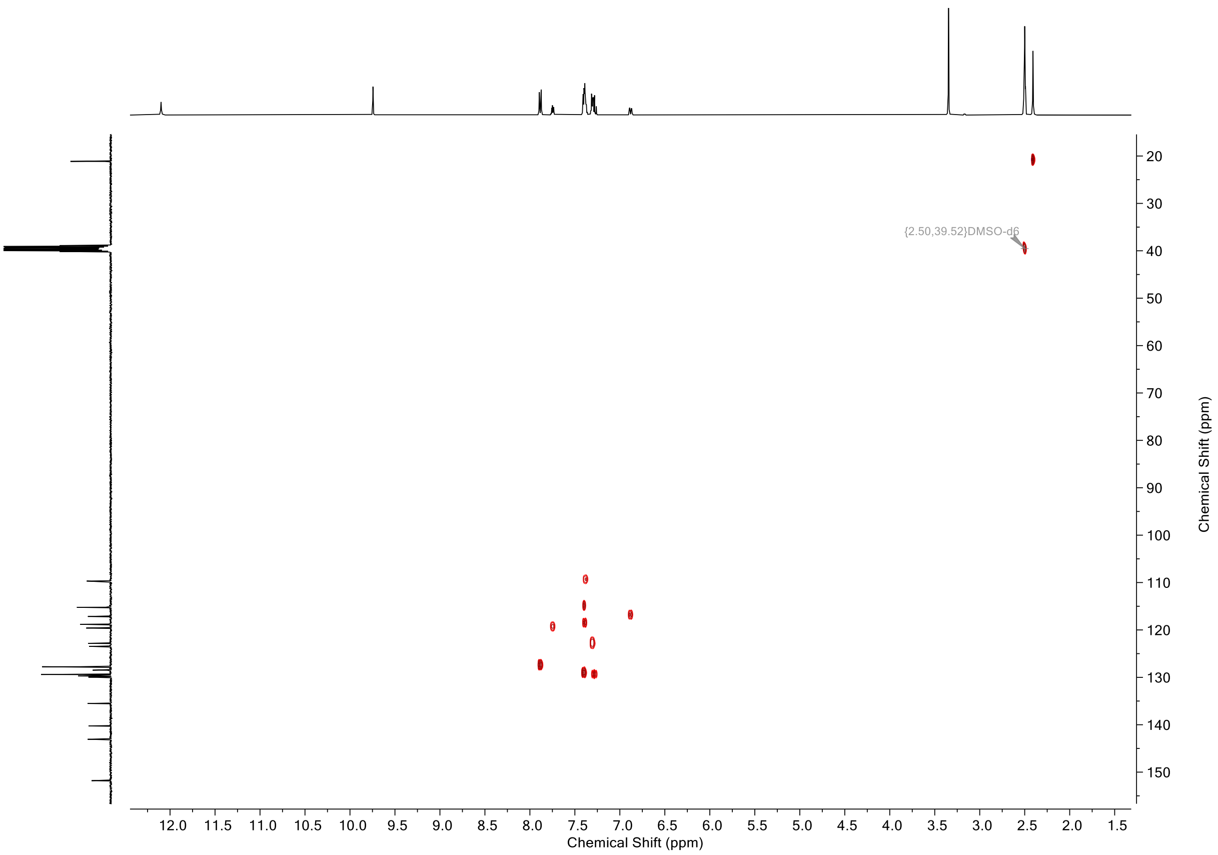

Figure S211: HSQC (DMSO-d_6): 1*H*-*N*-(2-(3-Hydroxyphenyl)-benzo[*d*]imidazol-1-yl)-4-methylbenzamide (**3i**).

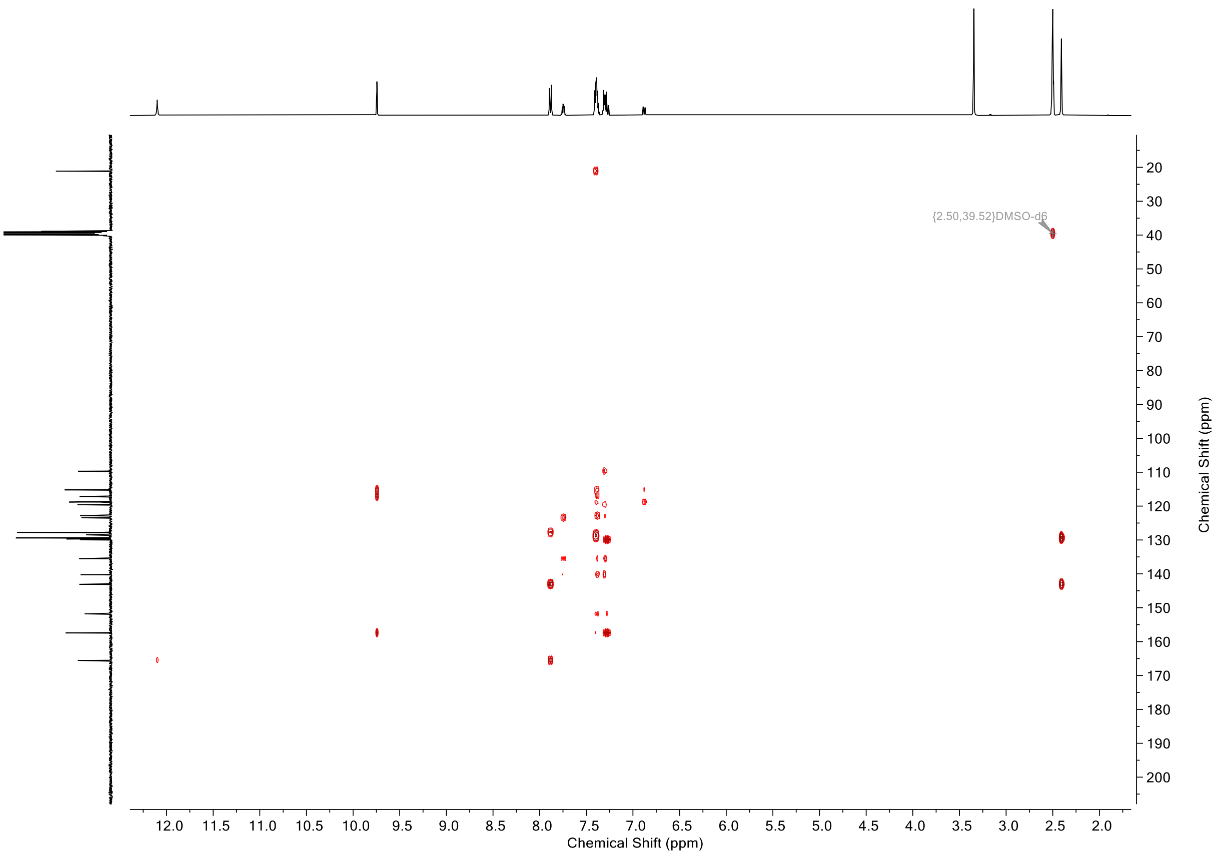

Figure S212: HMBC (DMSO-d_6): 1*H*-*N*-(2-(3-Hydroxyphenyl)-benzo[*d*]imidazol-1-yl)-4-methylbenzamide (**3i**).

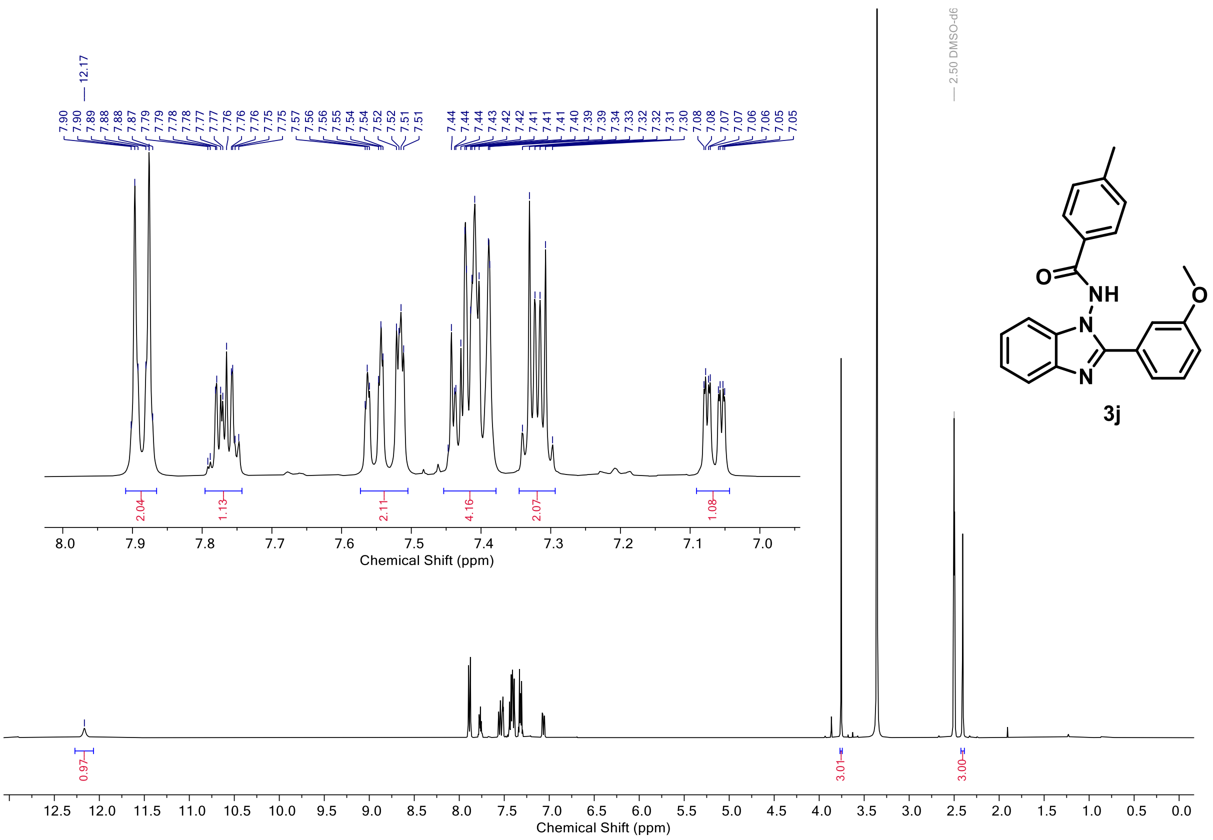

Figure S213: ^{1}H NMR (400 MHz, DMSO-d_6): 1*H*-*N*-(2-(3-Methoxyphenyl)-benzo[*d*]imidazol-1-yl)-4-methylbenzamide (**3j**).

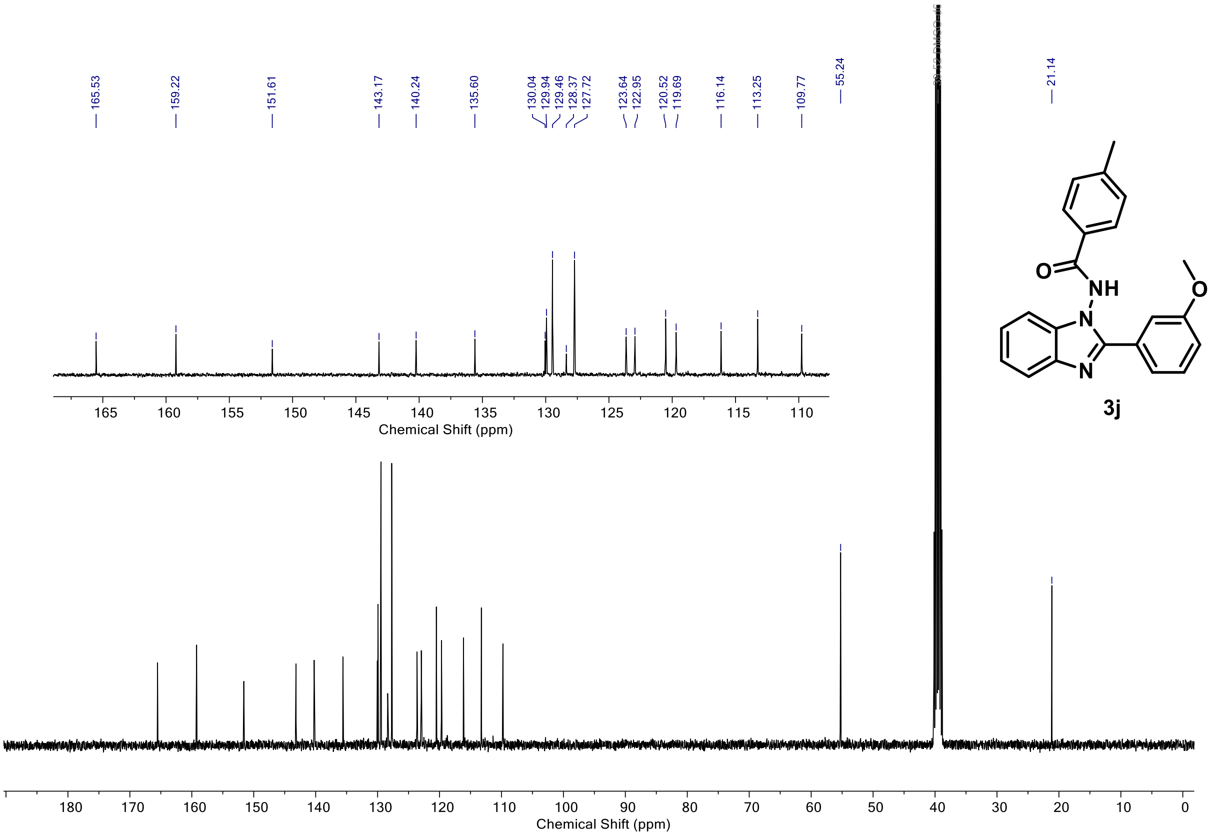

Figure S214: ^{13}C{^{1}H} NMR (101 MHz, DMSO-d_6): 1*H*-*N*-(2-(3-Methoxyphenyl)-benzo[*d*]imidazol-1-yl)-4-methylbenzamide (**3j**).

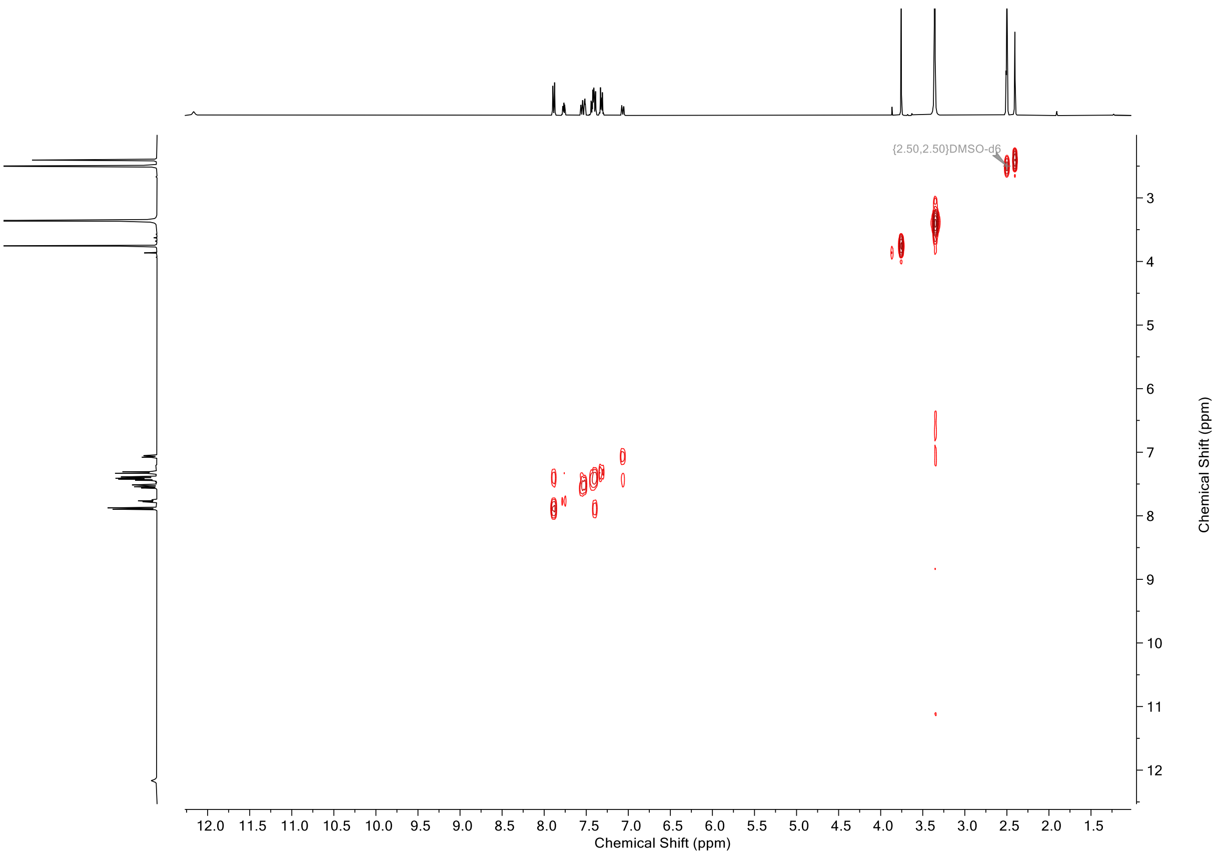

Figure S215: COSY (DMSO-d_6): 1*H*-*N*-(2-(3-Methoxyphenyl)-benzo[*d*]imidazol-1-yl)-4-methylbenzamide (**3j**).

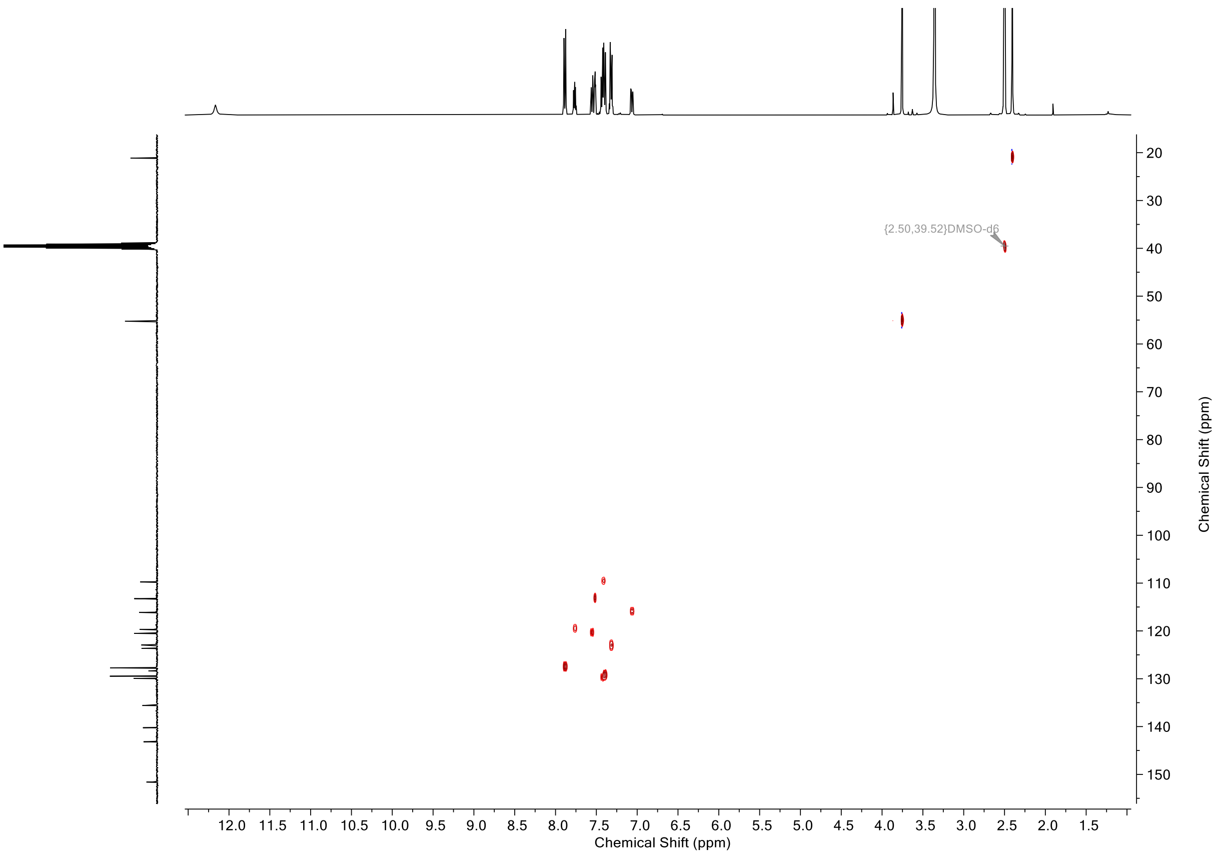

Figure S216: HSQC (DMSO-d_6): 1*H*-*N*-(2-(3-Methoxyphenyl)-benzo[*d*]imidazol-1-yl)-4-methylbenzamide (**3j**).

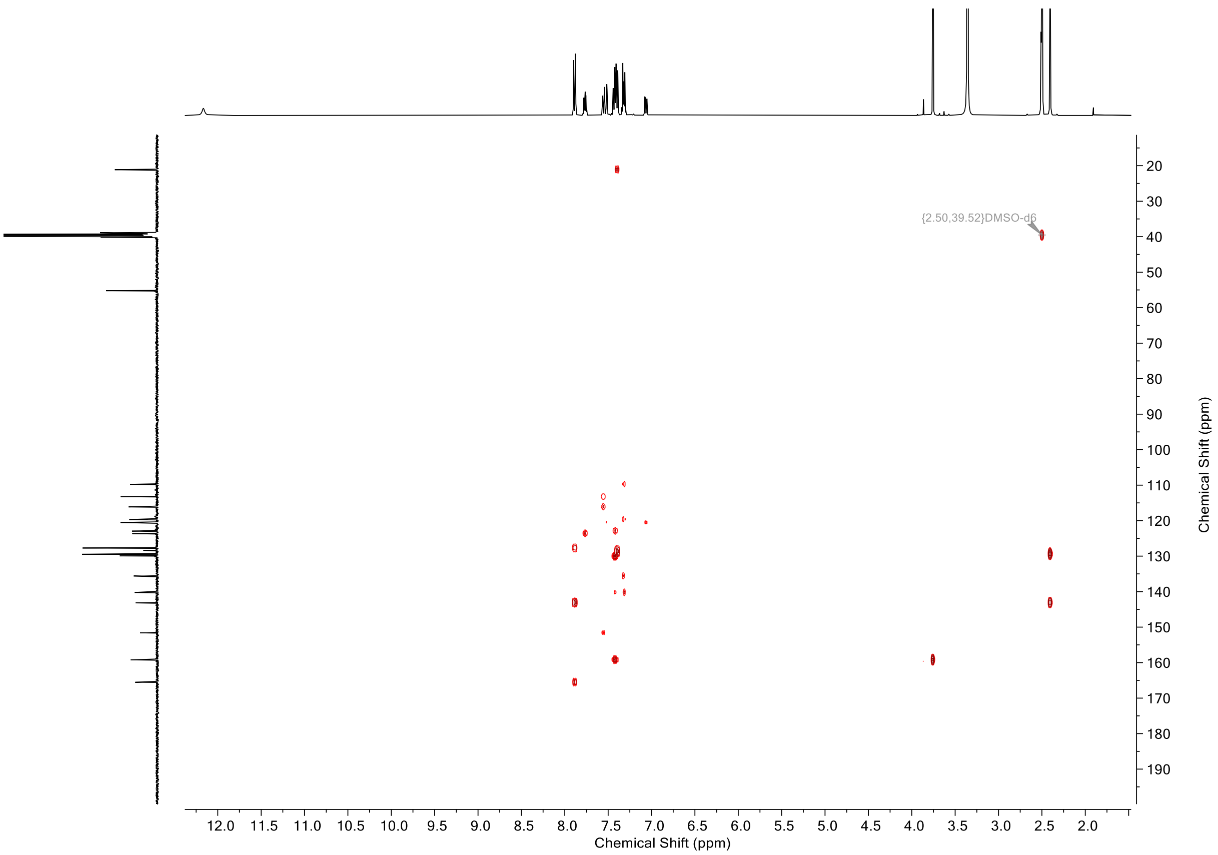

Figure S217: HMBC (DMSO-d_6): 1*H*-*N*-(2-(3-Methoxyphenyl)-benzo[*d*]imidazol-1-yl)-4-methylbenzamide (**3j**).

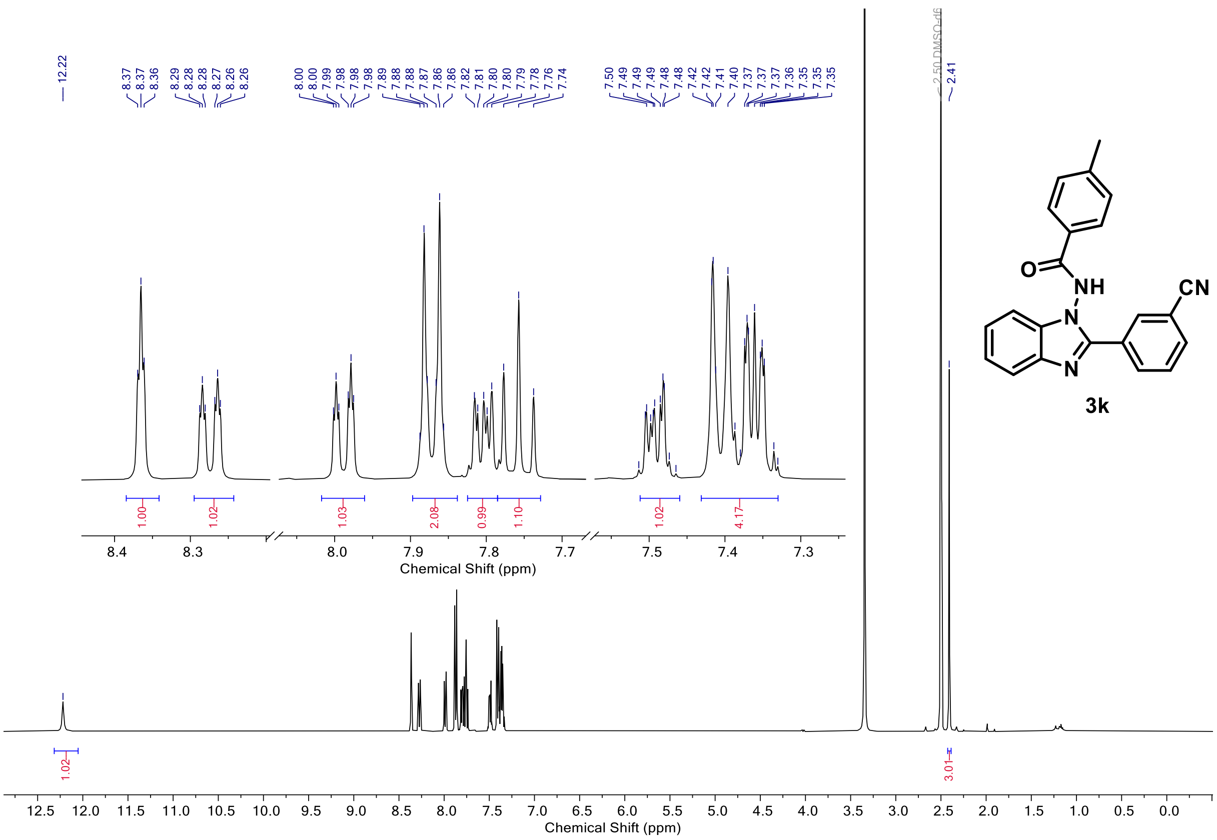

Figure S218: ^{1}H NMR (400 MHz, DMSO-d_6): 1*H*-*N*-(2-(3-Cyanophenyl)-benzo[*d*]imidazol-1-yl)-4-methylbenzamide (**3k**).

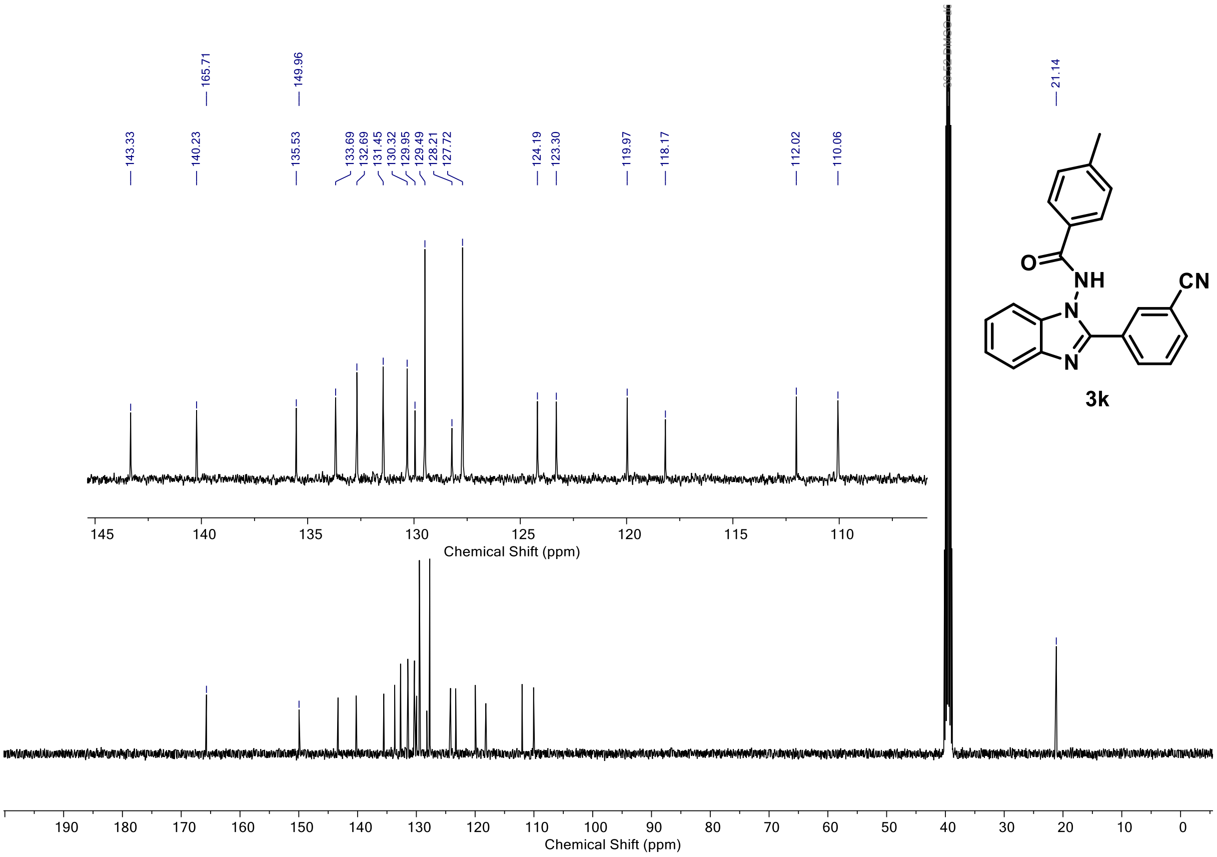

Figure S219: ^{13}C{^{1}H} NMR (101 MHz, DMSO-d_6): 1*H*-*N*-(2-(3-Cyanophenyl)-benzo[*d*]imidazol-1-yl)-4-methylbenz-amide (**3k**).

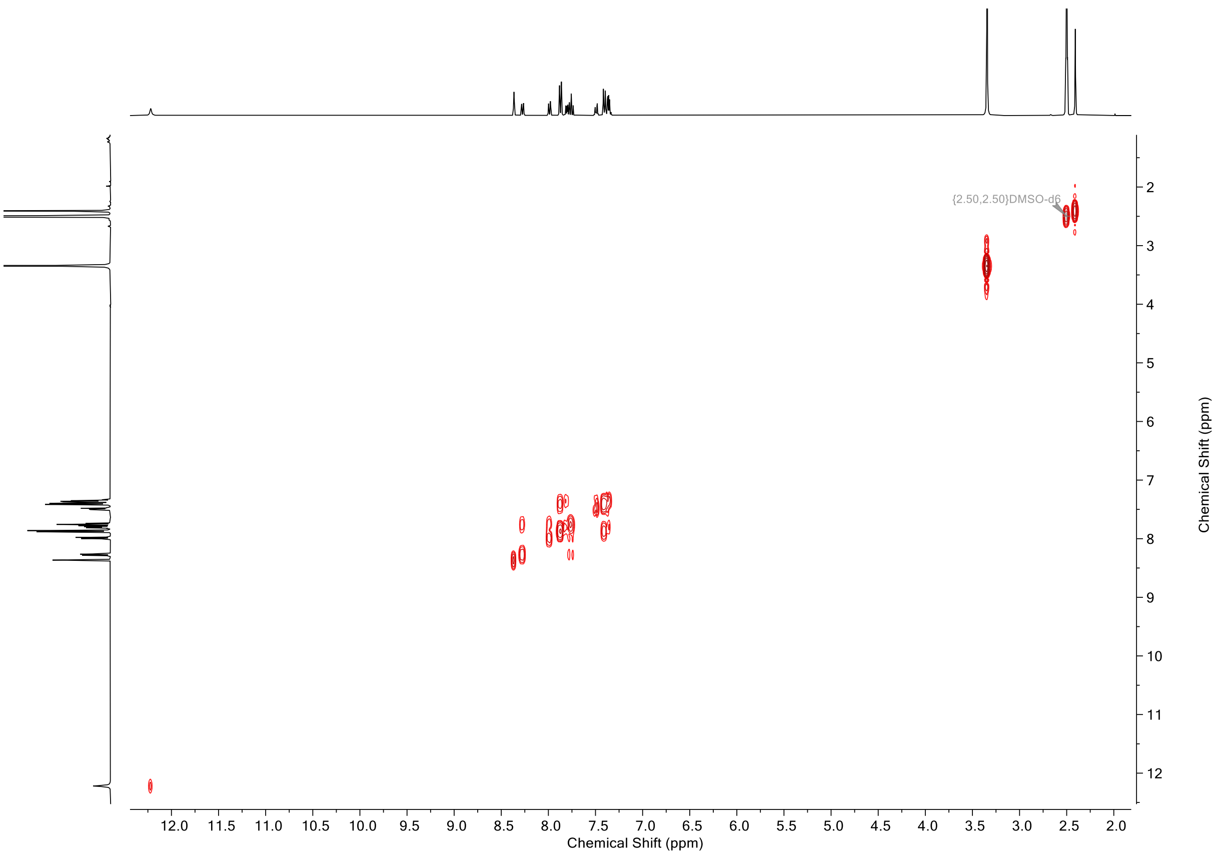

Figure S220: COSY (DMSO-d_6): 1*H*-*N*-(2-(3-Cyanophenyl)-benzo[*d*]imidazol-1-yl)-4-methylbenz-amide (**3k**).

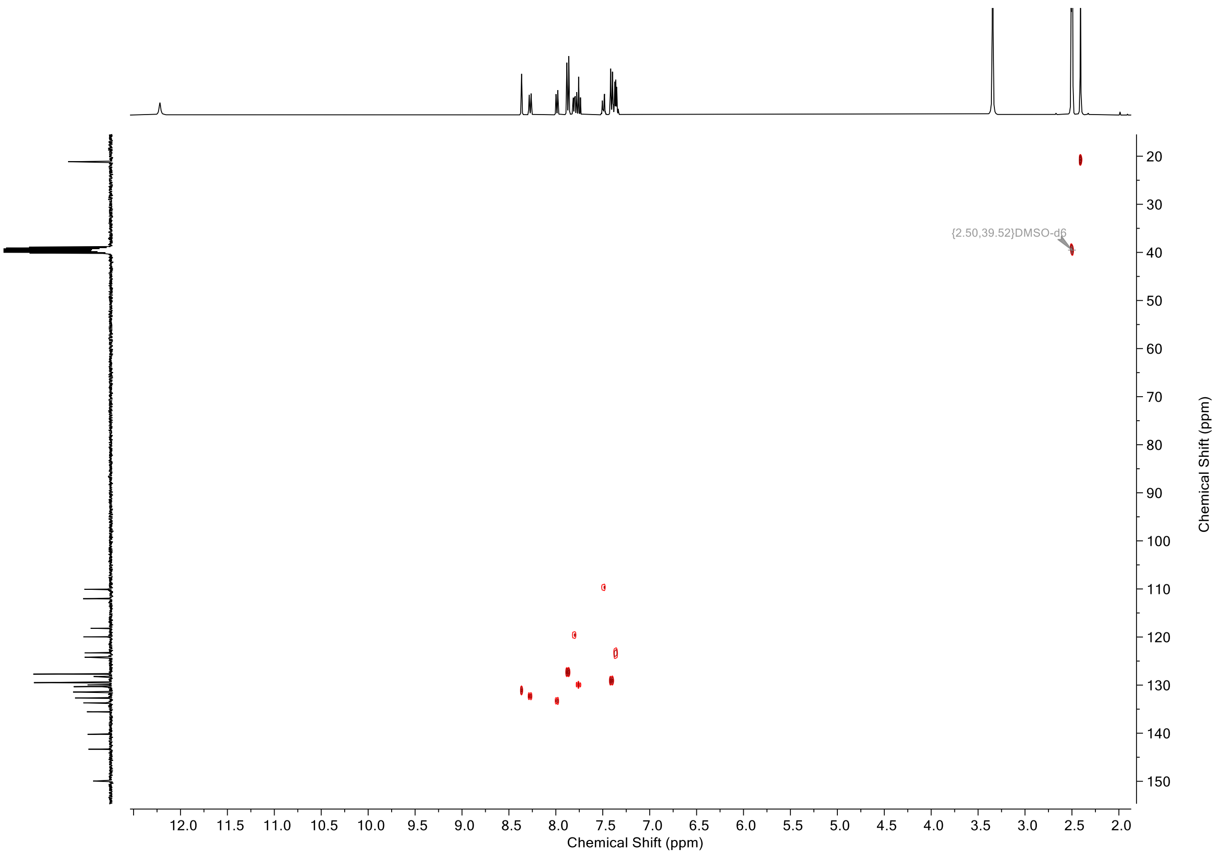

Figure S221: HSQC (DMSO-d_6): 1*H*-*N*-(2-(3-Cyanophenyl)-benzo[*d*]imidazol-1-yl)-4-methylbenz-amide (**3k**).

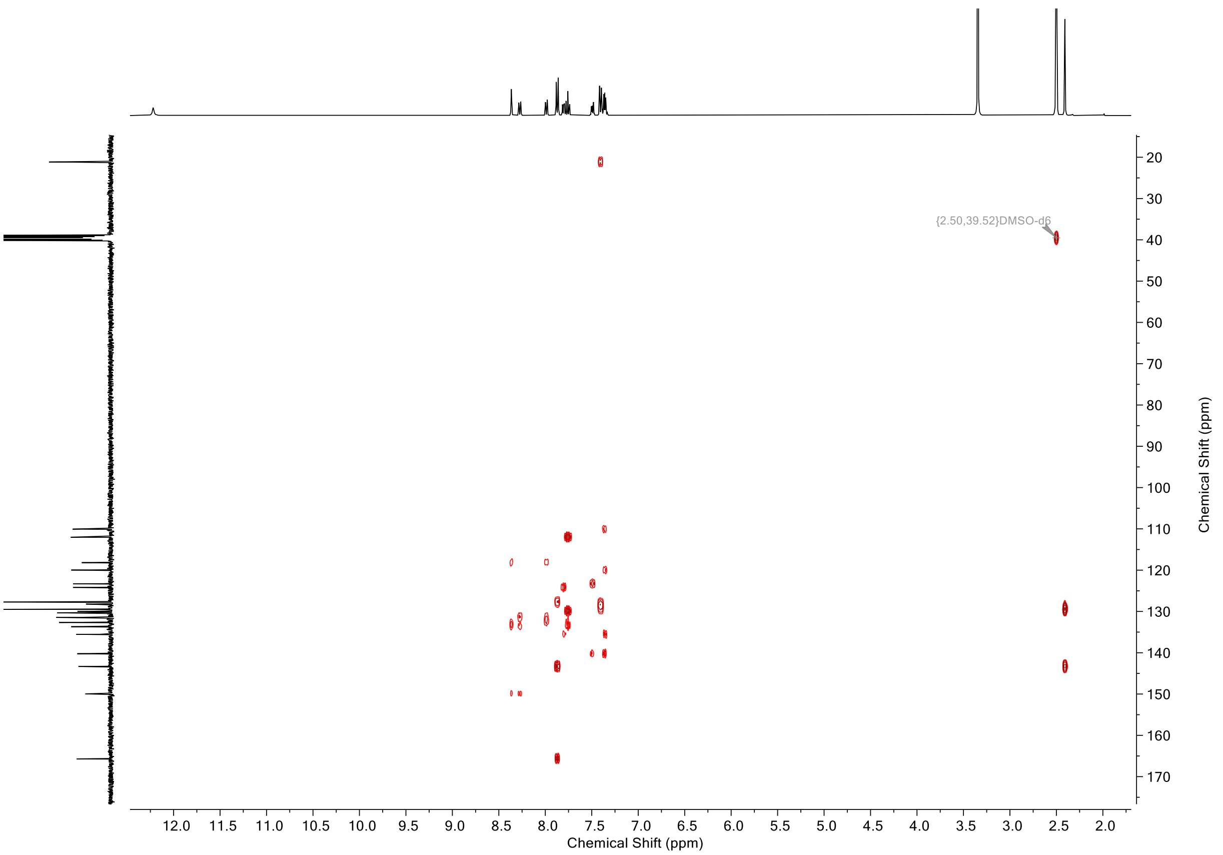

Figure S222: HMBC (DMSO-d_6): 1*H*-*N*-(2-(3-Cyanophenyl)-benzo[*d*]imidazol-1-yl)-4-methylbenz-amide (**3k**).

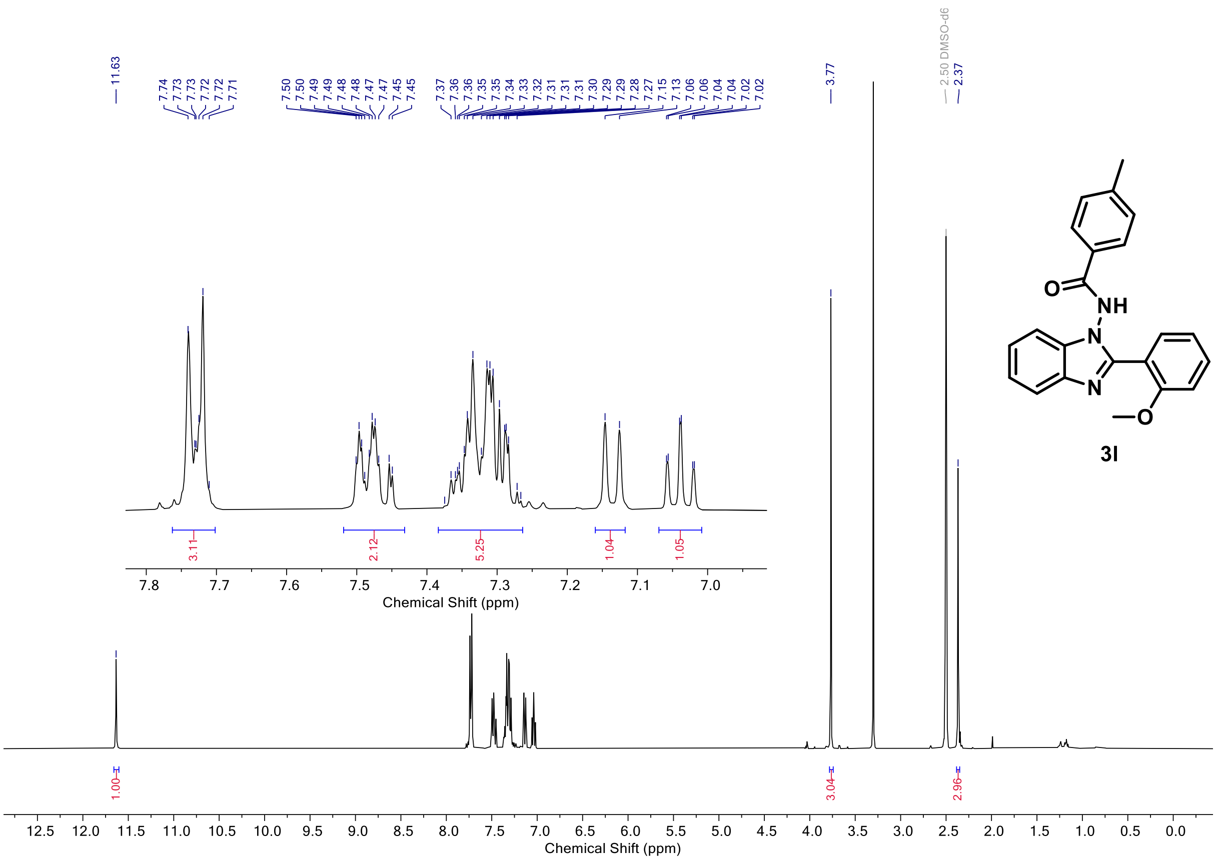

Figure S223: ^{1}H NMR (400 MHz, DMSO-d_6): 1*H*-*N*-(2-(2-Methoxyphenyl)-benzo[*d*]imidazol-1-yl)-4-methylbenz-amide (**3l**).

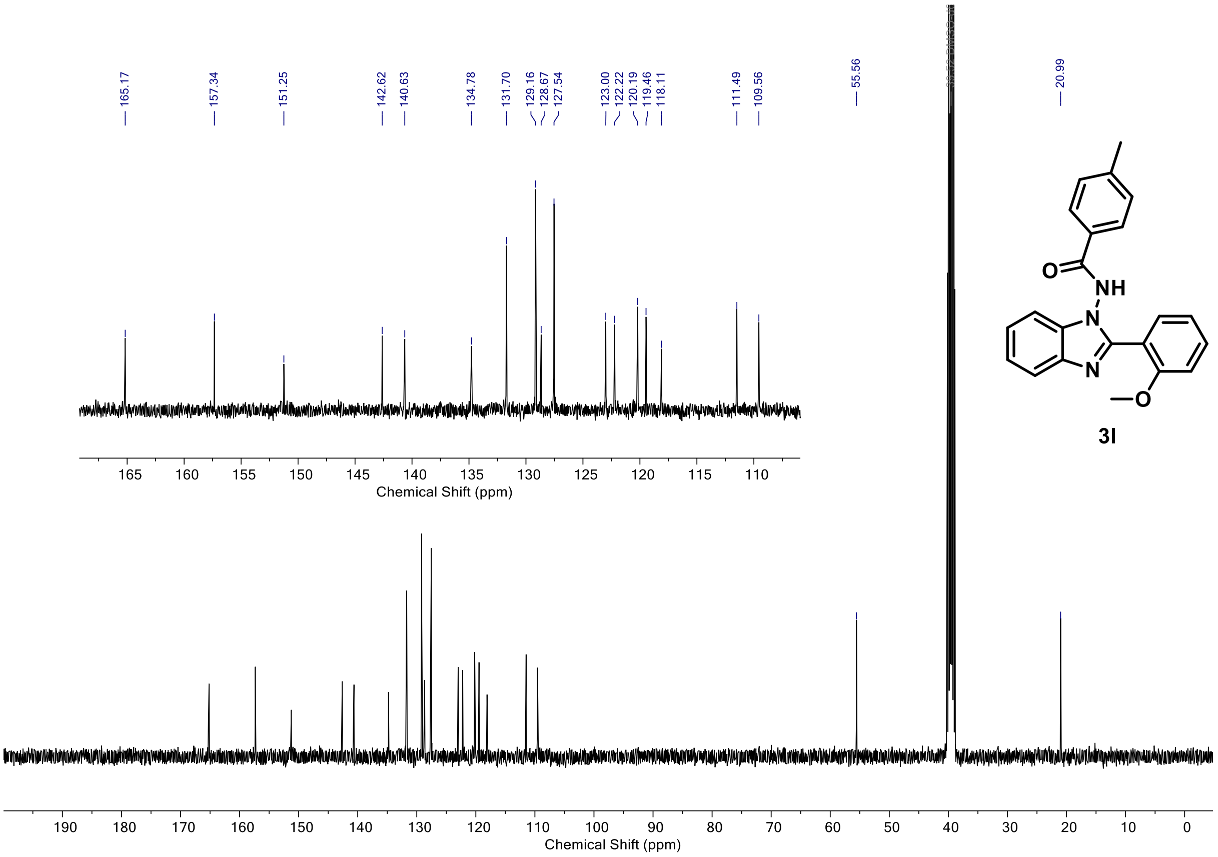

Figure S224: ^{13}C{^{1}H} NMR (101 MHz, DMSO-d_6): 1*H*-*N*-(2-(2-Methoxyphenyl)-benzo[*d*]imidazol-1-yl)-4-methylbenz-amide (**3l**).

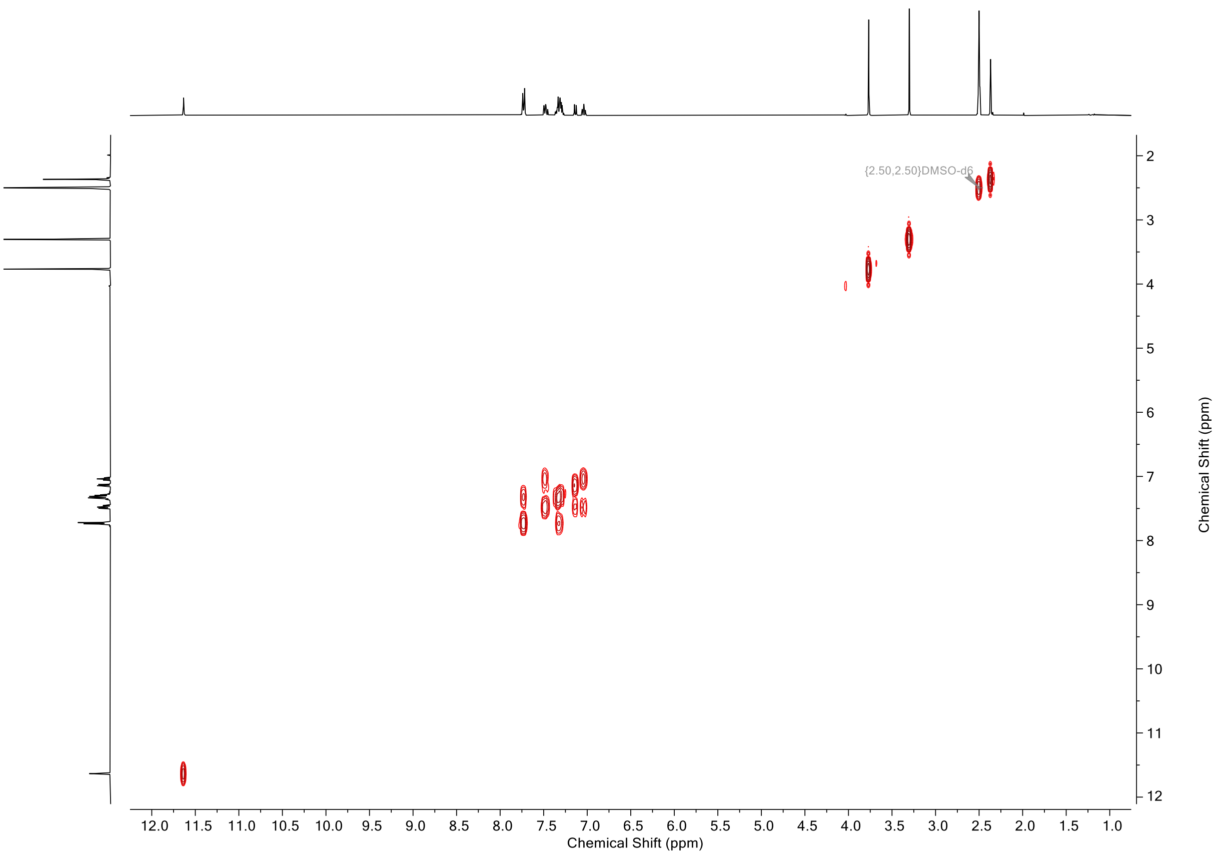

Figure S225: COSY (DMSO-d_6): 1*H*-*N*-(2-(2-Methoxyphenyl)-benzo[*d*]imidazol-1-yl)-4-methylbenz-amide (**3l**).

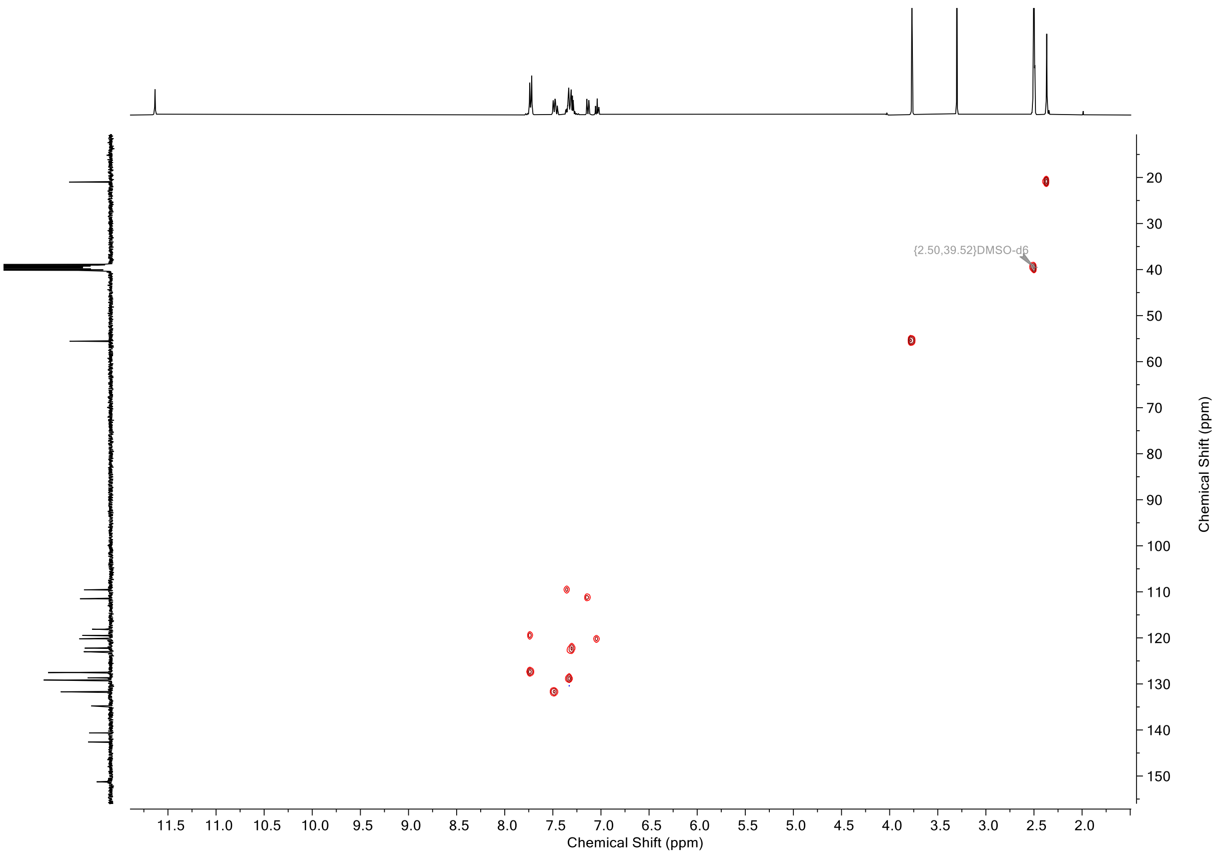

Figure S226: HSQC (DMSO-d_6): 1*H*-*N*-(2-(2-Methoxyphenyl)-benzo[*d*]imidazol-1-yl)-4-methylbenz-amide (**3l**).

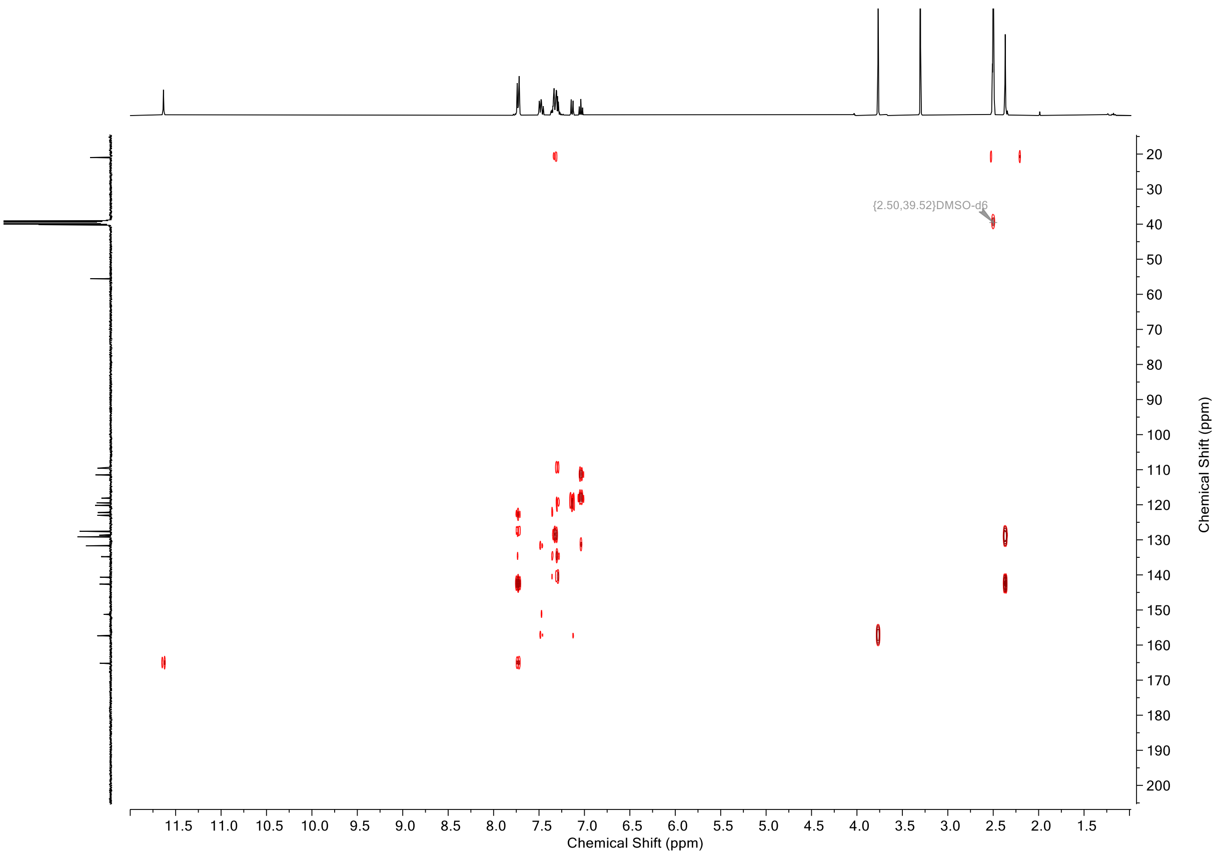

Figure S227: HMBC (DMSO-d_6): 1*H*-*N*-(2-(2-Methoxyphenyl)-benzo[*d*]imidazol-1-yl)-4-methylbenz-amide (**3l**).

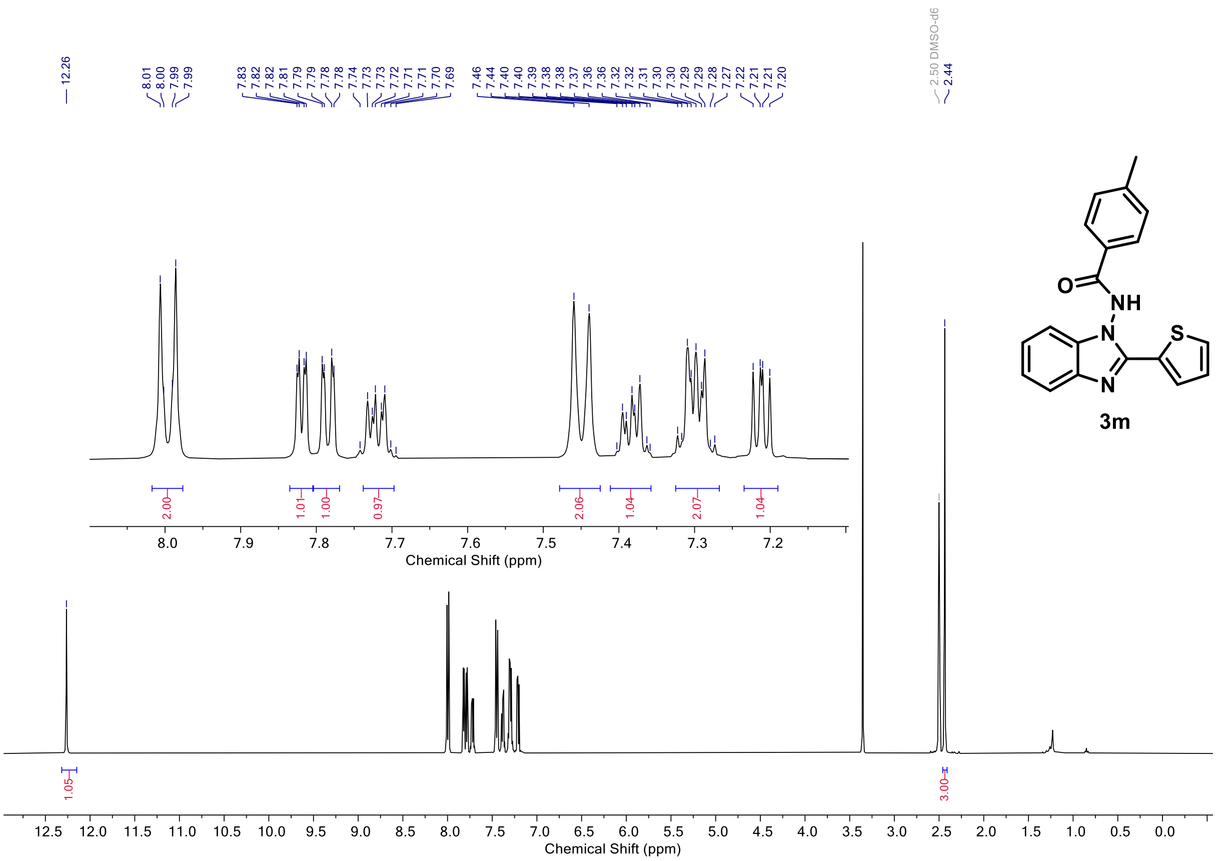

Figure S228: ^{1}H NMR (400 MHz, DMSO-d_6): 1*H*-4-Methyl-*N*-(2-(thien-2-yl)- benzo[*d*]imidazol-1-yl)benzamide (**3m**).

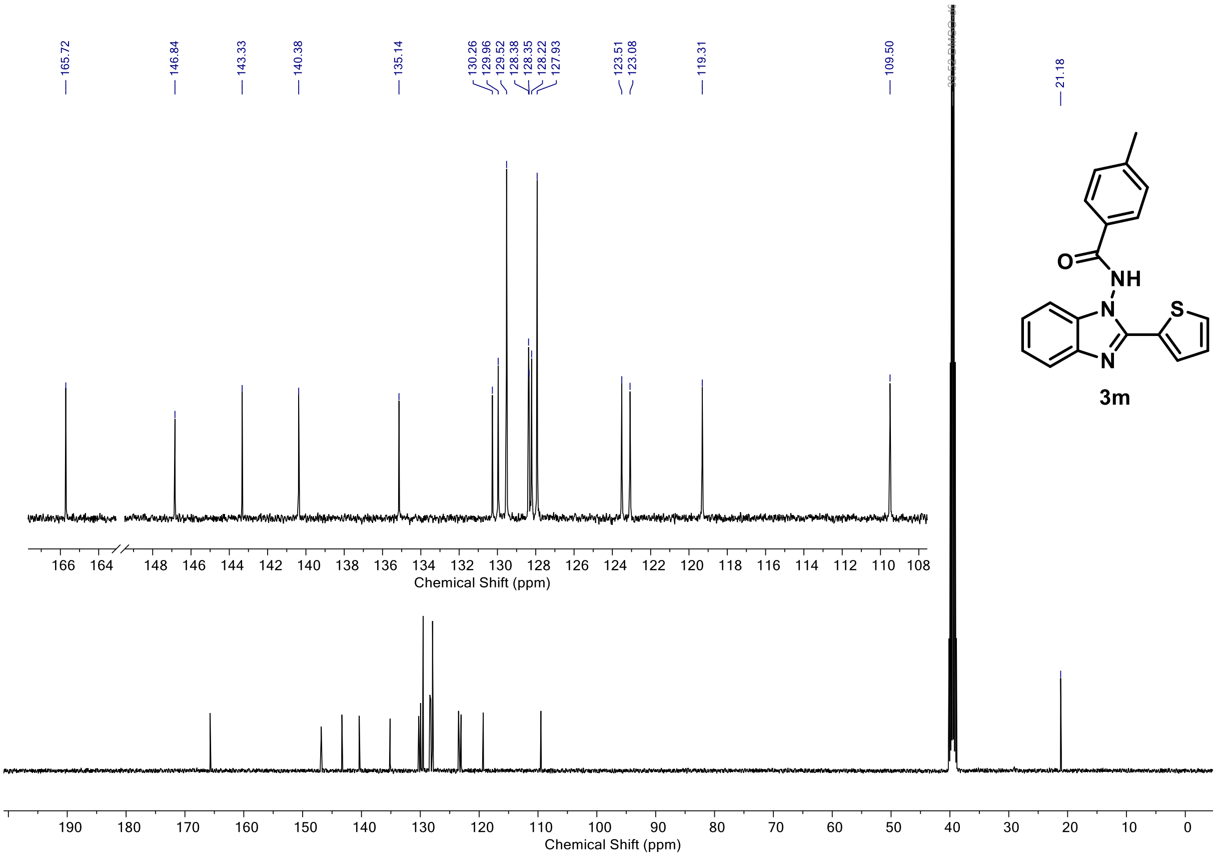

Figure S229: ^{13}C{^{1}H} NMR (101 MHz, DMSO-d_6): 1*H*-4-Methyl-*N*-(2-(thien-2-yl)-benzo[*d*]imidazol-1-yl)benz-amide (**3m**).

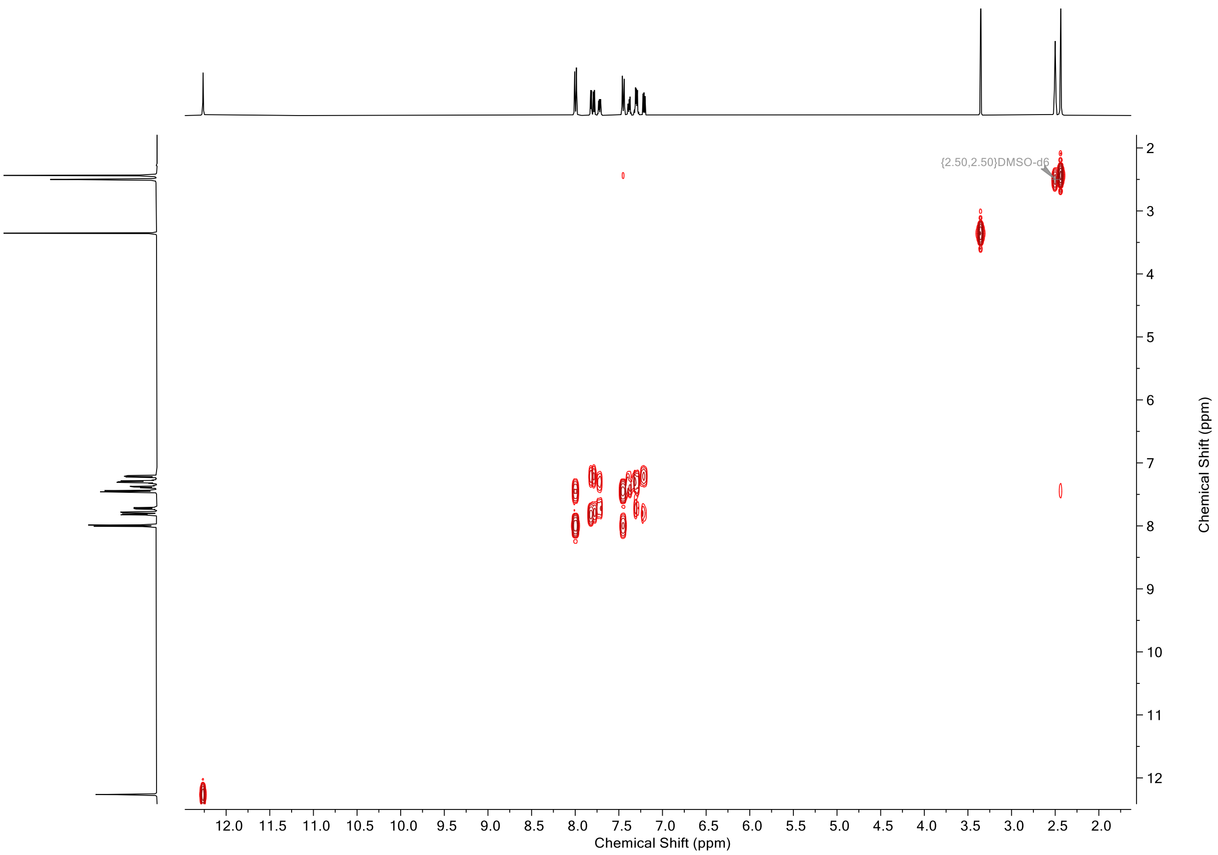

Figure S230: COSY (DMSO-d_6): 1*H*-4-Methyl-*N*-(2-(thien-2-yl)-benzo[*d*]imidazol-1-yl)benz-amide (**3m**).

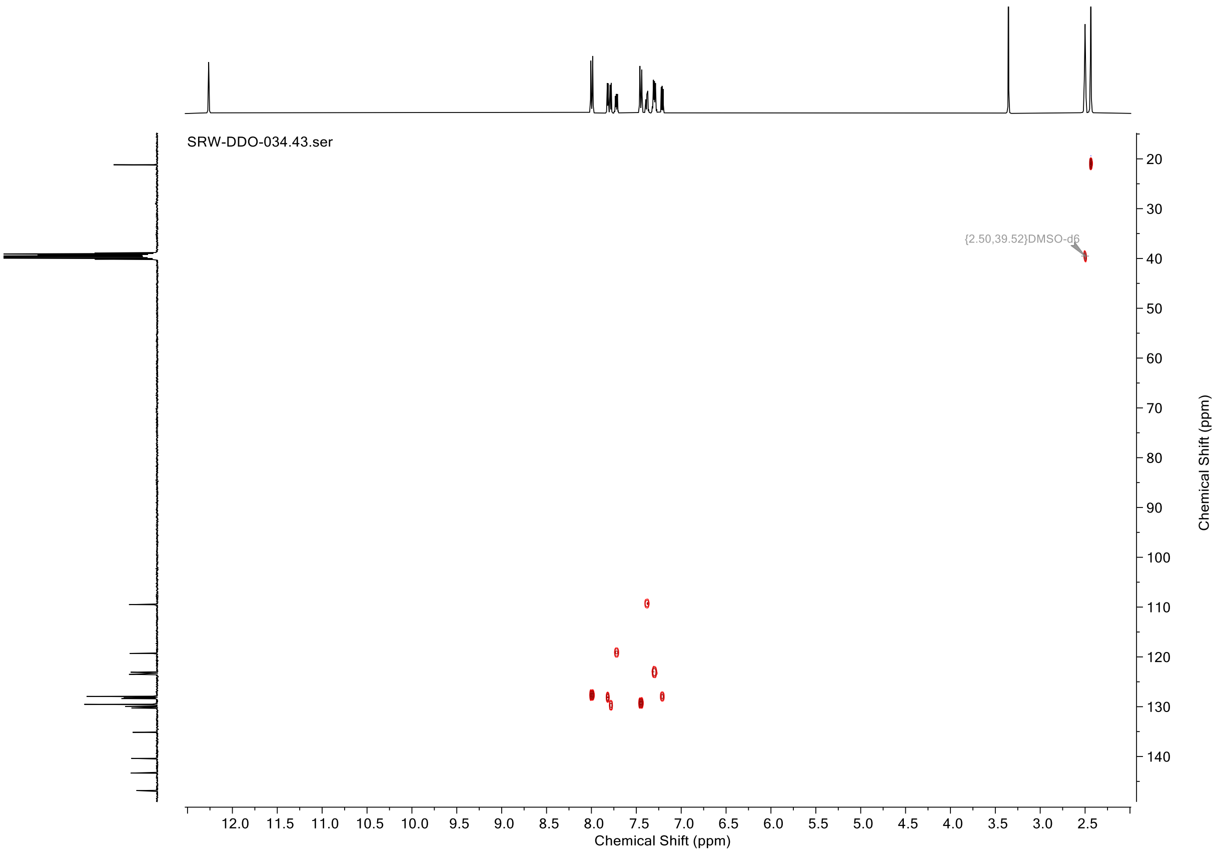

Figure S231: HSQC (DMSO-d_6): 1*H*-4-Methyl-*N*-(2-(thien-2-yl)-benzo[*d*]imidazol-1-yl)benz-amide (**3m**).

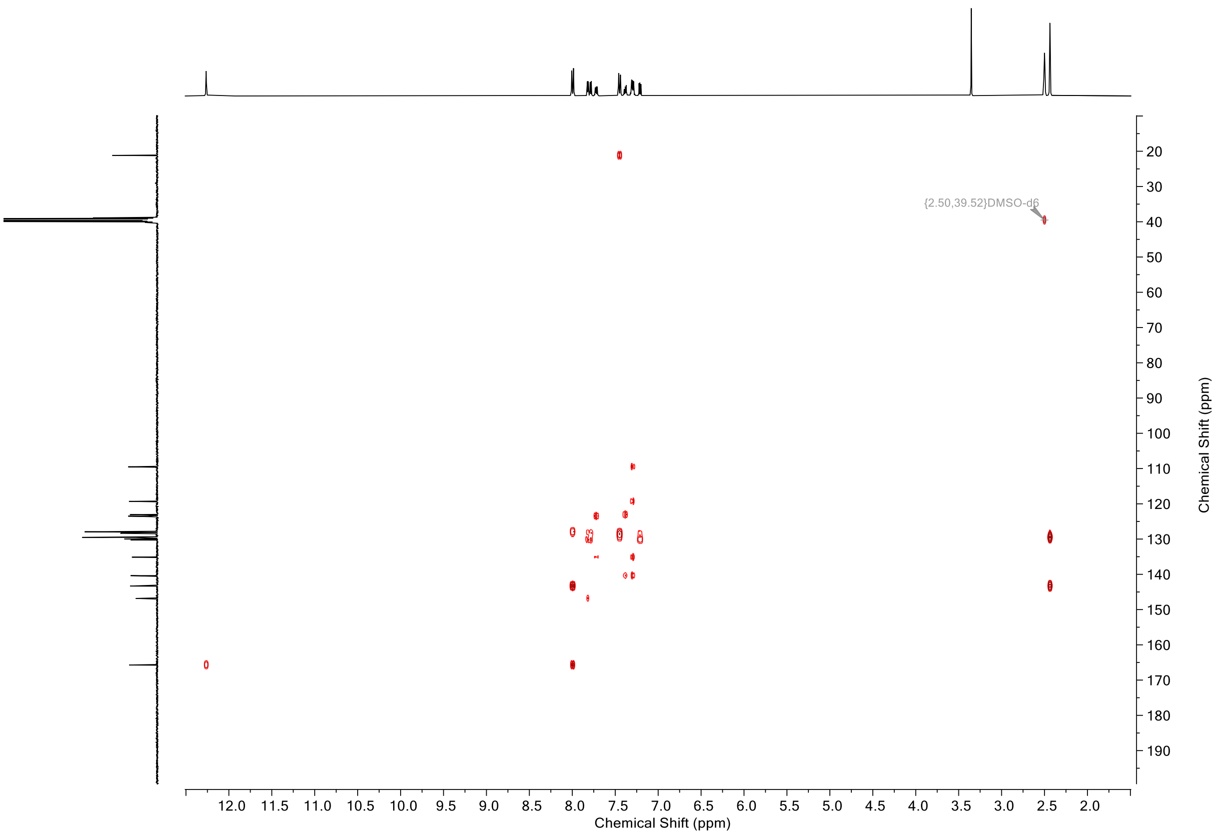

Figure S232: HMBC (DMSO-d_6): 1*H*-4-Methyl-*N*-(2-(thien-2-yl)-benzo[*d*]imidazol-1-yl)benz-amide (**3m**).

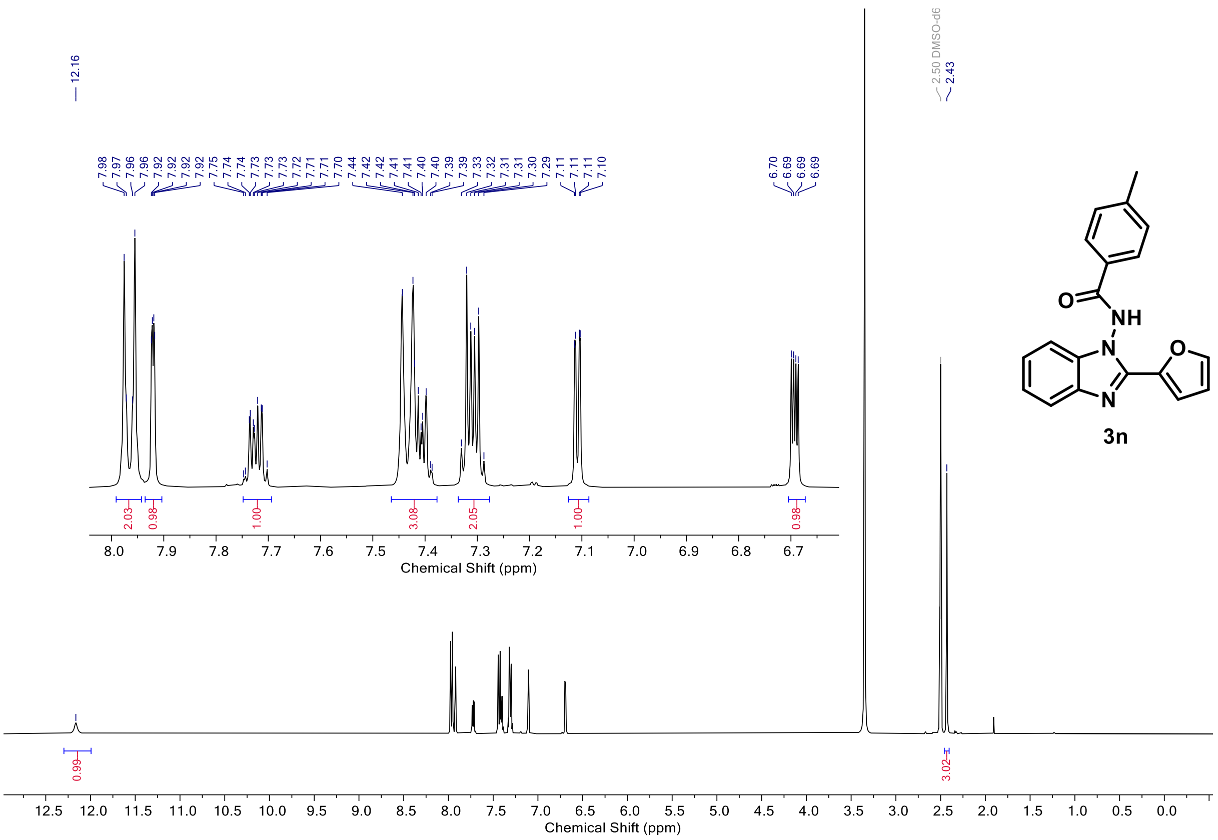

Figure S233: ^{1}H NMR (400 MHz, DMSO-d_6): 1*H*-*N*-(2-(Furan-2-yl)-benzo[*d*]imidazol-1-yl)benzamide (**3n**).

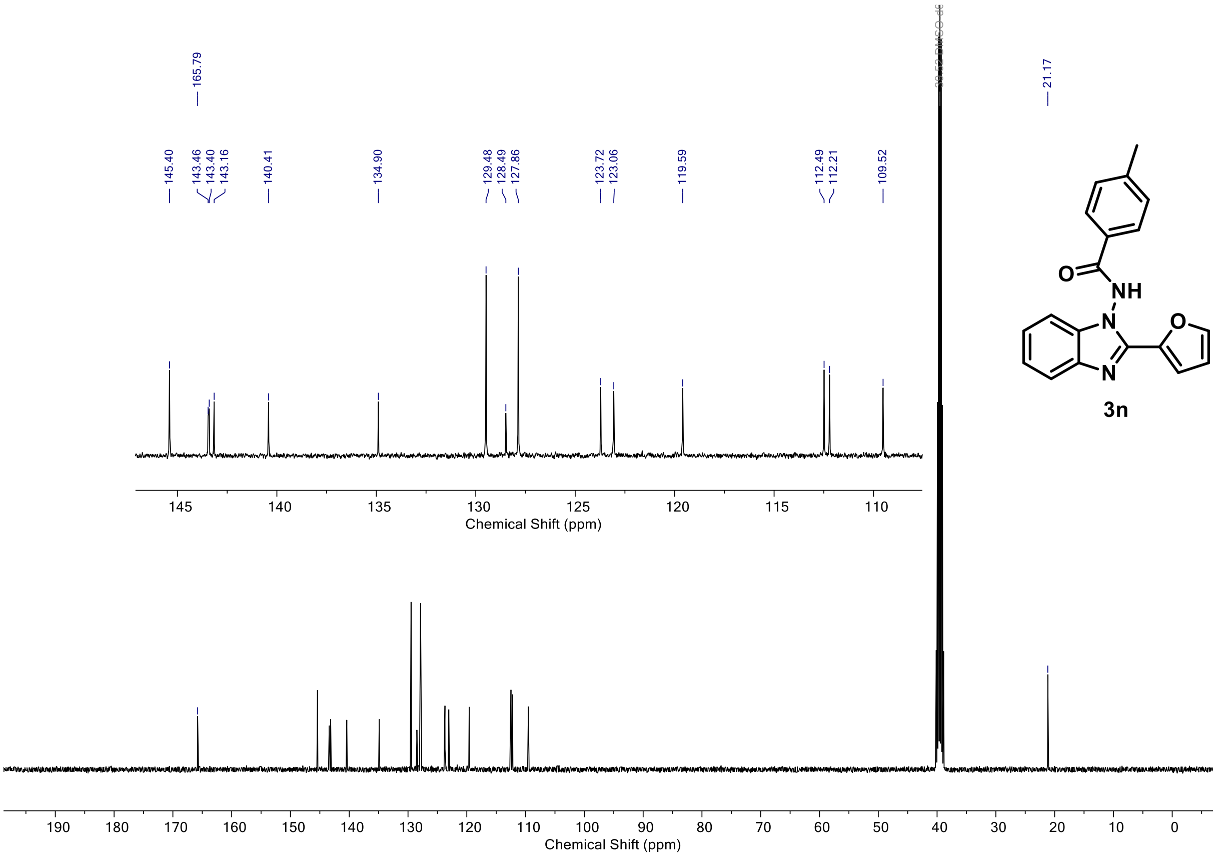

Figure S234: ^{13}C{^{1}H} NMR (101 MHz, DMSO-d_6): 1*H*-*N*-(2-(Furan-2-yl)-benzo[*d*]imidazol-1-yl)benzamide (**3n**).

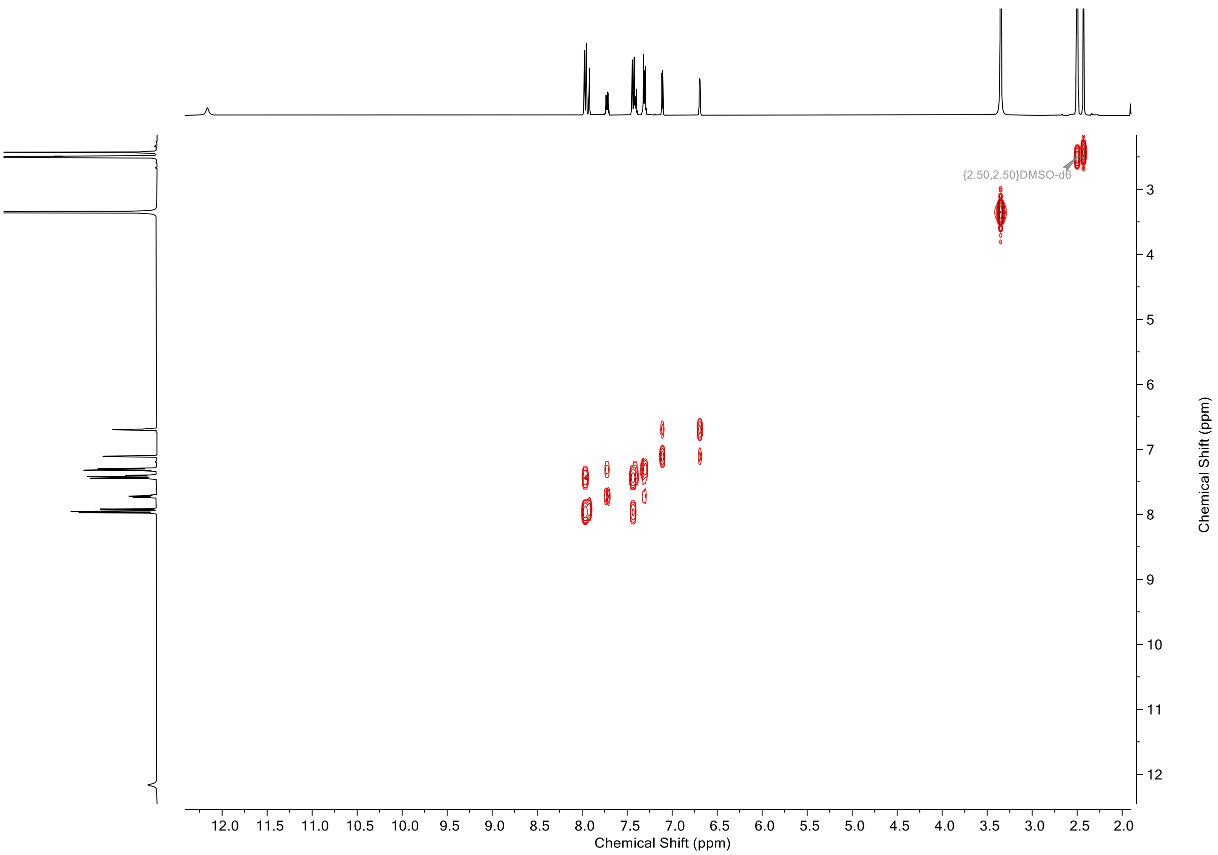

Figure S235: COSY (DMSO-d_6): 1*H*-*N*-(2-(Furan-2-yl)-benzo[*d*]imidazol-1-yl)benzamide (**3n**).

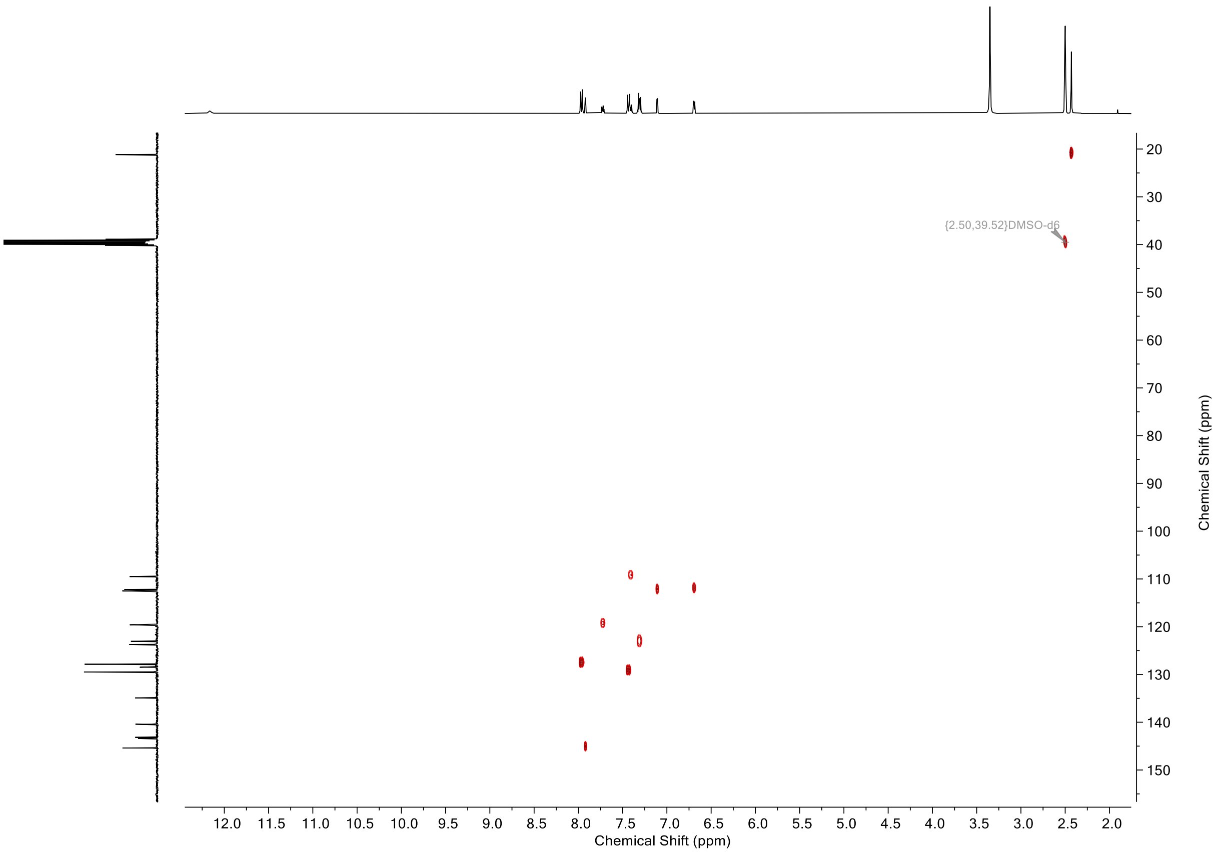

Figure S236: COSY (DMSO-d_6): 1*H*-*N*-(2-(Furan-2-yl)-benzo[*d*]imidazol-1-yl)benzamide (**3n**).

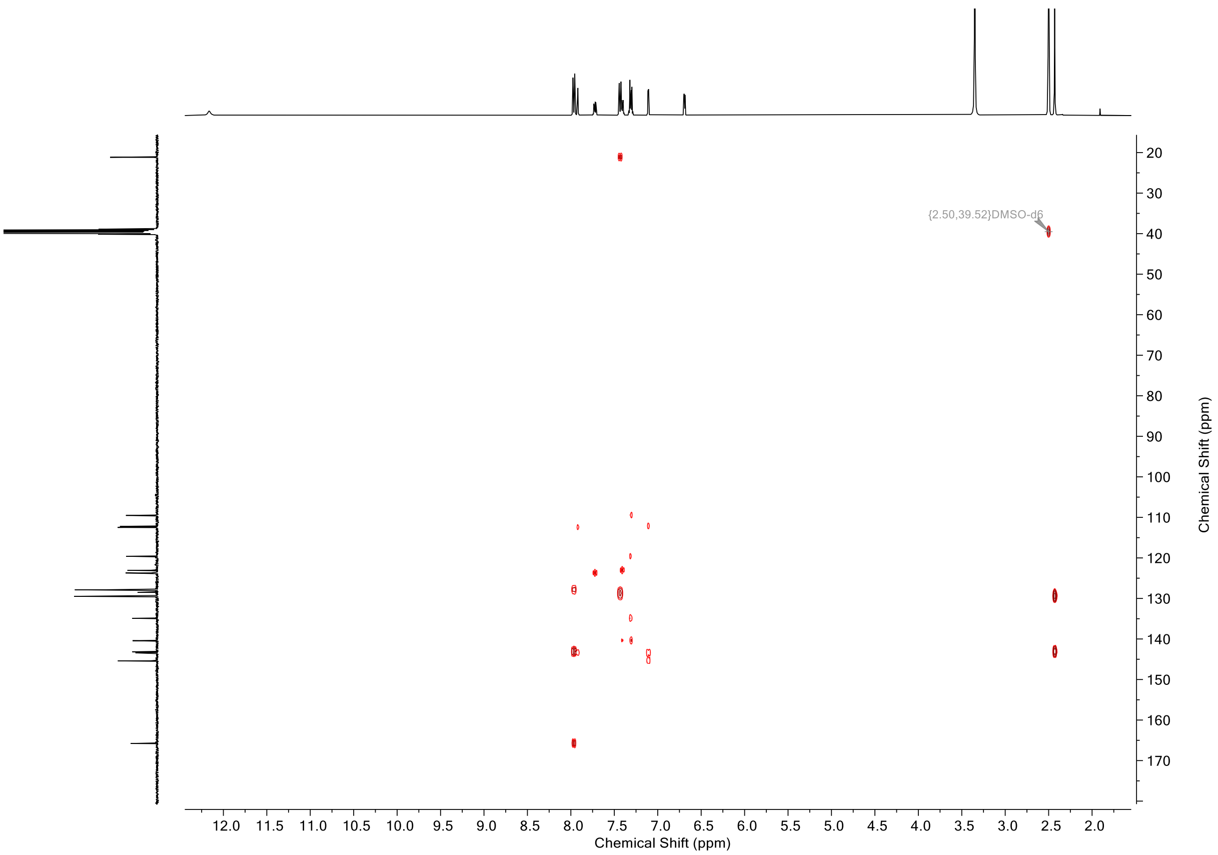

Figure S237: COSY (DMSO-d_6): 1*H*-*N*-(2-(Furan-2-yl)-benzo[*d*]imidazol-1-yl)benzamide (**3n**).

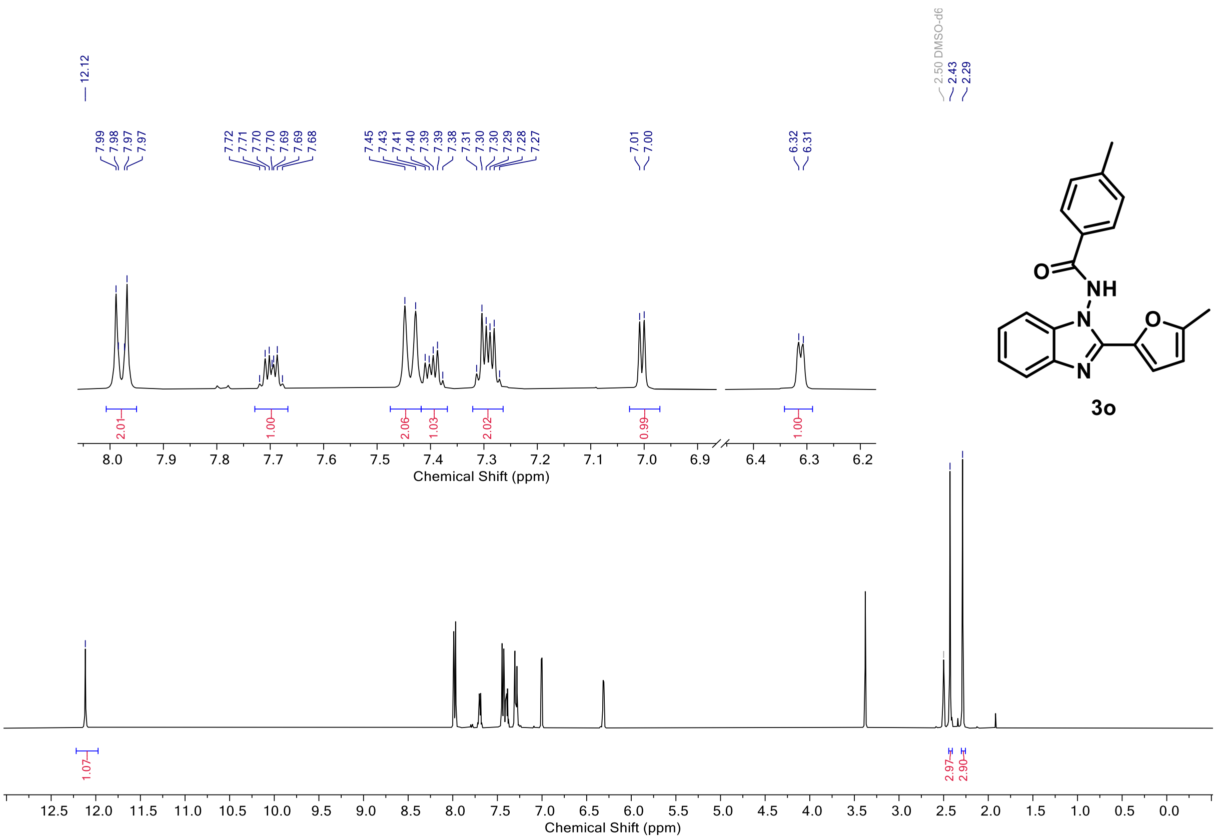

Figure S238: ^{1}H NMR (400 MHz, DMSO-d_6): 1*H*-4-Methyl-*N*-(2-(5-methylfuran-2-yl)-benzo[*d*]imidazol-1-yl)benz-amide (**3o**).

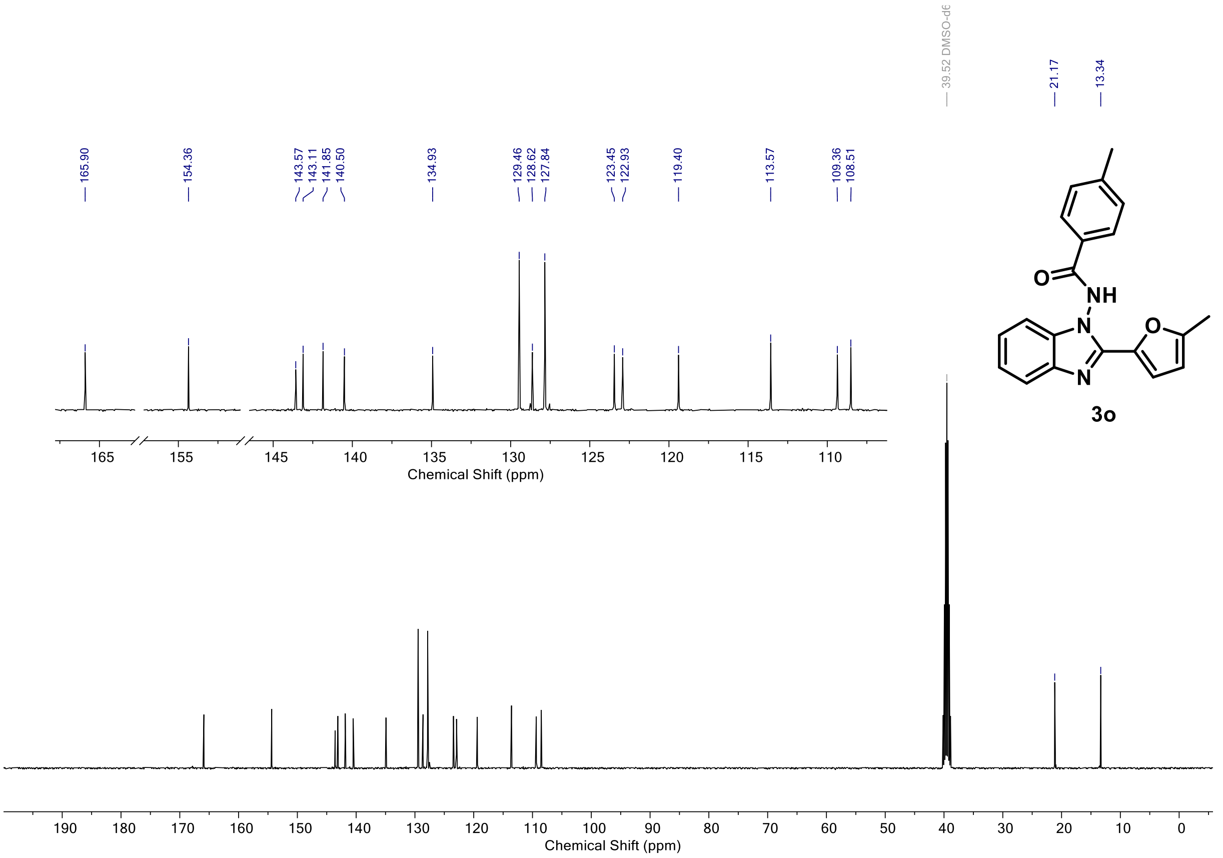

Figure S239: $^{13}C\{^{1}H\}$ NMR (101 MHz, DMSO-d_6): 1*H*-4-Methyl-*N*-(2-(5-methylfuran-2-yl)-benzo[*d*]imid-azol-1-yl)benz-amide (**3o**).

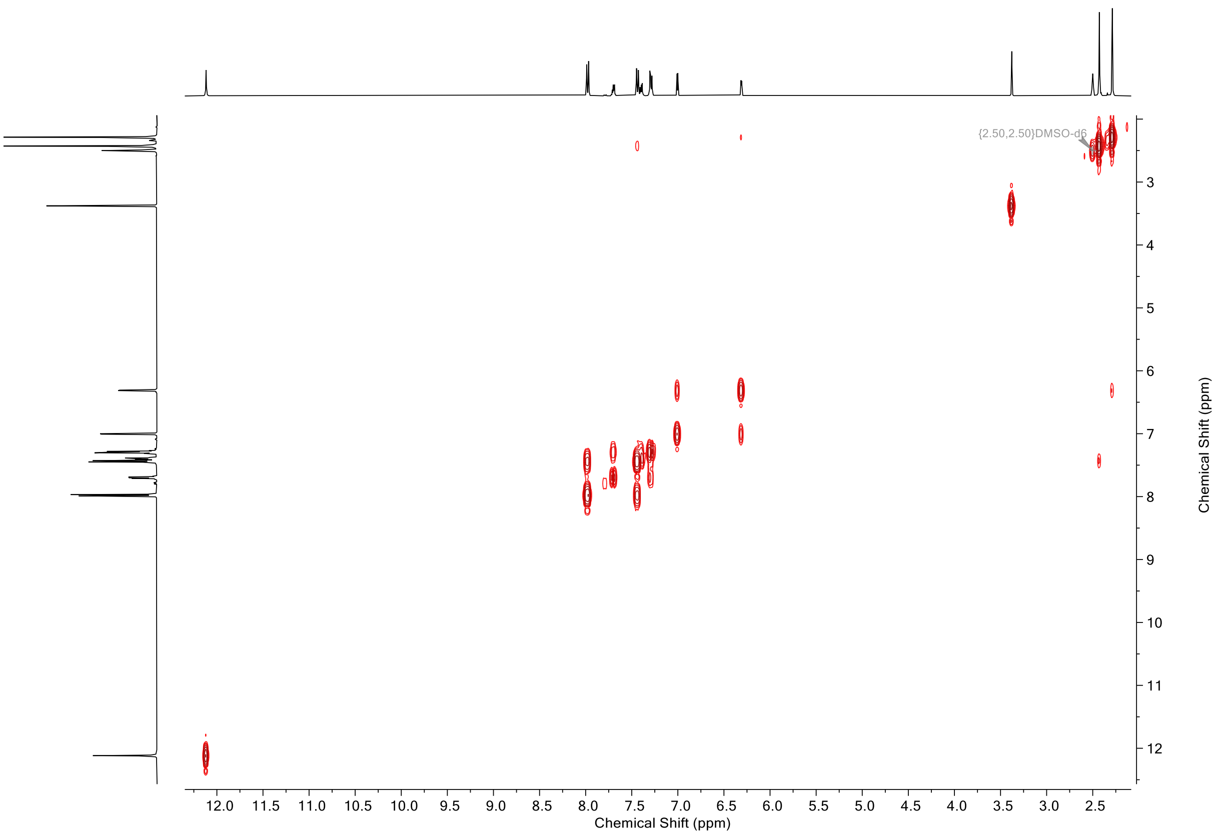

Figure S240: COSY (DMSO-d_6): 1*H*-4-Methyl-*N*-(2-(5-methylfuran-2-yl)-benzo[*d*]imidazol-1-yl)benzamide (**3o**).

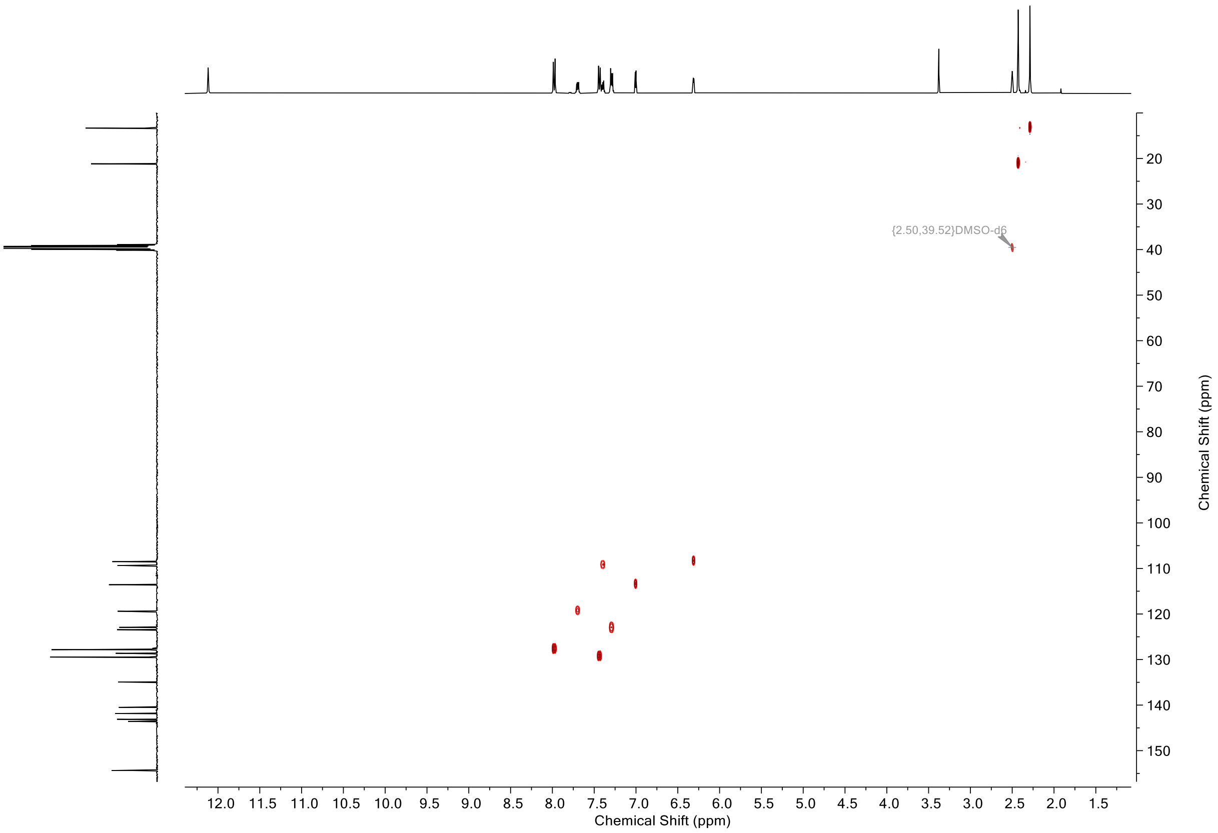

Figure S241: HSQC (DMSO-d_6): 1*H*-4-Methyl-*N*-(2-(5-methylfuran-2-yl)-benzo[*d*]imidazol-1-yl)benzamide (**3o**).

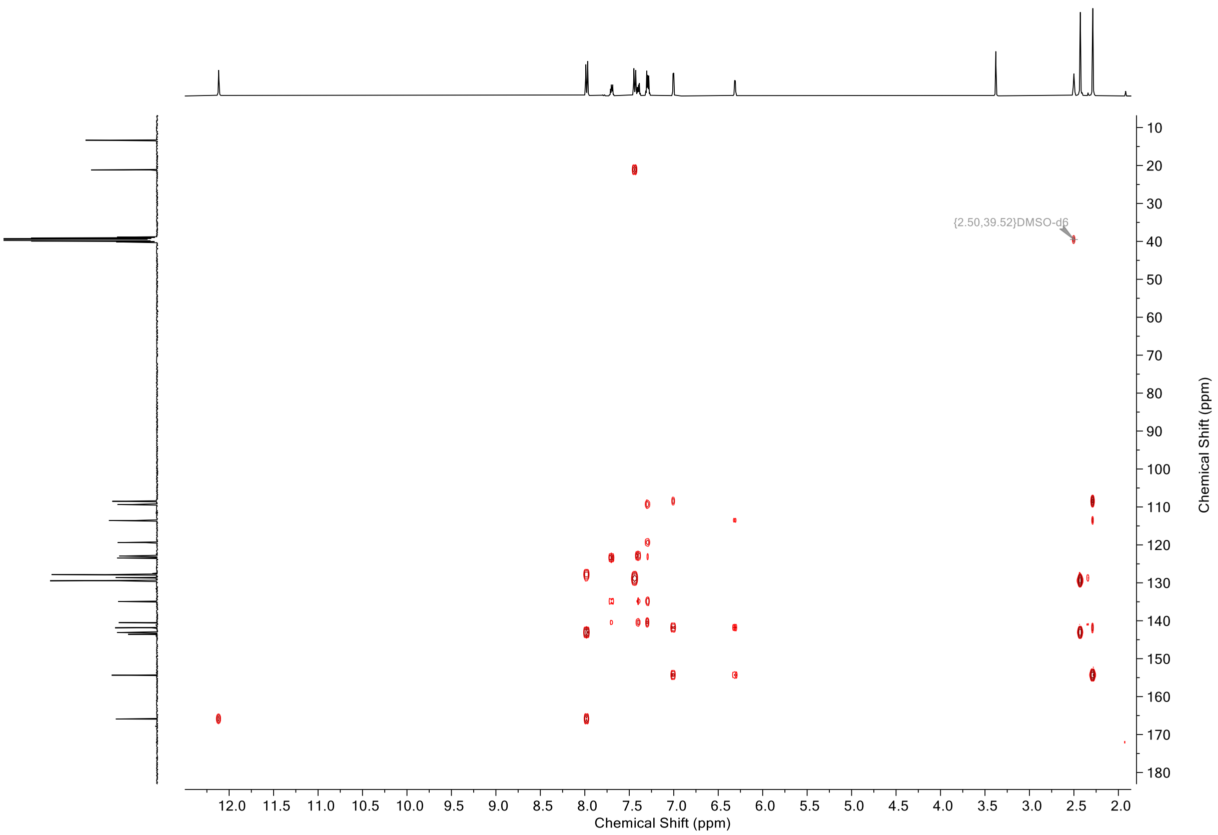

Figure S242: HMBC (DMSO-d_6): 1H-4-Methyl-N-(2-(5-methylfuran-2-yl)-benzo[d]imidazol-1-yl)benzamide (**3o**).

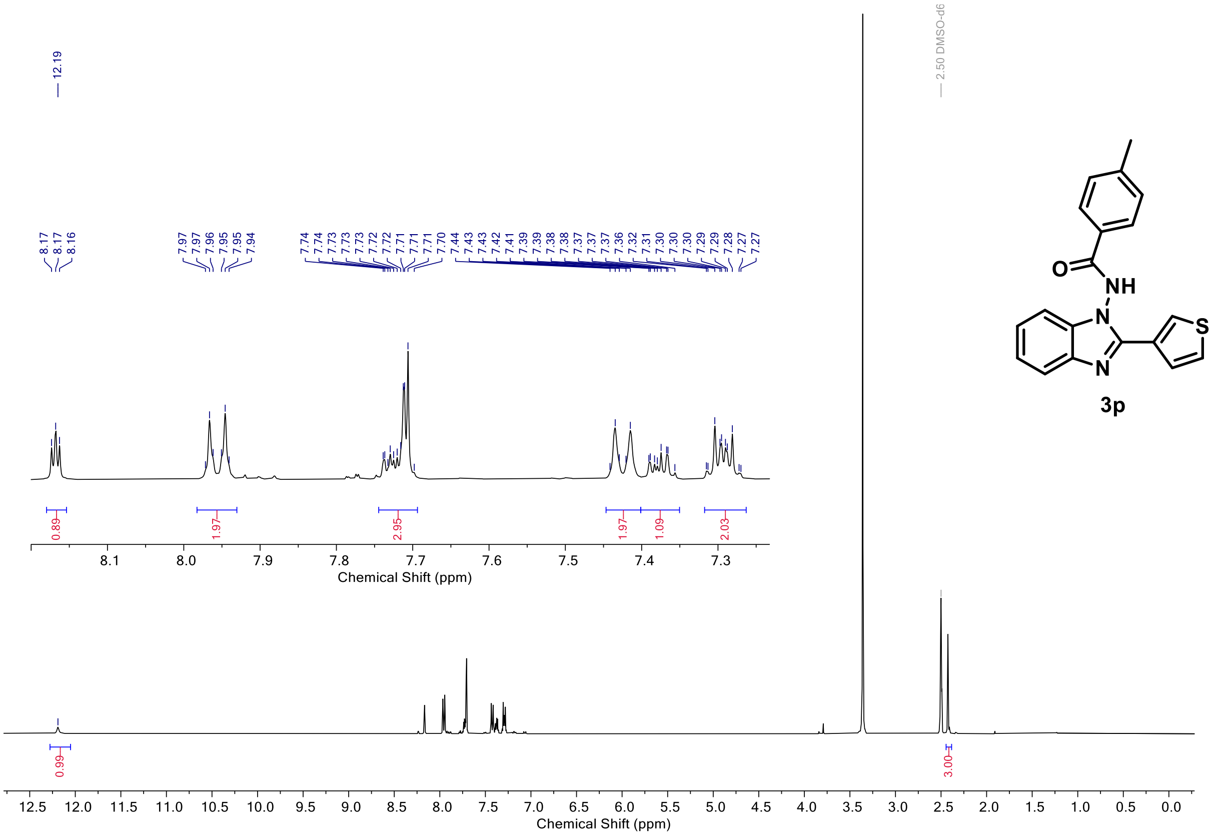

Figure S243: ^{1}H NMR (400 MHz, DMSO-d_6): 1*H*-4-Methyl-*N*-(2-(thien-3-yl)-benzo[*d*]imidazol-1-yl)benzamide (**3p**).

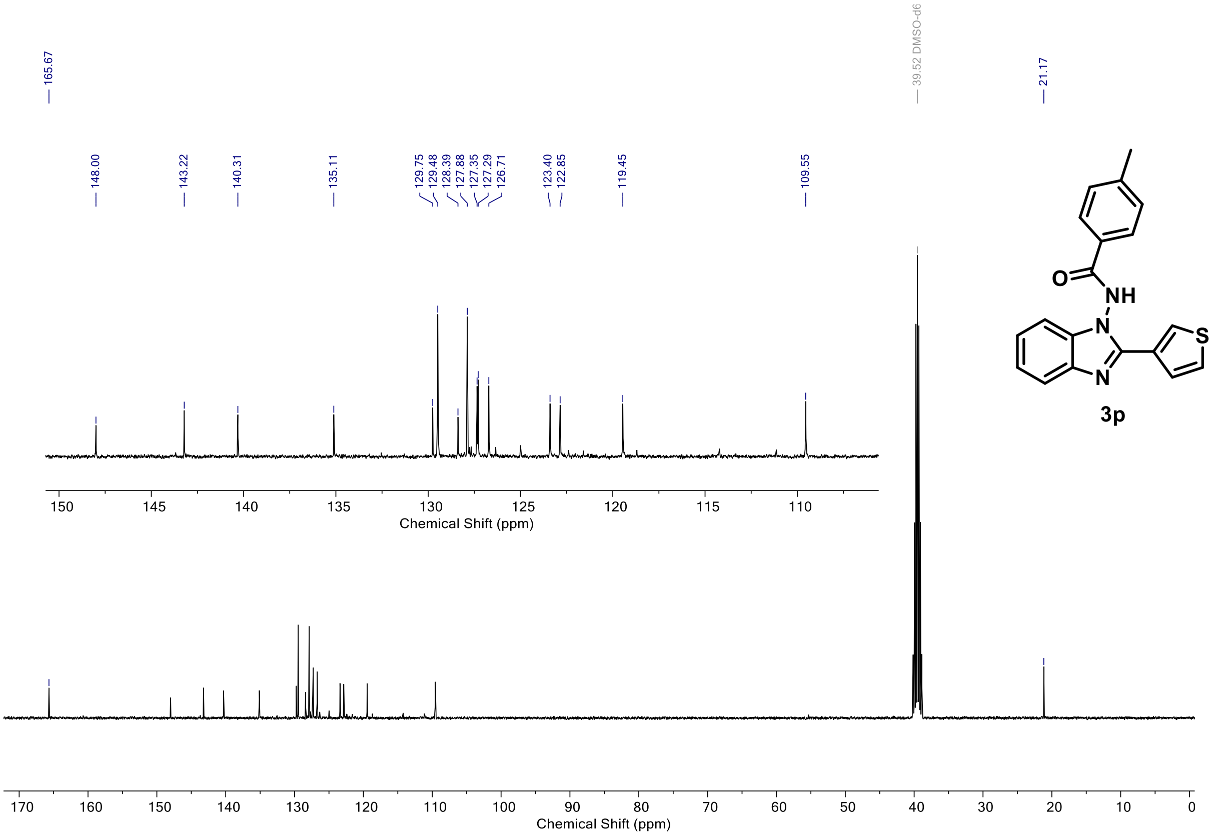

Figure S244: ^{13}C{^{1}H} NMR (101 MHz, DMSO-d_6): 1*H*-4-Methyl-*N*-(2-(thien-3-yl)-benzo[*d*]imidazol-1-yl)benz-amide (**3p**).

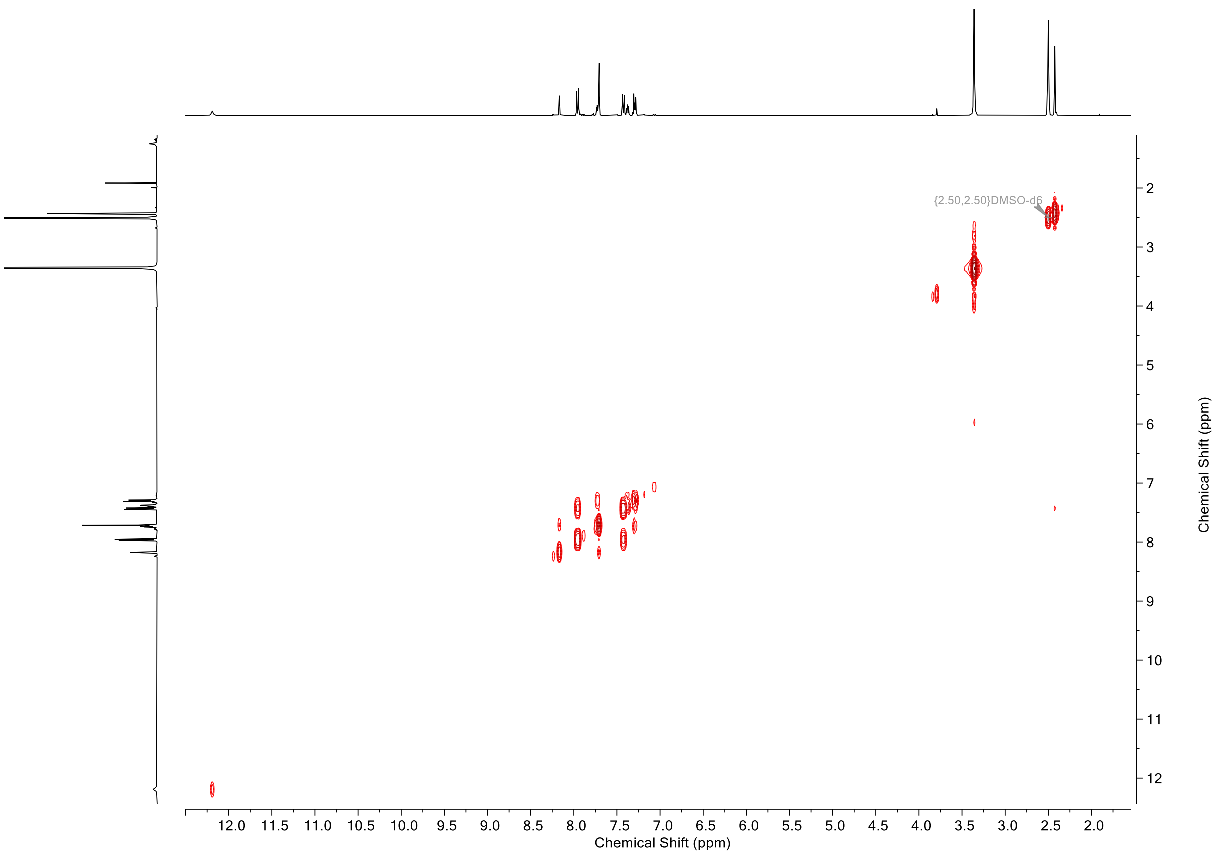

Figure S245: COSY (DMSO-d_6): 1*H*-4-Methyl-*N*-(2-(thien-3-yl)-benzo[*d*]imidazol-1-yl)benz-amide (**3p**).

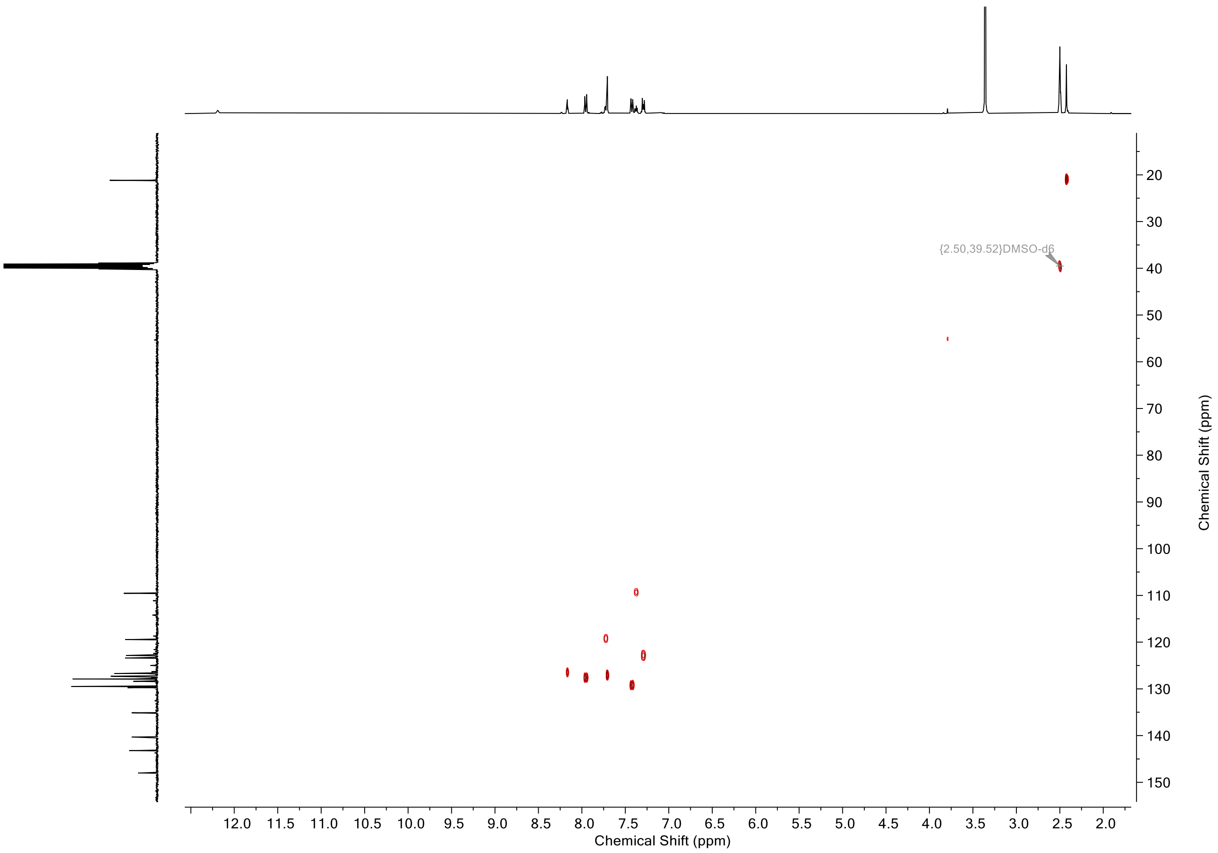

Figure S246: HSQC (DMSO-d_6): 1*H*-4-Methyl-*N*-(2-(thien-3-yl)-benzo[*d*]imidazol-1-yl)benz-amide (**3p**).

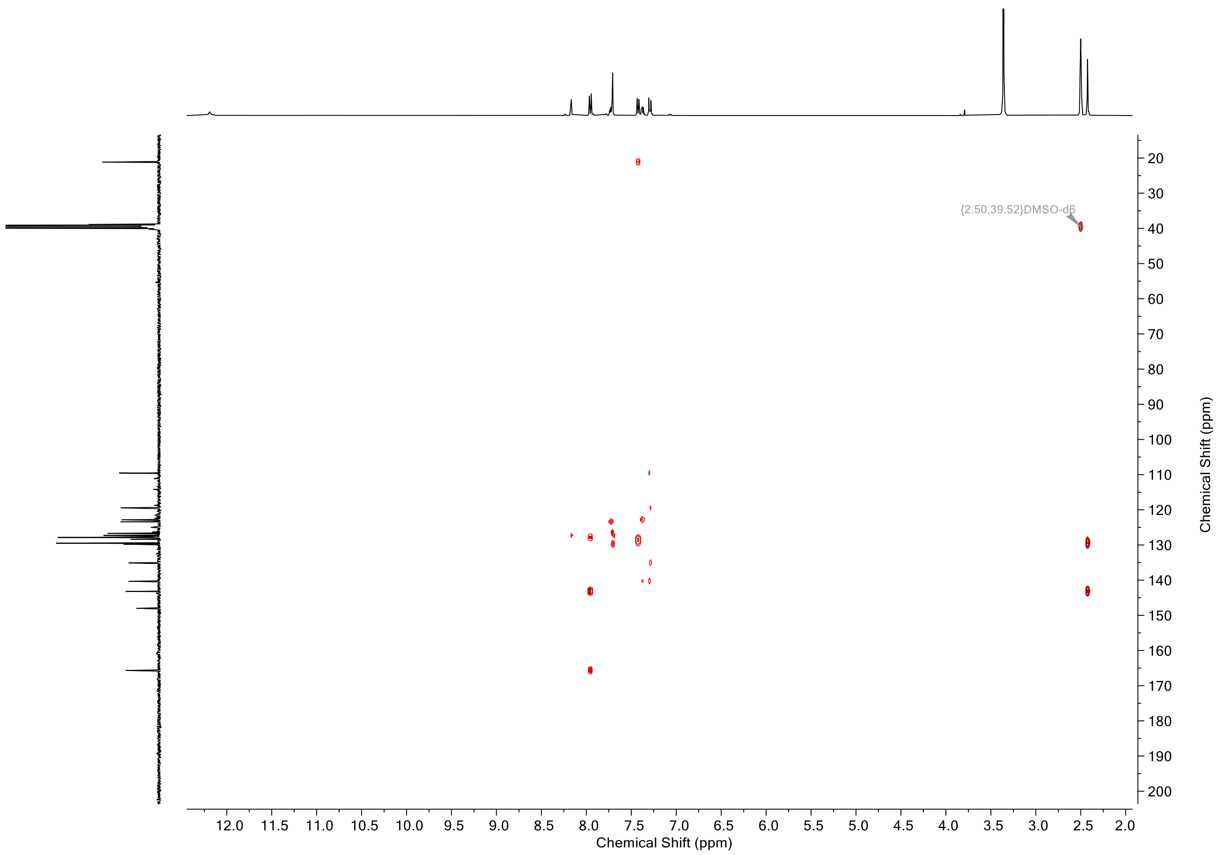

Figure S247: HMBC (DMSO-d_6): 1*H*-4-Methyl-*N*-(2-(thien-3-yl)-benzo[*d*]imidazol-1-yl)benz-amide (**3p**).

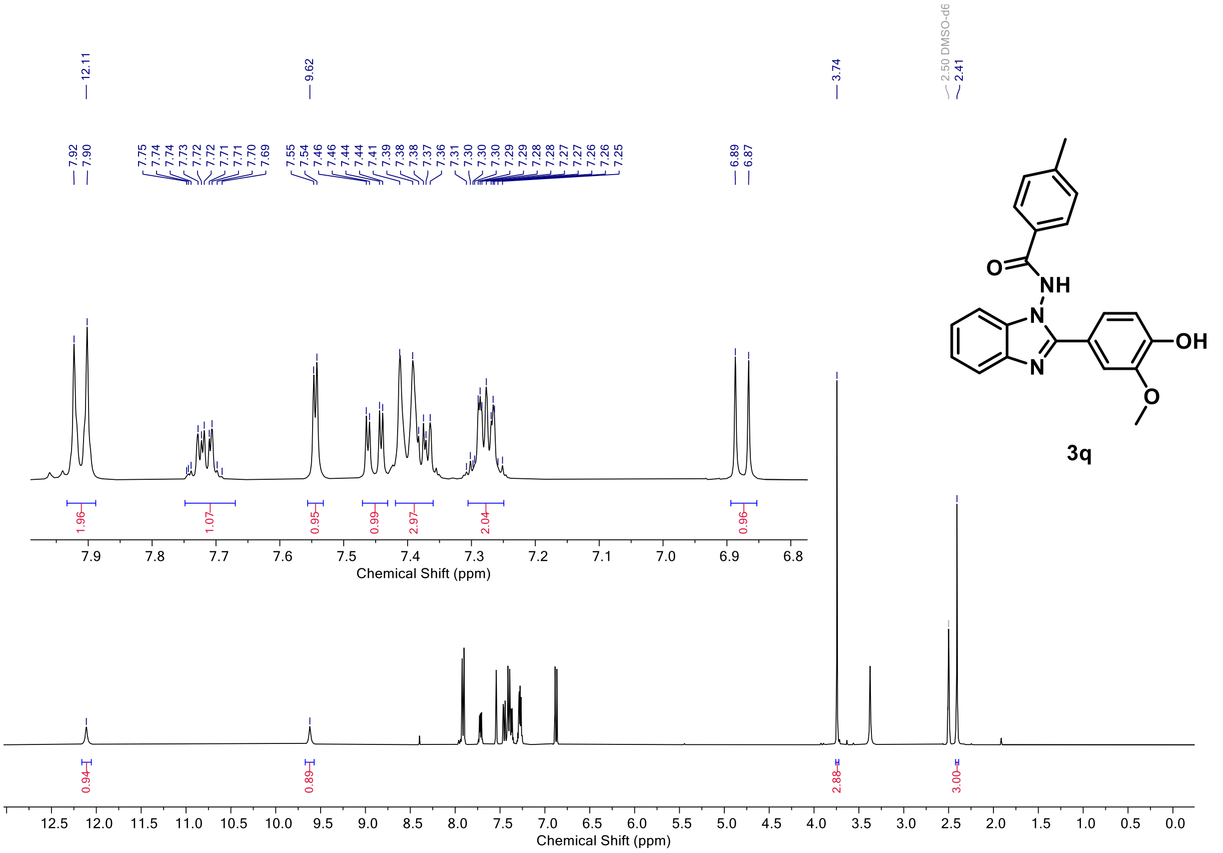

Figure S248: ^{1}H NMR (400 MHz, DMSO-d_6): 1*H*-*N*-(2-(4-Hydroxy-3-methoxyphenyl)-benzo[*d*]imidazol-1-yl)-4-methylbenzamide (**3q**).

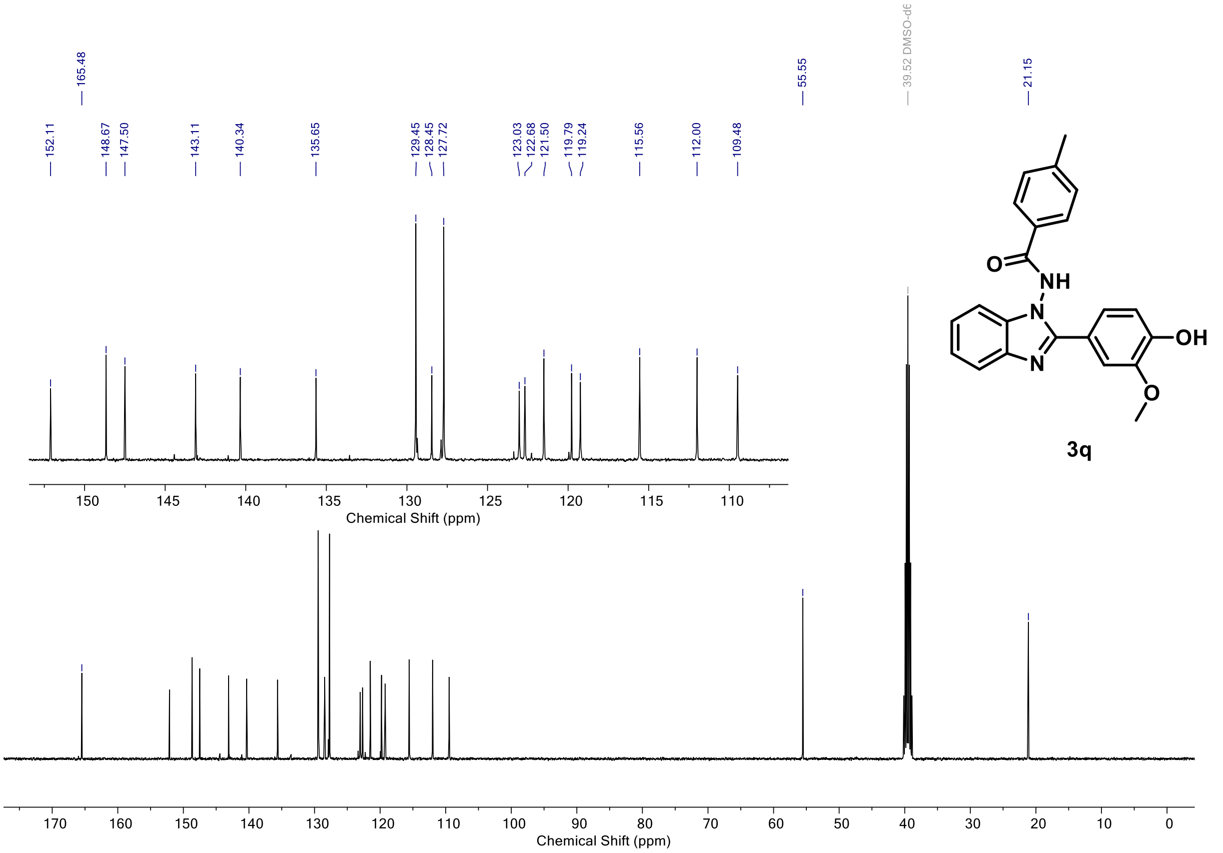

Figure S249: ^{13}C{^{1}H} NMR (101 MHz, DMSO-d_6): 1*H*-*N*-(2-(4-Hydroxy-3-methoxyphenyl)-benzo[*d*]imidazol-1-yl)-4-methylbenzamide (**3q**).

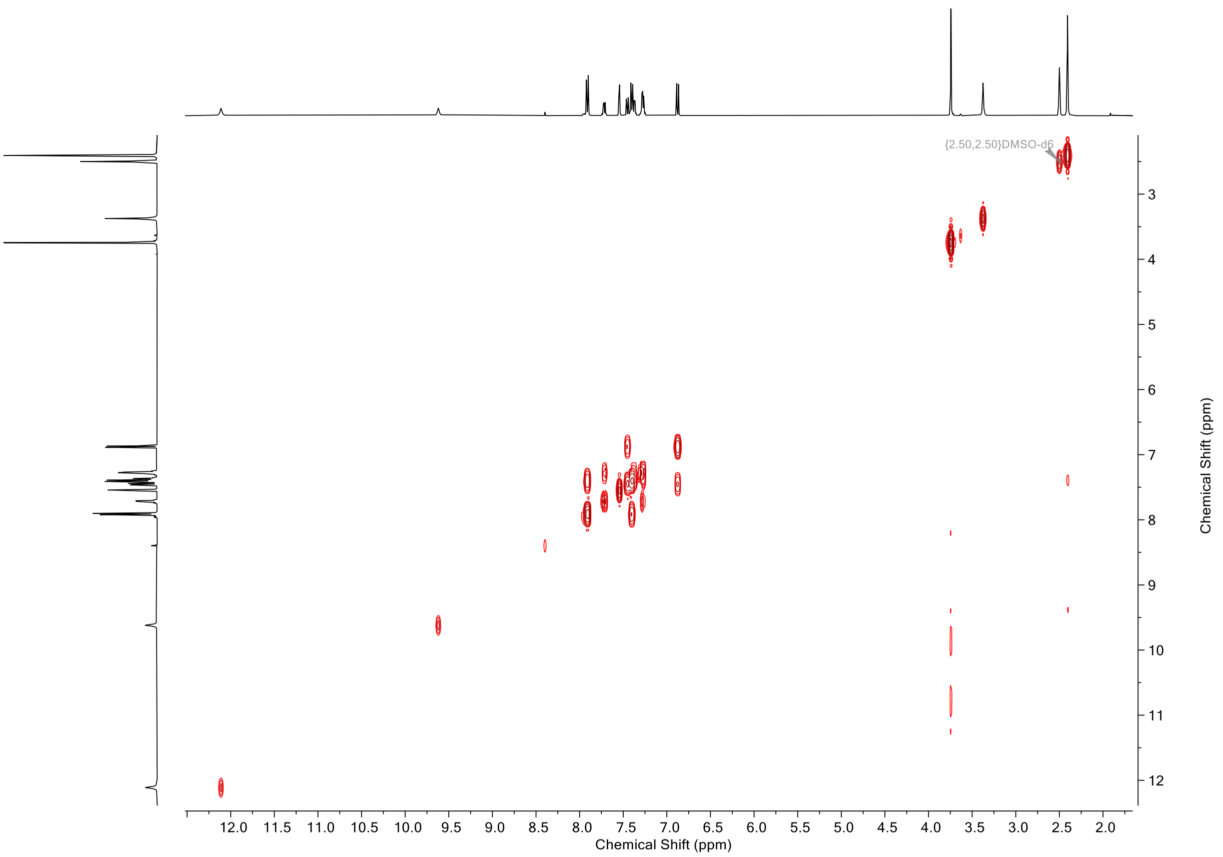

Figure S250: COSY (DMSO-d_6): 1*H*-*N*-(2-(4-Hydroxy-3-methoxyphenyl)-benzo[*d*]imidazol-1-yl)-4-methylbenzamide (**3q**).

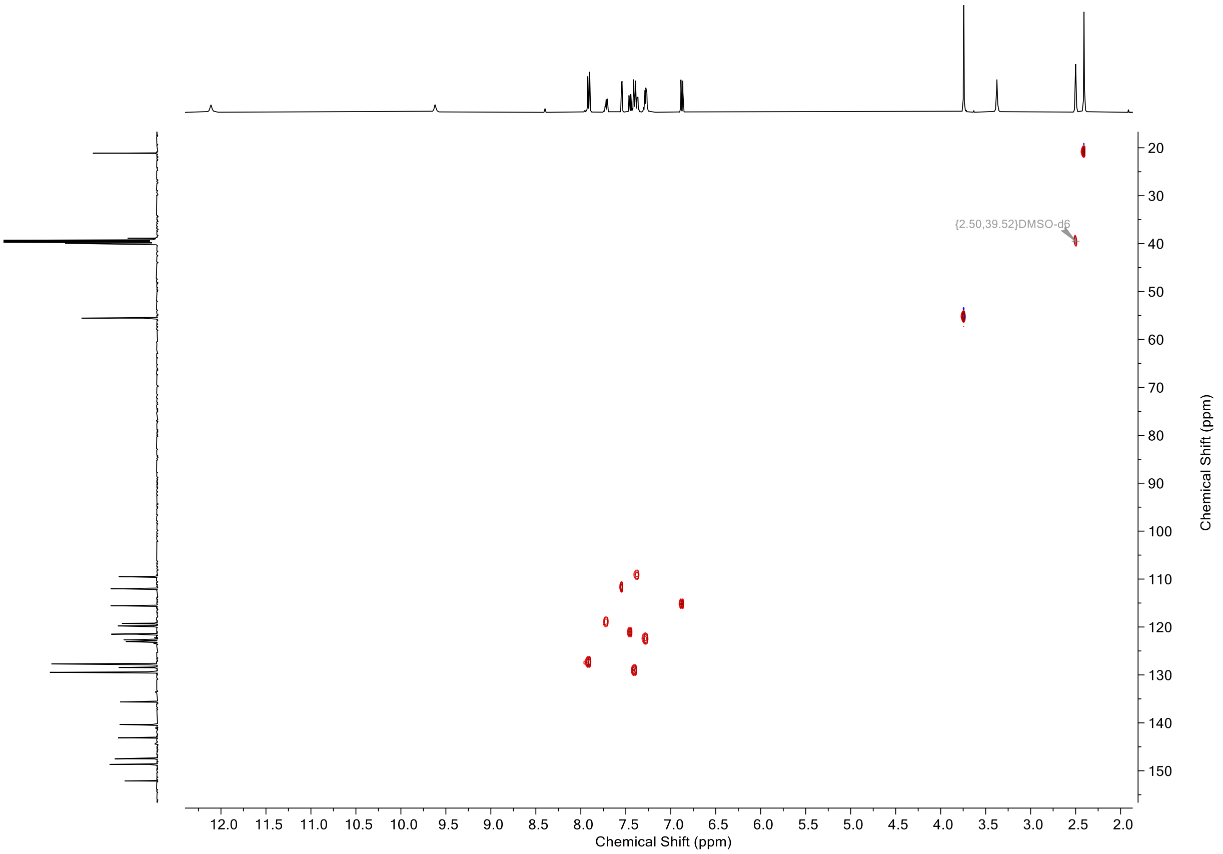

Figure S251: HSQC (DMSO-d_6): 1*H*-*N*-(2-(4-Hydroxy-3-methoxyphenyl)-benzo[*d*]imidazol-1-yl)-4-methylbenzamide (**3q**).

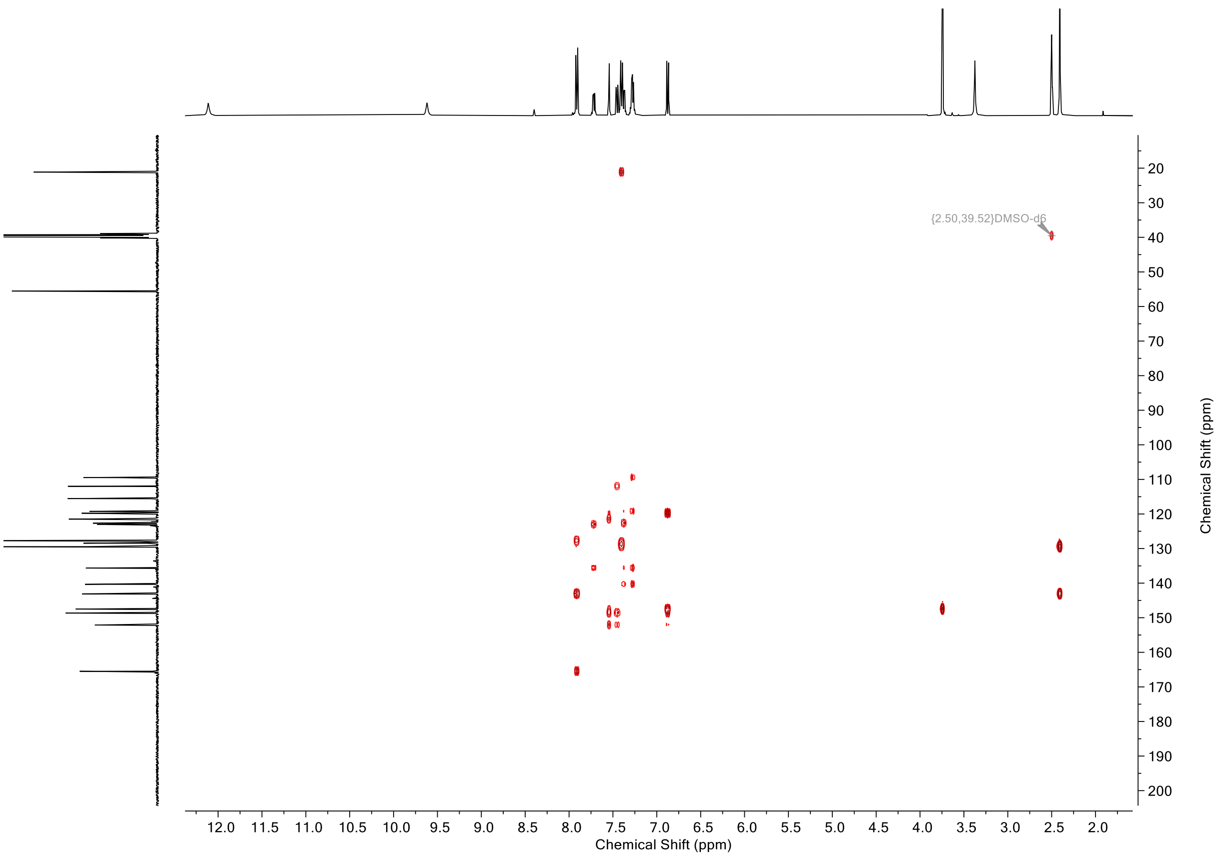

Figure S252: HMBC (DMSO-d_6): 1*H*-*N*-(2-(4-Hydroxy-3-methoxyphenyl)-benzo[*d*]imidazol-1-yl)-4-methylbenzamide (**3q**).

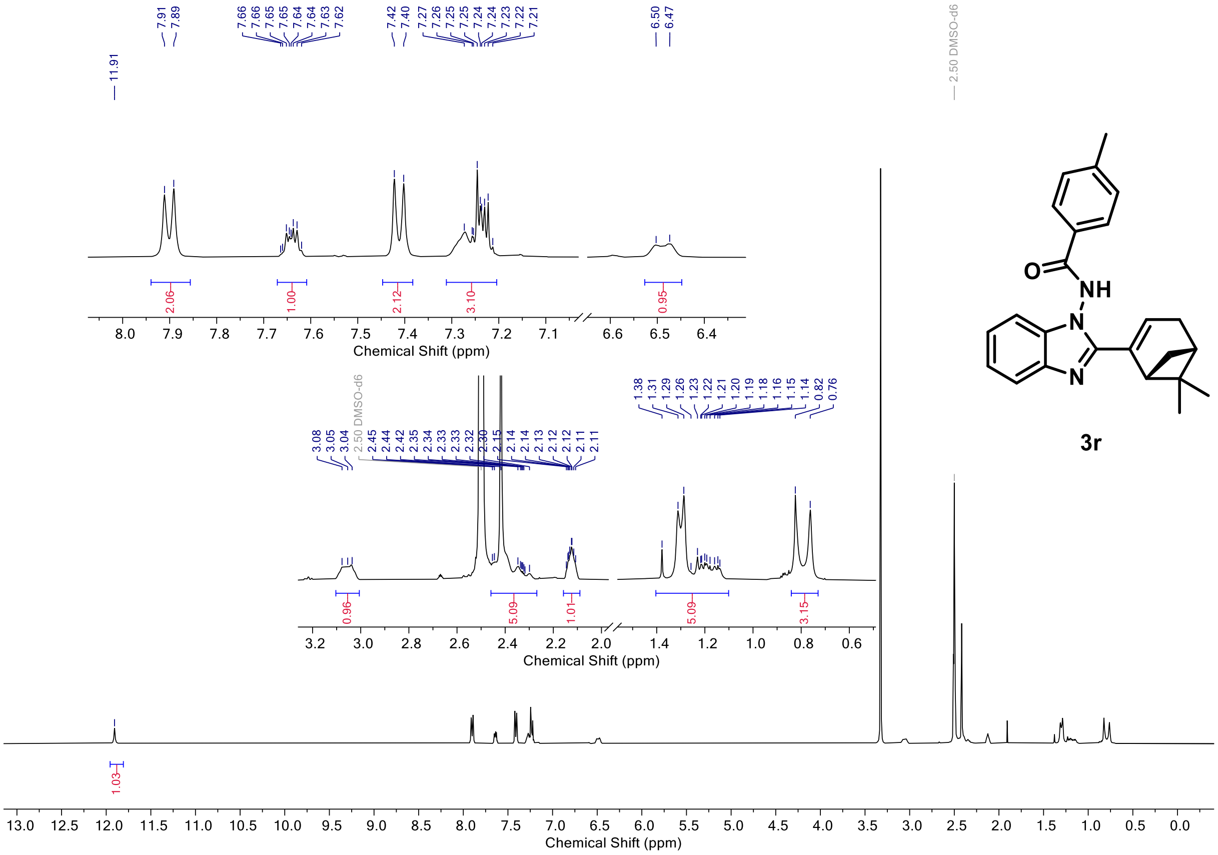

Figure S253: ^{1}H NMR (400 MHz, DMSO-d_6): 1*H*-*N*-(2-((1*R*,5*S*)-6,6-Dimethylbicyclo[3.1.1]hept-2-en-2-yl)-benzo[*d*]imidazol-1-yl)-4-methylbenzamide (**3r**).

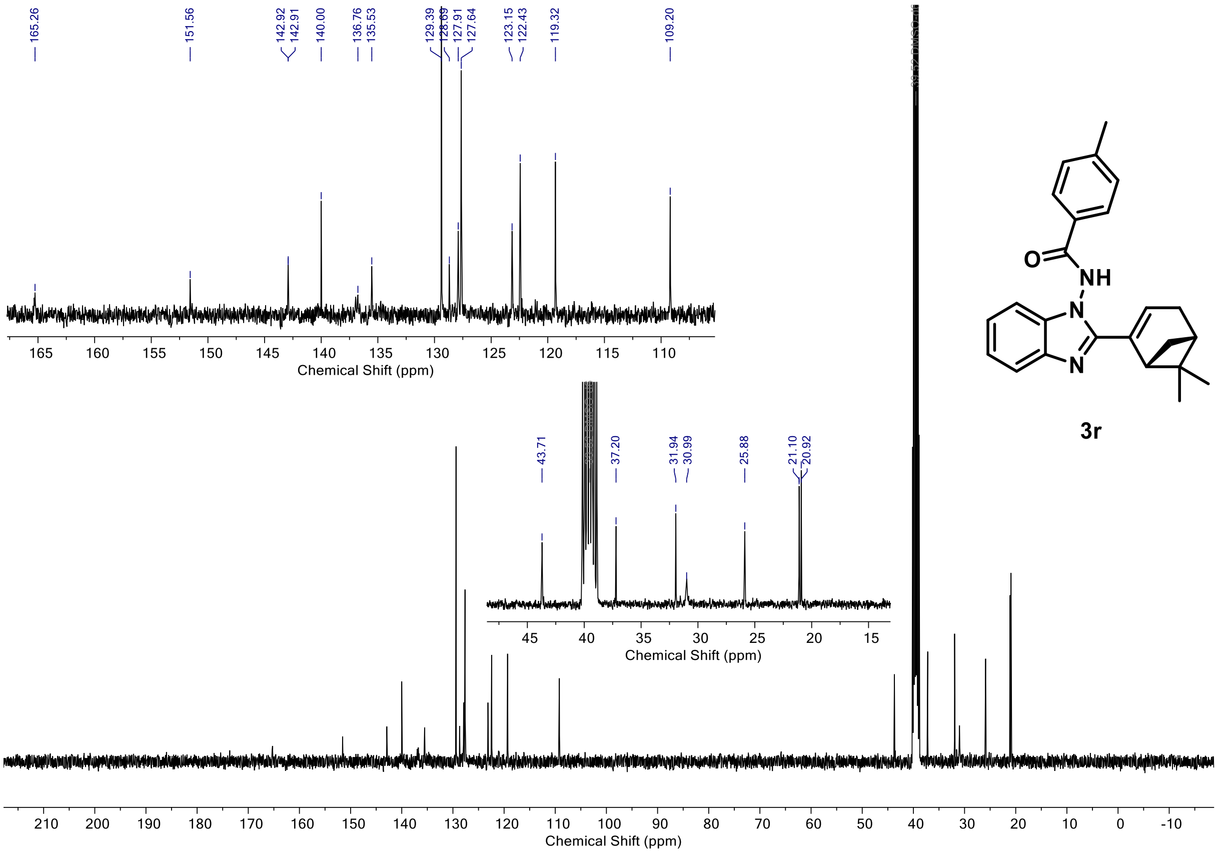

Figure S254: ^{13}C{^{1}H} NMR (101 MHz, DMSO-d_6): 1*H*-*N*-(2-((1*R*,5*S*)-6,6-Dimethylbicyclo[3.1.1]hept-2-en-2-yl)-benzo[*d*]imidazol-1-yl)-4-methylbenzamide (**3r**).

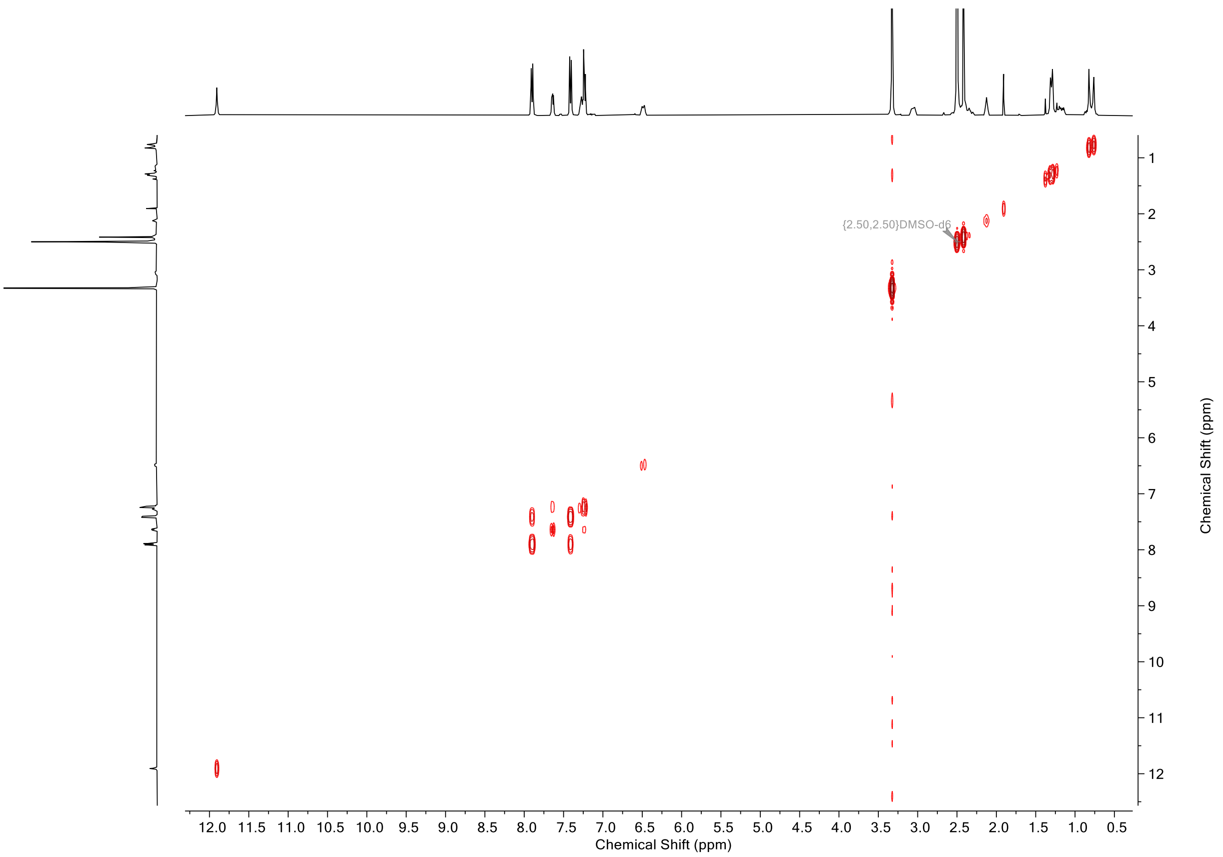

Figure S255: COSY (DMSO-d_6): 1*H*-*N*-(2-((1*R*,5*S*)-6,6-Dimethylbicyclo[3.1.1]hept-2-en-2-yl)-benzo[*d*]imidazol-1-yl)-4-methylbenzamide (**3r**).

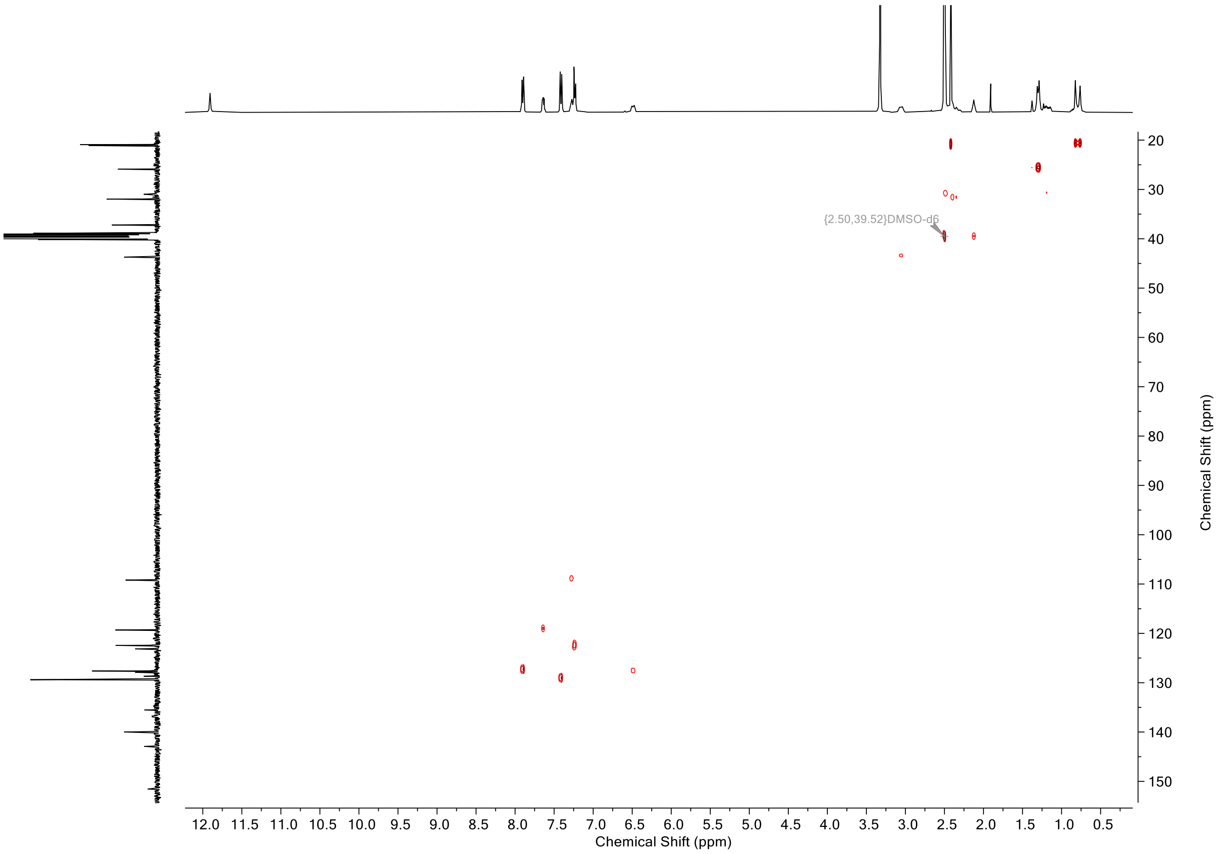

Figure S256: HSQC (DMSO-d_6): 1*H*-*N*-(2-((1*R*,5*S*)-6,6-Dimethylbicyclo[3.1.1]hept-2-en-2-yl)-benzo[*d*]imidazol-1-yl)-4-methylbenzamide (**3r**).

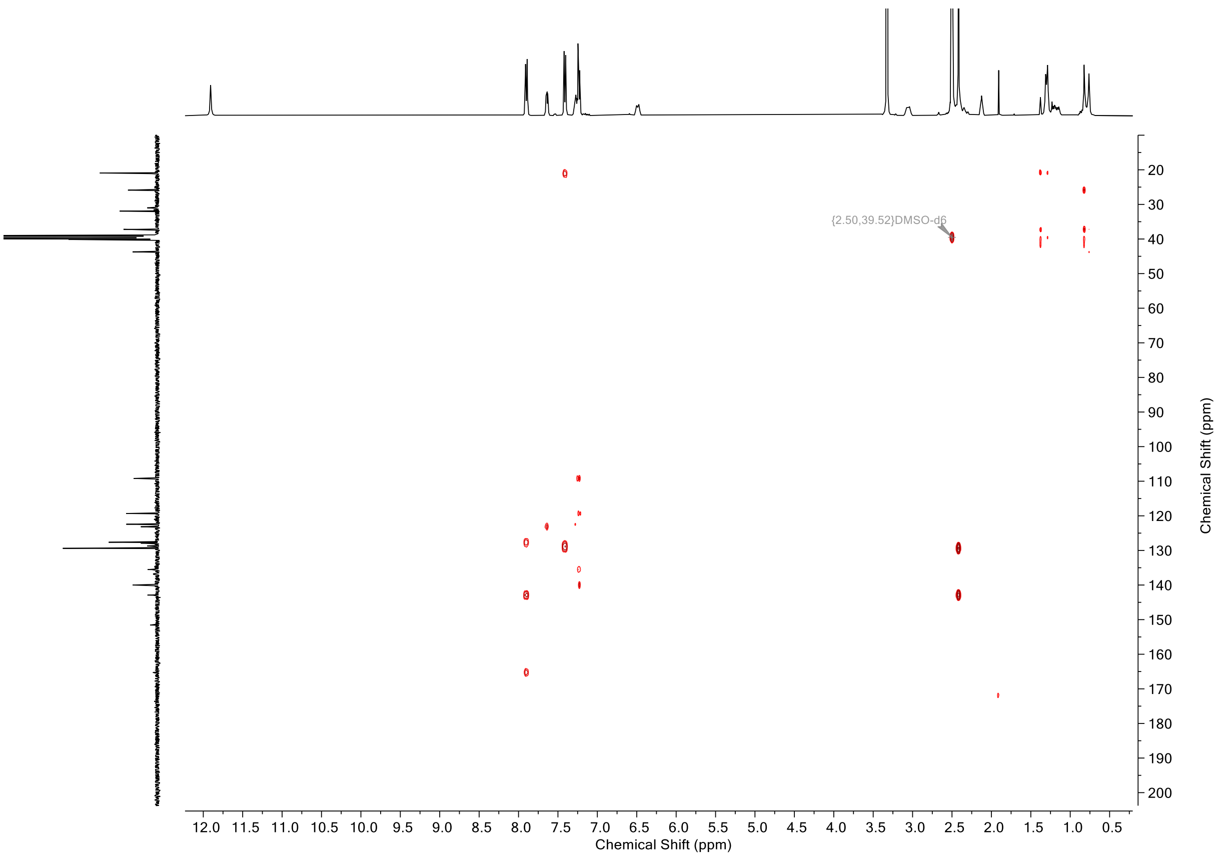

Figure S257: HMBC (DMSO-d_6): 1*H*-*N*-(2-((1*R*,5*S*)-6,6-Dimethylbicyclo[3.1.1]hept-2-en-2-yl)-benzo[*d*]imidazol-1-yl)-4-methylbenzamide (**3r**).

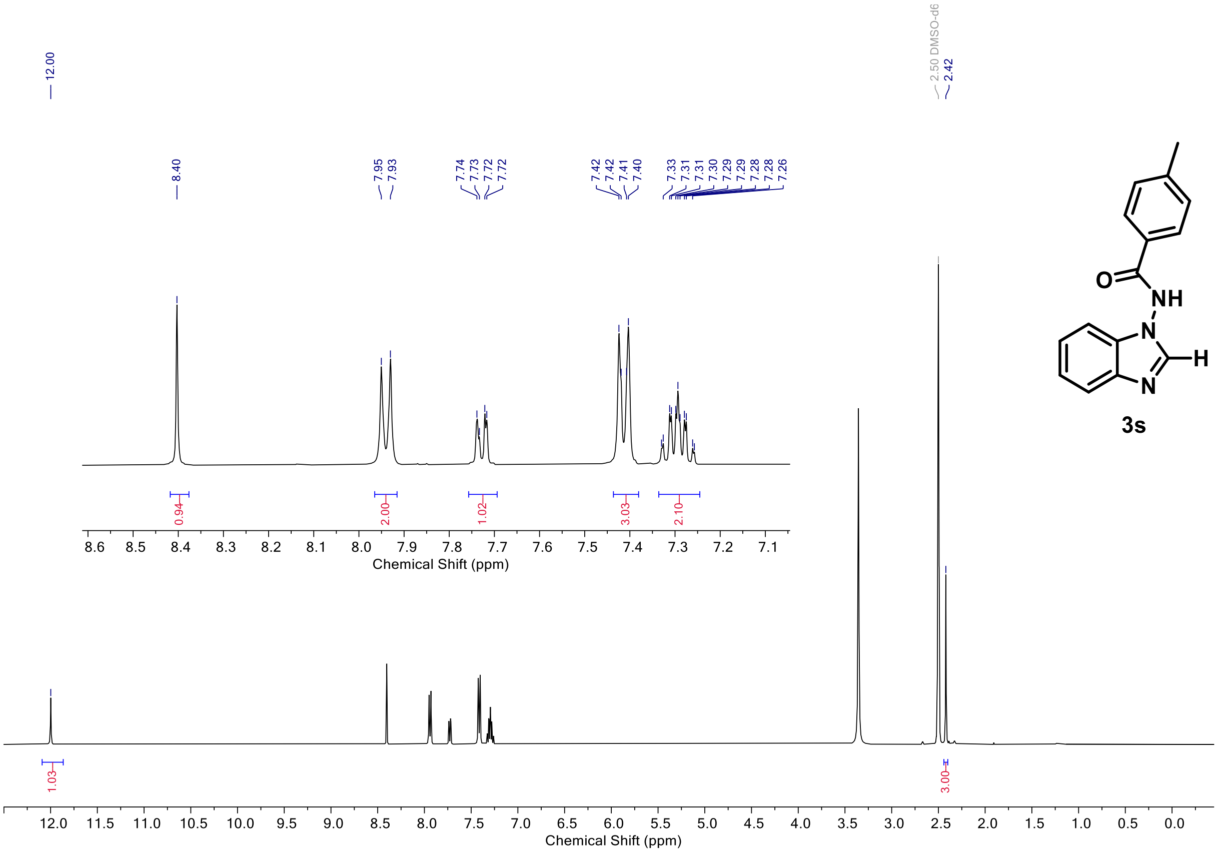

Figure S258: ^{1}H NMR (400 MHz, DMSO-d_6): 1*H*-*N*-(Benzo[*d*]imidazol-1-yl)-4-methylbenzamide (**3s**).

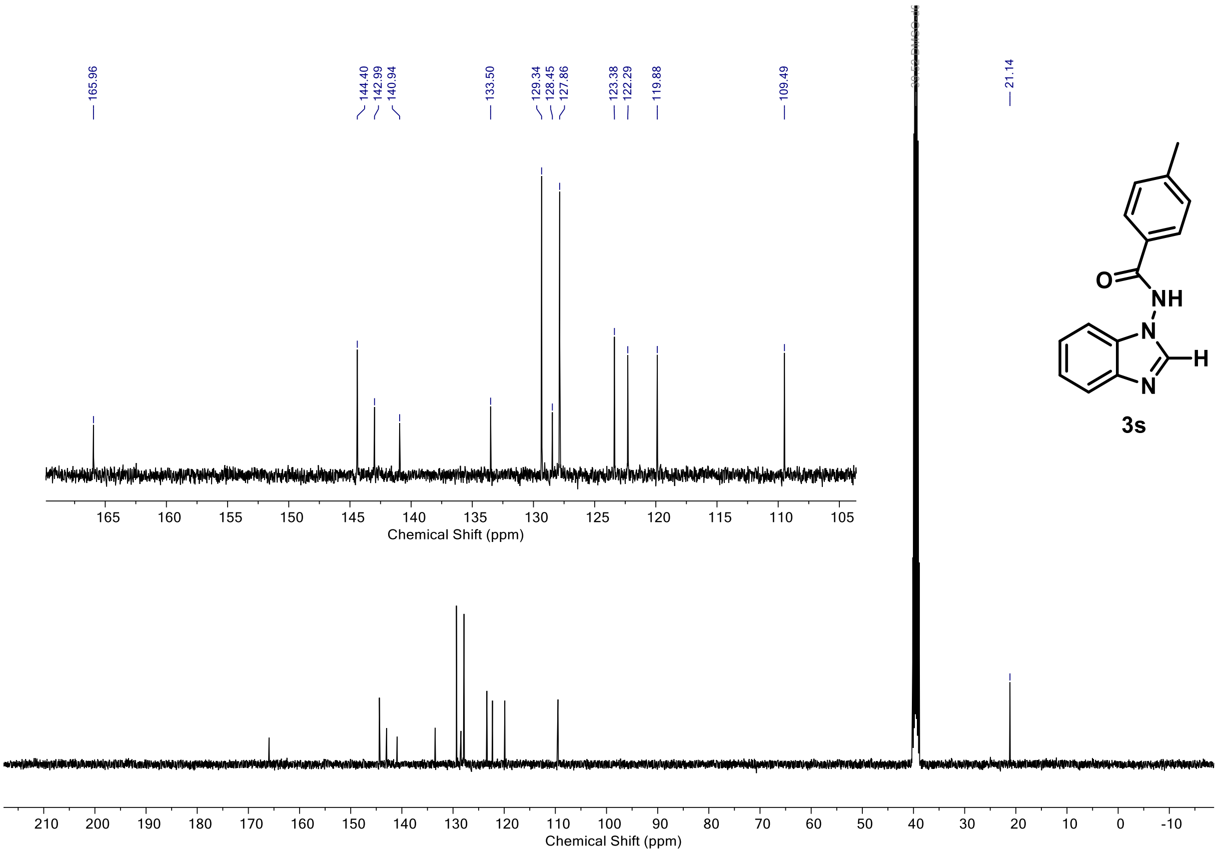

Figure S259: ^{13}C{^{1}H} NMR (101 MHz, DMSO-d_6): 1*H*-*N*-(Benzo[*d*]imidazol-1-yl)-4-methylbenzamide (**3s**).

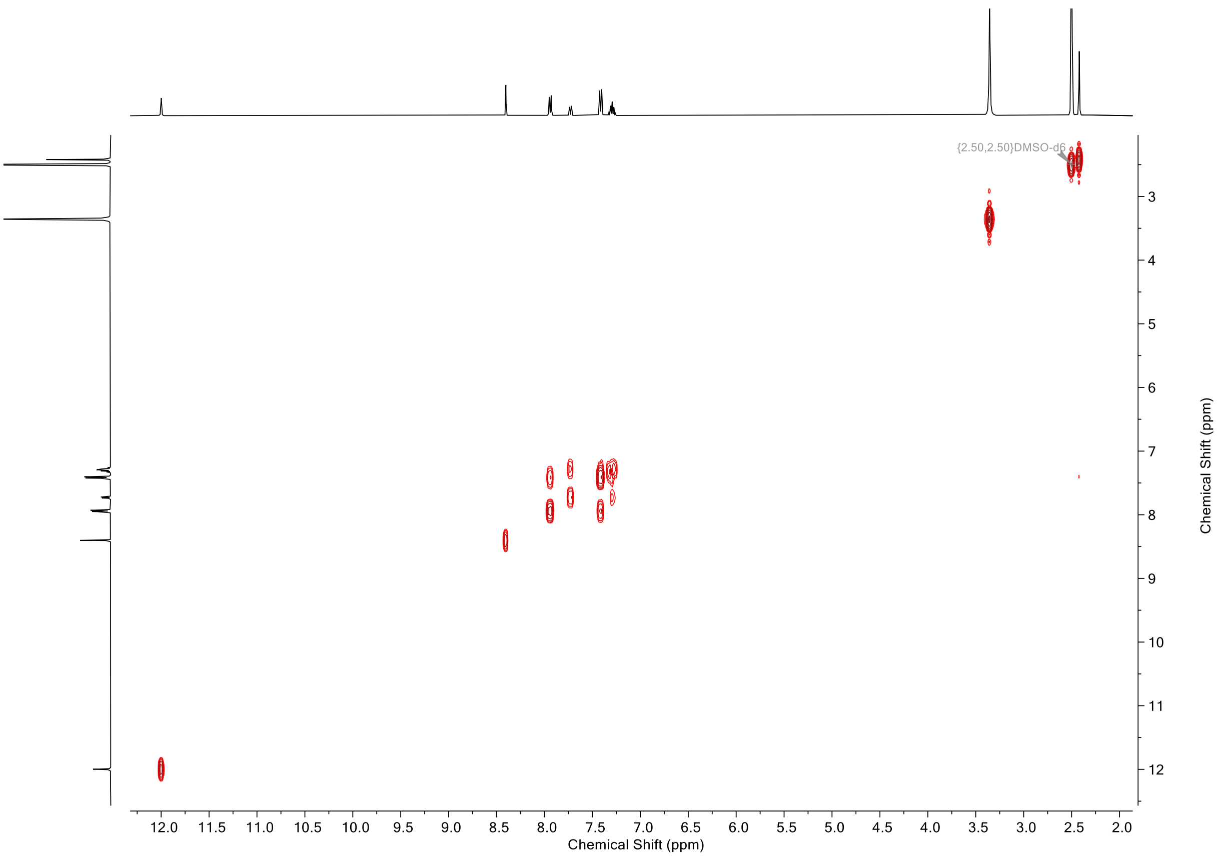

Figure S260: COSY (DMSO-d_6): 1*H*-*N*-(Benzo[*d*]imidazol-1-yl)-4-methylbenzamide (**3s**).

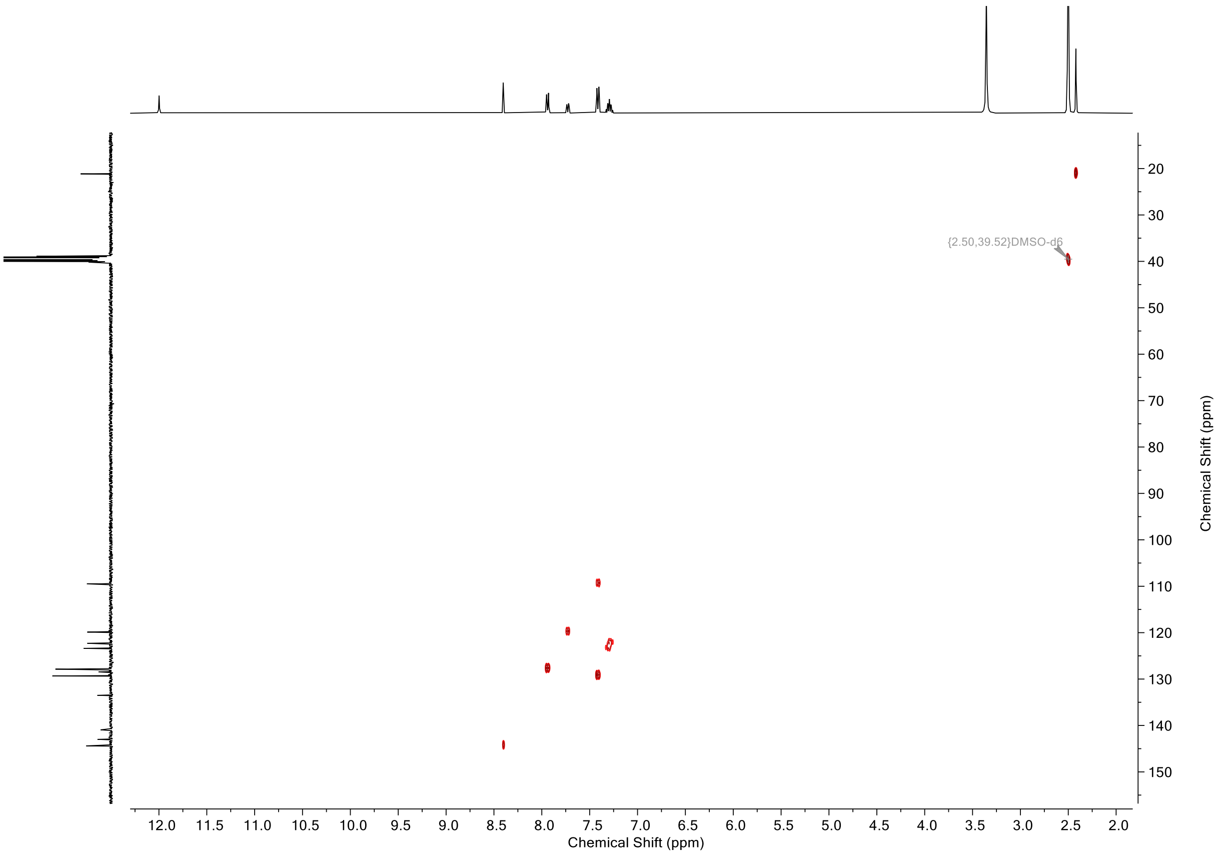

Figure S261: HSQC (DMSO-d_6): 1*H*-*N*-(Benzo[*d*]imidazol-1-yl)-4-methylbenzamide (**3s**).

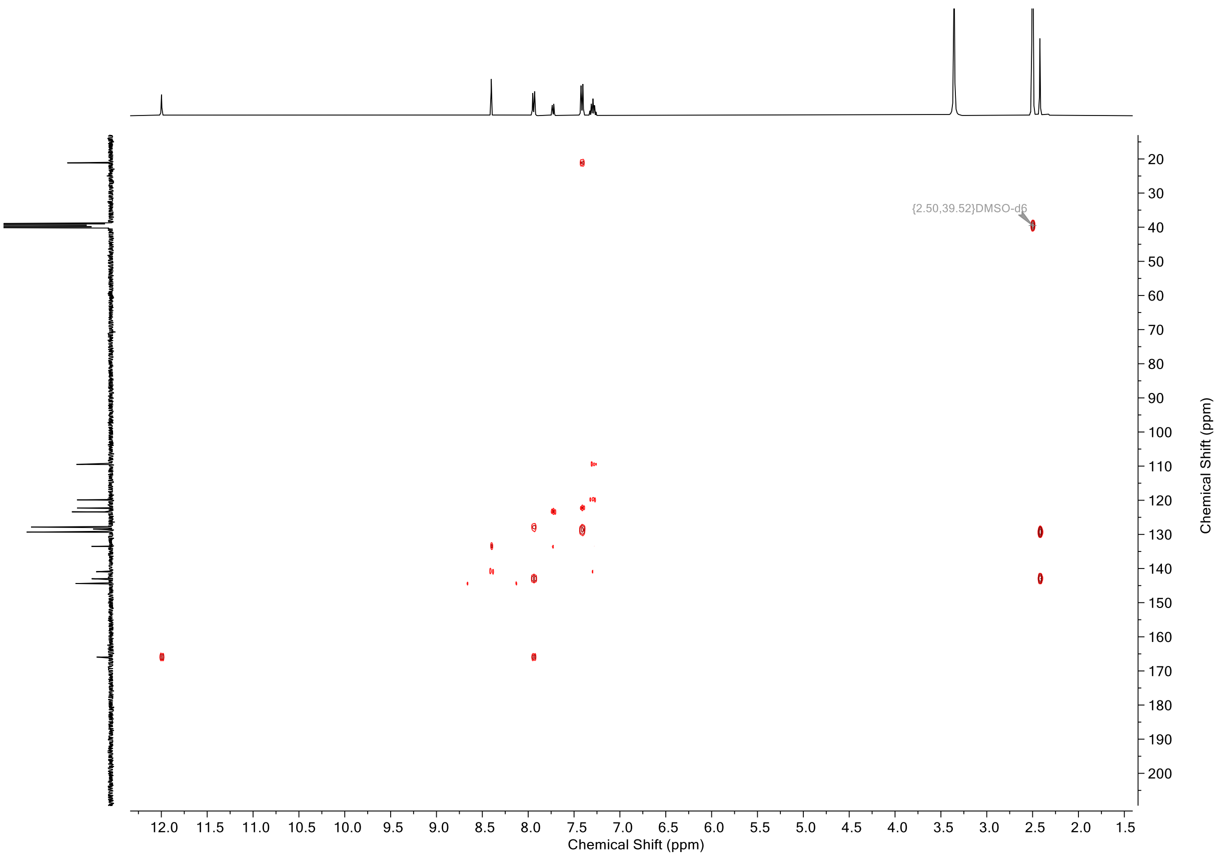

Figure S262: HMBC (DMSO-d_6): 1*H*-*N*-(Benzo[*d*]imidazol-1-yl)-4-methylbenzamide (**3s**).

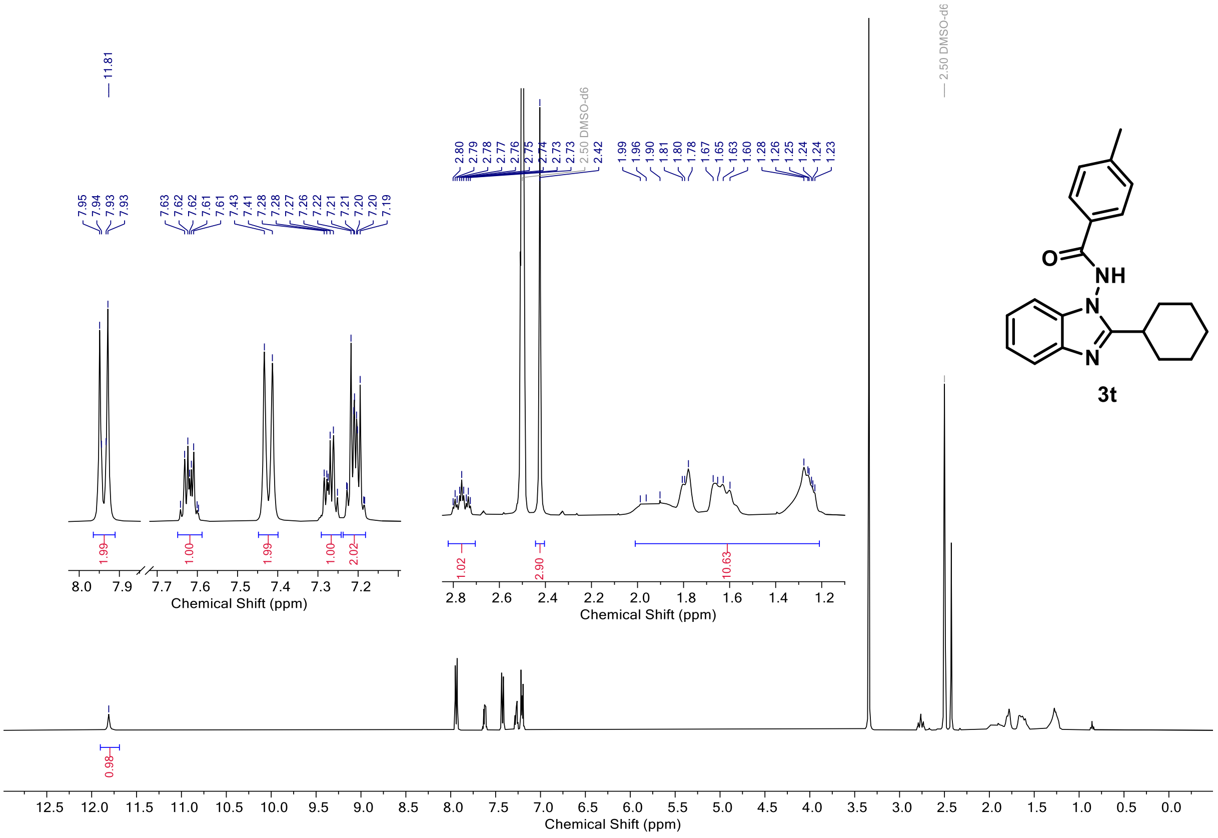

Figure S263: ^{1}H NMR (400 MHz, DMSO-d_6): 1*H*-*N*-(2-Cyclohexyl-benzo[*d*]imidazol-1-yl)-4-methylbenzamide (**3t**).

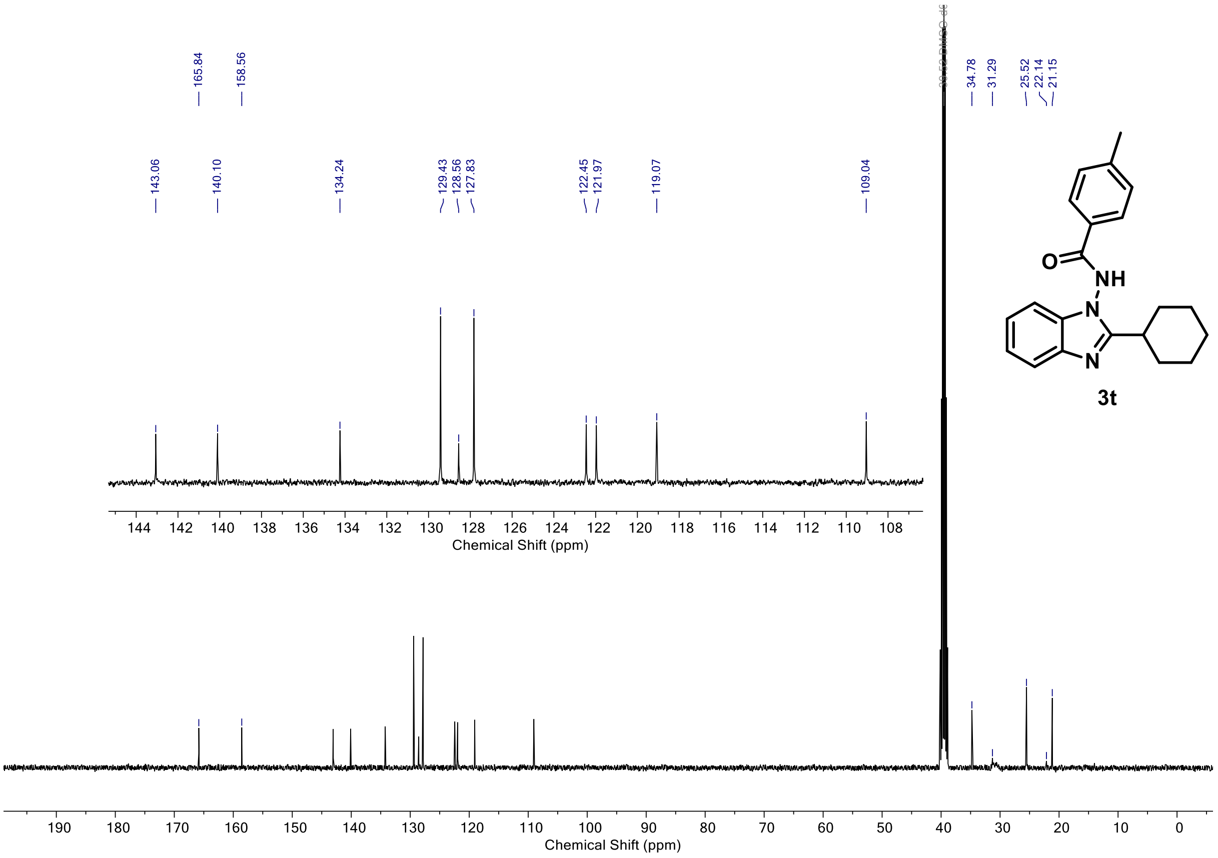

Figure S264: ^{13}C{^{1}H} NMR (101 MHz, DMSO-d_6): 1*H*-*N*-(2-Cyclohexyl-benzo[*d*]imidazol-1-yl)-4-methylbenzamide (**3t**).

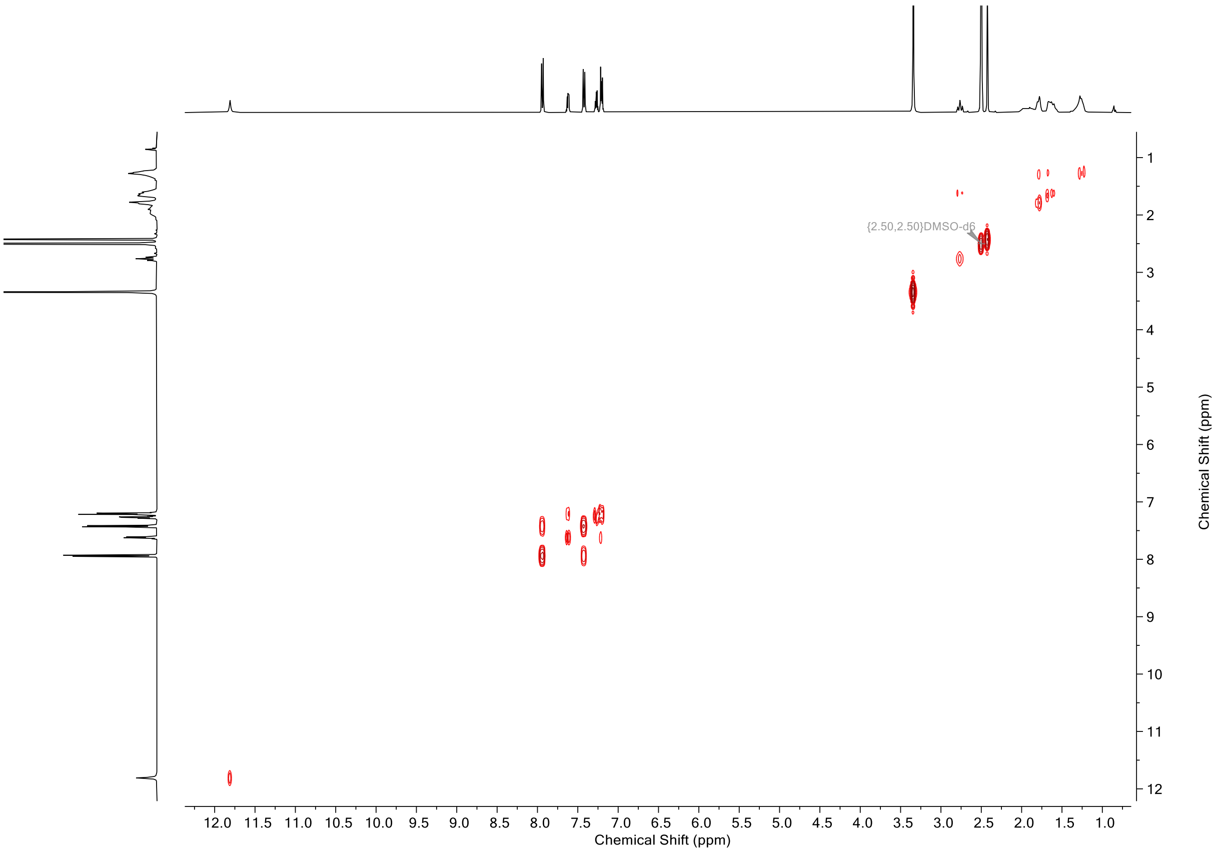

Figure S265: COSY (DMSO-d_6): 1*H*-*N*-(2-Cyclohexyl-benzo[*d*]imidazol-1-yl)-4-methylbenzamide (**3t**).

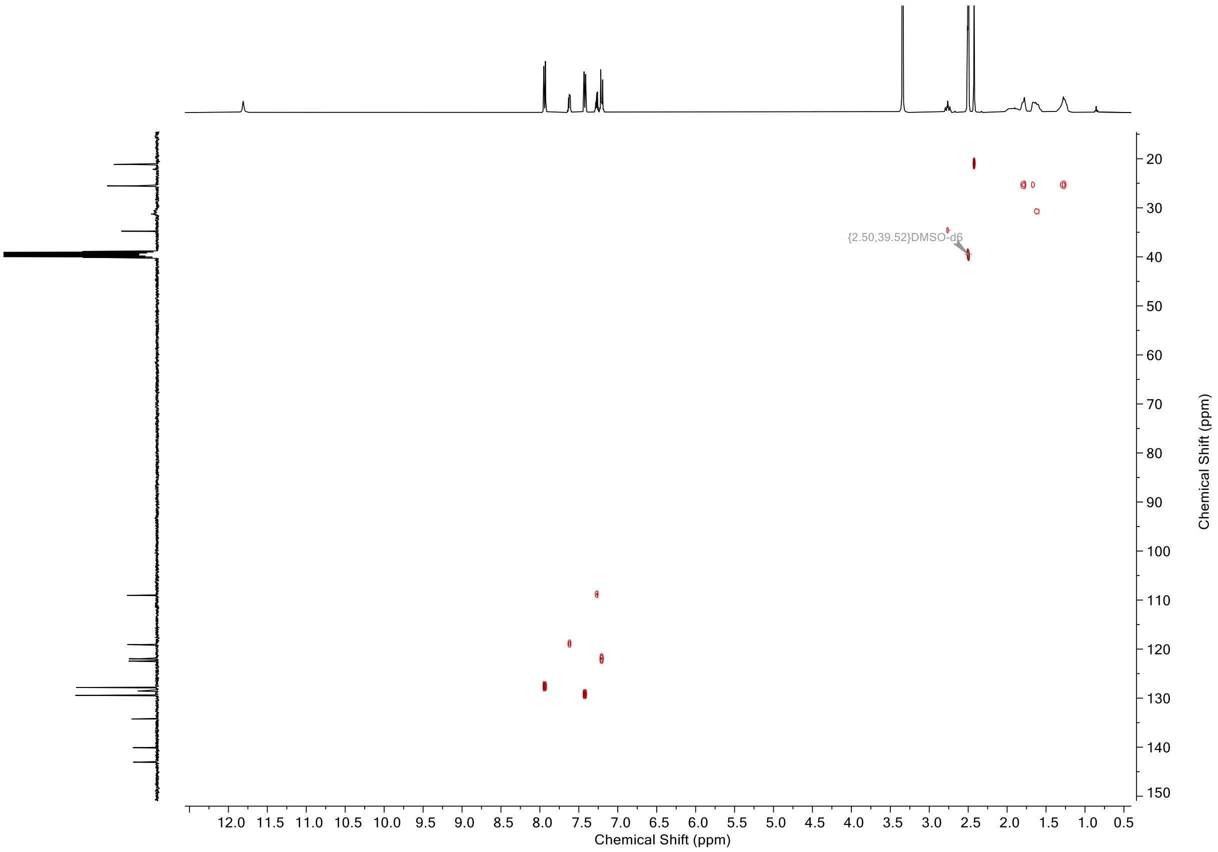

Figure S266: HSQC (DMSO-d_6): 1*H*-*N*-(2-Cyclohexyl-benzo[*d*]imidazol-1-yl)-4-methylbenzamide (**3t**).

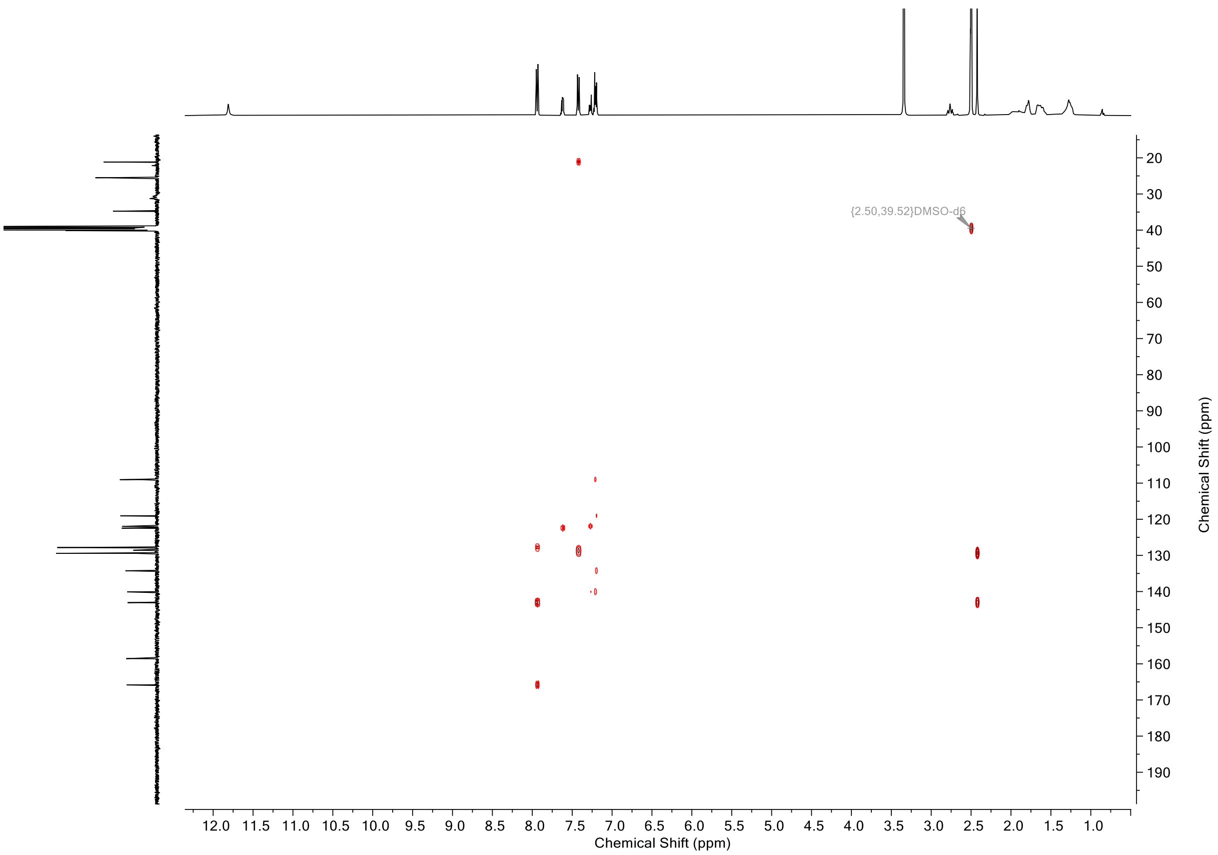

Figure S267: HMBC (DMSO-d_6): 1*H*-*N*-(2-Cyclohexyl-benzo[*d*]imidazol-1-yl)-4-methylbenzamide (**3t**).

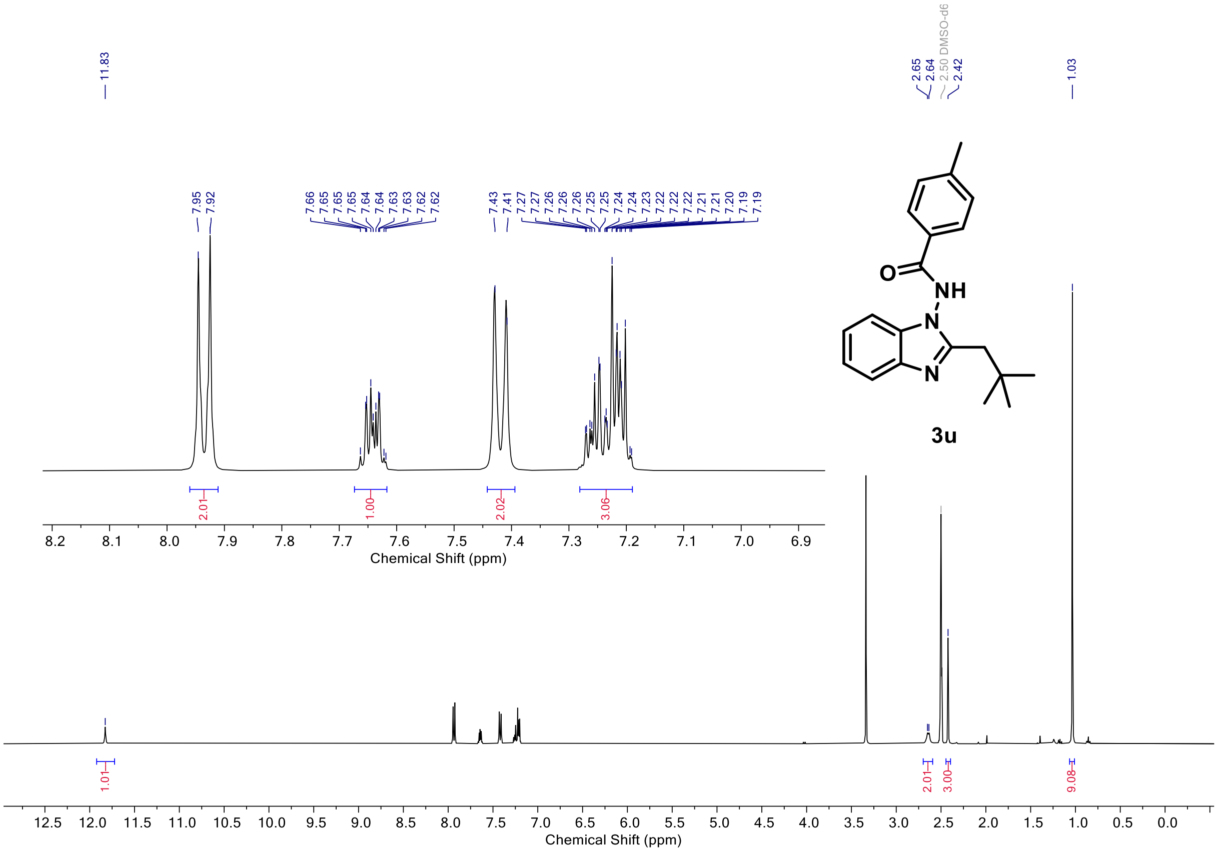

Figure S268: ^{1}H NMR (400 MHz, DMSO-d_6): 1*H*-4-Methyl-*N*-(2-neopentyl-benzo[*d*]imidazol-1-yl)benzamide (**3u**).

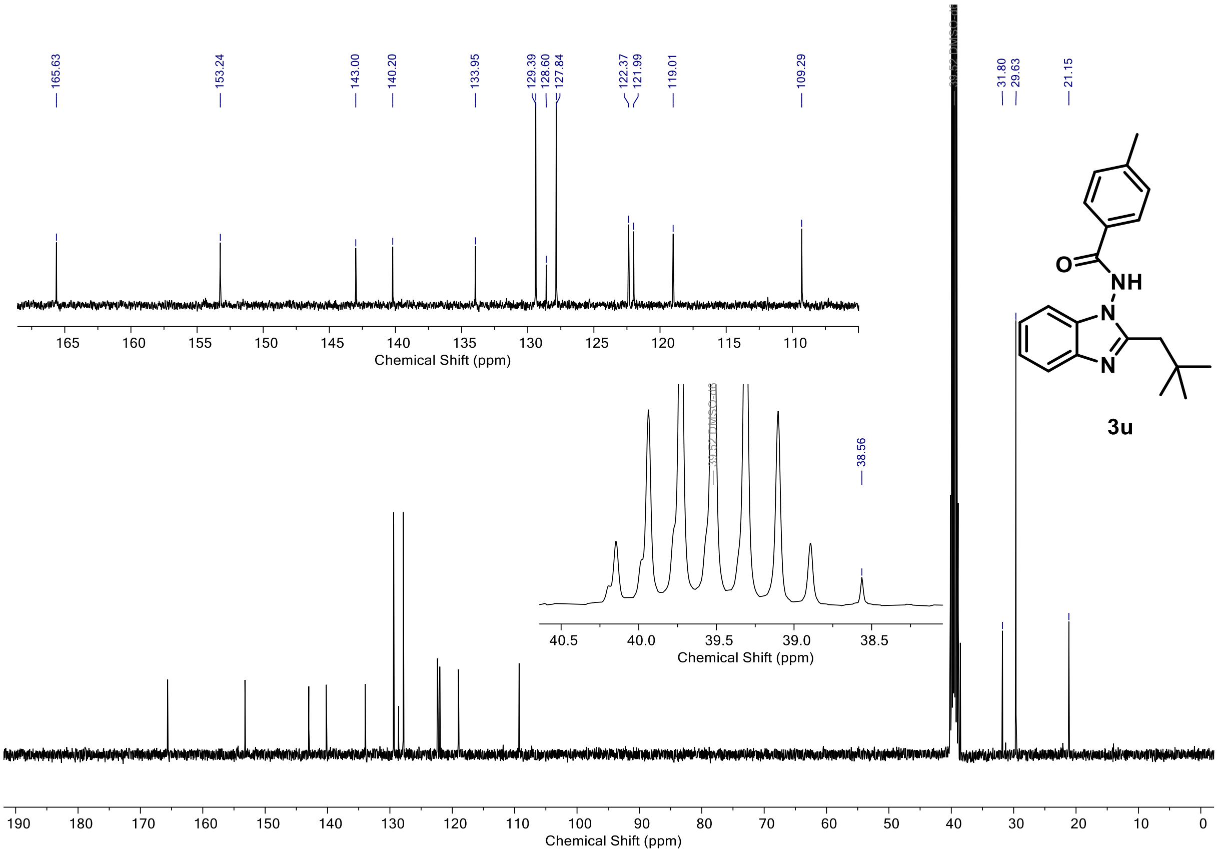

Figure S269: $^{13}C\{^{1}H\}$ NMR (101 MHz, DMSO-d_6): 1*H*-4-Methyl-*N*-(2-neopentyl-benzo[*d*]imidazol-1-yl)benzamide (**3u**).

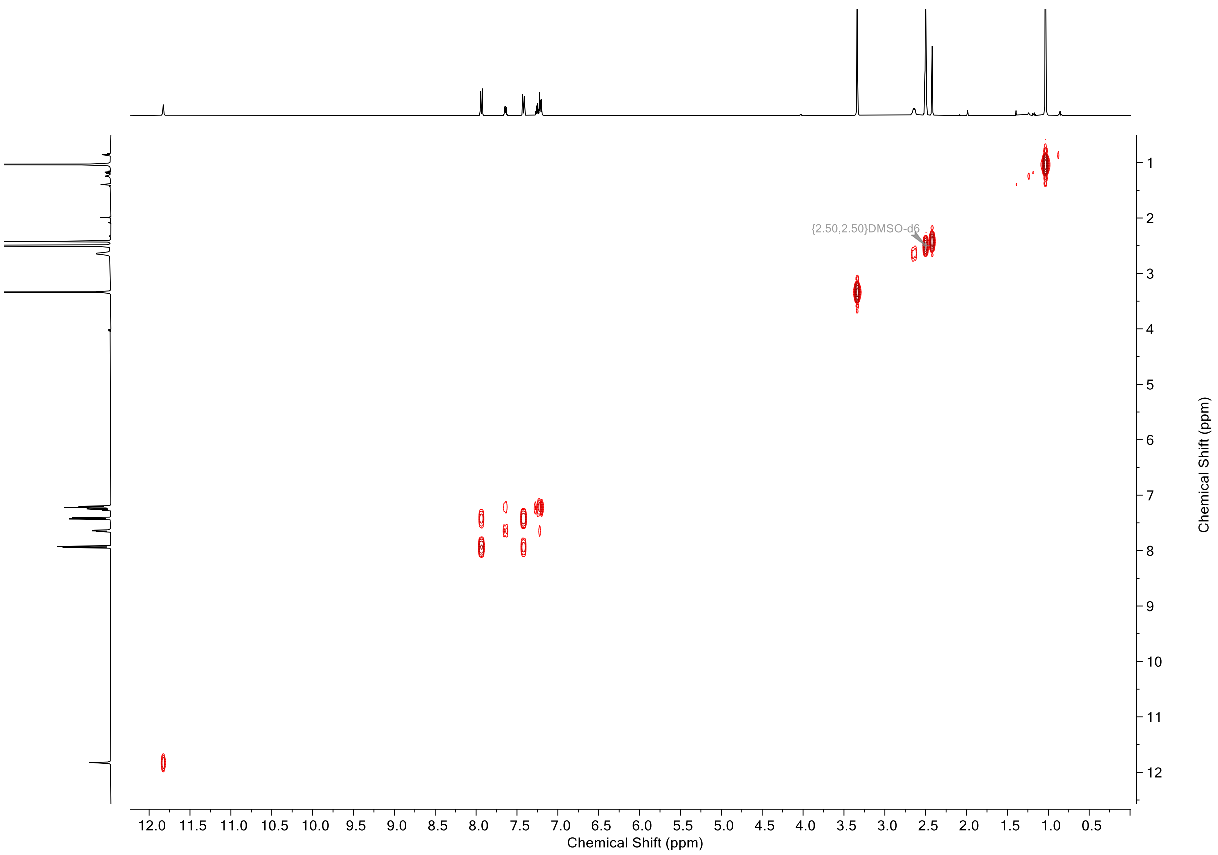

Figure S270: COSY (DMSO-d_6): 1*H*-4-Methyl-*N*-(2-neopentyl-benzo[*d*]imidazol-1-yl)benzamide (**3u**).

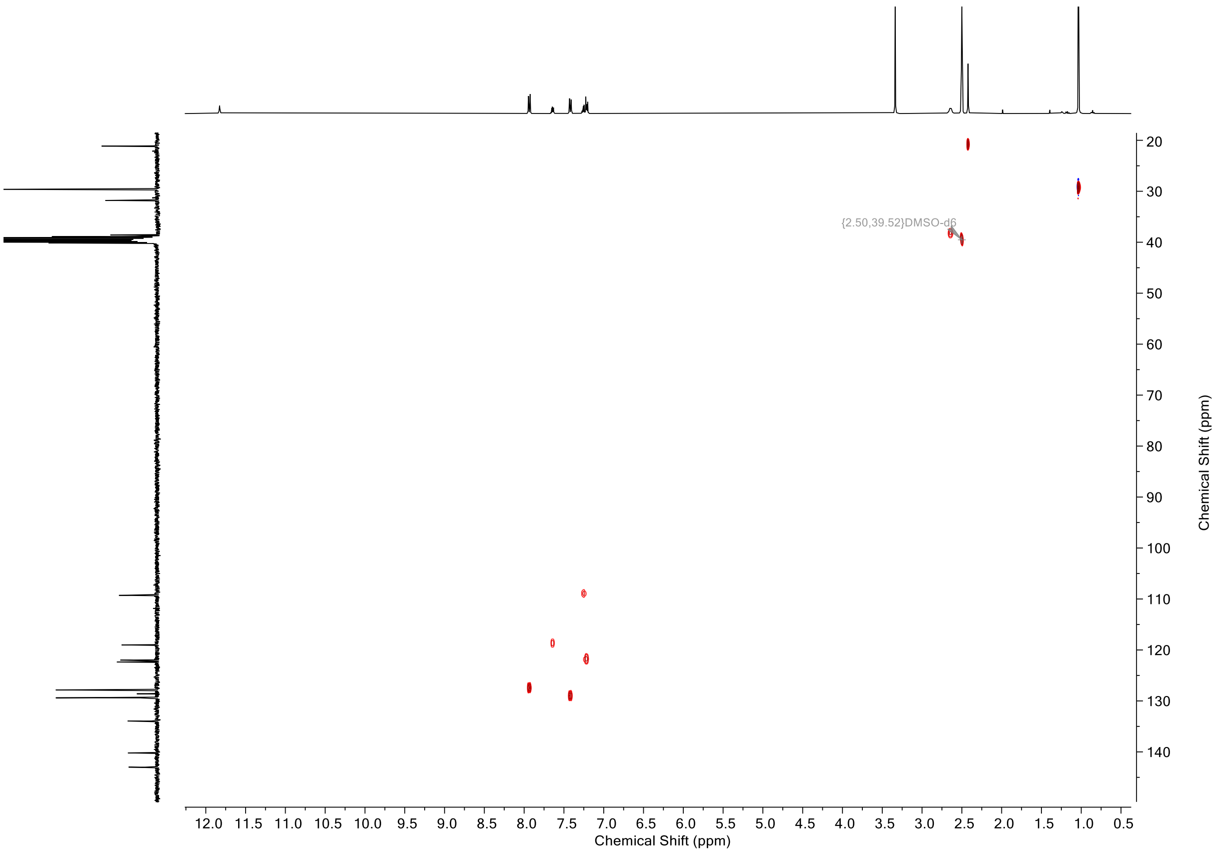

Figure S271: HSQC (DMSO-d_6): 1*H*-4-Methyl-*N*-(2-neopentyl-benzo[*d*]imidazol-1-yl)benzamide (**3u**).

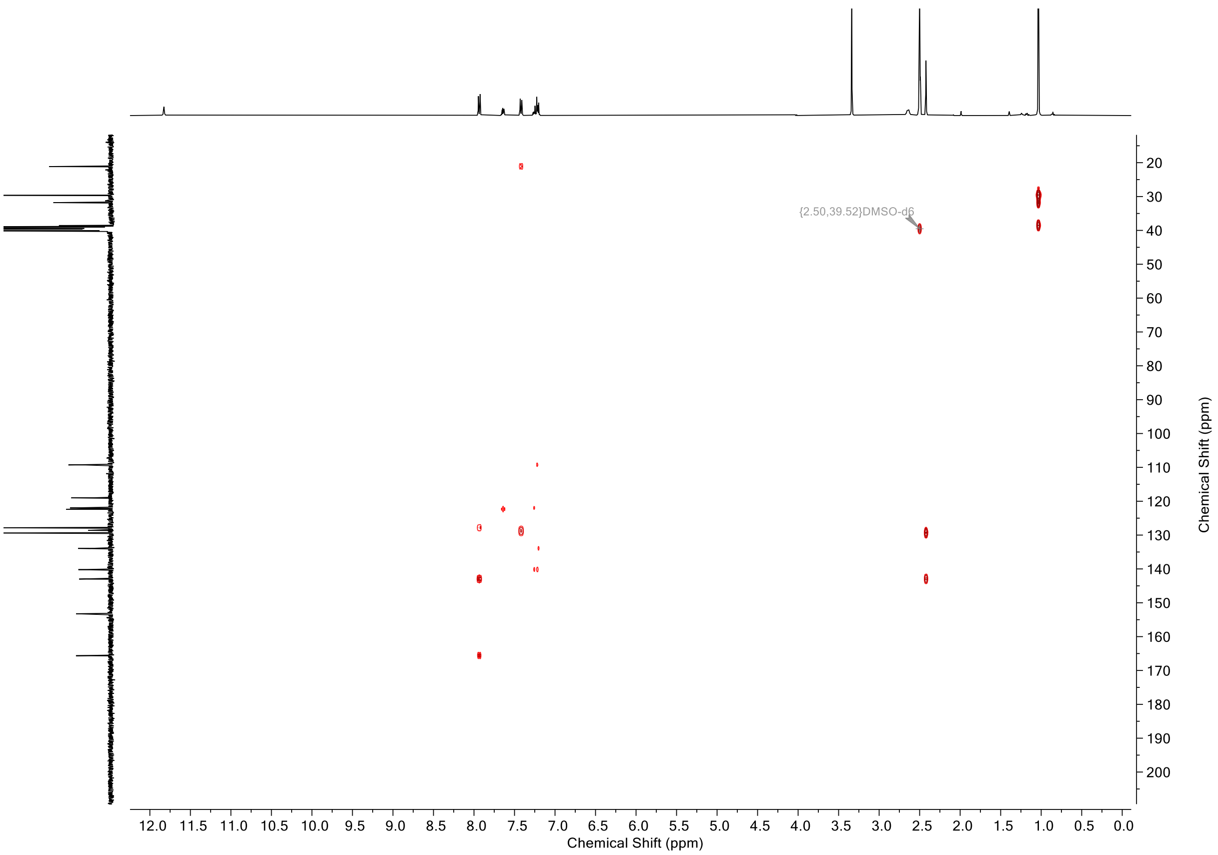

Figure S272: HMBC (DMSO-d_6): 1*H*-4-Methyl-*N*-(2-neopentyl-benzo[*d*]imidazol-1-yl)benzamide (**3u**).

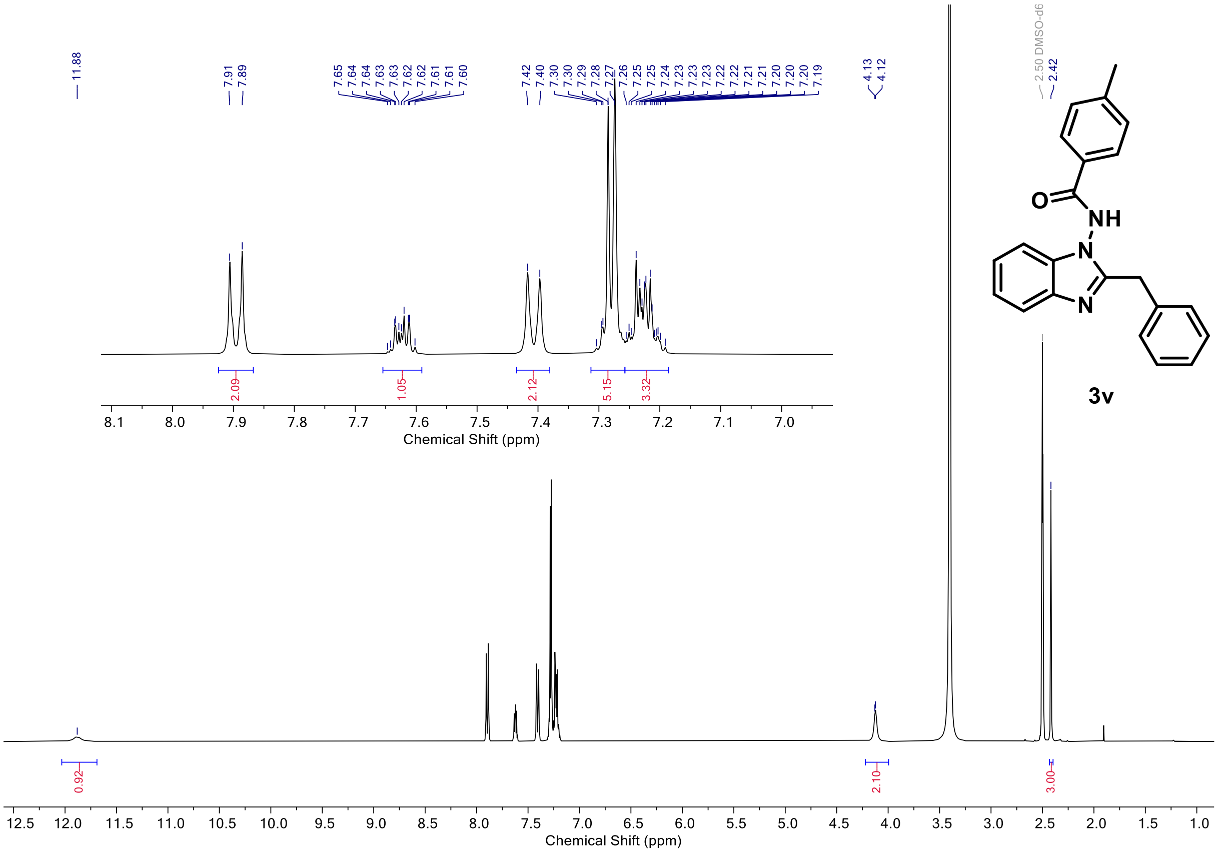

Figure S273: ^{1}H NMR (400 MHz, DMSO-d_6): 1*H*-*N*-(2-Benzyl-benzo[*d*]imidazol-1-yl)-4-methylbenzamide (**3v**).

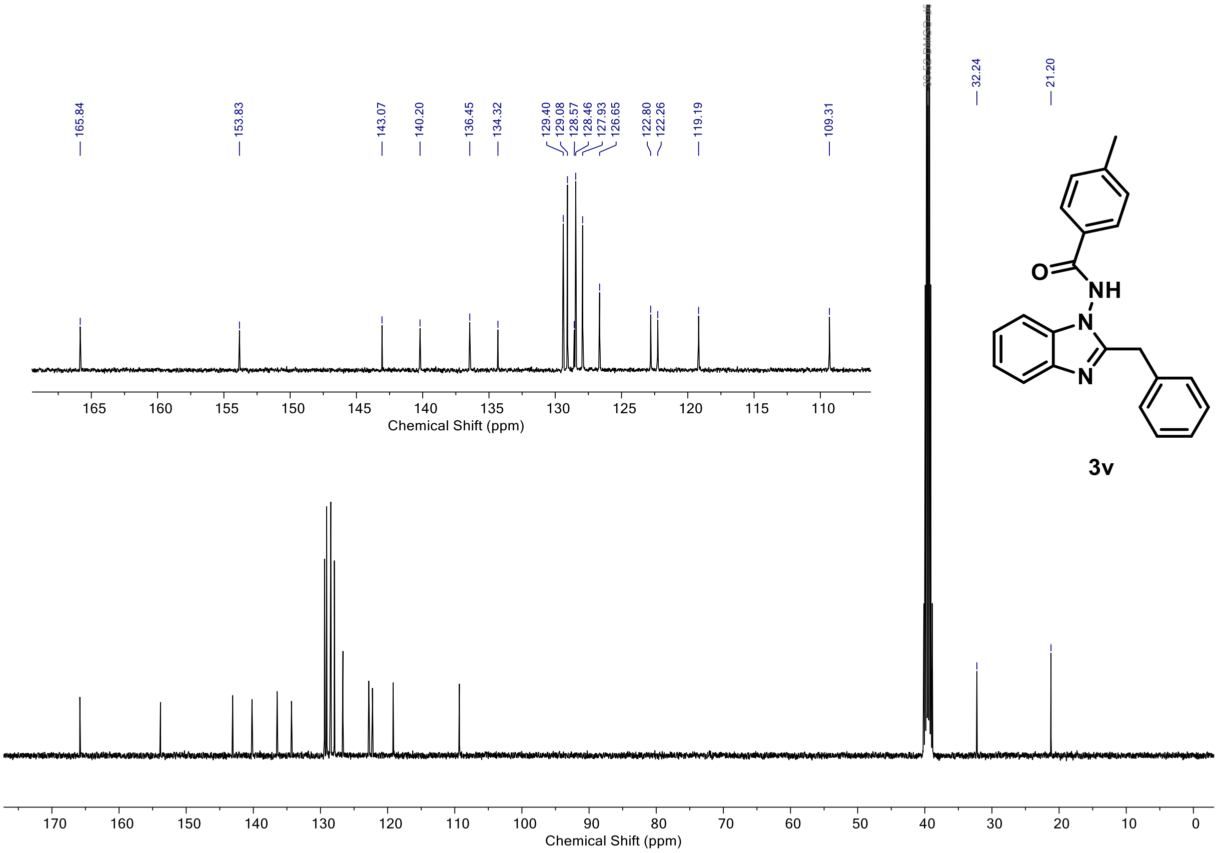

Figure S274: ^{13}C{^{1}H} NMR (101 MHz, DMSO-d_6): 1*H*-*N*-(2-Benzyl-benzo[*d*]imidazol-1-yl)-4-methylbenzamide (**3v**).

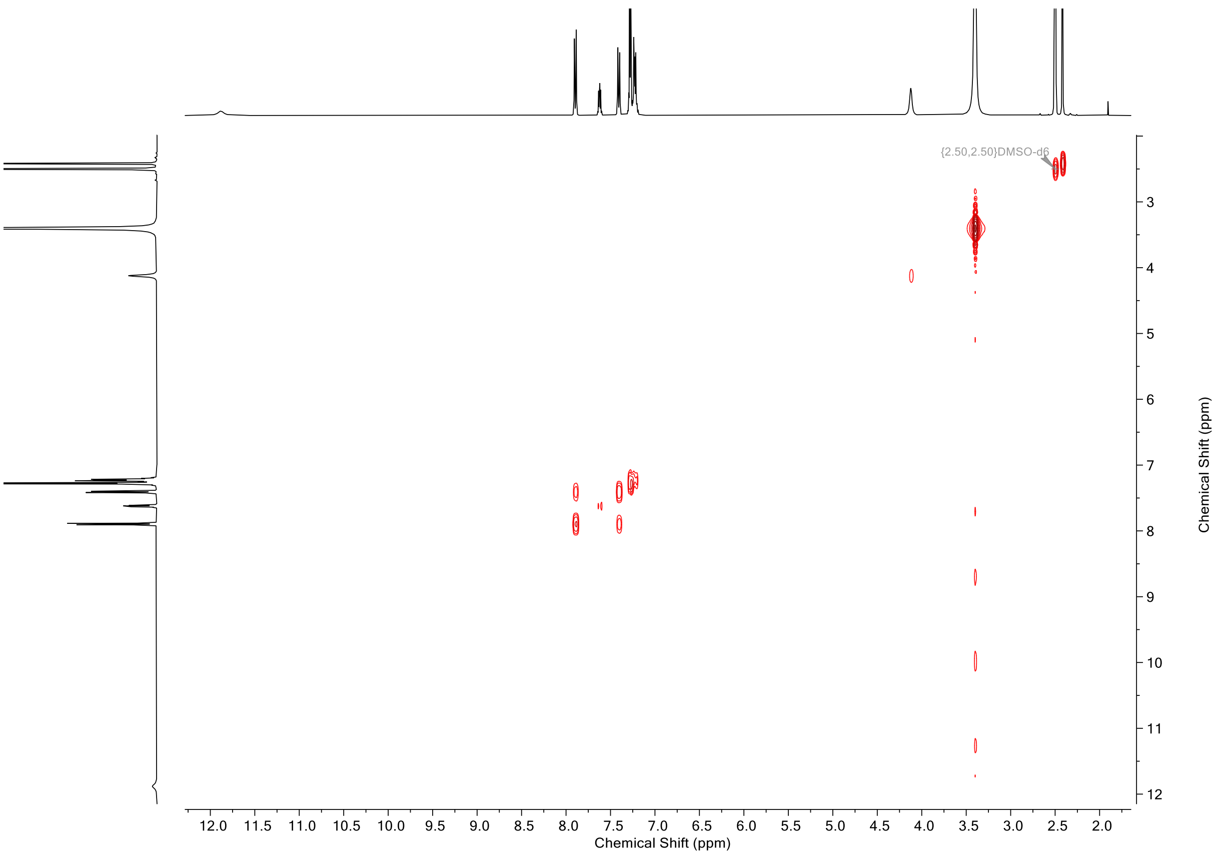

Figure S275: COSY (DMSO-d_6): 1*H*-*N*-(2-Benzyl-benzo[*d*]imidazol-1-yl)-4-methylbenzamide (**3v**).

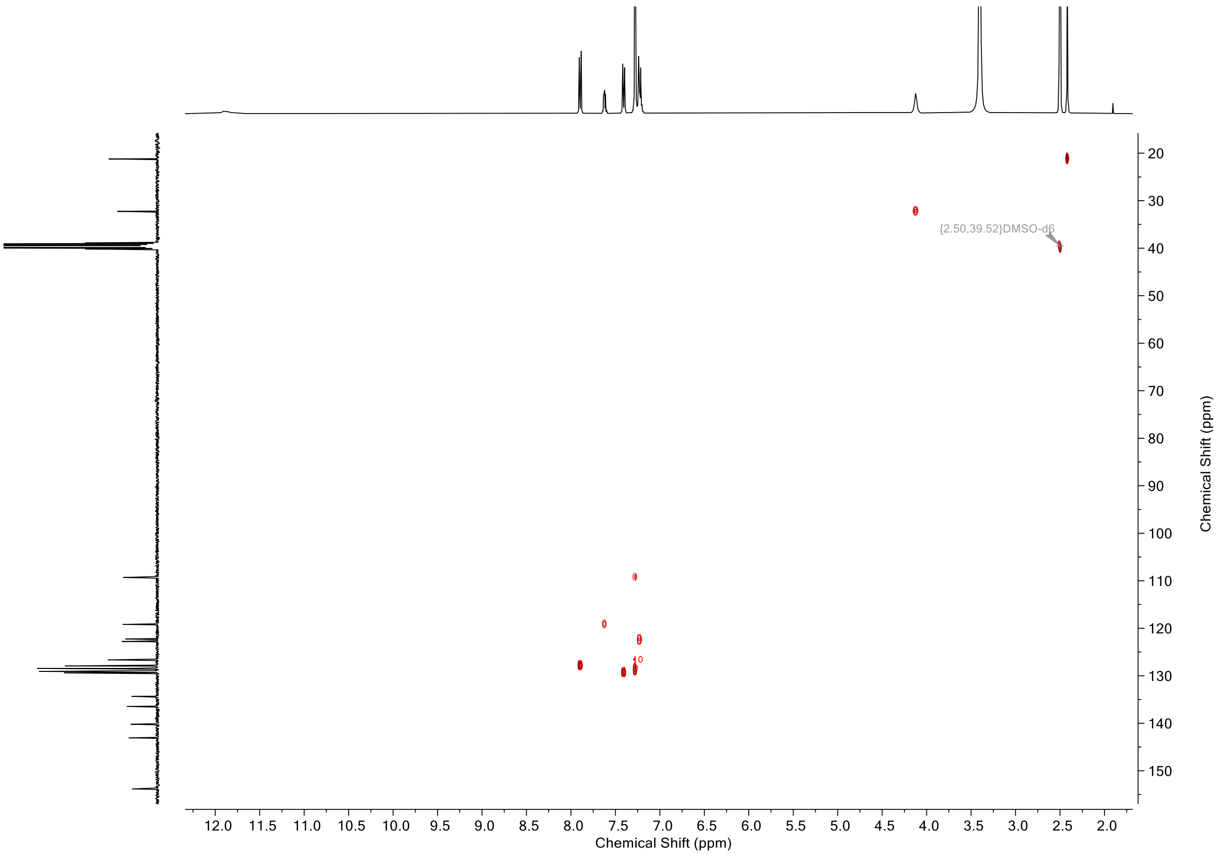

Figure S276: HSQC (DMSO-d_6): 1*H*-*N*-(2-Benzyl-benzo[*d*]imidazol-1-yl)-4-methylbenzamide (**3v**).

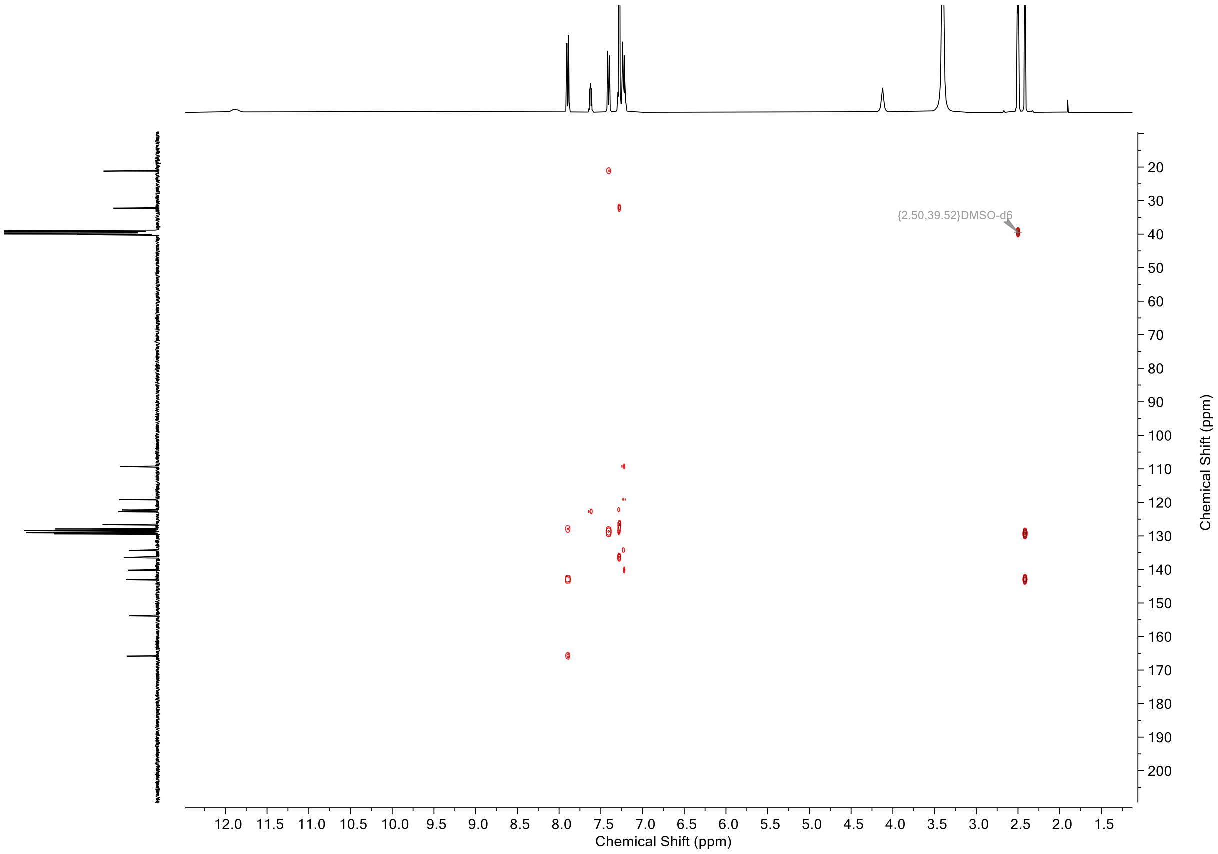

Figure S277: HMBC (DMSO-d_6): 1*H*-*N*-(2-Benzyl-benzo[*d*]imidazol-1-yl)-4-methylbenzamide (**3v**).

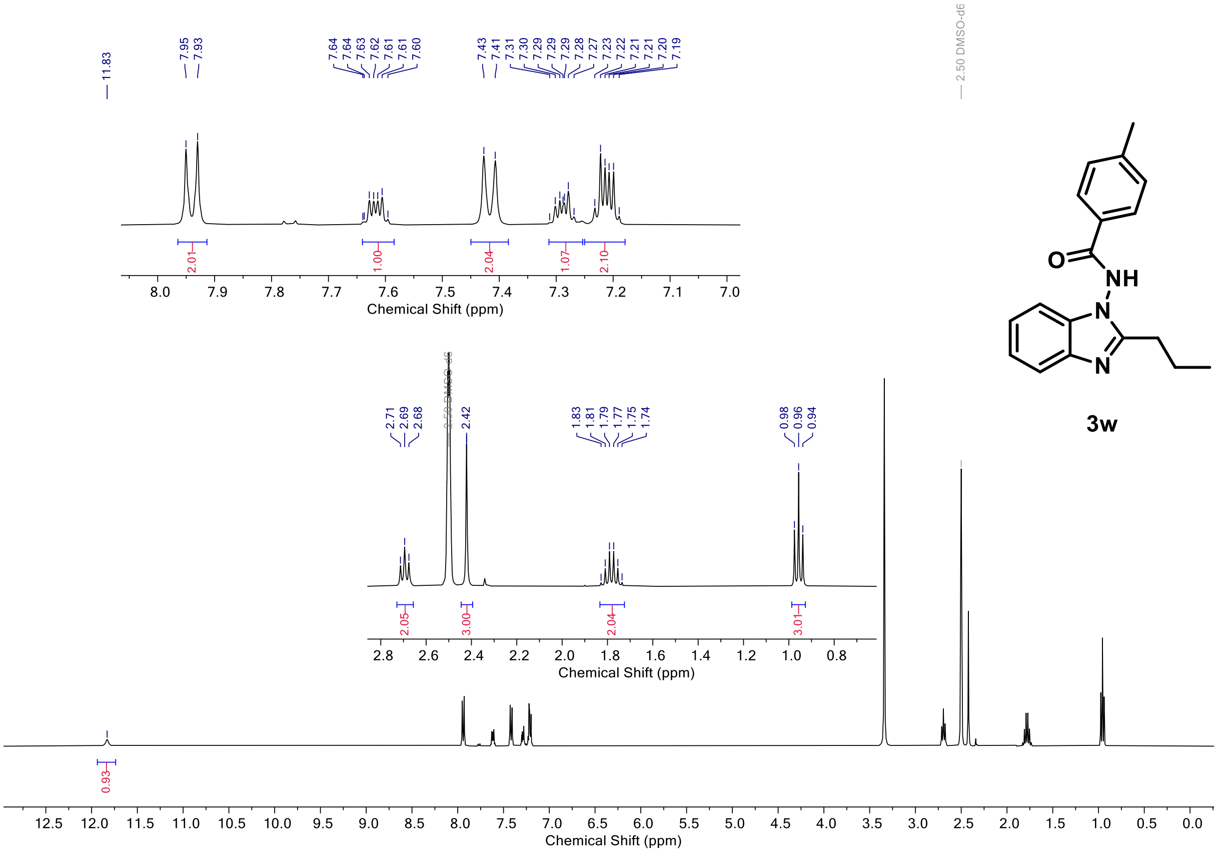

Figure S278: ^{1}H NMR (400 MHz, DMSO-d_6): 1*H*-4-Methyl-*N*-(2-propyl-benzo[*d*]imidazol-1-yl)benzamide (**3w**).

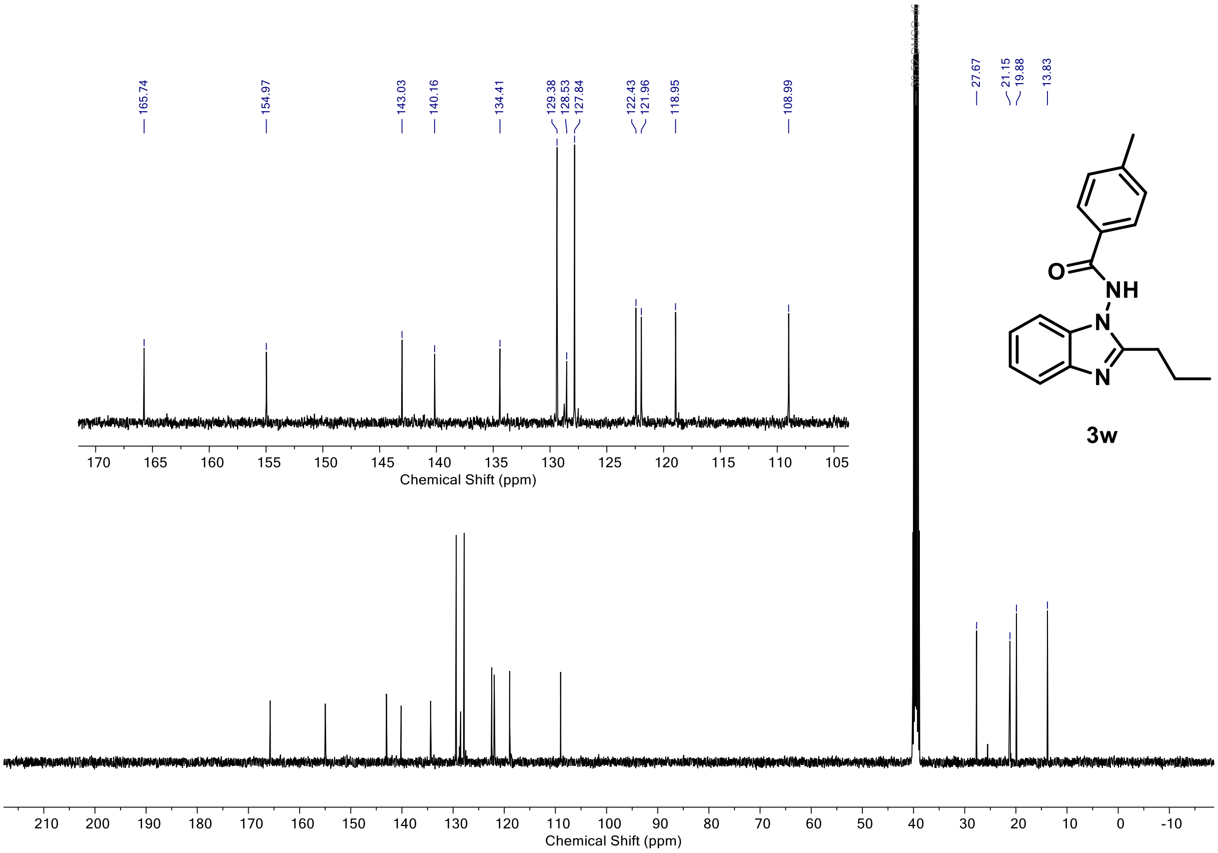

Figure S279: ^{13}C{^{1}H} NMR (101 MHz, DMSO-d_6): 1*H*-4-Methyl-*N*-(2-propyl-benzo[*d*]imidazol-1-yl)benzamide (**3w**).

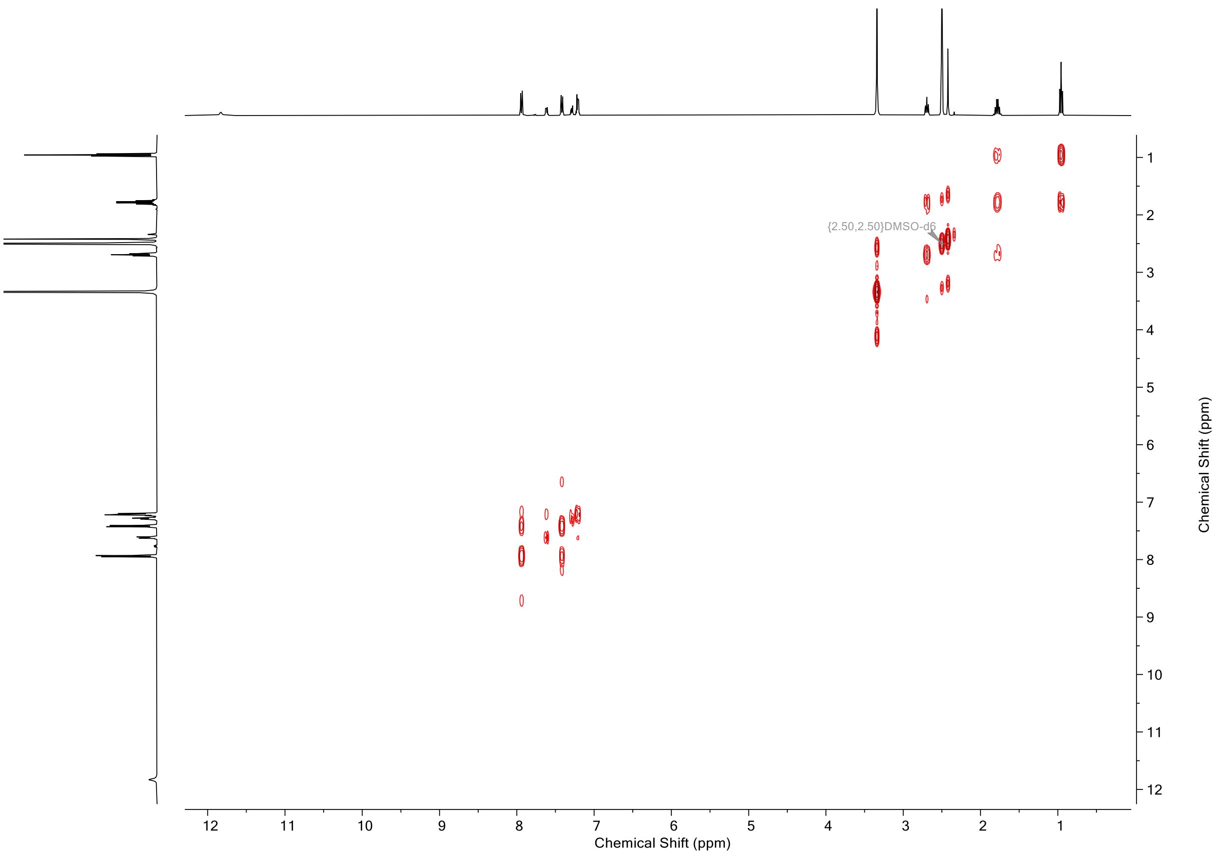

Figure S280: COSY (DMSO-d_6): 1*H*-4-Methyl-*N*-(2-propyl-benzo[*d*]imidazol-1-yl)benzamide (**3w**).

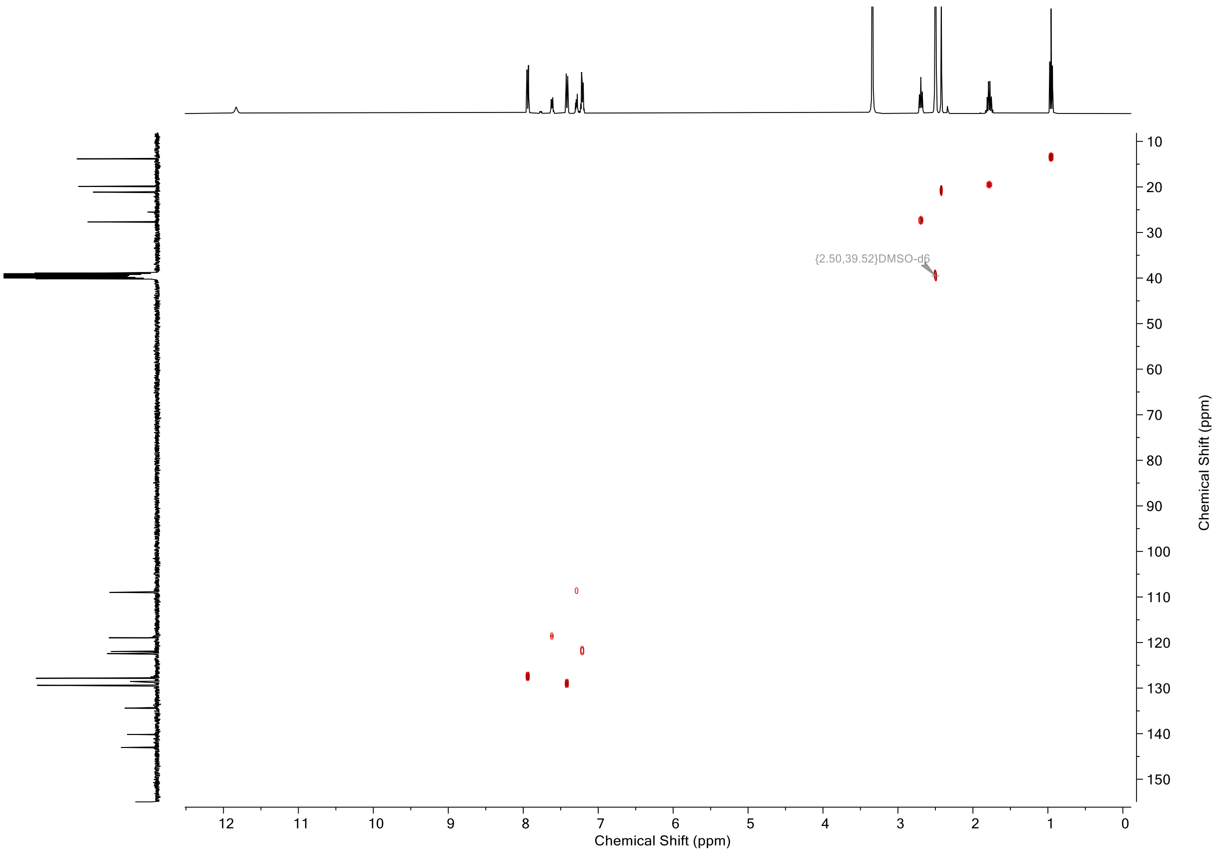

Figure S281: HSQC (DMSO-d_6): 1*H*-4-Methyl-*N*-(2-propyl-benzo[*d*]imidazol-1-yl)benzamide (**3w**).

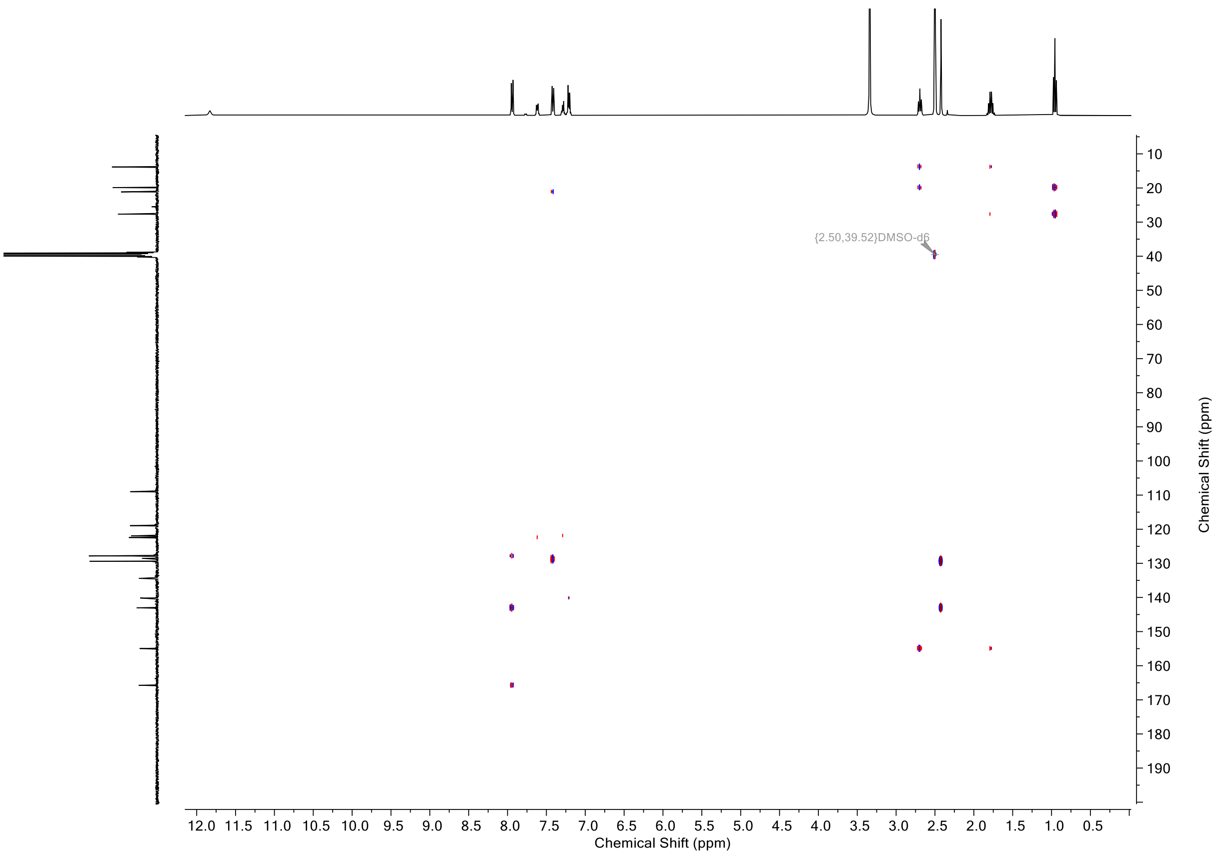

Figure S282: HMBC (DMSO-d_6): 1*H*-4-Methyl-*N*-(2-propyl-benzo[*d*]imidazol-1-yl)benzamide (**3w**).
